# Targeted Library of Phosphonic-Type Inhibitors of Human Neutrophil Elastase

**DOI:** 10.3390/molecules29051120

**Published:** 2024-03-01

**Authors:** Karolina Torzyk-Jurowska, Jaroslaw Ciekot, Lukasz Winiarski

**Affiliations:** 1 Division of Organic and Medicinal Chemistry, Faculty of Chemistry, Wroclaw University of Science and Technology, Wybrzeze Wyspianskiego 27, 50-370 Wroclaw, Poland; karolina.torzyk@pwr.edu.pl; 2 Department of Experimental Oncology, Hirszfeld Institute of Immunology and Experimental Therapy, Polish Academy of Sciences, 53-114 Wroclaw, Poland; jaroslaw.ciekot@hirszfeld.pl

**Keywords:** neutrophil elastase, proteinase 3, α-aminoalkylphosphonates

## Abstract

Despite many years of research, human neutrophil elastase (HNE) still remains an area of interest for many researchers. This multifunctional representative of neutrophil serine proteases is one of the most destructive enzymes found in the human body which can degrade most of the extracellular matrix. Overexpression or dysregulation of HNE may lead to the development of several inflammatory diseases. Previously, we presented the HNE inhibitor with k_inact_/K_I_ value over 2,000,000 [M^−1^s^−1^]. In order to optimize its structure, over 100 novel tripeptidyl derivatives of α-aminoalkylphosphonate diaryl esters were synthesized, and their activity toward HNE was checked. To confirm the selectivity of the resultant compounds, several of the most active were additionally checked against the two other neutrophil proteases: proteinase 3 and cathepsin G. The developed modifications allowed us to obtain a compound with significantly increased inhibitory activity against human neutrophil elastase with high selectivity toward cathepsin G, but none toward proteinase 3.

## 1. Introduction

Neutrophils are phagocytic granulocytes which are part of the innate immune system and constitute the first line of defence against pathogenic microorganisms. Inflammatory stimulation by bacteria or fungi induces the recruitment of these granulocytes to the inflammation sites [1]. Neutrophils attack an invading pathogen with an oxidative burst mainly based on the production of reactive oxygen species (ROS). In addition, they can also release antimicrobial peptides and a set of structurally related serine proteases, namely cathepsin G (CatG), neutrophil elastase (NE), proteinase 3 (PR3), and neutrophil serine proteinase 4 (NSP4). Release of antimicrobial peptides and neutrophil serine proteinases (NSPs) constitutes a non-oxidative mechanism of action [2,3]. Human neutrophil elastase (HNE, EC 3.4.21.37), also known as human leukocyte elastase (HLE), was first described by Janoff and Scherer in 1968 [4]. This enzyme is stored in azurophil (primary) granules of neutrophils. HNE belongs to the chymotrypsin-like serine protease superfamily. Next to other NSPs, it is also one of the most destructive enzymes found in the human body. It can degrade almost every element of the extracellular matrix (ECM), including elastin, proteoglycans, collagen, fibronectin, and laminin [4,5,6,7,8]. The activity of neutrophil elastase is not only degradative or antibacterial. It plays also an important role in the regulation of local inflammatory response [9]. Under normal physiological conditions, the heightened activity of neutrophil elastase is strictly regulated to avoid tissue degradation. Serpins, such as α1-antitrypsin (α1-AT), secretory leukocyte peptidase inhibitor (SLPI), and α2-macroglobulin (α2-MG) are natural endogenous serine protease inhibitors [10,11]. The unrestricted activity of elastase may lead to the development of the pathophysiological states including lung diseases such as chronic obstructive pulmonary disease (COPD), acute lung injury (ALI) and acute respiratory distress syndrome (ARDS), pulmonary emphysema, cystic fibrosis, or chronic bronchitis [12,13,14].

Diaryl esters of α-aminoalkylphosphonates and their peptidyl derivatives are known as highly specific and highly selective irreversible inhibitors of serine proteinases. The relatively easy synthetic approach to α-aminoalkylphosphonate diphenyl esters was described for the first time by Oleksyszyn et al. in 1979. Briefly, triphenyl phosphite undergoes an amidoalkylation reaction with benzyl carbamate and an appropriate aldehyde in acetic acid, affording α-aminophosphonate as a racemic mixture of both enantiomers [15]. It was only in 1989 that their ability to irreversibly inactivate serine proteases by acting as the transition state analogues was confirmed. Interactions between the inhibitor and active site serine residue lead to the formation of a covalent protease–inhibitor complex [16]. The most significant advantages of α-aminophosphonic-type inhibitors are their non-reactivity with cysteine, threonine, aspartyl, and metalloproteinases, along with their great stability in an aqueous solution including human plasma [17,18]. Importantly, α-aminophosphonic inhibitors do not show toxicity in vivo [19]. Therefore, many peptidyl derivatives of α-aminoalkylphosphonate diphenyl esters have been reported to act as effective irreversible inhibitors against several mammalian serine proteases, including trypsin, thrombin, uPA, granzymes, kallikreins, dipeptidyl peptidase IV, elastase, cathepsin G, and chymase, as well as bacterial (subtilisin, V8), and viral proteases (hepatitis C virus NS3/4A protease, West Nile virus NS2B/NS3 protease) [18,20,21,22,23,24,25,26,27,28].

**Scheme 1:**
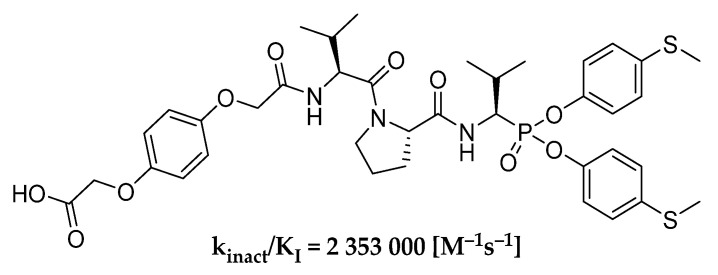
Structure of most active compound obtained in previous work.

In our previous works, we checked the preferable group for P1 position for simple α-aminophosphonate diaryl esters as inhibitors of human neutrophil elastase and the most active substituent into the ester ring [24]. As a result of these studies, we obtained the HNE inhibitor (Scheme 1), whose second-order rate constant of inhibition reaction (k_inact_/K_I_) is over 2,000,000 M^−1^s^−1^. We believe that further modifications of the previously described structure can improve kinetic parameters of designed peptydyl derivatives of 1-aminoalkylphosphonate diaryl esters toward HNE. The results presented by Kasperkiewicz et al. for the library of human neutrophil elastase coumarin substrates show that the introduction of unnatural amino acids could highly improve the potency of action [29].

To create a diverse library of over a hundred new phosphonic-type inhibitors of human neutrophil elastase, we designed peptydyl derivatives containing diaryl ester of 1-aminoalkylphosphonate as a warhead. Furthermore, we selected a group of proteinogenic and non-proteinogenic amino acids, both l and d configurations, and also a few amino acids with protective groups on the side chain (benzyl (Bzl) and benzyloxycarbonyl (Z)), as substrates for the synthesis of phosphonic tripeptides. We applied a classical solution chemical synthesis approach in the presence of *O*-(7-azabenzotriazol-1-yl)-*N*,*N*,*N′*,*N′*-tetramethyluronium hexafluorophosphate as a coupling reagent. The obtained library was then used in optimization studies to find the best sequence (positions P1–P3) showing the most potent inhibitory activity against human neutrophil elastase. The inhibitory potential was defined by enzyme kinetic studies, in the presence of a specific fluorogenic substrate for HNE (MeO-Suc-AAPV-AFC), and presented as the k_inact_/K_I_ value which constitutes a crucial kinetic parameter to identify covalent inhibitors.

Next, a few selected structures with the best potency toward HNE were tested in similar enzyme kinetic studies but in the presence of two other NSPs: proteinase 3 and cathepsin G, to determine their selectivity of action. The final step of our research included modifying the best candidate for a new peptydyl phosphonic-type inhibitor of HNE by introducing a hydroquinone moiety into the structure and separating the obtained racemic mixture into optically pure compounds. This division made it possible to compare the inhibitory potential against HNE, PR3, and CatG of individual diastereoisomers with configuration R or S at P1 position.

## 2. Results and Discussion

At the beginning, we decided to synthesize phosphonic tripeptides with the general formula Boc-(l)-Val-AA_1_-Val^P^(OC_6_H_4_-S-CH_3_)_2_ to check their influence on human neutrophil elastase inhibitory activity of different amino acid residue structures at P2 position. The inhibitory activity of 53 compounds was determined by enzyme kinetic studies in the presence of a MeO-Suc-AAPV-AFC as substrate for HNE and is presented in the table below as the k_inact_/K_I_ ratio (Table 1). All values were then individually compared with the reference compound (**27**, Boc-(l)-Val-(l)-Pro-Val^P^(OC_6_H_4_-S-CH_3_)_2_) showing the highest inhibitory potential toward HNE, confirmed in our previous work [24]. It was assumed that all compounds with higher values of the second-order rate constant of inhibition reaction (k_inact_/K_I_) than the reference compound had better activity and inhibitory potential against HNE, whereas all compounds showing lower k_inact_/K_I_ values were not good candidates for further optimization. 

All structures containing amino acids with d configuration at the P2 position presented above showed inappreciable or no inhibitory activity against HNE. However, this result was not dependent on the relative configuration since no significant activity was observed for most of the l-amino acids used in this iteration. The presence of both aliphatic and aromatic residues as well as elongation of the aliphatic side chain of the P2 amino acid residue caused a decrease in inhibitory activity against HNE and lower k_inact_/K_I_ values compared to the reference compound **27**. Among all of the obtained peptides with the sequence Boc-l-Val-AA_1_-Val^P^(OC_6_H_4_-S-CH_3_)_2_, compound **2** (Boc-l-Val-l-Ala-Val^P^(OC_6_H_4_-4-S-CH_3_)_2_) displayed the highest potency (k_inact_/K_I_ = 160,000 [M^−1^s^−1^]) and was the most active HNE inhibitor in this series. Unfortunately, none of the tested modifications displayed higher activity than the reference compound **27** (Boc-l-Val-l-Pro-Val^P^(OC_6_H_4_-4-S-CH_3_)_2_; k_inact_/K_I_ = 229,000 [M^−1^s^−1^]). Surprisingly, compound **29** (Boc-l-Val-l-Oic-Val^P^(OC_6_H_4_-4-S-CH_3_)_2_; k_inact_/K_I_ = 35,800 [M^−1^s^−1^])) had significantly lower activity against HNE than the reference compound **27**. Results for the library of human neutrophil elastase coumarin substrates presented by Kasperkiewicz et al. show that the substrate with l-Oic residue in the P2 position is around 60% more active than the substrate with l-Pro [29]. Probably these differences between our results and the results for coumarin substrates are caused by the differences in the reactive group structure. We chose the valine residue in P1 position, which is more bulky than the alanine residue presented in P1 position in the mentioned coumarin substrate library. This valine residue could cause steric interaction with l-Oic residue, which reduces alignment to the active site of the enzyme and decreases the inhibitory activity of compound **29** against human neutrophil elastase. Taking into account the information from further studies on the optimization of the P3 position, it was decided to choose the l-Pro residue in the P2 position as the most promising structure.

Next, the 56 compounds with the general formula Boc-AA_2_-(l)-Pro-Val^P^(OC_6_H_4_-S-CH_3_)_2_ were synthesized using an approach analogous to that of the first iteration for optimization of the P2 position, and their activity against human neutrophil elastase was checked by the same enzyme kinetic assay for HNE. The inhibitory potential of all phosphonic peptides was presented as the k_inact_/K_I_ [M^−1^s^−1^] value (Table 2), and once again all results were compared to the reference compound (**27**).

Investigating the results from Table 2 for the l and d pair of the same amino acid, it can be seen that for most cases the presence of the d configuration at P3 position causes a decrease in activity of the inhibitors. The exceptions are five compounds with d-amino acids: d-Pip, d-Nip, d-HPhe, d-Cys(Bzl) and d-Lys(Z) at P3 position, for which slightly better activity was observed compared to their l counterparts. Despite this, it is worth emphasizing that for all compounds containing d-amino acids, the k_inact_/K_I_ values were lower than those for the reference compound **27** (Boc-l-Val-l-Pro-Val^P^(OC_6_H_4_-4-S-CH_3_)_2_; k_inact_/K_I_ = 229,000 [M^−1^s^−1^]).

The highest activity was observed for compound **80** (Boc-d-Gln-l-Pro-Val^P^(OC_6_H_4_-4-S-CH_3_)_2_, k_inact_/K_I_ = 81,780 [M^−1^s^−1^]). These results clearly indicate that the human neutrophil elastase prefers L-amino acid residues at the P3 position, which coincides with the findings described above for the optimization of the P2 position. Secondly, the presence of protective groups, such as benzyl (Bzl) and benzyloxycarbonyl (Z), in the side chain had a negative effect and also reduced the inhibitory potential of peptidyl derivatives of α-aminoalkylphosphonate diphenyl esters toward HNE, compared to the reference **27**. From this group, compound **100** (Boc-l-Thr(Bzl)-l-Pro-Val^P^(OC_6_H_4_-4-S-CH_3_)_2_; k_inact_/K_I_ = 101,800 [M^−1^s^−1^]) showed the highest potential, but it was still 2.2× lower than the potential of compound **27**. Two other structures containing non-proteinogenic amino acids in P3 position, **62** (Boc-l-Abu-l-Pro-Val^P^(OC_6_H_4_-4-S-CH_3_)_2_; k_inact_/K_I_ = 227,200 [M^−1^s^−1^]) and **65** (Boc-l-Nva-l-Pro-Val^P^(OC_6_H_4_-4-S-CH_3_)_2_; k_inact_/K_I_ = 209,000 [M^−1^s^−1^]), had a level of inhibitory activity similar to that of **27**. Finally, better results than with the reference **27** were obtained for two phosphonic peptides with proteinogenic amino acids (methionine or glutamine) in P3, compound **75** (Boc-l-Met-l-Pro-Val^P^(OC_6_H_4_-4-S-CH_3_)_2_; k_inact_/K_I_ = 304,300 [M^−1^s^−1^]) and **79** (Boc-l-Gln-l-Pro-Val^P^(OC_6_H_4_-4-S-CH_3_)_2_; k_inact_/K_I_ = 743,300 [M^−1^s^−1^]). 

The introduction of the L-methionine at the P3 position resulted in increased inhibitory activity against HNE by almost 50% (k_inact_/K_I_ = 304,300 [M^−1^s^−1^]). The comparison of the inhibitory potential between compounds **70** (Boc-l-Nle-l-Pro-Val^P^(OC_6_H_4_-4-S-CH_3_)_2_; k_inact_/K_I_ = 125,250 [M^−1^s^−1^]) and **75** (Boc-l-Met-l-Pro-Val^P^(OC_6_H_4_-4-S-CH_3_)_2_; k_inact_/K_I_ = 304,300 [M^−1^s^−1^]) is also interesting due to the very similar structure of both inhibitors in that they have a sulfur atom instead of a CH_2_ group. Despite this similarity, there is a significant difference in inhibition: compound **75** shows 2.4× better activity against HNE than **70**. The gradual oxidation of the sulfur atom from the methionine side chain resulted in a 3.4× (Boc-l-Met(O)-l-Pro-Val^P^(OC_6_H_4_-4-S-CH_3_)_2_; k_inact_/K_I_ = 773,400 [M^−1^s^−1^]) and 7.1× (Boc-l-Met(O_2_)-l-Pro-Val^P^(OC_6_H_4_-4-S-CH_3_)_2_; k_inact_/K_I_ = 1,626,200 [M^−1^s^−1^]) increase in potency of action against HNE compared to the reference compound (k_inact_/K_I_ = 229,000 [M^−1^s^−1^]). It is worth noting a similar activity towards HNE of compounds **77** (Boc-l-Met(O)-l-Pro-Val^P^(OC_6_H_4_-4-S-CH_3_)_2_; k_inact_/K_I_ = 773,400 [M^−1^s^−1^]) and **79** (Boc-l-Gln-l-Pro-Val^P^(OC_6_H_4_-4-S-CH_3_)_2_; k_inact_/K_I_ = 743,300 [M^−1^s^−1^]), for which we can find significant similarities in the structure of the P3 residue. This shows how important the presence of an oxygen atom in this specific position of the side chain is for the interaction with the P3 pocket of HNE.

In order to verify the selectivity of the most active compounds of both groups, measurements of inhibitory activity against two other serine proteases, proteinase 3 and cathepsin G, were made. These enzymes are major representatives of neutrophil serine proteases (NSPs) which are closely related to human neutrophil elastase. Proteinase 3 is known as a close homologue of HNE with a very similar substrate specificity to small aliphatic residues, whereas cathepsin G presents a slightly different predisposition to binding hydrophobic (Phe or Leu) and basic (Lys or Arg) side chains at the S1 pocket [30]. From the created library of phosphonic-type inhibitors of human neutrophil elastase, the best candidates with the highest value of second-order rate constant of inhibition reaction were selected: five from the first iteration (compounds **1**, **2**, **6**, **8** and **29**) and five from the second iteration (compounds **62**, **75**, **77**, **78** and **79**). The inhibitory potential of these compounds was tested in analogous enzymatic assays in the presence of PR3 or CatG instead of HNE, and their activity (k_inact_/K_I_ [M^−1^s^−1^]) toward all three NSPs was compared to the reference compound **27** (Table 3 and Table 4).

For all compounds, a complete lack of inhibition or nonsignificant inhibitory effect (k_inact_/K_I_ < 50 M^−1^s^−1^) on the proteolytic activity of cathepsin G was observed (Table 3 and Table 4). Newly developed phosphonic-type inhibitors showed high selectivity toward CatG, so they can be successfully used to selectively inhibit only HNE, without any interaction with CatG present in the analysed sample. This result is probably caused by differences in the structure of active sites and dissimilar substrate specificity of HNE and CatG.

Unfortunately, an absolutely opposite effect was observed in the presence of proteinase 3. In this case, all of the tested inhibitors were recognized by the PR3 and specifically bound in its active site. Depending on the compound used, the observed inhibition of proteolysis was at various levels, as reflected by k_inact_/K_I_ values from almost 43,000 to more than 2,300,000 M^−1^s^−1^ (Table 3 and Table 4). Comparing the inhibitory potential towards HNE and PR3 for almost all selected inhibitors, except compounds **2** and **6,** showed higher specificity against proteinase 3. The high structural similarity of HNE and PR3 indicated a possible lack of selectivity of action, but such a relevant preference for PR3 was surprising. The three most potent phosphonic-type inhibitors of human neutrophil elastase (**79**, **77** and **78**) were approximately twice as powerful inhibitors of proteinase 3.

As the last part of the inhibitor structure optimization, we decided to introduce the hydroquinone moiety, used in previous research, on the *N*-terminal of compound **78**. It is important to note that all of the tested compounds were mixtures of two diastereoisomers, whereas the leading structure was tested as a single diastereoisomer. For this reason, an attempt was made to separate compound **111** into pure diastereoisomers. It was possible to obtain both diastereoisomers in pure form (**112, 113**). The previous studies led us to assume that the more active compound **113** has the R configuration at the P1 position, and the less active compound **112** has the S configuration [31,32,33]. The activity toward HNE, PR3 and CatG was inspected for all three compounds. The results are summarized in Table 5.

As we expected, the introduction of hydroquinone moiety to the structure of compound **78** (Boc-l-Met(O)_2_-l-Pro-Val^P^(OC_6_H_4_-S-CH_3_)_2_) resulted in a significant increase in the inhibitory potential of the peptidyl derivatives of α-aminoalkylphosphonate diaryl esters **111** towards HNE (k_inact_/K_I_ = 2,319,700 [M^−1^s^−1^]) and PR3 (k_inact_/K_I_ = 4,105,900 [M^−1^s^−1^]), with a simultaneous lack of activity against CatG (k_inact_/K_I_ < 50 [M^−1^s^−1^]). The results confirm the established higher inhibition of a single diastereoisomer, with the R configuration at the P1 position. The isolation of an optically pure compound **113** led to an increase in inhibitory activity against both proteases, human neutrophil elastase (k_inact_/K_I_ = 4,236,800 [M^−1^s^−1^]) and proteinase 3 (k_inact_/K_I_ = 8,951,600 [M^−1^s^−1^]), compared to the mixture (S,R) **111**. Similarly to the previous results, it was also noticed that these compounds showed definitely higher specificity towards PR3 than HNE. The chemical modification used in the described studies did not increase the selectivity of the action against human neutrophil elastase. Nevertheless, the remarkably high inhibitory activity confirmed by enzymatic kinetic assays makes these structures serious candidates for further structure–activity relationship studies in the discovery of new, highly specific and selective neutrophil serine protease inhibitors.

We presented a library of over 100 new phosphonic-type inhibitors of the human neutrophil elastase containing different proteinogenic and non-proteinogenic amino acid residues at P2 and P3 positions. The peptidyl derivatives of α-aminoalkylphosphonate diaryl esters show high specificity and great potential to act as human neutrophil elastase inhibitors, which was confirmed in kinetic studies. The top three most potent structures of all phosphonic tripeptides were **78** (Boc-l-Met(O)_2_-l-Pro-Val^P^(OC_6_H_4_-S-CH_3_)_2_; k_inact_/K_I_ = 1,626,200 [M^−1^s^−1^]), **77** (Boc-l-Met(O)-l-Pro-Val^P^(OC_6_H_4_-S-CH_3_)_2_; k_inact_/K_I_ = 773,400 [M^−1^s^−1^]) and **79** (Boc-l-Gln-l-Pro-Val^P^(OC_6_H_4_-S-CH_3_)_2_; k_inact_/K_I_ = 743,300 [M^−1^s^−1^]). A small modification of the best structure **78** led to the creation of new hydroquinone derivatives **111** and **113** showing, respectively, 1.4× (k_inact_/K_I_ = 2,319,700 [M^−1^s^−1^]) and 2.6× (k_inact_/K_I_ = 4,236,800 [M^−1^s^−1^]) higher inhibitory activity against HNE than the initial compound. 

Although these inhibitors do not show activity against cathepsin G, they have an excellent ability to inhibit the activity of proteinase 3. The lack of selectivity of action directed towards only one neutrophil serine protease does not exclude the possible use of these inhibitors in further research or therapy. The presented phosphonic-type compounds can be successfully applied as dual inhibitors of human neutrophil elastase and proteinase 3 in some chronic neutrophilic inflammatory diseases when it is required to stop the activity of both proteases [34]. The latest literature reports affirm that structures **111** and **113** can be some of the most powerful irreversible inhibitors which bind covalently to the active site of proteinase 3, with k_inact_/K_I_ values of 4,105,900 [M^−1^s^−1^] and 8,951,600 [M^−1^s^−1^], respectively [35].

## 3. Materials and Methods

All chemical reagents and solvents were taken from commercial sources and used without purification. High-performance liquid chromatography analyses were carried out on a Waters Binary Module System (Waters, Milford, MA, USA) with a dual λ absorbance detector system fitted with a Supelco Ascentis C8 HPLC column (250 mm  ×  4.6 mm, 5 µm, Supelco, Bellefonte, PA, USA) with a 1 mL/min flow rate operating on a linear gradient from 5 to 95% B within 20 min (solvent A: H_2_O with 0.05% trifluoroacetic acid, solvent B: MeCN with 0.05% trifluoroacetic acid). High-performance liquid chromatography purification was carried out in a Varian ProStar system (Varian, Australia) with a dual λ absorbance detector system with the application of a Supelco Ascentis C8 HPLC column (250 mm  ×  21.2 mm, 5 µm) with a 15 mL/min flow rate and a linear gradient from 20 to 100% B within 20 min or 40 to 100% B within 20 min (depending on compound solubility), solvent A: H_2_O with 0.05% trifluoroacetic acid, solvent B: MeCN with 0.05% trifluoroacetic acid. The nuclear magnetic resonance spectra (^1^H and ^31^P) were recorded on a 400 MHz NMR Jeol PCZ 400S or Bruker Avance 600 MHz (Bruker, Billerica, MA, USA). High resolution mass spectra were recorded with a Brucker micro TOF-Q II (Bruker, Billerica, MA, USA).

### 3.1. Chemistry

**Scheme 2:**
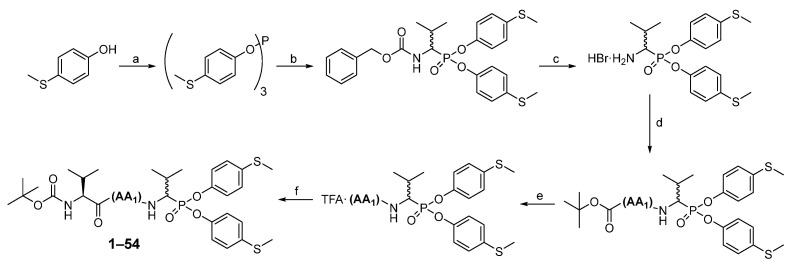
Synthesis of peptidyl derivatives of α-aminoalkylphosphonate diphenyl esters with different amino acid residues at the P2 position. (a) PCl_3_, MeCN, reflux, 3 h; (b) benzyl carbamate, (CH_3_)_2_CHCHO, AcOH, 80−90 °C, 2 h; (c) 33% HBr/AcOH, 2 h, RT; (d) *N*-Boc-(AA_1_)-OH, HATU, DIPEA, MeCN, 12 h, RT; (e) 33% TFA/DCM (*v*/*v*), 2 h, RT; (f) *N*-Boc-(l)-Val-OH, HATU, DIPEA, MeCN, 12 h, RT.

The first step in producing tripeptide derivatives of diaryl esters of α-aminoalkanephosphonic acids with the sequence Boc-(l)-Val-AA_1_-Val^P^(*O*-C_6_H_4_-4-SCH_3_)_2_ was the reaction between 4-(methylmercapto)phenol and phosphorus (III) chloride in boiling acetonitrile (Scheme 2). The resultant tri-(4-methylsulfidophenyl) phosphite was subjected to an α-amidoalkylation reaction with the aid of benzyl carbamate and isobutyraldehyde in acetic acid. The Cbz protecting group was then removed with a 33% HBr solution in acetic acid. The product was crystallized in methanol/diethyl ether and then subjected to a coupling reaction with appropriate Boc amino acid (AA_1_) using *O*-(7-Azabenzotriazol-1-yl)-*N*,*N*,*N′*,*N′*-tetramethyluronium hexafluorophosphate (HATU) as a coupling reagent. Washing (5% NaHCO_3_, 5% citric acid, brine) and purification in HPLC yielded pure phosphonic dipeptide. Next, the Boc protecting group was removed with a 33% solution of trifluoroacetic acid in dichloromethane, and the product was paired with the Boc-(l)-Val-OH, also employing HATU as a coupling reagent. After extraction and purification in HPLC, pure phosphonic tripeptide was obtained.

**Scheme 3:**
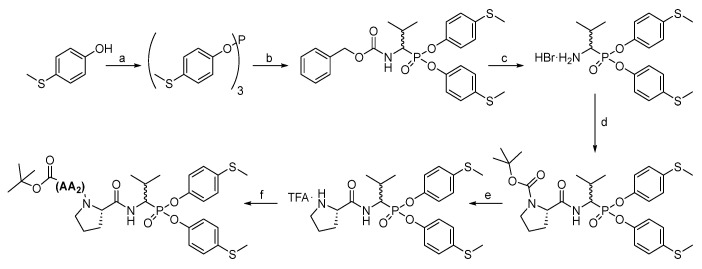
Synthesis of peptidyl derivatives of α-aminoalkylphosphonate diphenyl esters with different amino acid residues at the P3 position. (a) PCl_3_, MeCN, reflux, 3 h; (b) benzyl carbamate, (CH_3_)_2_CHCHO, AcOH, 80−90 °C, 2 h; (c) 33% HBr/AcOH, 2 h, RT; (d) *N*-Boc-(l)-Pro-OH, HATU, DIPEA, MeCN, 12 h, RT; (e) 33% TFA/DCM (*v*/*v*), 2 h, RT; (f) *N*-Boc-(AA_2_)-OH, HATU, DIPEA, MeCN, 12 h, RT.

A similar approach (Scheme 3) produced tripeptides with the Boc-AA_2_-(l)-Pro-Val^P^(OC_6_H_4_-S-CH_3_)_2_ sequences. Briefly, after removing the Cbz group from Cbz-Val^P^(*O*-C_6_H_4_-SCH_3_)_2_, Boc-(l)-Pro-OH was attached, with HATU as a coupling reagent, to the resulting compound, and then the Boc protecting group was removed with a 33% solution of trifluoroacetic acid in dichloromethane, and the appropriate Boc amino acid (AA_2_) was attached with HATU as a coupling reagent. Extraction and purification in HPLC resulted in pure phosphonic tripeptide.

**Scheme 4:**
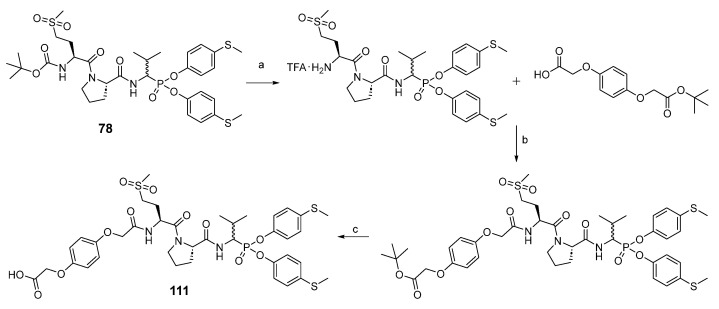
Synthesis of peptidyl derivatives of α-aminoalkylphosphonate diphenyl esters containing a hydroquinone moiety. (a) 33% TFA/DCM (*v*/*v*), 2 h, RT; (b) HATU, DIPEA, MeCN, 12 h, RT; (c) 33% TFA/DCM (*v*/*v*), 2 h, RT.

For the synthesis of peptidyl derivatives of α-aminoalkylphosphonate diphenyl esters containing a hydroquinone moiety (Scheme 4), first the Boc protecting group was removed from compound **78**, and a hydroquinone moiety, synthesized as described previously, was attached with HATU as a coupling reagent [24]. After extraction, the tert-butyl group was removed with a 33% solution of trifluoroacetic acid in dichloromethane. The crude product was purified in HPLC.

**Scheme 5:**
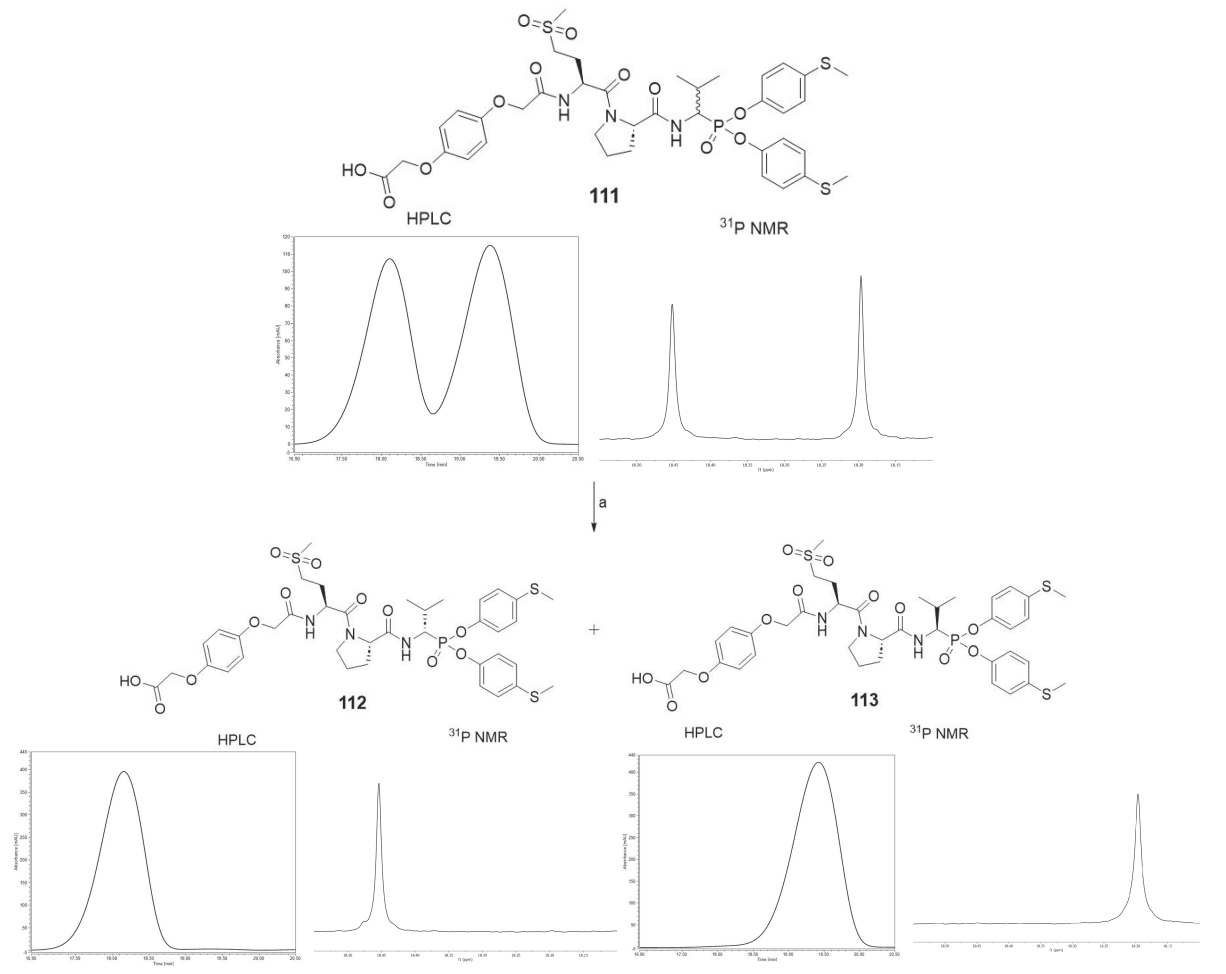
Obtaining peptidyl derivatives of α-aminoalkylphosphonate diphenyl esters containing a hydroquinone moiety as a pure diastereoisomers. (a) HPLC.

The HPLC method served the purpose of separating pure diastereoisomers of compound **111**. The separation was carried out at constant concentration of acetonitrile (40%) in water. After freeze-drying, the purity was confirmed by ^31^P NMR and analytical HPLC (Scheme 5).

*(tert-butyl ((2S)-1-((2S)-2-((1-(bis(4-(methylthio)phenoxy)phosphoryl)-2-methylpropyl)carbamoyl)pyrrolidin-1-yl)-4-(methylsulfonyl)-1-oxobutan-2-yl)carbamate)* (**78**) was prepared by means of general methods described above and purified by HPLC with a 15 mL/min flow rate, with a linear gradient from 20 to 100% B within 20 min (solvent A: H_2_O with 0.05% trifluoroacetic acid, solvent B: MeCN with 0.05% trifluoroacetic acid) to yield a white solid (68%). ^31^P NMR (162 MHz, Chloroform-d) δ 18.19 (s, 45%), 17.79 (s, 55%). ^1^H NMR (400 MHz, Chloroform-d) δ 7.29 (dd, *J* = 142.1, 10.3 Hz, 1H, NH), 7.23–7.14 (m, 4H, 4×ArH), 7.09–6.95 (m, 4H, 4×ArH), 5.51 (dd, *J* = 25.8, 8.4 Hz, 1H, NH), 4.85–4.44 (m, 3H, CHP, 2×CH), 3.77–3.51 (m, 2H, CH_2_), 3.19–2.99 (m, 2H, CH_2_), 2.87 (d, *J* = 46.8 Hz, 3H, CH_3_), 2.47–2.42 (m, 6H, 2×SCH_3_), 2.43–2.11 (m, 3H, CH_2_, CH), 2.11–1.79 (m, 4H, 2×CH_2_), 1.41 (s, 9H, Boc), 1.11–0.98 (m, 6H, 2×CH_3_). HRMS calcd. for C_33_H_48_N_3_O_9_PS_3_ [M + H]^+^ 758.2369 found 758.2386. HPLC: purity >99%; retention time 16.750 and 17.007 min.

*(2-(4-(2-(((2S)-1-((2S)-2-((1-(bis(4-(methylthio)phenoxy)phosphoryl)-2-methylpropyl)carbamoyl)pyrrolidin-1-yl)-4-(methylsulfonyl)-1-oxobutan-2-yl)amino)-2-oxoethoxy)phenoxy)acetic acid)* (**111**), the TFA salt (0.21 g, 0.27 mmol) of 78 obtained after *N*-Boc deprotection with a 33% solution of trifluoroacetic acid in dichloromethane, was dissolved in acetonitrile (5 mL), and *N*,*N*′-diisopropylethylamine (139 μL, 0.81 mmol) was added. Next, 2-(4-(2-(tert-butoxy)-2-oxoethoxy)phenoxy)acetic acid (92 mg, 0.33 mmol) was blended into the mixture, which was followed by the addition of HBTU (124 mg, 0.33 mmol). The reaction was performed overnight at room temperature. All volatile ingredients were removed under reduced pressure, and the resulting oil was dissolved in ethyl acetate (50 mL). The solution was washed with 5% NaHCO_3_ (2 × 25 mL), 5% citric acid (2 × 25 mL), and brine (25 mL). After drying over anhydrous MgSO_4_, the solution was filtered and concentrated in a vacuum. The resulting crude oil was dissolved in a 33% solution of trifluoroacetic acid in dichloromethane (5 mL). After 2 h, all volatile ingredients were removed under reduced pressure, and the product was purified by HPLC with a 15 mL/min flow rate using a linear gradient from 20 to 100% B within 20 min (solvent A: H_2_O with 0.05% trifluoroacetic acid, solvent B: MeCN with 0.05% trifluoroacetic acid) to yield a 233 mg white solid (63%). ^31^P NMR (243 MHz, Methanol-d4) δ 18.45 (s, 47%), 18.20 (s, 53%). ^1^H NMR (601 MHz, MeOD) δ 7.21–6.60 (m, 13H, 12×ArH, NH), 4.87–4.81 (m, 1H, NH), 4.59–4.34 (m, 7H, 2×CH_2_, CHP, 2×CH), 3.75–3.59 (m, 2H, CH_2_), 3.10–2.98 (m, 2H, CH_2_), 2.83 (d, *J* = 43.0 Hz, 3H, CH_3_), 2.39–2.31 (m, 6H, 2×SCH_3_), 2.32–2.15 (m, 2H, 0.5×CH_2_, CH), 2.13–2.02 (m, 2H, CH_2_), 2.00–1.65 (m, 3H, 1.5×CH_2_), 1.07 (dd, *J* = 20.3, 6.9 Hz, 3H, CH_3_), 1.01 (dd, *J* = 31.6, 6.6 Hz, 3H, CH_3_). HRMS calcd. for C_38_H_48_N_3_O_12_PS_3_ [M + H]^+^ 866.2216 found 866.2238. HPLC: purity >99%; retention time 15.450 min.

Analytical data and structures of all synthesised compounds are presented in Supplementary Materials.

### 3.2. Enzyme Kinetics

**Figure 1:**
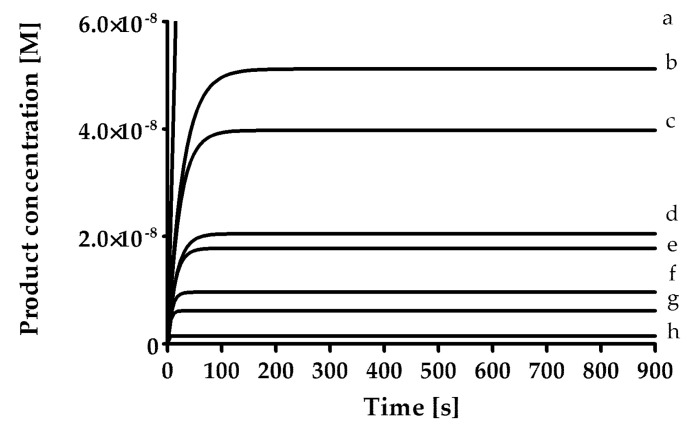
Progress curves for the inhibition of HNE (1.25 mU/mL) by compound **111**: (a) control; (b) 11.9 nM; (c) 13.9 nM; (d) 16.2 nM; (e) 18.9 nM; (f) 22 nM; (g) 25.7 nM; (h) 30 nM.

The rates of inhibition of human neutrophil elastase (HNE, 1.25 mU/mL; BioCentrum Ltd., Kraków, Poland), proteinase 3 (PR3, 7 nM, Elastin Products Company, Inc., Owensville, MO, USA) and cathepsin G (CatG, 20 nM; BioCentrum Ltd., Kraków, Poland) were measured in 0.1 M HEPES, 0.5 M NaCl, 0.03% Triton X-100 (pH 7.5) at 37 °C and with the use of fluorogenic substrate [HNE (MeO-Suc-AAPV-AFC; 40 μM); PR3 (MeO-Suc-AAPV-AFC, 125 μM); CatG (Suc-AAPF-AFC, 250 μM)]. As a positive control for all three enzymes, an α1-antitrypsin from human blood plasma (Cat. No. I-003, BioCentrum Ltd., Kraków, Poland) was used. The inhibitory activity of the synthesized compounds was determined by the progress curve method (Figure 1). Control curves in the absence of the inhibitor were linear. The standard deviation for presented values was calculated from the mean of three independent experiments and did not exceed 10%. All measurements were performed with the Spectra Max Gemini XPS spectrofluorometer (Molecular Devices, San Jose, CA, USA).

In the first phase of the test, screening was performed by measuring the inhibitory activity of each compound at a concentration of 25 µM, pre-incubated with the enzyme for 15 min. Then, for compounds that showed less than 5% inhibition, the lack of inhibitory properties was assumed, while for compounds inhibiting the enzyme in the range from 5% to 40%, it was assumed that the rate constant of the secondary reaction k_inact_/K_I_ was lower than 50 M^−1^s^−1^. For the remaining compounds, the exact k_inact_/K_I_ value was determined. The first-order rate constant (k_obs_) was determined for seven different inhibitor concentrations using Equation (1).

(1) $$\left[P\right]=\frac{v_{i}}{k_{obs}}\left[1-e^{\left(-k_{obs}t\right)}\right]$$

The secondary reaction rate constant k_inact_/K_I_ was determined using Equation (2).

(2) $$\frac{k_{obs}}{\left[I\right]}=\frac{k_{inact}}{K_{I}\left(1+\frac{\left[S\right]}{K_{M}}\right)}$$

## Figures and Tables

**Table 1:** Influence of the amino acid structure at P2 position of phosphonic peptides with the general formula Boc-(l)-Val-AA_1_-Val^P^(OC_6_H_4_-S-CH_3_)_2_ on the inhibitory activity toward human neutrophil elastase.

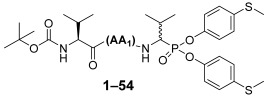					
**No.**	**AA_1_**	k_inact_/K_I_ [M^−1^s^−1^] *	**No.**	**AA_1_**	k_inact_/K_I_ [M^−1^s^−1^] *
**1**	Gly	53,300 ± 1900	**28**	d-Pro	<50
**2**	l-Ala	160,000 ± 5300	**29**	l-Oic	35,800 ± 1400
**3**	d-Ala	1820 ± 30	**30**	l-Pip	33,370 ± 2680
**4**	Aib	398 ± 26	**31**	d-Pip	NI
**5**	beta-Ala	<50	**32**	l-Tic	17,830 ± 1330
**6**	l-Abu	75,000 ± 2600	**33**	d-Tic	NI
**7**	d-Abu	<50	**34**	l-Nip	<50
**8**	l-Nva	56,300 ± 2800	**35**	l-Cpg	<50
**9**	d-Nva	<50	**36**	l-Chg	<50
**10**	l-Val	11,930 ± 570	**37**	d-Chg	<50
**11**	d-Val	NI	**38**	l-Phe	683 ± 51
**12**	l-Tle	NI	**39**	d-Phe	<50
**13**	d-Tle	NI	**40**	l-Cha	NI
**14**	l-Nle	26,070 ± 580	**41**	l-Igl	<50
**15**	d-Nle	NI	**42**	d-Igl	NI
**16**	l-Leu	9740 ± 630	**43**	l-Trp	<50
**17**	d-Leu	<50	**44**	d-Trp	NI
**18**	l-NptGly	<50	**45**	l-Asp(Bzl)	<50
**19**	l-Met	29,490 ± 590	**46**	d-Asp(Bzl)	NI
**20**	d-Met	<50	**47**	l-Cys(Bzl)	<50
**21**	l-Met(O)	21,620 ± 490	**48**	l-Glu(Bzl)	972 ± 63
**22**	l-Met(O_2_)	19,950 ± 490	**49**	d-Orn(Z)	<50
**23**	l-Ser(Bzl)	3580 ± 960	**50**	d-Tyr(Bzl)	NI
**24**	d-Ser(Bzl)	<50	**51**	l-Tyr(Bzl)	NI
**25**	l-Thr(Bzl)	<50	**52**	l-Lys(Z)	2040 ± 97
**26**	d-Thr(Bzl)	NI	**53**	d-Lys(Z)	NI
**27**	l-Pro	229,000 ± 4600	**54**	l-Hyp(Bzl)	8360 ± 398

* NI—no inhibition, **27**—Boc-(l)-Val-(l)-Pro-Val^P^(OC_6_H_4_-S-CH_3_)_2_—reference compound [24].

**Table 2:** Influence of amino acid structure at P3 position of phosphonic peptides with the general formula Boc-AA_2_-(l)-Pro-Val^P^(OC_6_H_4_-S-CH_3_)_2_ on the inhibitory activity toward human neutrophil elastase.

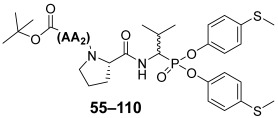					
**No.**	**AA_2_**	**k_inact_/K_I_ [M^−1^s^−1^]**	**No.**	**AA_2_**	**k_inact_/K_I_ [M^−1^s^−1^]**
**55**	Gly	40,100 ± 240	**83**	d-Pip	3690 ± 230
**56**	l-Ala	138,400 ± 4300	**84**	l-Nip	3615 ± 183
**57**	d-Ala	47,500 ± 2300	**85**	d-Nip	22,200 ± 157
**58**	Aib	1290 ± 33	**86**	1,4-*cis*-ACHC	1500 ± 130
**59**	AC3C	12,670 ± 260	**87**	1,4-*trans*-ACHC	12,840 ± 140
**60**	beta-Ala	11,400 ± 210	**88**	l-Cpg	105,860 ± 3500
**61**	GABA	13,520 ± 196	**89**	l-Phg	23,870 ± 1140
**62**	l-Abu	227,200 ± 9850	**90**	d-Phg	24,890 ± 600
**63**	d-Abu	32,320 ± 470	**91**	l-Chg	83,850 ± 4570
**64**	AC4C	4270 ± 230	**92**	d-Chg	29,340 ± 220
**65**	l-Nva	209,000 ± 3600	**93**	l-Phe	44,950 ± 2230
**66**	d-Nva	56,455 ± 2045	**94**	d-Phe	26,050 ± 460
**27** *	l-Val	229,000 ± 4600	**95**	l-Cha	20,670 ± 700
**67**	d-Val	11,600 ± 470	**96**	l-HPhe	57,050 ± 1360
**68**	l-Tle	102,350 ± 1920	**97**	d-HPhe	63,850 ± 2960
**69**	d-Tle	672 ± 44	**98**	l-Igl	45,980 ± 2800
**70**	l-Nle	125,250 ± 4790	**99**	d-Igl	19,000 ± 700
**71**	d-Nle	47,730 ± 2920	**100**	l-Thr(Bzl)	101,800 ± 4000
**72**	l-Leu	126,000 ± 2800	**101**	l-Cys(Bzl)	48,100 ± 1500
**73**	d-Leu	76,400 ± 3000	**102**	d-Cys(Bzl)	52,850 ± 1570
**74**	l-NptGly	78,800 ± 4300	**103**	l-Asp(Bzl)	79,000 ± 3500
**75**	l-Met	304,300 ± 14,600	**104**	d-Asp(Bzl)	22,100 ± 620
**76**	d-Met	63,650 ± 1690	**105**	l-Glu(Bzl)	26,300 ± 560
**77**	l-Met(O)	773,400 ± 60,000	**106**	l-His(Bzl)	42,750 ± 1190
**78**	l-Met(O)_2_	1,626,200 ± 114,200	**107**	l-Orn(Z)	67,050 ± 925
**79**	L-Gln	743,300 ± 39,700	**108**	d-Orn(Z)	60,250 ± 3200
**80**	d-Gln	81,780 ± 3570	**109**	l-Lys(Z)	55,100 ± 1500
**81**	l-Pro	1990 ± 77	**110**	d-Lys(Z)	75,700 ± 3200
**82**	l-Pip	1050 ± 82			

* **27**—Boc-(l)-Val-(l)-Pro-Val^P^(OC_6_H_4_-S-CH_3_)_2_—reference compound [24].

**Table 3:** Inhibition properties of selected Boc-(l)-Val-AA_1_-Val^P^(OC_6_H_4_-S-CH_3_)_2_ compounds toward human neutrophil elastase (HNE), proteinase 3 (PR3) and cathepsin G (CatG).

No.	AA_1_	k_inact_/K_I_ [M^−1^s^−1^]		
		HNE	PR3	CatG
**27**	l-Pro	229,000 ± 4600	605,700 ± 23,300	<50
**2**	l-Ala	160,000 ± 5300	128,200 ± 4600	<50
**6**	l-Abu	75,000 ± 2600	67,800 ± 2900	<50
**8**	l-Nva	56,300 ± 2800	64,500 ± 1900	NI
**1**	Gly	53,300 ± 1900	55,250 ± 1700	<50
**29**	l-Oic	35,800 ± 1400	42,900 ± 900	<50

**Table 4:** Inhibition properties of selected Boc-AA_2_-(l)-Pro-Val^P^(OC_6_H_4_-S-CH_3_)_2_ compounds toward human neutrophil elastase (HNE), proteinase 3 (PR3) and cathepsin G (CatG).

No.	AA_2_	k_inact_/K_I_ [M^−1^s^−1^]		
		HNE	PR3	CatG
**78**	l-Met(O)_2_	1,626,200 ± 114,200	2,324,000 ± 35,200	<50
**77**	l-Met(O)	773,400 ± 60,000	1,513,000 ± 12,700	<50
**79**	l-Gln	743,300 ± 39,700	1,258,300 ± 22,900	<50
**75**	l-Met	304,300 ± 14,600	405,500 ± 14,100	<50
**27**	l-Val	229,000 ± 4600	605,700 ± 23,300	<50
**62**	l-Abu	227,200 ± 9850	310,550 ± 3100	<50

**Table 5:** Inhibition properties of peptidyl derivatives of α-aminoalkylphosphonate diphenyl esters containing a hydroquinone moiety toward human neutrophil elastase (HNE), proteinase 3 (PR3) and cathepsin G (CatG).

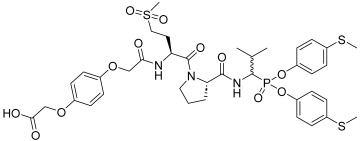				
**No.**	**Diastereoisomer**	**k_inact_/K_I_ [M^−1^s^−1^]**		
		**HNE**	**PR3**	**CatG**
**111**	S,R	2,319,700 ± 73,400	4,105,900 ± 176,000	<50
**112**	S	4200 ± 360	31,300 ± 1390	NI
**113**	R	4,236,800 ± 226,900	8,951,600 ± 332,400	<50
α1-antitrypsin		6,460,100 ± 332,600	18,841,600 ± 414,600	211,400 ± 11,000

## Data Availability

The data presented in this study are available on request from the corresponding author.

## Supplementary Materials

The following supporting information can be downloaded at: https://www.mdpi.com/article/10.3390/molecules29051120/s1, Structures of all used amino acids and analytical data of all final compounds.
