# Supplementary material for: Targeted Library of Phosphonic-Type Inhibitors of Human Neutrophil Elastase
Source: Molecules. 2024 Mar 1;29(5):1120. doi: 10.3390/molecules29051120 (PMC10934618; doi:10.3390/molecules29051120)
Supplement: Supplementary file 1 [file molecules-29-01120-s001.zip › molecules-2727743-supplementary.pdf]

Targeted library of phosphonic-type inhibitors of human neutrophil elastase

Karolina Torzyk-Jurowska[1], Jarosław Ciekot[2], Łukasz Winiarski[1,*]

[1] Division of Organic and Medicinal Chemistry, Faculty of Chemistry, Wroclaw University of Science and Technology, Wybrzeze Wyspianskiego 27, 50-370 Wroclaw, Poland.;
[2] Department of Experimental Oncology, Hirszfeld Institute of Immunology and Experimental Therapy, Polish Academy of Sciences, 53-114 Wroclaw, Poland;
***** Correspondence: lukasz.winiarski@pwr.edu.pl;

Amino Acid Structures 5
(1) Boc-L-Val-Gly-Val^P(OPh-4-SCH$_3$)$_2$ 9
(2) Boc-L-Val-L-Ala-Val^P(OPh-4-SCH$_3$)$_2$ 11
(3) Boc-L-Val-D-Ala-Val^P(OPh-4-SCH$_3$)$_2$ 13
(4) Boc-L-Val-Aib-Val^P(OPh-4-SCH$_3$)$_2$ 15
(5) Boc-L-Val-beta-Ala-Val^P(OPh-4-SCH$_3$)$_2$ 17
(6) Boc-L-Val-L-Abu-Val^P(OPh-4-SCH$_3$)$_2$ 19
(7) Boc-L-Val-D-Abu- Val^P(OPh-4-SCH$_3$)$_2$ 21
(8) Boc-L-Val-L-Nva- Val^P(OPh-4-SCH$_3$)$_2$ 23
(9) Boc-L-Val-D-Nva- Val^P(OPh-4-SCH$_3$)$_2$ 25
(10) Boc-L-Val-L-Val-Val^P(OPh-4-SCH$_3$)$_2$ 27
(11) Boc-L-Val-D-Val- Val^P(OPh-4-SCH$_3$)$_2$ 29
(12) Boc-L-Val-L-Tle- Val^P(OPh-4-SCH$_3$)$_2$ 31
(13) Boc-L-Val-D-Tle- Val^P(OPh-4-SCH$_3$)$_2$ 33
(14) Boc-L-Val-L-Nle- Val^P(OPh-4-SCH$_3$)$_2$ 35
(15) Boc-L-Val-D-Nle- Val^P(OPh-4-SCH$_3$)$_2$ 37
(16) Boc-L-Val-L-Leu-Val^P(OPh-4-SCH$_3$)$_2$ 39
(17) Boc-L-Val-D-Leu-Val^P(OPh-4-SCH$_3$)$_2$ 41
(18) Boc-L-Val-L-NptGly-ValP(OPh-4-SCH3)2 43
(19) Boc-L-Val-L-Met-Val^P(OPh-4-SCH$_3$)$_2$ 45
(20) Boc-L-Val-D-Met-Val^P(OPh-4-SCH$_3$)$_2$ 47
(21) Boc-L-Val-L-Met(O)- Val^P(OPh-4-SCH$_3$)$_2$ 49
(22) Boc-L-Val-L-Met(O2)- Val^P(OPh-4-SCH$_3$)$_2$ 51
(23) Boc-L-Val-L-Ser(Bzl)-Val^P(OPh-4-SCH$_3$)$_2$ 53
(24) Boc-L-Val-D-Ser(Bzl)-Val^P(OPh-4-SCH$_3$)$_2$ 55
(25) Boc-L-Val-L-Thr(Bzl)-Val^P(OPh-4-SCH$_3$)$_2$ 57
(26) Boc-L-Val-D-Thr(Bzl)- Val^P(OPh-4-SCH$_3$)$_2$ 59
(27) Boc-L-Val-L-Pro-Val^P(OPh-4-SCH$_3$)$_2$ 61
(28) Boc-L-Val-D-Pro-Val^P(OPh-4-SCH$_3$)$_2$ 62
(29) Boc-L-Val-L-Oic-Val^P(OPh-4-SCH$_3$)$_2$ 64
(30) Boc-L-Val-L-Pip-Val^P(OPh-4-SCH$_3$)$_2$ 66
(31) Boc-L-Val-D-Pip-Val^P(OPh-4-SCH$_3$)$_2$ 68
(32) Boc-L-Val-L-Tic-Val^P(OPh-4-SCH$_3$)$_2$ 70
(33) Boc-L-Val-D-Tic- Val^P(OPh-4-SCH$_3$)$_2$ 72
(34) Boc-L-Val-L-Nip-Val^P(OPh-4-SCH$_3$)$_2$ 74
(35) Boc-L-Val-L-Cpg-Val^P(OPh-4-SCH$_3$)$_2$ 76
(36) 344.2 Boc-L-Val-L-Chg-Val^P(OPh-4-SCH$_3$)$_2$ 78
(37) Boc-L-Val-D-Chg-Val^P(OPh-4-SCH$_3$)$_2$ 80

(38) Boc-L-Val-L-Phe- $Val^P(OPh\text{-}4\text{-}SCH_3)_2$ 82
(39) Boc-L-Val-D-Phe-$Val^P(OPh\text{-}4\text{-}SCH_3)_2$ 84
(40) Boc-L-Val-L-Cha- $Val^P(OPh\text{-}4\text{-}SCH_3)_2$ 86
(41) Boc-L-Val-D-Igl-$Val^P(OPh\text{-}4\text{-}SCH_3)_2$....88
(42) Boc-L-Val-D-Igl-$Val^P(OPh\text{-}4\text{-}SCH_3)_2$....90
(43) Boc-L-Val-L-Trp- $Val^P(OPh\text{-}4\text{-}SCH_3)_2$ 92
(44) Boc-L-Val-D-Trp-$Val^P(OPh\text{-}4\text{-}SCH_3)_2$ 94
(45) Boc-L-Val-L-Asp(Bzl)-$Val^P(OPh\text{-}4\text{-}SCH_3)_2$ 96
(46) Boc-L-Val-D-Asp(Bzl)-$Val^P(OPh\text{-}4\text{-}SCH_3)_2$ 98
(47) Boc-L-Val-L-Cys(Bzl)-$Val^P(OPh\text{-}4\text{-}SCH_3)_2$....100
(48) Boc-L-Val-L-Glu(Bzl)- $Val^P(OPh\text{-}4\text{-}SCH_3)_2$....102
(49) Boc-L-Val-D-Orn(Z)-$Val^P(OPh\text{-}4\text{-}SCH_3)_2$ 104
(50) Boc-L-Val-D-Tyr(Bzl)- $Val^P(OPh\text{-}4\text{-}SCH_3)_2$ 106
(51) Boc-L-Val-L-Tyr(Bzl)-$Val^P(OPh\text{-}4\text{-}SCH_3)_2$ 108
(52) Boc-L-Val-L-Lys(Z)-$Val^P(OPh\text{-}4\text{-}SCH_3)_2$ 110
(53) Boc-L-Val-D-Lys(Z)-$Val^P(OPh\text{-}4\text{-}SCH_3)_2$ 112
(54) 353.2 Boc-L-Val-L-Hyp(Bzl)-$Val^P(OPh\text{-}4\text{-}SCH_3)_2$....114
(55) Boc-Gly-L-Pro-$Val^P(OPh\text{-}4\text{-}SCH_3)_2$ 116
(56) Boc-L-Ala-L-Pro-$Val^P(OPh\text{-}4\text{-}SCH_3)_2$....118
(57) Boc-D-Ala-L-Pro-$Val^P(OPh\text{-}4\text{-}SCH_3)_2$....120
(58) Boc-Aib-L-Pro- $Val^P(OPh\text{-}4\text{-}SCH_3)_2$ 122
(59) Boc-AC3C-L-Pro- $Val^P(OPh\text{-}4\text{-}SCH_3)_2$ 124
(60) Boc-beta-Ala-L-Pro- $Val^P(OPh\text{-}4\text{-}SCH_3)_2$ 126
(61) Boc-GABA-L-Pro-$Val^P(OPh\text{-}4\text{-}SCH_3)_2$ 128
(62) Boc-L-Abu-L-Pro-$Val^P(OPh\text{-}4\text{-}SCH_3)_2$ 130
(63) Boc-D-Abu-L-Pro-$Val^P(OPh\text{-}4\text{-}SCH_3)_2$ 132
(64) Boc-AC4C-L-Pro-$Val^P(OPh\text{-}4\text{-}SCH_3)_2$ 134
(65) Boc-L-Nva-L-Pro- $Val^P(OPh\text{-}4\text{-}SCH_3)_2$....136
(66) Boc-D-Nva-L-Pro-$Val^P(OPh\text{-}4\text{-}SCH_3)_2$....138
(67) Boc-D-Val-L-Pro-$Val^P(OPh\text{-}4\text{-}SCH_3)_2$....140
(68) Boc-L-Tle-L-Pro-$Val^P(OPh\text{-}4\text{-}SCH_3)_2$ 142
(69) Boc-D-Tle-L-Pro- $Val^P(OPh\text{-}4\text{-}SCH_3)_2$ 144
(70) Boc-L-Nle-L-Pro-$Val^P(OPh\text{-}4\text{-}SCH_3)_2$ 146
(71) Boc-D-Nle-L-Pro-$Val^P(OPh\text{-}4\text{-}SCH_3)_2$ 148
(72) Boc-L-Leu-L-Pro- $Val^P(OPh\text{-}4\text{-}SCH_3)_2$ 150
(73) Boc-D-Leu-L-Pro-$Val^P(OPh\text{-}4\text{-}SCH_3)_2$ 152
(74) Boc-L-NptGly-L-Pro-$Val^P(OPh\text{-}4\text{-}SCH_3)_2$ 154
(75) Boc-L-Met-L-Pro- $Val^P(OPh\text{-}4\text{-}SCH_3)_2$ 156
(76) Boc-D-Met-L-Pro-$Val^P(OPh\text{-}4\text{-}SCH_3)_2$ 158

(77) Boc-L-Met(O)-L-Pro-Val^P(OPh-4-SCH_3)$_2$ 160
(78) Boc-L-Met(O)$_2$-L-Pro- Val^P(OPh-4-SCH_3)$_2$ 162
(79) Boc-L-Gln-L-Pro-Val^P(OPh-4-SCH_3)$_2$ 164
(80) Boc-D-Gln-L-Pro- Val^P(OPh-4-SCH_3)$_2$ 166
(81) Boc-L-Pro-L-Pro-Val^P(OPh-4-SCH_3)$_2$ 168
(82) Boc-L-Pip-L-Pro-Val^P(OPh-4-SCH_3)$_2$ 170
(83) Boc-D-Pip-L-Pro- Val^P(OPh-4-SCH_3)$_2$ 172
(84) Boc-L-Nip-L-Pro- Val^P(OPh-4-SCH_3)$_2$ 174
(85) Boc-D-Nip-L-Pro- Val^P(OPh-4-SCH_3)$_2$ 176
(86) Boc-1,4-cis-ACHC-L-Pro-Val^P(OPh-4-SCH_3)$_2$ 178
(87) Boc-1,4-trans-ACHC-L-Pro-Val^P(OPh-4-SCH_3)$_2$ 180
(88) Boc-L-Cpg-L-Pro-Val^P(OPh-4-SCH_3)$_2$ 182
(89) Boc-L-Phg-L-Pro-Val^P(OPh-4-SCH_3)$_2$ 184
(90) Boc-D-Phg-L-Pro- Val^P(OPh-4-SCH_3)$_2$ 186
(91) Boc-L-Chg-L-Pro-Val^P(OPh-4-SCH_3)$_2$ 188
(92) Boc-D-Chg-L-Pro-Val^P(OPh-4-SCH_3)$_2$ 190
(93) Boc-L-Phe-L-Pro-Val^P(OPh-4-SCH_3)$_2$ 192
(94) Boc-D-Phe-L-Pro- Val^P(OPh-4-SCH_3)$_2$ 194
(95) Boc-L-Cha-L-Pro-Val^P(OPh-4-SCH_3)$_2$ 196
(96) Boc-L-HPhe-L-Pro-Val^P(OPh-4-SCH_3)$_2$ 198
(97) Boc-D-HPhe-L-Pro-Val^P(OPh-4-SCH_3)$_2$ 200
(98) Boc-L-Igl-L-Pro-Val^P(OPh-4-SCH_3)$_2$ 202
(99) Boc-D-Igl-L-Pro-Val^P(OPh-4-SCH_3)$_2$ 204
(100) Boc-L-Thr(Bzl)-L-Pro- Val^P(OPh-4-SCH_3)$_2$ 206
(101) Boc-L-Cys(Bzl)-L-Pro- Val^P(OPh-4-SCH_3)$_2$ 208
(102) Boc-D-Cys(Bzl)-L-Pro-Val^P(OPh-4-SCH_3)$_2$ 210
(103) Boc-L-Asp(Bzl)-L-Pro- Val^P(OPh-4-SCH_3)$_2$ 212
(104) Boc-D-Asp(Bzl)-L-Pro- Val^P(OPh-4-SCH_3)$_2$ 214
(105) Boc-L-Glu(Bzl)-L-Pro- Val^P(OPh-4-SCH_3)$_2$ 216
(106) Boc-L-His(Bzl)-L-Pro-Val^P(OPh-4-SCH_3)$_2$ 218
(107) Boc-L-Orn(Z)-L-Pro-Val^P(OPh-4-SCH_3)$_2$ 220
(108) Boc-D-Orn(Z)-L-Pro-Val^P(OPh-4-SCH_3)$_2$ 222
(109) Boc-L-Lys(Z)-L-Pro-Val^P(OPh-4-SCH_3)$_2$ 224
(110) Boc-D-Lys(Z)-L-Pro-Val^P(OPh-4-SCH_3)$_2$ 226
(111) Hm-L-Met(O_2)-L-Pro- Val^P(OPh-4-SCH_3)$_2$ 228
(112) Hm-L-Met(O_2)-L-Pro-D-Val^P(OPh-4-SCH_3)$_2$ 230
(113) Hm-L-Met(O_2)-L-Pro-L-Val^P(OPh-4-SCH_3)$_2$ 232

Amino Acid Structures

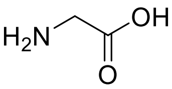

Gly
glycine

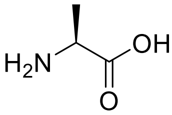

L-Ala
L-Alanine

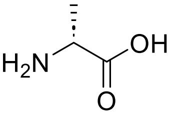

D-Ala
D-Alanine

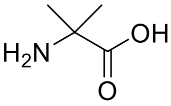

Aib
2-Aminoisobutyric acid

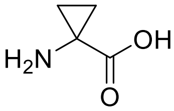

AC3C
1-Amino-1-cyclopropanecarboxylic acid

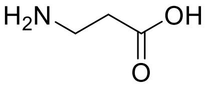

beta-Ala
beta Alanine

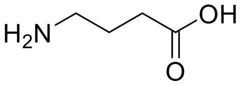

GABA
gamma-aminobutyric acid

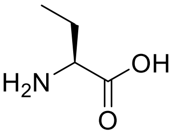

L-Abu
L-Aminobutyric acid

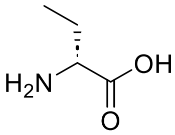

D-Abu
D-Aminobutyric acid

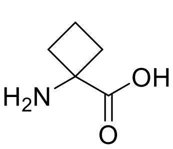

AC4C
1-cyclobutyl-1-carboxylic acid

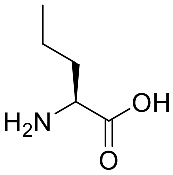

L-Nva
L-Norvaline

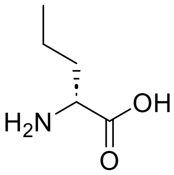

D-Nva
D-Norvaline

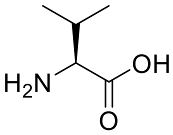

L-Val
L-Valine

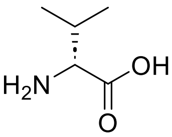

D-Val
D-Valine

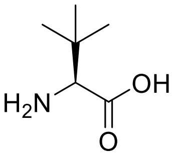

L-Tle
L-*tert*-Leucine

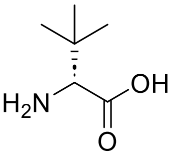

D-Tle
D-*tert*-Leucine

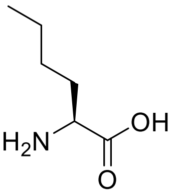

L-Nle
L-Norleucine

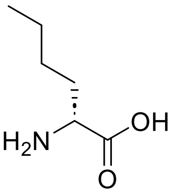

D-Nle
D-Norleucine

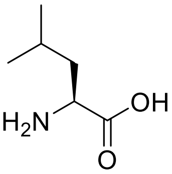

L-Leu
L-Leucine

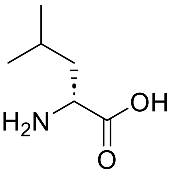

D-Leu
D-Leucine

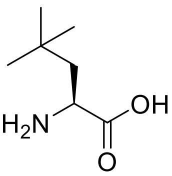

L-NptGly
L-Neopentylglycine

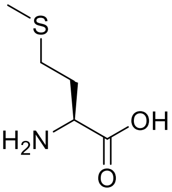

L-Met
L-Methionine

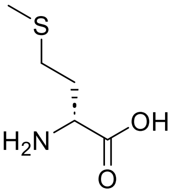

D-Met
D-Methionine

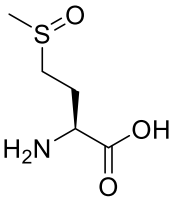

L-Met(O)
L-Methionine sulfoxide

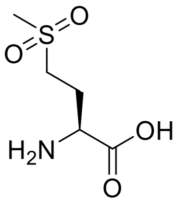

L-Met(O)$_2$
L-Methionine sulfone

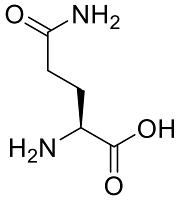

L-Gln
L-Glutamine

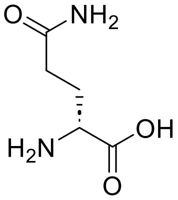

D-Gln
D-Glutamine

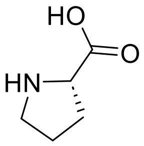

L-Pro
L-Proline

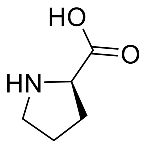

D-Pro
D-Proline

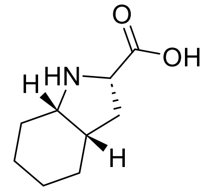

L-Oic
L-Octahydroindole-2-carboxylic acid

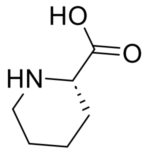

L-Pip
L-Pipecolic acid

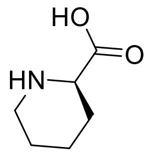

D-Pip
D-Pipecolic acid

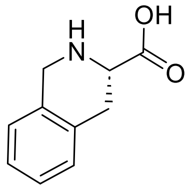

L-Tic
L-Tetrahydroisoquinoline-3-carboxylic acid

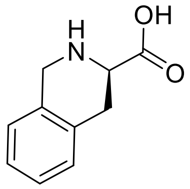

D-Tic
D-Tetrahydroisoquinoline-3-carboxylic acid

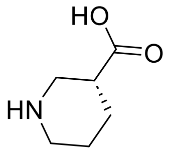

L-Nip
L-Nipecotic Acid

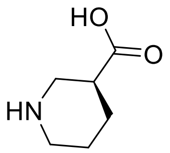

D-Nip
D-Nipecotic Acid

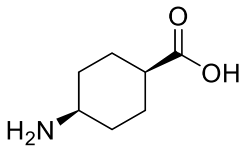

1,4-*cis*-ACHC
***cis*-4-Amino-cyclohexanecarboxylic acid**

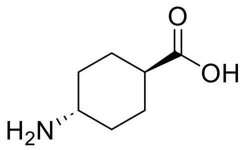

1,4-*trans*-ACHC
***trans*-4-Amino-cyclohexanecarboxylic acid**

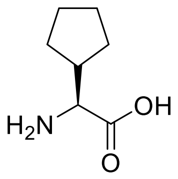

L-Cpg
L-Cyclopentylglycine

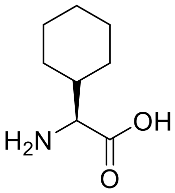

L-Chg
L-Cyclohexylglycine

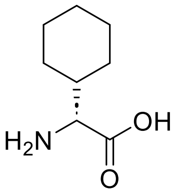

D-Chg
D-Cyclohexylglycine

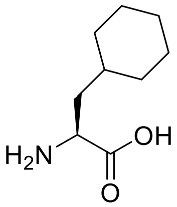

L-Cha
L-Cyclohexylalanine

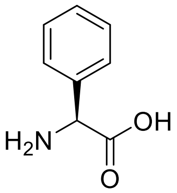

L-Phg
L-Phenylglycine

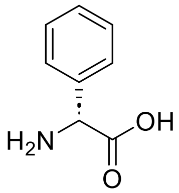

D-Phg
D-Phenylglycine

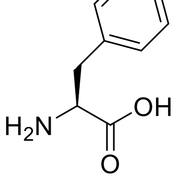

L-Phe
L-Phenylalanine

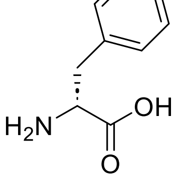

D-Phe
D-Phenylalanine

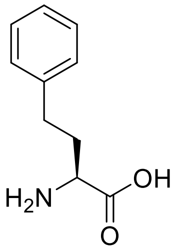

L-HPhe
L-Homophenylalanine

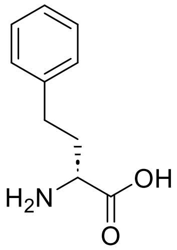

D-HPhe
D-Homophenylalanine

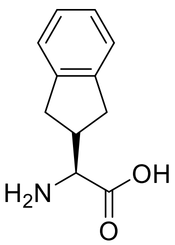

L-Igl
L-Indanylglycine

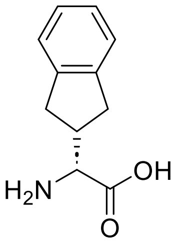

D-Igl
D-Indanylglycine

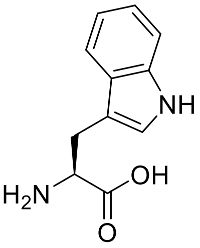

L-Trp
L-Tryptophan

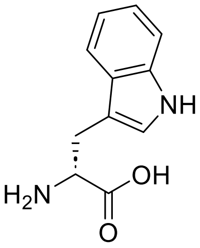

D-Trp
D-Tryptophan

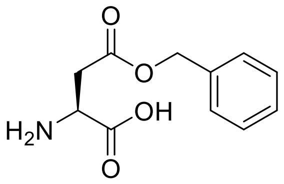

L-Asp(Bzl)
L-Aspartic acid beta-benzyl ester

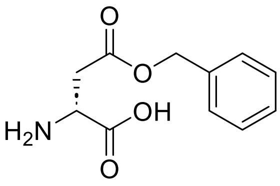

D-Asp(Bzl)
D-Aspartic acid beta-benzyl ester

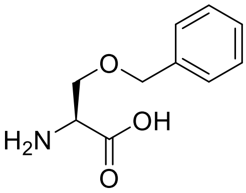

L-Ser(Bzl)
O-benzyl-L-serine

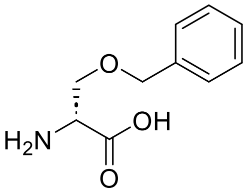

D-Ser(Bzl)
O-benzyl-D-serine

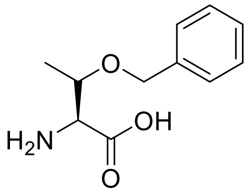

L-Thr(Bzl)
O-benzyl-L-threonine

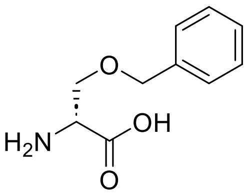

D-Thr(Bzl)
O-benzyl-D-threonine

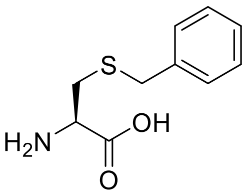

L-Cys(Bzl)
S-benzyl-L-cysteine

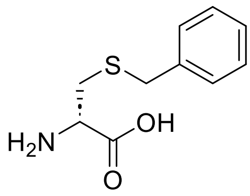

D-Cys(Bzl)
O-benzyl-D-cysteine

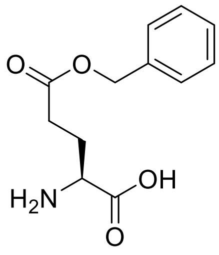

L-Glu(Bzl)
L-Glutamic acid gamma benzyl ester

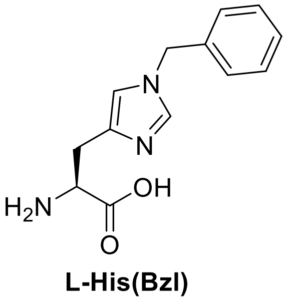

L-His(Bzl)
N(im)-benzyl-L-histidine

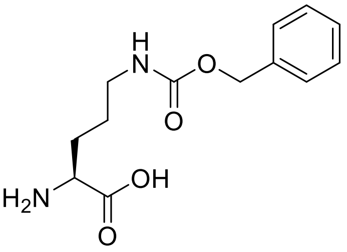

L-Orn(Z)
N-delta-benzyloxycarbonyl-L-ornithin

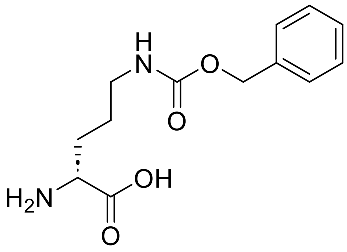

D-Orn(Z)
N-delta-benzyloxycarbonyl-D-ornithin

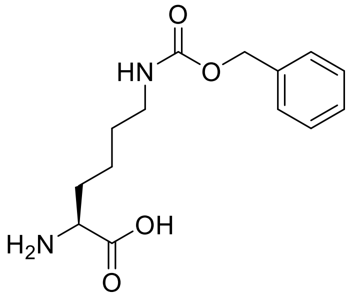

L-Lys(Z)
N-epsilon-benzyloxycarbonyl-L-lysine

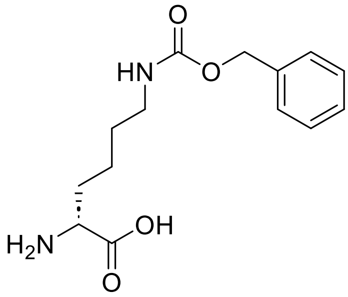

D-Lys(Z)
N-epsilon-benzyloxycarbonyl-D-lysine

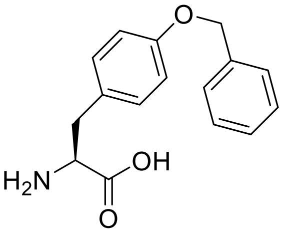

L-Tyr(Bzl)
O-benzyl-L-tyrosine

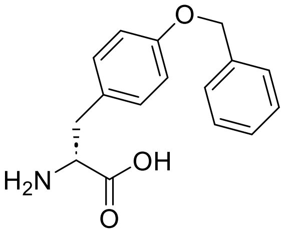

D-Tyr(Bzl)
O-benzyl-D-tyrosine

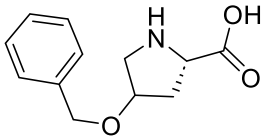

L-Hyp(Bzl)
O-benzyl-L-hydroxyproline

(1) Boc-L-Val-Gly-Val^{P}(OPh-4-SCH$_3$)$_2$

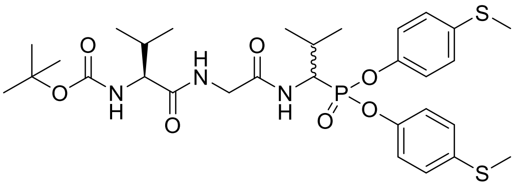

Name: *tert*-butyl ((2S)-1-((2-((1-(bis(4-(methylthio)phenoxy)phosphoryl)-2-methylpropyl)amino)-2-oxoethyl)amino)-3-methyl-1-oxobutan-2-yl)carbamate
^{31}P NMR (162 MHz, Chloroform-d) δ 17.70 (s, 49%), 17.68 (s, 51%).
^{1}H NMR (400 MHz, Chloroform-d) δ 7.43 – 7.30 (m, 1H, NH), 7.21 – 6.95 (m, 9H, 8xArH, NH), 5.24 (d, *J* = 8.6 Hz, 1H, NH), 4.79 – 4.66 (m, 1H, CHP), 4.07 – 3.80 (m, 3H, CH, CH$_2$), 2.43 – 2.40 (m, 6H, 2xSCH$_3$), 2.38 – 2.29 (m, 1H, CH), 2.16 – 2.05 (m, 1H, CH), 1.41 (s, 9H, Boc), 1.04 – 1.00 (m, 6H, 2xCH$_3$), 0.92 (d, *J* = 6.8 Hz, 3H, CH$_3$), 0.87 (d, *J* = 6.8 Hz, 3H, CH$_3$).
HRMS: $C_{30}H_{44}N_3O_7PS_2$ [M+Na]$^+$ calc. 676.2256 found 676.2239
HPLC: purity >99%; retention time 17.807 min

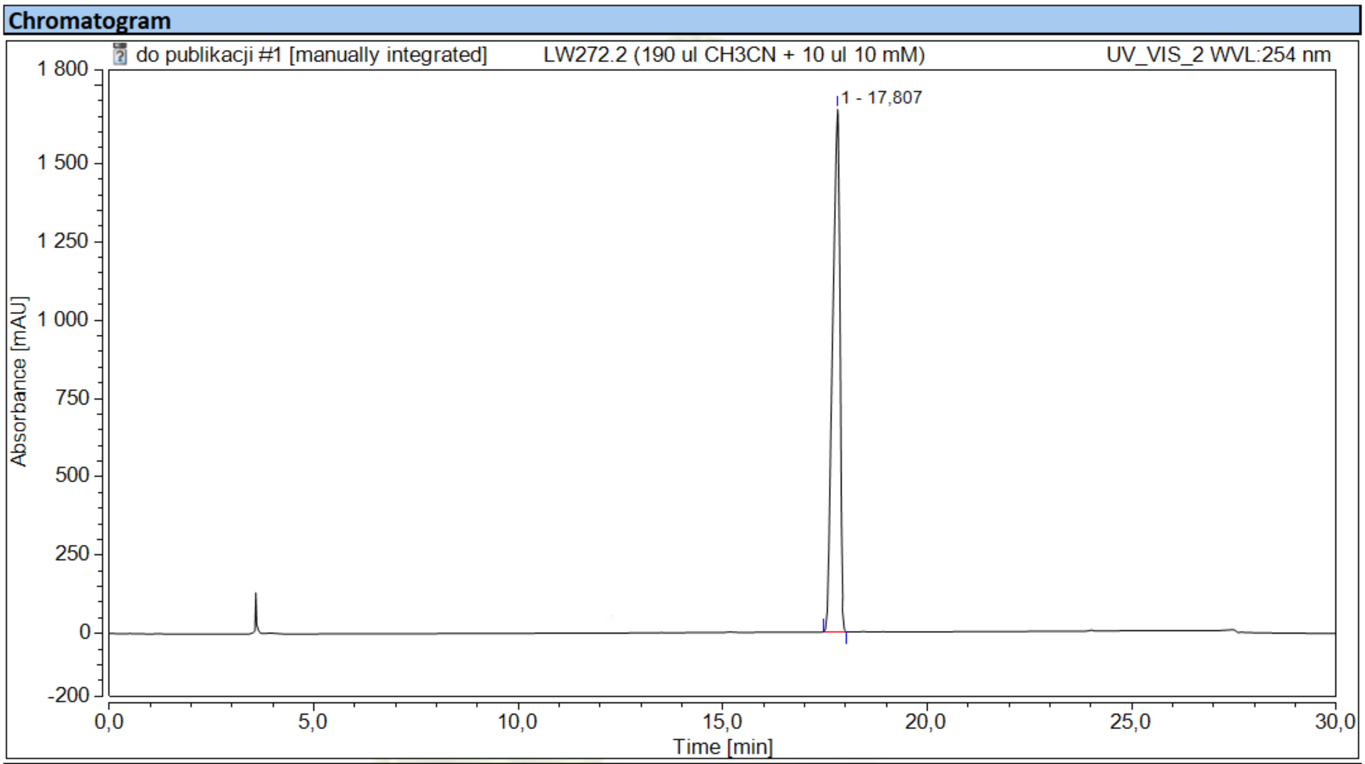

Integration Results

No.	Peak Name	Retention Time min	Area mAU*min	Height mAU	Relative Area %	Relative Height %	Amount n.a.
1		17,807	358,158	1666,615	100,00	100,00	n.a.
**Total:**			**358,158**	**1666,615**	**100,00**	**100,00**	

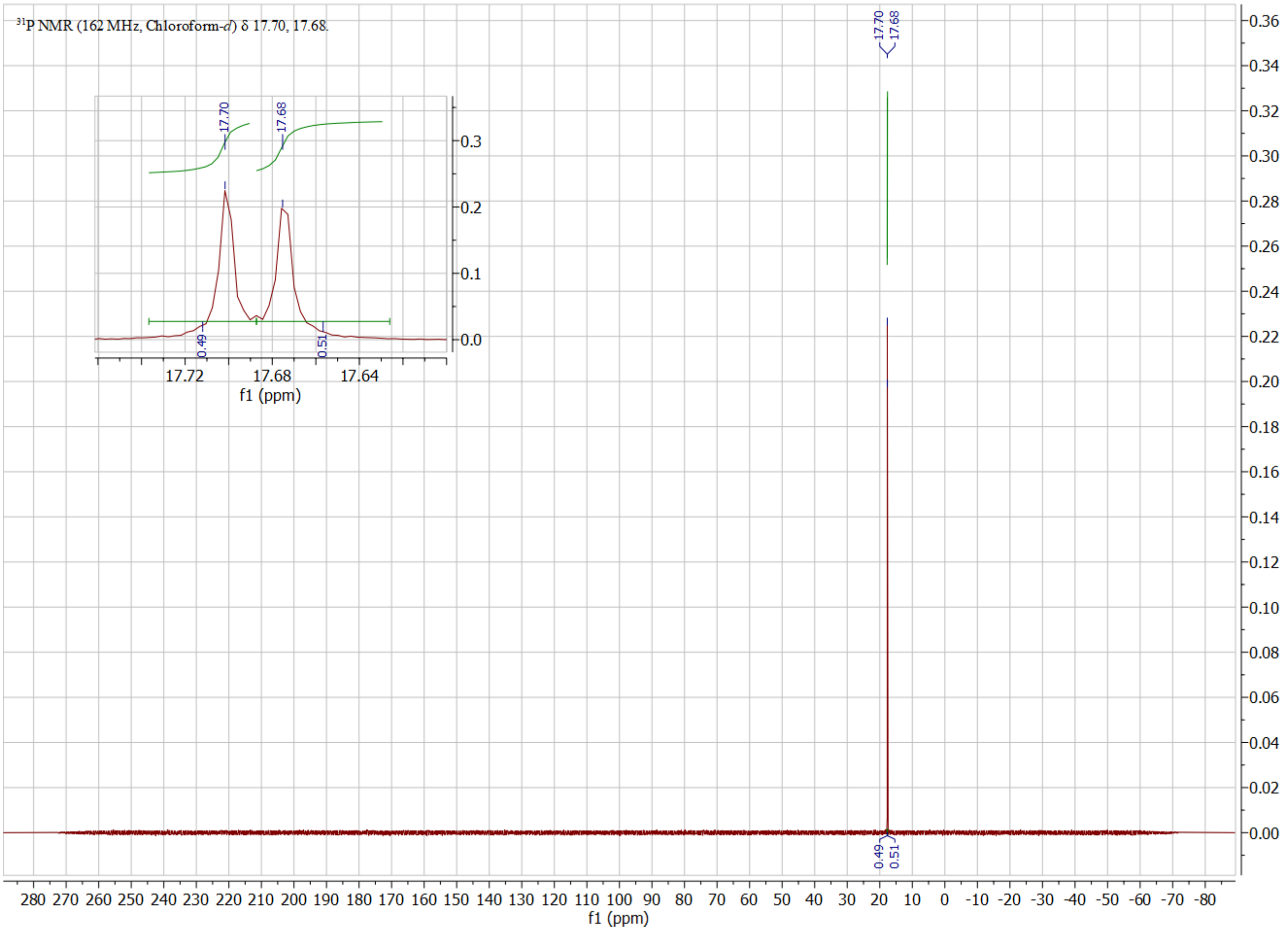
31P NMR (162 MHz, Chloroform-d) δ 17.70, 17.68.
f1 (ppm)

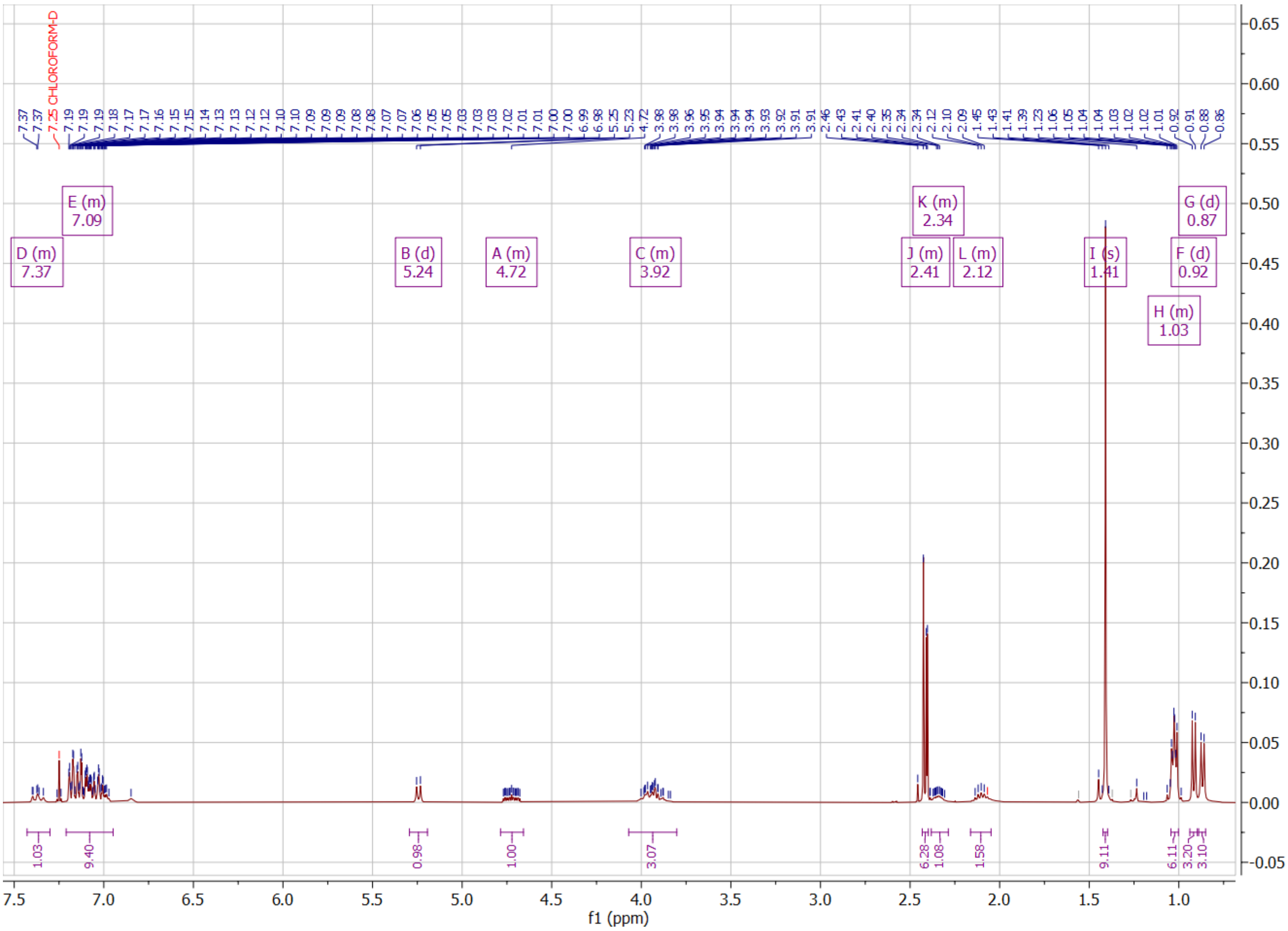
CHLOROFORM-D
E (m) 7.09
D (m) 7.37
B (d) 5.24
A (m) 4.72
C (m) 3.92
K (m) 2.34
J (m) 2.41
L (m) 2.12
I (s) 1.41
G (d) 0.87
F (d) 0.92
H (m) 1.03
f1 (ppm)

(2) Boc-L-Val-L-Ala-Val^P(OPh-4-SCH_3)$_2$

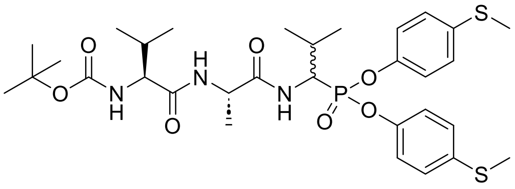

Name: *tert*-butyl ((2S)-1-(((2S)-1-((1-(bis(4-(methylthio)phenoxy)phosphoryl)-2-methylpropyl)amino)-1-oxopropan-2-yl)amino)-3-methyl-1-oxobutan-2-yl)carbamate
^{31}P NMR (162 MHz, Chloroform-d) δ 17.98 (s, 50%), 17.90 (s, 50%).
^{1}H NMR (400 MHz, Chloroform-d) δ 7.22 – 6.98 (m, 9H, 8xArH, NH), 6.64 (dd, J = 22.6, 7.2 Hz, 1H, NH), 5.12 (dd, J = 24.4, 8.4 Hz, 1H, NH), 4.78 – 4.63 (m, 1H, CHP), 4.61 – 4.50 (m, 1H, CH), 3.95 – 3.86 (m, 1H, CH), 2.43 – 2.41 (m, 6H, 2xSCH_3), 2.40 – 2.29 (m, 1H, CH), 2.16 – 2.04 (m, 1H, CH), 1.42 (d, J = 4.9 Hz, 9H, Boc), 1.32 (dd, J = 43.9, 7.0 Hz, 3H, CH_3), 1.10 – 0.98 (m, 6H, 2xCH_3), 0.95 – 0.81 (m, 6H, 2xCH_3).
HRMS: $C_{31}H_{46}N_3O_7PS_2$ $[M+Na]^+$ calc. 690.2412 found 690.2400
HPLC: purity >99%; retention time 18.087 min

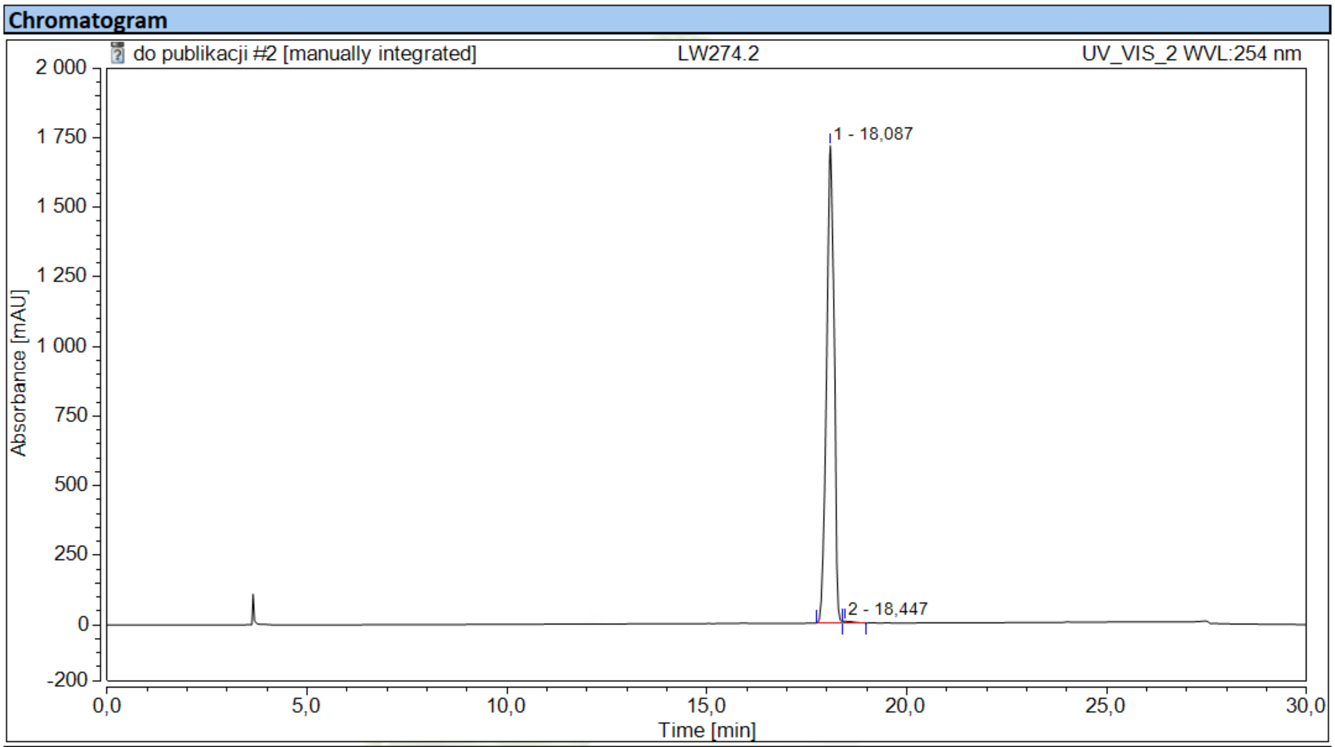

Integration Results

No.	Peak Name	Retention Time min	Area mAU*min	Height mAU	Relative Area %	Relative Height %	Amount n.a.
1		18,087	372,718	1714,232	99,51	99,62	n.a.
2		18,447	1,836	6,517	0,49	0,38	n.a.
**Total:**			**374,555**	**1720,750**	**100,00**	**100,00**	

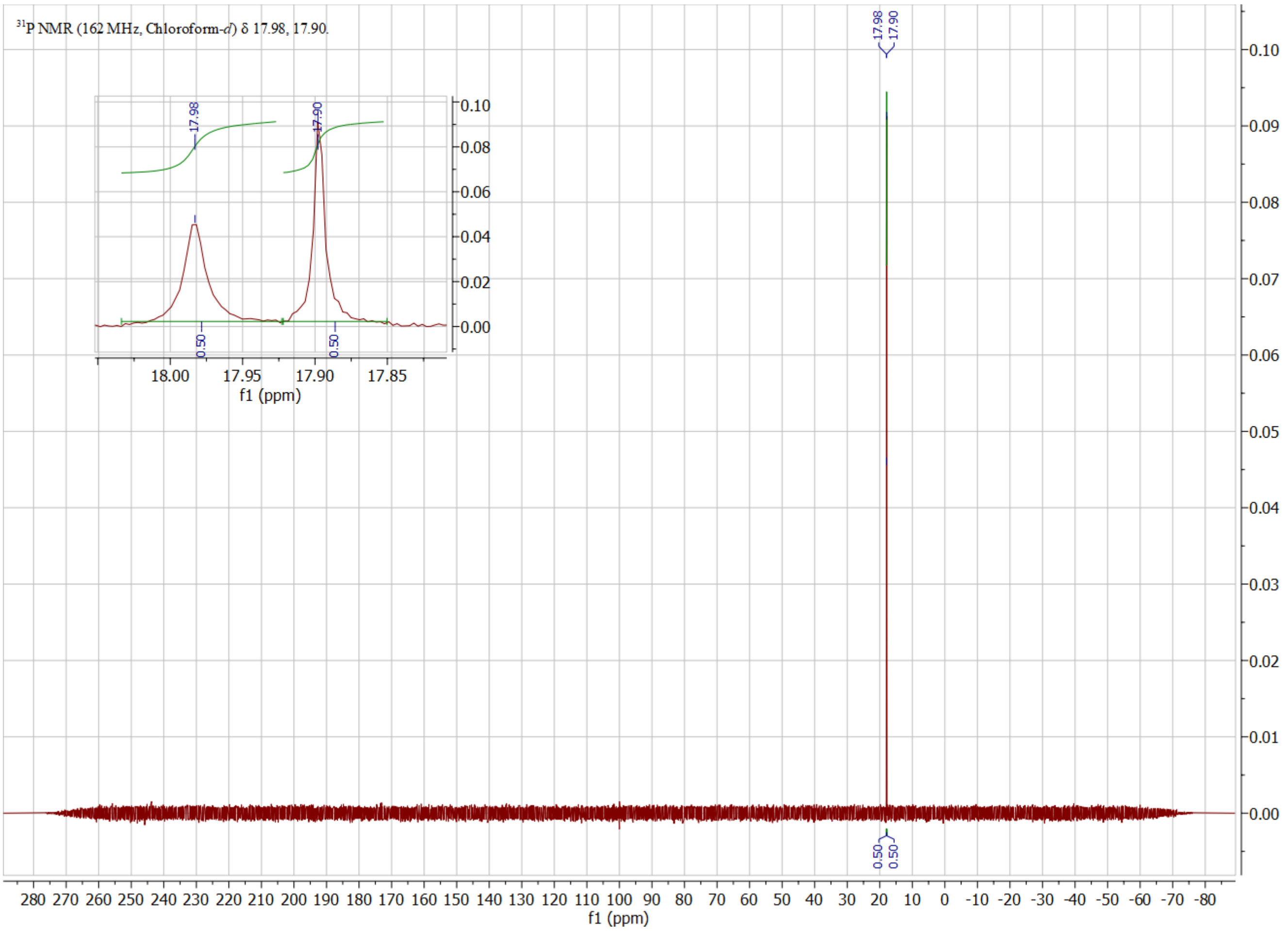

^{31}P NMR (162 MHz, Chloroform-*d*) δ 17.98, 17.90.
17.98
17.90
0.50
0.50
f1 (ppm)

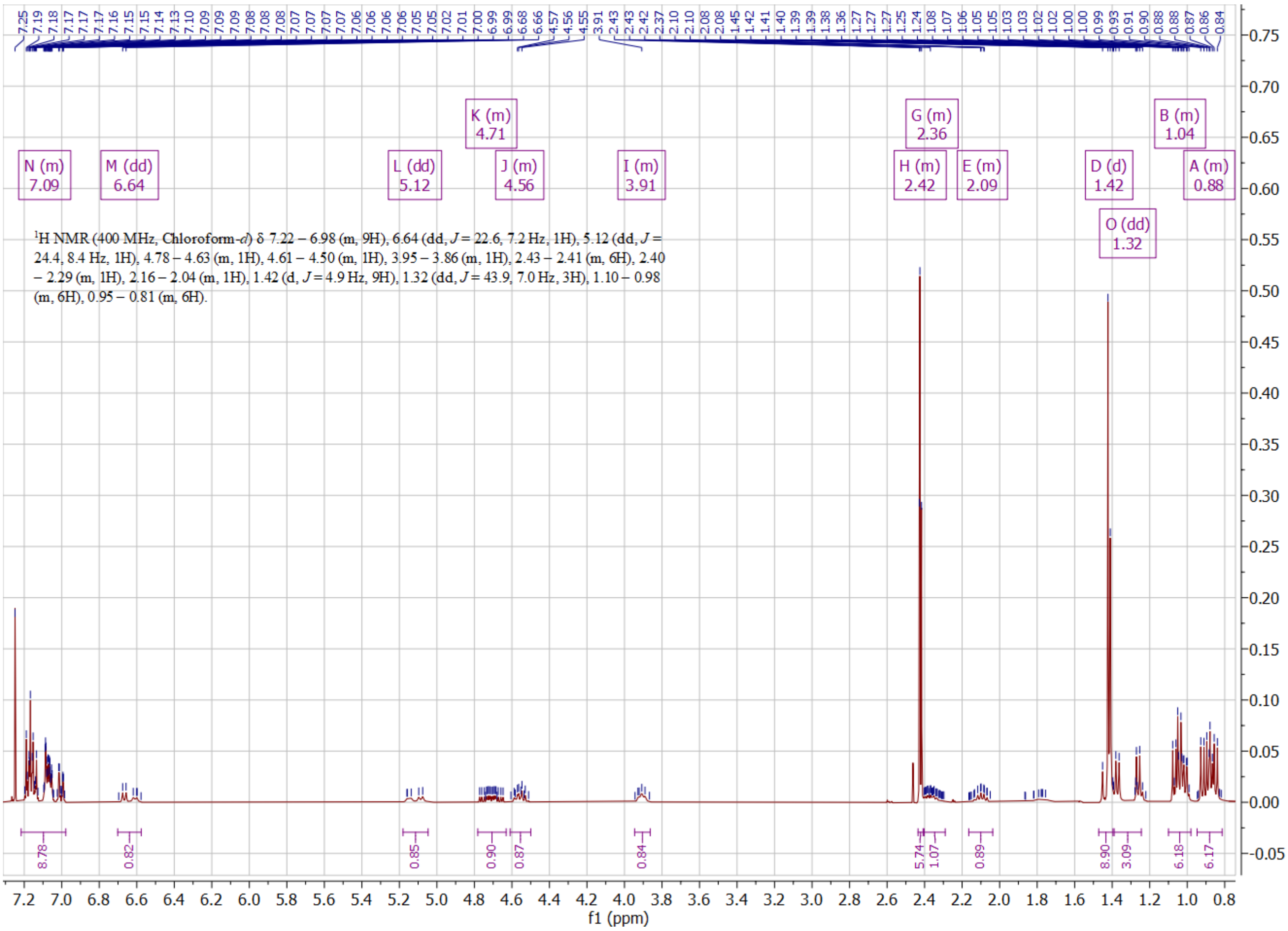

N (m) 7.09
M (dd) 6.64
L (dd) 5.12
K (m) 4.71
J (m) 4.56
I (m) 3.91
H (m) 2.42
G (m) 2.36
E (m) 2.09
D (d) 1.42
O (dd) 1.32
B (m) 1.04
A (m) 0.88
^{1}H NMR (400 MHz, Chloroform-*d*) δ 7.22 – 6.98 (m, 9H), 6.64 (dd, *J* = 22.6, 7.2 Hz, 1H), 5.12 (dd, *J* = 24.4, 8.4 Hz, 1H), 4.78 – 4.63 (m, 1H), 4.61 – 4.50 (m, 1H), 3.95 – 3.86 (m, 1H), 2.43 – 2.41 (m, 6H), 2.40 – 2.29 (m, 1H), 2.16 – 2.04 (m, 1H), 1.42 (d, *J* = 4.9 Hz, 9H), 1.32 (dd, *J* = 43.9, 7.0 Hz, 3H), 1.10 – 0.98 (m, 6H), 0.95 – 0.81 (m, 6H).
f1 (ppm)

(3) Boc-L-Val-D-Ala-Val^P(OPh-4-SCH$_3$)$_2$

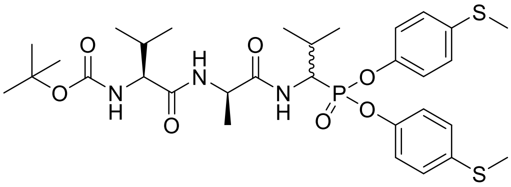

Name: *tert*-butyl ((2S)-1-(((2R)-1-((1-(bis(4-(methylthio)phenoxy)phosphoryl)-2-methylpropyl)amino)-1-oxopropan-2-yl)amino)-3-methyl-1-oxobutan-2-yl)carbamate

^{31}P NMR (162 MHz, Chloroform-d) δ 18.12 (s, 62%), 17.93 (s, 38%).

^{1}H NMR (400 MHz, Chloroform-d) δ 7.22 – 7.12 (m, 5H, 4xArH, NH), 7.11 – 6.99 (m, 4H, 4xArH), 6.82 – 6.55 (m, 1H, NH), 5.15 (dd, J = 28.0, 8.4 Hz, 1H, NH), 4.76 – 4.64 (m, 1H, CHP), 4.62 – 4.49 (m, 1H, CH), 3.98 – 3.85 (m, 1H, CH), 2.46 – 2.39 (m, 6H, 2xSCH$_3$), 2.40 – 2.29 (m, 1H, CH), 2.16 – 2.03 (m, 1H, CH), 1.46 – 1.38 (m, 9H, Boc), 1.31 (dd, J = 35.0, 7.0 Hz, 3H, CH$_3$), 1.06 – 0.97 (m, 6H, 2xCH$_3$), 0.92 (dd, J = 6.8, 4.7 Hz, 3H, CH$_3$), 0.86 (dd, J = 9.5, 6.7 Hz, 3H, CH$_3$).

HRMS: $C_{31}H_{46}N_3O_7PS_2$ [M+Na]$^+$ calc. 690.2412 found 690.2406

HPLC: purity >99%; retention time 18.173 min

Chromatogram

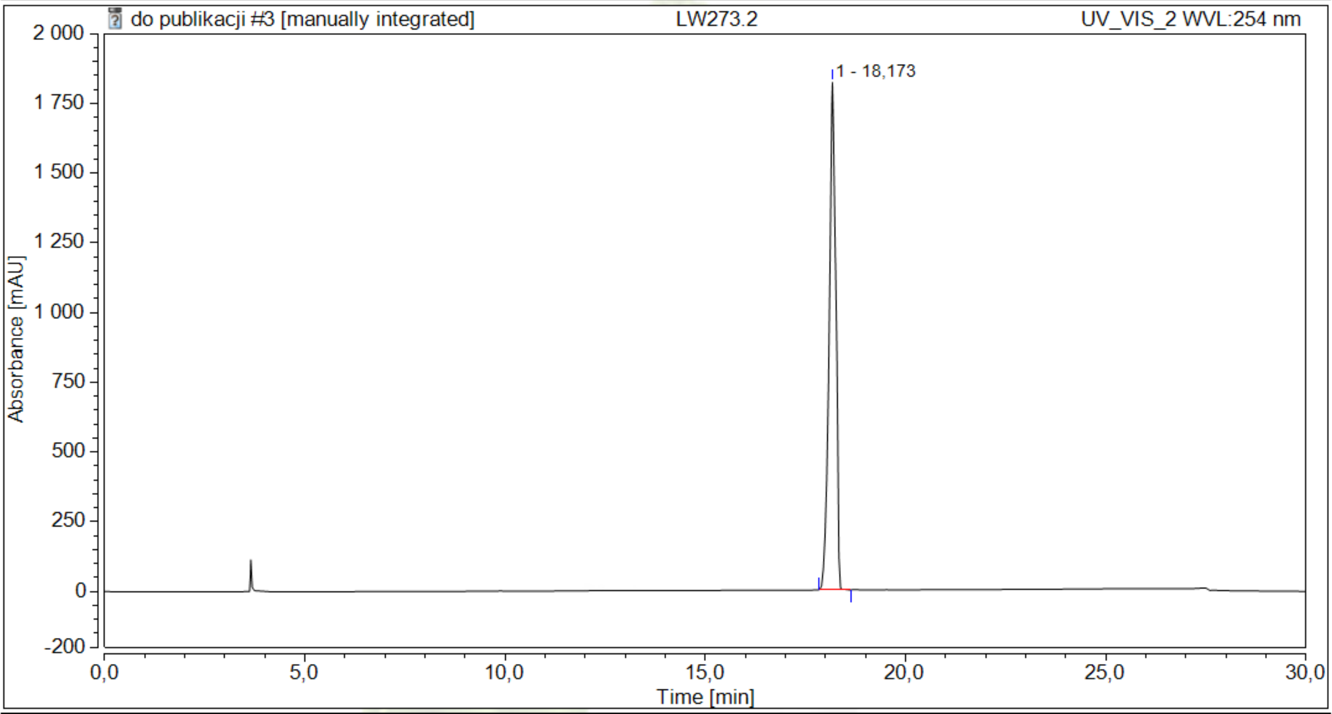

Integration Results

No.	Peak Name	Retention Time min	Area mAU*min	Height mAU	Relative Area %	Relative Height %	Amount n.a.
1		18,173	350,032	1819,067	100,00	100,00	n.a.
**Total:**			**350,032**	**1819,067**	**100,00**	**100,00**	

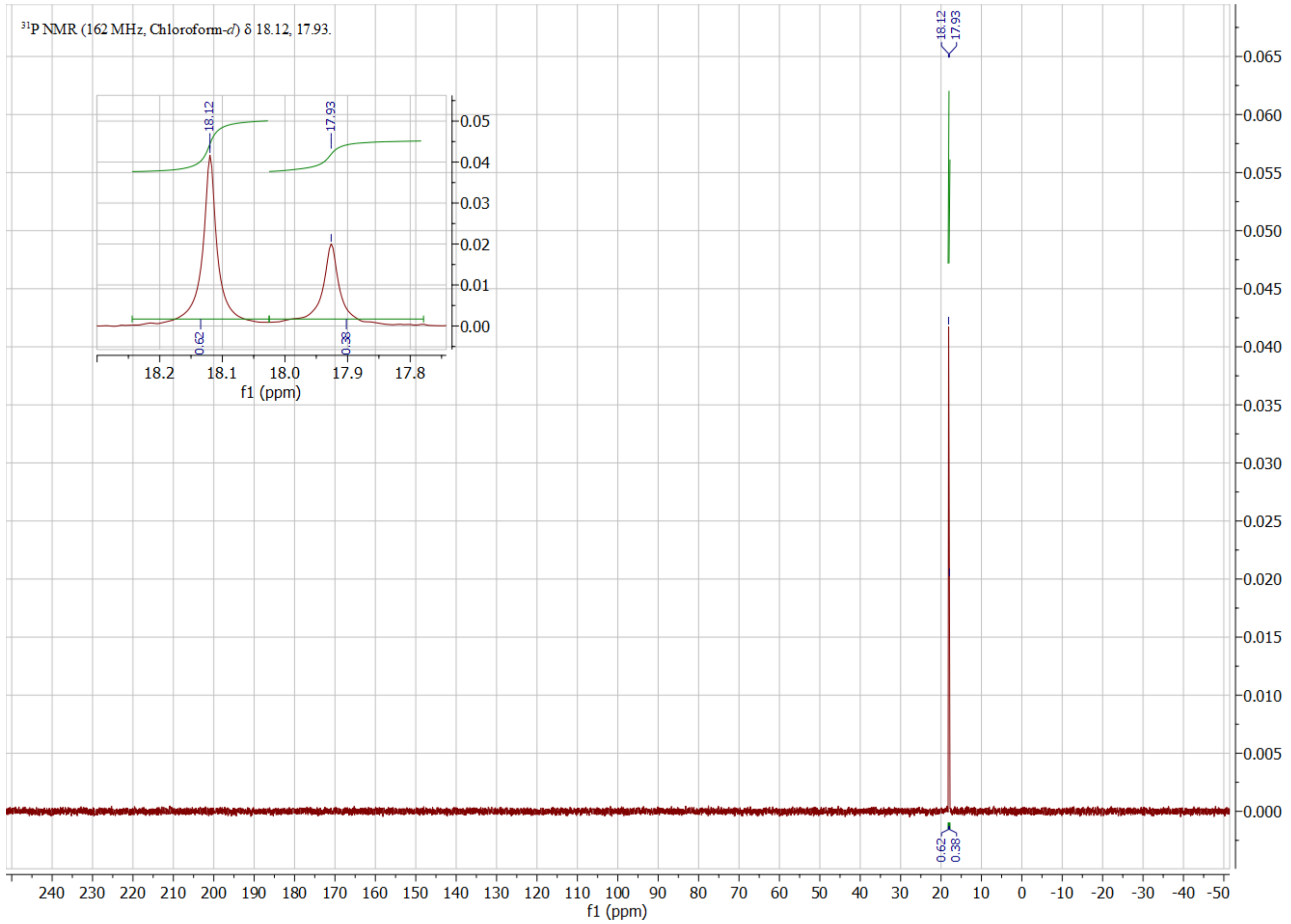

^{31}P NMR (162 MHz, Chloroform-d) δ 18.12, 17.93.
f1 (ppm)

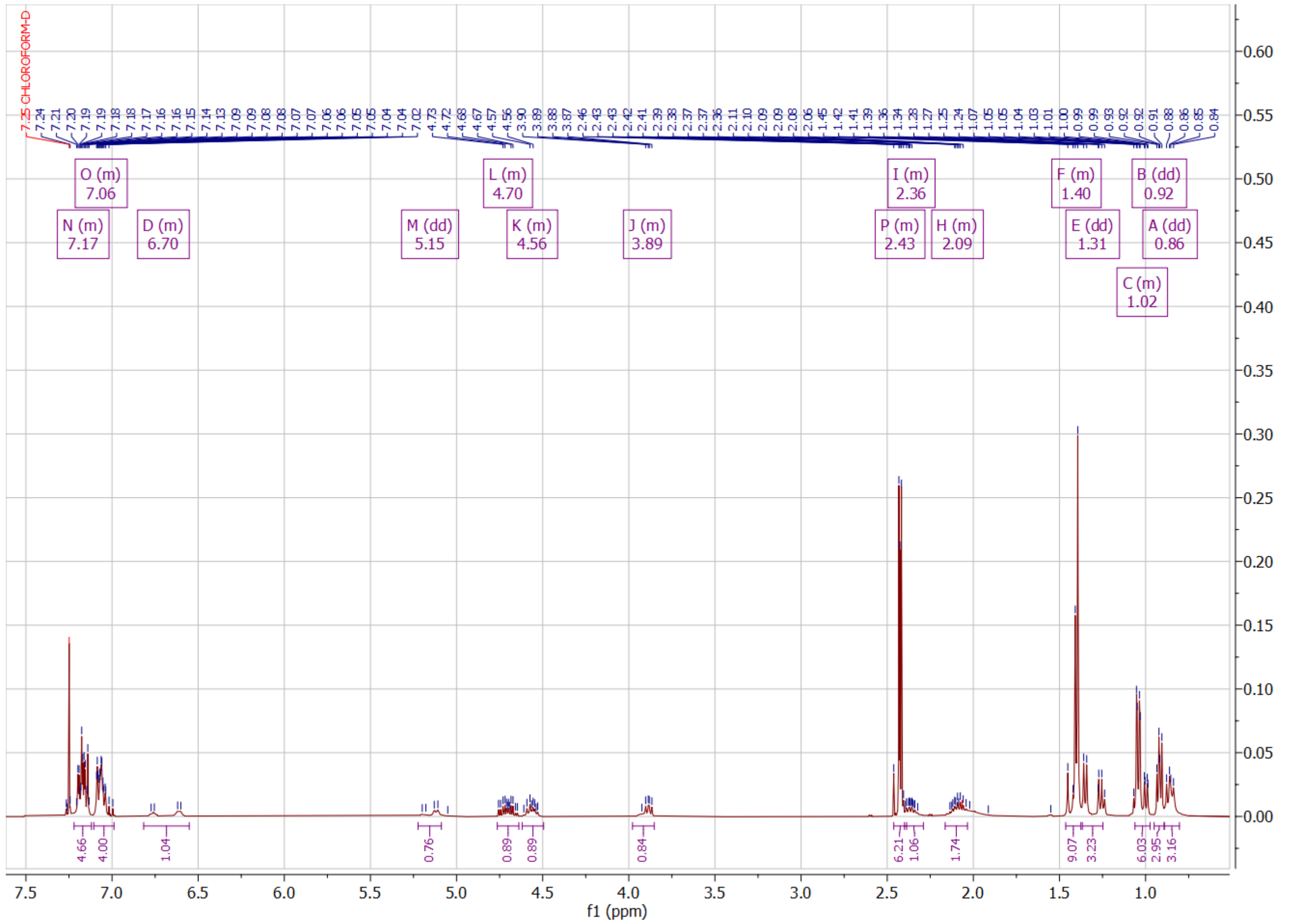

f1 (ppm)

(4) Boc-L-Val-Aib-Val^P(OPh-4-SCH$_3$)$_2$

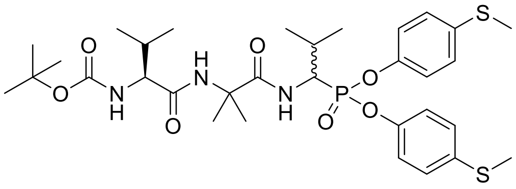

Name: *tert*-butyl ((2S)-1-((1-((1-(bis(4-(methylthio)phenoxy)phosphoryl)-2-methylpropyl)amino)-2-methyl-1-oxopropan-2-yl)amino)-3-methyl-1-oxobutan-2-yl)carbamate
^{31}P NMR (162 MHz, Chloroform-d) δ 18.81 (s, 36%), 18.71 (s, 64%).
^{1}H NMR (400 MHz, Chloroform-d) δ 7.32 (dd, J = 35.1, 10.0 Hz, 1H, NH), 7.22 – 7.15 (m, 4H, 4xArH), 7.14 – 7.03 (m, 4H, 4xArH), 6.46 (d, J = 26.2 Hz, 1H, NH), 4.99 (dd, J = 28.6, 7.7 Hz, 1H, NH), 4.78 – 4.69 (m, 1H, CHP), 3.84 – 3.77 (m, 1H, CH), 2.46 – 2.42 (m, 6H, 2xSCH$_3$), 2.42 – 2.32 (m, 1H, CH), 2.21 – 2.12 (m, 1H, CH), 1.58 – 1.44 (m, 6H, 2xCH$_3$), 1.41 (d, J = 2.7 Hz, 9H, Boc), 1.12 – 1.03 (m, 6H, 2xCH$_3$), 0.98 – 0.88 (m, 6H, 2xCH$_3$).
HRMS: $C_{32}H_{48}N_3O_7PS_2$ $[M+Na]^+$ calc. 704.2569 found 704.2555
HPLC: purity >99%; retention time 18.863 min

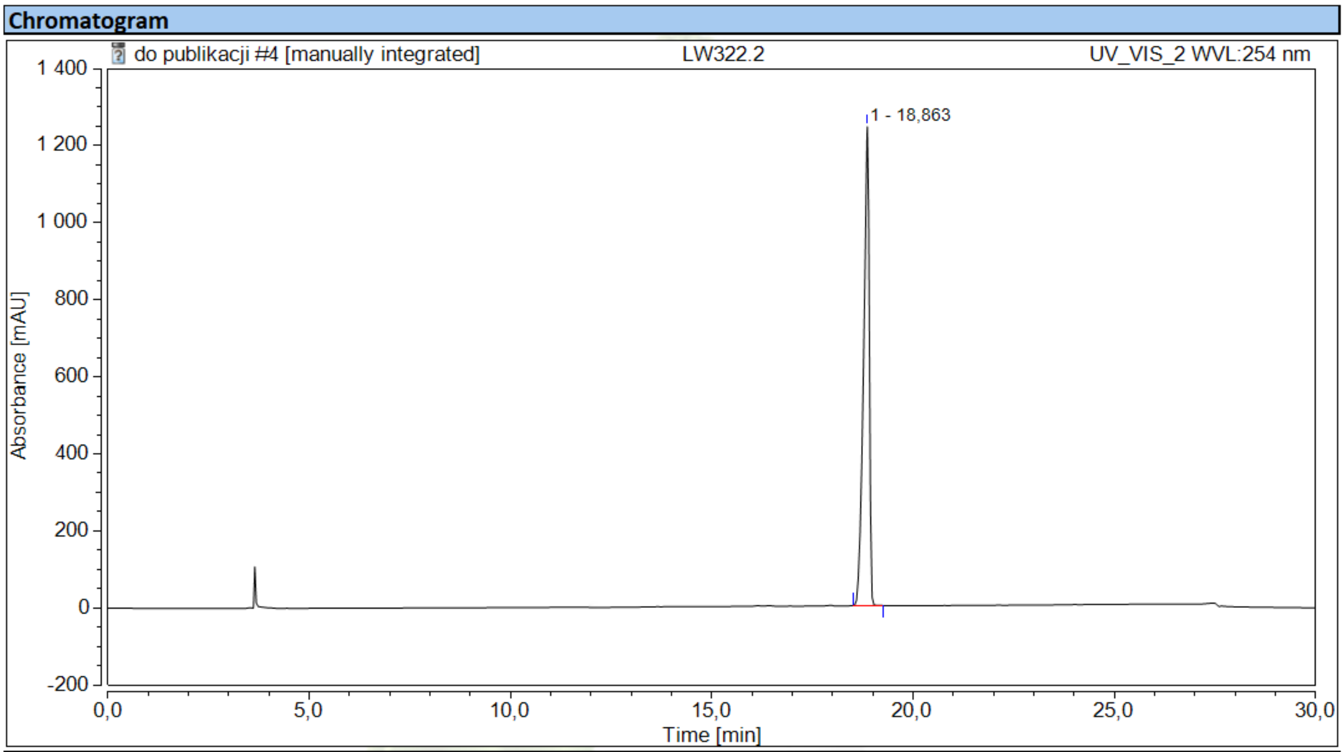

Integration Results

No.	Peak Name	Retention Time min	Area mAU*min	Height mAU	Relative Area %	Relative Height %	Amount n.a.
1		18,863	202,312	1242,815	100,00	100,00	n.a.
**Total:**			**202,312**	**1242,815**	**100,00**	**100,00**	

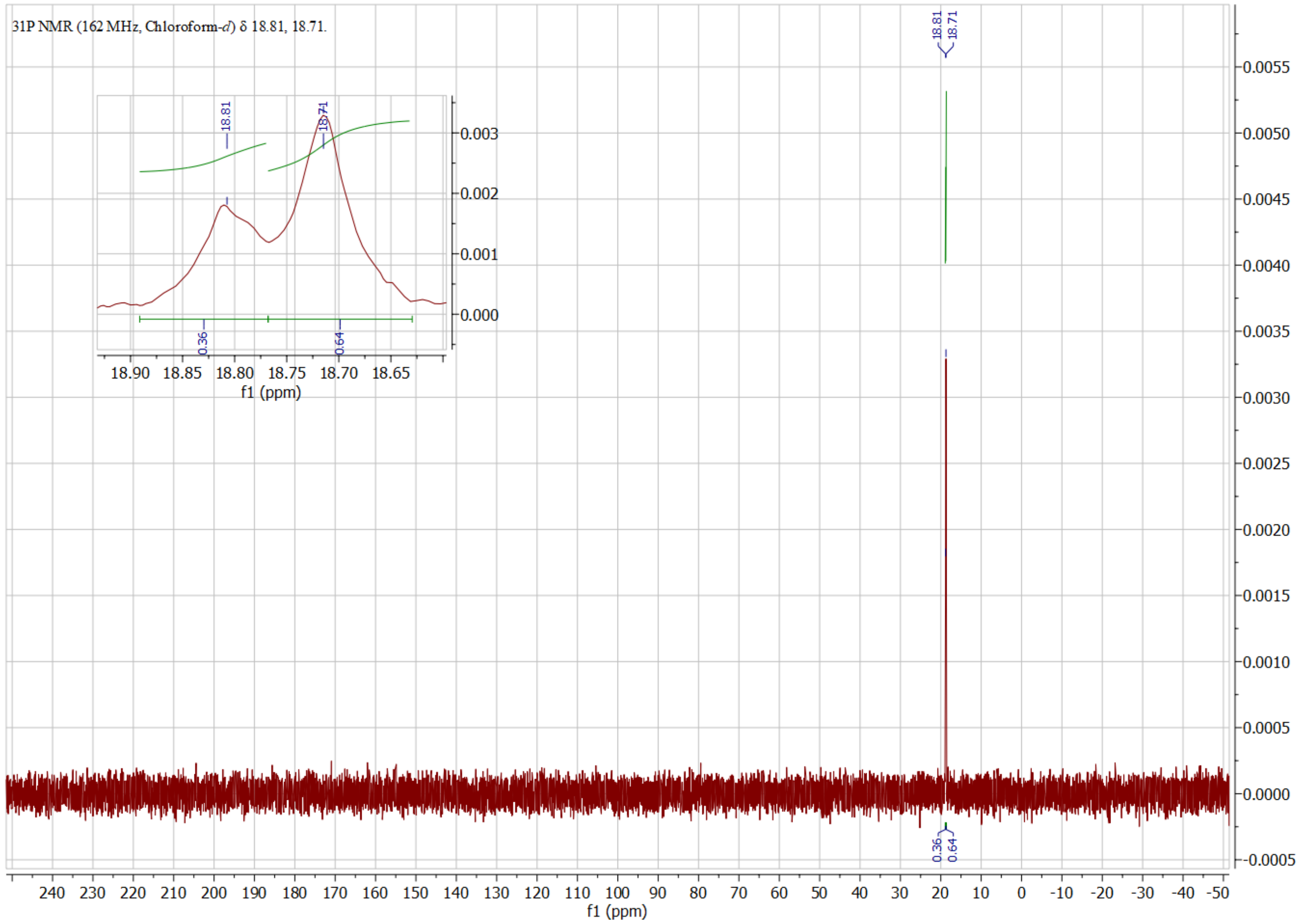
31P NMR (162 MHz, Chloroform-d) δ 18.81, 18.71.
18.81
18.71
0.36
0.64
f1 (ppm)

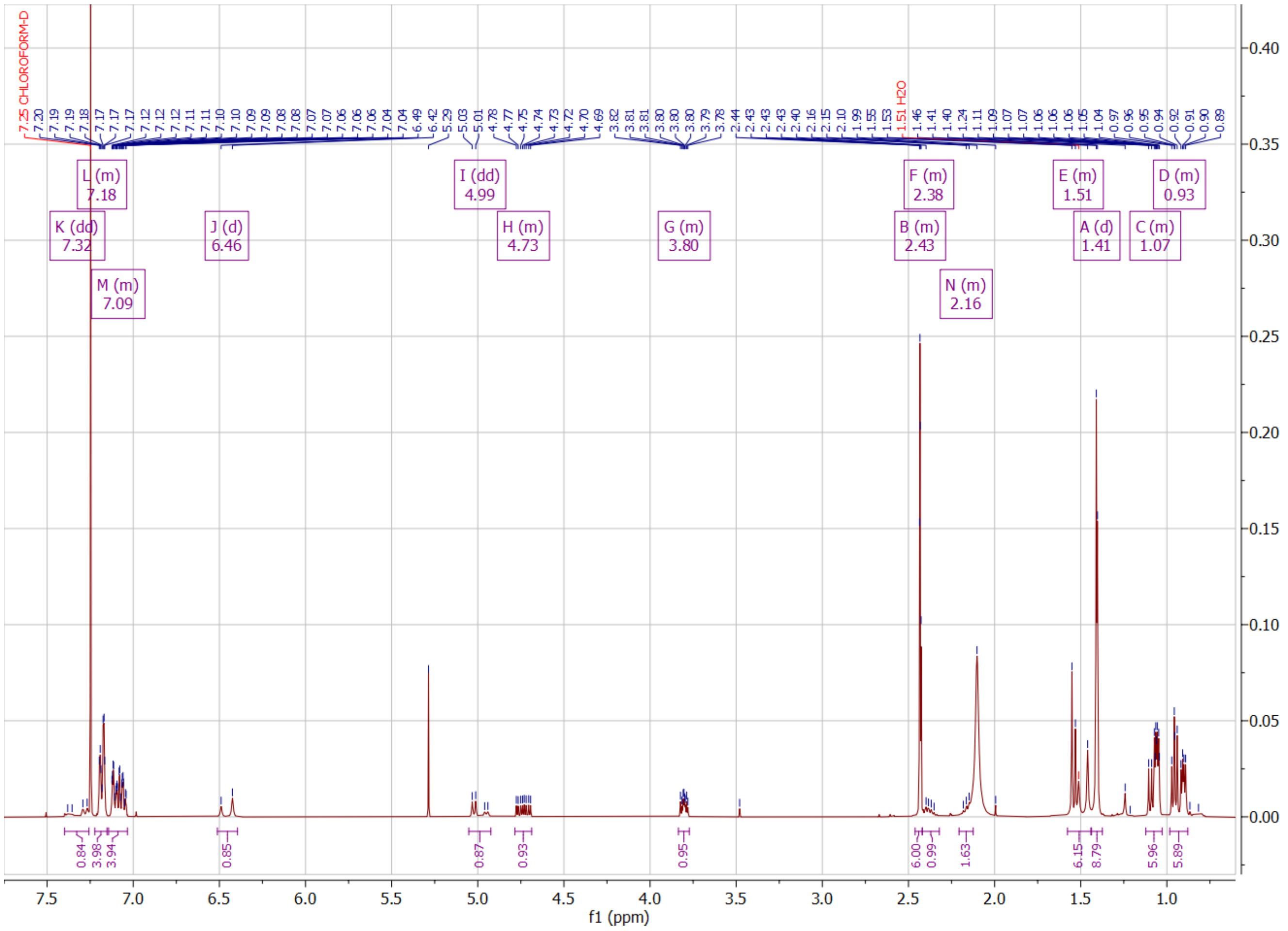
7.25 CHLOROFORM-D
1.51 H2O
L (m) 7.18
K (dd) 7.32
M (m) 7.09
J (d) 6.46
I (dd) 4.99
H (m) 4.73
G (m) 3.80
F (m) 2.38
B (m) 2.43
N (m) 2.16
E (m) 1.51
A (d) 1.41
D (m) 0.93
C (m) 1.07
f1 (ppm)

(5) Boc-L-Val-beta-Ala-Val^{P}(OPh-4-SCH$_3$)$_2$

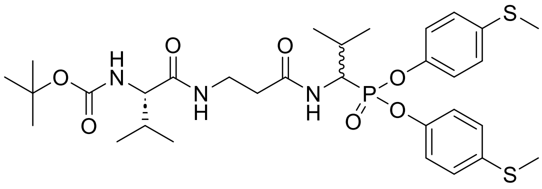

Name: *tert*-butyl ((2S)-1-((3-((1-(bis(4-(methylthio)phenoxy)phosphoryl)-2-methylpropyl)amino)-3-oxopropyl)amino)-3-methyl-1-oxobutan-2-yl)carbamate

^{31}P NMR (162 MHz, Chloroform-d) δ 20.82 (s, 50%), 18.45 (s, 50%).

^{1}H NMR (400 MHz, Chloroform-d) δ 7.39 (d, J = 9.3 Hz, 1H, NH), 7.23 – 6.97 (m, 8H, 8xArH), 6.93 – 6.70 (m, 1H, NH), 5.20 – 5.06 (m, 1H, NH), 4.77 – 4.59 (m, 1H, CHP), 3.87 – 3.72 (m, 1H, CH), 3.60 – 3.04 (m, 2H, CH$_2$), 2.53 – 2.40 (m, 7H, 2xSCH$_3$, CH), 2.39 – 2.22 (m, 2H, CH$_2$), 2.14 – 1.75 (m, 1H, CH), 1.41 (d, J = 6.3 Hz, 9H, Boc), 1.18 – 0.94 (m, 6H, 2xCH$_3$), 0.90 – 0.72 (m, 6H, 2xCH$_3$).

HRMS: $C_{31}H_{46}N_3O_7PS_2$ $[M+Na]^+$ calc. 690.2412 found 690.2378

HPLC: purity >98%; retention time 17.573 and 17.750 min

Chromatogram

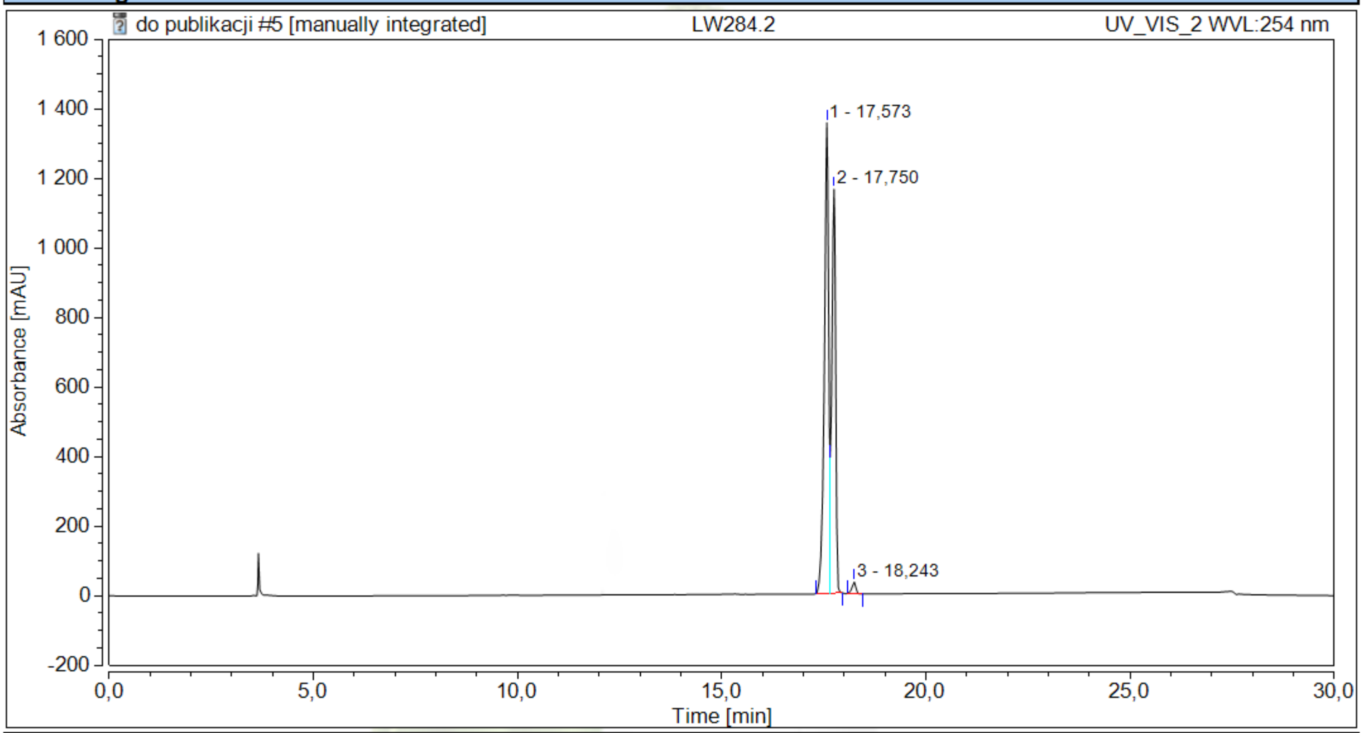

Integration Results

No.	Peak Name	Retention Time min	Area mAU*min	Height mAU	Relative Area %	Relative Height %	Amount n.a.
1		17,573	164,434	1352,920	56,16	53,11	n.a.
2		17,750	124,739	1161,031	42,61	45,58	n.a.
3		18,243	3,601	33,565	1,23	1,32	n.a.
**Total:**			**292,774**	**2547,516**	**100,00**	**100,00**	

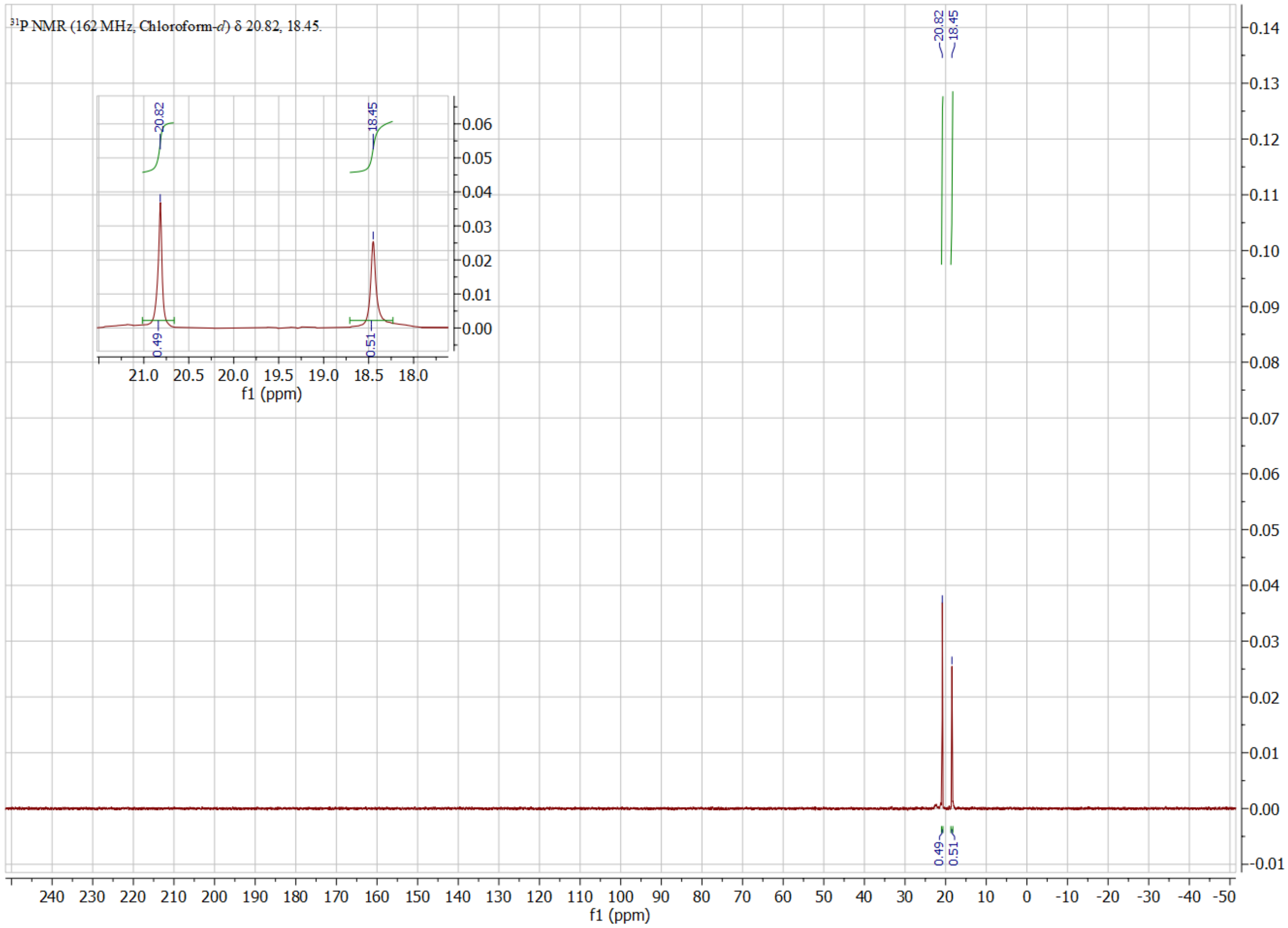
^{31}P NMR (162 MHz, Chloroform-d) δ 20.82, 18.45.
20.82
18.45
0.49
0.51
f1 (ppm)

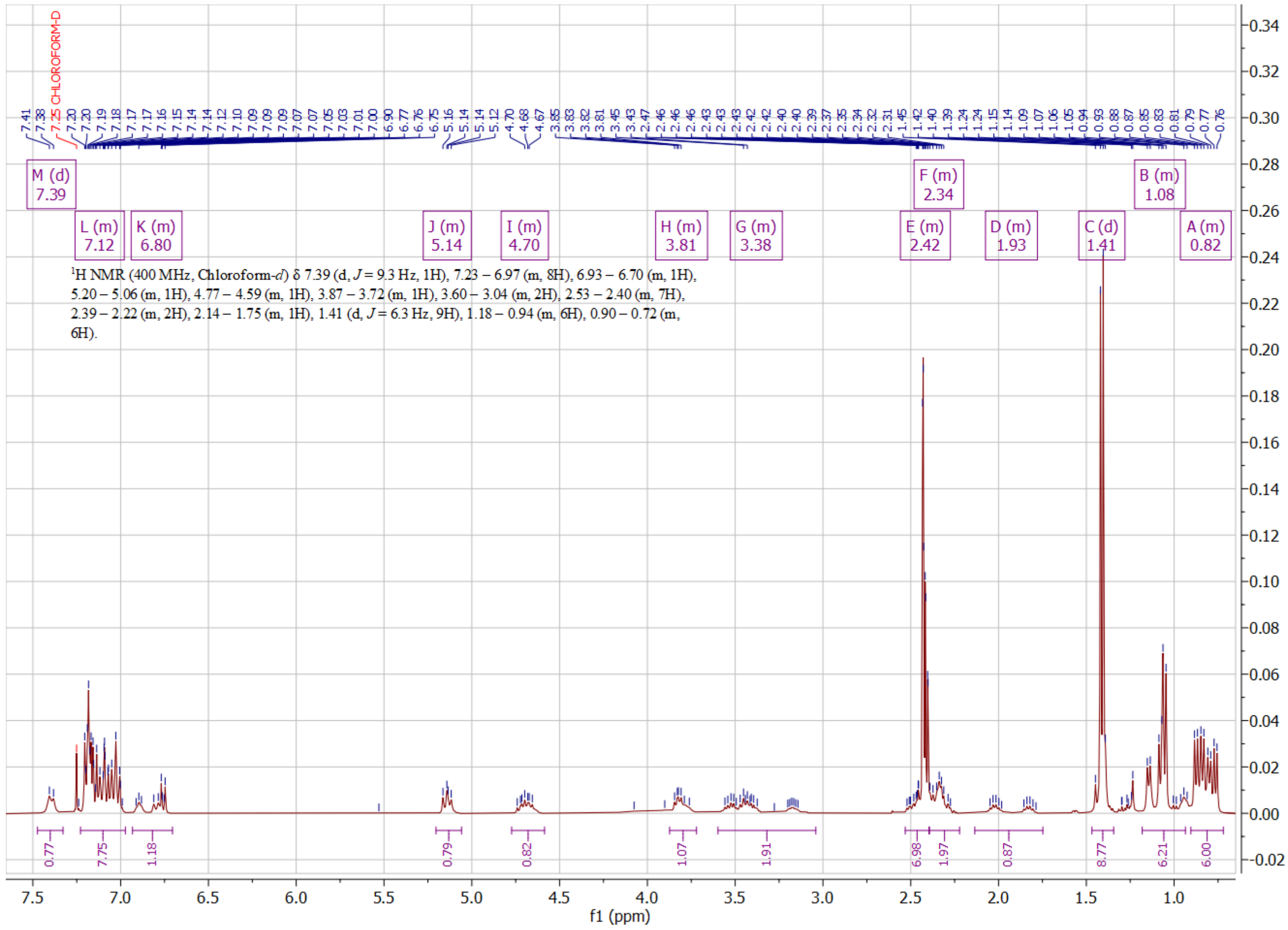
^{1}H NMR (400 MHz, Chloroform-d) δ 7.39 (d, J = 9.3 Hz, 1H), 7.23 – 6.97 (m, 8H), 6.93 – 6.70 (m, 1H), 5.20 – 5.06 (m, 1H), 4.77 – 4.59 (m, 1H), 3.87 – 3.72 (m, 1H), 3.60 – 3.04 (m, 2H), 2.53 – 2.40 (m, 7H), 2.39 – 2.22 (m, 2H), 2.14 – 1.75 (m, 1H), 1.41 (d, J = 6.3 Hz, 9H), 1.18 – 0.94 (m, 6H), 0.90 – 0.72 (m, 6H).
CHLOROFORM-D
M (d) 7.39
L (m) 7.12
K (m) 6.80
J (m) 5.14
I (m) 4.70
H (m) 3.81
G (m) 3.38
F (m) 2.34
E (m) 2.42
D (m) 1.93
C (d) 1.41
B (m) 1.08
A (m) 0.82
0.77
7.75
1.18
0.79
0.82
1.07
1.91
6.98
1.97
0.87
8.77
6.21
6.00
f1 (ppm)

(6) Boc-L-Val-L-Abu-Val^P(OPh-4-SCH$_3$)$_2$

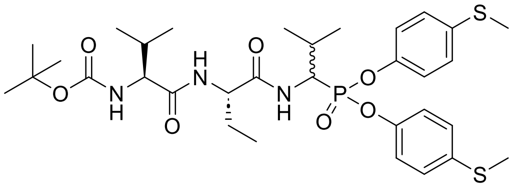

Name: *tert*-butyl ((2S)-1-(((2S)-1-((1-(bis(4-(methylthio)phenoxy)phosphoryl)-2-methylpropyl)amino)-1-oxobutan-2-yl)amino)-3-methyl-1-oxobutan-2-yl)carbamate

^{31}P NMR (162 MHz, Chloroform-d) δ 18.19 (s, 53%), 18.09 (s, 47%).

^{1}H NMR (400 MHz, Chloroform-d) δ 7.20 – 7.13 (m, 4H, 4xArH), 7.11 – 6.92 (m, 5H, 4xArH, NH), 6.61 (dd, J = 29.2, 7.8 Hz, 1H, NH), 5.10 (dd, J = 45.3, 8.3 Hz, 1H, NH), 4.79 – 4.65 (m, 1H, CHP), 4.49 – 4.41 (m, 1H, CH), 3.89 (dd, J = 8.4, 6.3 Hz, 1H, CH), 2.46 – 2.39 (m, 6H, 2xSCH$_3$), 2.41 – 2.30 (m, 1H, CH), 2.17 – 2.05 (m, 1H, CH), 1.98 – 1.73 (m, 1H, CH$_2$), 1.71 – 1.55 (m, 1H, CH$_2$), 1.41 (d, J = 6.2 Hz, 9H, Boc), 1.11 – 0.99 (m, 6H, 2xCH$_3$), 0.95 – 0.83 (m, 9H, 3xCH$_3$).

HRMS: $C_{32}H_{48}N_3O_7PS_2$ [M+Na]$^+$ calc. 704.2569 found 704.2588

HPLC: purity >99%; retention time 18.457 and 18.613 min

Chromatogram

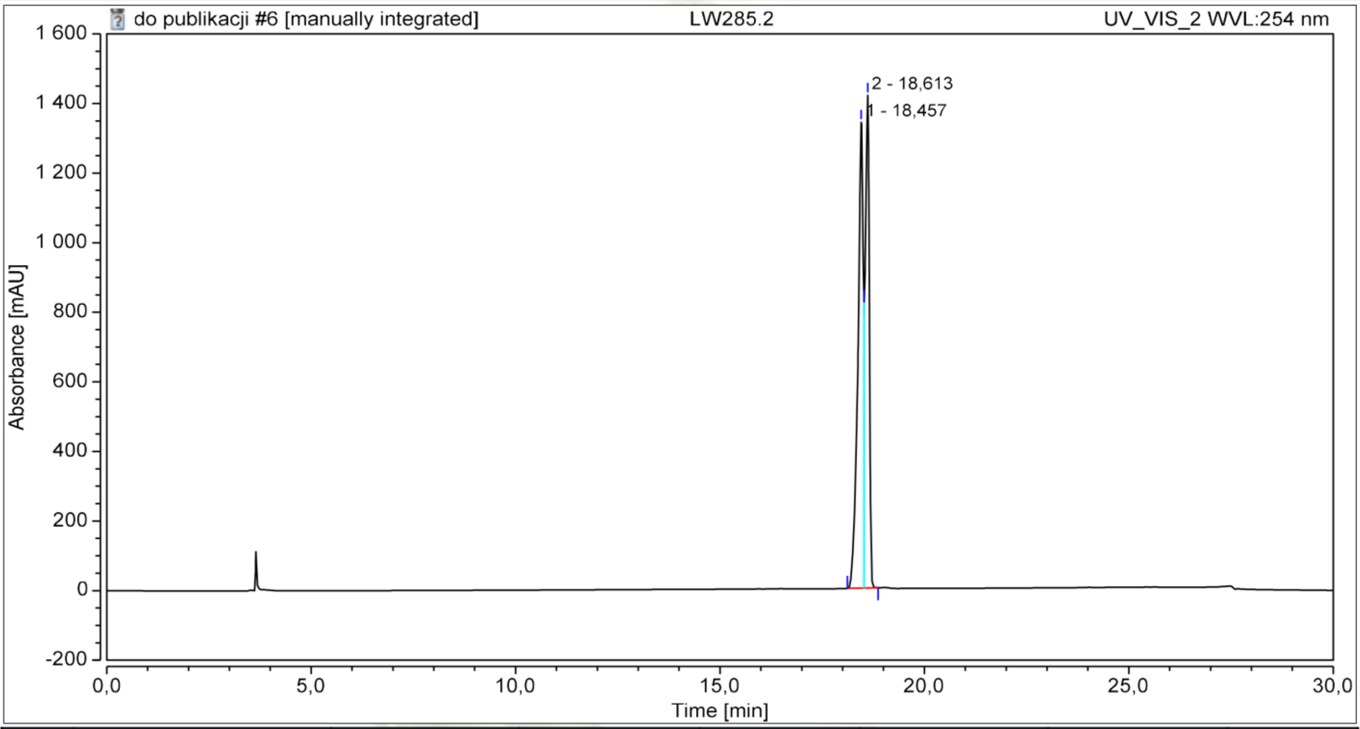

Integration Results

No.	Peak Name	Retention Time min	Area mAU*min	Height mAU	Relative Area %	Relative Height %	Amount n.a.
1		18,457	204,683	1338,952	55,49	48,61	n.a.
2		18,613	164,188	1415,650	44,51	51,39	n.a.
**Total:**			**368,871**	**2754,602**	**100,00**	**100,00**	

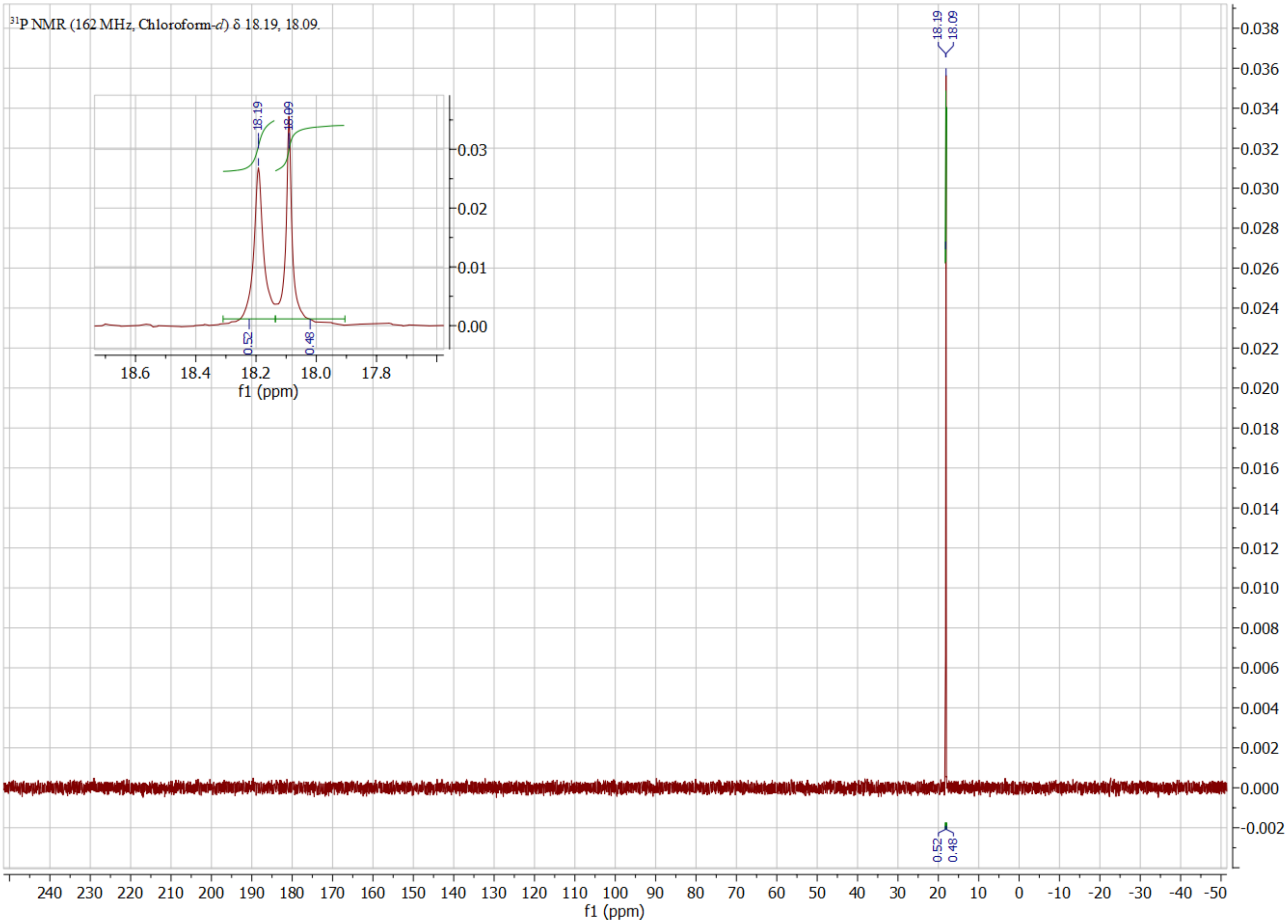
31P NMR (162 MHz, Chloroform-d) δ 18.19, 18.09.
18.19
18.09
0.52
0.48
f1 (ppm)

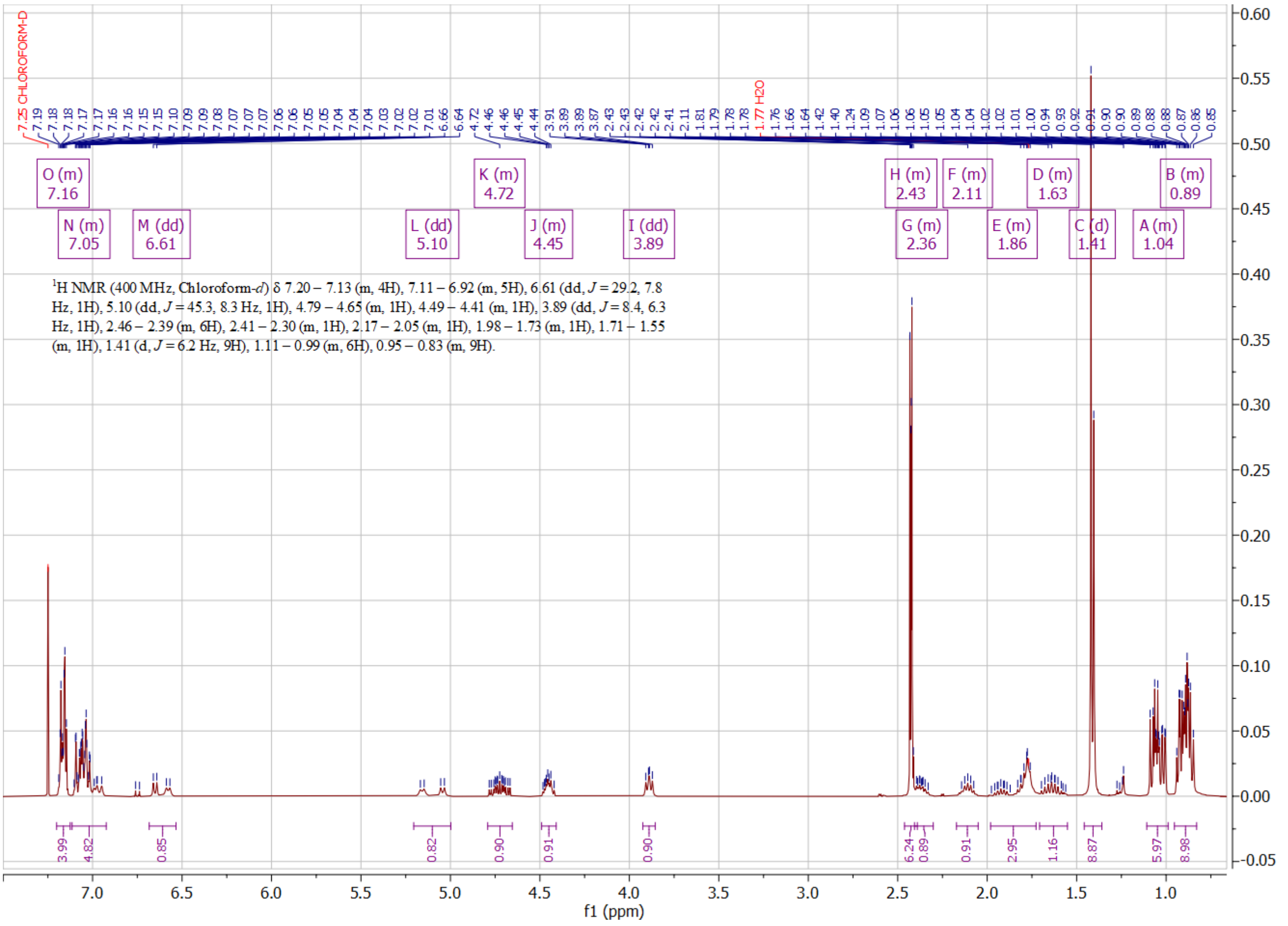
7.25 CHLOROFORM-D
1.77 H2O
O (m) 7.16
N (m) 7.05
M (dd) 6.61
L (dd) 5.10
K (m) 4.72
J (m) 4.45
I (dd) 3.89
H (m) 2.43
G (m) 2.36
F (m) 2.11
E (m) 1.86
D (m) 1.63
C (d) 1.41
B (m) 0.89
A (m) 1.04
1H NMR (400 MHz, Chloroform-d) δ 7.20 – 7.13 (m, 4H), 7.11 – 6.92 (m, 5H), 6.61 (dd, J = 29.2, 7.8 Hz, 1H), 5.10 (dd, J = 45.3, 8.3 Hz, 1H), 4.79 – 4.65 (m, 1H), 4.49 – 4.41 (m, 1H), 3.89 (dd, J = 8.4, 6.3 Hz, 1H), 2.46 – 2.39 (m, 6H), 2.41 – 2.30 (m, 1H), 2.17 – 2.05 (m, 1H), 1.98 – 1.73 (m, 1H), 1.71 – 1.55 (m, 1H), 1.41 (d, J = 6.2 Hz, 9H), 1.11 – 0.99 (m, 6H), 0.95 – 0.83 (m, 9H).
f1 (ppm)

(7) Boc-L-Val-D-Abu- Val^{P}(OPh-4-SCH$_3$)$_2$

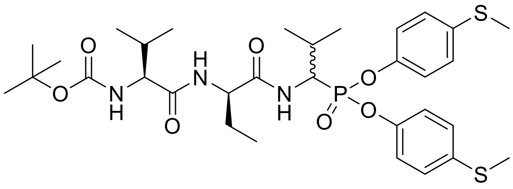

Name: *tert*-butyl ((2S)-1-(((2R)-1-((1-(bis(4-(methylthio)phenoxy)phosphoryl)-2-methylpropyl)amino)-1-oxobutan-2-yl)amino)-3-methyl-1-oxobutan-2-yl)carbamate

^{31}P NMR (162 MHz, Chloroform-d) δ 18.19 (s, 43%), 18.06 (s, 57%).

^{1}H NMR (400 MHz, Chloroform-d) δ 7.24 – 7.00 (m, 9H, 8xArH, NH), 6.75 (dd, J = 74.5, 7.7 Hz, 1H, NH), 5.18 (dd, J = 67.6, 8.5 Hz, 1H, NH), 4.79 – 4.64 (m, 1H, CHP), 4.53 – 4.42 (m, 1H, CH), 3.98 – 3.87 (m, 1H, CH), 2.47 – 2.41 (m, 6H, 2xSCH$_3$), 2.41 – 2.32 (m, 1H, CH), 2.17 – 2.03 (m, 1H, CH), 2.00 – 1.71 (m, 1H, CH$_2$), 1.68 – 1.52 (m, 1H, CH$_2$), 1.40 (d, J = 5.9 Hz, 9H, Boc), 1.02 (dd, J = 22.6, 6.2 Hz, 6H, 2xCH$_3$), 0.96 – 0.77 (m, 9H, 3xCH$_3$).

HRMS: $C_{32}H_{48}N_3O_7PS_2$ [M+Na]$^+$ calc. 704.2569 found 704.2545

HPLC: purity >99%; retention time 18.547 and 18.677 min

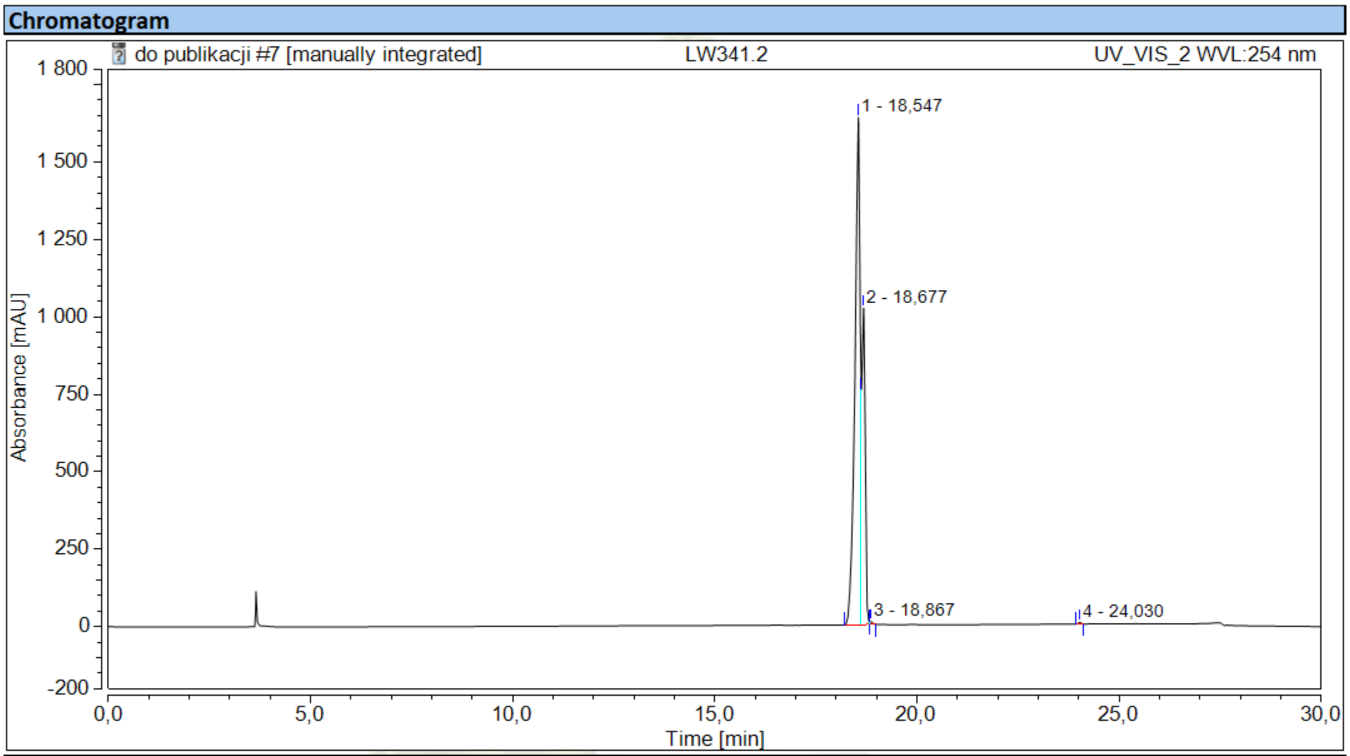

Integration Results

No.	Peak Name	Retention Time min	Area mAU*min	Height mAU	Relative Area %	Relative Height %	Amount n.a.
1		18,547	247,363	1637,453	70,98	61,23	n.a.
2		18,677	99,783	1021,828	28,63	38,21	n.a.
3		18,867	0,923	9,002	0,26	0,34	n.a.
4		24,030	0,442	5,874	0,13	0,22	n.a.
**Total:**			**348,511**	**2674,157**	**100,00**	**100,00**	

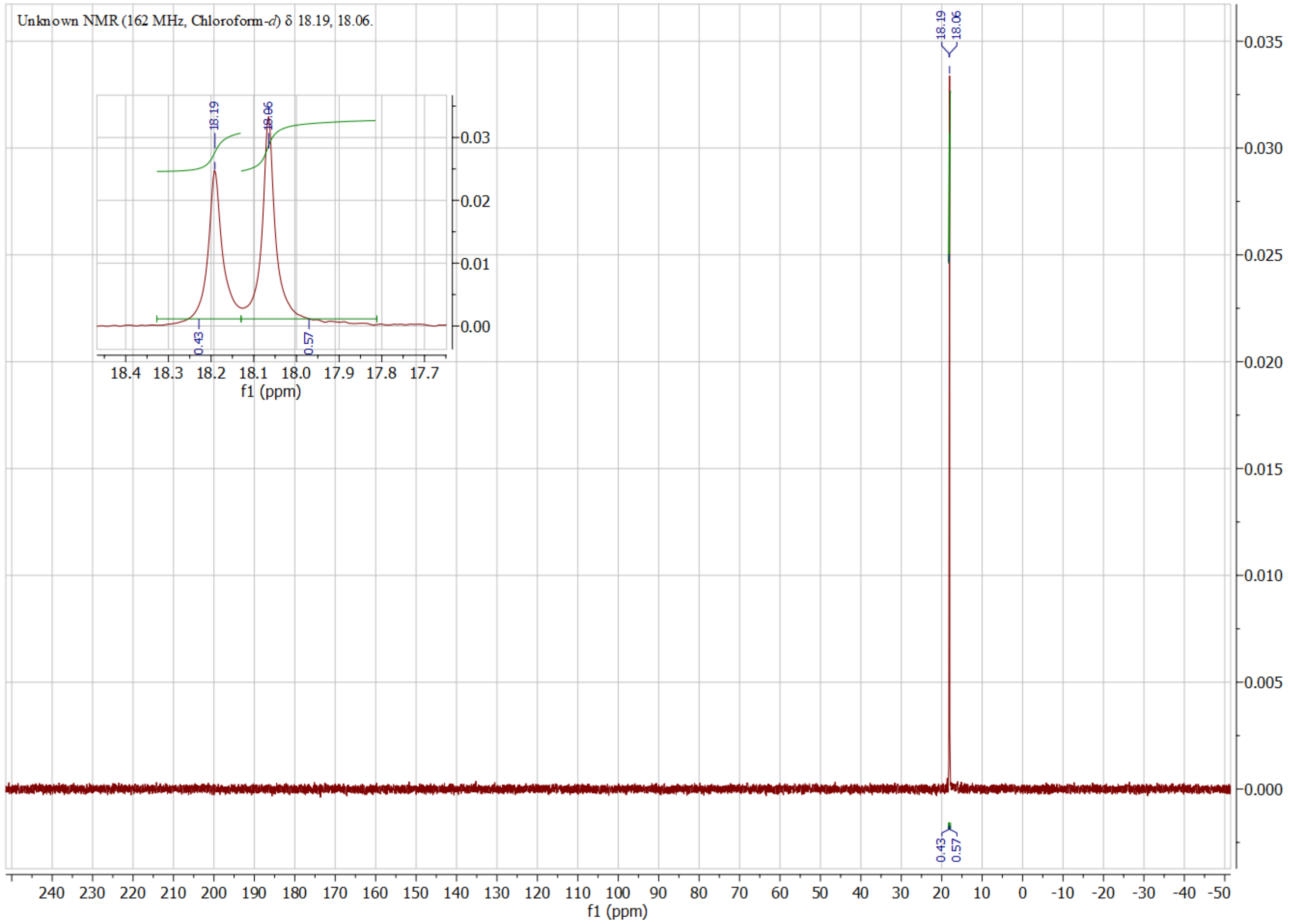

Unknown NMR (162 MHz, Chloroform-*d*) δ 18.19, 18.06.
f1 (ppm)

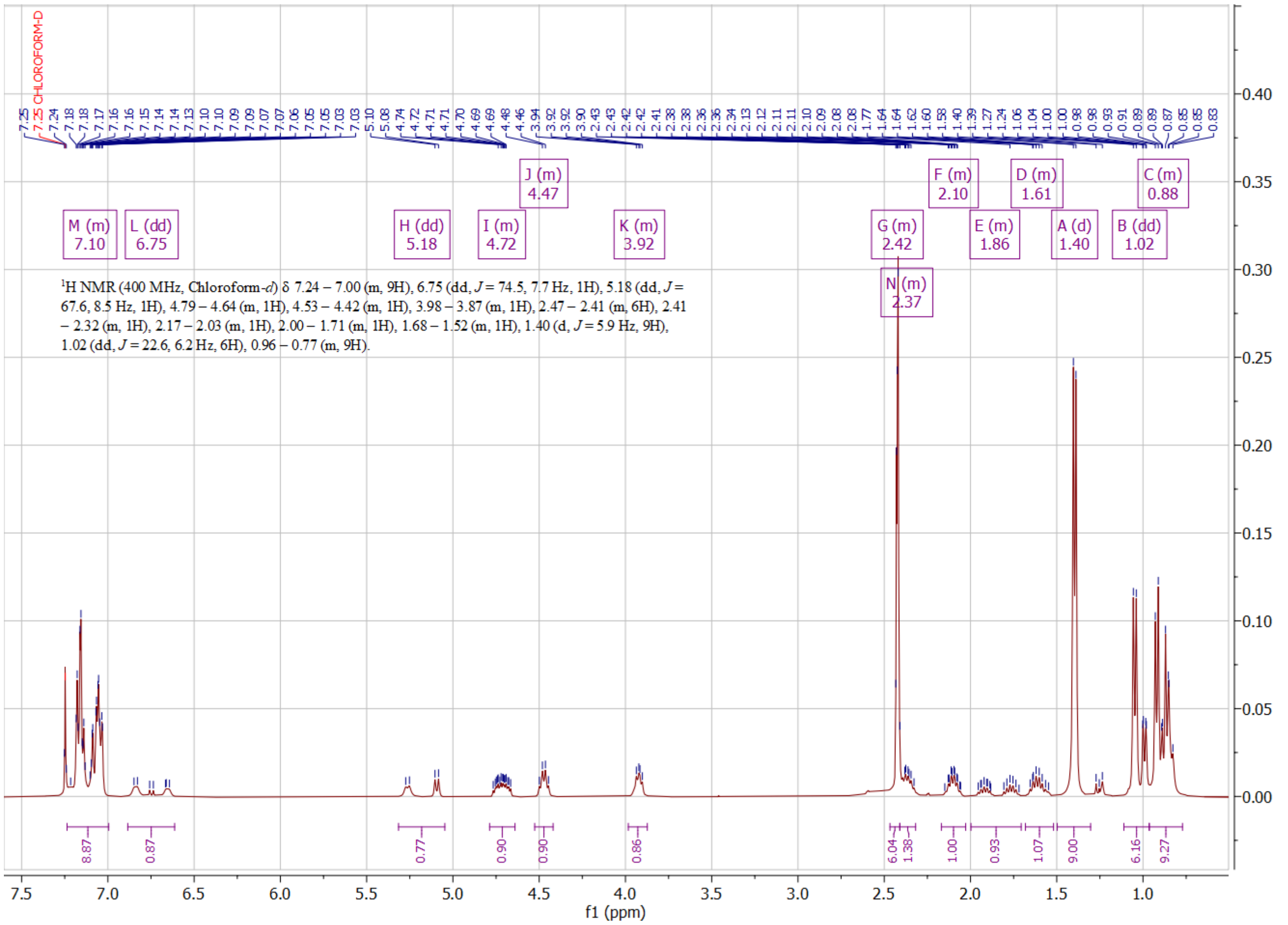

7.25 CHLOROFORM-D
M (m) 7.10
L (dd) 6.75
H (dd) 5.18
I (m) 4.72
J (m) 4.47
K (m) 3.92
G (m) 2.42
N (m) 2.37
F (m) 2.10
E (m) 1.86
D (m) 1.61
A (d) 1.40
B (dd) 1.02
C (m) 0.88
^{1}H NMR (400 MHz, Chloroform-*d*) δ 7.24 – 7.00 (m, 9H), 6.75 (dd, *J* = 74.5, 7.7 Hz, 1H), 5.18 (dd, *J* = 67.6, 8.5 Hz, 1H), 4.79 – 4.64 (m, 1H), 4.53 – 4.42 (m, 1H), 3.98 – 3.87 (m, 1H), 2.47 – 2.41 (m, 6H), 2.41 – 2.32 (m, 1H), 2.17 – 2.03 (m, 1H), 2.00 – 1.71 (m, 1H), 1.68 – 1.52 (m, 1H), 1.40 (d, *J* = 5.9 Hz, 9H), 1.02 (dd, *J* = 22.6, 6.2 Hz, 6H), 0.96 – 0.77 (m, 9H).
f1 (ppm)

(8) Boc-L-Val-L-Nva- Val^P(OPh-4-SCH$_3$)$_2$

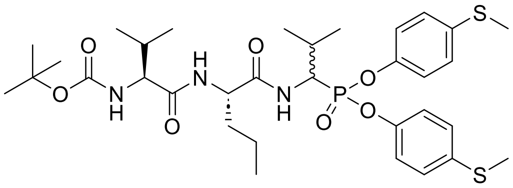

Name: *tert*-butyl ((2S)-1-(((2S)-1-((1-(bis(4-(methylthio)phenoxy)phosphoryl)-2-methylpropyl)amino)-1-oxopentan-2-yl)amino)-3-methyl-1-oxobutan-2-yl)carbamate
^{31}P NMR (162 MHz, Chloroform-d) δ 18.17 (s, 51%), 18.02 (s, 49%).
^{1}H NMR (400 MHz, Chloroform-d) δ 7.21 – 6.98 (m, 9H, 8xArH, NH), 6.64 (dd, J = 27.2, 7.9 Hz, 1H, NH), 5.18 (dd, J = 64.3, 8.5 Hz, 1H, NH), 4.81 – 4.65 (m, 1H, CHP), 4.57 – 4.48 (m, 1H, CH), 3.94 – 3.82 (m, 1H, CH), 2.42 (d, J = 3.6 Hz, 6H, 2xSCH$_3$), 2.40 – 2.30 (m, 1H, CH), 2.15 – 2.04 (m, 1H, CH), 1.88 – 1.48 (m, 2H, CH$_2$), 1.41 (d, J = 5.4 Hz, 9H, Boc), 1.35 – 1.20 (m, 2H, CH$_2$), 1.10 – 0.98 (m, 6H, 2xCH$_3$), 0.93 – 0.77 (m, 9H, 3xCH$_3$).
HRMS: $C_{33}H_{50}N_3O_7PS_2$ [M+Na]$^+$ calc. 718.2725 found 718.2728
HPLC: purity >99%; retention time 18.913 and 19.127 min

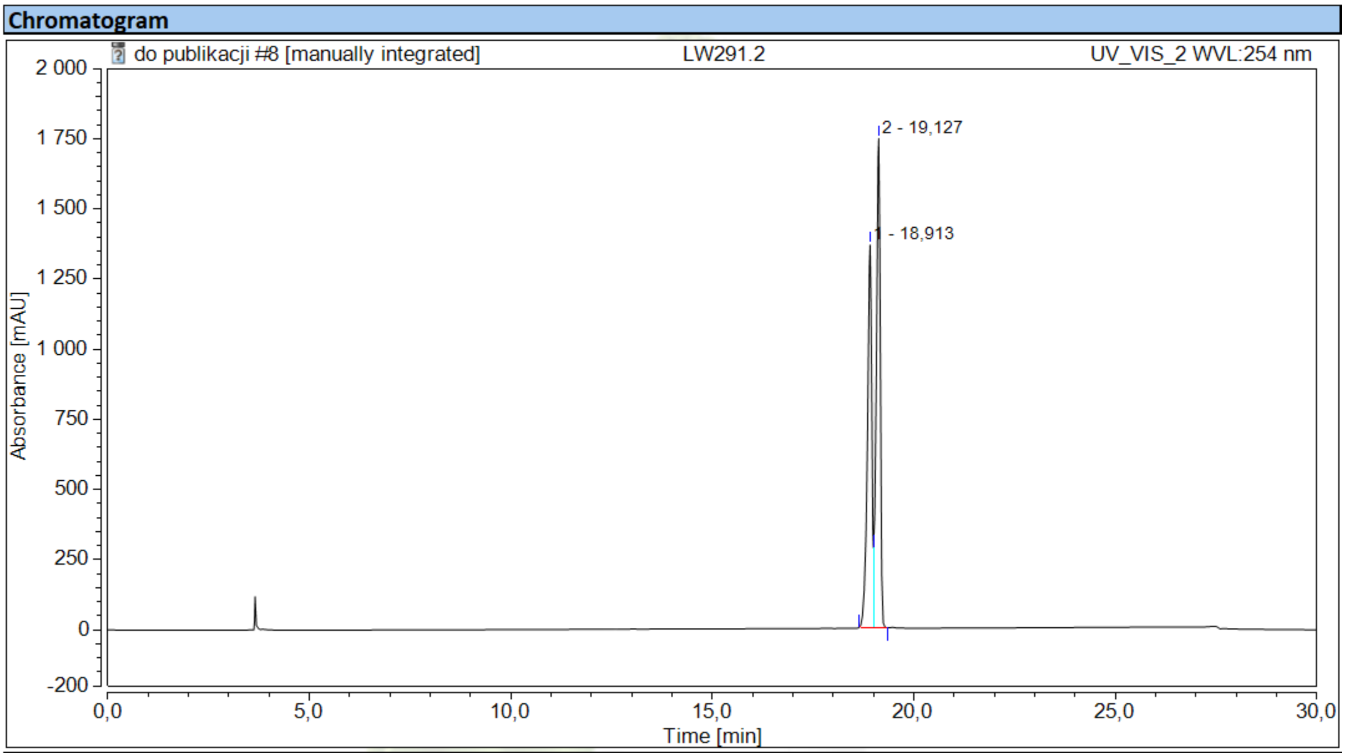

Integration Results

No.	Peak Name	Retention Time min	Area mAU*min	Height mAU	Relative Area %	Relative Height %	Amount n.a.
1		18,913	168,975	1362,429	45,90	43,88	n.a.
2		19,127	199,148	1742,682	54,10	56,12	n.a.
**Total:**			**368,123**	**3105,111**	**100,00**	**100,00**	

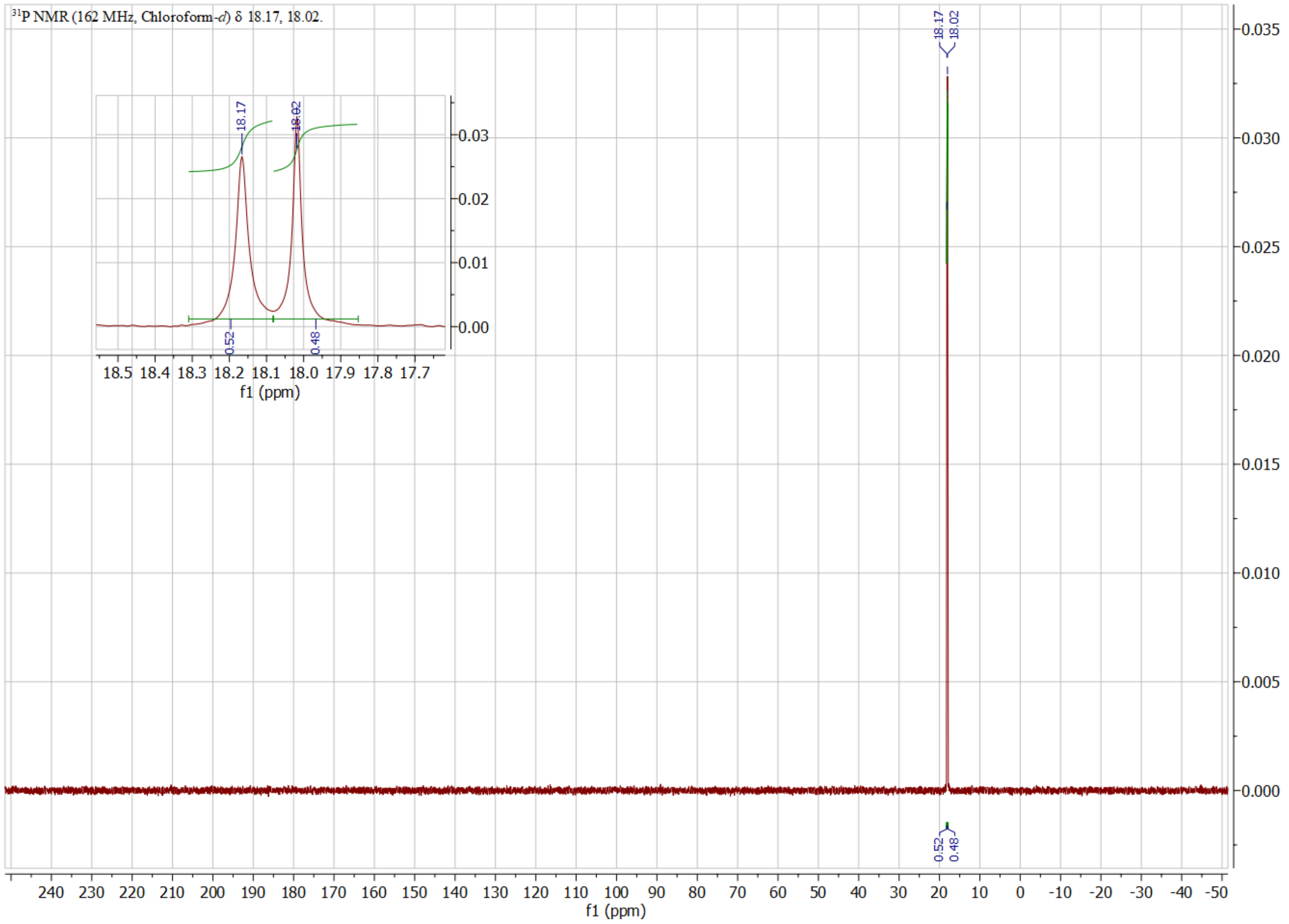
^{31}P NMR (162 MHz, Chloroform-*d*) δ 18.17, 18.02.
f1 (ppm)

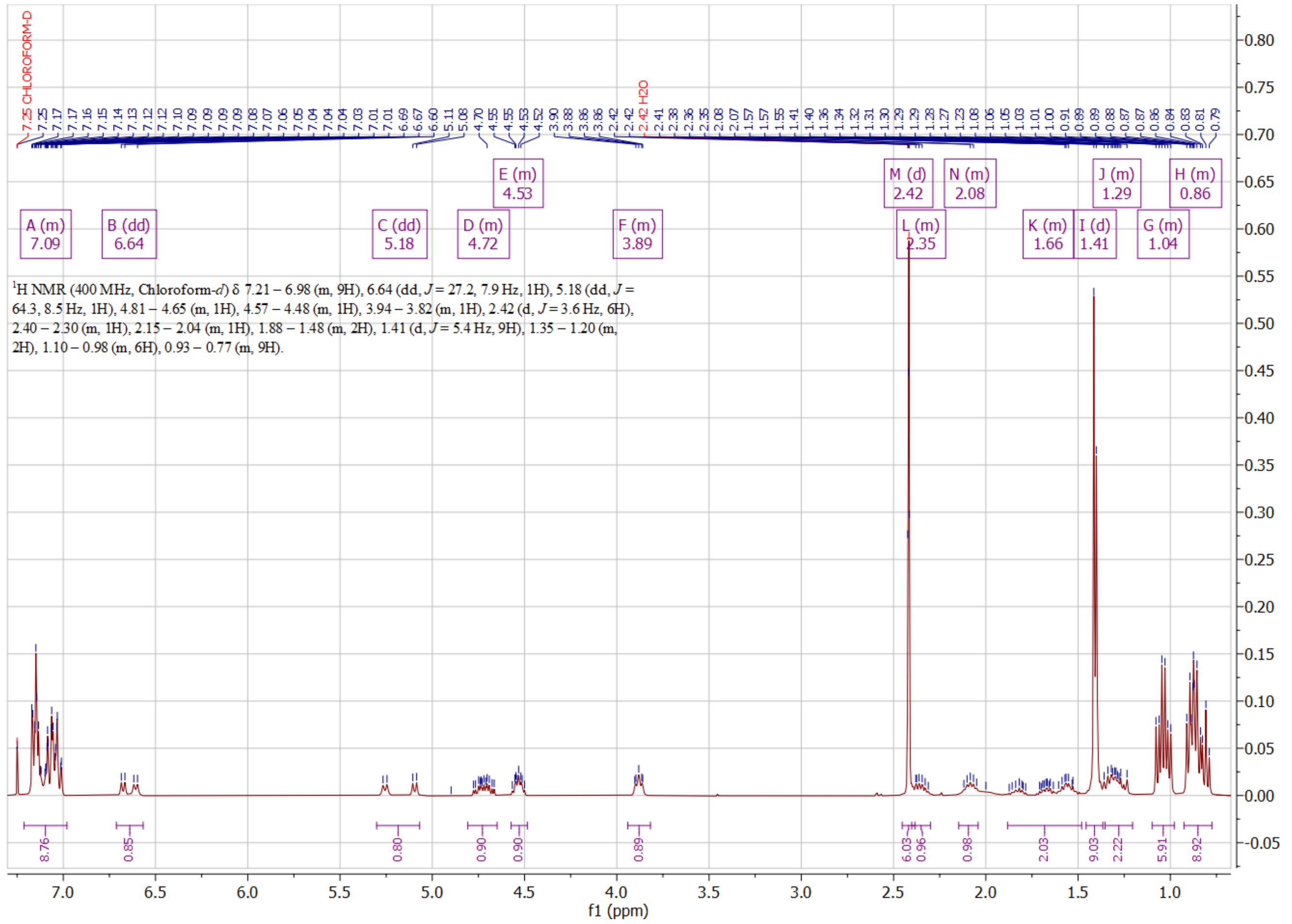
^{1}H NMR (400 MHz, Chloroform-*d*) δ 7.21 – 6.98 (m, 9H), 6.64 (dd, *J* = 27.2, 7.9 Hz, 1H), 5.18 (dd, *J* = 64.3, 8.5 Hz, 1H), 4.81 – 4.65 (m, 1H), 4.57 – 4.48 (m, 1H), 3.94 – 3.82 (m, 1H), 2.42 (d, *J* = 3.6 Hz, 6H), 2.40 – 2.30 (m, 1H), 2.15 – 2.04 (m, 1H), 1.88 – 1.48 (m, 2H), 1.41 (d, *J* = 5.4 Hz, 9H), 1.35 – 1.20 (m, 2H), 1.10 – 0.98 (m, 6H), 0.93 – 0.77 (m, 9H).
f1 (ppm)

(9) Boc-L-Val-D-Nva- Val^{P}(OPh-4-SCH$_3$)$_2$

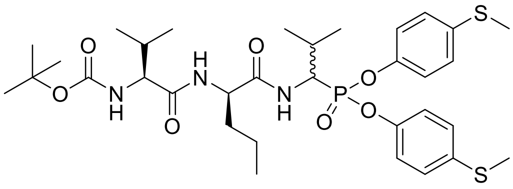

Name: *tert*-butyl ((2S)-1-(((2R)-1-((1-(bis(4-(methylthio)phenoxy)phosphoryl)-2-methylpropyl)amino)-1-oxopentan-2-yl)amino)-3-methyl-1-oxobutan-2-yl)carbamate

^{31}P NMR (162 MHz, Chloroform-d) δ 18.31 (s, 47%), 18.07 (s, 53%).

^{1}H NMR (400 MHz, Chloroform-d) δ 7.22 – 7.14 (m, 4H, 4xArH), 7.12 – 7.02 (m, 4H, 4xArH), 7.03 – 6.93 (m, 1H, NH), 6.56 (dd, J = 54.4, 8.0 Hz, 1H, NH), 5.06 (dd, J = 45.0, 8.3 Hz, 1H, NH), 4.77 – 4.65 (m, 1H, CHP), 4.51 – 4.40 (m, 1H, CH), 3.95 – 3.87 (m, 1H, CH), 2.43 (dd, J = 2.2, 0.8 Hz, 6H, 3xSCH$_3$), 2.41 – 2.30 (m, 1H, CH), 2.19 – 2.06 (m, 1H, CH), 1.85 – 1.66 (m, 1H, CH$_2$), 1.63 – 1.47 (m, 1H, CH$_2$), 1.40 (d, J = 6.9 Hz, 9H, Boc), 1.36 – 1.22 (m, 2H, CH$_2$), 1.10 – 0.97 (m, 6H, 2xCH$_3$), 0.96 – 0.77 (m, 9H, 3xCH$_3$).

HRMS: $C_{33}H_{50}N_3O_7PS_2$ [M+Na]$^+$ calc. 718.2725 found 718.2706

HPLC: purity >99%; retention time 19.023 and 19.203 min

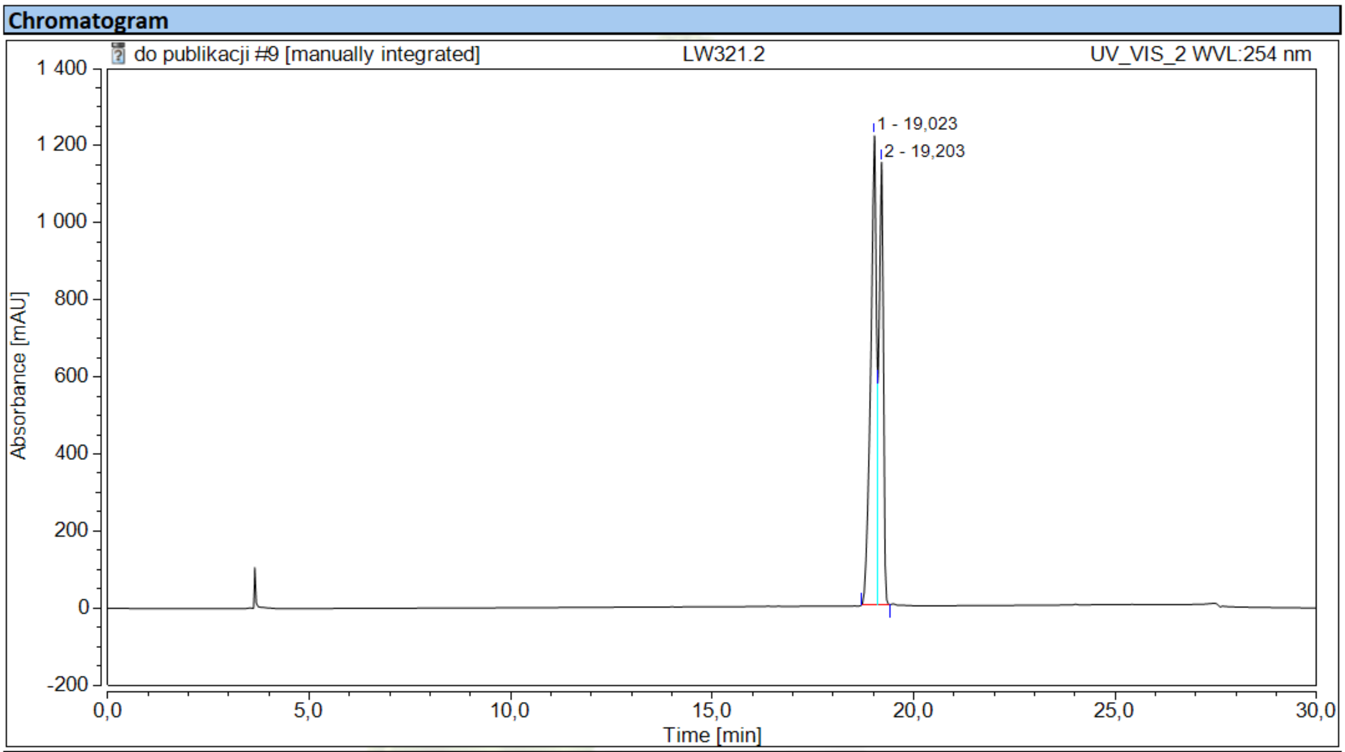

Integration Results

No.	Peak Name	Retention Time min	Area mAU*min	Height mAU	Relative Area %	Relative Height %	Amount n.a.
1		19,023	201,180	1217,819	58,41	51,46	n.a.
2		19,203	143,226	1148,646	41,59	48,54	n.a.
**Total:**			**344,407**	**2366,465**	**100,00**	**100,00**	

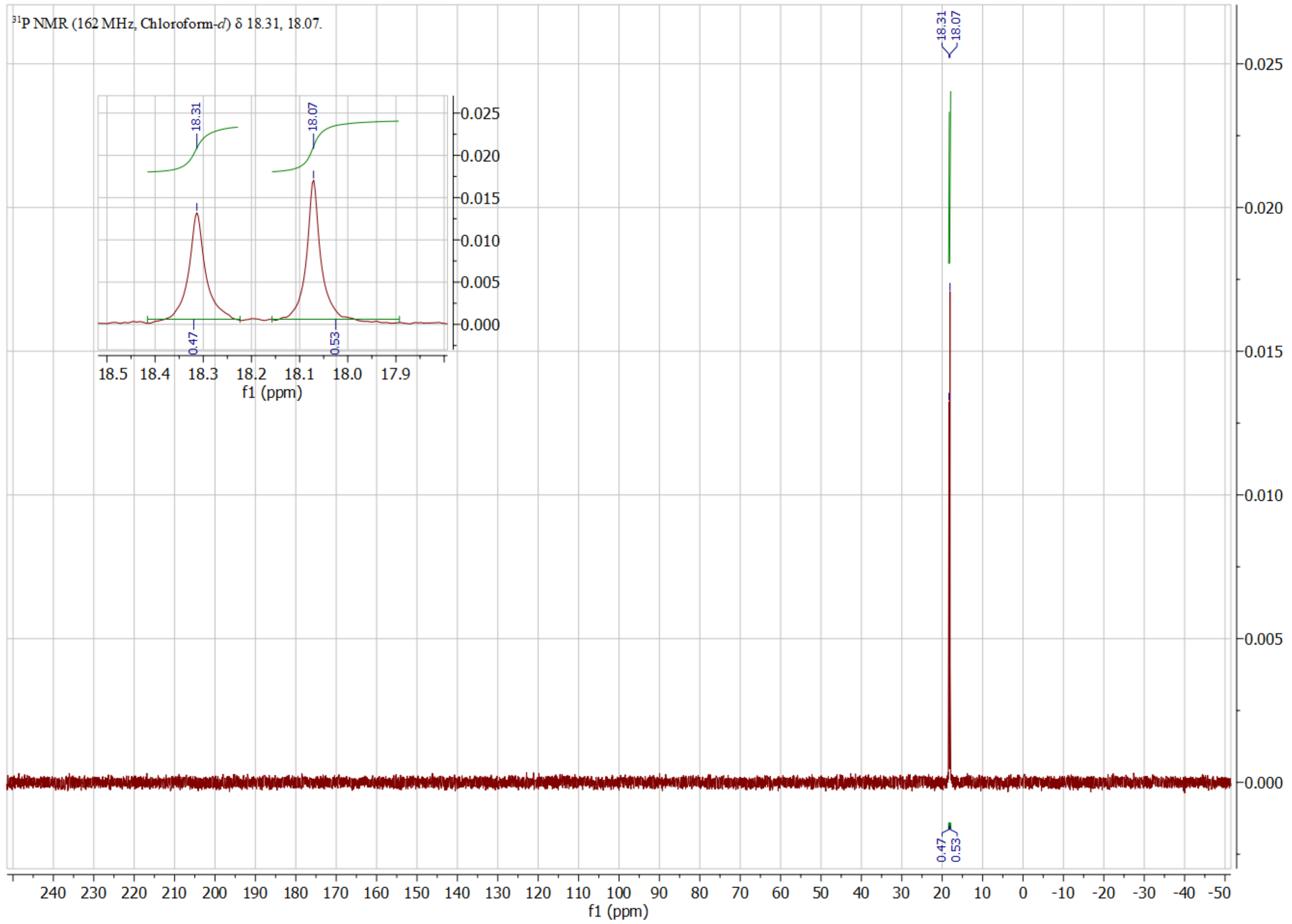

31P NMR (162 MHz, Chloroform-d) δ 18.31, 18.07.
18.31
18.07
0.47
0.53
f1 (ppm)

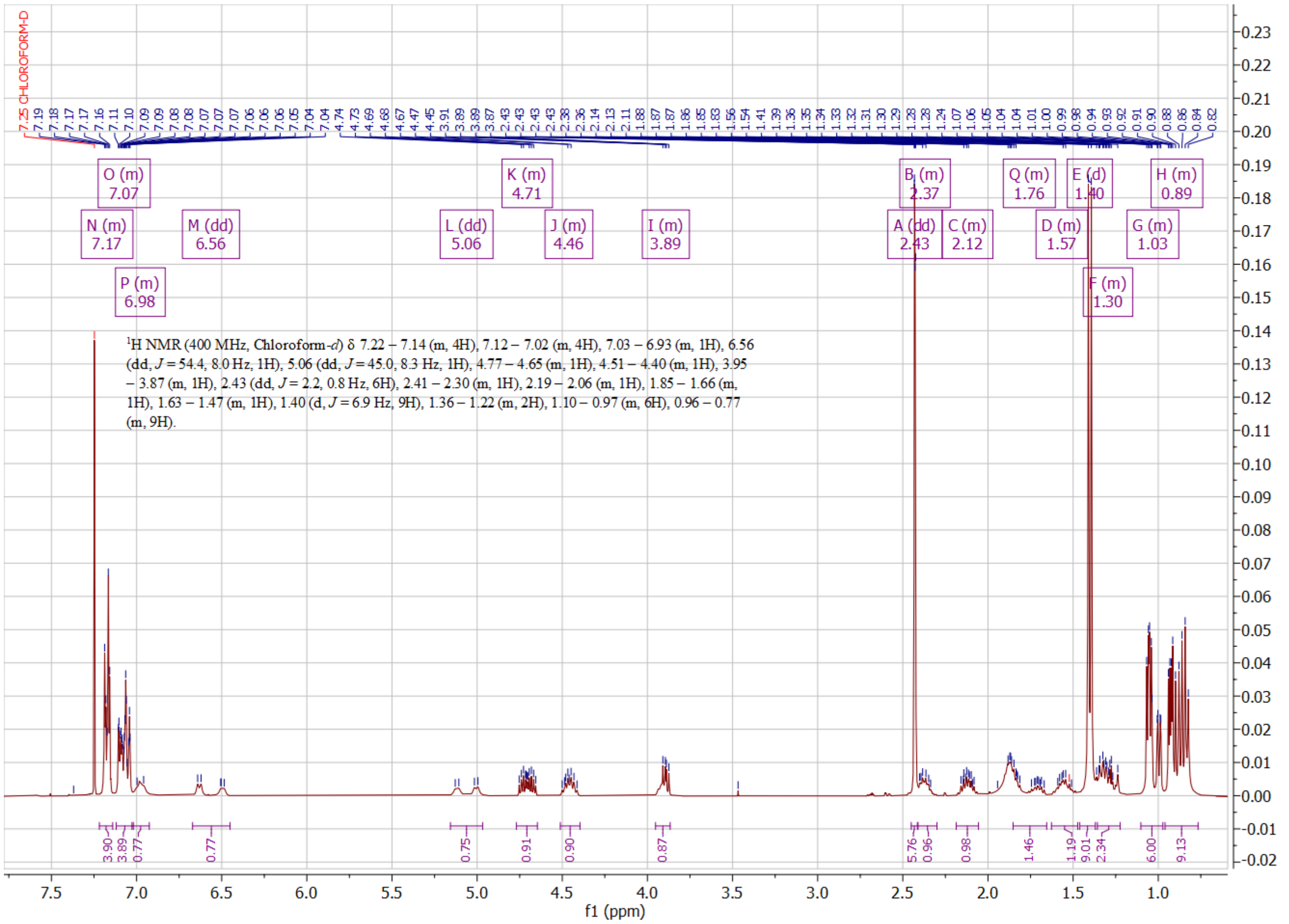

7.25 CHLOROFORM-D
1H NMR (400 MHz, Chloroform-d) δ 7.22 – 7.14 (m, 4H), 7.12 – 7.02 (m, 4H), 7.03 – 6.93 (m, 1H), 6.56 (dd, J = 54.4, 8.0 Hz, 1H), 5.06 (dd, J = 45.0, 8.3 Hz, 1H), 4.77 – 4.65 (m, 1H), 4.51 – 4.40 (m, 1H), 3.95 – 3.87 (m, 1H), 2.43 (dd, J = 2.2, 0.8 Hz, 6H), 2.41 – 2.30 (m, 1H), 2.19 – 2.06 (m, 1H), 1.85 – 1.66 (m, 1H), 1.63 – 1.47 (m, 1H), 1.40 (d, J = 6.9 Hz, 9H), 1.36 – 1.22 (m, 2H), 1.10 – 0.97 (m, 6H), 0.96 – 0.77 (m, 9H).
O (m) 7.07
N (m) 7.17
P (m) 6.98
M (dd) 6.56
L (dd) 5.06
K (m) 4.71
J (m) 4.46
I (m) 3.89
B (m) 2.37
A (dd) 2.43
C (m) 2.12
Q (m) 1.76
D (m) 1.57
E (d) 1.40
F (m) 1.30
G (m) 1.03
H (m) 0.89
f1 (ppm)

(10) Boc-L-Val-L-Val-Val^P(OPh-4-SCH$_3$)$_2$

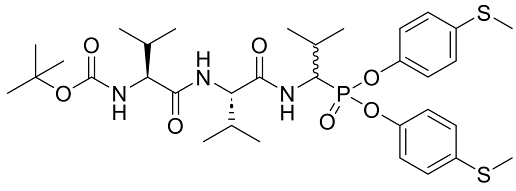

Name: *tert*-butyl ((2S)-1-(((2S)-1-((1-(bis(4-(methylthio)phenoxy)phosphoryl)-2-methylpropyl)amino)-3-methyl-1-oxobutan-2-yl)amino)-3-methyl-1-oxobutan-2-yl)carbamate

^{31}P NMR (162 MHz, Chloroform-d) δ 18.36 (s).

^{1}H NMR (400 MHz, Chloroform-d) δ 7.21 – 7.13 (m, 4H, 4xArH), 7.11 – 7.00 (m, 4H, 4xArH), 6.92 – 6.80 (m, 1H, NH), 6.62 (dd, J = 22.7, 8.5 Hz, 1H, NH), 5.09 (dd, J = 64.8, 8.1 Hz, 1H, NH), 4.80 – 4.68 (m, 1H, CHP), 4.42 – 4.31 (m, 1H, CH), 3.89 – 3.83 (m, 1H, CH), 2.45 – 2.41 (m, 6H, 2xSCH$_3$), 2.41 – 2.31 (m, 1H, CH), 2.31 – 2.07 (m, 2H, 2xCH), 1.41 (d, J = 9.8 Hz, 9H, Boc), 1.12 – 0.84 (m, 18H, 6xCH$_3$).

HRMS: $C_{33}H_{50}N_3O_7PS_2$ [M+Na]$^+$ calc. 718.2725 found 718.2733

HPLC: purity >99%; retention time 18.857 and 19.063 min

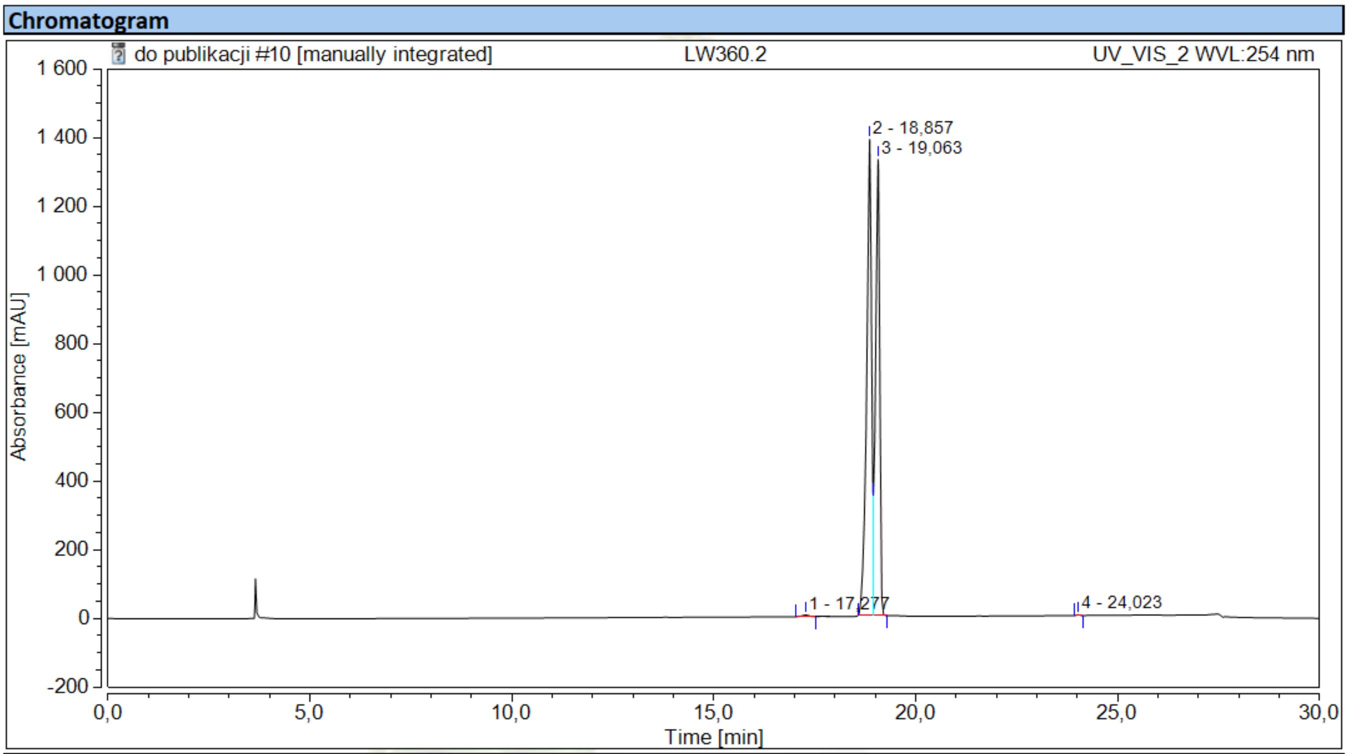

Integration Results

No.	Peak Name	Retention Time min	Area mAU*min	Height mAU	Relative Area %	Relative Height %	Amount n.a.
1		17,277	1,007	5,017	0,28	0,18	n.a.
2		18,857	195,298	1386,650	55,09	50,93	n.a.
3		19,063	158,019	1328,209	44,57	48,78	n.a.
4		24,023	0,213	2,824	0,06	0,10	n.a.
**Total:**			**354,538**	**2722,699**	**100,00**	**100,00**	

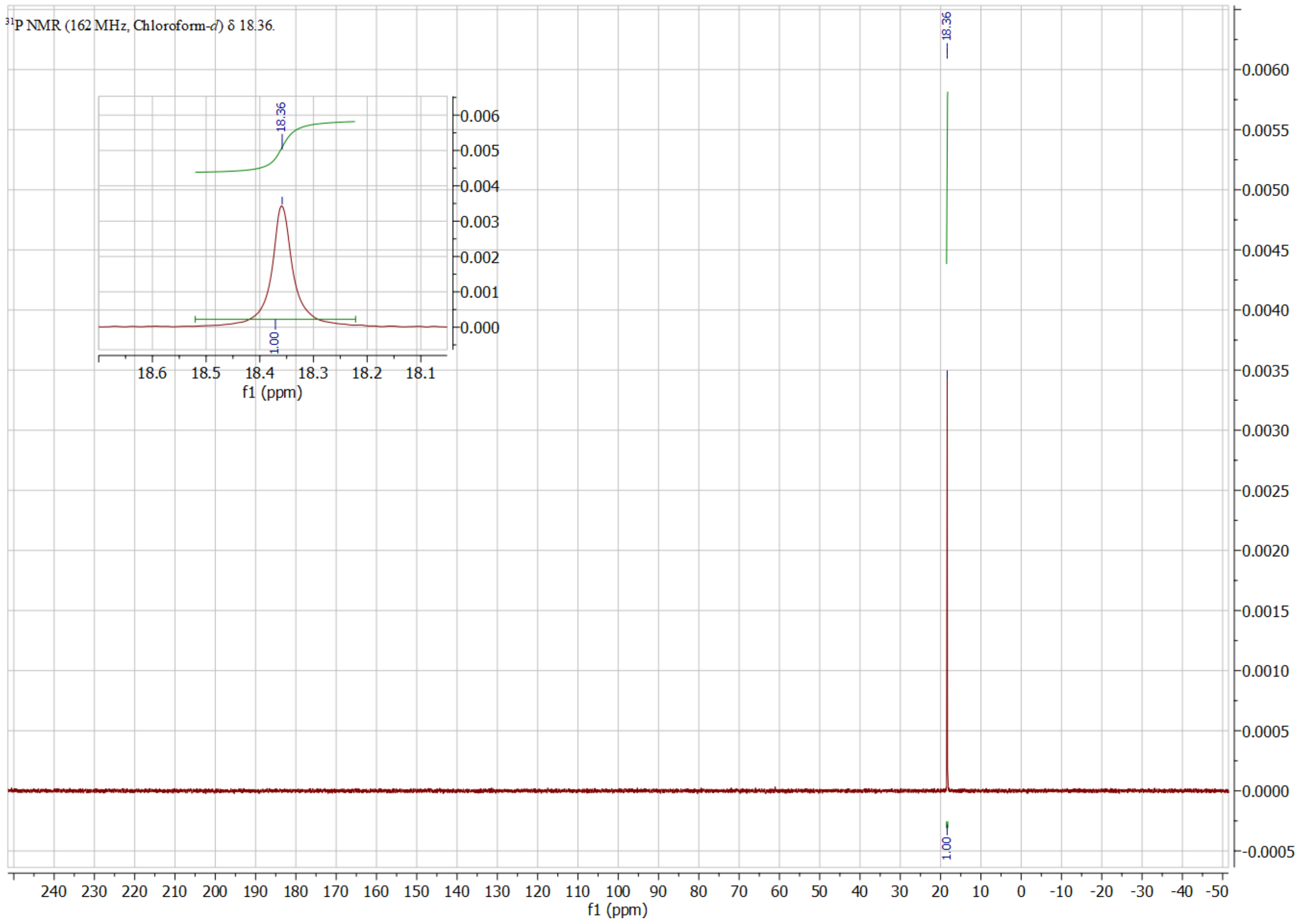

31P NMR (162 MHz, Chloroform-d) δ 18.36.
f1 (ppm)

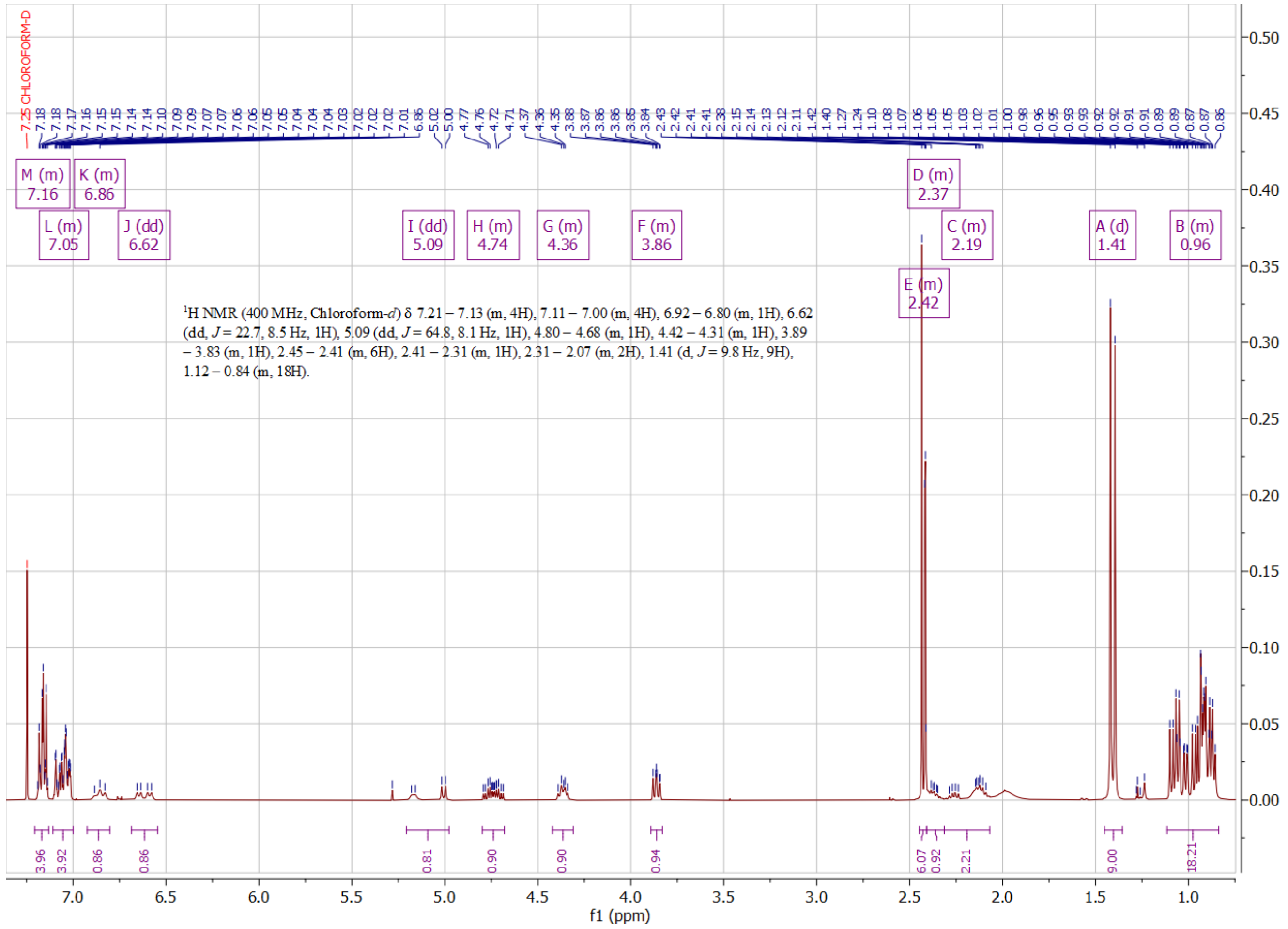

1H NMR (400 MHz, Chloroform-d) δ 7.21 – 7.13 (m, 4H), 7.11 – 7.00 (m, 4H), 6.92 – 6.80 (m, 1H), 6.62 (dd, J = 22.7, 8.5 Hz, 1H), 5.09 (dd, J = 64.8, 8.1 Hz, 1H), 4.80 – 4.68 (m, 1H), 4.42 – 4.31 (m, 1H), 3.89 – 3.83 (m, 1H), 2.45 – 2.41 (m, 6H), 2.41 – 2.31 (m, 1H), 2.31 – 2.07 (m, 2H), 1.41 (d, J = 9.8 Hz, 9H), 1.12 – 0.84 (m, 18H).
f1 (ppm)

(11) Boc-L-Val-D-Val- Val^P(OPh-4-SCH$_3$)$_2$

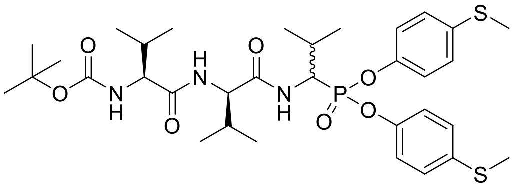

Name: *tert*-butyl ((2S)-1-(((2R)-1-((1-(bis(4-(methylthio)phenoxy)phosphoryl)-2-methylpropyl)amino)-3-methyl-1-oxobutan-2-yl)amino)-3-methyl-1-oxobutan-2-yl)carbamate

^{31}P NMR (162 MHz, Chloroform-d) δ 18.32 (s, 44%), 18.30 (s, 56%).

^{1}H NMR (400 MHz, Chloroform-d) δ 7.20 – 7.12 (m, 4H, 4xArH), 7.10 – 7.00 (m, 4H, 4xArH), 6.97 (dd, J = 15.0, 6.7 Hz, 1H, NH), 6.84 – 6.62 (m, 1H, NH), 5.17 (dd, J = 98.0, 8.2 Hz, 1H, NH), 4.80 – 4.66 (m, 1H, CHP), 4.44 – 4.36 (m, 1H, CH), 3.95 (dd, J = 8.1, 5.6 Hz, 1H, CH), 2.47 – 2.40 (m, 6H, 2xSCH$_3$), 2.40 – 2.30 (m, 1H, CH), 2.30 – 2.02 (m, 2H, 2xCH), 1.40 (d, J = 5.7 Hz, 9H, Boc), 1.11 – 0.73 (m, 18H, 6xCH$_3$).

HRMS: $C_{33}H_{50}N_3O_7PS_2$ [M+Na]$^+$ calc. 718.2725 found 718.2703

HPLC: purity >99%; retention time 18.963 and 19.170 min

Chromatogram

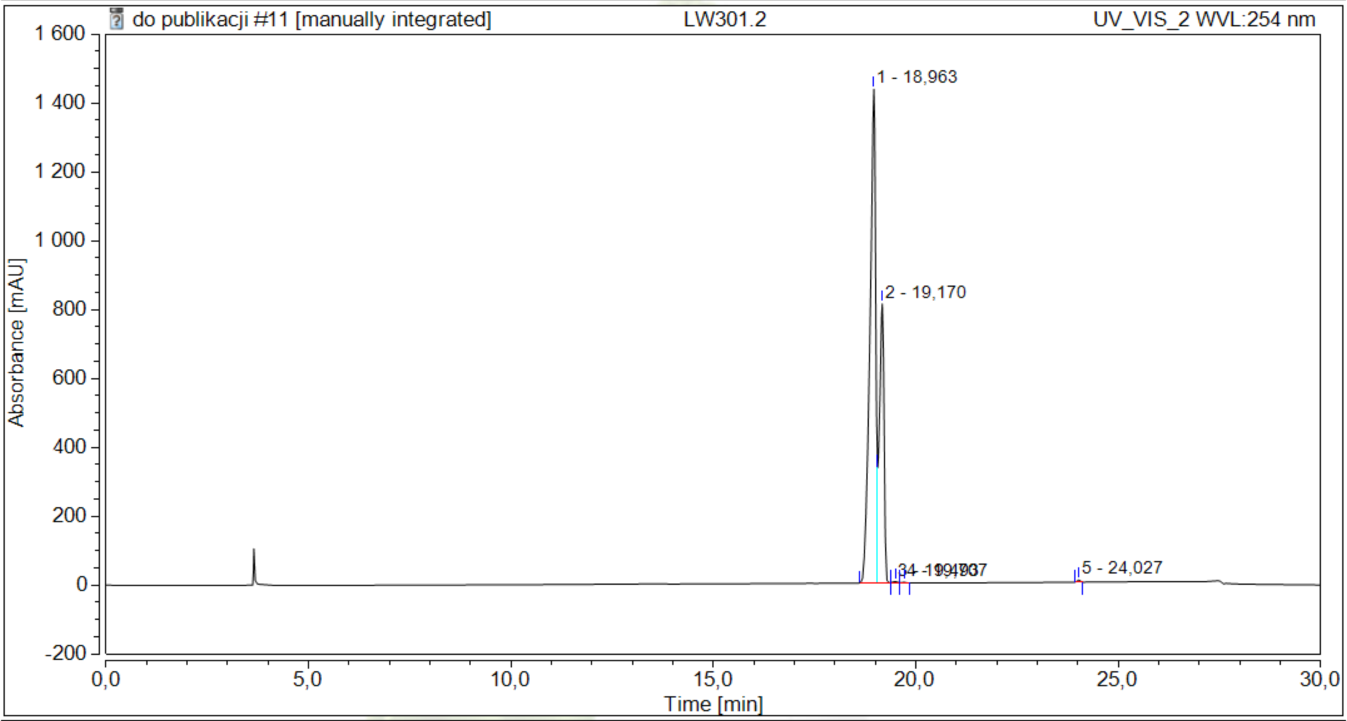

Integration Results

No.	Peak Name	Retention Time min	Area mAU*min	Height mAU	Relative Area %	Relative Height %	Amount n.a.
1		18,963	238,203	1432,966	69,31	63,57	n.a.
2		19,170	104,330	809,486	30,36	35,91	n.a.
3		19,493	0,367	3,179	0,11	0,14	n.a.
4		19,707	0,174	1,542	0,05	0,07	n.a.
5		24,027	0,585	7,043	0,17	0,31	n.a.
**Total:**			**343,659**	**2254,215**	**100,00**	**100,00**	

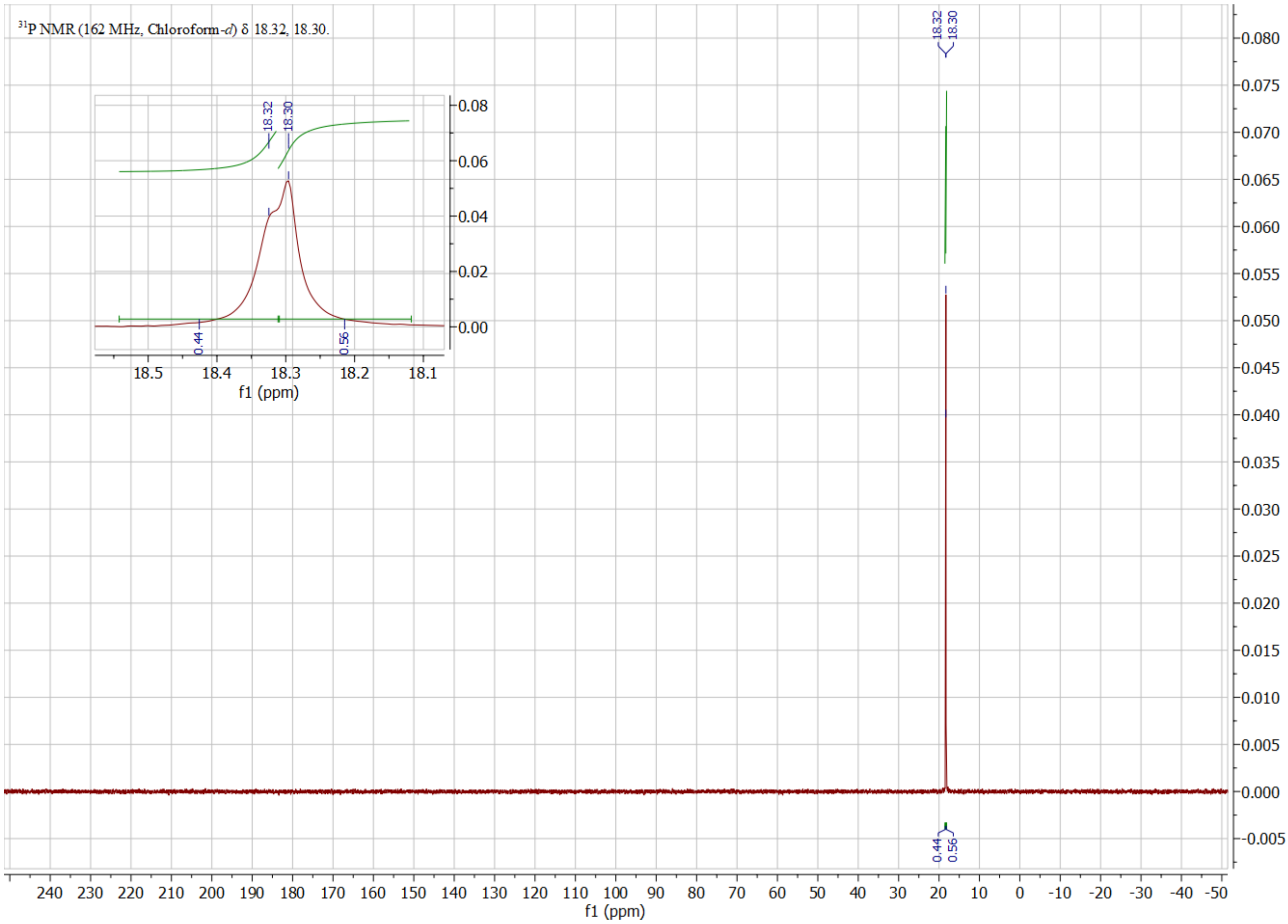

31P NMR (162 MHz, Chloroform-*d*) δ 18.32, 18.30.
f1 (ppm)

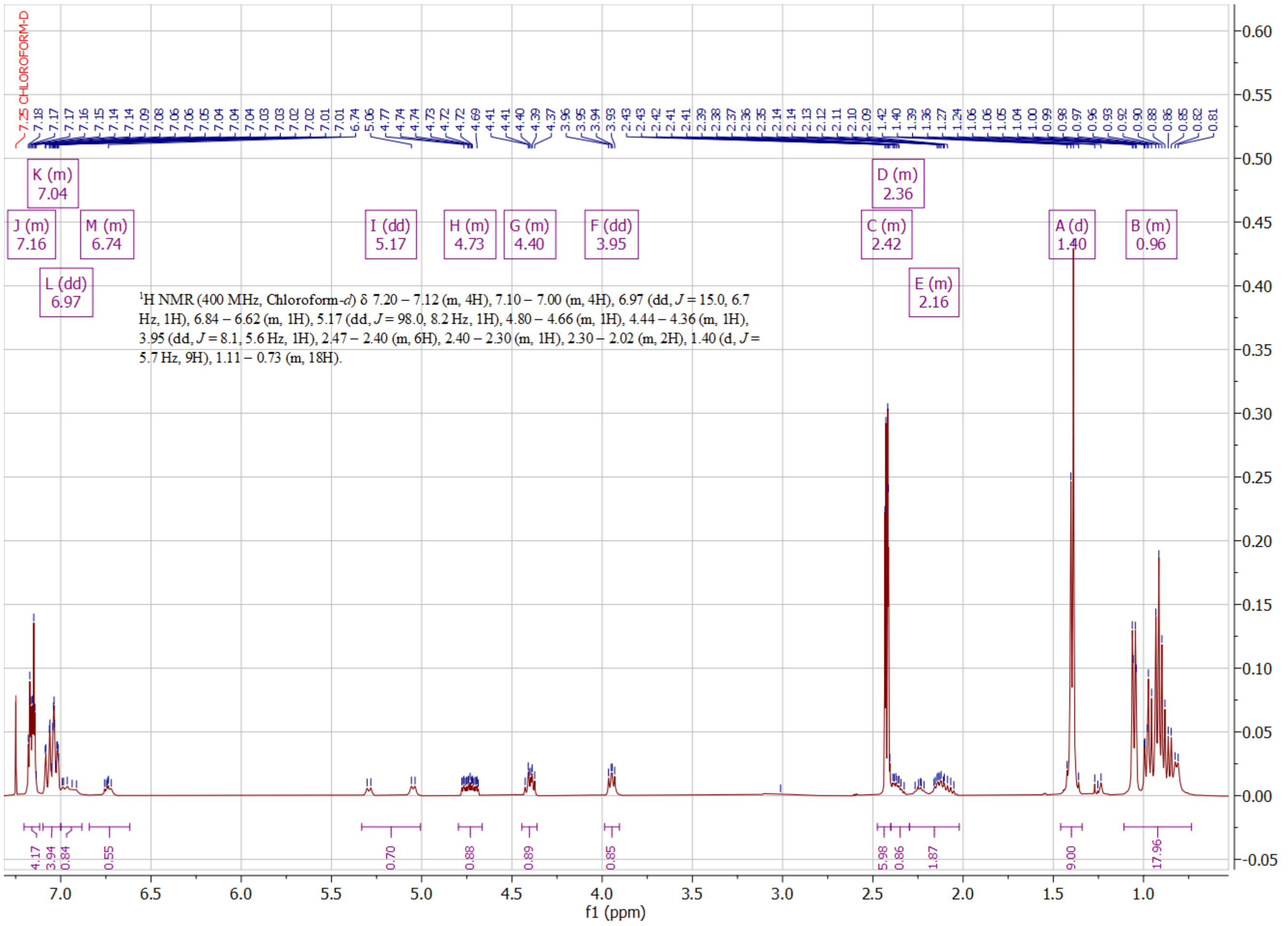

1H NMR (400 MHz, Chloroform-*d*) δ 7.20 – 7.12 (m, 4H), 7.10 – 7.00 (m, 4H), 6.97 (dd, *J* = 15.0, 6.7 Hz, 1H), 6.84 – 6.62 (m, 1H), 5.17 (dd, *J* = 98.0, 8.2 Hz, 1H), 4.80 – 4.66 (m, 1H), 4.44 – 4.36 (m, 1H), 3.95 (dd, *J* = 8.1, 5.6 Hz, 1H), 2.47 – 2.40 (m, 6H), 2.40 – 2.30 (m, 1H), 2.30 – 2.02 (m, 2H), 1.40 (d, *J* = 5.7 Hz, 9H), 1.11 – 0.73 (m, 18H).
f1 (ppm)

(12) Boc-L-Val-L-Tle- Val^{P}(OPh-4-SCH$_3$)$_2$

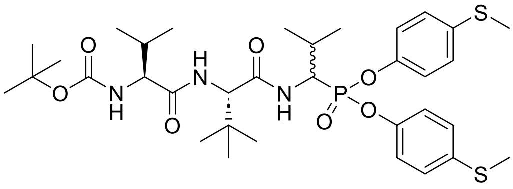

Name: *tert*-butyl ((2S)-1-(((2S)-1-((1-(bis(4-(methylthio)phenoxy)phosphoryl)-2-methylpropyl)amino)-3,3-dimethyl-1-oxobutan-2-yl)amino)-3-methyl-1-oxobutan-2-yl)carbamate

^{31}P NMR (162 MHz, Chloroform-d) δ 18.54 (s, 61%), 18.51 (s, 39%).

^{1}H NMR (400 MHz, Chloroform-d) δ 7.22 – 6.90 (m, 9H, 8xArH, NH), 6.62 (d, J = 9.3 Hz, 1H, NH), 5.19 (dd, J = 133.0, 8.3 Hz, 1H, NH), 4.84 – 4.68 (m, 1H, CHP), 4.43 (dd, J = 69.9, 9.0 Hz, 1H, CH), 3.89 – 3.79 (m, 1H, CH), 2.45 – 2.40 (m, 6H, 2xSCH$_3$), 2.40 – 2.29 (m, 1H, CH), 2.16 – 2.00 (m, 1H, CH), 1.40 (d, J = 7.1 Hz, 9H, Boc), 1.13 – 0.97 (m, 15H, 5xCH$_3$), 0.92 – 0.78 (m, 6H, 2xCH$_3$).

HRMS: $C_{34}H_{52}N_3O_7PS_2$ [M+Na]$^+$ calc. 732.2882 found 732.2848

HPLC: purity >98%; retention time 19.503 and 19.613 min

Chromatogram

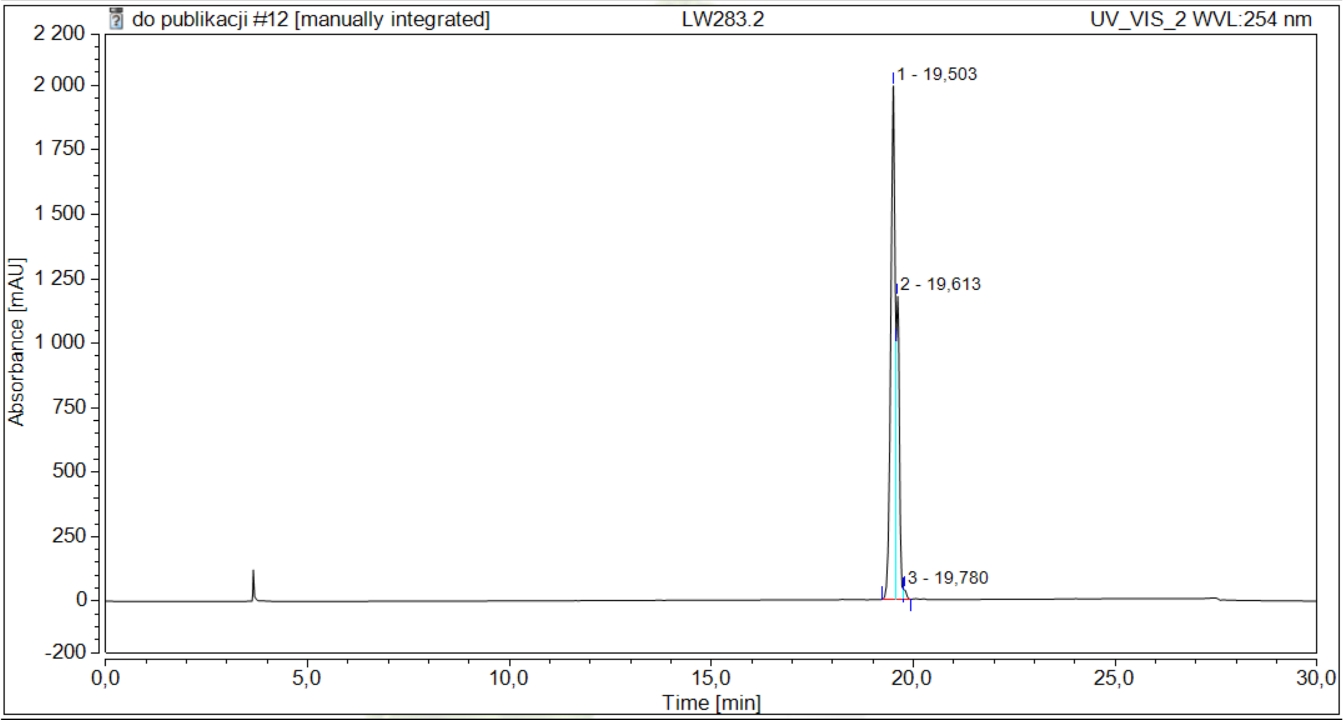

Integration Results

No.	Peak Name	Retention Time min	Area mAU*min	Height mAU	Relative Area %	Relative Height %	Amount n.a.
1		19,503	247,188	1990,786	70,12	62,14	n.a.
2		19,613	101,740	1174,462	28,86	36,66	n.a.
3		19,780	3,586	38,431	1,02	1,20	n.a.
**Total:**			**352,515**	**3203,679**	**100,00**	**100,00**	

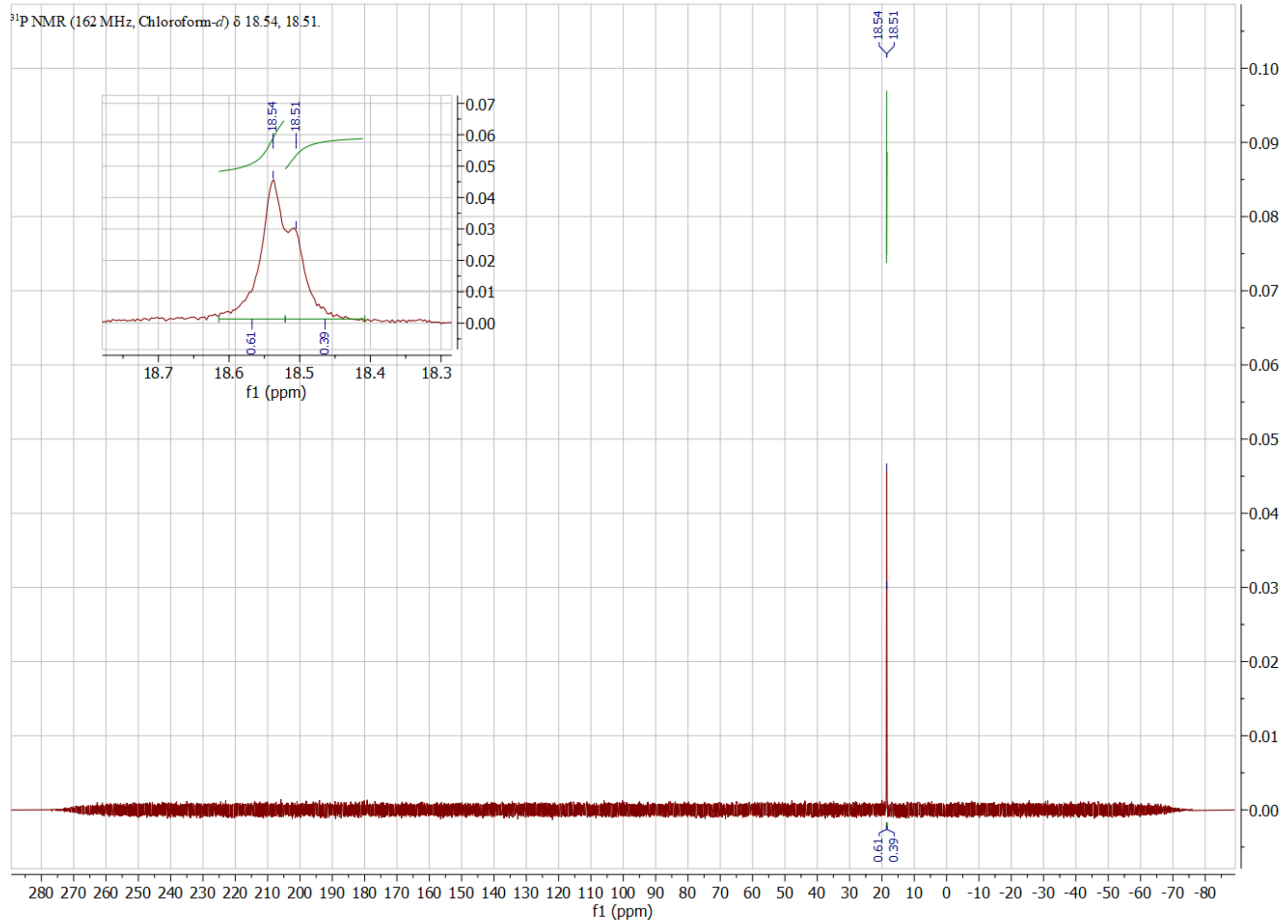
31P NMR (162 MHz, Chloroform-d) δ 18.54, 18.51.
f1 (ppm)

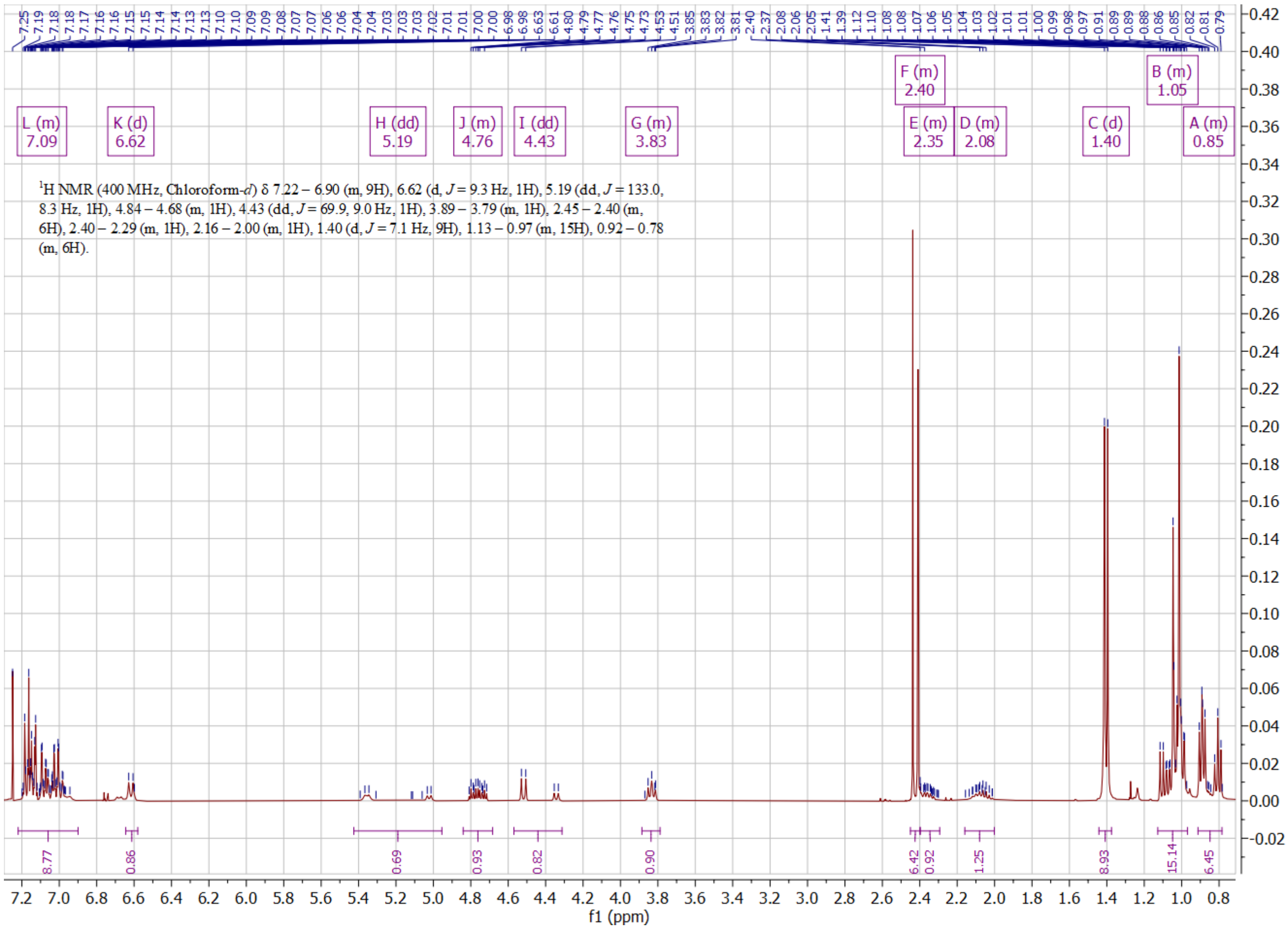
1H NMR (400 MHz, Chloroform-d) δ 7.22 – 6.90 (m, 9H), 6.62 (d, J = 9.3 Hz, 1H), 5.19 (dd, J = 133.0, 8.3 Hz, 1H), 4.84 – 4.68 (m, 1H), 4.43 (dd, J = 69.9, 9.0 Hz, 1H), 3.89 – 3.79 (m, 1H), 2.45 – 2.40 (m, 6H), 2.40 – 2.29 (m, 1H), 2.16 – 2.00 (m, 1H), 1.40 (d, J = 7.1 Hz, 9H), 1.13 – 0.97 (m, 15H), 0.92 – 0.78 (m, 6H).
L (m) 7.09
K (d) 6.62
H (dd) 5.19
J (m) 4.76
I (dd) 4.43
G (m) 3.83
F (m) 2.40
E (m) 2.35
D (m) 2.08
C (d) 1.40
B (m) 1.05
A (m) 0.85
f1 (ppm)

(13) Boc-L-Val-D-Tle- $Val^P(OPh\text{-}4\text{-}SCH_3)_2$

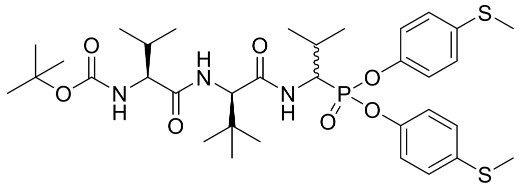

Name: *tert*-butyl ((2S)-1-(((2R)-1-((1-(bis(4-(methylthio)phenoxy)phosphoryl)-2-methylpropyl)amino)-3,3-dimethyl-1-oxobutan-2-yl)amino)-3-methyl-1-oxobutan-2-yl)carbamate
^{31}P NMR (162 MHz, Chloroform-d) δ 18.52 (s, 33%), 18.47 (s, 67%).
^{1}H NMR (400 MHz, Chloroform-d) δ 7.22 – 6.55 (m, 10H, 8xArH, 2xNH), 5.13 (dd, J = 145.8, 6.2 Hz, 1H, NH), 4.81 – 4.70 (m, 1H, CHP), 4.44 (dd, J = 73.2, 9.3 Hz, 1H, CH), 4.00 – 3.87 (m, 1H, CH), 2.47 – 2.41 (m, 6H, $2xSCH_3$), 2.41 – 2.29 (m, 1H, CH), 2.27 – 2.11 (m, 1H, CH), 1.40 (d, J = 6.9 Hz, 9H, Boc), 1.09 – 0.70 (m, 21H, $7xCH_3$).
HRMS: $C_{34}H_{52}N_3O_7PS_2$ $[M+Na]^+$ calc. 732.2882 found 732.2878
HPLC: purity >99%; retention time 19.653 and 19.787 min

Chromatogram

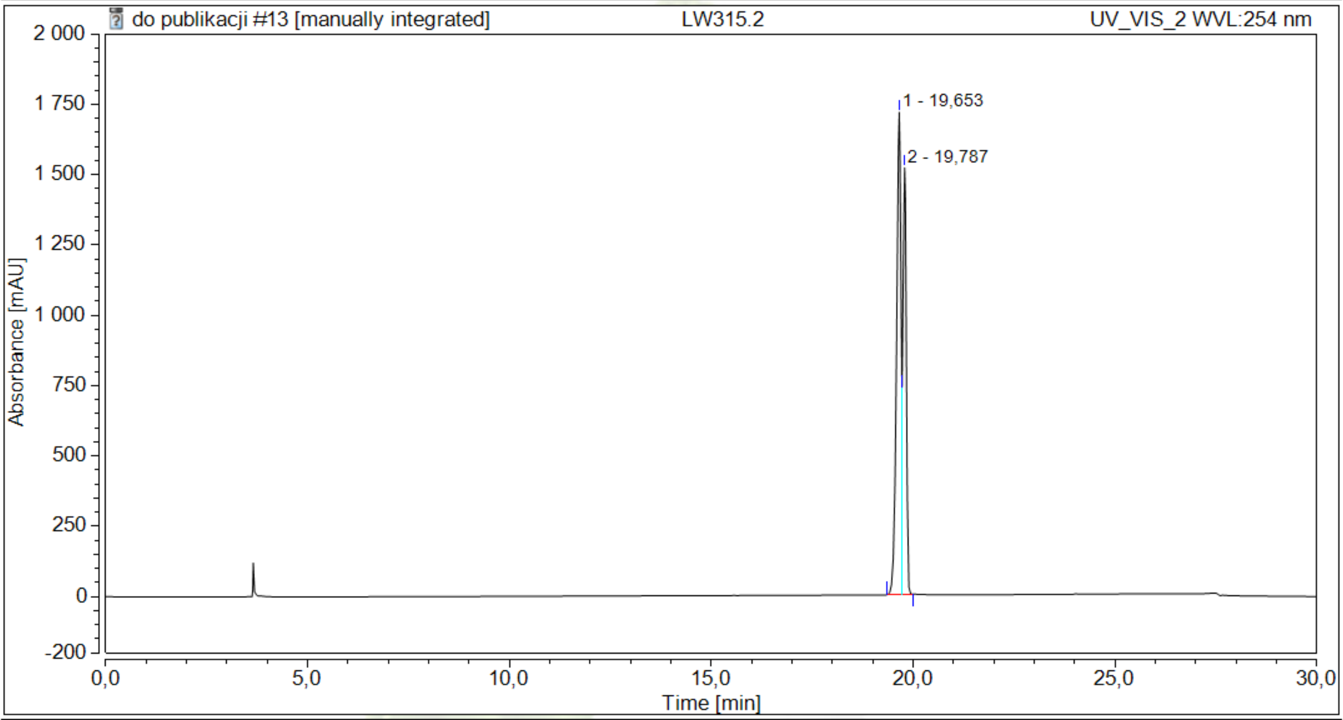

Integration Results

No.	Peak Name	Retention Time min	Area mAU*min	Height mAU	Relative Area %	Relative Height %	Amount n.a.
1		19,653	214,250	1713,689	59,04	53,06	n.a.
2		19,787	148,669	1516,088	40,96	46,94	n.a.
**Total:**			**362,919**	**3229,777**	**100,00**	**100,00**	

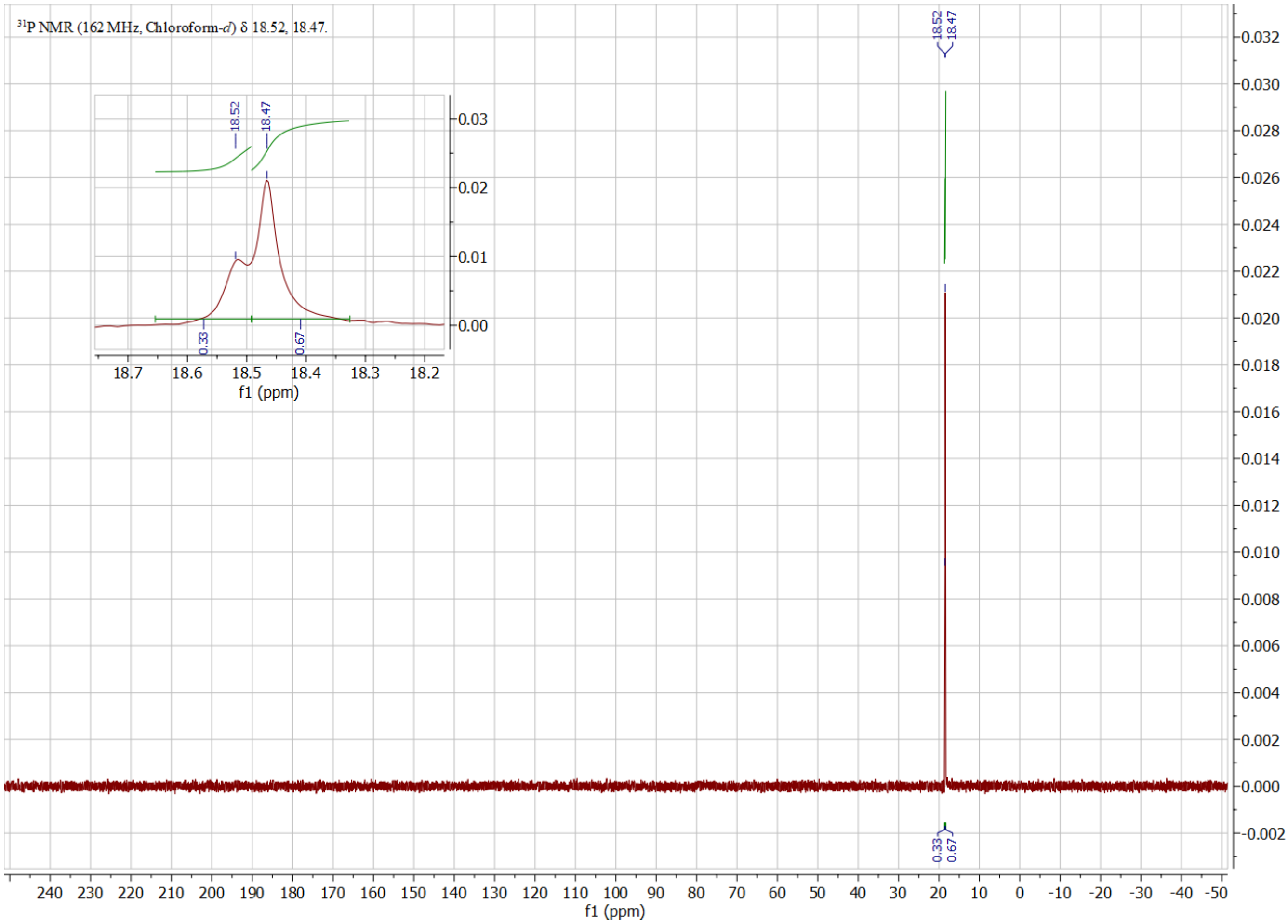
^{31}P NMR (162 MHz, Chloroform-*d*) δ 18.52, 18.47.
f1 (ppm)

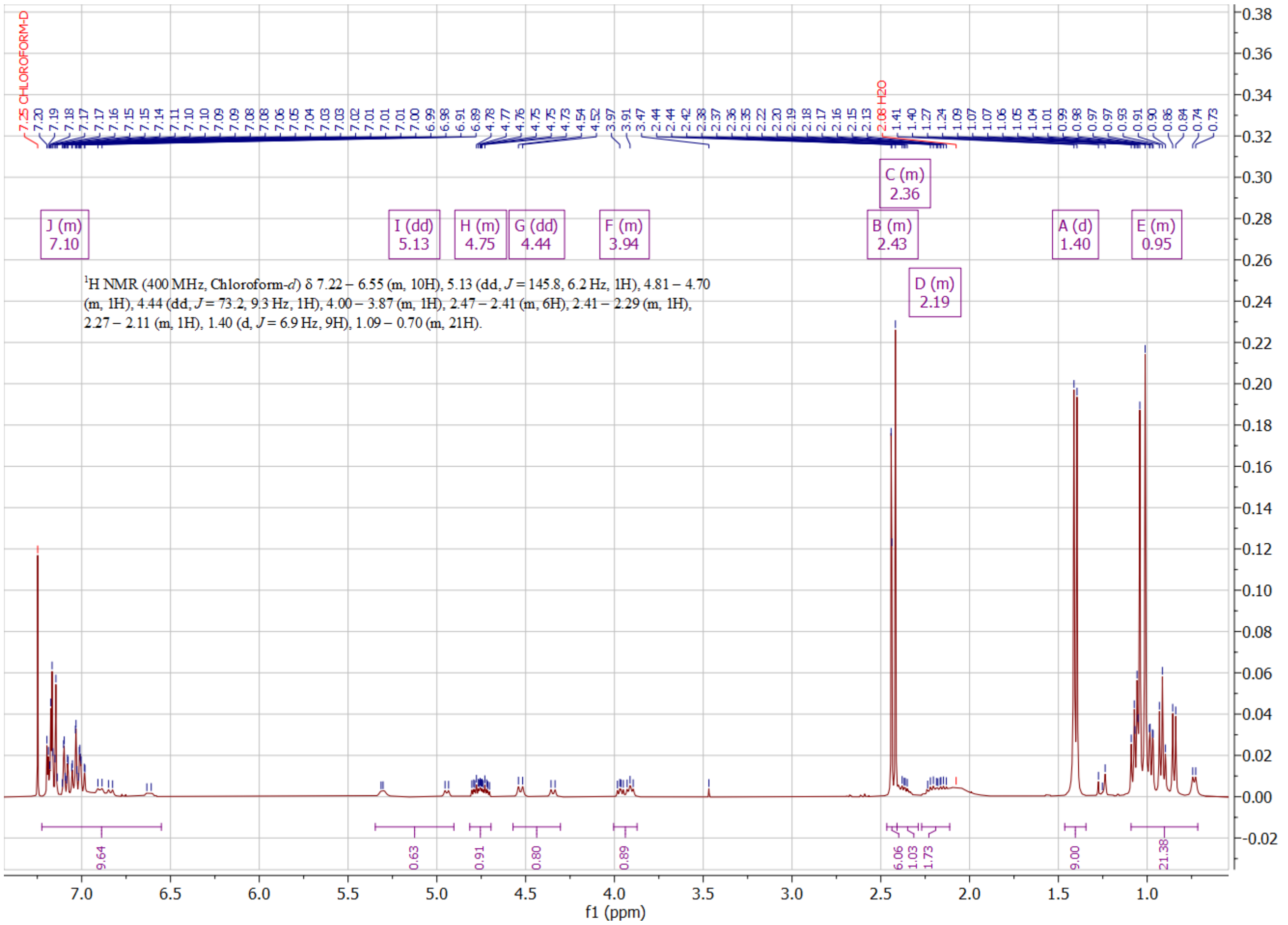
^{1}H NMR (400 MHz, Chloroform-*d*) δ 7.22 – 6.55 (m, 10H), 5.13 (dd, *J* = 145.8, 6.2 Hz, 1H), 4.81 – 4.70 (m, 1H), 4.44 (dd, *J* = 73.2, 9.3 Hz, 1H), 4.00 – 3.87 (m, 1H), 2.47 – 2.41 (m, 6H), 2.41 – 2.29 (m, 1H), 2.27 – 2.11 (m, 1H), 1.40 (d, *J* = 6.9 Hz, 9H), 1.09 – 0.70 (m, 21H).
J (m) 7.10
I (dd) 5.13
H (m) 4.75
G (dd) 4.44
F (m) 3.94
C (m) 2.36
B (m) 2.43
D (m) 2.19
A (d) 1.40
E (m) 0.95
f1 (ppm)

(14) Boc-L-Val-L-Nle- Val^{P}(OPh-4-SCH$_3$)$_2$

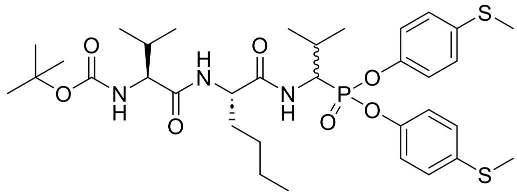

Name: *tert*-butyl ((2S)-1-(((2S)-1-((1-(bis(4-(methylthio)phenoxy)phosphoryl)-2-methylpropyl)amino)-1-oxohexan-2-yl)amino)-3-methyl-1-oxobutan-2-yl)carbamate
^{31}P NMR (162 MHz, Chloroform-d) δ 18.17 (s, 43%), 18.15 (s, 57%).
^{1}H NMR (400 MHz, Chloroform-d) δ 7.20 – 7.13 (m, 4H, 4xArH), 7.12 – 7.01 (m, 4H, 4xArH), 6.91 (t, J = 10.0 Hz, 1H, NH), 6.55 (dd, J = 26.1, 7.7 Hz, 1H, NH), 5.07 (dd, J = 46.3, 8.3 Hz, 1H, NH), 4.77 – 4.65 (m, 1H, CHP), 4.51 – 4.43 (m, 1H, CH), 3.91 – 3.84 (m, 1H, CH), 2.45 – 2.41 (m, 6H, 2xSCH$_3$), 2.41 – 2.29 (m, 1H, CH), 2.18 – 2.04 (m, 1H, CH), 1.90 – 1.69 (m, 1H, CH$_2$), 1.66 – 1.52 (m, 1H, CH$_2$), 1.41 (d, J = 6.4 Hz, 9H, Boc), 1.32 – 1.21 (m, 4H, 2xCH$_2$), 1.11 – 0.98 (m, 6H, 2xCH$_3$), 0.95 – 0.74 (m, 9H, 3xCH$_3$).
HRMS: $C_{34}H_{52}N_3O_7PS_2$ [M+Na]$^+$ calc. 732.2882 found 732.2873
HPLC: purity >98%; retention time 19.390 and 19.697 min

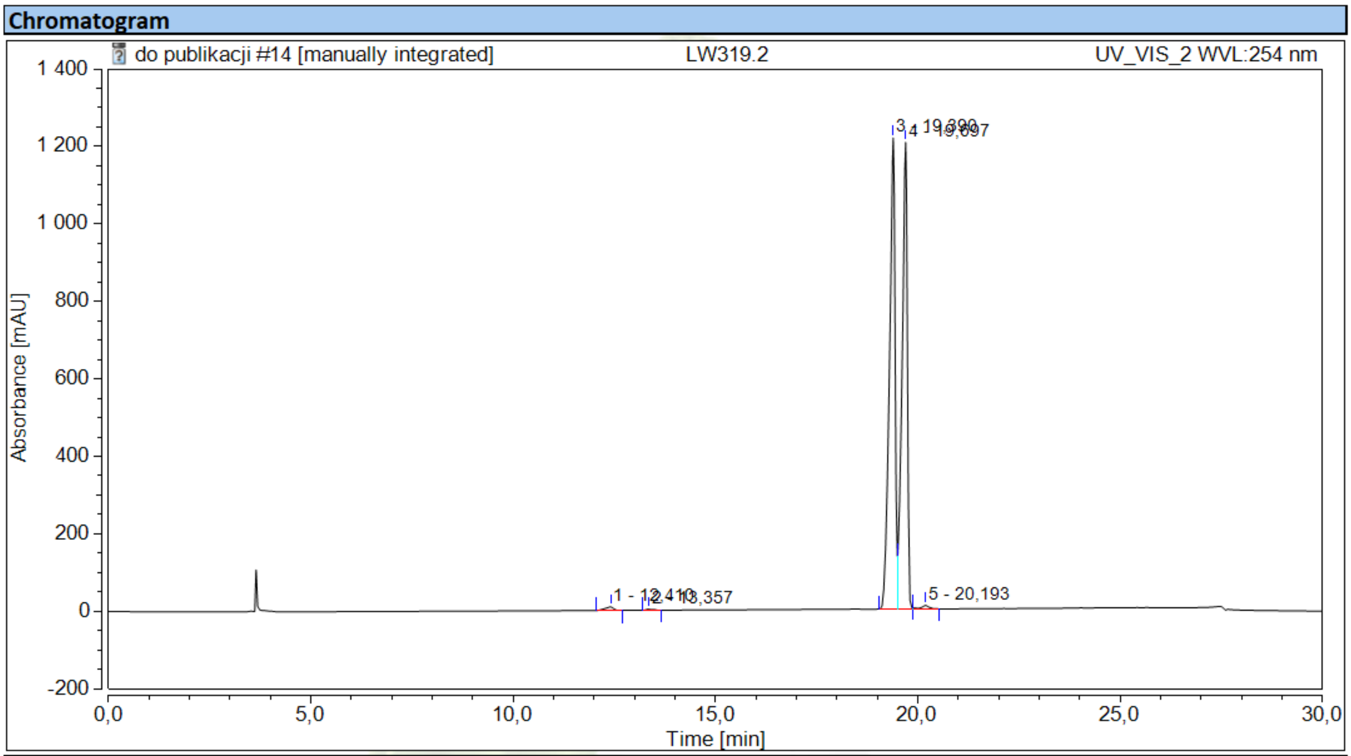

Integration Results

No.	Peak Name	Retention Time min	Area mAU*min	Height mAU	Relative Area %	Relative Height %	Amount n.a.
1		12,410	2,217	9,704	0,58	0,40	n.a.
2		13,357	0,464	2,402	0,12	0,10	n.a.
3		19,390	195,761	1216,305	51,45	49,80	n.a.
4		19,697	179,901	1204,540	47,28	49,32	n.a.
5		20,193	2,173	9,497	0,57	0,39	n.a.
**Total:**			**380,517**	**2442,450**	**100,00**	**100,00**	

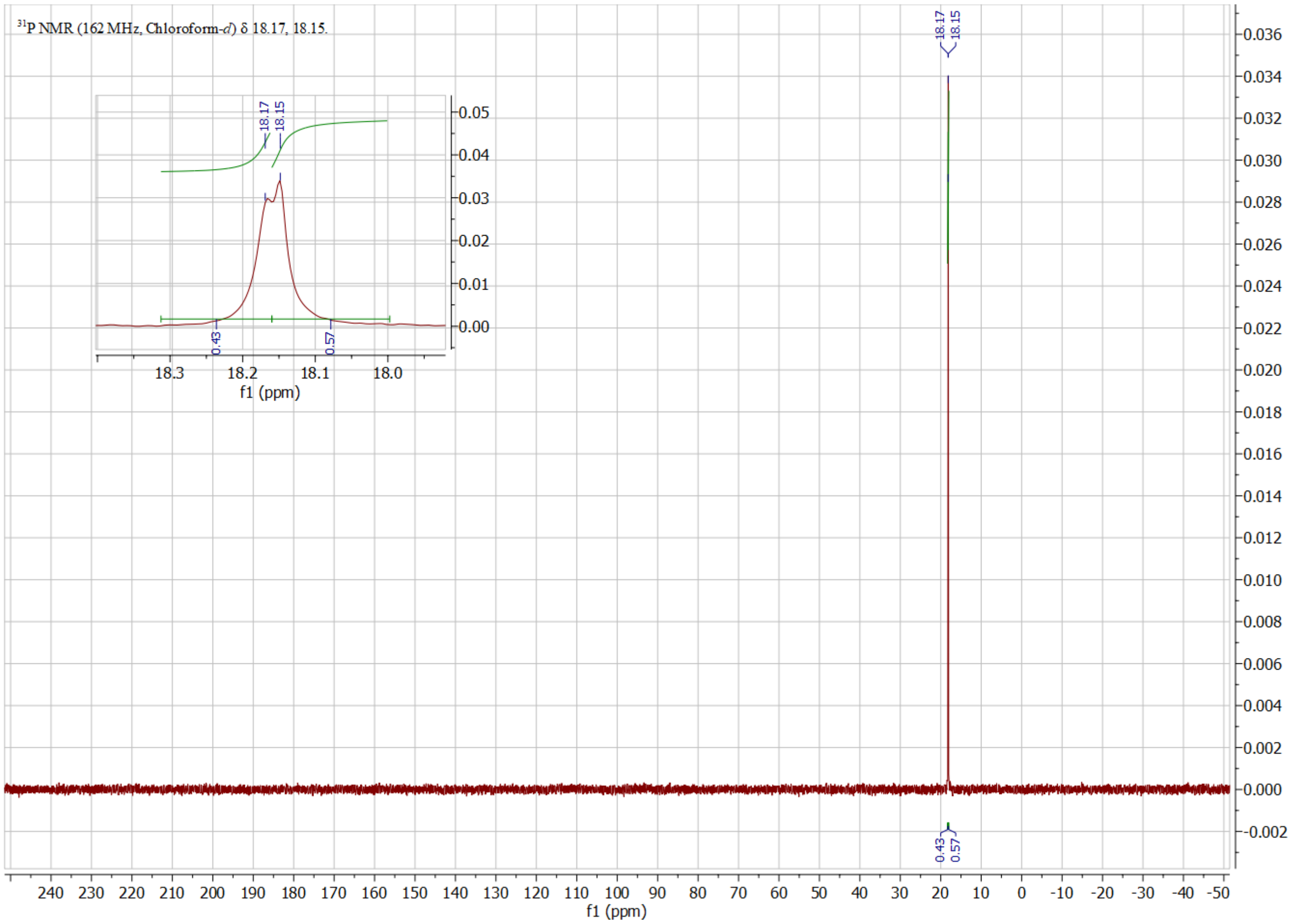
^{31}P NMR (162 MHz, Chloroform-*d*) δ 18.17, 18.15.
18.17
18.15
0.43
0.57
f1 (ppm)

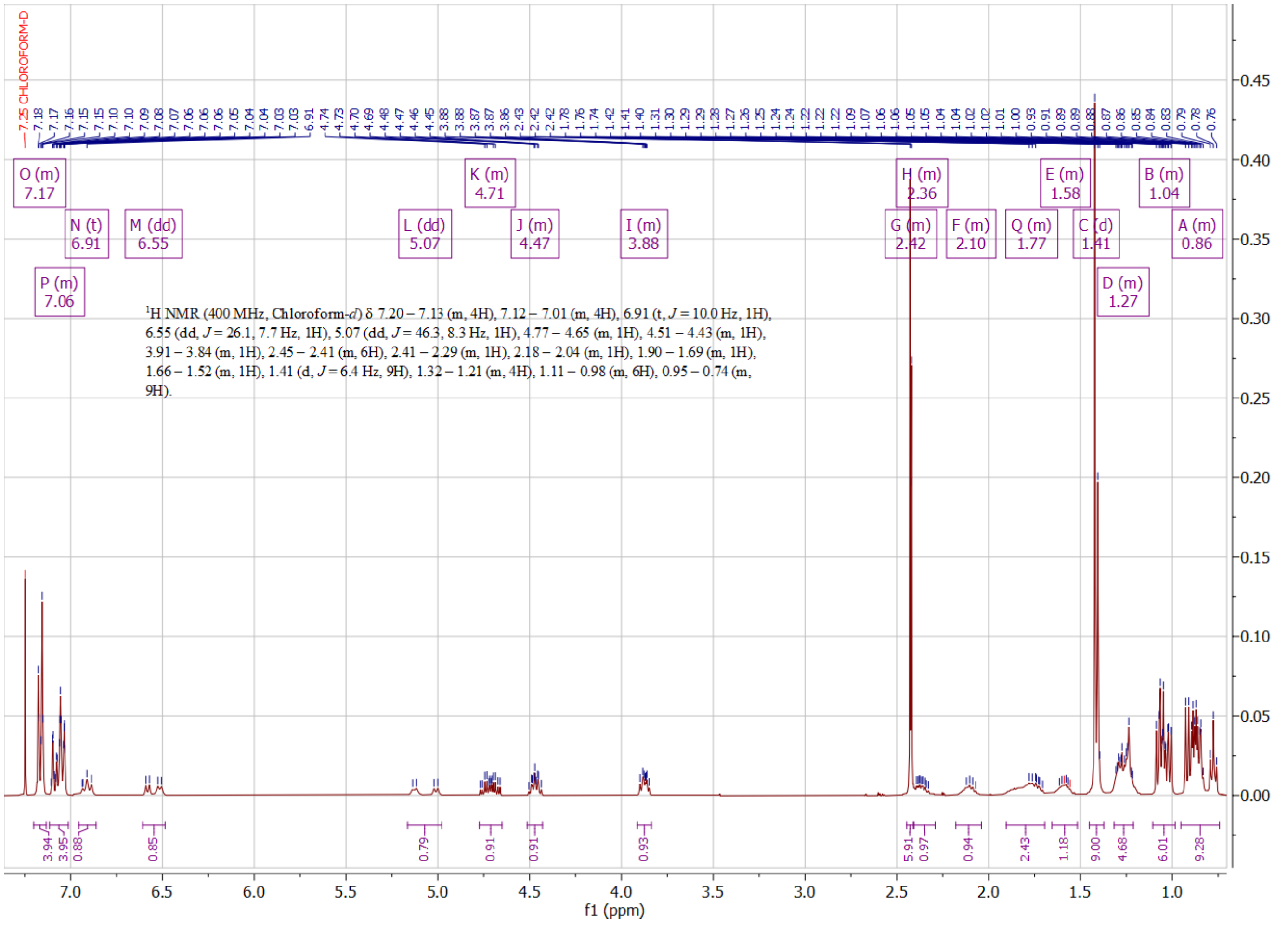
7.25 CHLOROFORM-D
O (m) 7.17
N (t) 6.91
M (dd) 6.55
P (m) 7.06
K (m) 4.71
L (dd) 5.07
J (m) 4.47
I (m) 3.88
H (m) 2.36
G (m) 2.42
F (m) 2.10
Q (m) 1.77
E (m) 1.58
C (d) 1.41
D (m) 1.27
B (m) 1.04
A (m) 0.86
^{1}H NMR (400 MHz, Chloroform-*d*) δ 7.20 – 7.13 (m, 4H), 7.12 – 7.01 (m, 4H), 6.91 (t, *J* = 10.0 Hz, 1H), 6.55 (dd, *J* = 26.1, 7.7 Hz, 1H), 5.07 (dd, *J* = 46.3, 8.3 Hz, 1H), 4.77 – 4.65 (m, 1H), 4.51 – 4.43 (m, 1H), 3.91 – 3.84 (m, 1H), 2.45 – 2.41 (m, 6H), 2.41 – 2.29 (m, 1H), 2.18 – 2.04 (m, 1H), 1.90 – 1.69 (m, 1H), 1.66 – 1.52 (m, 1H), 1.41 (d, *J* = 6.4 Hz, 9H), 1.32 – 1.21 (m, 4H), 1.11 – 0.98 (m, 6H), 0.95 – 0.74 (m, 9H).
f1 (ppm)

(15) Boc-L-Val-D-Nle- Val^P(OPh-4-SCH$_3$)$_2$

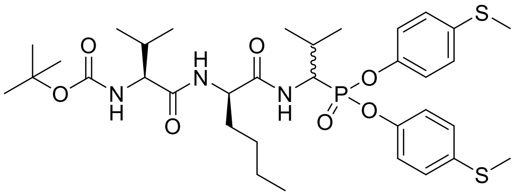

Name: *tert*-butyl ((2S)-1-(((2R)-1-((1-(bis(4-(methylthio)phenoxy)phosphoryl)-2-methylpropyl)amino)-1-oxohexan-2-yl)amino)-3-methyl-1-oxobutan-2-yl)carbamate

^{31}P NMR (162 MHz, Chloroform-d) δ 18.31 (s, 55%), 18.08 (s, 45%).

^{1}H NMR (400 MHz, Chloroform-d) δ 7.22 – 7.13 (m, 4H), 7.13 – 6.92 (m, 5H), 6.59 (dd, J = 56.3, 5.8 Hz, 1H), 5.08 (dd, J = 50.6, 7.1 Hz, 1H), 4.77 – 4.65 (m, 1H), 4.51 – 4.41 (m, 1H), 3.95 – 3.87 (m, 1H), 2.43 (dd, J = 1.8, 1.2 Hz, 6H), 2.41 – 2.24 (m, 1H), 2.18 – 2.05 (m, 1H), 1.93 – 1.66 (m, 1H), 1.64 – 1.51 (m, 1H), 1.40 (d, J = 6.7 Hz, 9H), 1.31 – 1.20 (m, 4H), 1.10 – 0.97 (m, 6H), 0.96 – 0.74 (m, 9H).

HRMS: $C_{34}H_{52}N_3O_7PS_2$ [M+Na]+ calc. 732.2882 found 732.2864

HPLC: purity >98%; retention time 19.573 and 19.813 min

Chromatogram

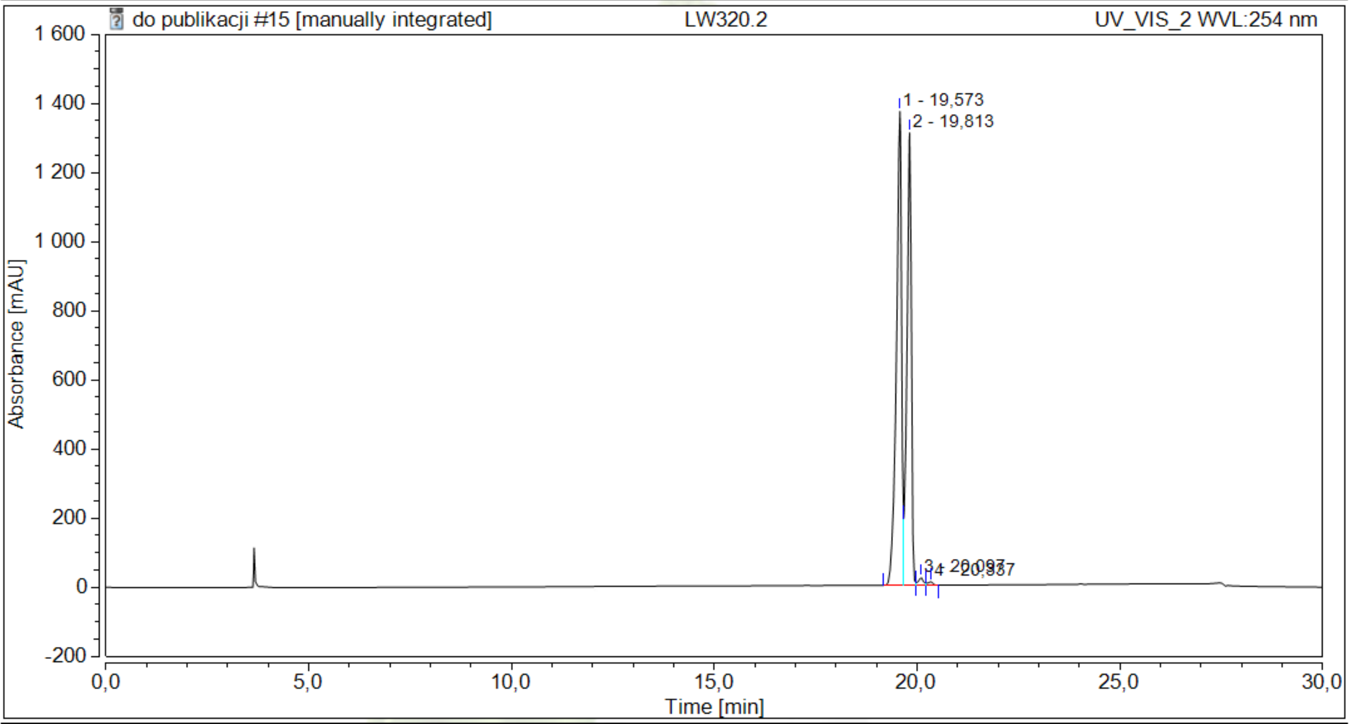

Integration Results

No.	Peak Name	Retention Time min	Area mAU*min	Height mAU	Relative Area %	Relative Height %	Amount n.a.
1		19,573	201,084	1370,770	54,50	50,55	n.a.
2		19,813	163,435	1308,748	44,29	48,26	n.a.
3		20,097	3,071	22,062	0,83	0,81	n.a.
4		20,337	1,392	10,322	0,38	0,38	n.a.
**Total:**			**368,981**	**2711,902**	**100,00**	**100,00**	

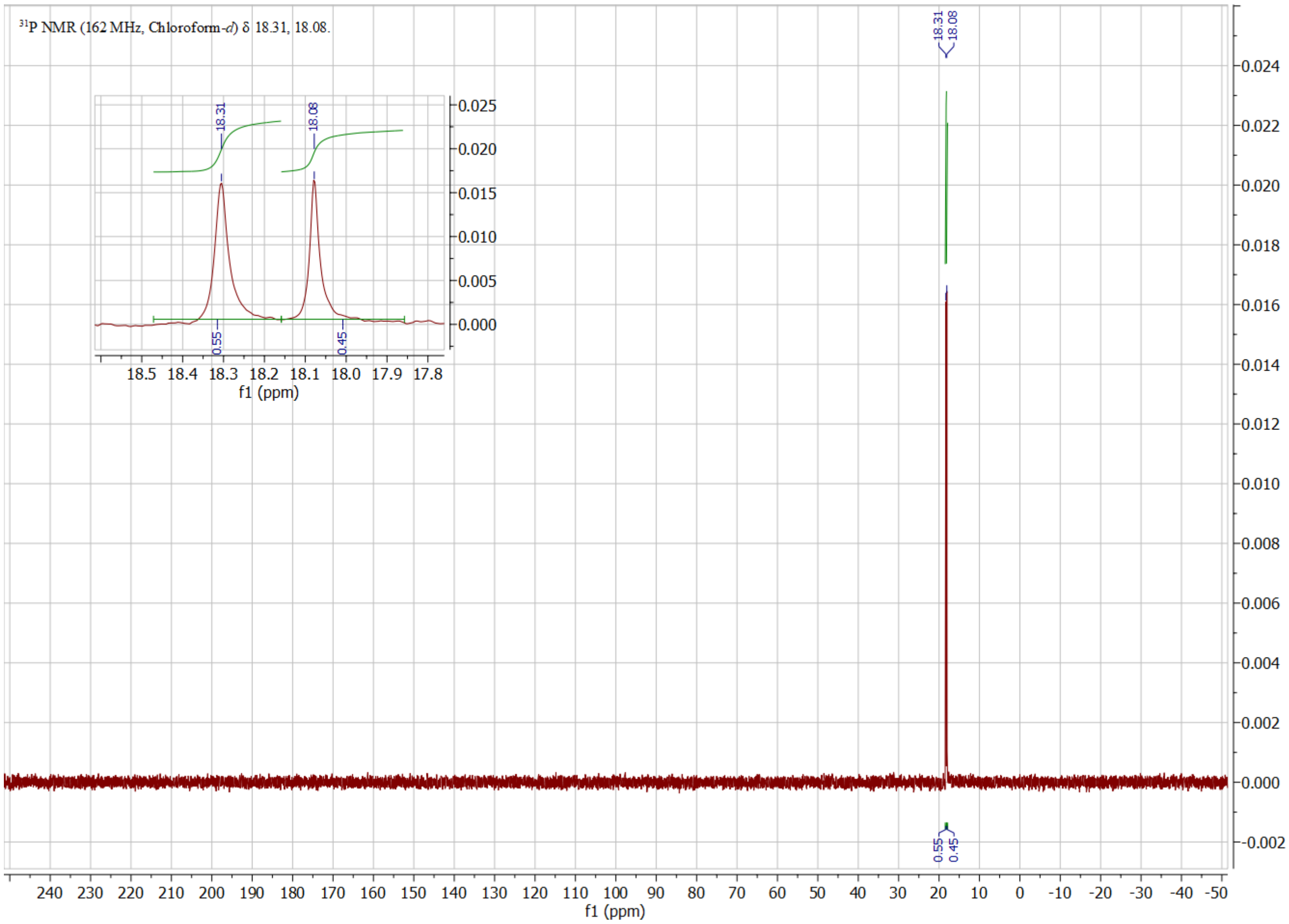
^{31}P NMR (162 MHz, Chloroform-*d*) δ 18.31, 18.08.
f1 (ppm)

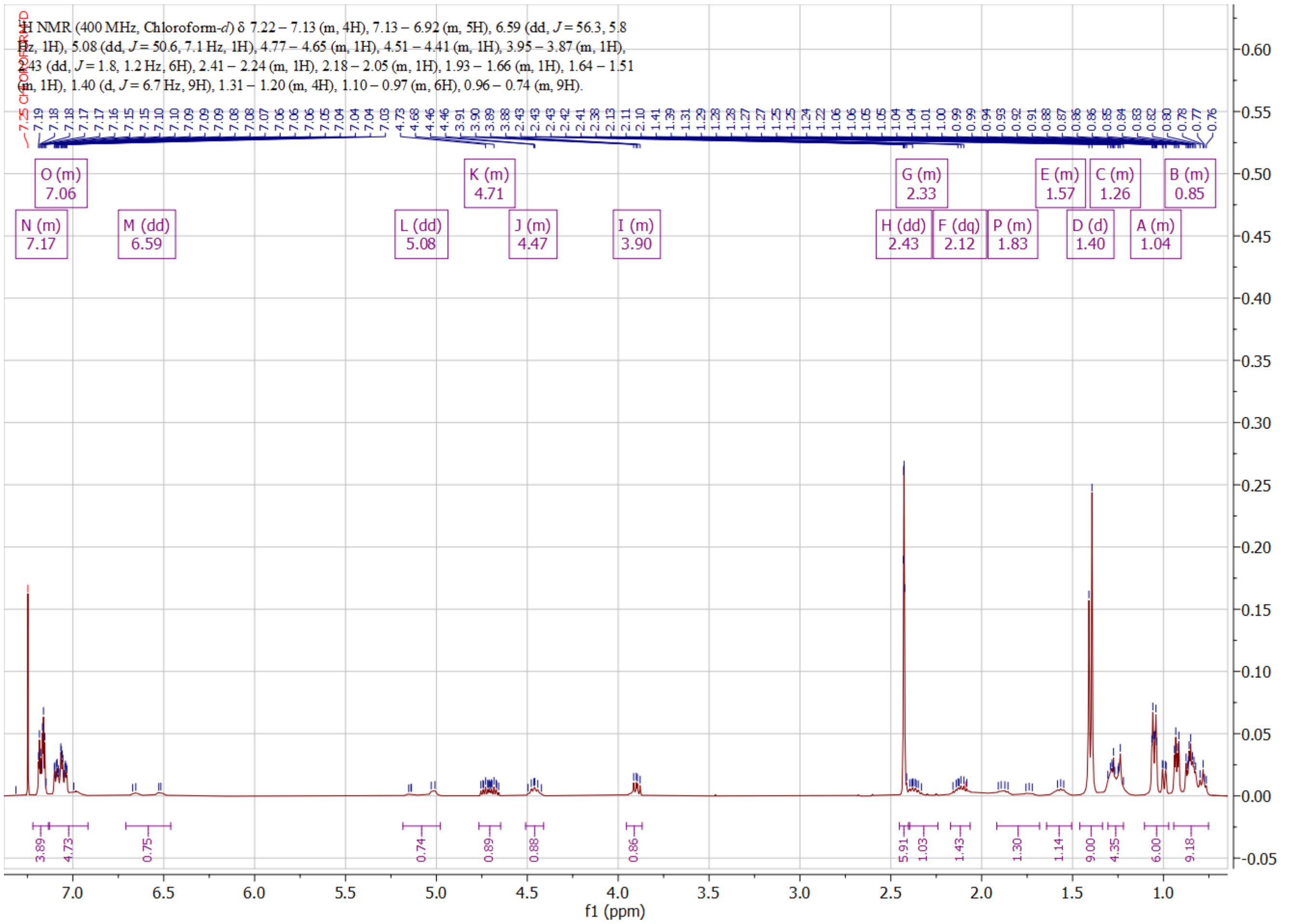
^{1}H NMR (400 MHz, Chloroform-*d*) δ 7.22 – 7.13 (m, 4H), 7.13 – 6.92 (m, 5H), 6.59 (dd, *J* = 56.3, 5.8 Hz, 1H), 5.08 (dd, *J* = 50.6, 7.1 Hz, 1H), 4.77 – 4.65 (m, 1H), 4.51 – 4.41 (m, 1H), 3.95 – 3.87 (m, 1H), 2.43 (dd, *J* = 1.8, 1.2 Hz, 6H), 2.41 – 2.24 (m, 1H), 2.18 – 2.05 (m, 1H), 1.93 – 1.66 (m, 1H), 1.64 – 1.51 (m, 1H), 1.40 (d, *J* = 6.7 Hz, 9H), 1.31 – 1.20 (m, 4H), 1.10 – 0.97 (m, 6H), 0.96 – 0.74 (m, 9H).
f1 (ppm)

(16) Boc-L-Val-L-Leu-Val^P(OPh-4-SCH$_3$)$_2$

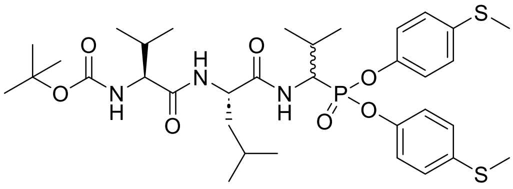

Name: *tert*-butyl ((2S)-1-(((2S)-1-((1-(bis(4-(methylthio)phenoxy)phosphoryl)-2-methylpropyl)amino)-4-methyl-1-oxopentan-2-yl)amino)-3-methyl-1-oxobutan-2-yl)carbamate

^{31}P NMR (162 MHz, Chloroform-d) δ 18.37(s, 59%), 18.14 (s, 41%).

^{1}H NMR (400 MHz, Chloroform-d) δ ^{1}H NMR (400 MHz, CHLOROFORM-*D*) δ 7.21 – 7.14 (m, 4H, 4xArH), 7.12 – 7.00 (m, 4H, 4xArH), 6.88 – 6.78 (m, 1H, NH), 6.46 – 6.38 (m, 1H, NH), 5.00 (dd, *J* = 43.1, 8.3 Hz, 1H, NH), 4.76 – 4.64 (m, 1H, CHP), 4.54 – 4.41 (m, 1H, CH), 3.89 – 3.80 (m, 1H, CH), 2.46 – 2.41 (m, 6H, 2xSCH$_3$), 2.41 – 2.27 (m, 1H, CH), 2.22 – 2.01 (m, 1H, CH), 1.73 – 1.45 (m, 3H, CH$_2$, CH), 1.41 (d, *J* = 6.6 Hz, 9H, Boc), 1.11 – 1.00 (m, 6H, 2xCH$_3$), 0.94 – 0.82 (m, 12H, 4xCH$_3$).

HRMS: $C_{34}H_{52}N_3O_7PS_2$ [M+Na]+ calc. 732.2882 found 732.2874

HPLC: purity >98%; retention time 19.357 and 19.570 min

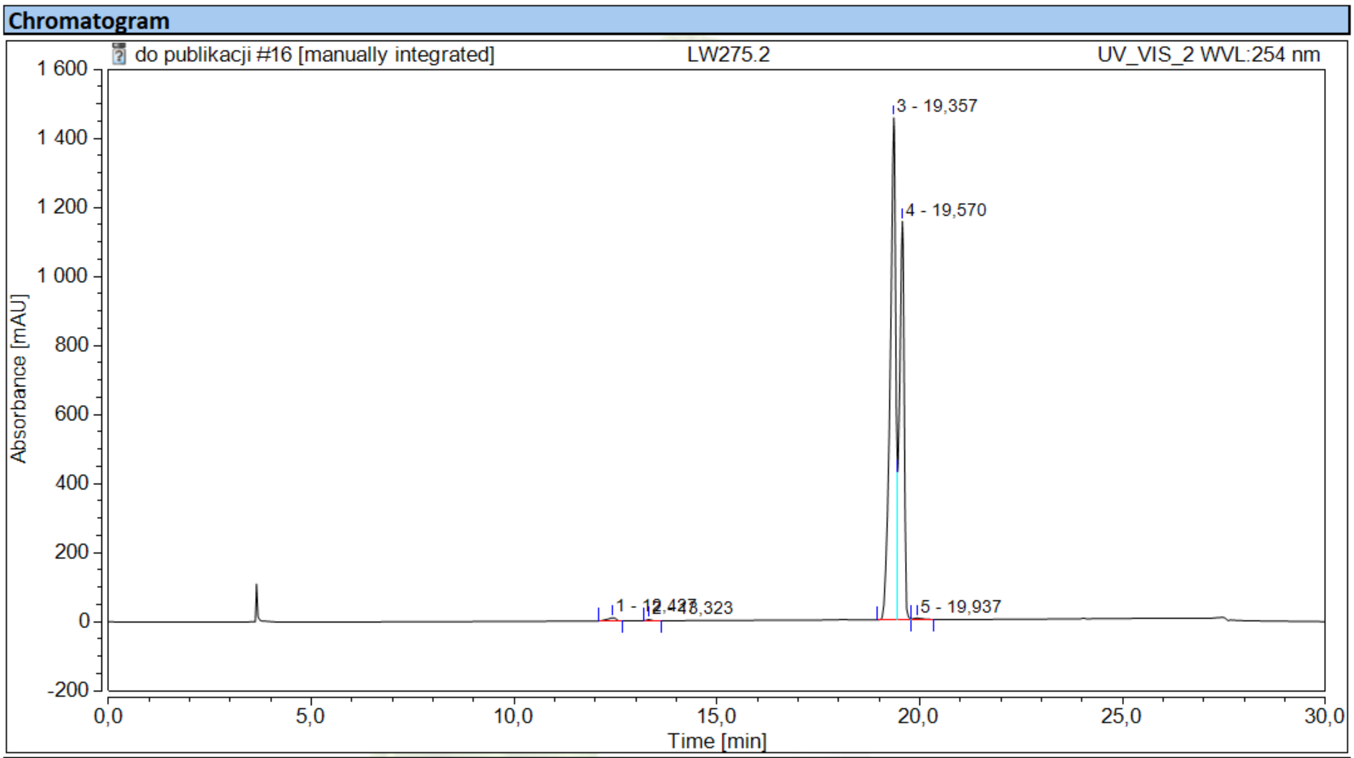

Integration Results

No.	Peak Name	Retention Time min	Area mAU*min	Height mAU	Relative Area %	Relative Height %	Amount n.a.
1		12,427	2,256	9,302	0,58	0,35	n.a.
2		13,323	0,405	3,354	0,10	0,13	n.a.
3		19,357	231,119	1453,677	59,79	55,38	n.a.
4		19,570	151,576	1153,570	39,21	43,95	n.a.
5		19,937	1,205	4,930	0,31	0,19	n.a.
**Total:**			**386,562**	**2624,833**	**100,00**	**100,00**	

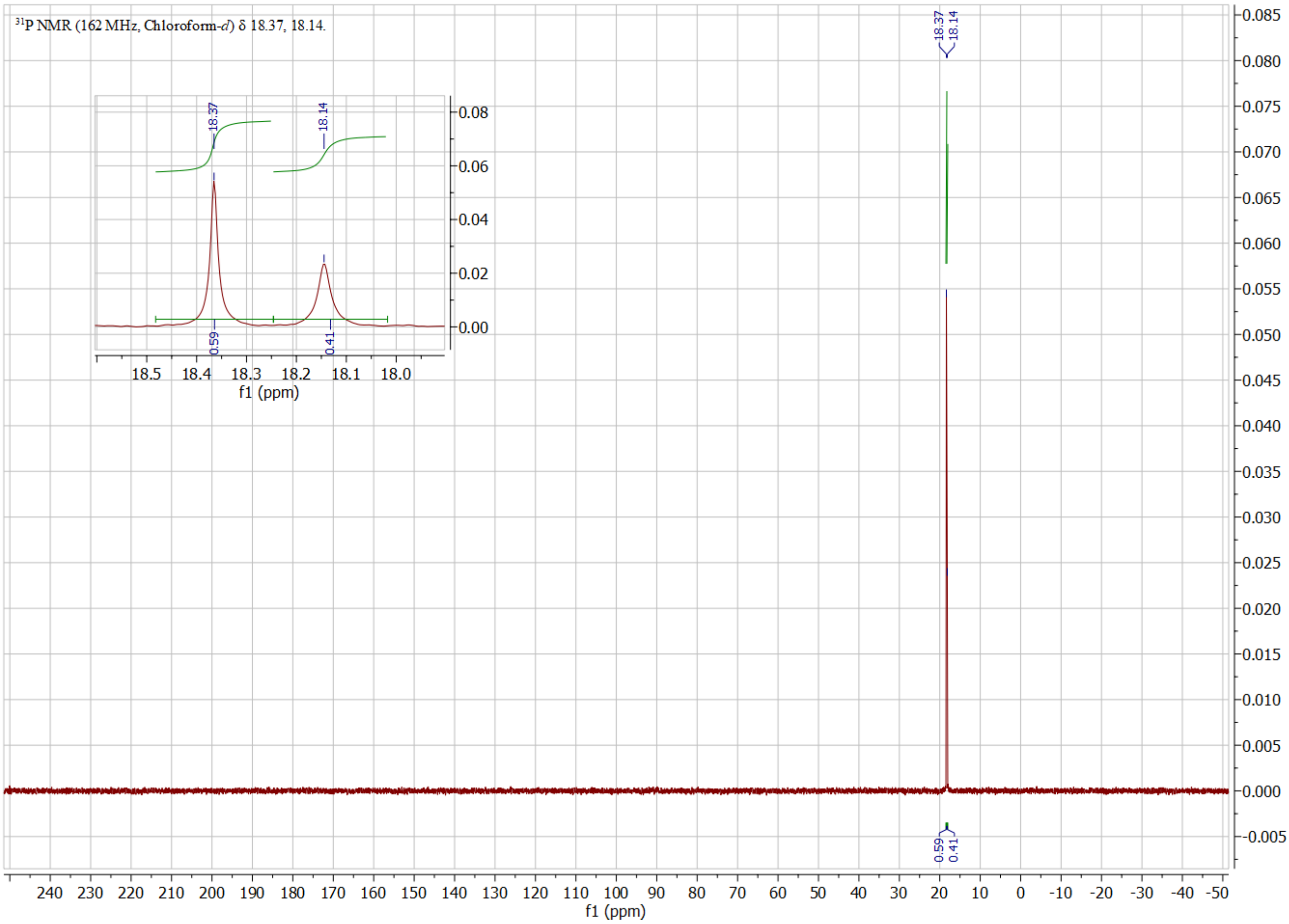
^{31}P NMR (162 MHz, Chloroform-d) δ 18.37, 18.14.
f1 (ppm)

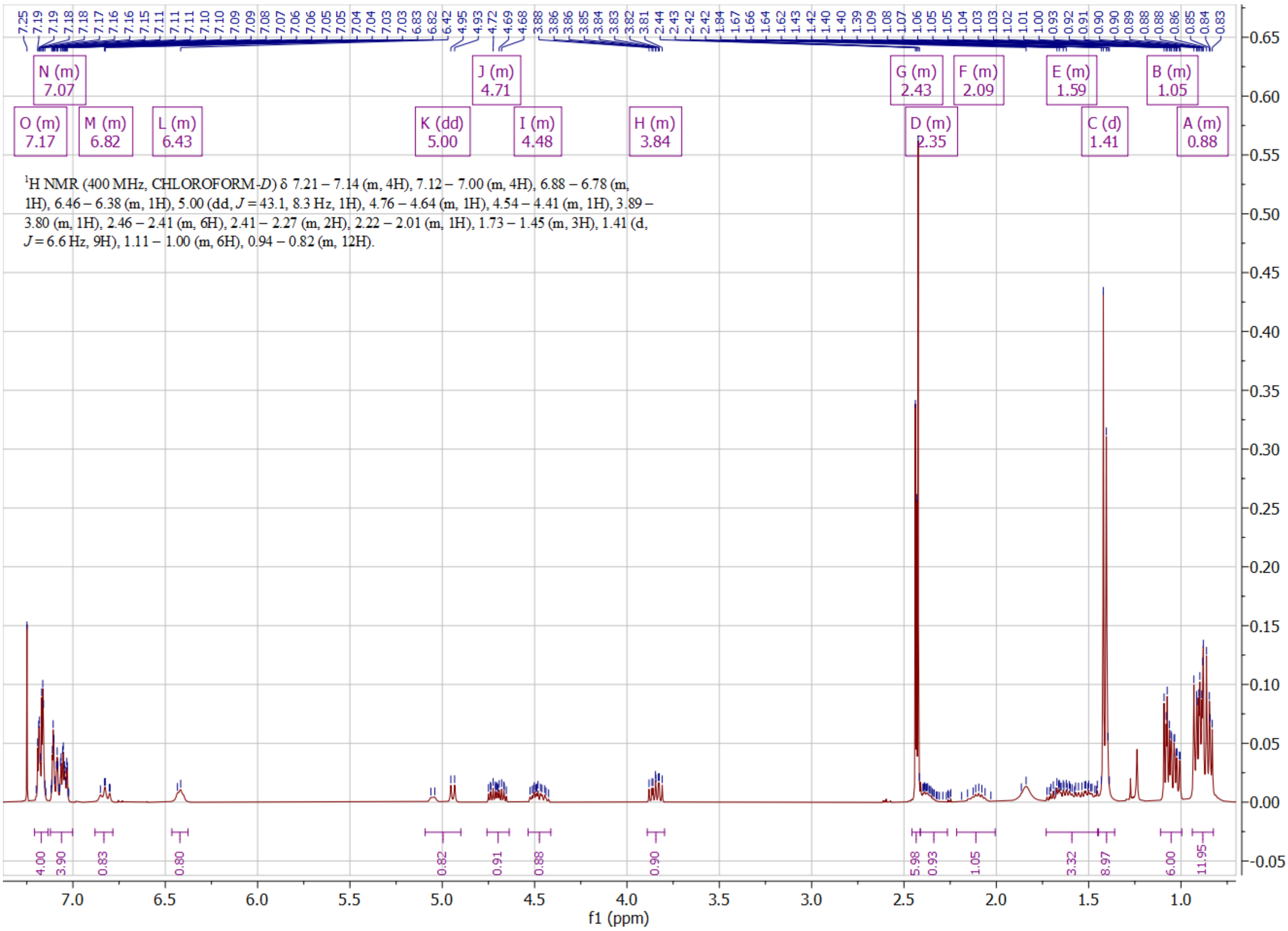
^{1}H NMR (400 MHz, CHLOROFORM-D) δ 7.21 – 7.14 (m, 4H), 7.12 – 7.00 (m, 4H), 6.88 – 6.78 (m, 1H), 6.46 – 6.38 (m, 1H), 5.00 (dd, J = 43.1, 8.3 Hz, 1H), 4.76 – 4.64 (m, 1H), 4.54 – 4.41 (m, 1H), 3.89 – 3.80 (m, 1H), 2.46 – 2.41 (m, 6H), 2.41 – 2.27 (m, 2H), 2.22 – 2.01 (m, 1H), 1.73 – 1.45 (m, 3H), 1.41 (d, J = 6.6 Hz, 9H), 1.11 – 1.00 (m, 6H), 0.94 – 0.82 (m, 12H).
f1 (ppm)

(17) Boc-L-Val-D-Leu-Val^P(OPh-4-SCH$_3$)$_2$

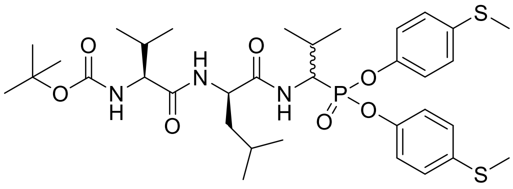

Name: *tert*-butyl ((2S)-1-(((2R)-1-((1-(bis(4-(methylthio)phenoxy)phosphoryl)-2-methylpropyl)amino)-4-methyl-1-oxopentan-2-yl)amino)-3-methyl-1-oxobutan-2-yl)carbamate

^{31}P NMR (162 MHz, Chloroform-d) δ 18.43 (s, 45%), 18.02 (s, 55%).

^{1}H NMR (400 MHz, Chloroform-d) δ 7.23 – 7.14 (m, 4H, 4xArH), 7.14 – 7.03 (m, 4H, 4xArH), 7.03 – 6.73 (m, 1H, NH), 6.54 (dd, *J* = 78.6, 7.8 Hz, 1H, NH), 5.06 (dd, *J* = 54.3, 8.3 Hz, 1H, NH), 4.77 – 4.64 (m, 1H, CHP), 4.55 – 4.41 (m, 1H, CH), 3.94 – 3.85 (m, 1H, CH), 2.47 – 2.41 (m, 6H, , 2xSCH$_3$), 2.41 – 2.30 (m, 1H, CH), 2.18 – 2.05 (m, 1H, CH), 1.74 – 1.45 (m, 3H, CH$_2$, CH), 1.40 (d, *J* = 7.9 Hz, 9H, Boc), 1.11 – 0.96 (m, 6H, 2xCH$_3$), 0.94 – 0.80 (m, 12H, 4xCH$_3$).

HRMS: $C_{34}H_{52}N_3O_7PS_2$ [M+Na]+ calc. 732.2882 found 732.2891

HPLC: purity >99%; retention time 19.500 and 19.703 min

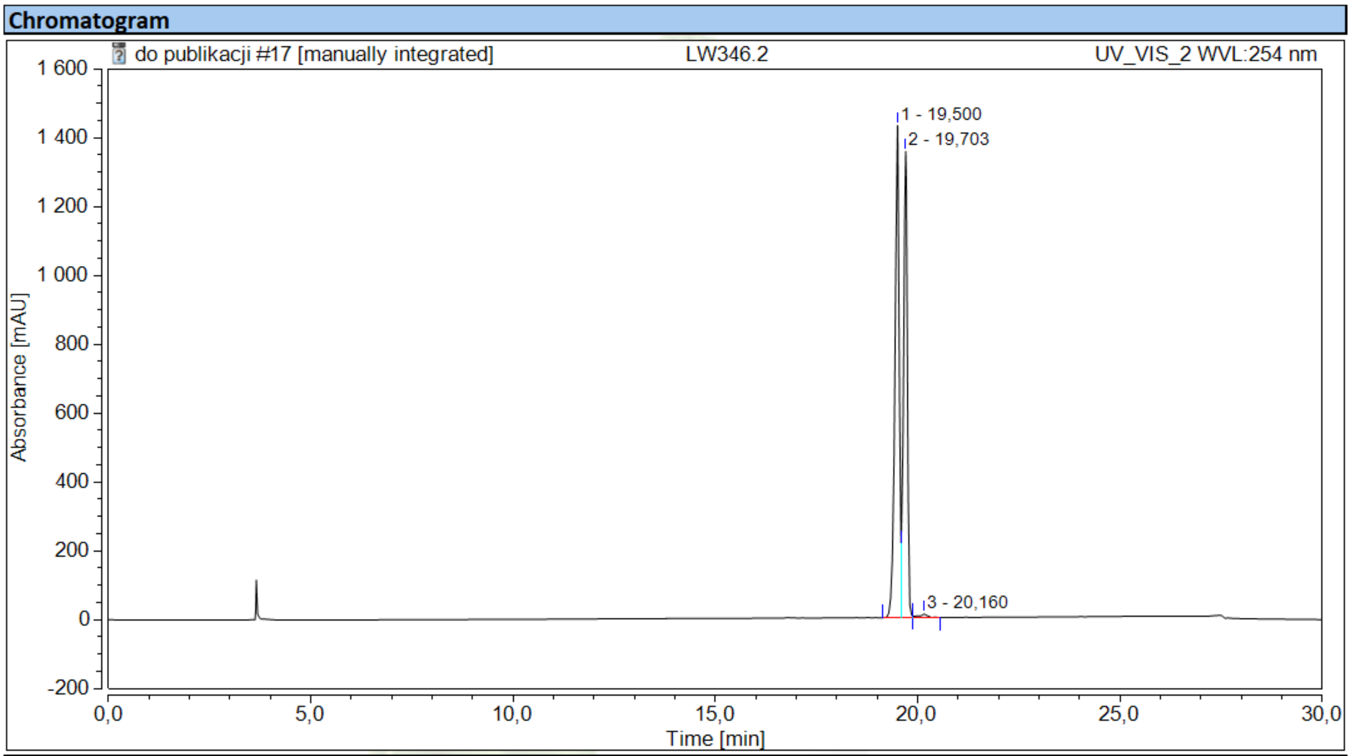

Integration Results

No.	Peak Name	Retention Time min	Area mAU*min	Height mAU	Relative Area %	Relative Height %	Amount n.a.
1		19,500	172,959	1429,321	53,22	51,16	n.a.
2		19,703	149,534	1354,174	46,02	48,47	n.a.
3		20,160	2,469	10,525	0,76	0,38	n.a.
**Total:**			**324,962**	**2794,020**	**100,00**	**100,00**	

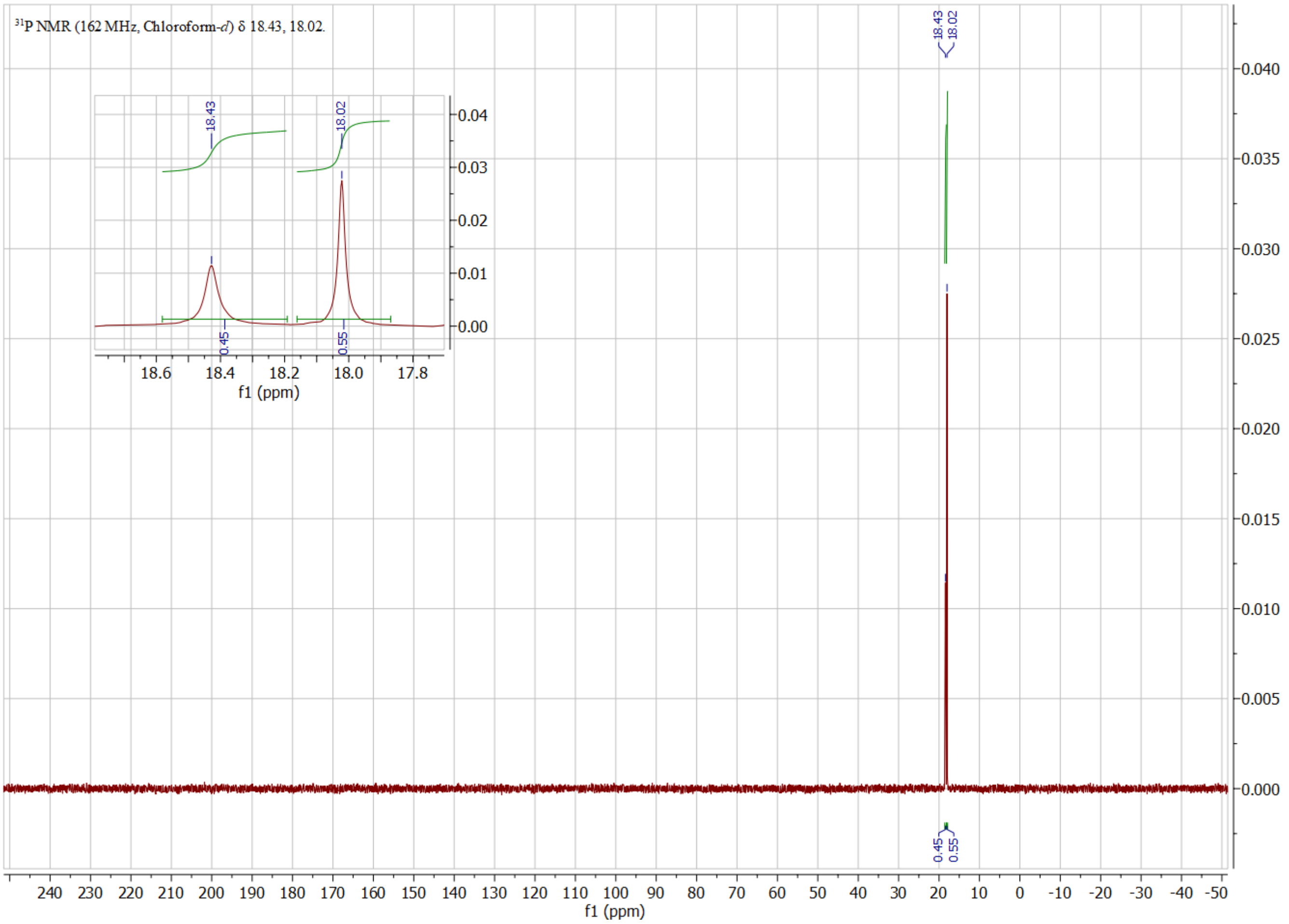
31P NMR (162 MHz, Chloroform-d) δ 18.43, 18.02.
18.43
18.02
0.45
0.55
f1 (ppm)

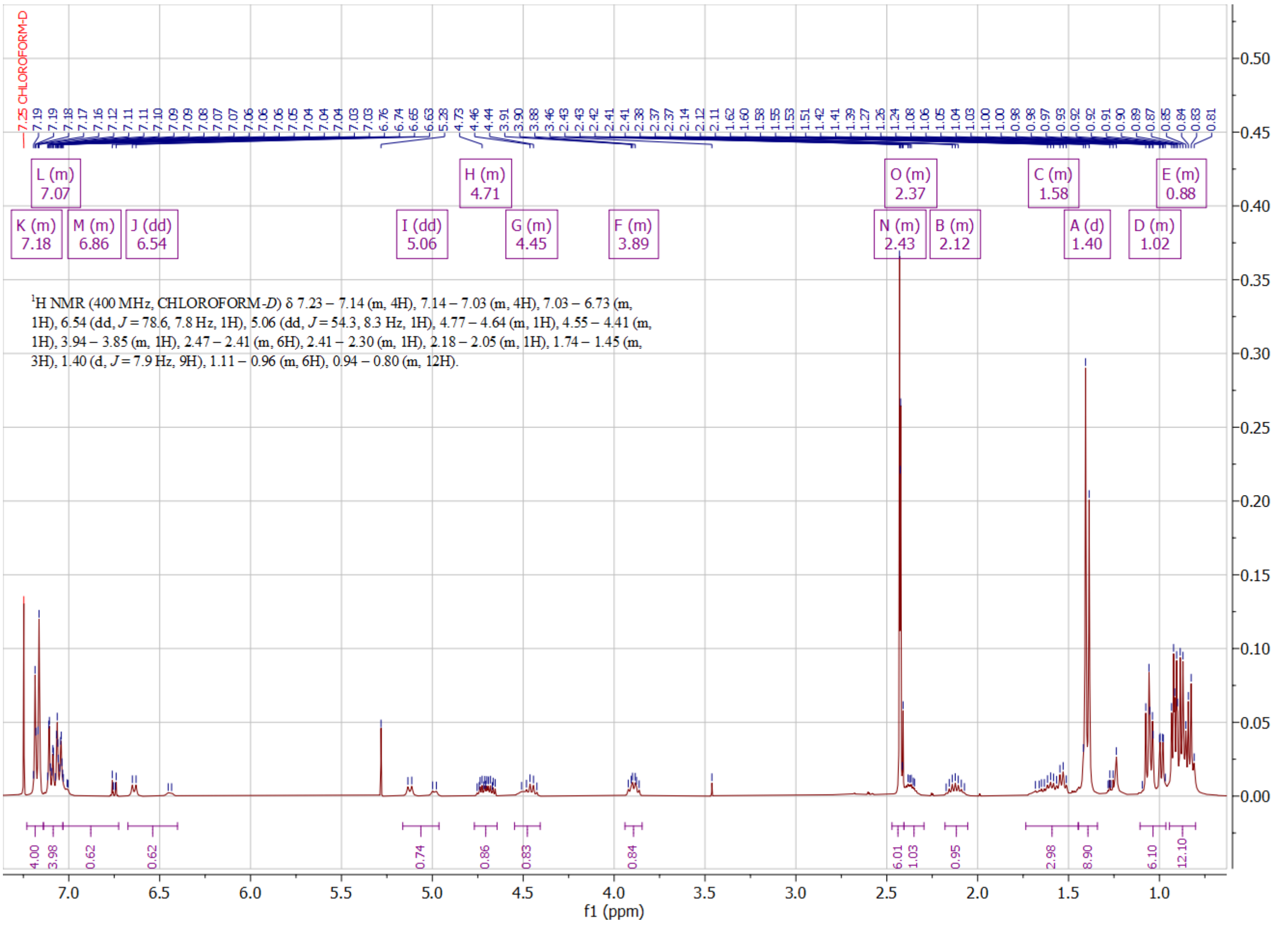
7.25 CHLOROFORM-D
L (m) 7.07
H (m) 4.71
O (m) 2.37
C (m) 1.58
E (m) 0.88
K (m) 7.18
M (m) 6.86
J (dd) 6.54
I (dd) 5.06
G (m) 4.45
F (m) 3.89
N (m) 2.43
B (m) 2.12
A (d) 1.40
D (m) 1.02
1H NMR (400 MHz, CHLOROFORM-D) δ 7.23 – 7.14 (m, 4H), 7.14 – 7.03 (m, 4H), 7.03 – 6.73 (m, 1H), 6.54 (dd, J = 78.6, 7.8 Hz, 1H), 5.06 (dd, J = 54.3, 8.3 Hz, 1H), 4.77 – 4.64 (m, 1H), 4.55 – 4.41 (m, 1H), 3.94 – 3.85 (m, 1H), 2.47 – 2.41 (m, 6H), 2.41 – 2.30 (m, 1H), 2.18 – 2.05 (m, 1H), 1.74 – 1.45 (m, 3H), 1.40 (d, J = 7.9 Hz, 9H), 1.11 – 0.96 (m, 6H), 0.94 – 0.80 (m, 12H).
f1 (ppm)

(18) Boc-L-Val-L-NptGly-ValP(OPh-4-SCH3)2

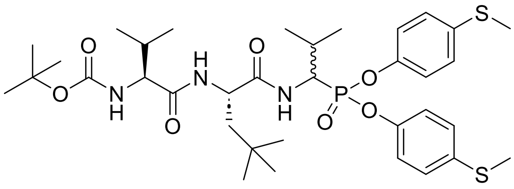

Name: *tert*-butyl ((2S)-1-(((2S)-1-((1-(bis(4-(methylthio)phenoxy)phosphoryl)-2-methylpropyl)amino)-4,4-dimethyl-1-oxopentan-2-yl)amino)-3-methyl-1-oxobutan-2-yl)carbamate

^{31}P NMR (162 MHz, Chloroform-d) δ 18.53 (s, 50%), 18.32 (s, 50%).

^{1}H NMR (400 MHz, Chloroform-d) δ 7.22 – 7.01 (m, 8H, 8XArH), 6.84 (dd, J = 37.1, 10.0 Hz, 1H, NH), 6.40 (d, J = 7.9 Hz, 1H, NH), 4.93 (dd, J = 42.9, 8.4 Hz, 1H, NH), 4.74 – 4.62 (m, 1H, CHP), 4.53 – 4.41 (m, 1H, CH), 3.89 – 3.78 (m, 1H, CH), 2.47 – 2.41 (m, 6H, 2xSCH$_3$), 2.41 – 2.28 (m, 1H, CH), 2.21 – 2.06 (m, 1H, CH), 1.98 – 1.80 (m, 2H, CH$_2$), 1.41 (d, J = 10.6 Hz, 9H, Boc), 1.11 – 0.99 (m, 6H, 2xCH$_3$), 0.91 (d, J = 12.4 Hz, 9H, 3xCH$_3$), 0.92 – 0.82 (m, 6H, 2xCH$_3$).

HRMS: $C_{35}H_{54}N_3O_7PS_2$ [M+Na]$^+$ calc. 746.3038 found 746.3046

HPLC: purity >98%; retention time 19.840 and 20.033 min

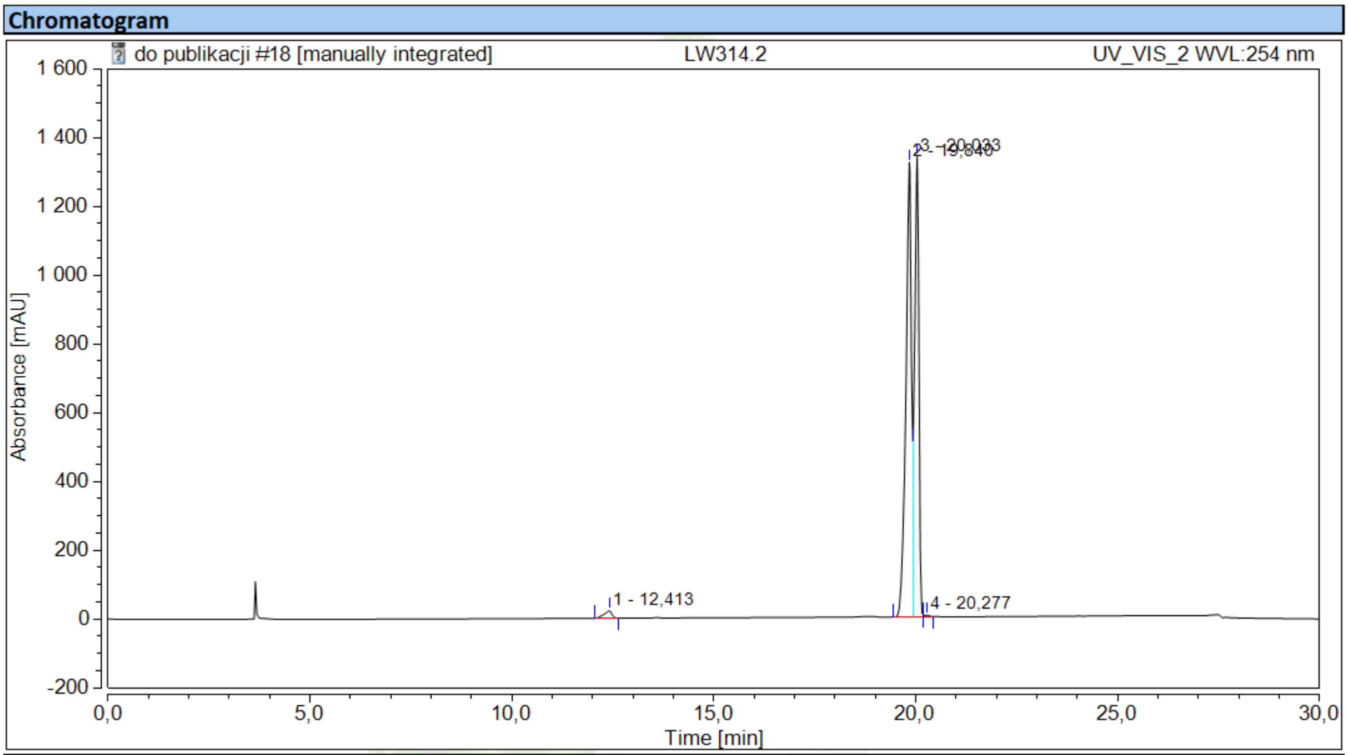

Integration Results

No.	Peak Name	Retention Time min	Area mAU*min	Height mAU	Relative Area %	Relative Height %	Amount n.a.
1		12,413	4,915	22,539	1,31	0,84	n.a.
2		19,840	205,463	1321,675	54,68	49,21	n.a.
3		20,033	164,804	1337,354	43,86	49,79	n.a.
4		20,277	0,567	4,424	0,15	0,16	n.a.
**Total:**			**375,749**	**2685,992**	**100,00**	**100,00**	

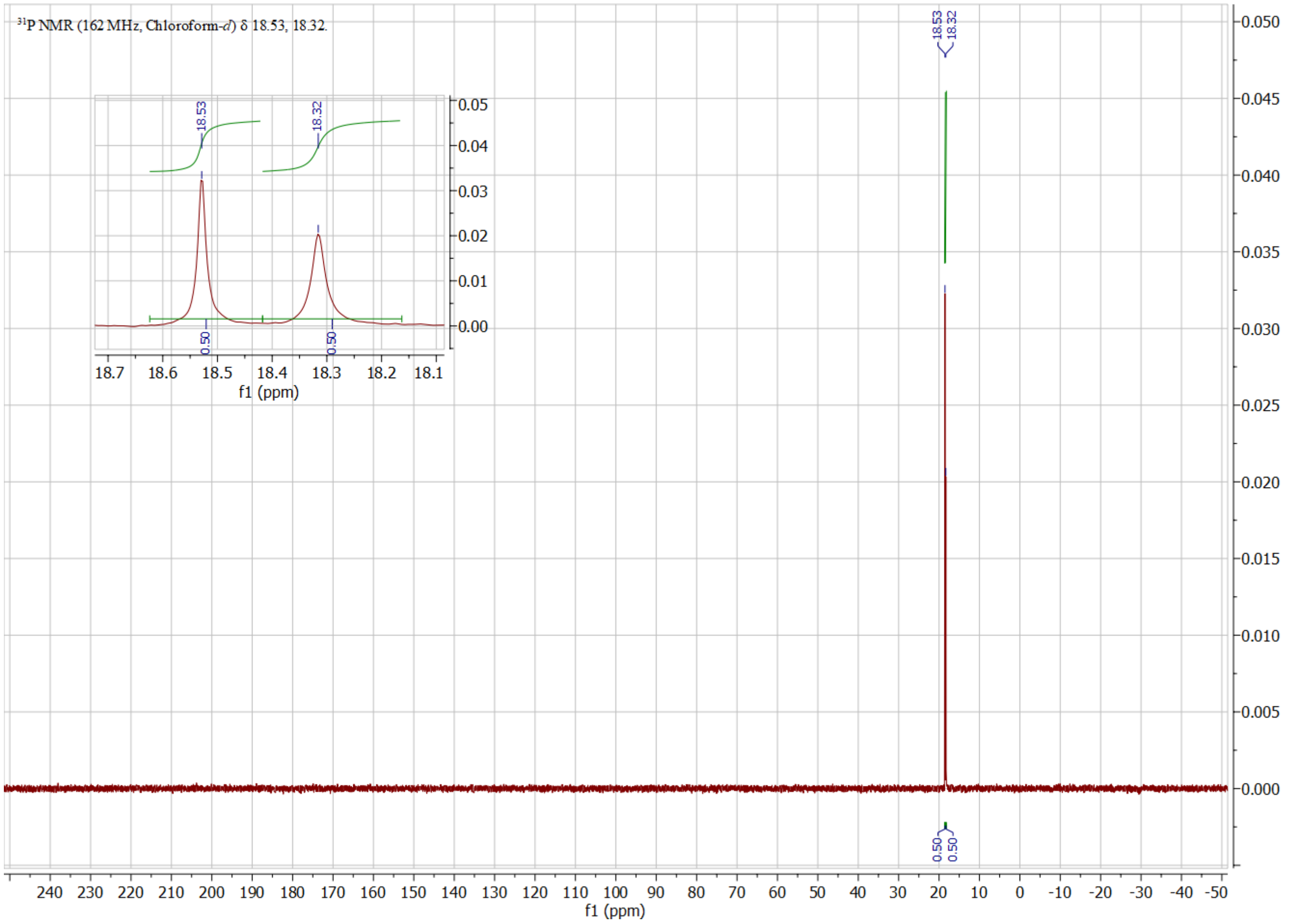
^{31}P NMR (162 MHz, Chloroform-*d*) δ 18.53, 18.32.
f1 (ppm)

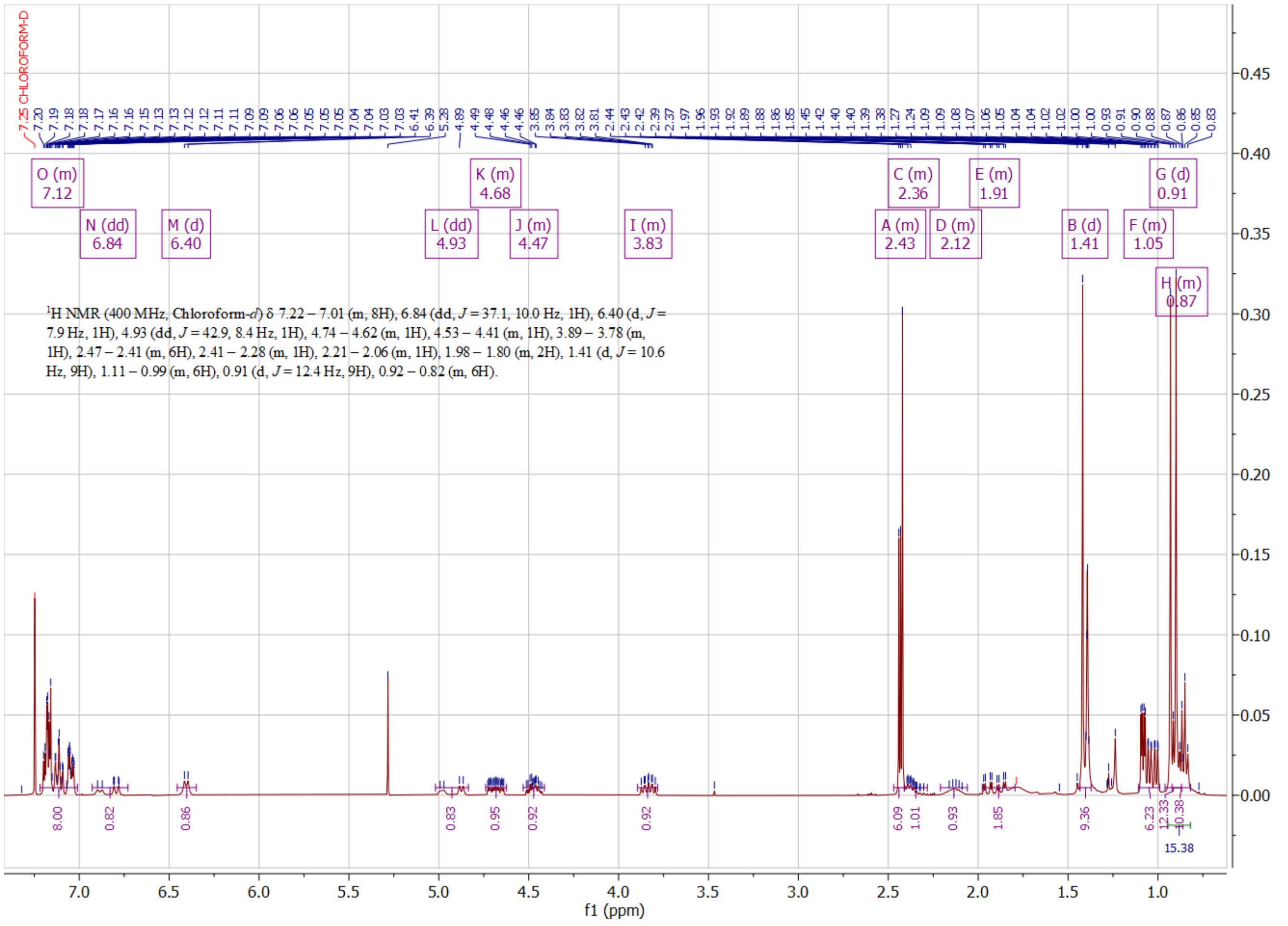
^{1}H NMR (400 MHz, Chloroform-*d*) δ 7.22 – 7.01 (m, 8H), 6.84 (dd, *J* = 37.1, 10.0 Hz, 1H), 6.40 (d, *J* = 7.9 Hz, 1H), 4.93 (dd, *J* = 42.9, 8.4 Hz, 1H), 4.74 – 4.62 (m, 1H), 4.53 – 4.41 (m, 1H), 3.89 – 3.78 (m, 1H), 2.47 – 2.41 (m, 6H), 2.41 – 2.28 (m, 1H), 2.21 – 2.06 (m, 1H), 1.98 – 1.80 (m, 2H), 1.41 (d, *J* = 10.6 Hz, 9H), 1.11 – 0.99 (m, 6H), 0.91 (d, *J* = 12.4 Hz, 9H), 0.92 – 0.82 (m, 6H).
7.25 CHLOROFORM-D
f1 (ppm)

(19) Boc-L-Val-L-Met-Val^{P}(OPh-4-SCH$_3$)$_2$

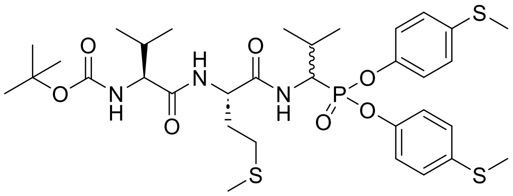

Name: *tert*-butyl ((2S)-1-(((2S)-1-((1-(bis(4-(methylthio)phenoxy)phosphoryl)-2-methylpropyl)amino)-4-(methylthio)-1-oxobutan-2-yl)amino)-3-methyl-1-oxobutan-2-yl)carbamate

^{31}P NMR (162 MHz, Chloroform-d) δ 18.08 (s).

^{1}H NMR (400 MHz, Chloroform-d) δ 7.21 – 7.14 (m, 4H, 4xArH), 7.12 (dd, *J* = 8.9, 1.3 Hz, 1H, NH), 7.09 – 6.97 (m, 4H, 4xArH), 6.88 (dd, *J* = 53.7, 7.7 Hz, 1H, NH), 5.02 (dd, *J* = 33.4, 8.0 Hz, 1H, NH), 4.77 – 4.63 (m, 2H, CHP, CH), 3.93 – 3.83 (m, 1H, CH), 2.62 – 2.45 (m, 2H, CH$_2$), 2.45 – 2.41 (m, 6H, 2xSCH$_3$), 2.41 – 2.31 (m, 1H, CH), 2.18 – 1.93 (m, 3H, CH$_2$, CH), 2.05 (d, *J* = 19.5 Hz, 3H, CH$_3$), 1.41 (d, *J* = 9.4 Hz, 9H, Boc), 1.12 – 0.99 (m, 6H, 2xCH$_3$), 0.96 – 0.82 (m, 6H, 2xCH$_3$).

HRMS: $C_{33}H_{50}N_3O_7PS_3$ [M+Na]$^+$ calc. 750.2446 found 750.2450

HPLC: purity >99%; retention time 18.793 and 18.963 min

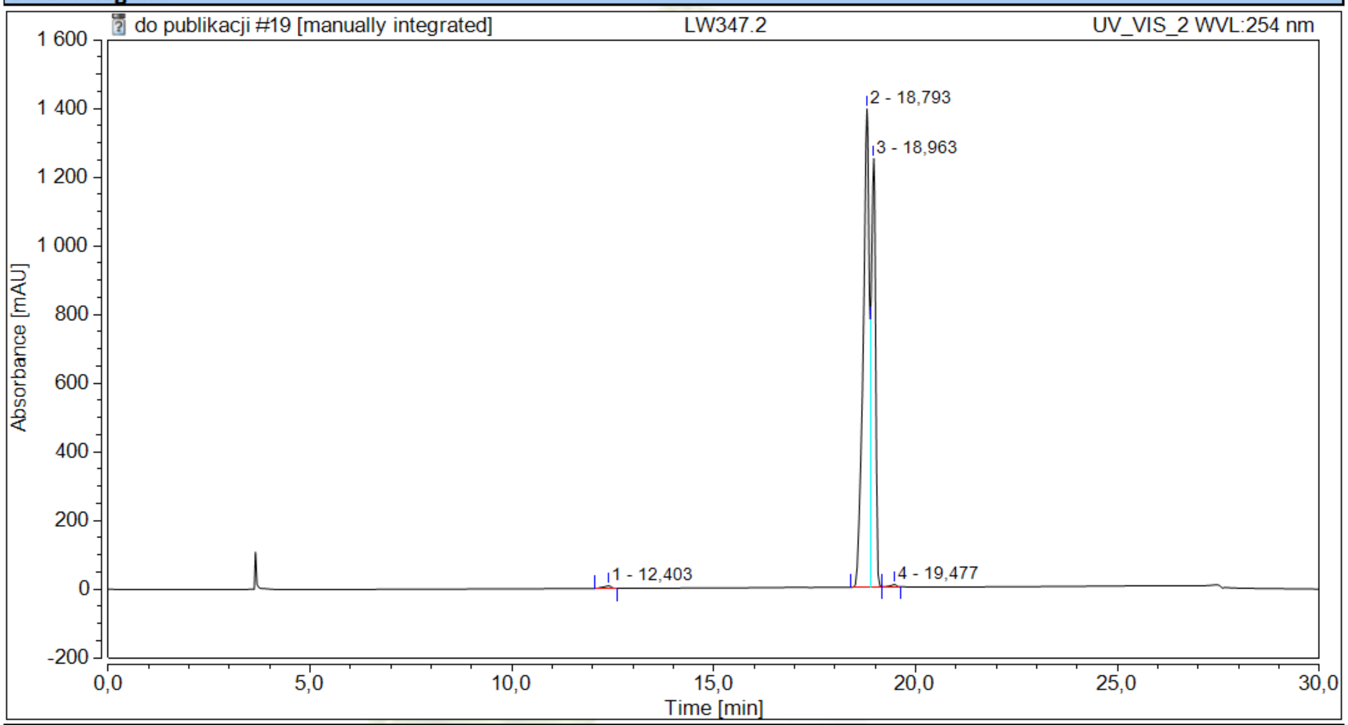

Integration Results

No.	Peak Name	Retention Time min	Area mAU*min	Height mAU	Relative Area %	Relative Height %	Amount n.a.
1		12,403	1,749	7,865	0,44	0,30	n.a.
2		18,793	237,376	1392,708	59,64	52,44	n.a.
3		18,963	157,496	1248,058	39,57	46,99	n.a.
4		19,477	1,362	7,378	0,34	0,28	n.a.
**Total:**			**397,984**	**2656,009**	**100,00**	**100,00**	

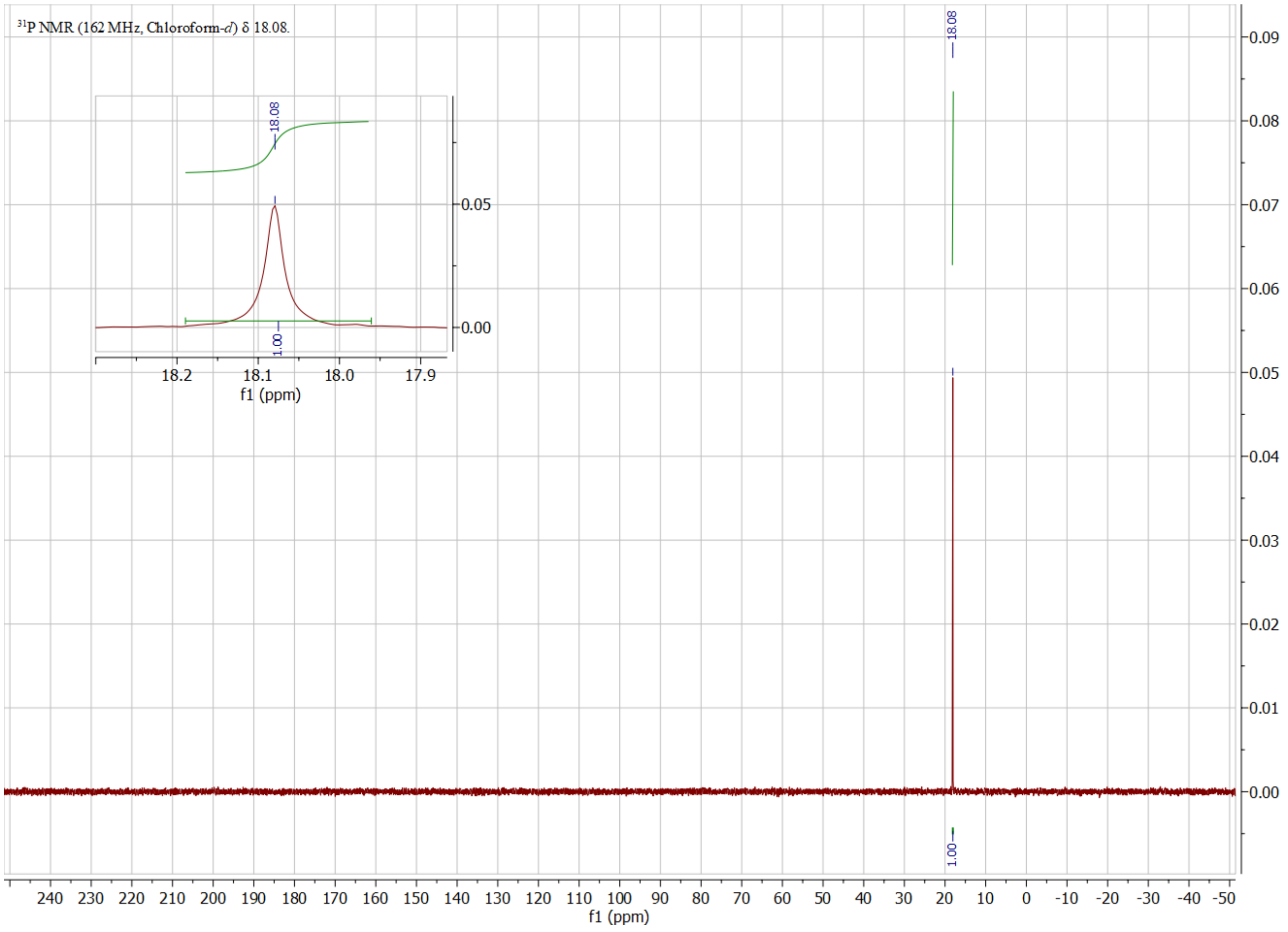

31P NMR (162 MHz, Chloroform-d) δ 18.08.
18.08
1.00
f1 (ppm)

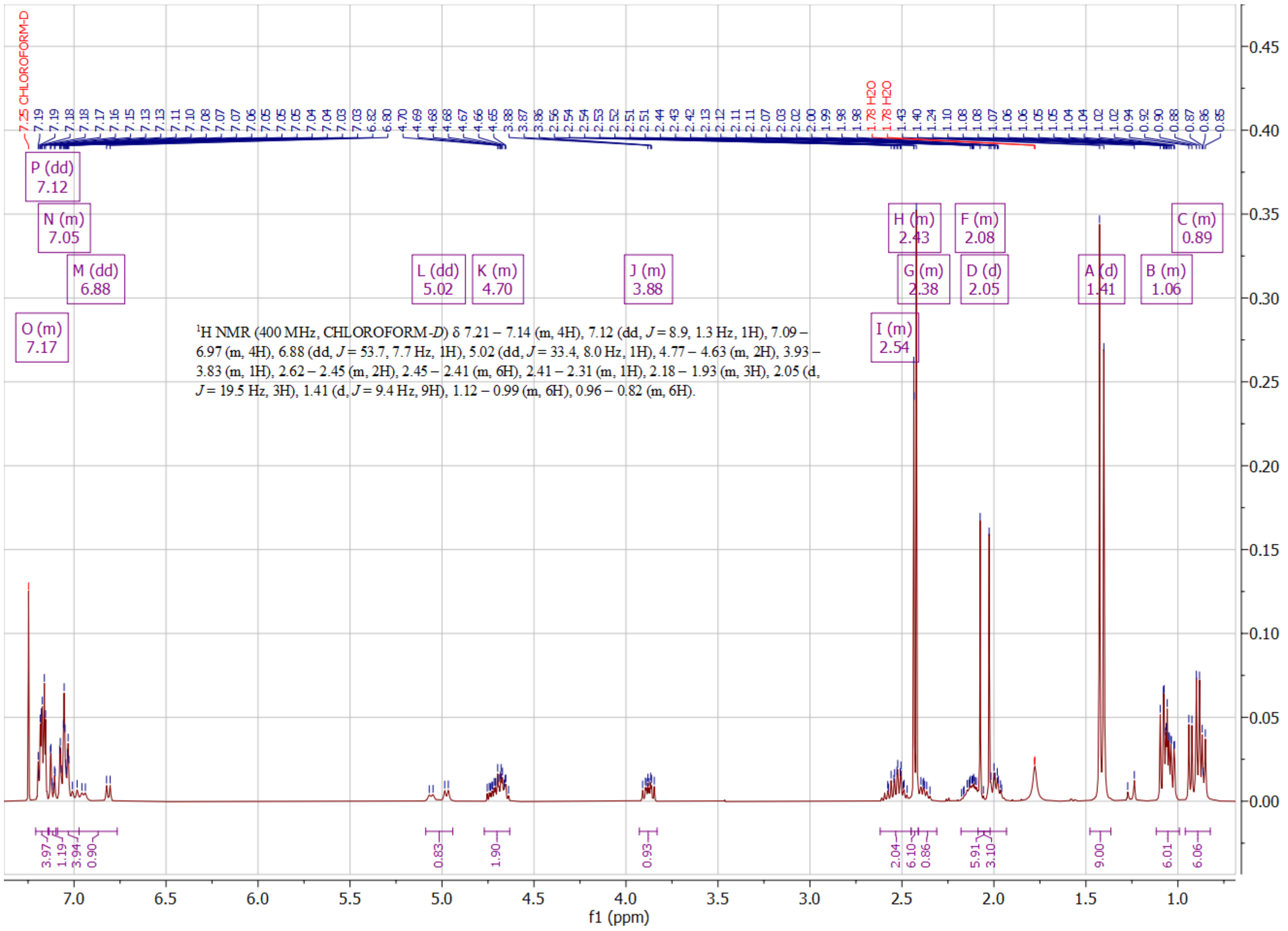

1H NMR (400 MHz, CHLOROFORM-D) δ 7.21 – 7.14 (m, 4H), 7.12 (dd, J = 8.9, 1.3 Hz, 1H), 7.09 – 6.97 (m, 4H), 6.88 (dd, J = 53.7, 7.7 Hz, 1H), 5.02 (dd, J = 33.4, 8.0 Hz, 1H), 4.77 – 4.63 (m, 2H), 3.93 – 3.83 (m, 1H), 2.62 – 2.45 (m, 2H), 2.45 – 2.41 (m, 6H), 2.41 – 2.31 (m, 1H), 2.18 – 1.93 (m, 3H), 2.05 (d, J = 19.5 Hz, 3H), 1.41 (d, J = 9.4 Hz, 9H), 1.12 – 0.99 (m, 6H), 0.96 – 0.82 (m, 6H).
7.25 CHLOROFORM-D
1.78 H2O
P (dd) 7.12
N (m) 7.05
M (dd) 6.88
O (m) 7.17
L (dd) 5.02
K (m) 4.70
J (m) 3.88
H (m) 2.43
G (m) 2.38
I (m) 2.54
F (m) 2.08
D (d) 2.05
A (d) 1.41
B (m) 1.06
C (m) 0.89
f1 (ppm)

(20) Boc-L-Val-D-Met-Val^P(OPh-4-SCH$_3$)$_2$

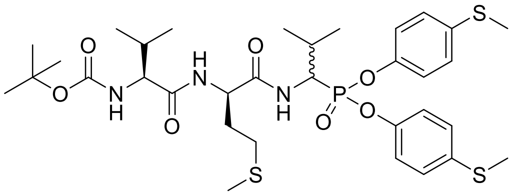

Name: *tert*-butyl ((2S)-1-(((2R)-1-((1-(bis(4-(methylthio)phenoxy)phosphoryl)-2-methylpropyl)amino)-4-(methylthio)-1-oxobutan-2-yl)amino)-3-methyl-1-oxobutan-2-yl)carbamate

^{31}P NMR (162 MHz, Chloroform-d) δ 18.15 (s, 46%), 18.00 (s, 54%).

^{1}H NMR (400 MHz, Chloroform-d) δ 7.22 – 7.01 (m, 9H, 8xArH, NH), 6.87 (dd, *J* = 21.5, 7.8 Hz, 1H, NH), 5.05 (dd, *J* = 42.1, 8.3 Hz, 1H, NH), 4.75 – 4.64 (m, 2H, CHP, CH), 3.98 – 3.85 (m, 1H, CH), 2.61 – 2.45 (m, 2H, CH$_2$), 2.45 – 2.42 (m, 6H, , 2xSCH$_3$), 2.42 – 2.32 (m, 1H, CH), 2.20 – 1.88 (m, 3H, CH$_2$, CH), 2.04 (dd, *J* = 17.9, 0.9 Hz, 3H, CH$_3$), 1.40 (d, *J* = 8.6 Hz, 9H, Boc), 1.11 – 0.98 (m, 6H, 2xCH$_3$), 0.96 – 0.81 (m, 6H, 2xCH$_3$).

HRMS: $C_{33}H_{50}N_3O_7PS_3$ [M+Na]$^+$ calc. 750.2446 found 750.2456

HPLC: purity >99%; retention time 18.843 and 19.030 min

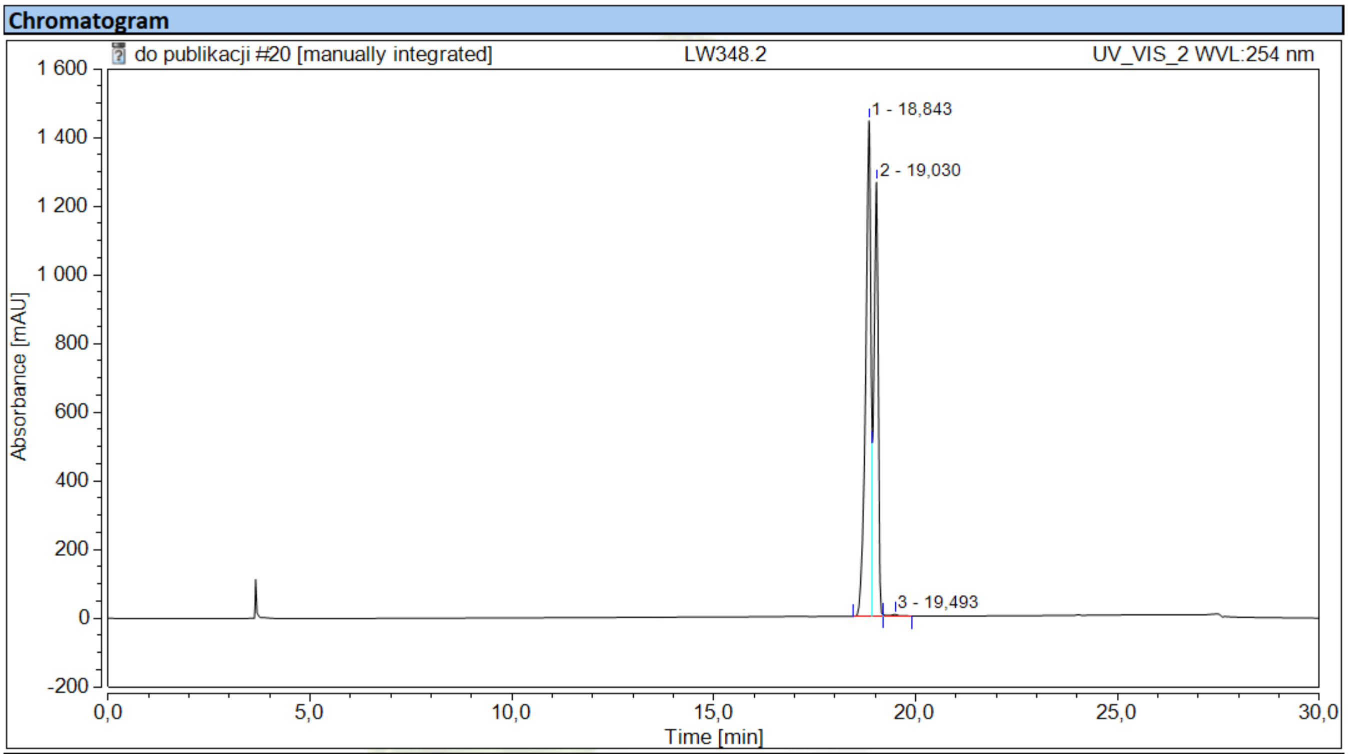

Integration Results

No.	Peak Name	Retention Time min	Area mAU*min	Height mAU	Relative Area %	Relative Height %	Amount n.a.
1		18,843	217,010	1443,215	58,32	53,20	n.a.
2		19,030	153,499	1264,124	41,25	46,60	n.a.
3		19,493	1,607	5,578	0,43	0,21	n.a.
**Total:**			**372,116**	**2712,917**	**100,00**	**100,00**	

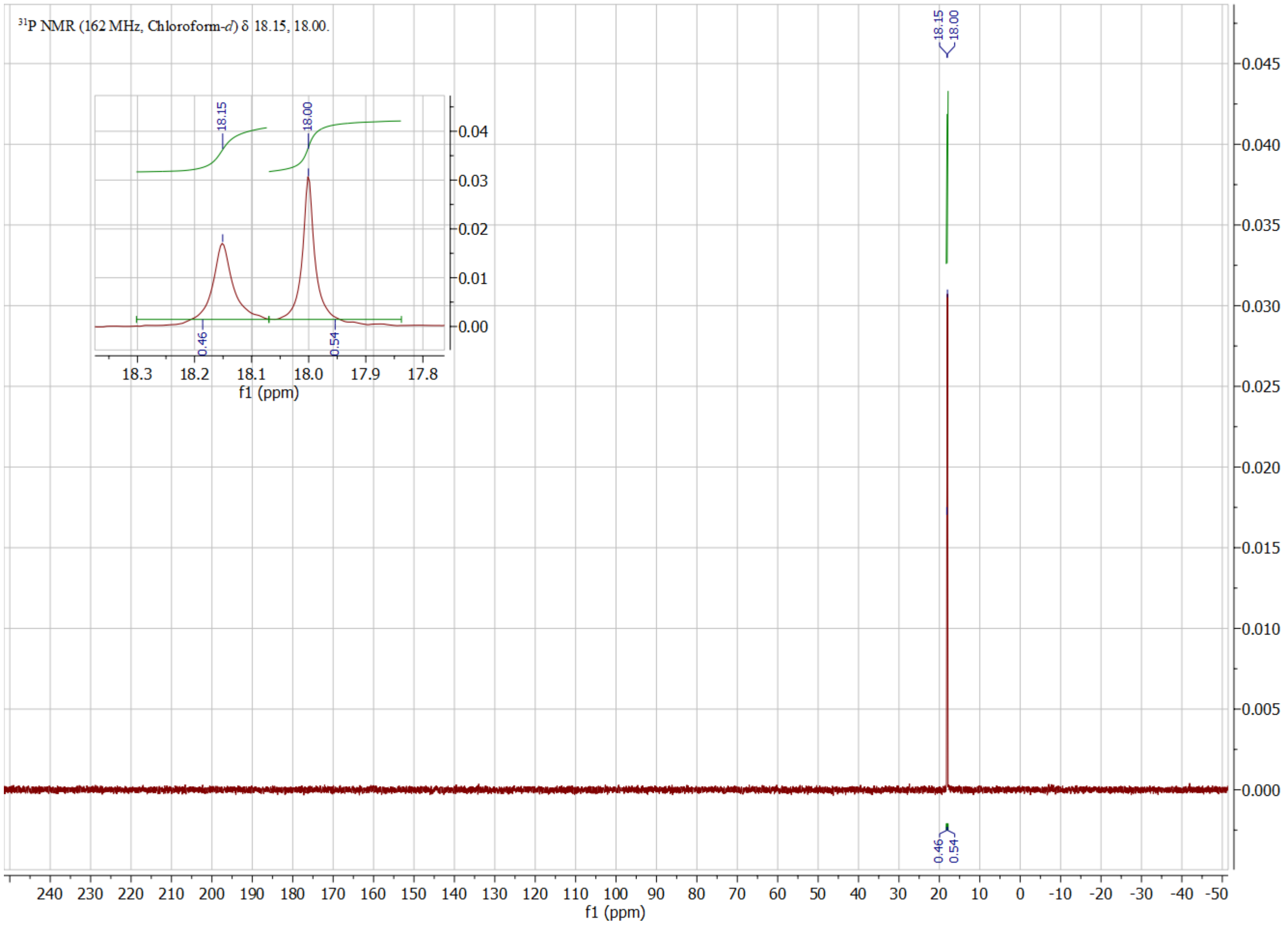
31P NMR (162 MHz, Chloroform-d) δ 18.15, 18.00.
18.15
18.00
0.46
0.54
f1 (ppm)

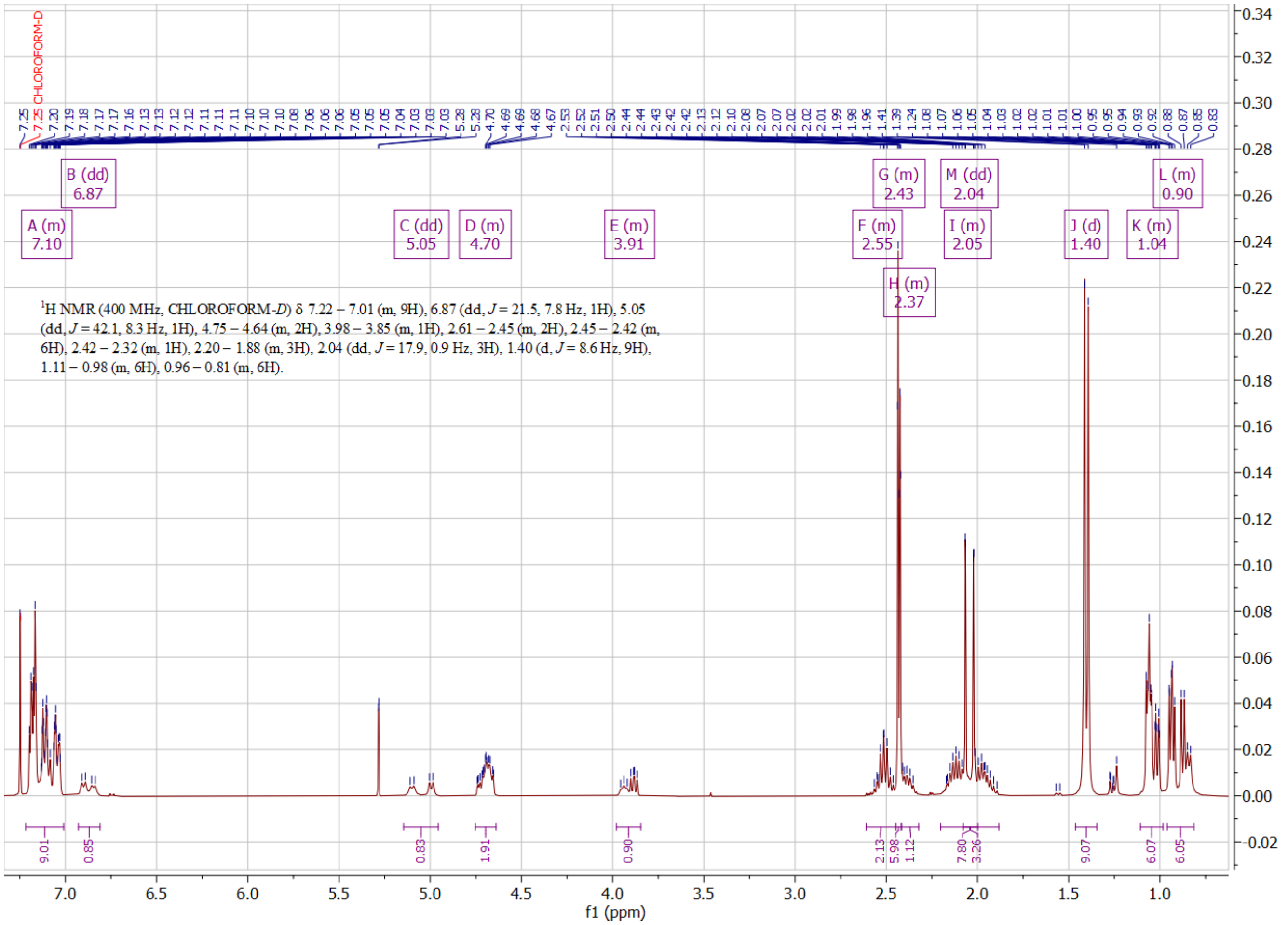
CHLOROFORM-D
A (m) 7.10
B (dd) 6.87
C (dd) 5.05
D (m) 4.70
E (m) 3.91
F (m) 2.55
G (m) 2.43
H (m) 2.37
I (m) 2.05
M (dd) 2.04
J (d) 1.40
K (m) 1.04
L (m) 0.90
1H NMR (400 MHz, CHLOROFORM-D) δ 7.22 – 7.01 (m, 9H), 6.87 (dd, J = 21.5, 7.8 Hz, 1H), 5.05 (dd, J = 42.1, 8.3 Hz, 1H), 4.75 – 4.64 (m, 2H), 3.98 – 3.85 (m, 1H), 2.61 – 2.45 (m, 2H), 2.45 – 2.42 (m, 6H), 2.42 – 2.32 (m, 1H), 2.20 – 1.88 (m, 3H), 2.04 (dd, J = 17.9, 0.9 Hz, 3H), 1.40 (d, J = 8.6 Hz, 9H), 1.11 – 0.98 (m, 6H), 0.96 – 0.81 (m, 6H).
9.01
0.85
0.83
1.91
0.90
2.13
5.98
1.12
7.80
3.26
9.07
6.07
6.05
f1 (ppm)

(21) Boc-L-Val-L-Met(O)- Val^P(OPh-4-SCH$_3$)$_2$

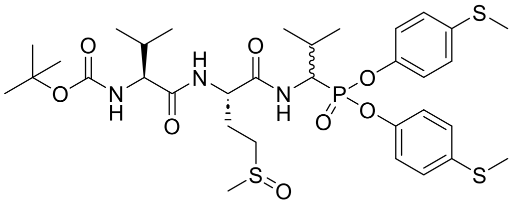

Name: *tert*-butyl ((2S)-1-(((2S)-1-((1-(bis(4-(methylthio)phenoxy)phosphoryl)-2-methylpropyl)amino)-4-(methylsulfinyl)-1-oxobutan-2-yl)amino)-3-methyl-1-oxobutan-2-yl)carbamate
^{31}P NMR (162 MHz, Chloroform-d) δ 18.50 (s, 26%), 18.48 (s, 25%), 18.28 (s, 18%), 18.16 (s, 31%).
^{1}H NMR (400 MHz, Chloroform-d) δ 8.06 – 7.41 (m, 1H, NH), 7.29 – 6.97 (m, 9H, 8xArH, NH), 5.23 – 5.09 (m, 1H, NH), 4.92 – 4.63 (m, 2H, CHP, CH), 3.99 – 3.85 (m, 1H, CH), 3.11 – 2.60 (m, 2H, CH$_2$), 2.60 – 2.45 (m, 3H, CH$_3$), 2.43 (dd, *J* = 3.8, 2.3 Hz, 6H, 2xSCH$_3$), 2.42 – 1.98 (m, 4H, CH$_2$, 2xCH), 1.41 (d, *J* = 9.9 Hz, 9H, Boc), 1.11 – 1.01 (m, 6H, 2xCH$_3$), 0.95 – 0.85 (m, 6H, 2xCH$_3$).
HRMS: $C_{33}H_{50}N_3O_8PS_3$ [M+Na]$^+$ calc. 766.2395 found 766.2402
HPLC: purity >99%; retention time 16.880 min

Chromatogram

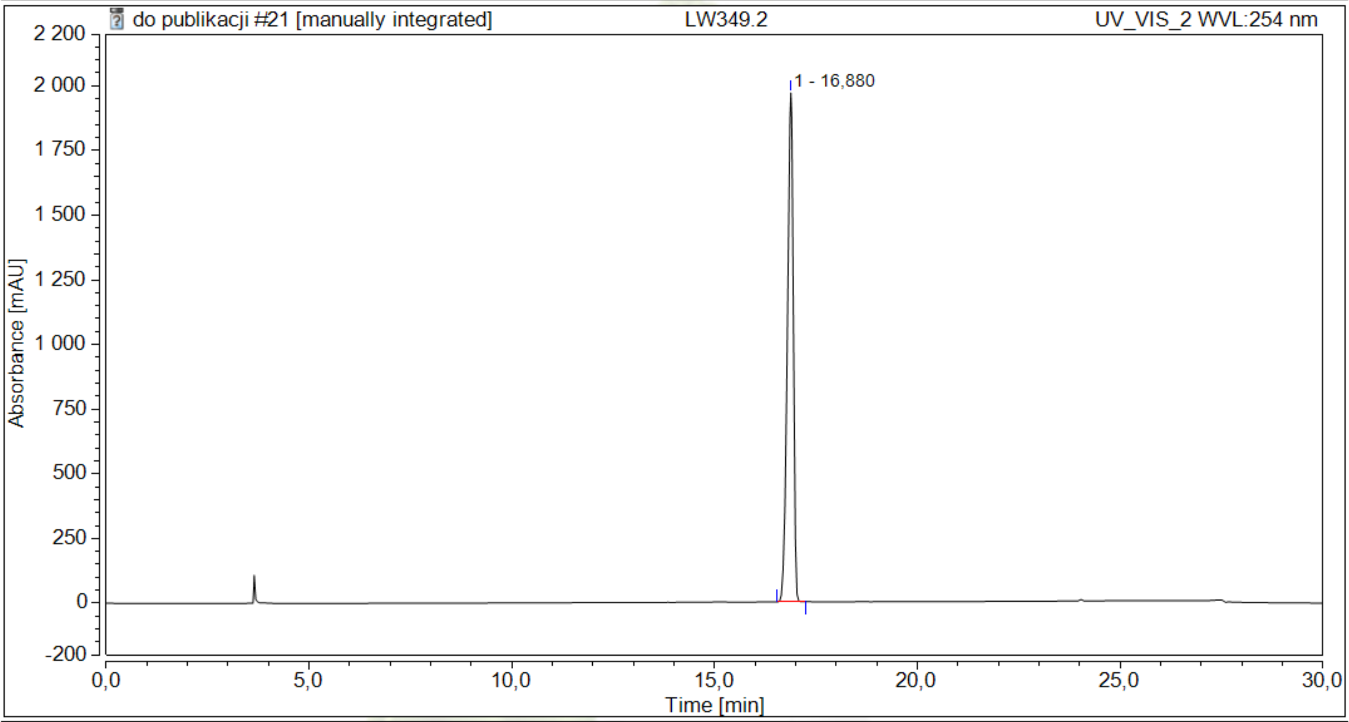

Integration Results

No.	Peak Name	Retention Time min	Area mAU*min	Height mAU	Relative Area %	Relative Height %	Amount n.a.
1		16,880	331,419	1967,646	100,00	100,00	n.a.
**Total:**			**331,419**	**1967,646**	**100,00**	**100,00**	

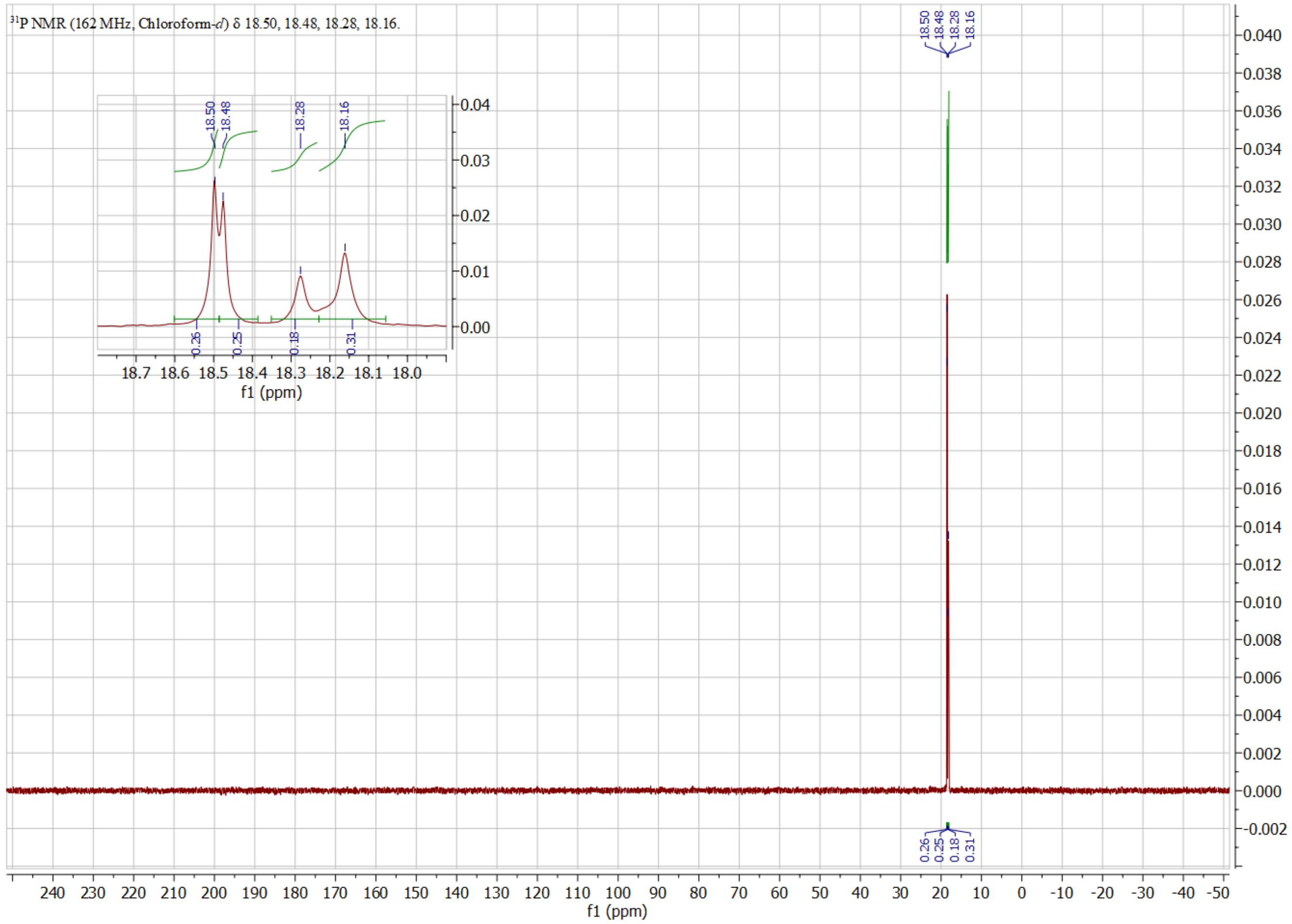
^{31}P NMR (162 MHz, Chloroform-*d*) δ 18.50, 18.48, 18.28, 18.16.
18.50
18.48
18.28
18.16
0.26
0.25
0.18
0.31
f1 (ppm)

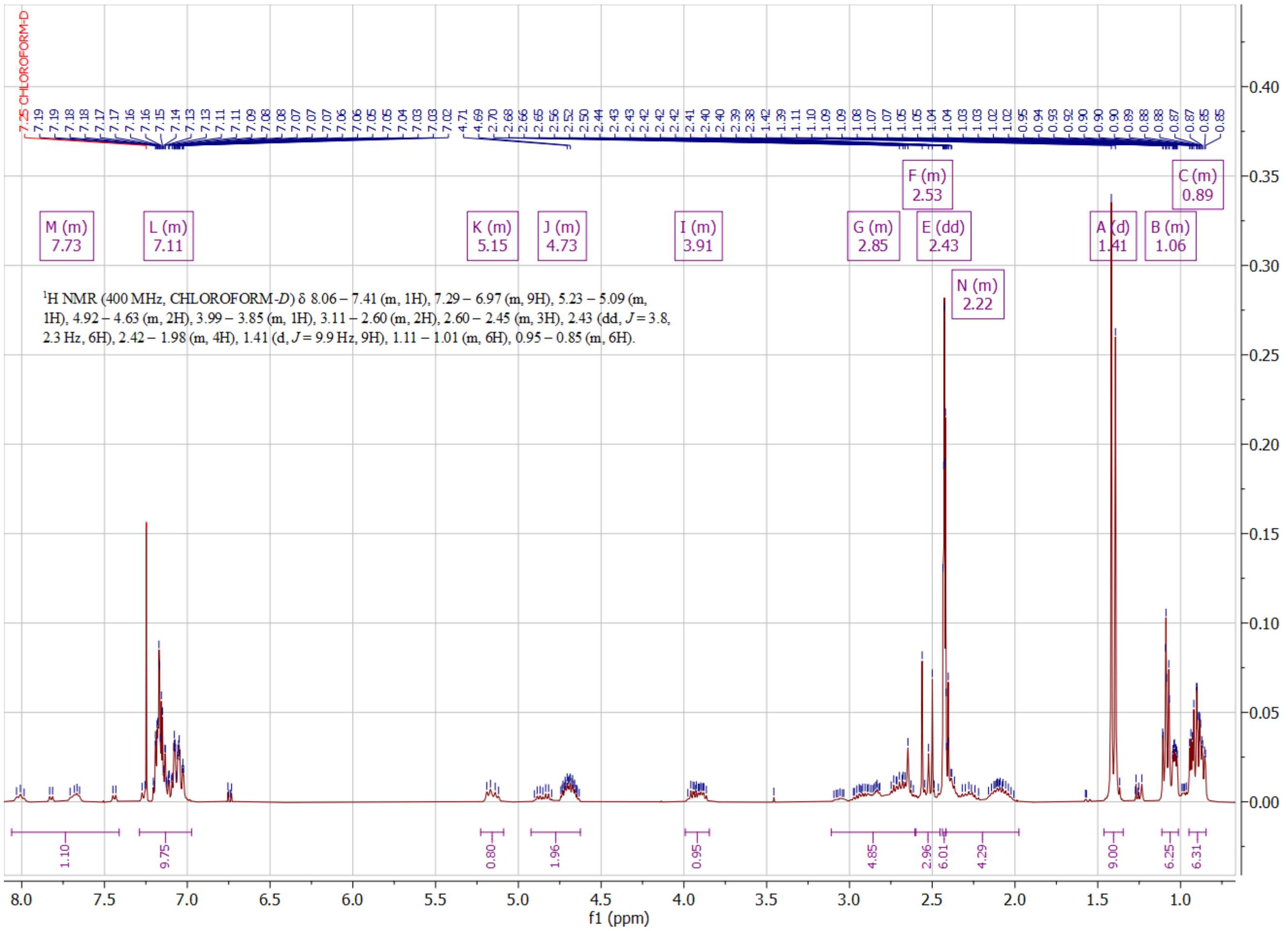
7.25 CHLOROFORM-D
M (m) 7.73
L (m) 7.11
K (m) 5.15
J (m) 4.73
I (m) 3.91
G (m) 2.85
F (m) 2.53
E (dd) 2.43
N (m) 2.22
A (d) 1.41
B (m) 1.06
C (m) 0.89
^{1}H NMR (400 MHz, CHLOROFORM-*D*) δ 8.06 – 7.41 (m, 1H), 7.29 – 6.97 (m, 9H), 5.23 – 5.09 (m, 1H), 4.92 – 4.63 (m, 2H), 3.99 – 3.85 (m, 1H), 3.11 – 2.60 (m, 2H), 2.60 – 2.45 (m, 3H), 2.43 (dd, *J* = 3.8, 2.3 Hz, 6H), 2.42 – 1.98 (m, 4H), 1.41 (d, *J* = 9.9 Hz, 9H), 1.11 – 1.01 (m, 6H), 0.95 – 0.85 (m, 6H).
1.10
9.75
0.80
1.96
0.95
4.85
2.96
6.01
4.29
9.00
6.25
6.31
f1 (ppm)

(22) Boc-L-Val-L-Met(O2)- Val^P(OPh-4-SCH$_3$)$_2$

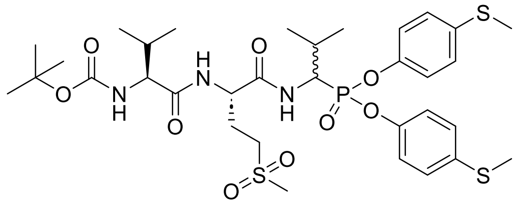

Name: *tert*-butyl ((2S)-1-(((2S)-1-((1-(bis(4-(methylthio)phenoxy)phosphoryl)-2-methylpropyl)amino)-4-(methylsulfonyl)-1-oxobutan-2-yl)amino)-3-methyl-1-oxobutan-2-yl)carbamate
^{31}P NMR (162 MHz, Chloroform-d) δ 18.06 (s, 52%), 17.73 (s, 48%).
^{1}H NMR (400 MHz, Chloroform-d) δ 7.56 (dd, *J* = 50.6, 10.2 Hz, 1H, NH), 7.23 – 6.95 (m, 9H, 8xArH, NH), 5.19 (dd, *J* = 49.4, 7.6 Hz, 1H, NH), 4.87 – 4.62 (m, 2H, CHP, CH), 3.93 – 3.80 (m, 1H, CH), 3.29 – 3.03 (m, 2H, CH$_2$), 2.85 (d, *J* = 27.7 Hz, 3H, CH$_3$), 2.42 (d, *J* = 2.8 Hz, 6H, 2xSCH$_3$), 2.39 – 2.31 (m, 1H, CH), 2.30 – 2.13 (m, 2H, CH$_2$), 2.13 – 2.00 (m, 1H, CH), 1.41 (d, *J* = 8.4 Hz, 9H, Boc), 1.14 – 0.99 (m, 6H, 2xCH$_3$), 0.96 – 0.77 (m, 6H, 2xCH$_3$).
HRMS: $C_{33}H_{50}N_3O_9PS_3$ [M+Na]$^+$ calc. 782.2344 found 782.2357
HPLC: purity >99%; retention time 17.573 min

Chromatogram

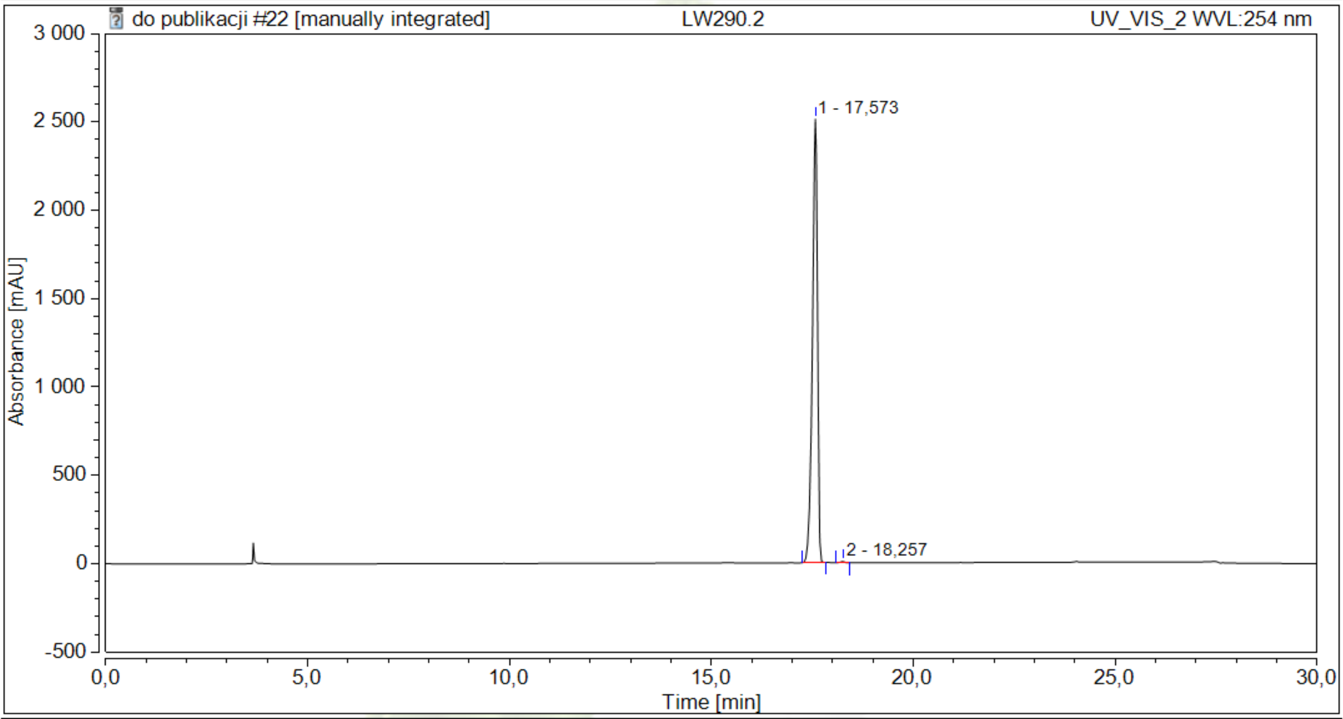

Integration Results

No.	Peak Name	Retention Time min	Area mAU*min	Height mAU	Relative Area %	Relative Height %	Amount n.a.
1		17,573	362,729	2509,071	99,78	99,71	n.a.
2		18,257	0,808	7,248	0,22	0,29	n.a.
**Total:**			**363,536**	**2516,319**	**100,00**	**100,00**	

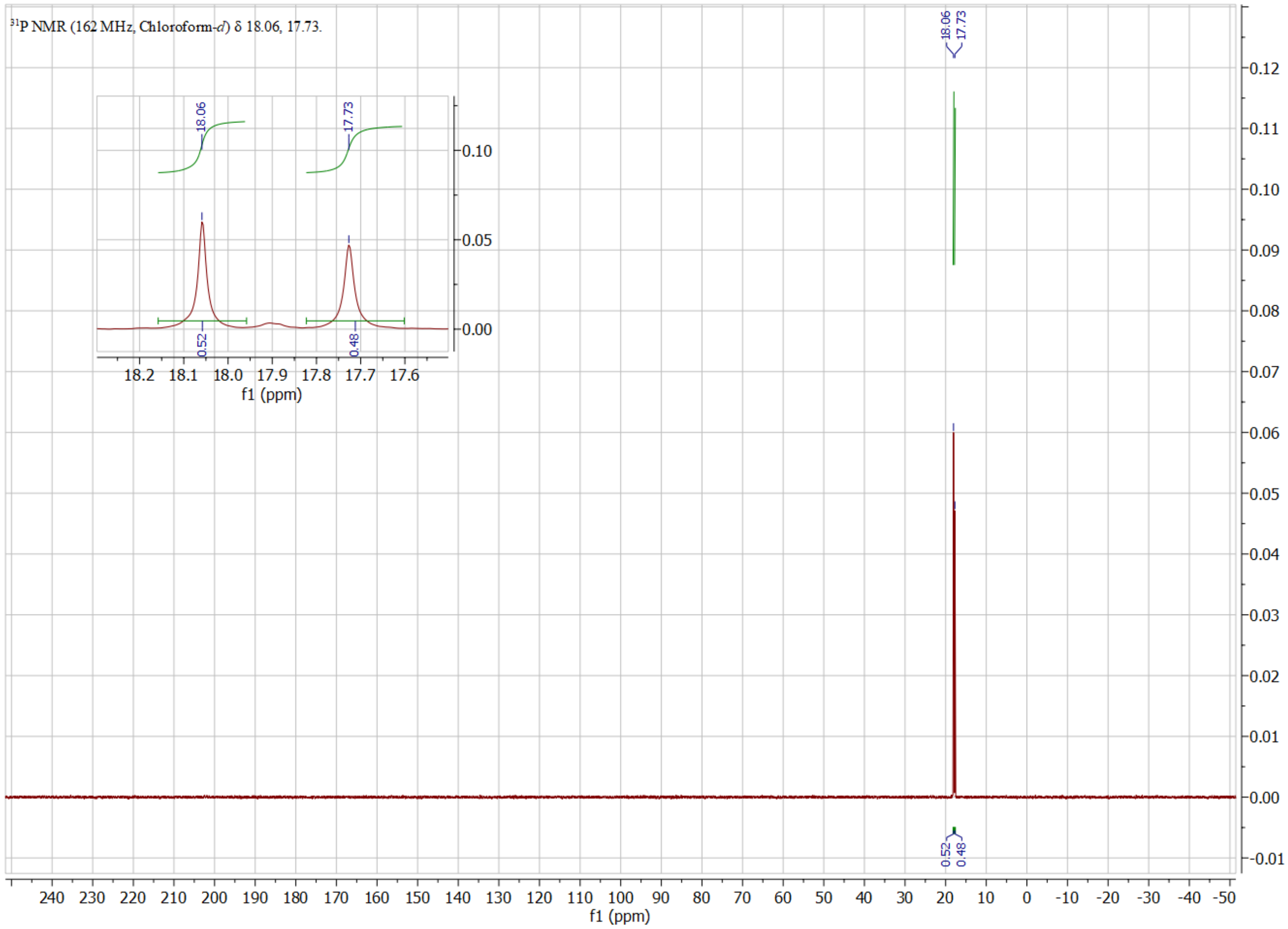
31P NMR (162 MHz, Chloroform-d) δ 18.06, 17.73.
f1 (ppm)

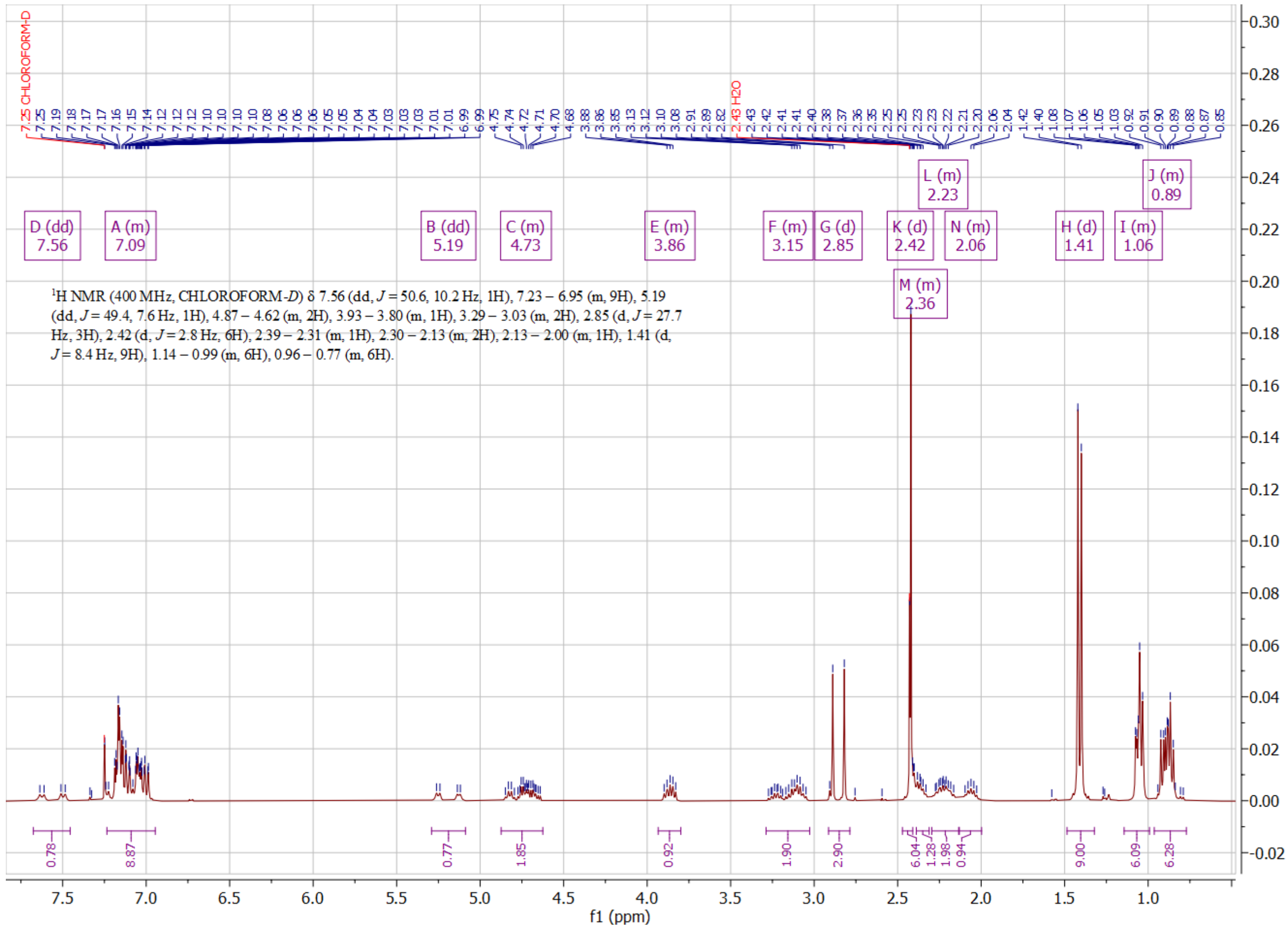
1H NMR (400 MHz, CHLOROFORM-D) δ 7.56 (dd, J = 50.6, 10.2 Hz, 1H), 7.23 – 6.95 (m, 9H), 5.19 (dd, J = 49.4, 7.6 Hz, 1H), 4.87 – 4.62 (m, 2H), 3.93 – 3.80 (m, 1H), 3.29 – 3.03 (m, 2H), 2.85 (d, J = 27.7 Hz, 3H), 2.42 (d, J = 2.8 Hz, 6H), 2.39 – 2.31 (m, 1H), 2.30 – 2.13 (m, 2H), 2.13 – 2.00 (m, 1H), 1.41 (d, J = 8.4 Hz, 9H), 1.14 – 0.99 (m, 6H), 0.96 – 0.77 (m, 6H).
f1 (ppm)

(23) Boc-L-Val-L-Ser(Bzl)-Val^{P}(OPh-4-SCH$_3$)$_2$

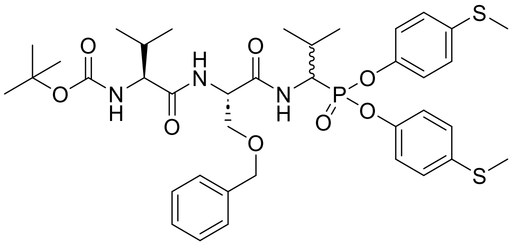

Name: *tert*-butyl ((2S)-1-(((2S)-3-(benzyloxy)-1-((1-(bis(4-(methylthio)phenoxy)phosphoryl)-2-methylpropyl)amino)-1-oxopropan-2-yl)amino)-3-methyl-1-oxobutan-2-yl)carbamate

^{31}P NMR (162 MHz, Chloroform-d) δ 18.15 (s, 36%), 18.04 (s, 14%), 17.93 (s, 50%).

^{1}H NMR (400 MHz, Chloroform-d) δ 7.32 – 7.25 (m, 5H, 5xArH), 7.20 – 6.90 (m, 9H, 8xArH, NH), 6.87 – 6.75 (m, 1H, NH), 5.09 – 4.90 (m, 1H, NH), 4.79 – 4.64 (m, 1H, CHP), 4.64 – 4.46 (m, 3H, CH$_2$, CH), 3.99 – 3.87 (m, 2H, CH$_2$), 3.58 – 3.42 (m, 1H, CH), 2.42 (d, J = 1.9 Hz, 6H, 2xSCH$_3$), 2.41 – 2.30 (m, 1H, CH), 2.26 – 2.05 (m, 1H, CH), 1.43 – 1.36 (m, 9H, Boc), 1.06 – 0.84 (m, 12H, 4xCH$_3$).

HRMS: $C_{38}H_{52}N_3O_8PS_2$ [M+Na]$^+$ calc. 796.2831 found 796.2838

HPLC: purity >99%; retention time 19.817 and 19.940 min

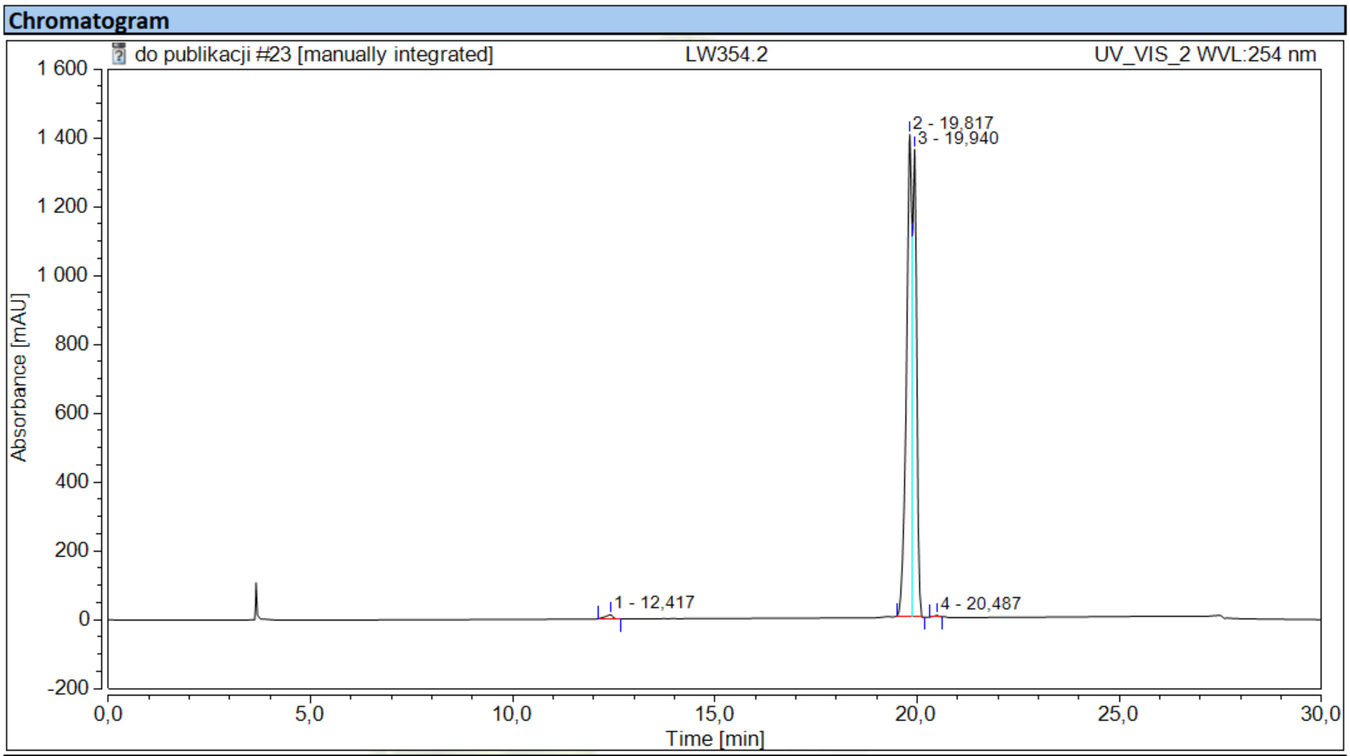

Integration Results

No.	Peak Name	Retention Time min	Area mAU*min	Height mAU	Relative Area %	Relative Height %	Amount n.a.
1		12,417	2,569	12,368	0,69	0,45	n.a.
2		19,817	214,920	1400,877	57,64	50,47	n.a.
3		19,940	154,836	1358,220	41,52	48,93	n.a.
4		20,487	0,559	4,434	0,15	0,16	n.a.
**Total:**			**372,883**	**2775,899**	**100,00**	**100,00**	

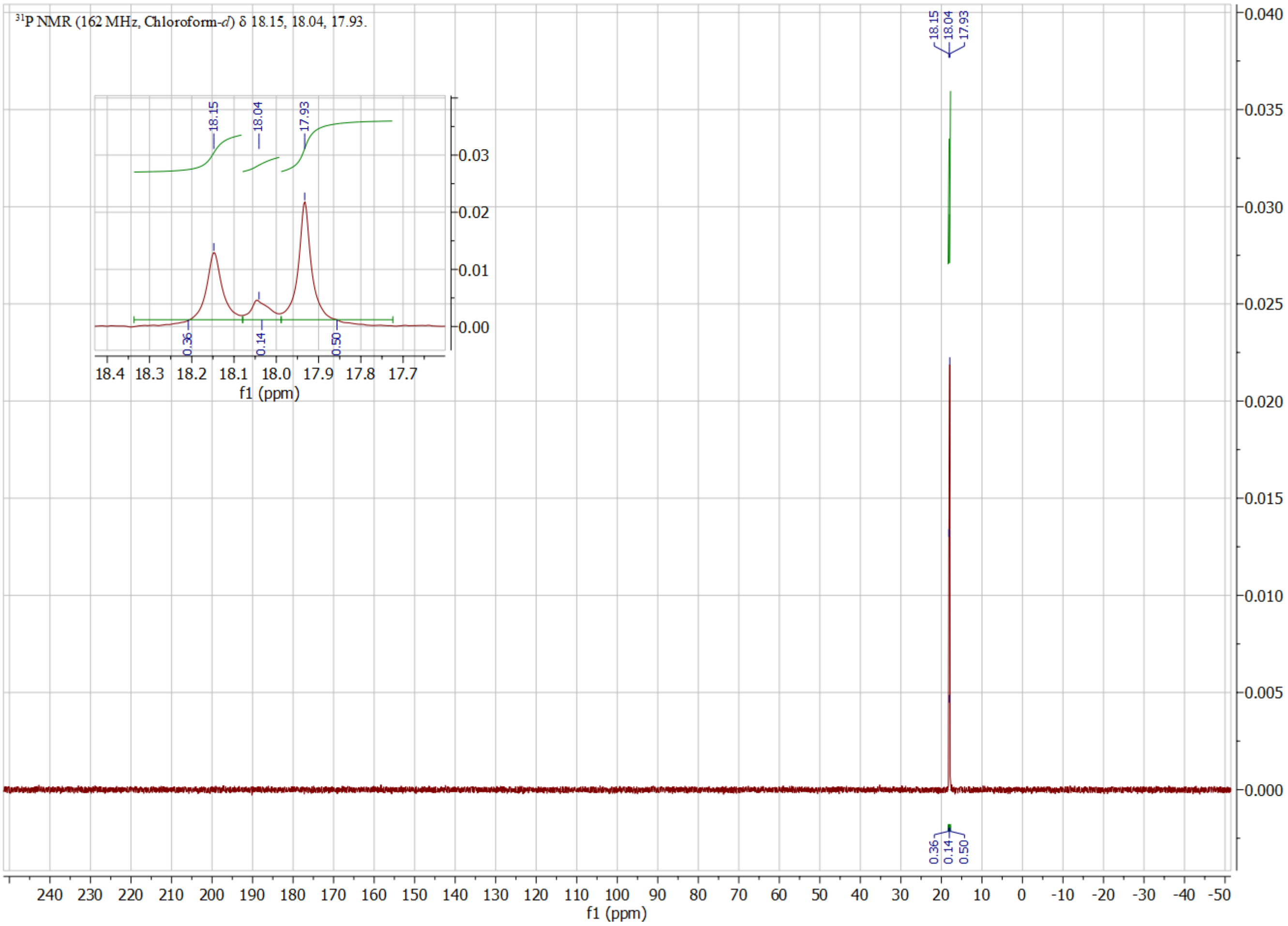
31P NMR (162 MHz, Chloroform-d) δ 18.15, 18.04, 17.93.
f1 (ppm)

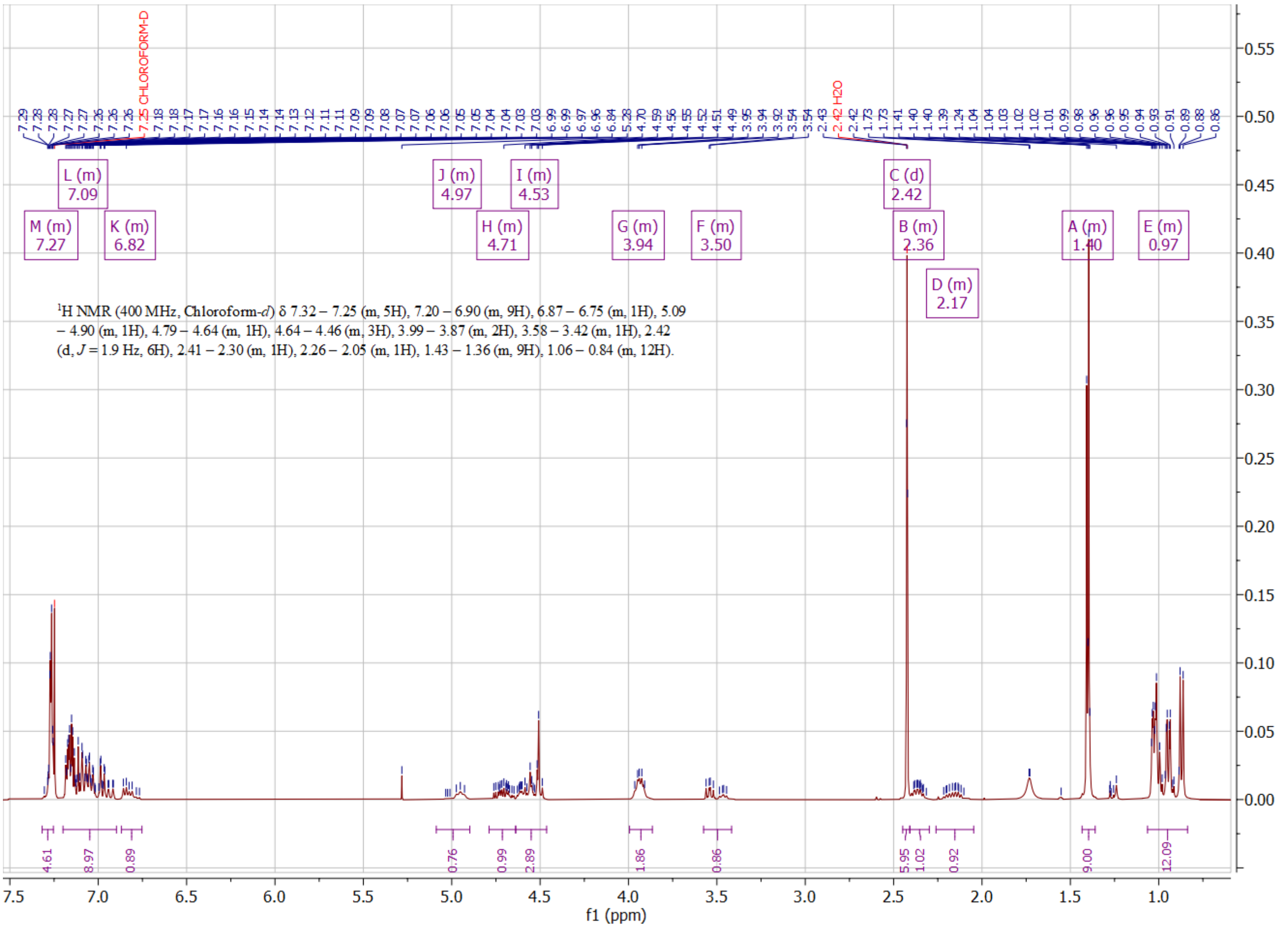
1H NMR (400 MHz, Chloroform-d) δ 7.32 – 7.25 (m, 5H), 7.20 – 6.90 (m, 9H), 6.87 – 6.75 (m, 1H), 5.09 – 4.90 (m, 1H), 4.79 – 4.64 (m, 1H), 4.64 – 4.46 (m, 3H), 3.99 – 3.87 (m, 2H), 3.58 – 3.42 (m, 1H), 2.42 (d, J = 1.9 Hz, 6H), 2.41 – 2.30 (m, 1H), 2.26 – 2.05 (m, 1H), 1.43 – 1.36 (m, 9H), 1.06 – 0.84 (m, 12H).
CHLOROFORM-D
H2O
f1 (ppm)

(24) Boc-L-Val-D-Ser(Bzl)-Val^{P}(OPh-4-SCH$_3$)$_2$

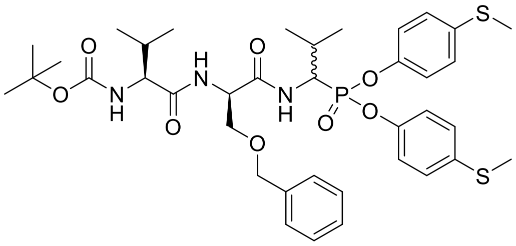

Name: *tert*-butyl ((2S)-1-(((2R)-3-(benzyloxy)-1-((1-(bis(4-(methylthio)phenoxy)phosphoryl)-2-methylpropyl)amino)-1-oxopropan-2-yl)amino)-3-methyl-1-oxobutan-2-yl)carbamate
^{31}P NMR (162 MHz, Chloroform-d) δ 18.04 (s, 51%), 18.00 (s, 49%).
^{1}H NMR (400 MHz, Chloroform-d) δ 7.29 – 7.24 (m, 5H, 5xArH), 7.24 – 6.93 (m, 9H, 8xArH, NH), 6.81 (dd, *J* = 21.8, 6.9 Hz, 1H, NH), 5.07 – 4.93 (m, 1H, NH), 4.77 – 4.65 (m, 1H, CHP), 4.65 – 4.45 (m, 3H, CH$_2$, CH), 4.01 – 3.83 (m, 2H, CH$_2$), 3.57 – 3.39 (m, 1H, CH), 2.46 – 2.40 (m, 6H, 2xSCH$_3$), 2.40 – 2.31 (m, 1H, CH), 2.19 – 2.01 (m, 1H, CH), 1.39 (d, *J* = 4.4 Hz, 9H, Boc), 1.06 – 0.83 (m, 12H, 4xCH$_3$).
HRMS: $C_{38}H_{52}N_3O_8PS_2$ [M+Na]+ calc. 796.2831 found 796.2821
HPLC: purity >98%; retention time 19.850 and 20.010 min

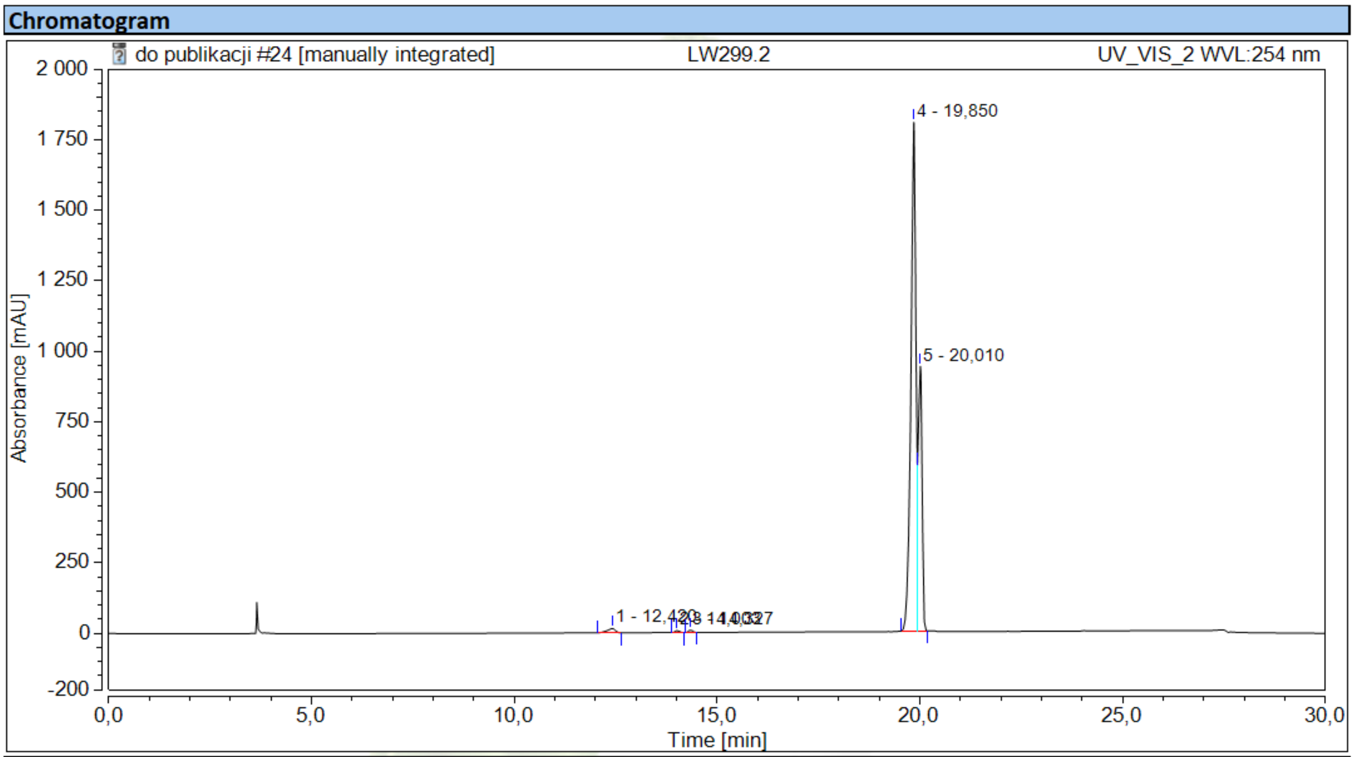

Integration Results

No.	Peak Name	Retention Time min	Area mAU*min	Height mAU	Relative Area %	Relative Height %	Amount n.a.
1		12,420	3,485	15,880	0,96	0,57	n.a.
2		14,003	0,816	6,782	0,23	0,24	n.a.
3		14,327	1,032	8,124	0,29	0,29	n.a.
4		19,850	251,688	1802,596	69,69	65,05	n.a.
5		20,010	104,145	937,750	28,84	33,84	n.a.
**Total:**			**361,166**	**2771,133**	**100,00**	**100,00**	

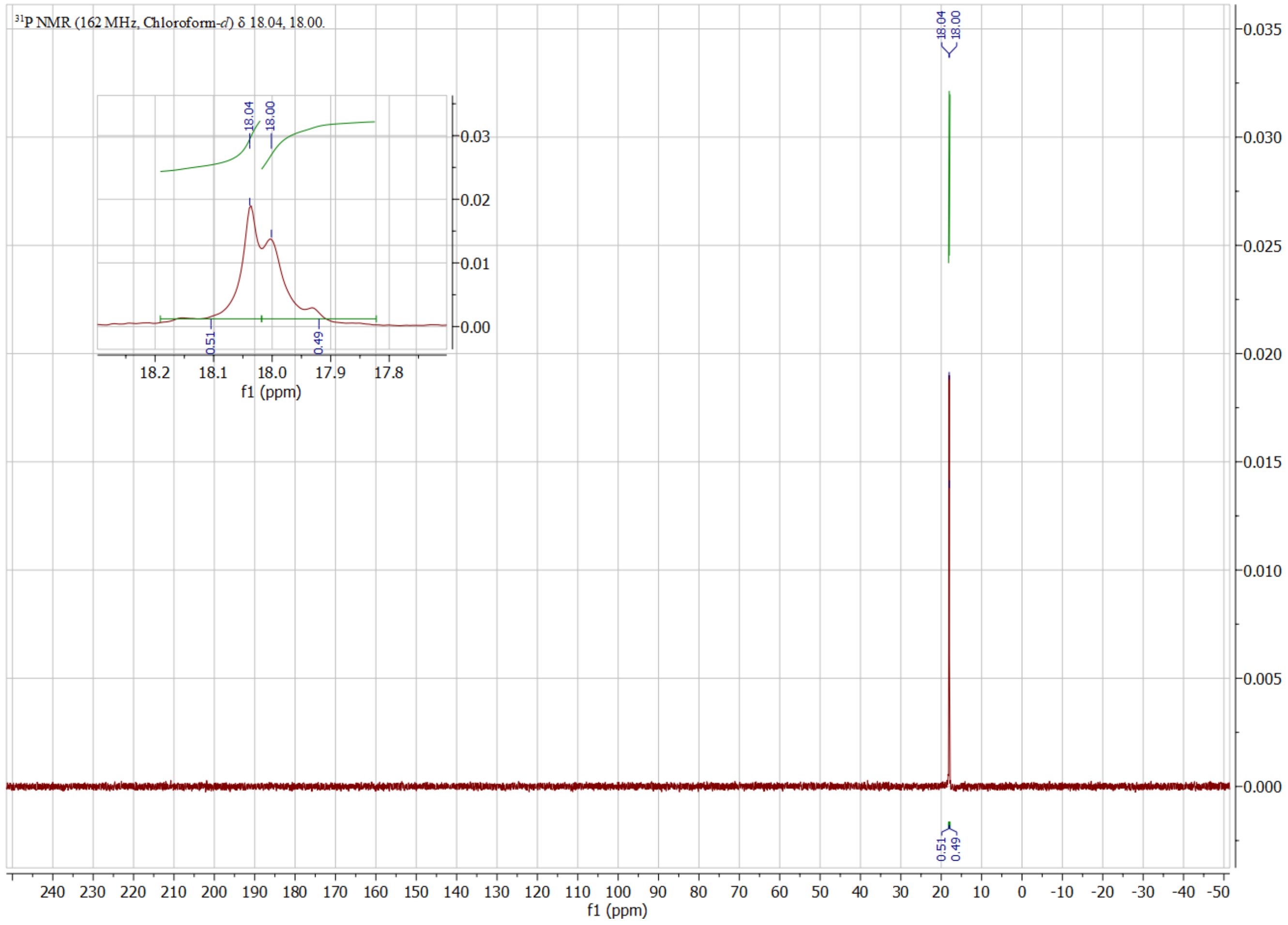
31P NMR (162 MHz, Chloroform-d) δ 18.04, 18.00.
18.04
18.00
0.51
0.49
f1 (ppm)

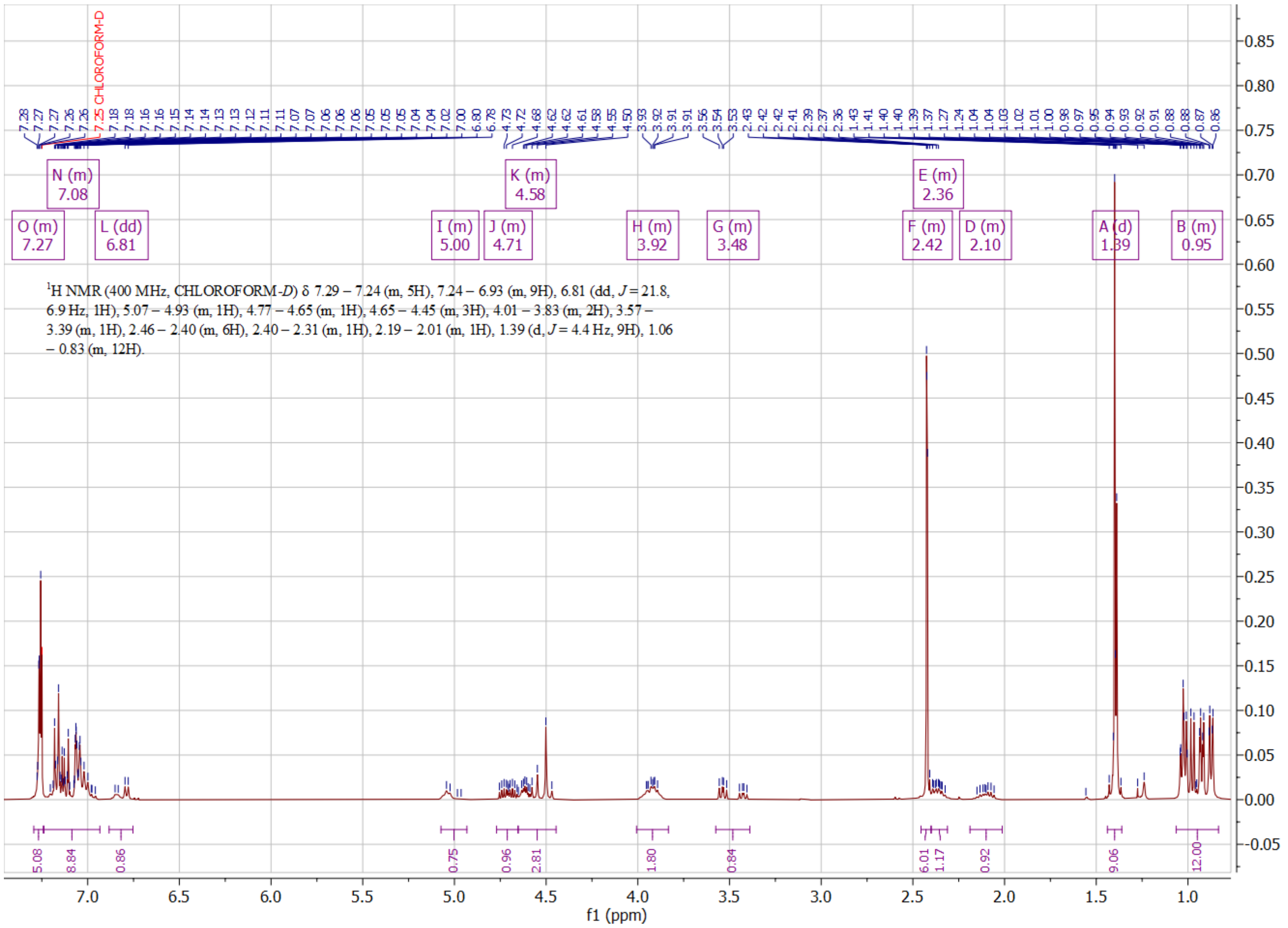
1H NMR (400 MHz, CHLOROFORM-D) δ 7.29 – 7.24 (m, 5H), 7.24 – 6.93 (m, 9H), 6.81 (dd, J = 21.8, 6.9 Hz, 1H), 5.07 – 4.93 (m, 1H), 4.77 – 4.65 (m, 1H), 4.65 – 4.45 (m, 3H), 4.01 – 3.83 (m, 2H), 3.57 – 3.39 (m, 1H), 2.46 – 2.40 (m, 6H), 2.40 – 2.31 (m, 1H), 2.19 – 2.01 (m, 1H), 1.39 (d, J = 4.4 Hz, 9H), 1.06 – 0.83 (m, 12H).
7.25 CHLOROFORM-D
O (m) 7.27
N (m) 7.08
L (dd) 6.81
I (m) 5.00
J (m) 4.71
K (m) 4.58
H (m) 3.92
G (m) 3.48
F (m) 2.42
E (m) 2.36
D (m) 2.10
A (d) 1.39
B (m) 0.95
f1 (ppm)

(25) Boc-L-Val-L-Thr(Bzl)-Val^{P}(OPh-4-SCH$_3$)$_2$

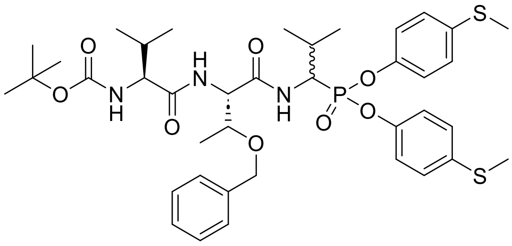

Name: *tert*-butyl ((2S)-1-(((2S,3S)-3-(benzyloxy)-1-((1-(bis(4-(methylthio)phenoxy)phosphoryl)-2-methylpropyl)amino)-1-oxobutan-2-yl)amino)-3-methyl-1-oxobutan-2-yl)carbamate

^{31}P NMR (162 MHz, Chloroform-d) δ 18.45 (s, 46%), 17.94 (s, 54%).

^{1}H NMR (400 MHz, Chloroform-d) δ 7.33 – 6.91 (m, 15H, 13xArH, 2xNH), 4.97 (d, *J* = 5.5 Hz, 1H, NH), 4.78 – 4.48 (m, 4H, CH$_2$, CHP, CH), 4.31 – 4.18 (m, 1H, CH), 4.01 – 3.92 (m, 1H, CH), 2.47 – 2.41 (m, 6H, 2xSCH$_3$), 2.41 – 2.30 (m, 1H, CH), 2.26 – 2.11 (m, 1H, CH), 1.40 (d, *J* = 3.8 Hz, 9H, Boc), 1.19 (dd, *J* = 10.4, 6.5 Hz, CH$_3$), 1.07 – 0.88 (m, 12H, 4xCH$_3$).

HRMS: $C_{39}H_{54}N_3O_8PS_2$ [M+Na]$^+$ calc. 810.2988 found 810.3001

HPLC: purity >99%; retention time 20.493 min

Chromatogram

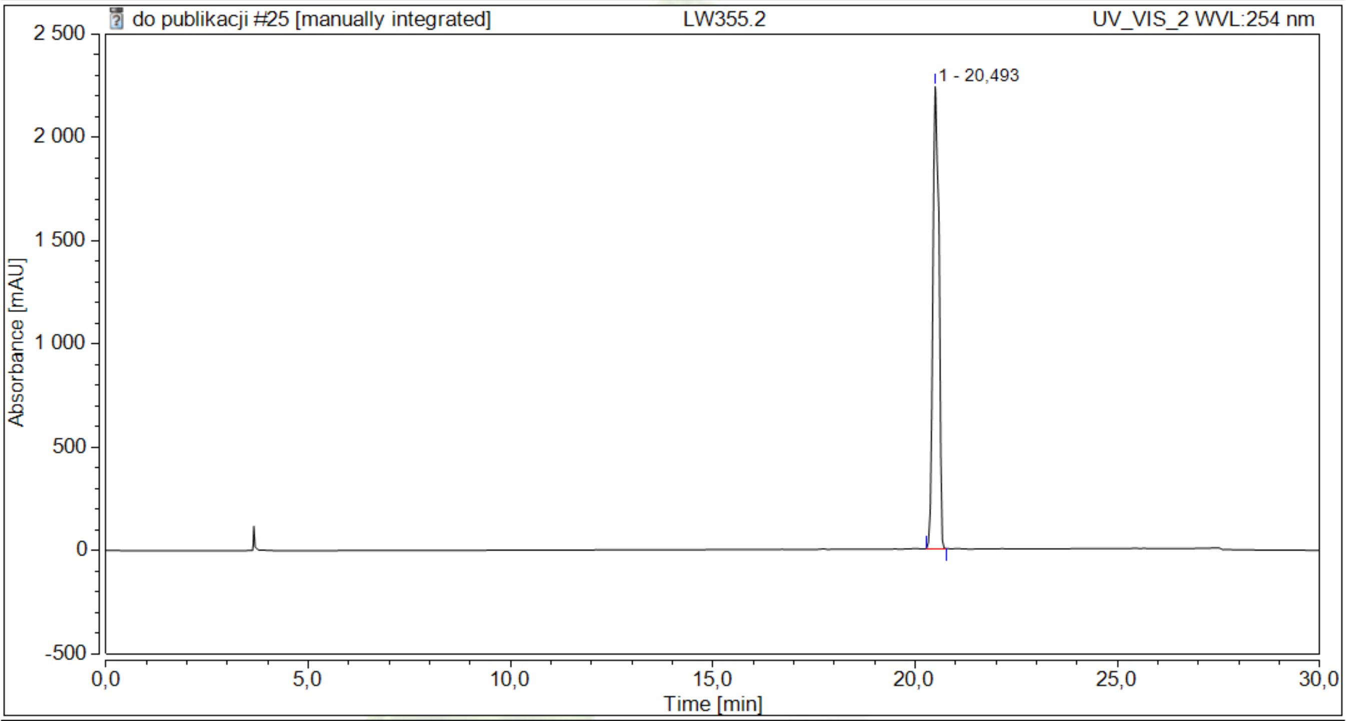

Integration Results

No.	Peak Name	Retention Time min	Area mAU*min	Height mAU	Relative Area %	Relative Height %	Amount n.a.
1		20,493	388,048	2235,403	100,00	100,00	n.a.
**Total:**			**388,048**	**2235,403**	**100,00**	**100,00**	

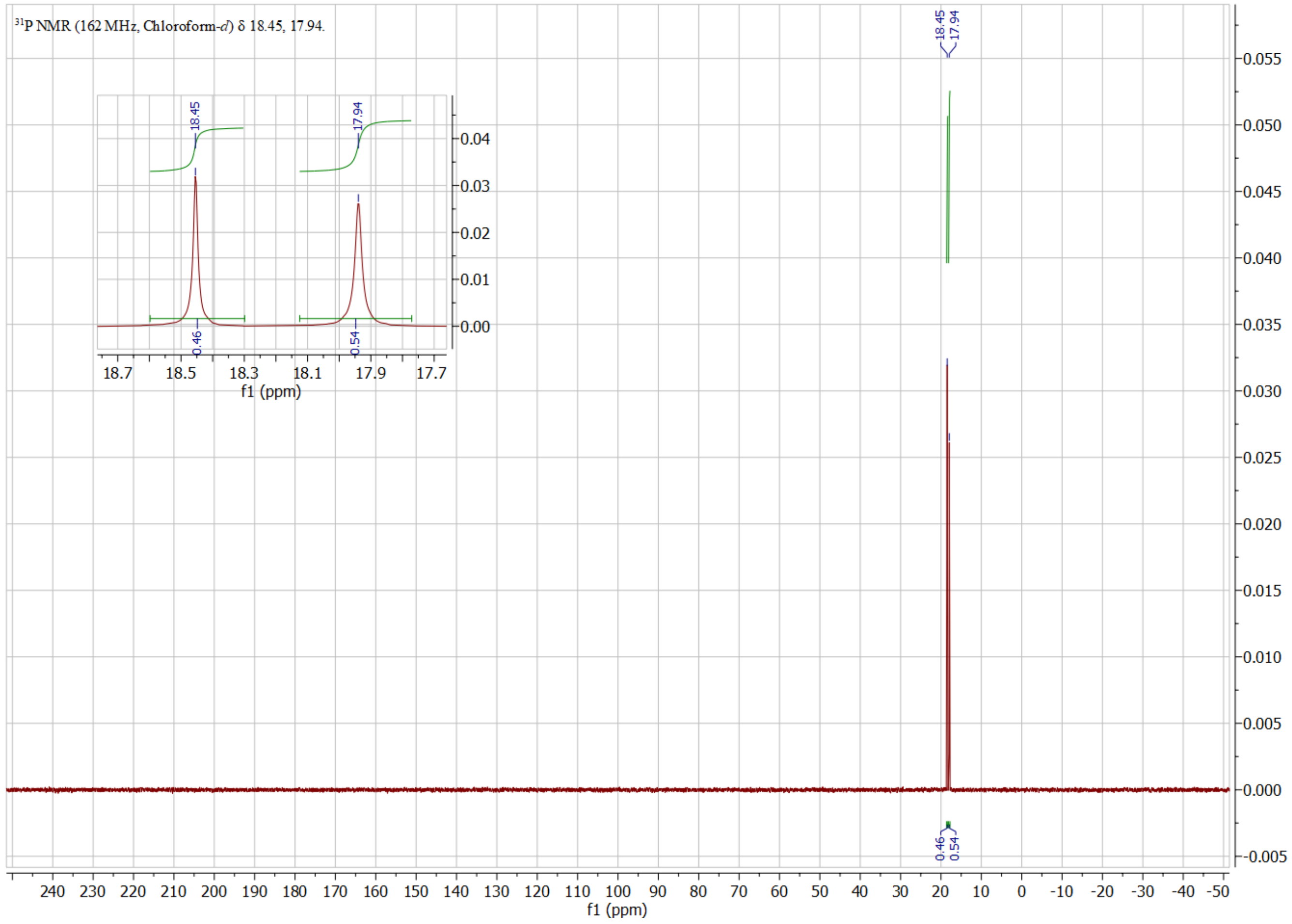

^{31}P NMR (162 MHz, Chloroform-*d*) δ 18.45, 17.94.

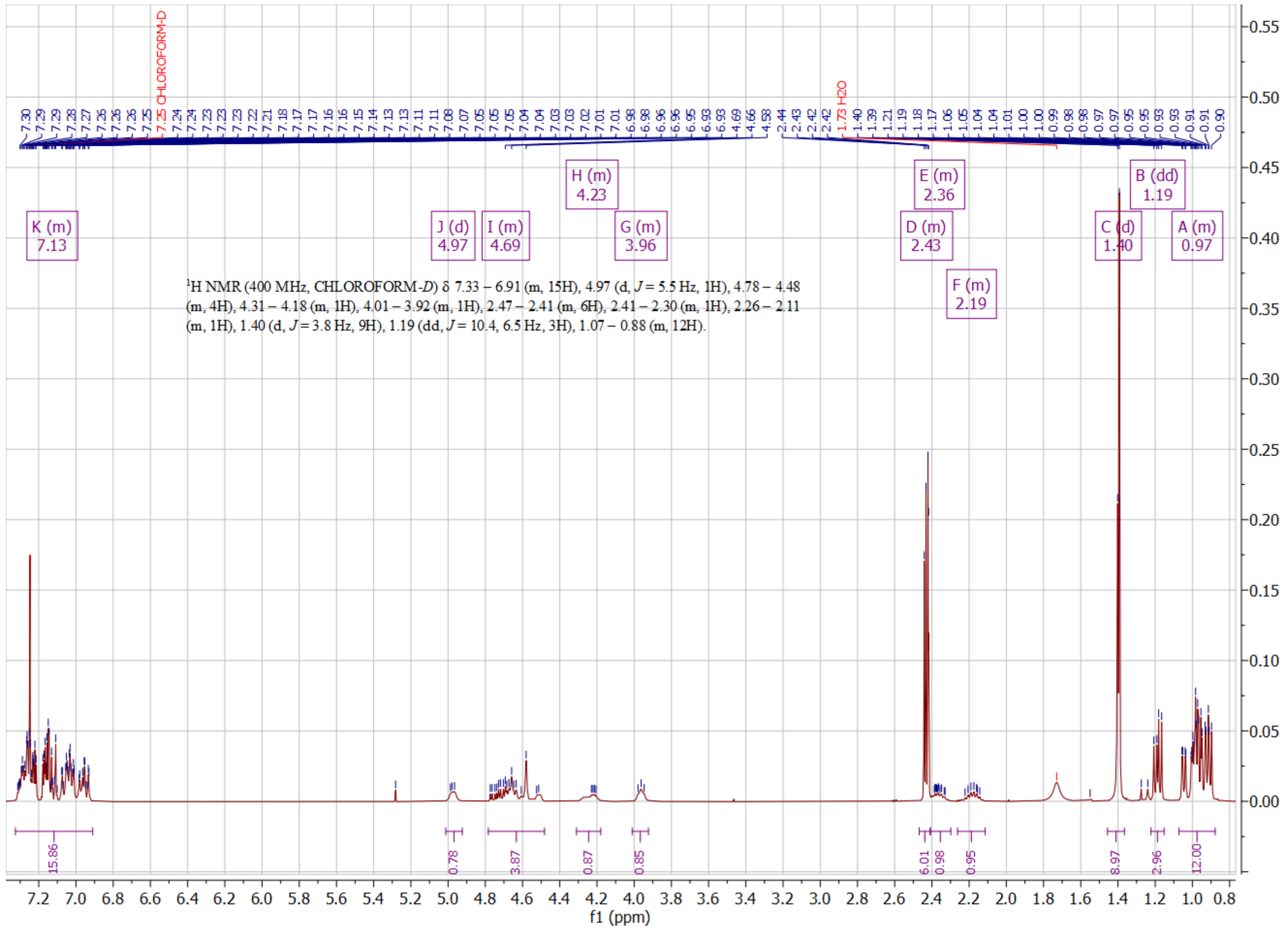

^{1}H NMR (400 MHz, CHLOROFORM-*D*) δ 7.33 – 6.91 (m, 15H), 4.97 (d, *J* = 5.5 Hz, 1H), 4.78 – 4.48 (m, 4H), 4.31 – 4.18 (m, 1H), 4.01 – 3.92 (m, 1H), 2.47 – 2.41 (m, 6H), 2.41 – 2.30 (m, 1H), 2.26 – 2.11 (m, 1H), 1.40 (d, *J* = 3.8 Hz, 9H), 1.19 (dd, *J* = 10.4, 6.5 Hz, 3H), 1.07 – 0.88 (m, 12H).

(26) Boc-L-Val-D-Thr(Bzl)- Val^{P}(OPh-4-SCH$_3$)$_2$

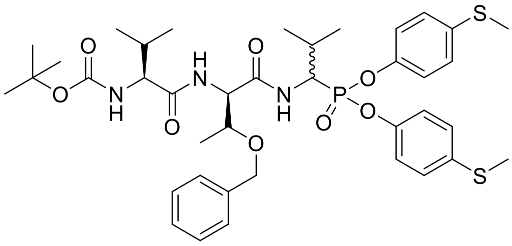

Name: *tert*-butyl ((2S)-1-(((2R,3R)-3-(benzyloxy)-1-((1-(bis(4-(methylthio)phenoxy)phosphoryl)-2-methylpropyl)amino)-1-oxobutan-2-yl)amino)-3-methyl-1-oxobutan-2-yl)carbamate
^{31}P NMR (162 MHz, Chloroform-d) δ 18.43 (s, 69%), 18.00 (s, 31%).
^{1}H NMR (400 MHz, Chloroform-d) δ 7.34 – 7.25 (m, 4H, 3xArH, NH), 7.24 – 6.85 (m, 11H, 10xArH, NH), 5.02 (d, J = 8.0 Hz, 1H, NH), 4.78 – 4.48 (m, 4H, CH$_2$, CHP, CH), 4.28 – 4.16 (m, 1H, CH), 4.05 – 3.92 (m, 1H, CH), 2.45 – 2.40 (m, 6H, 2xSCH$_3$), 2.40 – 2.30 (m, 1H, CH), 2.22 – 2.07 (m, 1H, CH), 1.40 (d, J = 1.9 Hz, 9H, Boc), 1.18 (dd, J = 7.3, 6.5 Hz, 3H, CH$_3$), 1.03 – 0.85 (m, 12H, 4xCH$_3$).
HRMS: $C_{39}H_{54}N_3O_8PS_2$ [M+Na]$^+$ calc. 810.2988 found 810.3005
HPLC: purity >99%; retention time 20.473 and 20.573 min

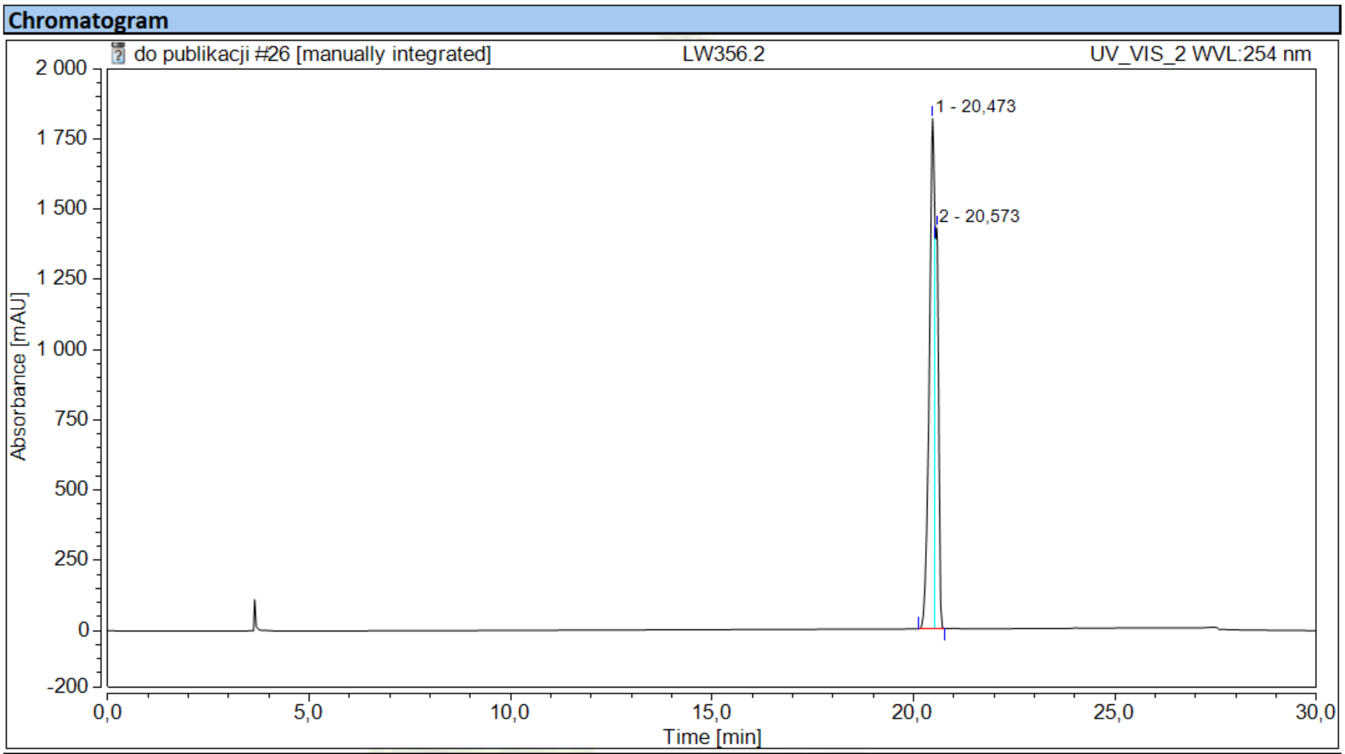

Integration Results

No.	Peak Name	Retention Time min	Area mAU*min	Height mAU	Relative Area %	Relative Height %	Amount n.a.
1		20,473	280,574	1814,893	68,58	56,01	n.a.
2		20,573	128,523	1425,596	31,42	43,99	n.a.
**Total:**			**409,097**	**3240,489**	**100,00**	**100,00**	

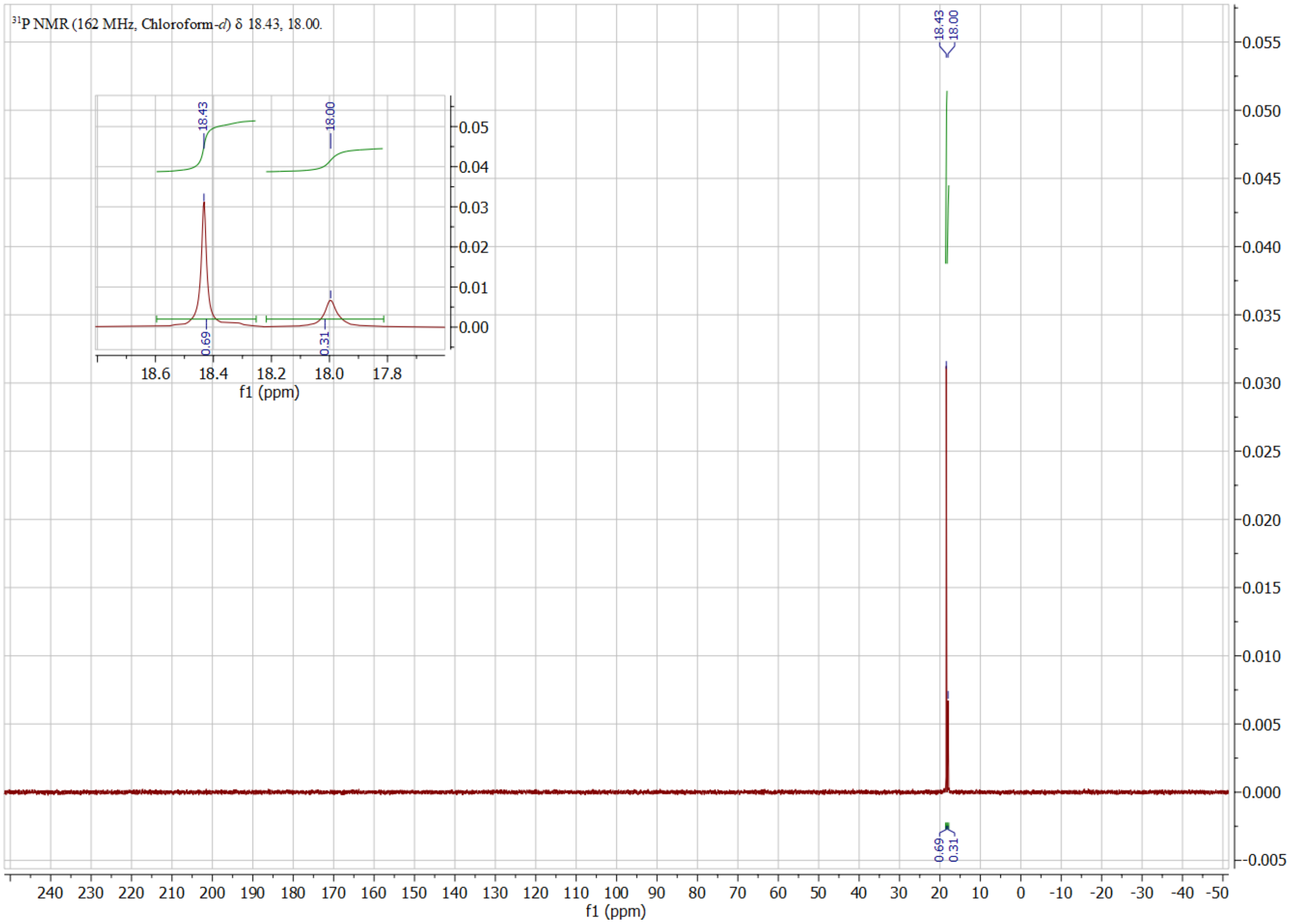

31P NMR (162 MHz, Chloroform-d) δ 18.43, 18.00.
f1 (ppm)

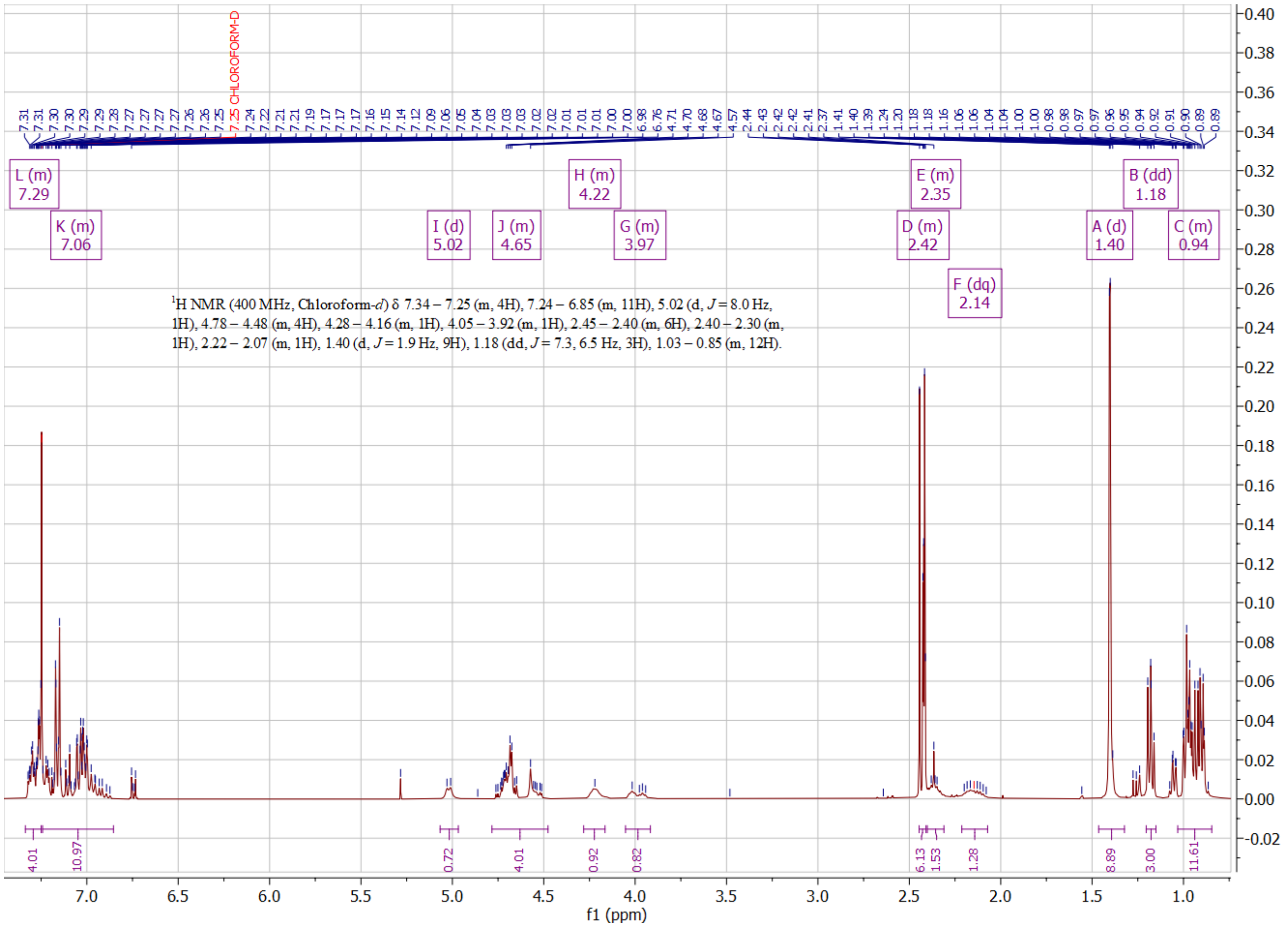

1H NMR (400 MHz, Chloroform-d) δ 7.34 – 7.25 (m, 4H), 7.24 – 6.85 (m, 11H), 5.02 (d, J = 8.0 Hz, 1H), 4.78 – 4.48 (m, 4H), 4.28 – 4.16 (m, 1H), 4.05 – 3.92 (m, 1H), 2.45 – 2.40 (m, 6H), 2.40 – 2.30 (m, 1H), 2.22 – 2.07 (m, 1H), 1.40 (d, J = 1.9 Hz, 9H), 1.18 (dd, J = 7.3, 6.5 Hz, 3H), 1.03 – 0.85 (m, 12H).
7.25 CHLOROFORM-D
f1 (ppm)

(27) Boc-L-Val-L-Pro-Val^{P}(OPh-4-SCH$_3$)$_2$

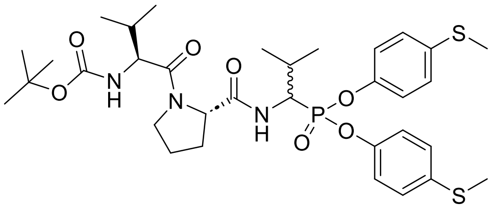

Reference compound, analytical data from our previous publication: Winiarski, Ł.; Oleksyszyn, J.; Sieńczyk, M. Human neutrophil elastase phosphonic inhibitors with improved potency of action. *J. Med. Chem.* **2012**, *55*, 6541–6553.

Name: *tert*-butyl ((2S)-1-((2S)-2-((1-(bis(4-(methylthio)phenoxy)phosphoryl)-2-methylpropyl)carbamoyl)pyrrolidin-1-yl)-3-methyl-1-oxobutan-2-yl)carbamate
^{31}P NMR (121 MHz, CDCl3) δ 18.15 (s, 52%), 18.48 (s, 48%).
^{1}H NMR (300 MHz, CDCl3) δ 7.03–7.23 (m, 8H), 5.30 (dd, J = 61.7, 9.3 Hz, 1H), 4.49– 4.78 (m, 2H), 4.20–4.34 (m, 1H), 3.43–3.80 (m, 2H), 2.44 (d, J = 2.7 Hz, 6H), 2.04–2.41 (m, 2H), 1.74–2.03 (m, 4H), 1.42 (d, J = 2.0 Hz, 9H), 0.81–1.14 (m, 12H).
HRMS: $C_{33}H_{48}N_3O_7PS_2$ [M+H]$^+$ calc. 694.2750 found 694.2738

HPLC: purity >99%; retention time 18.817 and 19.053 min

Chromatogram

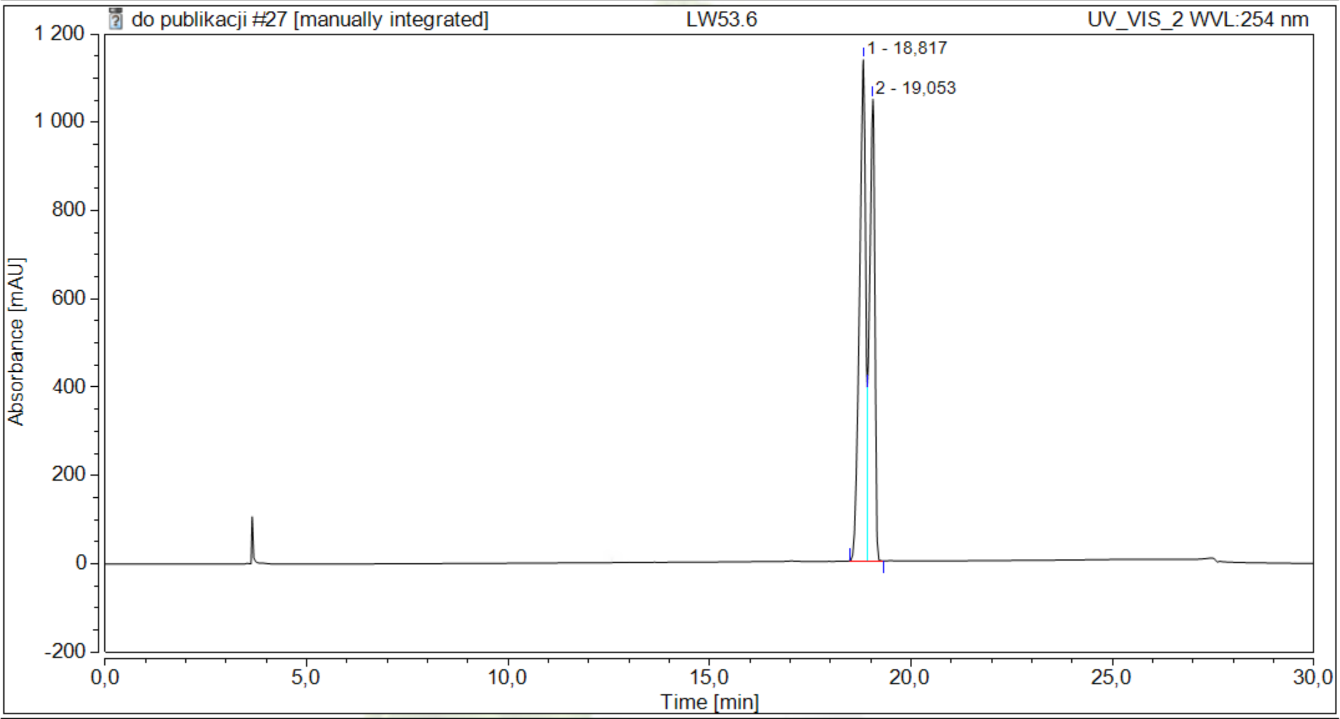

Integration Results

No.	Peak Name	Retention Time min	Area mAU*min	Height mAU	Relative Area %	Relative Height %	Amount n.a.
1		18,817	199,989	1135,211	55,62	52,05	n.a.
2		19,053	159,562	1045,952	44,38	47,95	n.a.
**Total:**			**359,551**	**2181,163**	**100,00**	**100,00**	

(28) Boc-L-Val-D-Pro-Val^P(OPh-4-SCH$_3$)$_2$

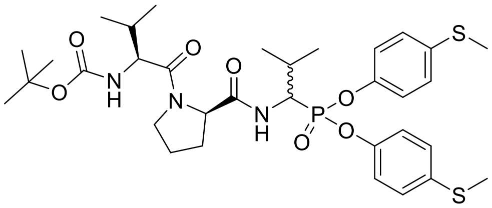

Name: *tert*-butyl ((2S)-1-((2R)-2-((1-(bis(4-(methylthio)phenoxy)phosphoryl)-2-methylpropyl)carbamoyl)pyrrolidin-1-yl)-3-methyl-1-oxobutan-2-yl)carbamate

^{31}P NMR (162 MHz, Chloroform-d) δ 18.42 (s, 58%), 18.19 (s, 42%).

^{1}H NMR (400 MHz, Chloroform-d) δ 7.57 (dd, *J* = 122.0, 9.7 Hz, 1H, NH), 7.22 – 7.00 (m, 8H, 8xArH), 5.23 (t, *J* = 8.0 Hz, 1H, NH), 4.74 – 4.53 (m, 2H, CHP, CH), 4.31 – 4.19 (m, 1H, CH), 3.90 – 3.76 (m, 1H, CH$_2$), 3.58 – 3.44 (m, 1H, CH$_2$), 2.43 (d, *J* = 4.6 Hz, 6H, 3xSCH$_3$), 2.42 – 1.73 (m, 6H, 2xCH$_2$, 2xCH), 1.45 – 1.30 (m, 9H, Boc), 1.20 – 0.83 (m, 12H, 4xCH$_3$).

HRMS: $C_{33}H_{48}N_3O_7PS_2$ [M+Na]$^+$ calc. 716.2569 found 716.2552

HPLC: purity >99%; retention time 18.967 min

Chromatogram

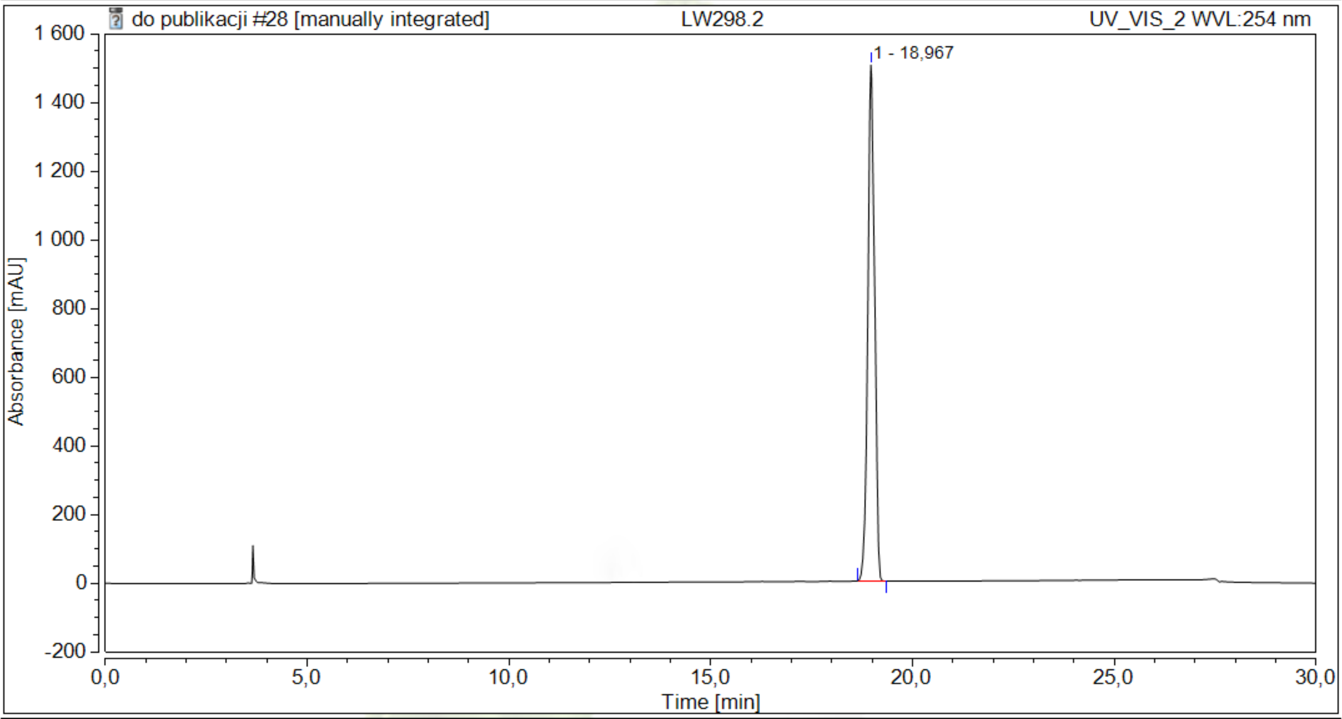

Integration Results

No.	Peak Name	Retention Time min	Area mAU*min	Height mAU	Relative Area %	Relative Height %	Amount n.a.
1		18,967	292,341	1502,416	100,00	100,00	n.a.
**Total:**			**292,341**	**1502,416**	**100,00**	**100,00**	

31P NMR (162 MHz, Chloroform-*d*) δ 18.42, 18.19.

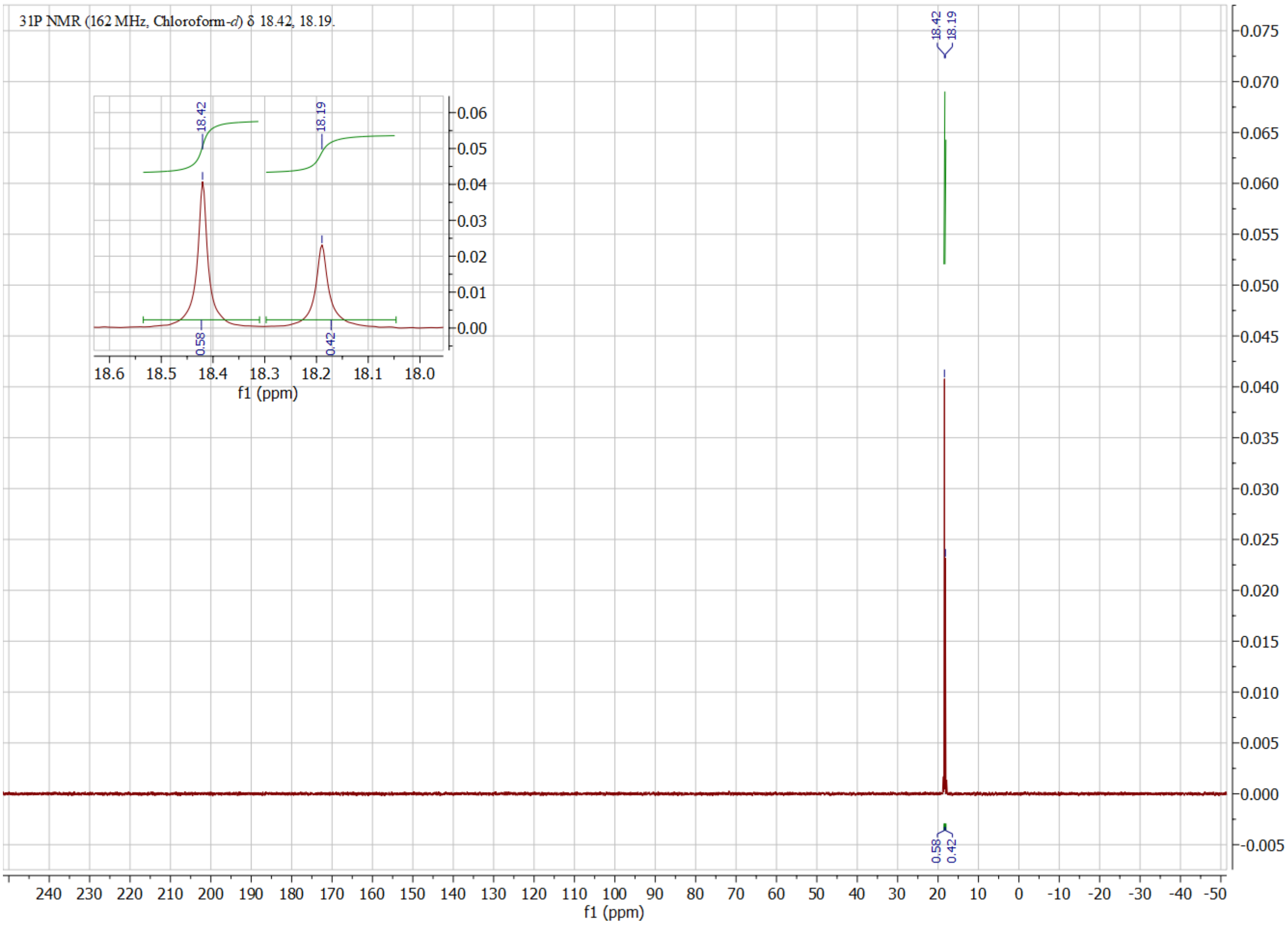

^{1}H NMR (400 MHz, CHLOROFORM-*D*) δ 7.57 (dd, *J* = 122.0, 9.7 Hz, 1H), 7.22 – 7.00 (m, 8H), 5.23 (t, *J* = 8.0 Hz, 1H), 4.74 – 4.53 (m, 2H), 4.31 – 4.19 (m, 1H), 3.90 – 3.76 (m, 1H), 3.58 – 3.44 (m, 1H), 2.43 (d, *J* = 4.6 Hz, 6H), 2.42 – 1.73 (m, 6H), 1.45 – 1.30 (m, 9H), 1.20 – 0.83 (m, 12H).

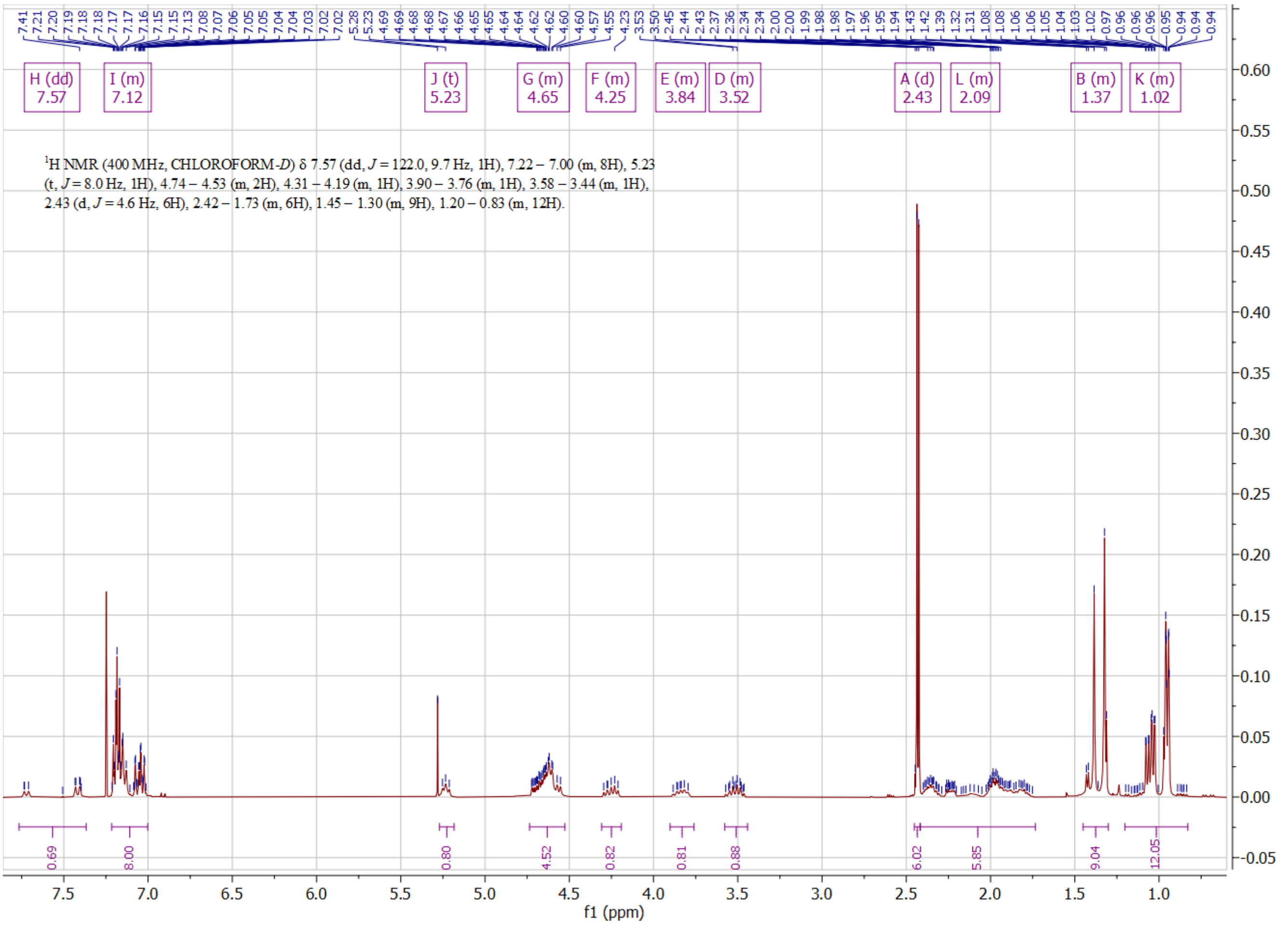

(29) Boc-L-Val-L-Oic-Val^{P}(OPh-4-SCH$_3$)$_2$

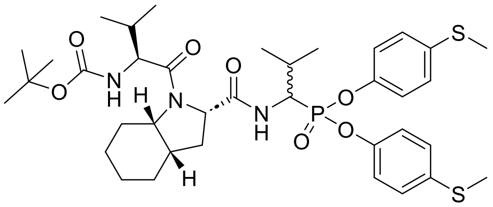

Name: *tert*-butyl ((2S)-1-((2S,3aS,7aS)-2-((1-(bis(4-(methylthio)phenoxy)phosphoryl)-2-methylpropyl)carbamoyl)octahydro-1H-indol-1-yl)-3-methyl-1-oxobutan-2-yl)carbamate
^{31}P NMR (162 MHz, Chloroform-d) δ 18.96(s, 44%), 18.27 (s, 56%). 1H NMR
^{1}H NMR (400 MHz, Chloroform-d) δ 7.41 (dd, *J* = 66.0, 10.1 Hz, 1H, NH), 7.27 – 6.99 (m, 8H, 8xArH), 5.16 (dd, *J* = 37.5, 9.9 Hz, 1H, NH), 4.79 – 4.59 (m, 1H, CHP), 4.51 – 4.10 (m, 2H, 2xCH), 2.48 – 2.40 (m, 6H, 2xSCH$_3$), 2.40 – 2.18 (m, 3H, CH$_2$, CH), 2.09 – 1.84 (m, 2H, 2xCH), 1.84 – 1.55 (m, 5H, 2xCH$_2$, CH), 1.55 – 1.44 (m, 2H, CH$_2$), 1.42 (d, *J* = 5.6 Hz, 9H, Boc), 1.30 – 1.13 (m, 2H, CH$_2$), 1.13 – 0.97 (m, 6H, 2xCH$_3$), 0.97 – 0.78 (m, 6H, 2xCH$_3$).
HRMS: $C_{37}H_{54}N_3O_7PS_2$ [M+Na]$^+$ calc. 770.3038 found 770.3038
HPLC: purity >99%; retention time 21.010 and 21.287 min

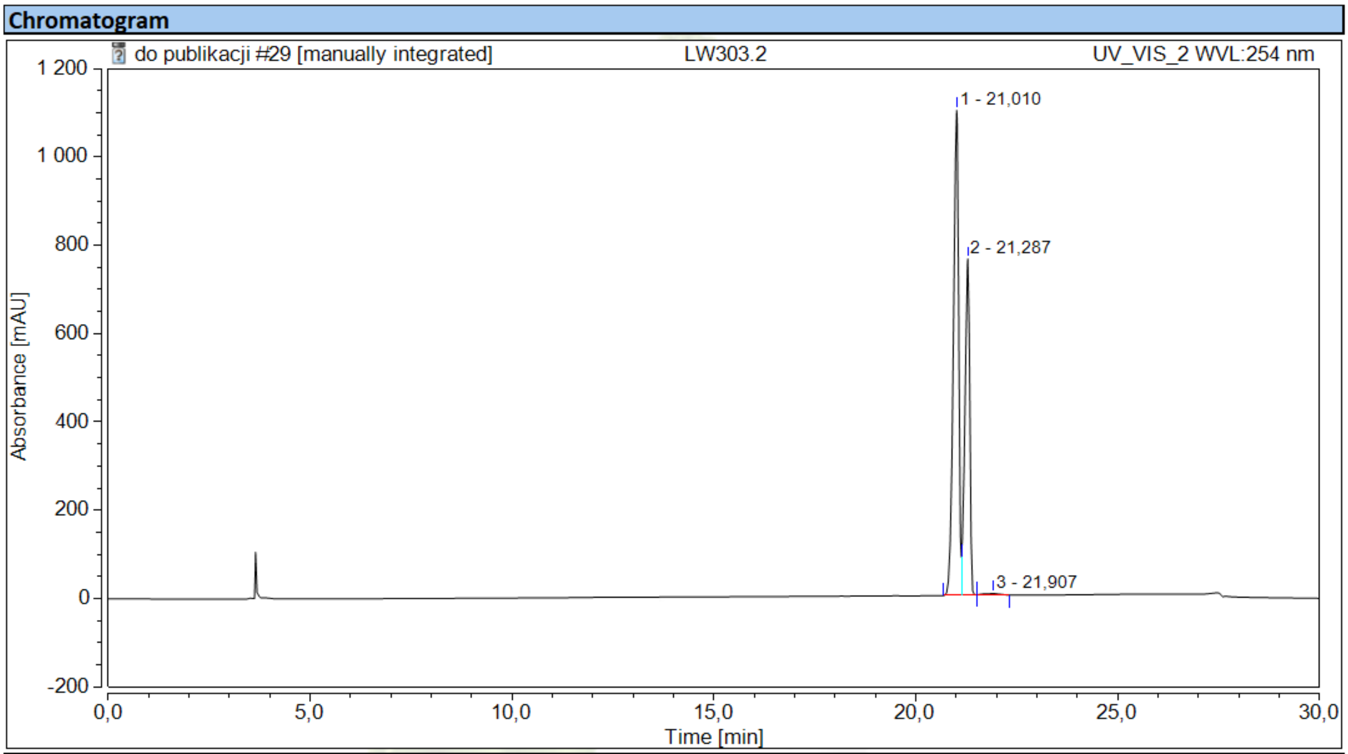

Integration Results

No.	Peak Name	Retention Time min	Area mAU*min	Height mAU	Relative Area %	Relative Height %	Amount n.a.
1		21,010	166,256	1098,398	60,69	58,93	n.a.
2		21,287	105,767	761,811	38,61	40,87	n.a.
3		21,907	1,931	3,737	0,70	0,20	n.a.
**Total:**			**273,954**	**1863,946**	**100,00**	**100,00**	

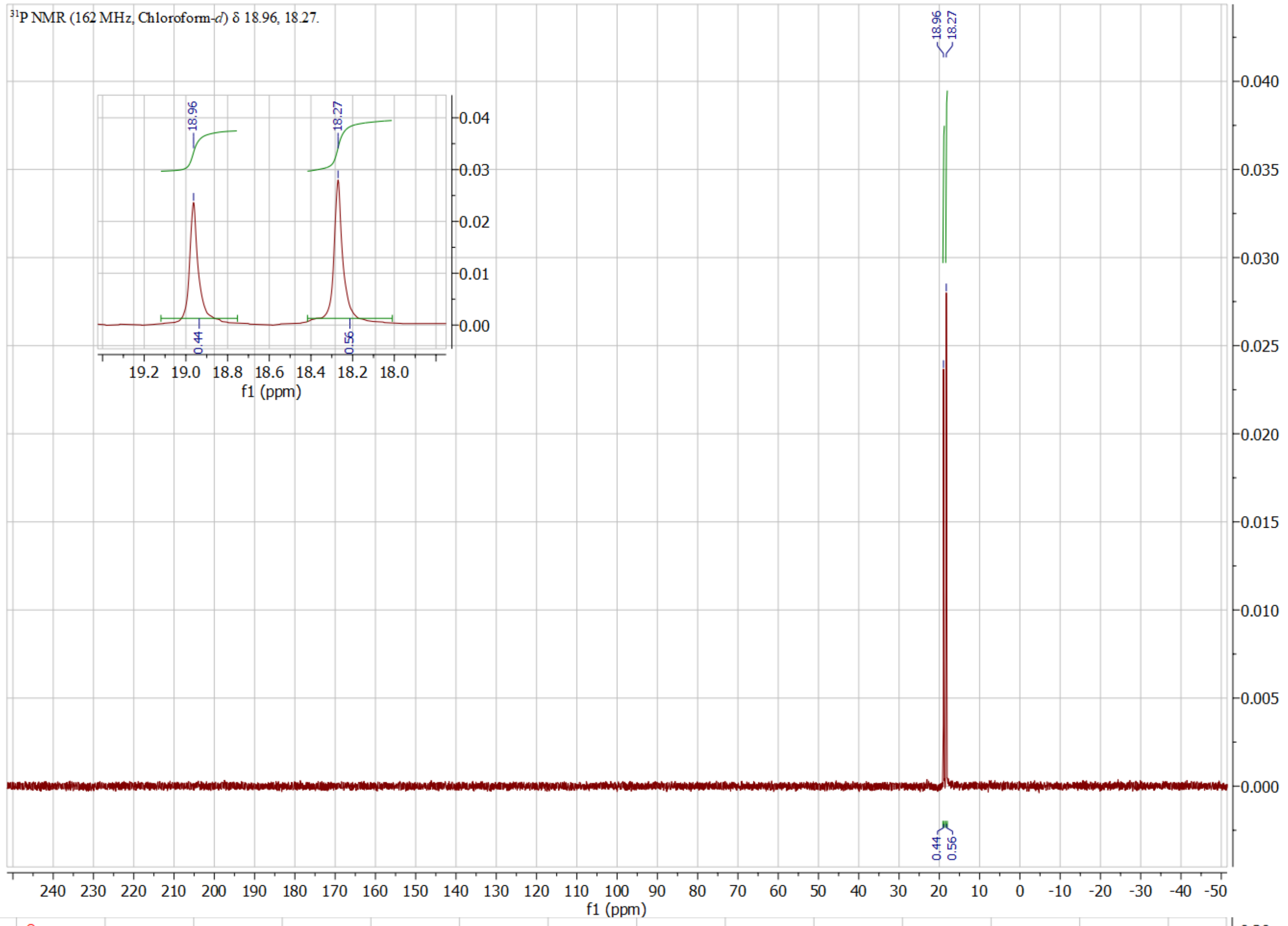
31P NMR (162 MHz, Chloroform-d) δ 18.96, 18.27.
18.96
18.27
0.44
0.56
f1 (ppm)

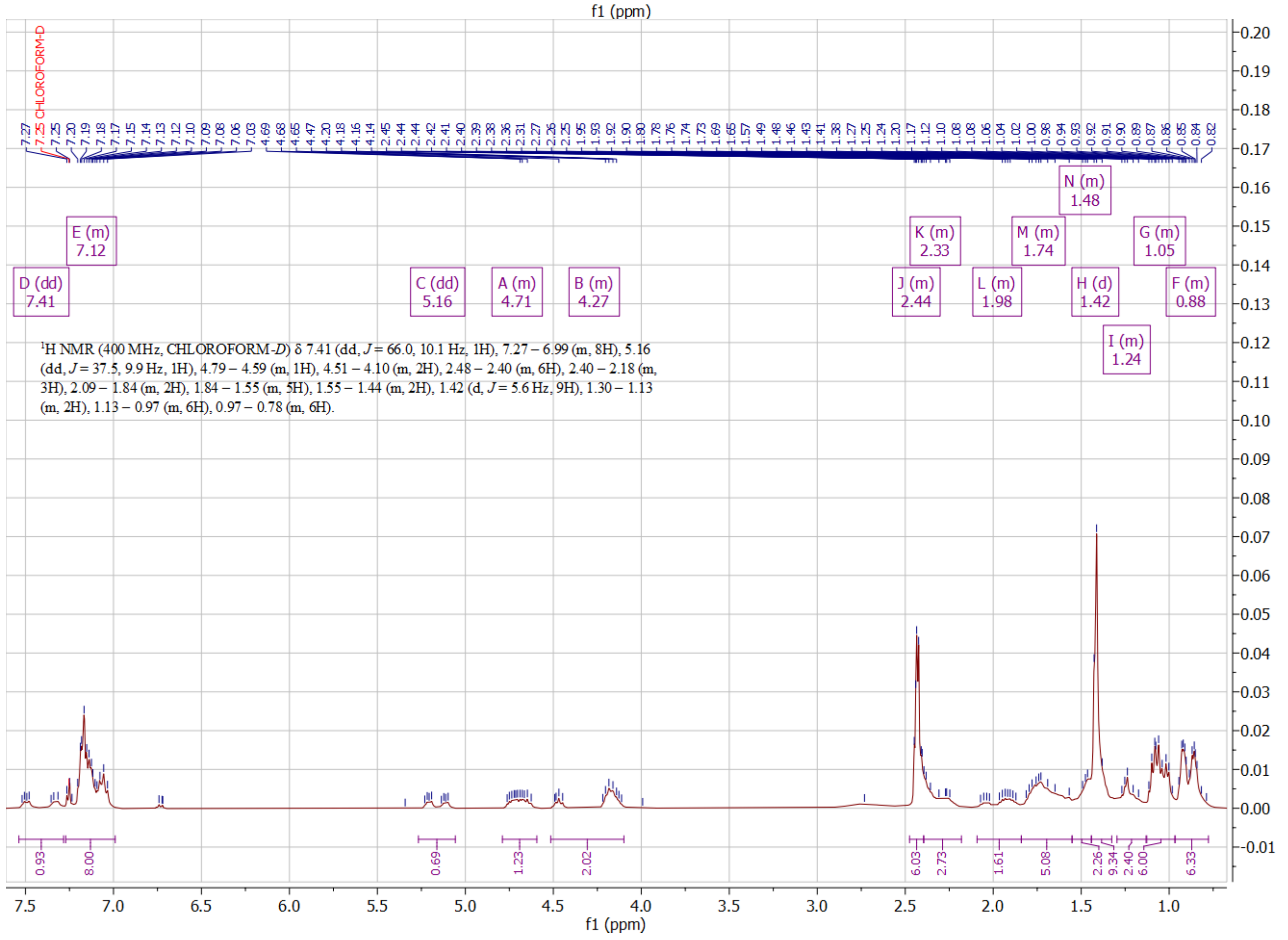
CHLOROFORM-D
D (dd) 7.41
E (m) 7.12
C (dd) 5.16
A (m) 4.71
B (m) 4.27
J (m) 2.44
K (m) 2.33
L (m) 1.98
M (m) 1.74
N (m) 1.48
H (d) 1.42
I (m) 1.24
G (m) 1.05
F (m) 0.88
1H NMR (400 MHz, CHLOROFORM-D) δ 7.41 (dd, J = 66.0, 10.1 Hz, 1H), 7.27 – 6.99 (m, 8H), 5.16 (dd, J = 37.5, 9.9 Hz, 1H), 4.79 – 4.59 (m, 1H), 4.51 – 4.10 (m, 2H), 2.48 – 2.40 (m, 6H), 2.40 – 2.18 (m, 3H), 2.09 – 1.84 (m, 2H), 1.84 – 1.55 (m, 5H), 1.55 – 1.44 (m, 2H), 1.42 (d, J = 5.6 Hz, 9H), 1.30 – 1.13 (m, 2H), 1.13 – 0.97 (m, 6H), 0.97 – 0.78 (m, 6H).
f1 (ppm)

(30) Boc-L-Val-L-Pip-Val^{P}(OPh-4-SCH$_3$)$_2$

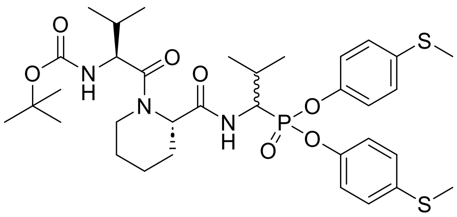

Name: *tert*-butyl ((2S)-1-((2S)-2-((1-(bis(4-(methylthio)phenoxy)phosphoryl)-2-methylpropyl)carbamoyl)piperidin-1-yl)-3-methyl-1-oxobutan-2-yl)carbamate

^{31}P NMR (162 MHz, Chloroform-d) δ 18.97 (s, 19%), 18.69 (s, 39%), 18.11 (s, 42%).

^{1}H NMR (400 MHz, Chloroform-d) δ 7.24 – 6.94 (m, 8H, 8xArH), 6.40 – 6.20 (m, 1H, NH), 5.39 – 5.05 (m, 2H, NH, CH), 4.81 – 4.50 (m, 2H, CH$_2$), 4.23 – 3.74 (m, 1H, CHP), 3.29 – 2.50 (m, 1H, CH), 2.49 – 2.41 (m, 6H, 2xSCH$_3$), 2.41 – 2.10 (m, 2H, 2xCH), 2.03 – 1.81 (m, 2H, CH$_2$), 1.68 – 1.44 (m, 4H, 2xCH$_2$), 1.44 – 1.28 (m, 9H, Boc), 1.11 – 0.77 (m, 12H, 4xCH$_3$).

HRMS: $C_{34}H_{50}N_3O_7PS_2$ [M+Na]$^+$ calc. 730.2725 found 730.2708

HPLC: purity >99%; retention time 19.987 and 20.133 min

Chromatogram

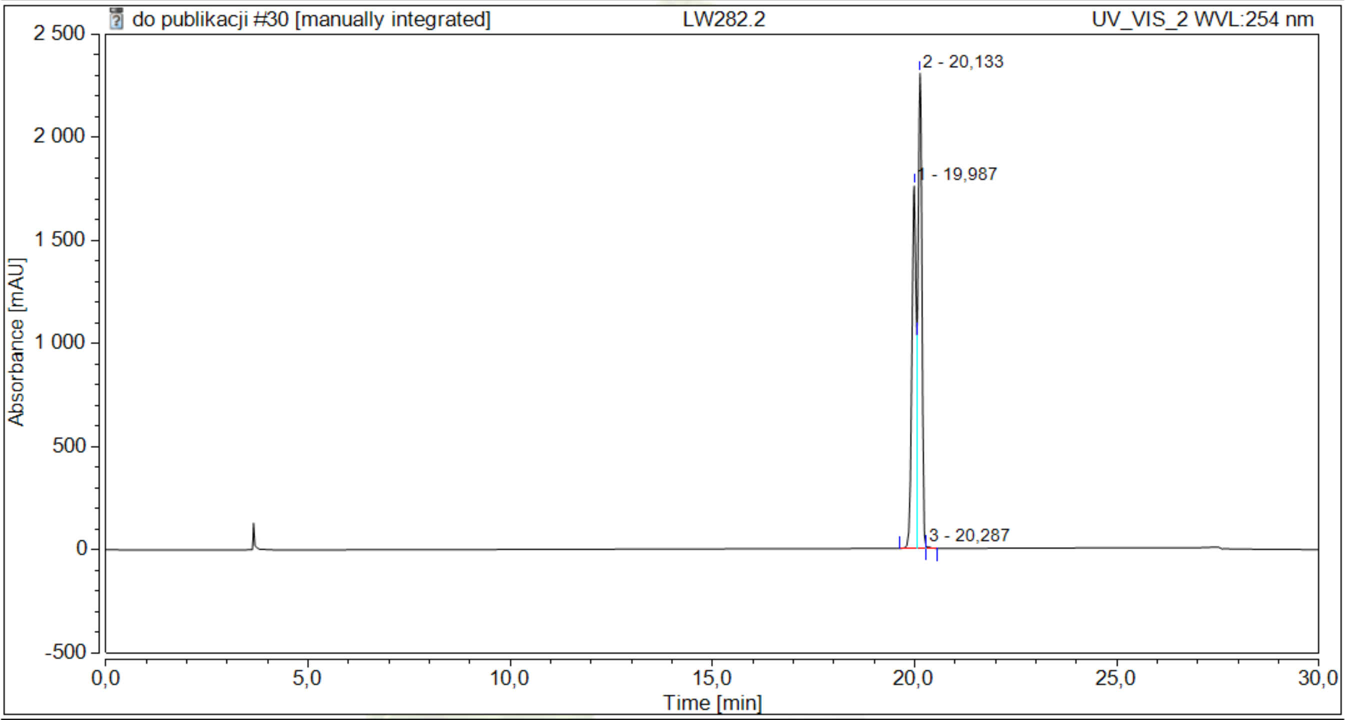

Integration Results

No.	Peak Name	Retention Time min	Area mAU*min	Height mAU	Relative Area %	Relative Height %	Amount n.a.
1		19,987	208,225	1756,423	44,81	43,20	n.a.
2		20,133	255,759	2302,715	55,04	56,64	n.a.
3		20,287	0,720	6,307	0,15	0,16	n.a.
**Total:**			**464,704**	**4065,445**	**100,00**	**100,00**	

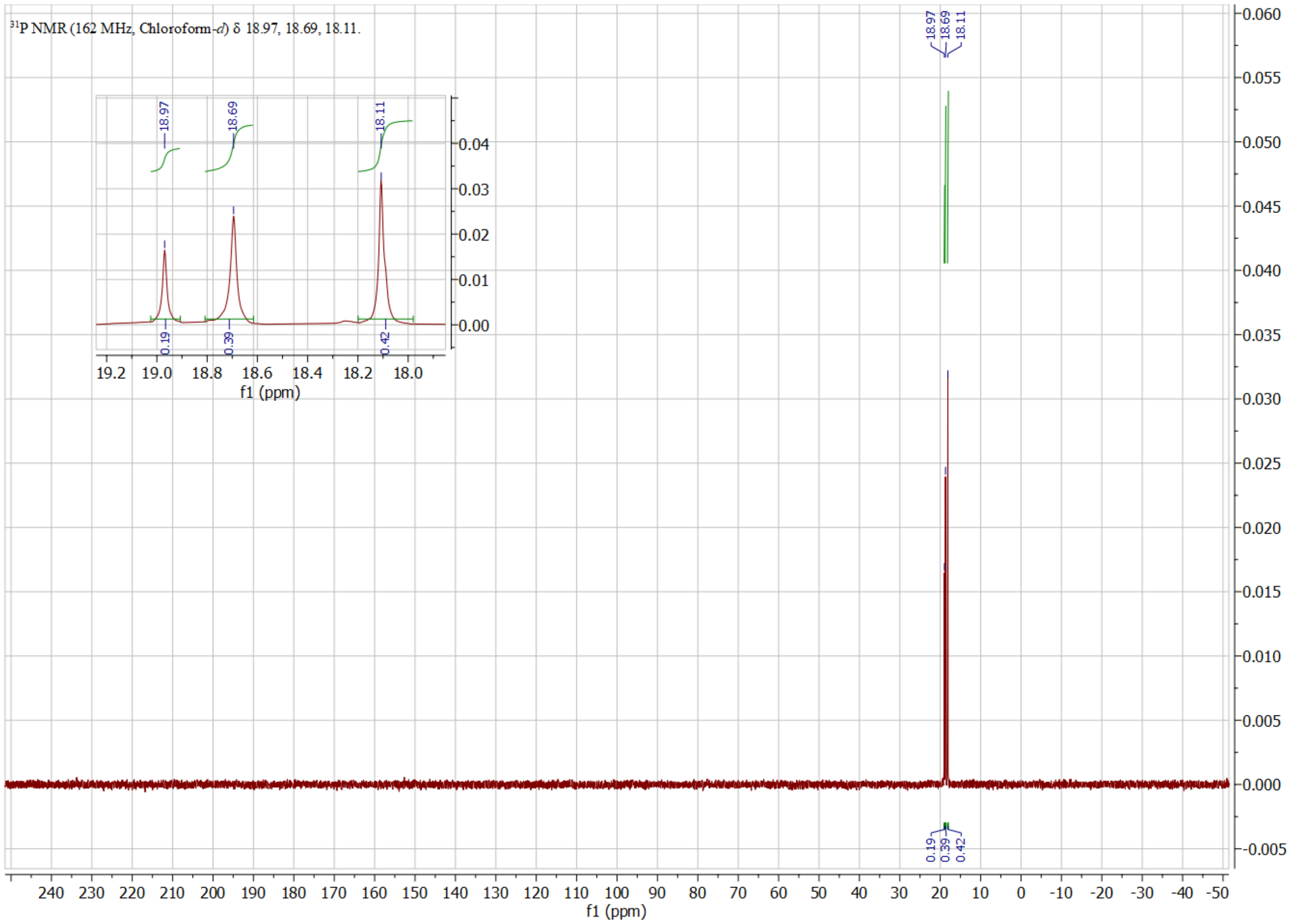

^{31}P NMR (162 MHz, Chloroform-*d*) δ 18.97, 18.69, 18.11.
f1 (ppm)

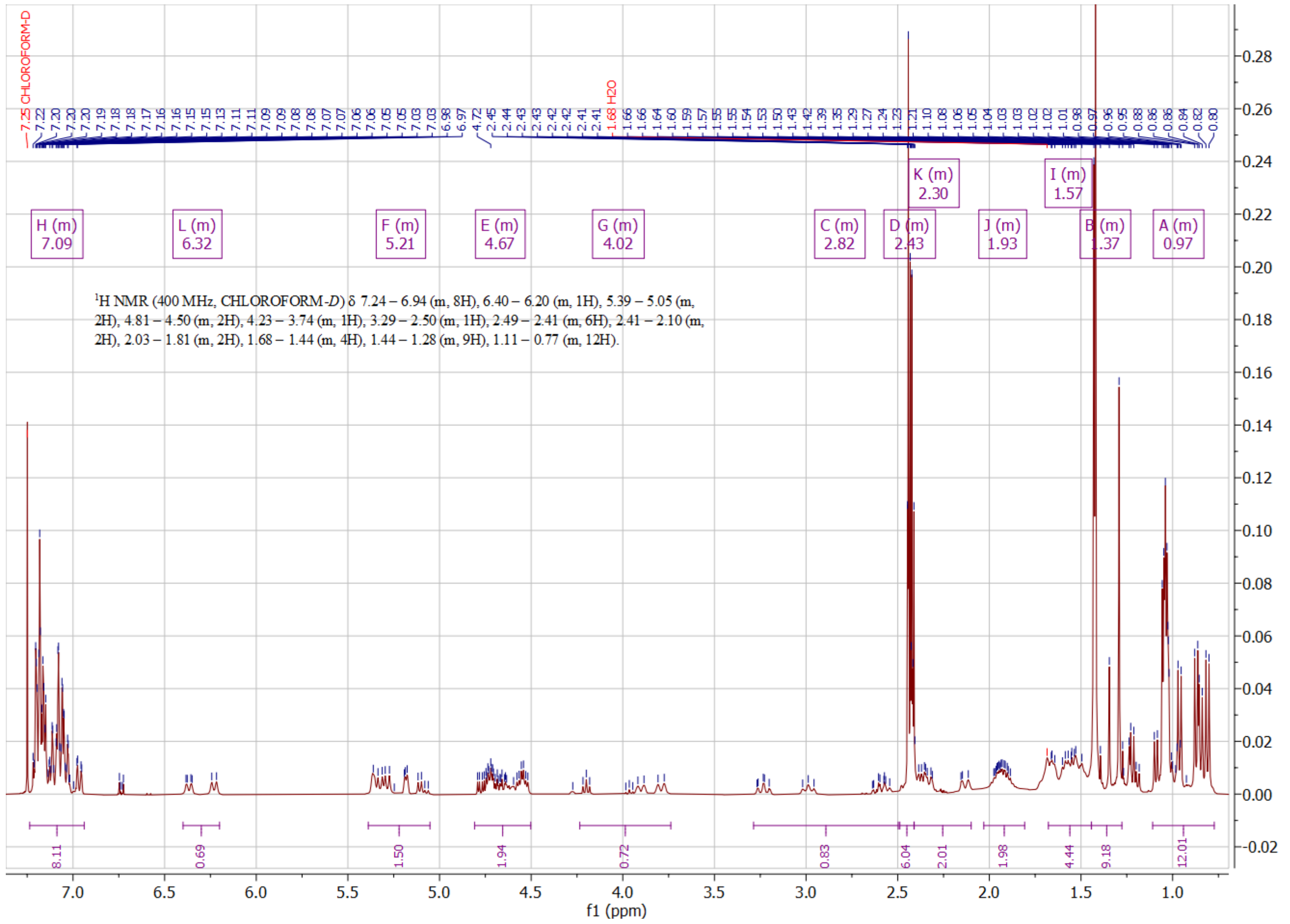

^{1}H NMR (400 MHz, CHLOROFORM-*D*) δ 7.24 – 6.94 (m, 8H), 6.40 – 6.20 (m, 1H), 5.39 – 5.05 (m, 2H), 4.81 – 4.50 (m, 2H), 4.23 – 3.74 (m, 1H), 3.29 – 2.50 (m, 1H), 2.49 – 2.41 (m, 6H), 2.41 – 2.10 (m, 2H), 2.03 – 1.81 (m, 2H), 1.68 – 1.44 (m, 4H), 1.44 – 1.28 (m, 9H), 1.11 – 0.77 (m, 12H).
f1 (ppm)

(31) Boc-L-Val-D-Pip-Val^P(OPh-4-SCH$_3$)$_2$

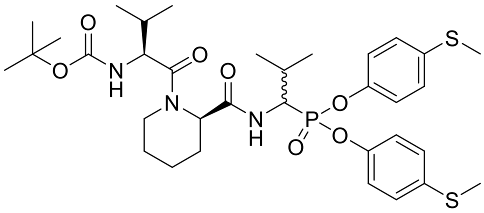

Name: *tert*-butyl ((2S)-1-((2R)-2-((1-(bis(4-(methylthio)phenoxy)phosphoryl)-2-methylpropyl)carbamoyl)piperidin-1-yl)-3-methyl-1-oxobutan-2-yl)carbamate
^{31}P NMR (162 MHz, Chloroform-d) δ 18.66 (s, 53%), 18.10 (s, 47%).
^{1}H NMR (400 MHz, Chloroform-d) δ 7.24 – 7.00 (m, 8H, 8xArH), 6.91 (td, *J* = 9.6, 9.6 Hz, 1H, NH), 5.36 – 5.10 (m, 2H, NH, CH), 4.76 – 4.62 (m, 1H, CHP), 4.53 – 4.38 (m, 1H, CH), 4.01 – 3.80 (m, 1H, CH$_2$), 3.18 – 3.04 (m, 1H, CH$_2$), 2.49 – 2.42 (m, 6H, 2xSCH$_3$), 2.42 – 2.17 (m, 2H, 2xCH), 2.02 – 1.66 (m, 2H, CH$_2$), 1.66 – 1.41 (m, 4H, 2xCH$_2$), 1.34 (d, *J* = 9.6 Hz, 9H, Boc), 1.14 – 0.82 (m, 12H, 4xCH$_3$).
HRMS: $C_{34}H_{50}N_3O_7PS_2$ [M+Na]$^+$ calc. 730.2725 found 730.2713
HPLC: purity >99%; retention time 20.190 and 20.340 min

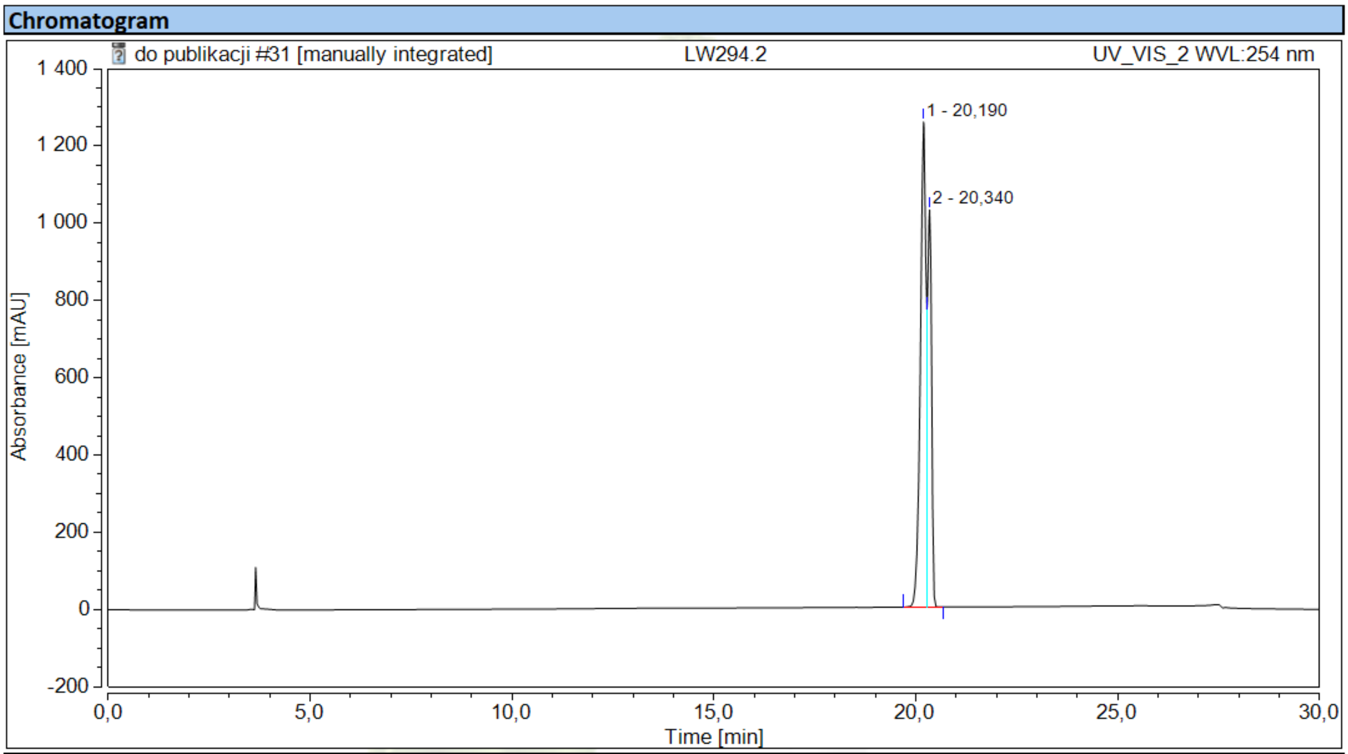

Integration Results

No.	Peak Name	Retention Time min	Area mAU*min	Height mAU	Relative Area %	Relative Height %	Amount n.a.
1		20,190	199,441	1256,077	62,39	54,98	n.a.
2		20,340	120,237	1028,374	37,61	45,02	n.a.
**Total:**			**319,679**	**2284,451**	**100,00**	**100,00**	

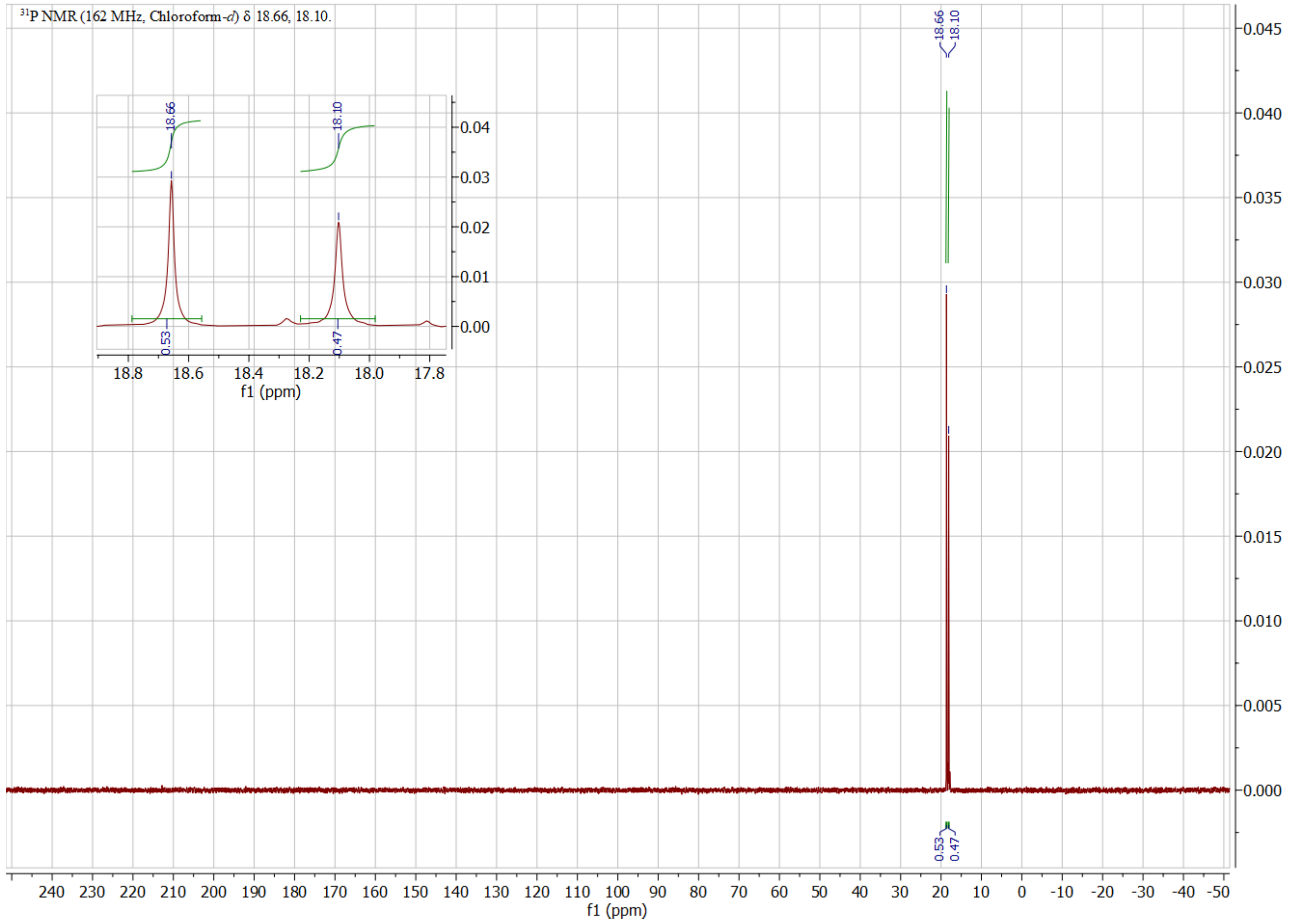

^{31}P NMR (162 MHz, Chloroform-*d*) δ 18.66, 18.10.
f1 (ppm)

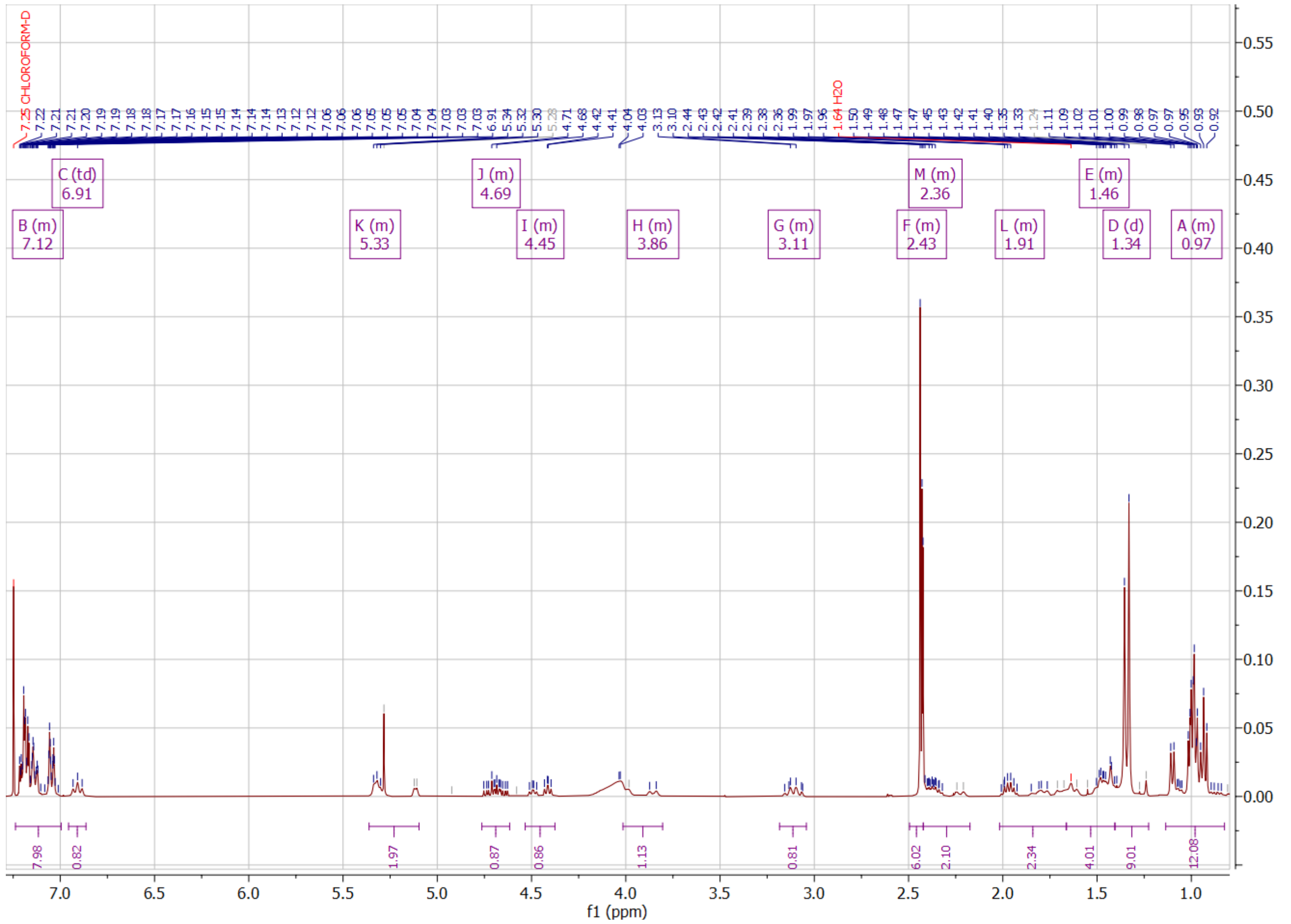

CHLOROFORM-D
H2O
B (m) 7.12
C (td) 6.91
K (m) 5.33
J (m) 4.69
I (m) 4.45
H (m) 3.86
G (m) 3.11
F (m) 2.43
M (m) 2.36
L (m) 1.91
E (m) 1.46
D (d) 1.34
A (m) 0.97
f1 (ppm)

(32) Boc-L-Val-L-Tic-Val^P(OPh-4-SCH$_3$)$_2$

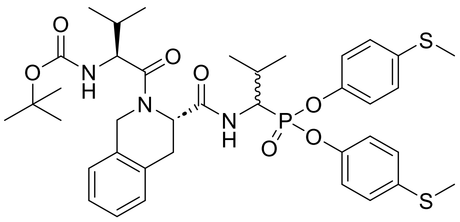

Name: *tert*-butyl ((2S)-1-((3S)-3-((1-(bis(4-(methylthio)phenoxy)phosphoryl)-2-methylpropyl)carbamoyl)-3,4-dihydroisoquinolin-2(1H)-yl)-3-methyl-1-oxobutan-2-yl)carbamate

^{31}P NMR (162 MHz, Chloroform-d) δ 19.08 (s, 21%), 18.64 (s, 25%), 18.21 (s, 47%), 17.96 (s, 7%).

^{1}H NMR (400 MHz, Chloroform-d) δ 7.28 – 6.80 (m, 12H, 12xArH), 6.77 – 6.41 (m, 1H, NH), 5.47 – 5.14 (m, 1H, NH), 4.99 – 4.87 (m, 1H, CH), 4.74 – 4.11 (m, 3H, CH$_2$, CHP), 3.71 – 2.56 (m, 3H, CH$_2$, CH), 2.49 – 2.37 (m, 6H, 2xSCH$_3$), 2.36 – 2.17 (m, 1H, CH), 2.12 – 1.88 (m, 1H, CH), 1.44 – 1.30 (m, 9H, Boc), 1.15 – 0.81 (m, 12H, 4xCH$_3$).

HRMS: $C_{38}H_{50}N_3O_7PS_2$ [M+Na]$^+$ calc. 778.2725 found 778.2715

HPLC: purity >99%; retention time 20.107 and 20.297 min

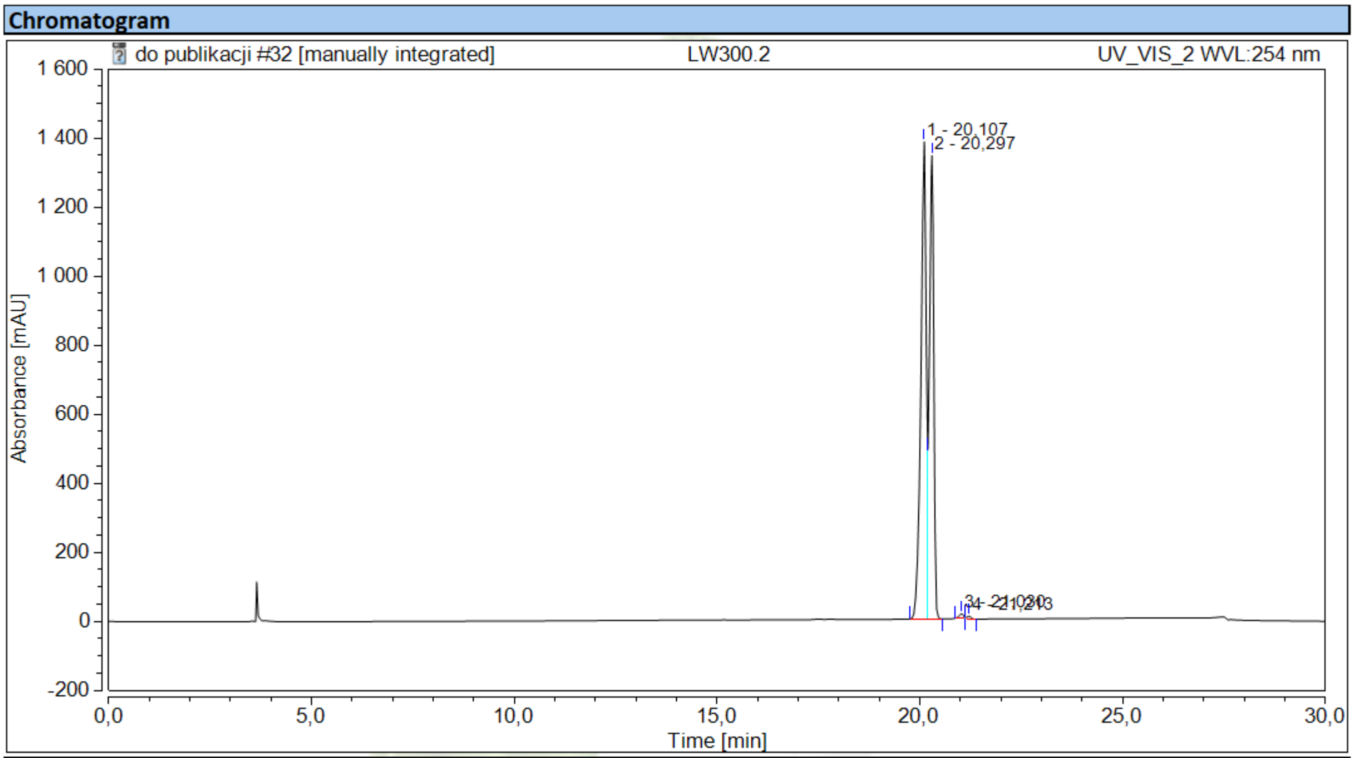

Integration Results

No.	Peak Name	Retention Time min	Area mAU*min	Height mAU	Relative Area %	Relative Height %	Amount n.a.
1		20,107	208,628	1381,674	54,93	50,32	n.a.
2		20,297	168,529	1341,930	44,37	48,87	n.a.
3		21,030	1,793	14,491	0,47	0,53	n.a.
4		21,213	0,843	7,849	0,22	0,29	n.a.
**Total:**			**379,794**	**2745,945**	**100,00**	**100,00**	

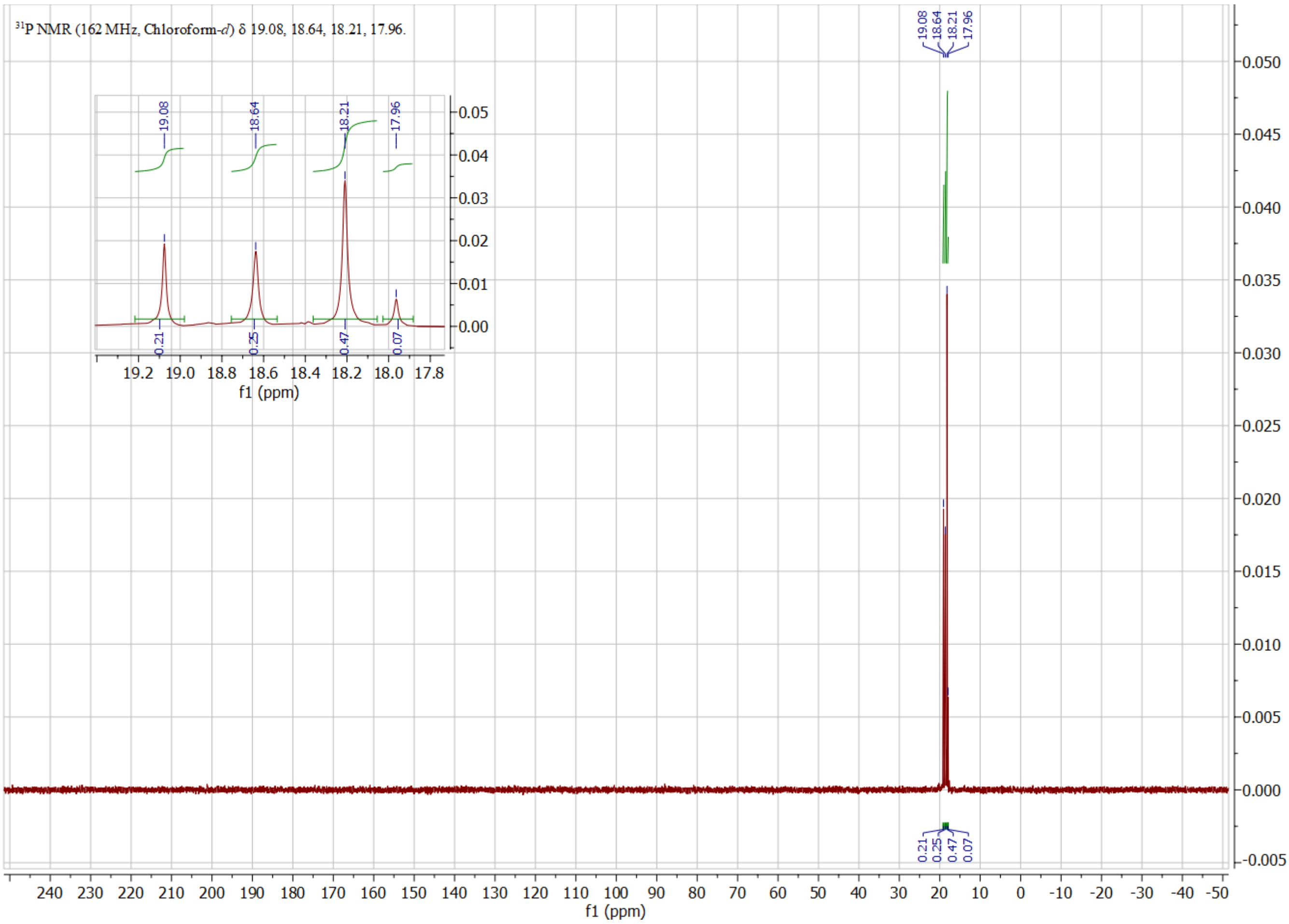
31P NMR (162 MHz, Chloroform-d) δ 19.08, 18.64, 18.21, 17.96.
f1 (ppm)

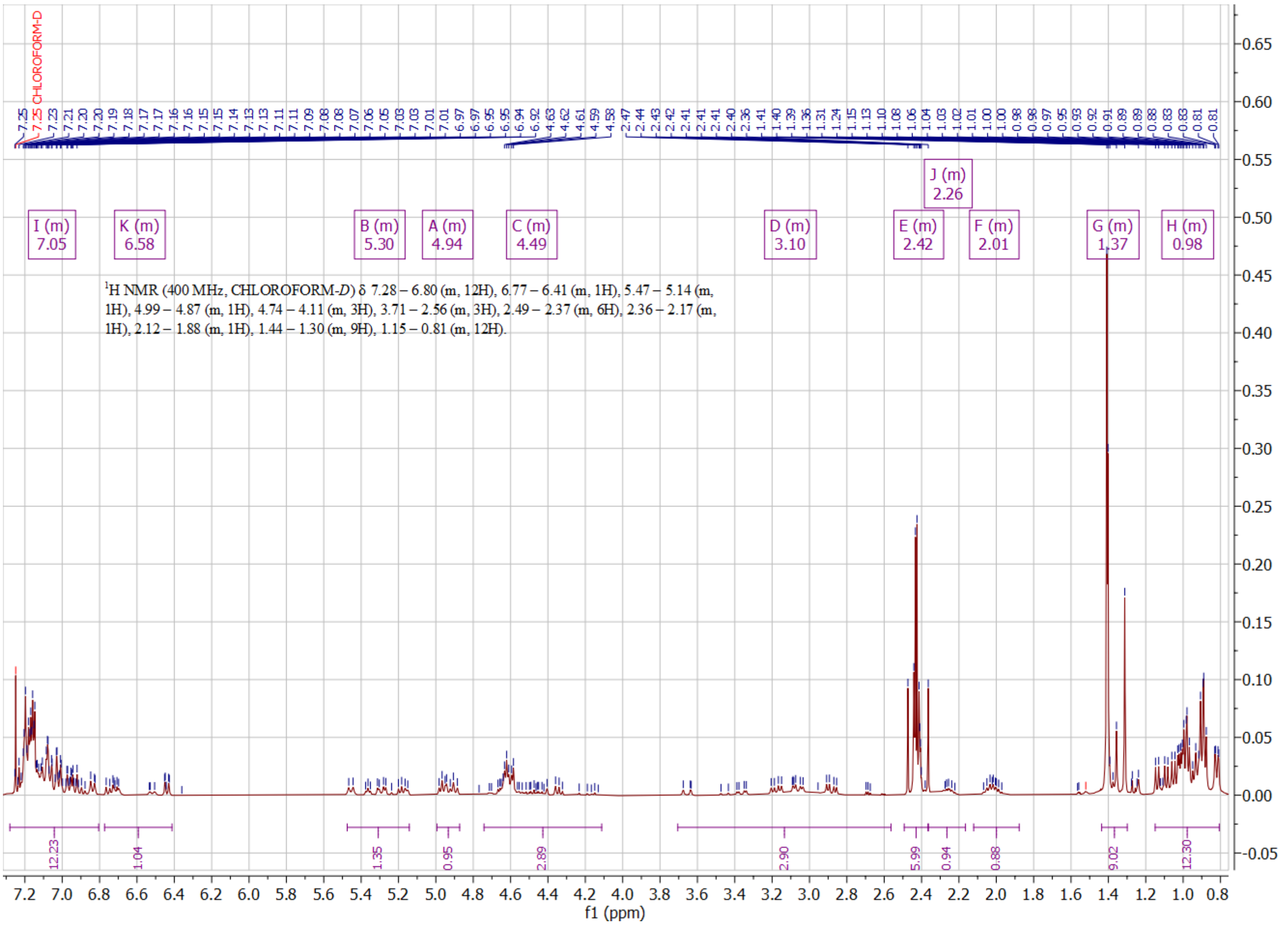
1H NMR (400 MHz, CHLOROFORM-D) δ 7.28 – 6.80 (m, 12H), 6.77 – 6.41 (m, 1H), 5.47 – 5.14 (m, 1H), 4.99 – 4.87 (m, 1H), 4.74 – 4.11 (m, 3H), 3.71 – 2.56 (m, 3H), 2.49 – 2.37 (m, 6H), 2.36 – 2.17 (m, 1H), 2.12 – 1.88 (m, 1H), 1.44 – 1.30 (m, 9H), 1.15 – 0.81 (m, 12H).
f1 (ppm)

(33) Boc-L-Val-D-Tic- Val^{P}(OPh-4-SCH$_3$)$_2$

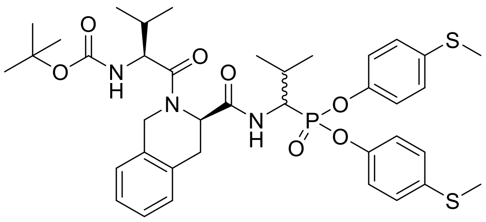

Name: *tert*-butyl ((2S)-1-((3R)-3-((1-(bis(4-(methylthio)phenoxy)phosphoryl)-2-methylpropyl)carbamoyl)-3,4-dihydroisoquinolin-2(1H)-yl)-3-methyl-1-oxobutan-2-yl)carbamate

^{31}P NMR (162 MHz, Chloroform-d) δ 18.79 (s, 46%), 18.32 (s, 54%).

^{1}H NMR (400 MHz, Chloroform-d) δ 7.24 – 6.84 (m, 12H, 12xArH), 6.67 (dd, J = 35.6, 9.3 Hz, 1H, NH), 5.60 – 4.95 (m, 2H, NH, CH), 4.94 – 4.65 (m, 2H, CH$_2$), 4.61 – 4.40 (m, 2H, CH$_2$), 3.52 – 3.36 (m, 1H, CHP), 3.05 – 2.89 (m, 1H, CH), 2.45 – 2.39 (m, 6H, 2xSCH$_3$), 2.33 – 2.17 (m, 1H, CH), 2.05 – 1.95 (m, 1H, CH), 1.43 – 1.32 (m, 9H, Boc), 1.05 – 0.60 (m, 12H, 4xCH$_3$).

HRMS: $C_{38}H_{50}N_3O_7PS_2$ [M+Na]$^+$ calc. 778.2725 found 778.2731

HPLC: purity >98%; retention time 20.377 and 20.170 min

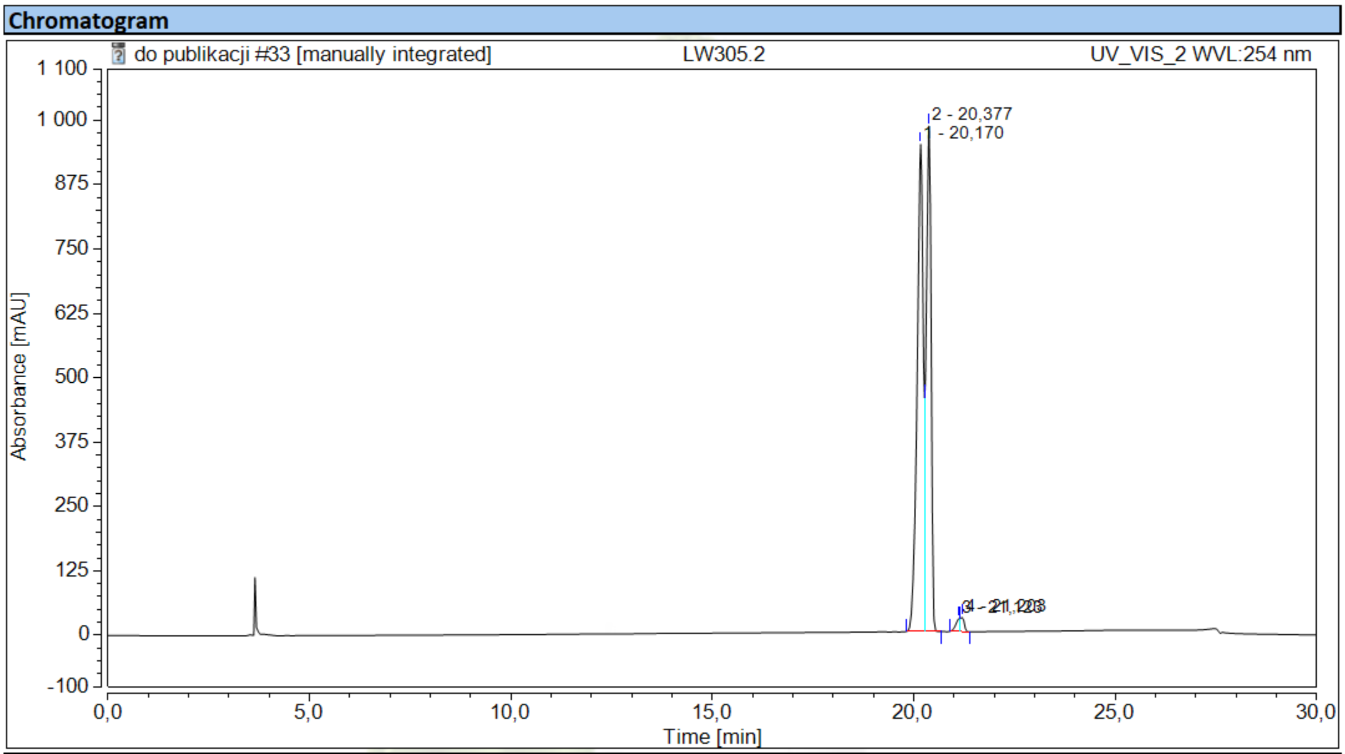

Integration Results

No.	Peak Name	Retention Time min	Area mAU*min	Height mAU	Relative Area %	Relative Height %	Amount n.a.
1		20,170	165,523	945,909	53,52	47,76	n.a.
2		20,377	137,718	981,307	44,53	49,55	n.a.
3		21,123	3,150	25,049	1,02	1,26	n.a.
4		21,203	2,907	28,320	0,94	1,43	n.a.
**Total:**			**309,298**	**1980,585**	**100,00**	**100,00**	

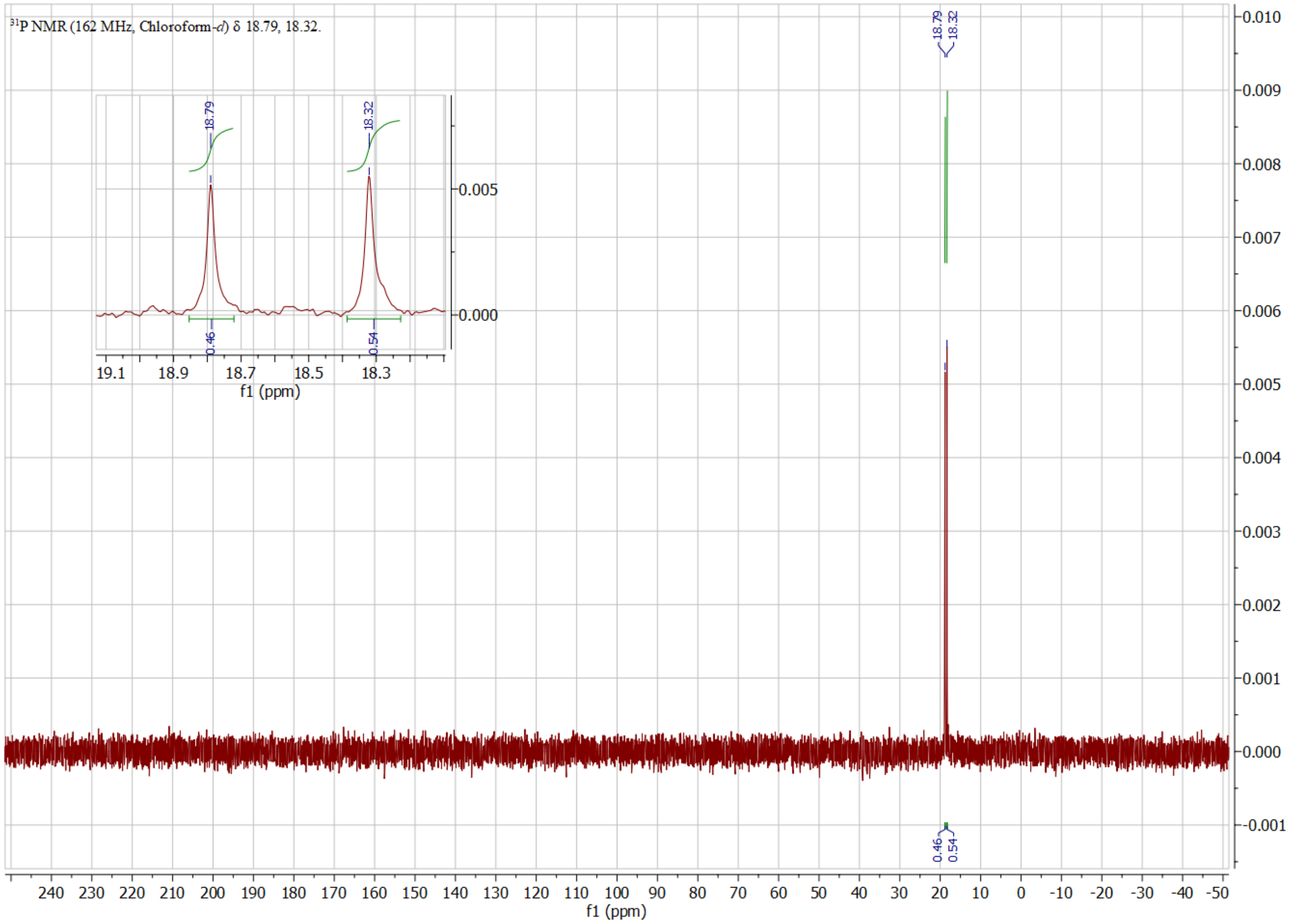
31P NMR (162 MHz, Chloroform-d) δ 18.79, 18.32.
f1 (ppm)

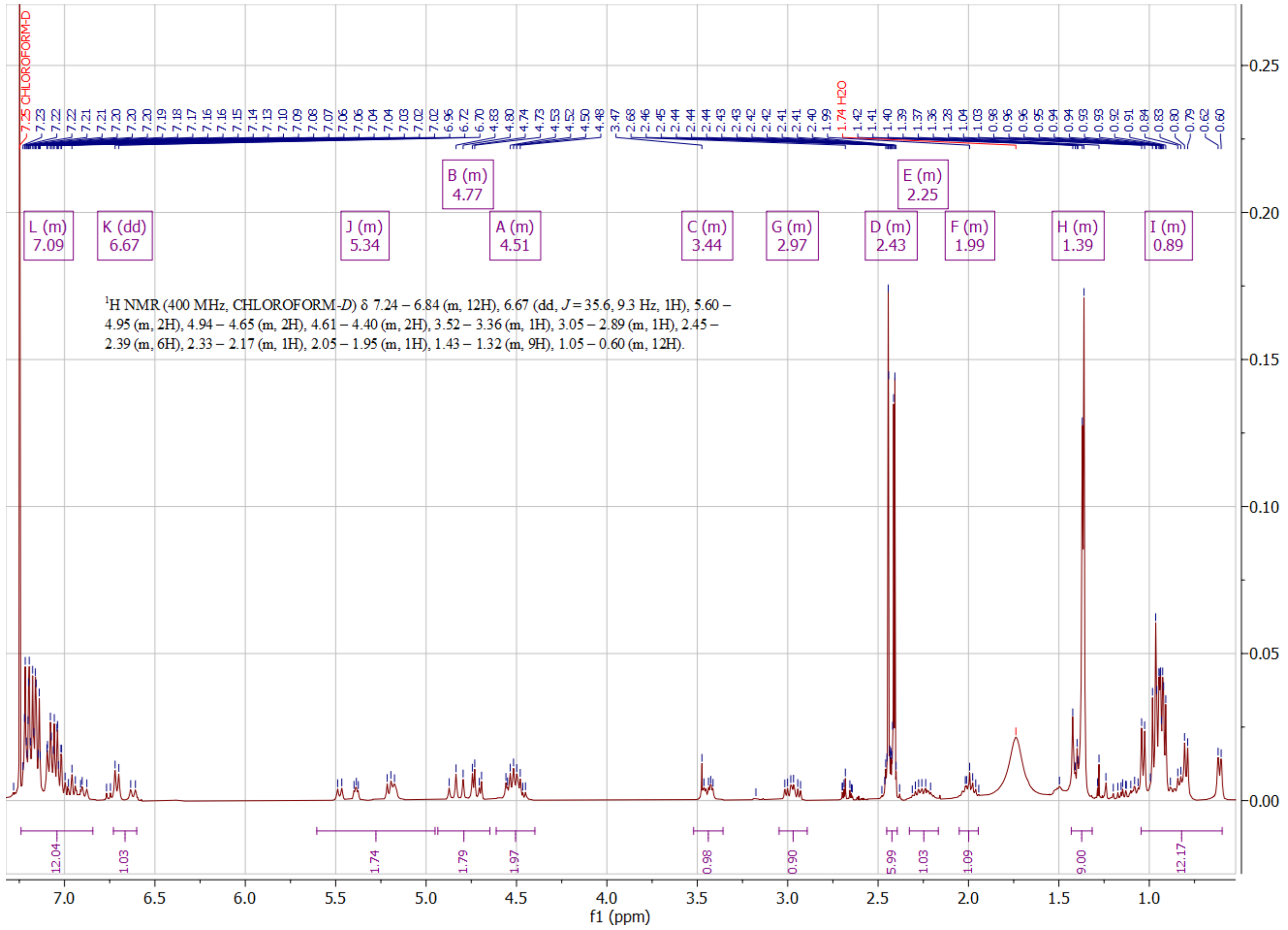
1H NMR (400 MHz, CHLOROFORM-D) δ 7.24 – 6.84 (m, 12H), 6.67 (dd, J = 35.6, 9.3 Hz, 1H), 5.60 – 4.95 (m, 2H), 4.94 – 4.65 (m, 2H), 4.61 – 4.40 (m, 2H), 3.52 – 3.36 (m, 1H), 3.05 – 2.89 (m, 1H), 2.45 – 2.39 (m, 6H), 2.33 – 2.17 (m, 1H), 2.05 – 1.95 (m, 1H), 1.43 – 1.32 (m, 9H), 1.05 – 0.60 (m, 12H).
f1 (ppm)

(34) Boc-L-Val-L-Nip-Val^P(OPh-4-SCH$_3$)$_2$

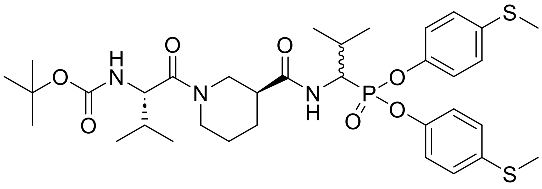

Name: *tert*-butyl ((2S)-1-((3S)-3-((1-(bis(4-(methylthio)phenoxy)phosphoryl)-2-methylpropyl)carbamoyl)piperidin-1-yl)-3-methyl-1-oxobutan-2-yl)carbamate
^{31}P NMR (243 MHz, CDCl3) δ 18.42 (s, 32%), 17.78 (s, 30%), 17.57 (s, 13%), 17.44 (s, 25%).
^{1}H NMR (601 MHz, CDCl3) δ 7.22 – 7.00 (m, 8H, 8xArH), 6.84 (dd, J = 130.5, 10.2 Hz, 1H, NH), 5.52 – 5.27 (m, 1H, NH), 4.82 – 4.72 (m, 1H, CHP), 4.61 – 3.76 (m, 3H, CH$_2$, CH), 3.64 – 3.35 (m, 1H, CH$_2$), 3.22 – 2.93 (m, 1H, CH$_2$), 2.48 – 2.41 (m, 6H, 2xSCH$_3$), 2.41 – 2.27 (m, 2H, 2xCH), 2.04 – 1.82 (m, 2H, CH$_2$), 1.82 – 1.61 (m, 2H, CH$_2$), 1.53 – 1.44 (m, 1H, CH), 1.44 – 1.32 (m, 9H, Boc), 1.18 – 1.00 (m, 6H, 2xCH$_3$), 0.94 – 0.73 (m, 6H, 2xCH$_3$).
HRMS: $C_{34}H_{50}N_3O_7PS_2$ [M+Na]$^+$ calc. 730.2725 found 730.2718
HPLC: purity >99%; retention time 19.043 min

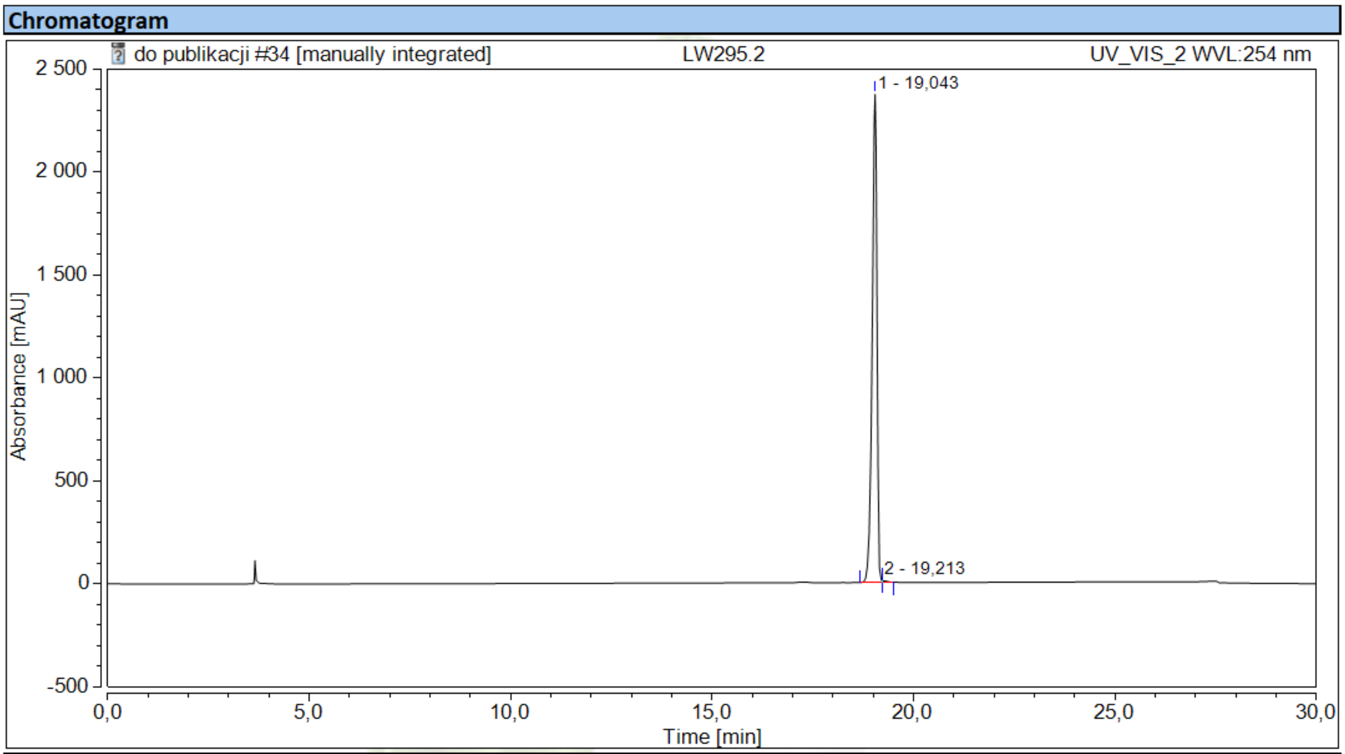

Integration Results

No.	Peak Name	Retention Time min	Area mAU*min	Height mAU	Relative Area %	Relative Height %	Amount n.a.
1		19,043	322,319	2369,236	99,63	99,61	n.a.
2		19,213	1,206	9,201	0,37	0,39	n.a.
**Total:**			**323,524**	**2378,436**	**100,00**	**100,00**	

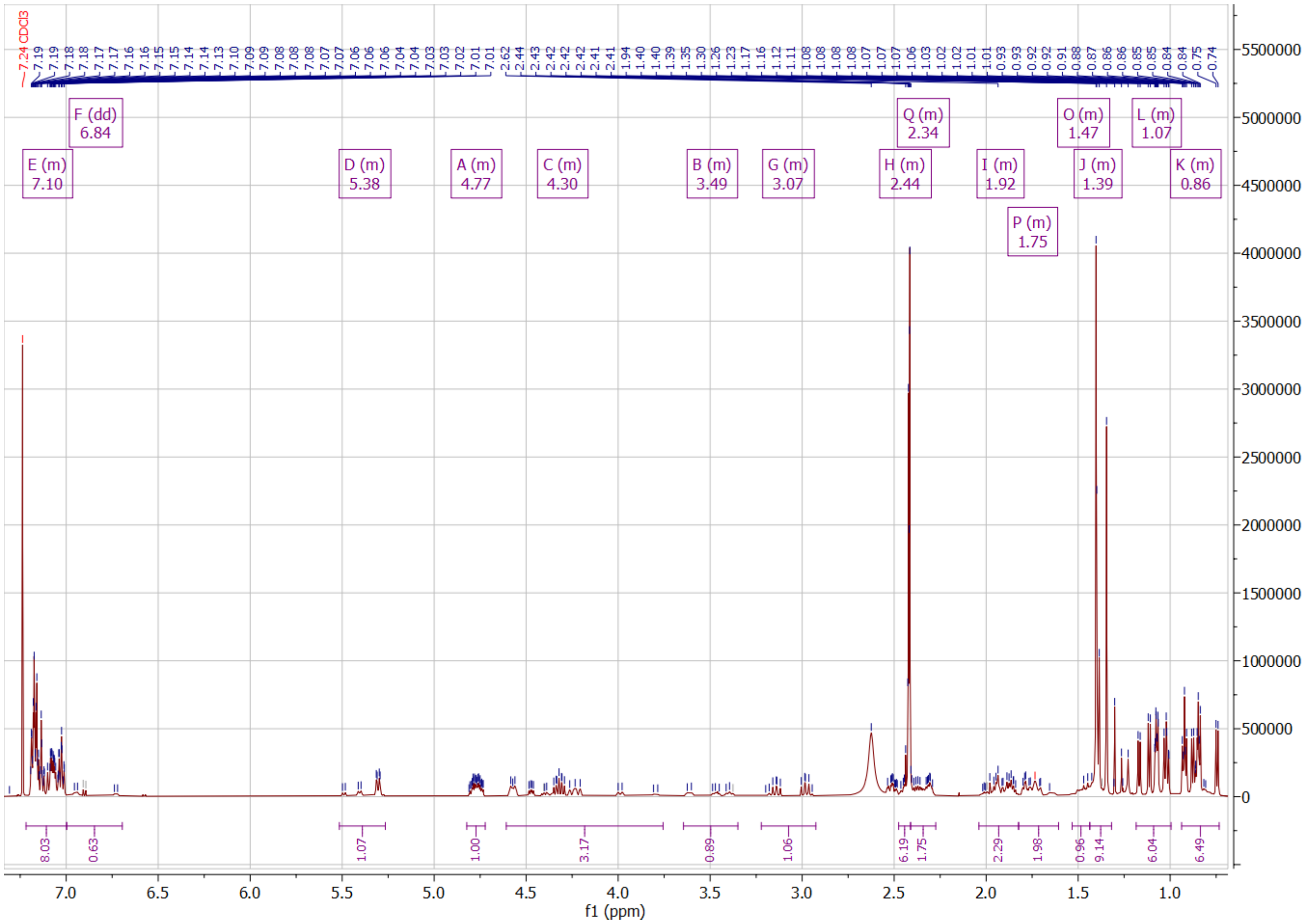

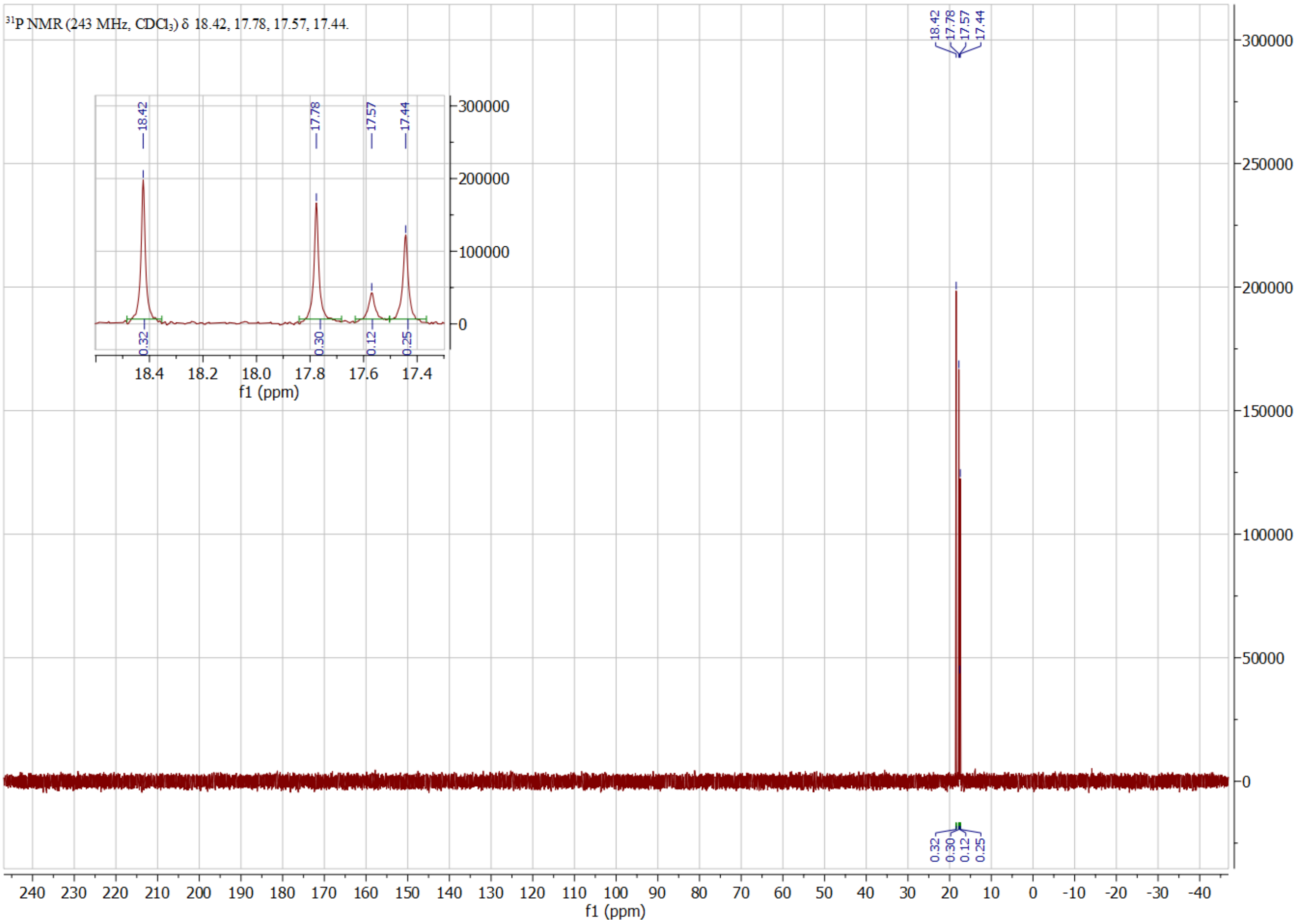

^{31}P NMR (243 MHz, $CDCl_3$) δ 18.42, 17.78, 17.57, 17.44.

(35) Boc-L-Val-L-Cpg-Val^{P}(OPh-4-SCH$_3$)$_2$

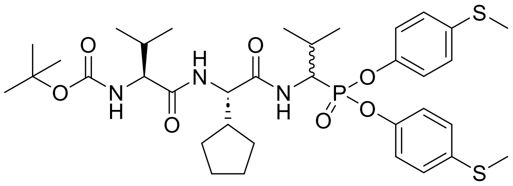

Name: *tert*-butyl ((2S)-1-(((1S)-2-((1-(bis(4-(methylthio)phenoxy)phosphoryl)-2-methylpropyl)amino)-1-cyclopentyl-2-oxoethyl)amino)-3-methyl-1-oxobutan-2-yl)carbamate

^{31}P NMR (162 MHz, Chloroform-d) δ 18.43 (s, 45%), 18.40 (s, 55%).

^{1}H NMR (400 MHz, Chloroform-d) δ 7.21 – 7.12 (m, 4H, 4xArH), 7.12 – 6.90 (m, 5H, 4xArH, NH), 6.63 (dd, J = 26.2, 8.1 Hz, 1H, NH), 5.11 (dd, J = 75.1, 8.5 Hz, 1H, NH), 4.78 – 4.66 (m, 1H, CHP), 4.42 – 4.32 (m, 1H, CH), 3.90 – 3.81 (m, 1H, CH), 2.47 – 2.41 (m, 6H, 2xSCH$_3$), 2.41 – 2.22 (m, 2H, 2xCH), 2.16 – 2.02 (m, 1H, CH), 1.81 – 1.65 (m, 2H, CH$_2$), 1.65 – 1.45 (m, 4H, 2XCH$_2$), 1.44 – 1.36 (m, 9H, Boc), 1.31 – 1.21 (m, 2H, CH$_2$), 1.12 – 0.97 (m, 6H, 2xCH$_3$), 0.94 – 0.80 (m, 6H, 2xCH$_3$).

HRMS: $C_{35}H_{52}N_3O_7PS_2$ [M+Na]$^+$ calc. 744.2882 found 744.2879

HPLC: purity >99%; retention time 19.417 and 19.667 min

Chromatogram

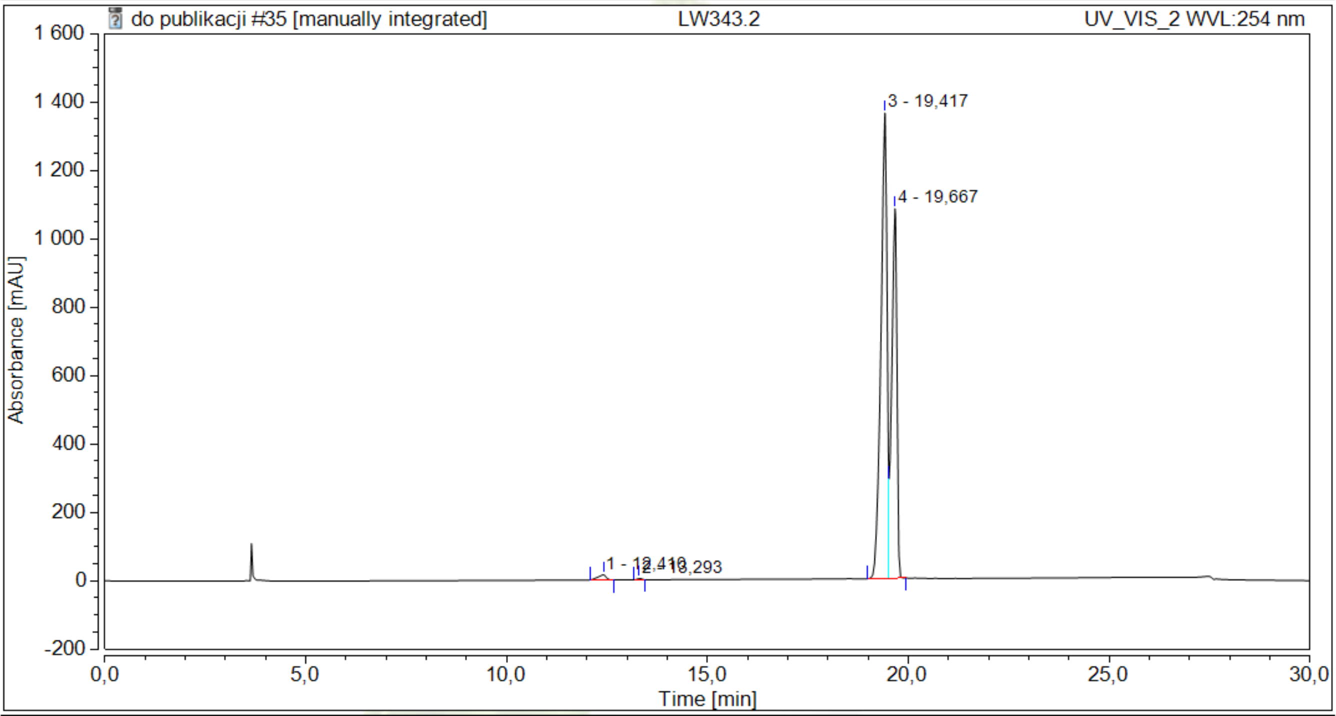

Integration Results

No.	Peak Name	Retention Time min	Area mAU*min	Height mAU	Relative Area %	Relative Height %	Amount n.a.
1		12,410	3,516	15,279	0,87	0,62	n.a.
2		13,293	0,464	3,574	0,11	0,15	n.a.
3		19,417	240,607	1360,620	59,44	55,31	n.a.
4		19,667	160,180	1080,339	39,57	43,92	n.a.
**Total:**			**404,768**	**2459,812**	**100,00**	**100,00**	

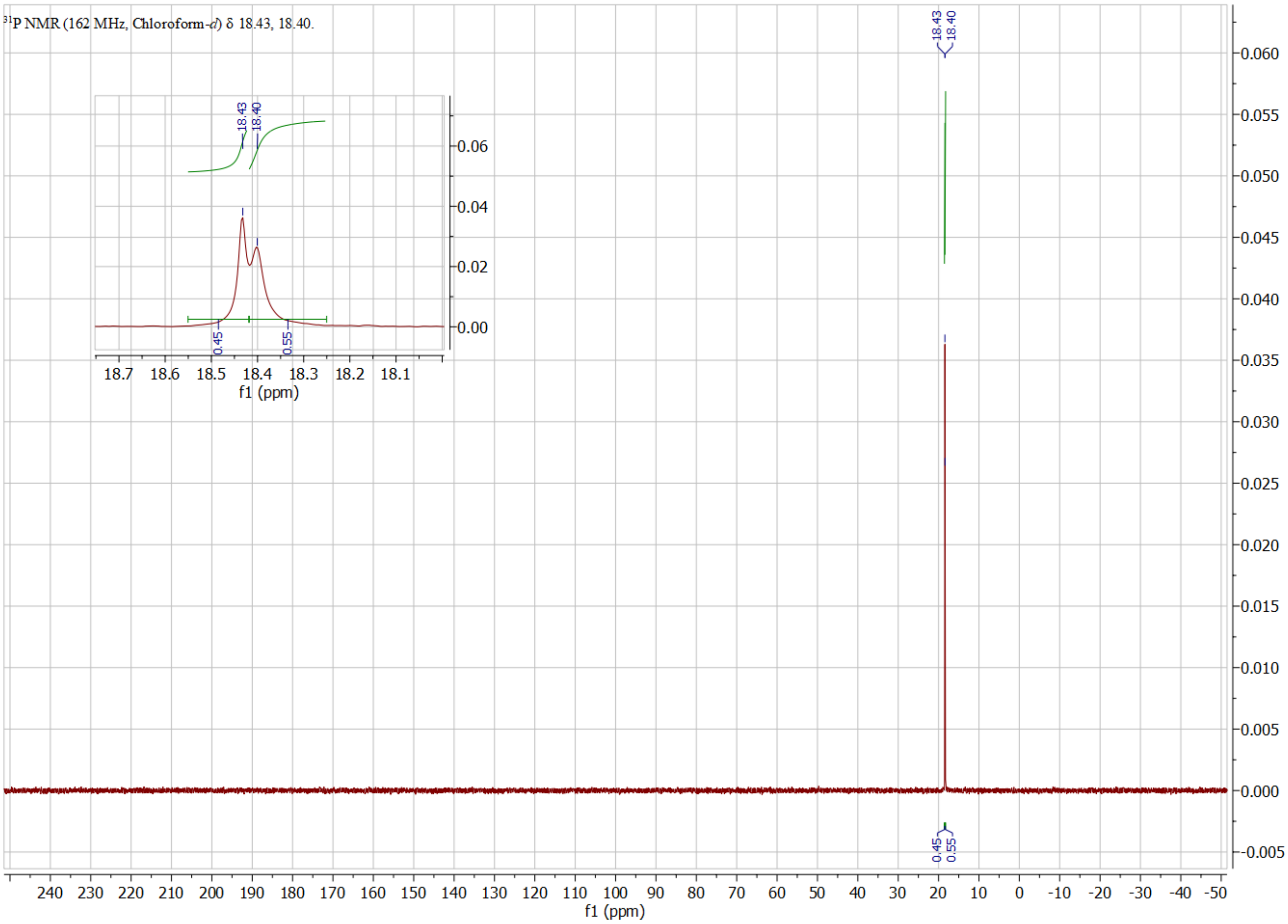

31P NMR (162 MHz, Chloroform-d) δ 18.43, 18.40.
f1 (ppm)

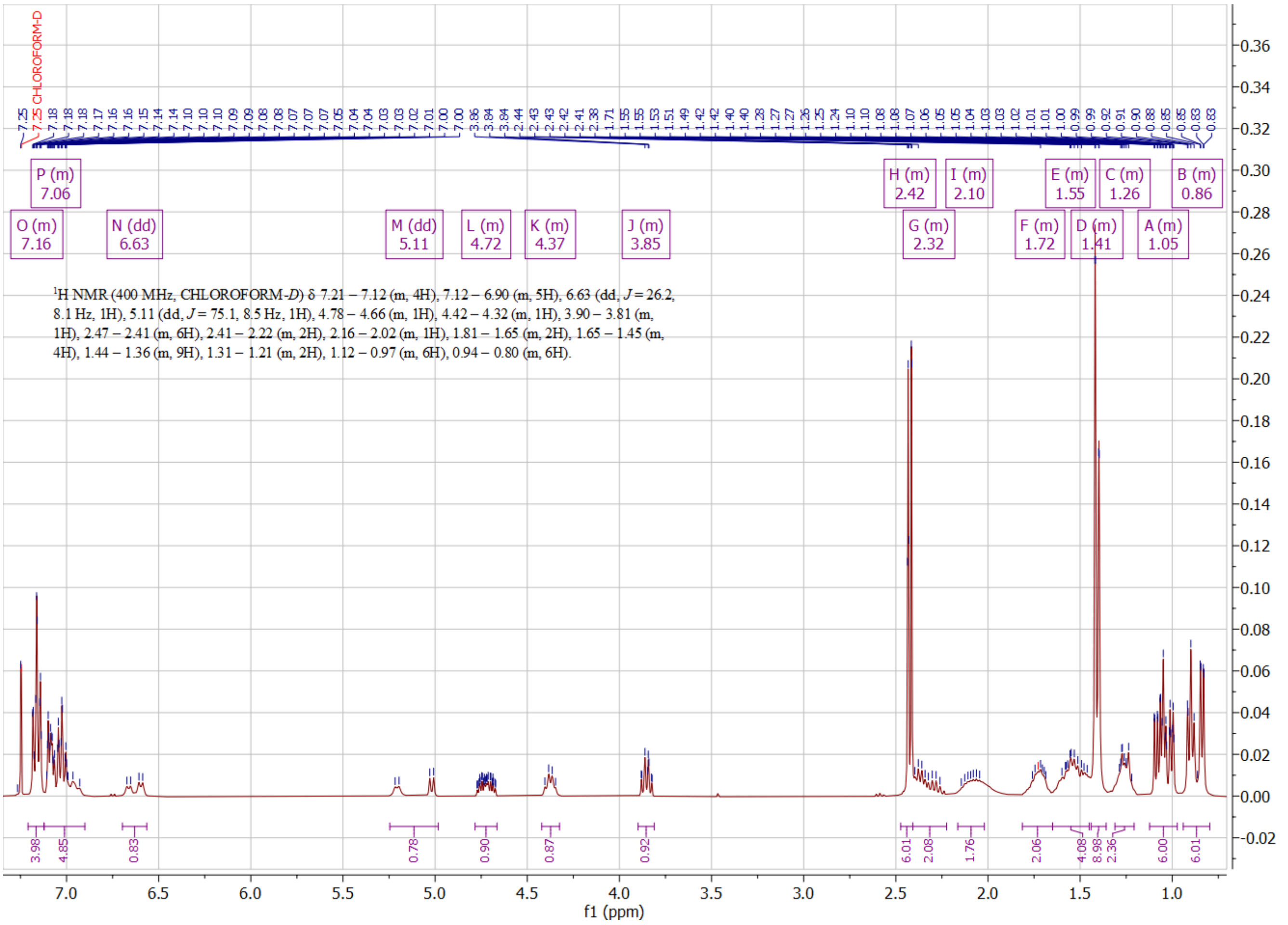

1H NMR (400 MHz, CHLOROFORM-D) δ 7.21 – 7.12 (m, 4H), 7.12 – 6.90 (m, 5H), 6.63 (dd, J = 26.2, 8.1 Hz, 1H), 5.11 (dd, J = 75.1, 8.5 Hz, 1H), 4.78 – 4.66 (m, 1H), 4.42 – 4.32 (m, 1H), 3.90 – 3.81 (m, 1H), 2.47 – 2.41 (m, 6H), 2.41 – 2.22 (m, 2H), 2.16 – 2.02 (m, 1H), 1.81 – 1.65 (m, 2H), 1.65 – 1.45 (m, 4H), 1.44 – 1.36 (m, 9H), 1.31 – 1.21 (m, 2H), 1.12 – 0.97 (m, 6H), 0.94 – 0.80 (m, 6H).
f1 (ppm)

(36) 344.2 Boc-L-Val-L-Chg-Val^{P}(OPh-4-SCH$_3$)$_2$

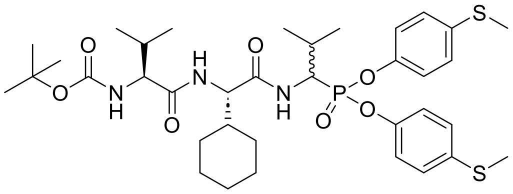

Name: *tert*-butyl ((2S)-1-(((1S)-2-((1-(bis(4-(methylthio)phenoxy)phosphoryl)-2-methylpropyl)amino)-1-cyclohexyl-2-oxoethyl)amino)-3-methyl-1-oxobutan-2-yl)carbamate

^{31}P NMR (162 MHz, Chloroform-d) δ 18.34 (s).

^{1}H NMR (400 MHz, Chloroform-d) δ 7.19 – 7.12 (m, 4H, 4xArH), 7.10 – 6.98 (m, 4H, 4xArH), 6.89 (dd, J = 34.5, 10.5 Hz, 1H, NH), 6.61 (dd, J = 23.7, 8.6 Hz, 1H, NH), 5.14 (dd, J = 84.0, 8.2 Hz, 1H, NH), 4.79 – 4.67 (m, 1H, CHP), 4.45 – 4.31 (m, 1H, CH), 3.89 – 3.81 (m, 1H, CH), 2.42 (dd, J = 6.4, 1.7 Hz, 6H, 2xSCH$_3$), 2.40 – 2.30 (m, 1H, CH), 2.18 – 2.03 (m, 1H, CH), 1.93 – 1.84 (m, 1H, CH), 1.84 – 1.54 (m, 6H, 3xCH$_2$), 1.41 (d, J = 7.1 Hz, 9H, Boc), 1.17 – 0.93 (m, 10H, 2xCH$_3$, 2xCH$_2$), 0.92 – 0.77 (m, 6H, 2xCH$_3$).

HRMS: $C_{36}H_{54}N_3O_7PS_2$ [M+Na]$^+$ calc. 758.3038 found 758.3048

HPLC: purity >98%; retention time 19.830 and 20.167 min

Chromatogram

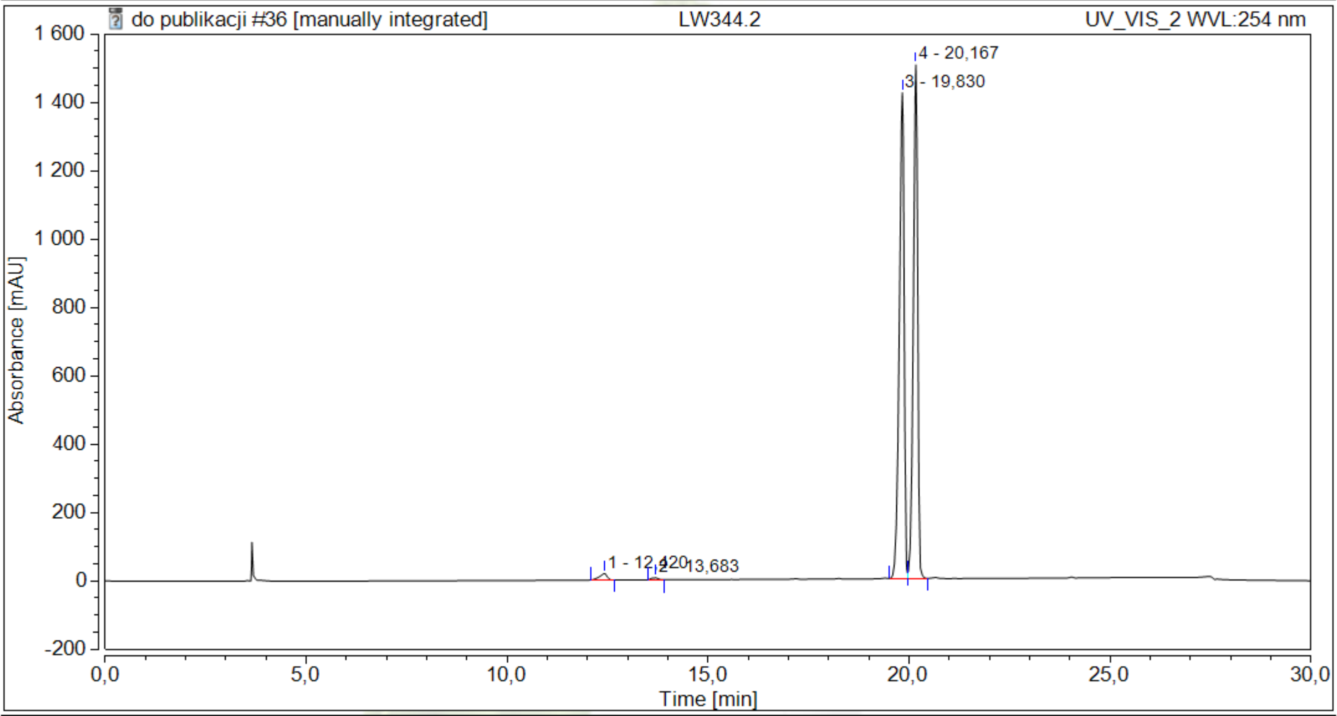

Integration Results

No.	Peak Name	Retention Time min	Area mAU*min	Height mAU	Relative Area %	Relative Height %	Amount n.a.
1		12,420	4,171	19,482	1,07	0,66	n.a.
2		13,683	1,126	6,356	0,29	0,22	n.a.
3		19,830	197,108	1420,715	50,46	48,18	n.a.
4		20,167	188,222	1502,174	48,18	50,94	n.a.
**Total:**			**390,626**	**2948,726**	**100,00**	**100,00**	

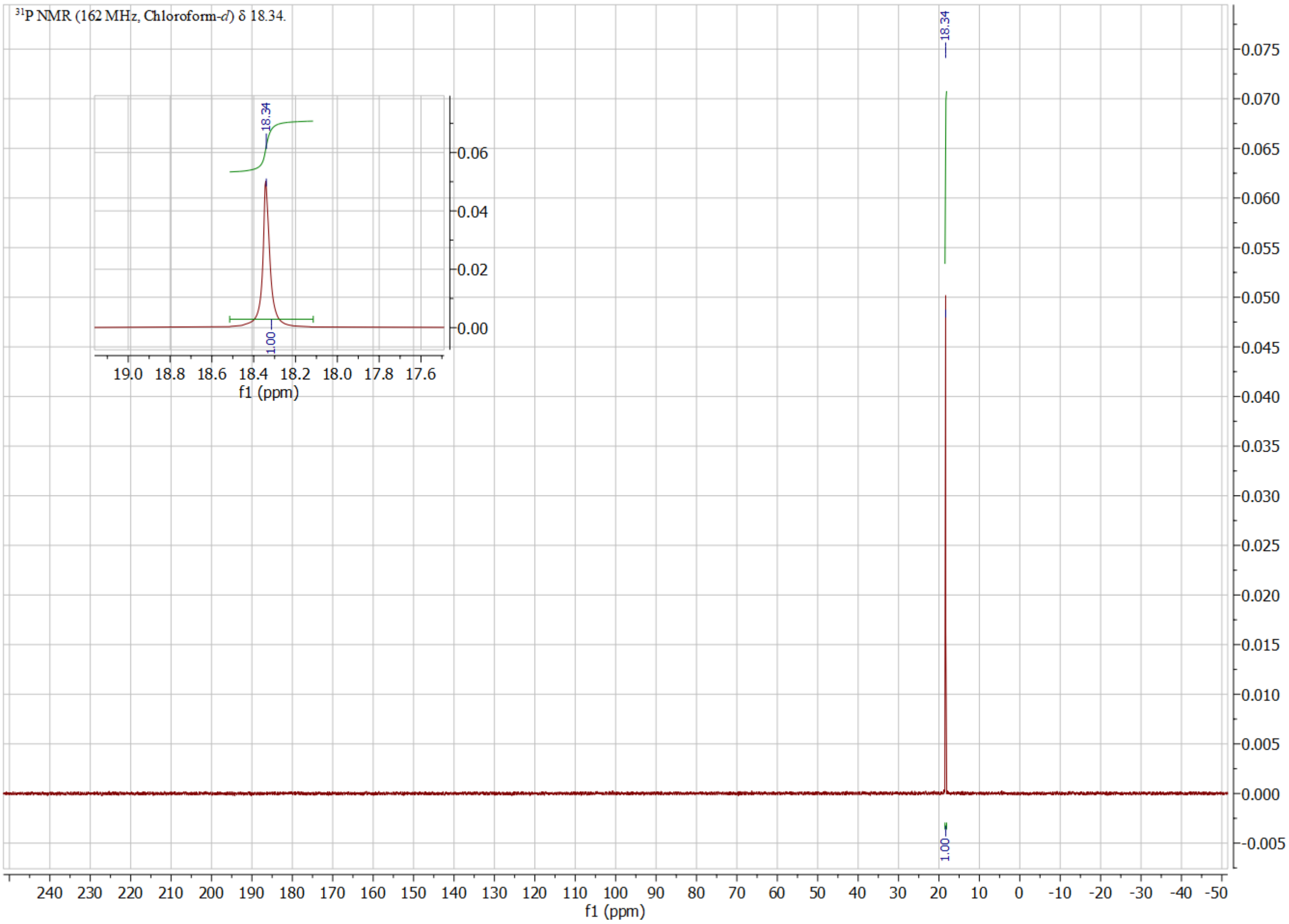
31P NMR (162 MHz, Chloroform-d) δ 18.34.
f1 (ppm)

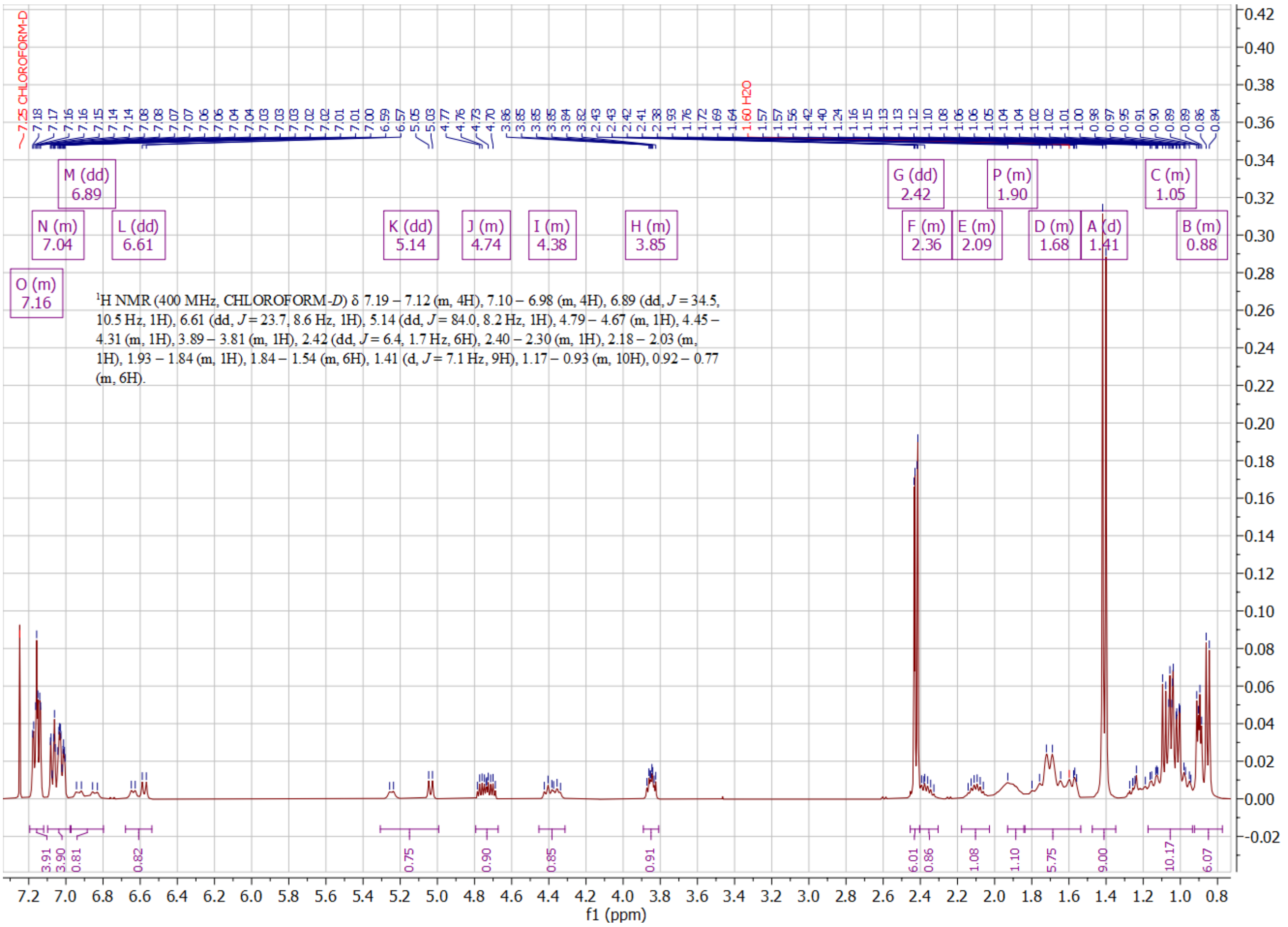
1H NMR (400 MHz, CHLOROFORM-D) δ 7.19 – 7.12 (m, 4H), 7.10 – 6.98 (m, 4H), 6.89 (dd, J = 34.5, 10.5 Hz, 1H), 6.61 (dd, J = 23.7, 8.6 Hz, 1H), 5.14 (dd, J = 84.0, 8.2 Hz, 1H), 4.79 – 4.67 (m, 1H), 4.45 – 4.31 (m, 1H), 3.89 – 3.81 (m, 1H), 2.42 (dd, J = 6.4, 1.7 Hz, 6H), 2.40 – 2.30 (m, 1H), 2.18 – 2.03 (m, 1H), 1.93 – 1.84 (m, 1H), 1.84 – 1.54 (m, 6H), 1.41 (d, J = 7.1 Hz, 9H), 1.17 – 0.93 (m, 10H), 0.92 – 0.77 (m, 6H).
f1 (ppm)

(37) Boc-L-Val-D-Chg-Val^{P}(OPh-4-SCH$_3$)$_2$

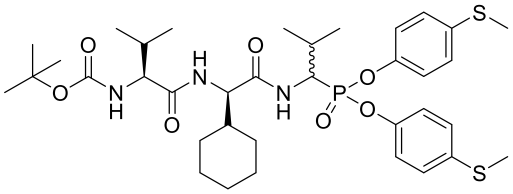

Name: *tert*-butyl ((2S)-1-(((1R)-2-((1-(bis(4-(methylthio)phenoxy)phosphoryl)-2-methylpropyl)amino)-1-cyclohexyl-2-oxoethyl)amino)-3-methyl-1-oxobutan-2-yl)carbamate

^{31}P NMR (162 MHz, Chloroform-d) δ 18.39 (s, 47%), 18.21 (s, 53%).

^{1}H NMR (400 MHz, Chloroform-d) δ 7.21 – 6.88 (m, 9H, 8xArH, NH), 6.78 (dd, J = 64.0, 7.9 Hz, 1H, NH), 5.43 – 4.92 (m, 1H, NH), 4.81 – 4.65 (m, 1H, CHP), 4.48 – 4.34 (m, 1H, CH), 3.98 – 3.86 (m, 1H, CH), 2.42 (d, J = 5.7 Hz, 7H, , 2xSCH$_3$, CH), 2.19 – 2.08 (m, 1H, CH), 1.97 – 1.47 (m, 7H, 3xCH$_2$, CH), 1.40 (d, J = 3.3 Hz, 9H, Boc), 1.11 – 1.04 (m, 6H, 2xCH$_3$), 1.04 – 0.76 (m, 10H, 2xCH$_3$, 2xCH$_2$).

HRMS: $C_{36}H_{54}N_3O_7PS_2$ [M+Na]$^+$ calc. 758.3038 found 758.3044

HPLC: purity >98%; retention time 19.970 and 20.277 min

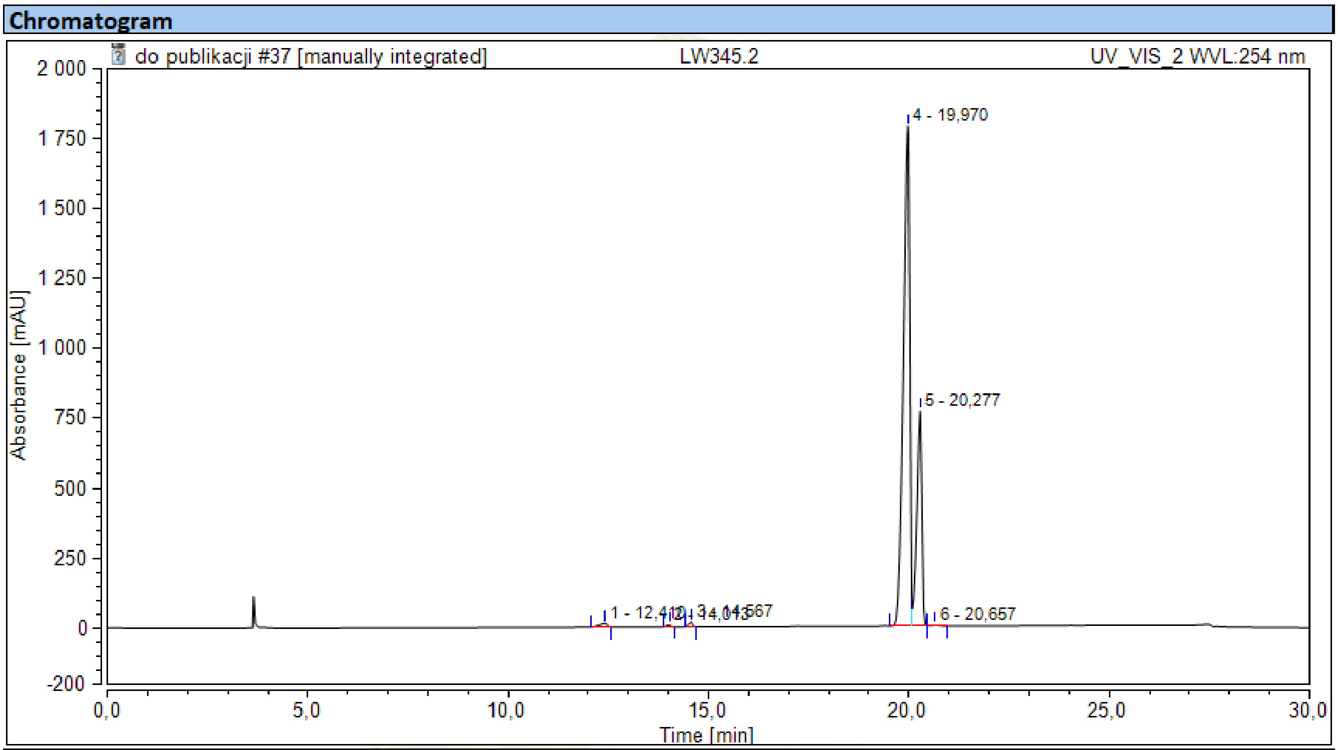

Integration Results

No.	Peak Name	Retention Time min	Area mAU*min	Height mAU	Relative Area %	Relative Height %	Amount n.a.
1		12,410	3,235	14,505	0,74	0,56	n.a.
2		14,013	0,850	7,091	0,19	0,27	n.a.
3		14,567	2,034	17,931	0,46	0,69	n.a.
4		19,970	317,954	1788,303	72,27	68,77	n.a.
5		20,277	114,974	769,457	26,13	29,59	n.a.
6		20,657	0,912	3,168	0,21	0,12	n.a.
**Total:**			**439,958**	**2600,456**	**100,00**	**100,00**	

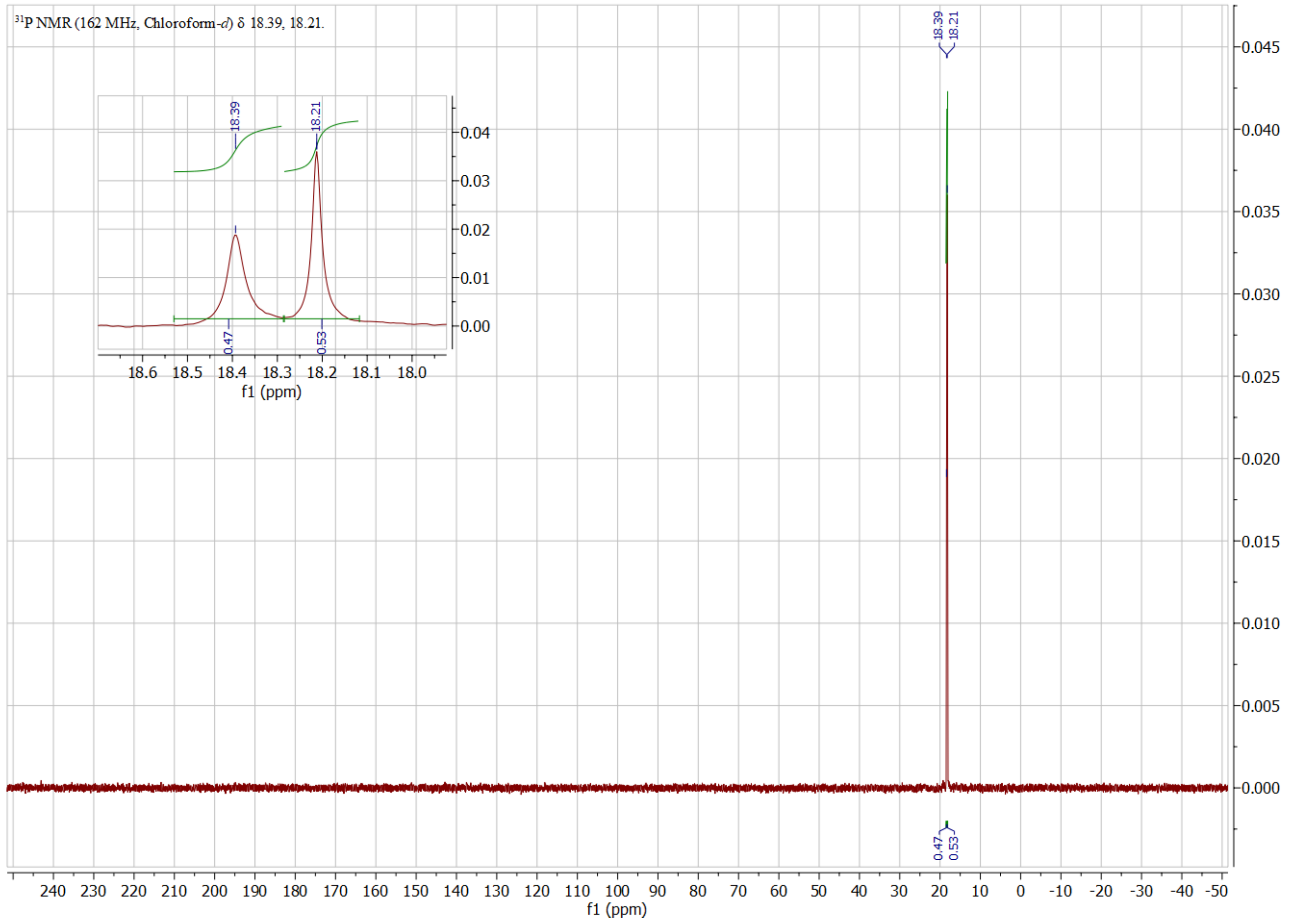

31P NMR (162 MHz, Chloroform-d) δ 18.39, 18.21.
18.39
18.21
0.47
0.53
f1 (ppm)

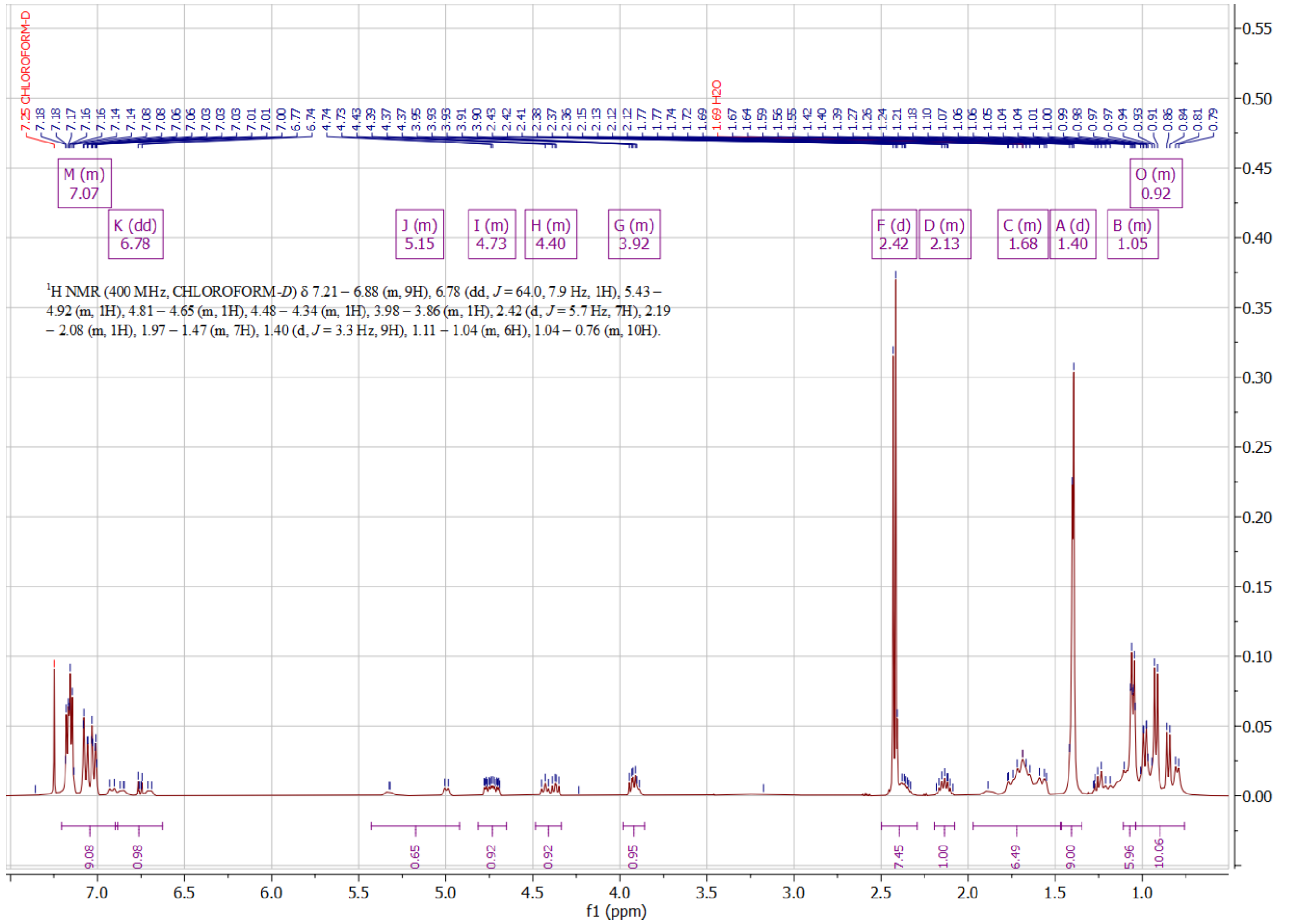

7.25 CHLOROFORM-D
1.69 H2O
M (m) 7.07
K (dd) 6.78
J (m) 5.15
I (m) 4.73
H (m) 4.40
G (m) 3.92
F (d) 2.42
D (m) 2.13
C (m) 1.68
A (d) 1.40
B (m) 1.05
O (m) 0.92
1H NMR (400 MHz, CHLOROFORM-D) δ 7.21 – 6.88 (m, 9H), 6.78 (dd, J = 64.0, 7.9 Hz, 1H), 5.43 – 4.92 (m, 1H), 4.81 – 4.65 (m, 1H), 4.48 – 4.34 (m, 1H), 3.98 – 3.86 (m, 1H), 2.42 (d, J = 5.7 Hz, 7H), 2.19 – 2.08 (m, 1H), 1.97 – 1.47 (m, 7H), 1.40 (d, J = 3.3 Hz, 9H), 1.11 – 1.04 (m, 6H), 1.04 – 0.76 (m, 10H).
9.08
0.98
0.65
0.92
0.92
0.95
7.45
1.00
6.49
9.00
5.96
10.06
f1 (ppm)

(38) Boc-L-Val-L-Phe- Val^{P}(OPh-4-SCH$_3$)$_2$

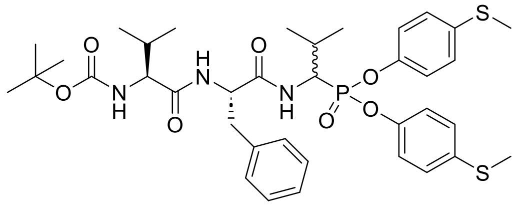

Name: *tert*-butyl ((2S)-1-(((2S)-1-((1-(bis(4-(methylthio)phenoxy)phosphoryl)-2-methylpropyl)amino)-1-oxo-3-phenylpropan-2-yl)amino)-3-methyl-1-oxobutan-2-yl)carbamate

^{31}P NMR (162 MHz, Chloroform-d) δ 18.13 (s, 48%), 18.01 (s, 52%).

^{1}H NMR (400 MHz, Chloroform-d) δ 7.25 (s, 10H, 9xArH, NH), 7.10 – 7.00 (m, 4H, 4xArH), 6.61 – 6.50 (m, 1H, NH), 4.89 – 4.77 (m, 1H, NH), 4.77 – 4.61 (m, 2H, CHP, CH), 3.89 – 3.81 (m, 1H, CH), 3.14 – 2.98 (m, 2H, CH$_2$), 2.44 – 2.40 (m, 6H, 2xSCH$_3$), 2.40 – 2.23 (m, 1H, CH), 2.18 – 1.98 (m, 1H, CH), 1.39 (d, J = 4.1 Hz, 9H, Boc), 1.04 – 0.96 (m, 3H, CH$_3$), 0.94 – 0.78 (m, 6H, 2xCH$_3$), 0.73 (dd, J = 16.6, 6.5 Hz, 3H, CH$_3$).

HRMS: $C_{37}H_{50}N_3O_7PS_2$ $[M+Na]^+$ calc. 766.2725 found 766.2703

HPLC: purity >99%; retention time 19.513 min

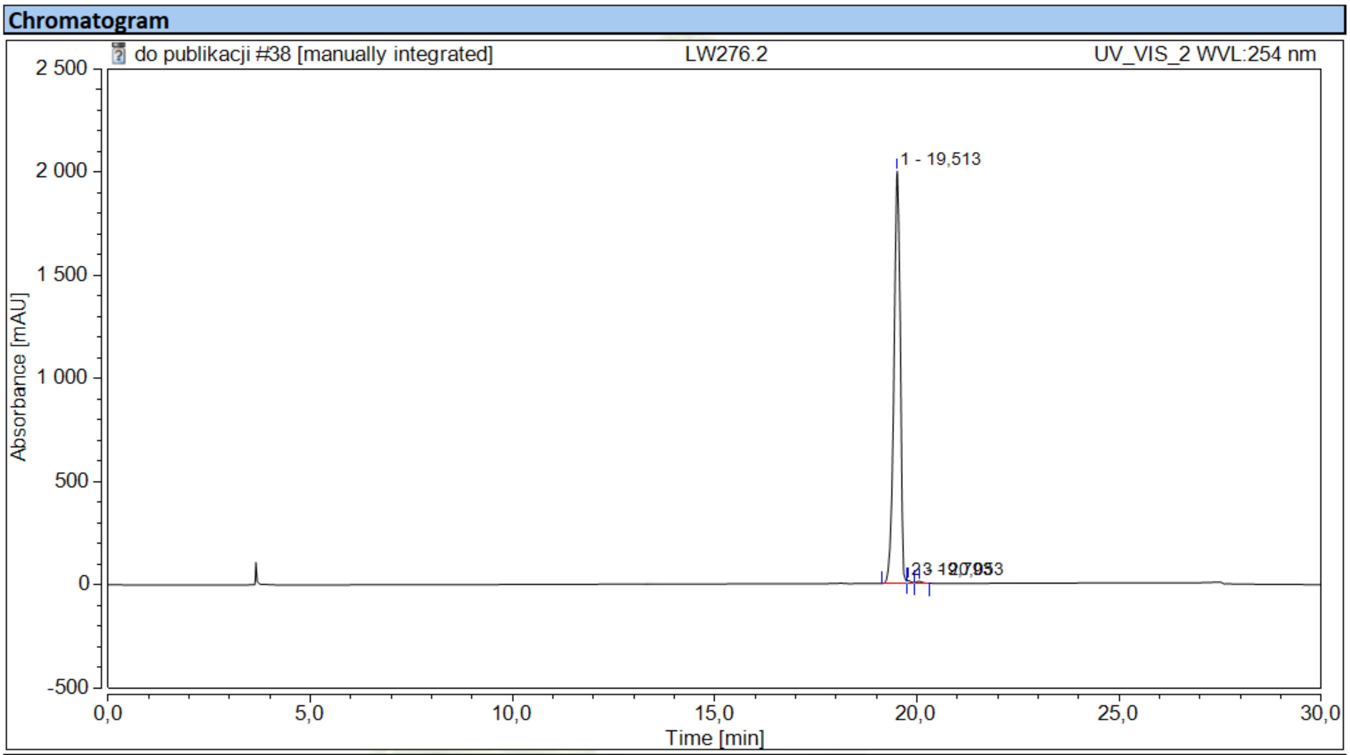

Integration Results

No.	Peak Name	Retention Time min	Area mAU*min	Height mAU	Relative Area %	Relative Height %	Amount n.a.
1		19,513	361,273	1997,430	99,09	98,82	n.a.
2		19,793	1,592	13,202	0,44	0,65	n.a.
3		20,053	1,730	10,573	0,47	0,52	n.a.
**Total:**			**364,596**	**2021,205**	**100,00**	**100,00**	

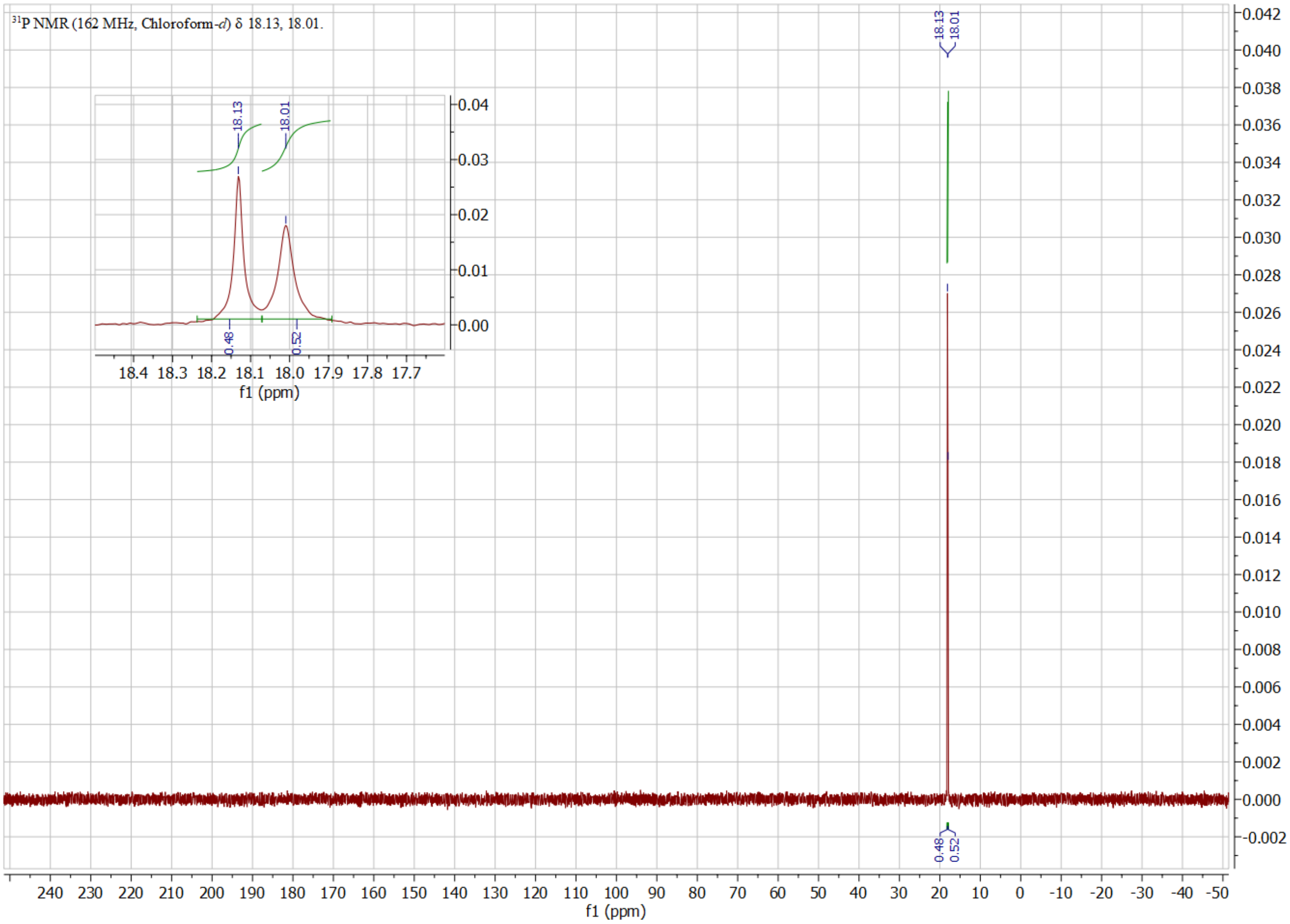

^{31}P NMR (162 MHz, Chloroform-*d*) δ 18.13, 18.01.
f1 (ppm)

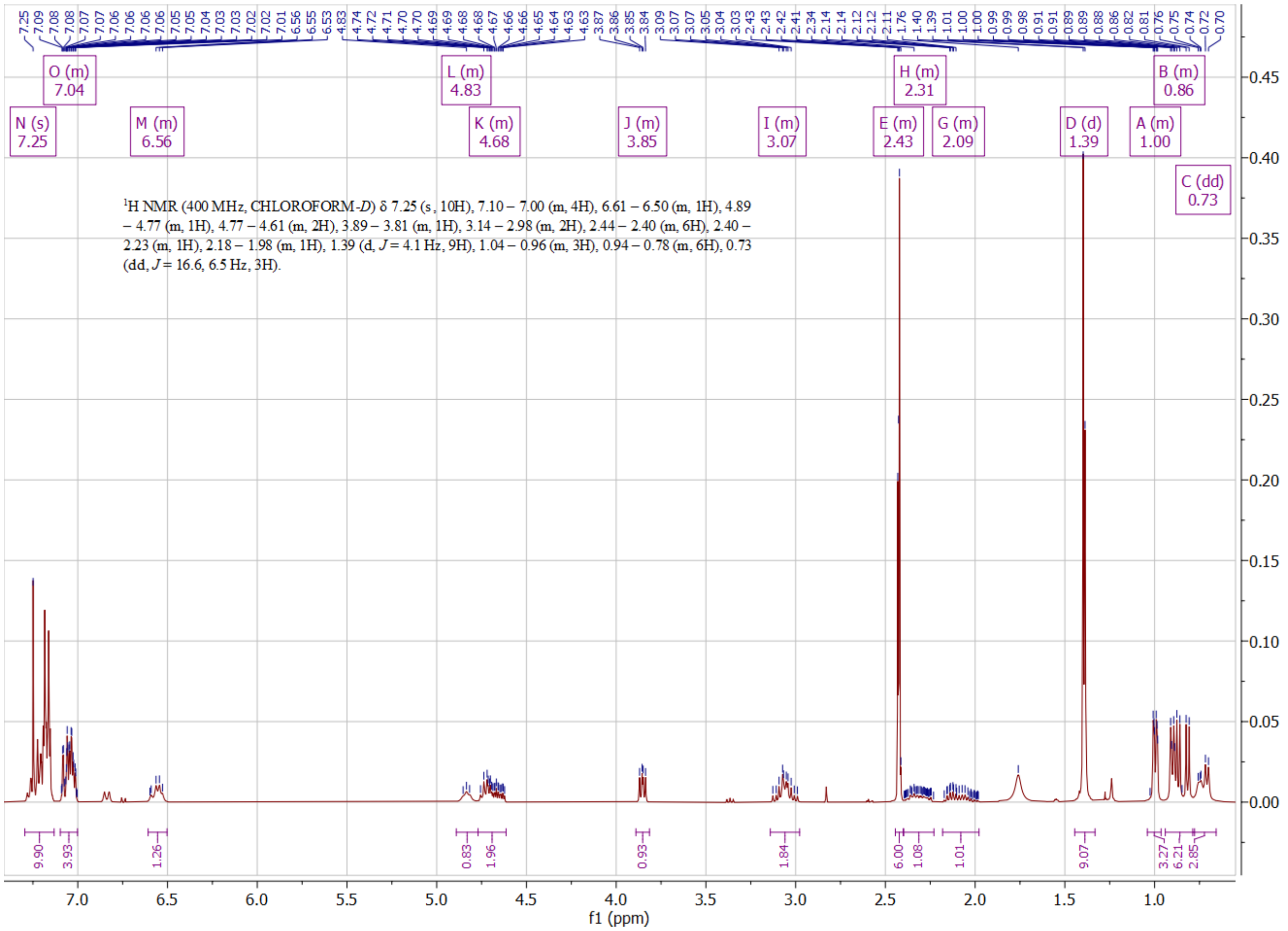

^{1}H NMR (400 MHz, CHLOROFORM-*D*) δ 7.25 (s, 10H), 7.10 – 7.00 (m, 4H), 6.61 – 6.50 (m, 1H), 4.89 – 4.77 (m, 1H), 4.77 – 4.61 (m, 2H), 3.89 – 3.81 (m, 1H), 3.14 – 2.98 (m, 2H), 2.44 – 2.40 (m, 6H), 2.40 – 2.23 (m, 1H), 2.18 – 1.98 (m, 1H), 1.39 (d, *J* = 4.1 Hz, 9H), 1.04 – 0.96 (m, 3H), 0.94 – 0.78 (m, 6H), 0.73 (dd, *J* = 16.6, 6.5 Hz, 3H).
f1 (ppm)

(39) Boc-L-Val-D-Phe-Val^P(OPh-4-SCH$_3$)$_2$

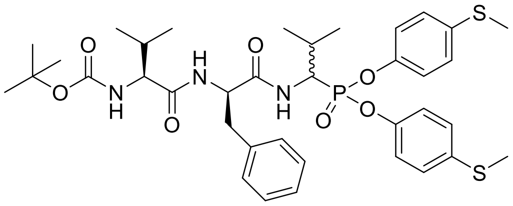

Name: *tert*-butyl ((2S)-1-(((2R)-1-((1-(bis(4-(methylthio)phenoxy)phosphoryl)-2-methylpropyl)amino)-1-oxo-3-phenylpropan-2-yl)amino)-3-methyl-1-oxobutan-2-yl)carbamate
^{31}P NMR (162 MHz, Chloroform-d) δ 18.12 (s, 40%), 17.87 (s, 60%).
^{1}H NMR (400 MHz, Chloroform-d) δ 7.24 – 7.13 (m, 9H, 9xArH), 7.12 – 6.75 (m, 5H, 4xArH, NH), 6.48 (dd, J = 103.3, 7.8 Hz, 1H, NH), 4.99 (dd, J = 43.9, 8.4 Hz, 1H, NH), 4.81 – 4.60 (m, 2H, CHP, CH), 3.86 – 3.71 (m, 1H, CH), 3.22 – 2.85 (m, 2H, CH$_2$), 2.49 – 2.41 (m, 6H, 2xSCH$_3$), 2.41 – 2.24 (m, 1H, CH), 1.98 – 1.81 (m, 1H, CH), 1.38 (d, J = 2.2 Hz, 9H, Boc), 1.05 – 0.88 (m, 6H, 2xCH$_3$), 0.78 – 0.63 (m, 6H, 2xCH$_3$).
HRMS: $C_{37}H_{50}N_3O_7PS_2$ [M+Na]+ calc. 766.2725 found 766.2735
HPLC: purity >99%; retention time 19.783 min

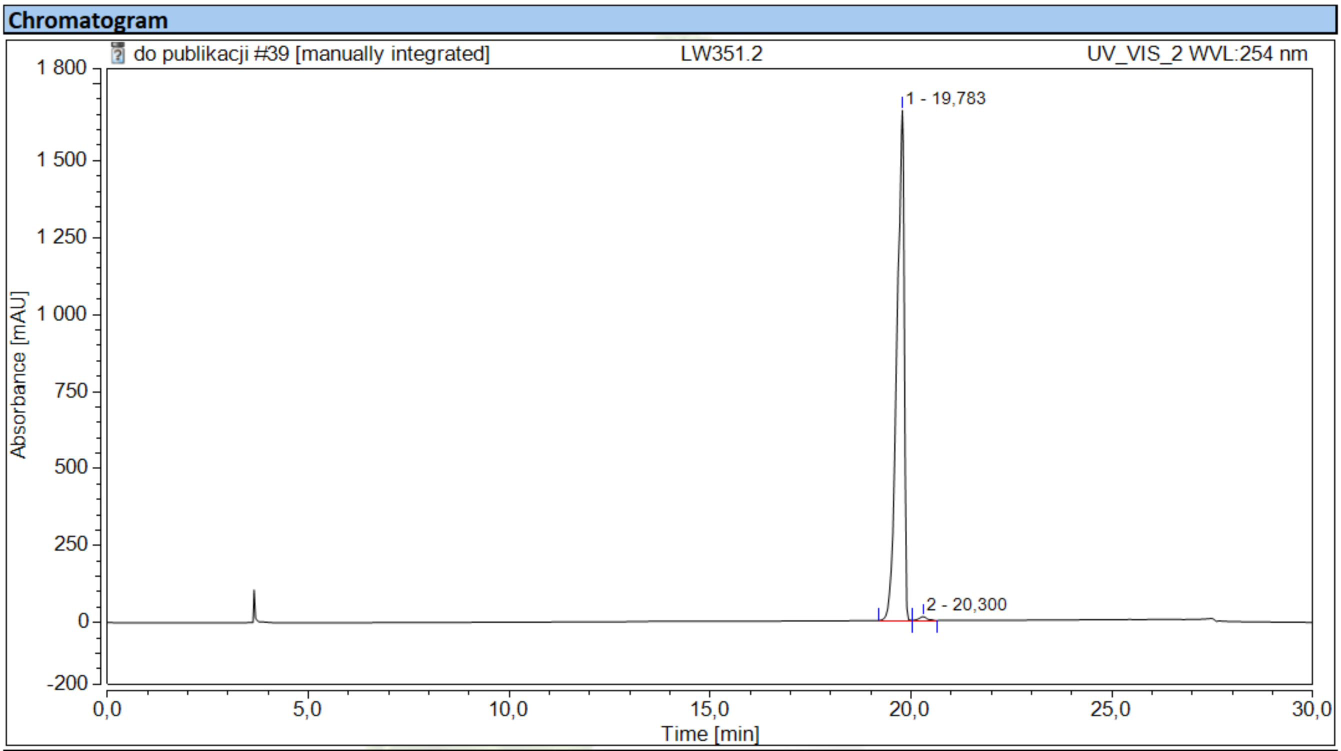

Integration Results

No.	Peak Name	Retention Time min	Area mAU*min	Height mAU	Relative Area %	Relative Height %	Amount n.a.
1		19,783	350,053	1656,937	99,17	99,23	n.a.
2		20,300	2,921	12,863	0,83	0,77	n.a.
**Total:**			**352,975**	**1669,799**	**100,00**	**100,00**	

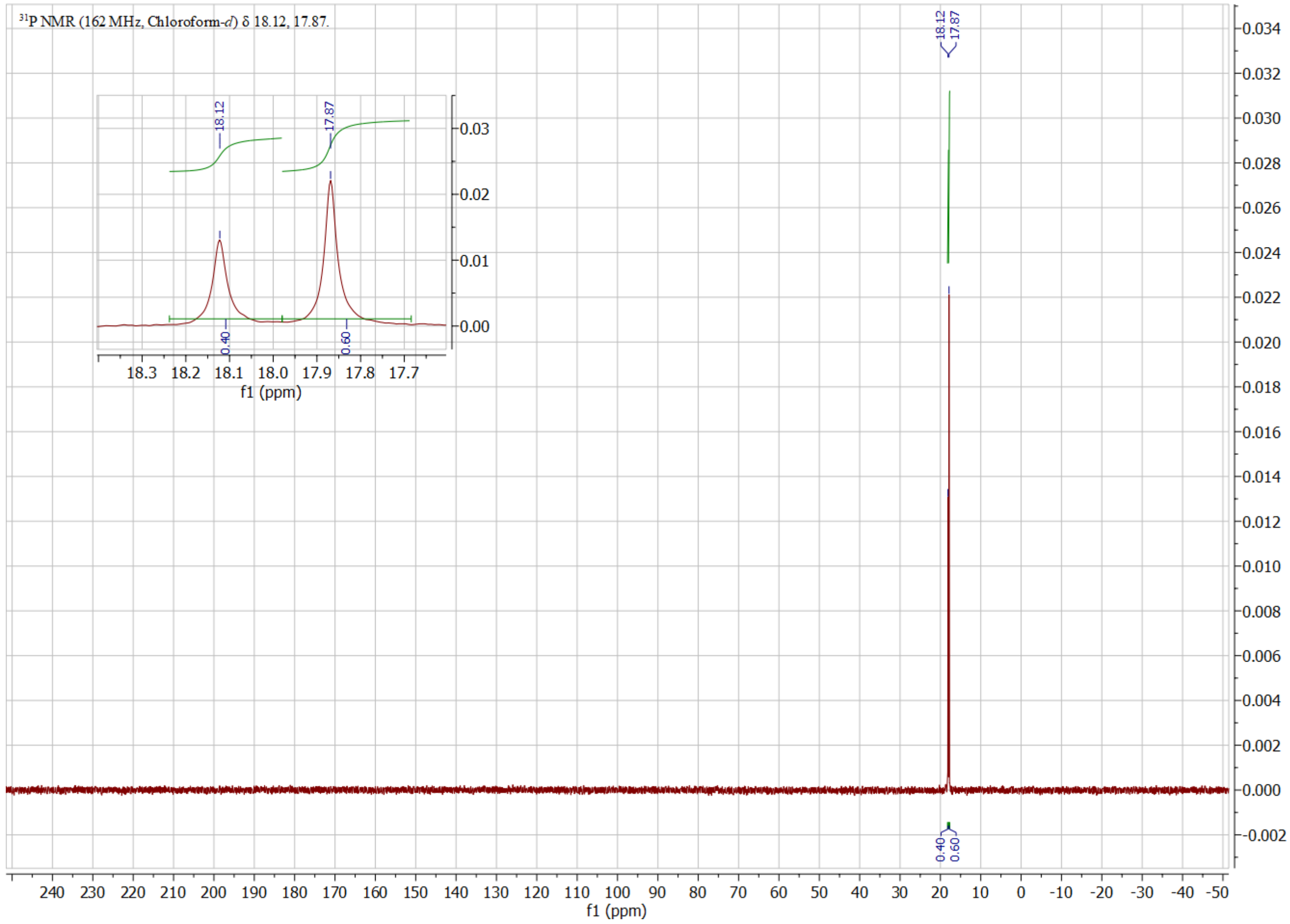
31P NMR (162 MHz, Chloroform-d) δ 18.12, 17.87.
18.12
17.87
0.40
0.60
f1 (ppm)

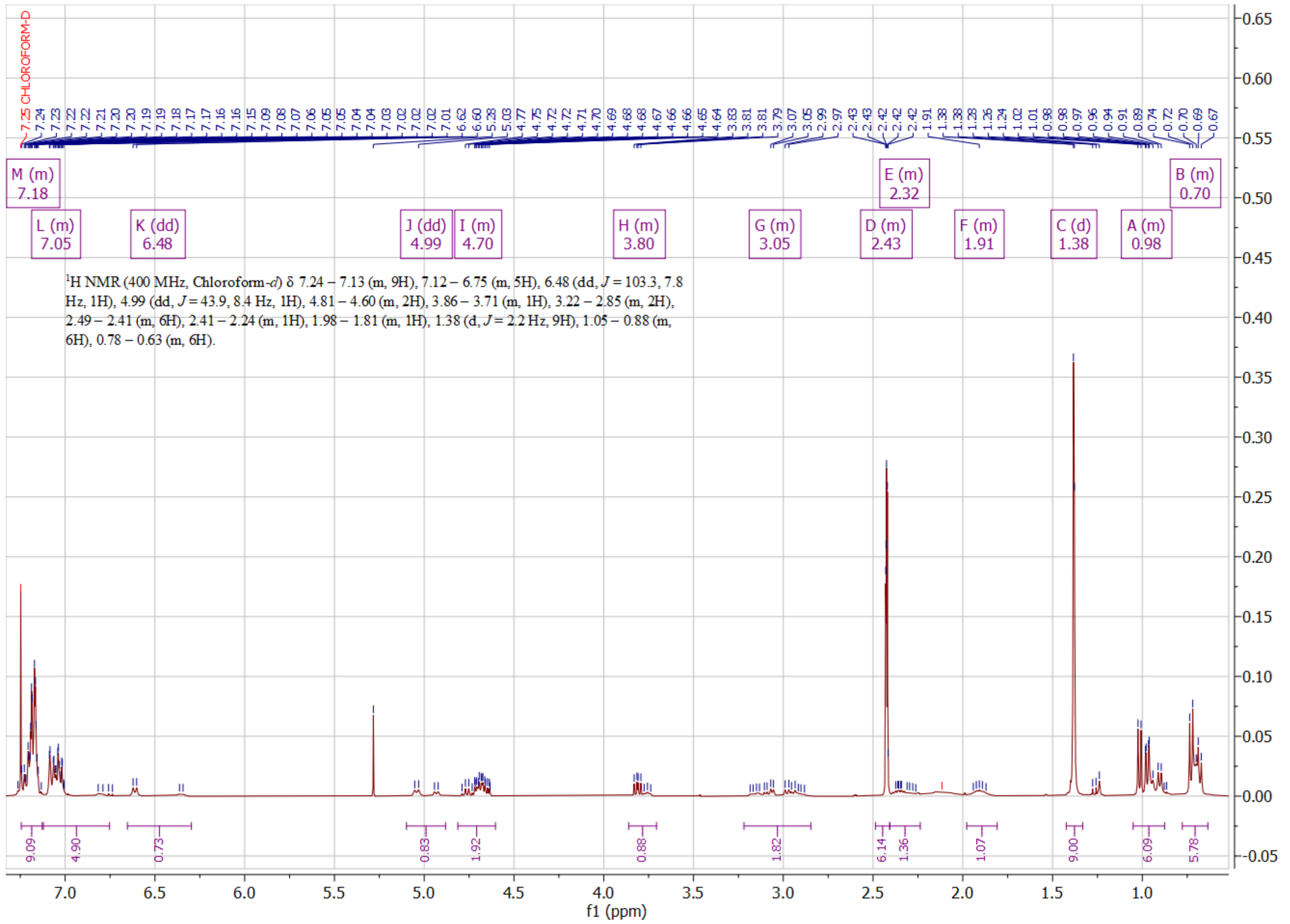
CHLOROFORM-D
M (m) 7.18
L (m) 7.05
K (dd) 6.48
J (dd) 4.99
I (m) 4.70
H (m) 3.80
G (m) 3.05
E (m) 2.32
D (m) 2.43
F (m) 1.91
C (d) 1.38
A (m) 0.98
B (m) 0.70
1H NMR (400 MHz, Chloroform-d) δ 7.24 – 7.13 (m, 9H), 7.12 – 6.75 (m, 5H), 6.48 (dd, J = 103.3, 7.8 Hz, 1H), 4.99 (dd, J = 43.9, 8.4 Hz, 1H), 4.81 – 4.60 (m, 2H), 3.86 – 3.71 (m, 1H), 3.22 – 2.85 (m, 2H), 2.49 – 2.41 (m, 6H), 2.41 – 2.24 (m, 1H), 1.98 – 1.81 (m, 1H), 1.38 (d, J = 2.2 Hz, 9H), 1.05 – 0.88 (m, 6H), 0.78 – 0.63 (m, 6H).
9.09
4.90
0.73
0.83
1.92
0.88
1.82
6.14
1.36
1.07
9.00
6.09
5.78
f1 (ppm)

(40) Boc-L-Val-L-Cha- Val^{P}(OPh-4-SCH$_3$)$_2$

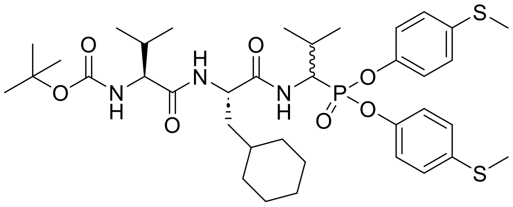

Name: *tert*-butyl ((2S)-1-(((2S)-1-((1-(bis(4-(methylthio)phenoxy)phosphoryl)-2-methylpropyl)amino)-3-cyclohexyl-1-oxopropan-2-yl)amino)-3-methyl-1-oxobutan-2-yl)carbamate

^{31}P NMR (162 MHz, Chloroform-d) δ 18.36 (s, 56%), 18.17 (s, 44%).

^{1}H NMR (400 MHz, Chloroform-d) δ 7.21 – 7.13 (m, 4H, 4xArH), 7.13 – 7.01 (m, 4H, 4xArH), 6.87 (d, *J* = 10.4 Hz, 1H, NH), 6.43 (d, *J* = 7.2 Hz, 1H, NH), 5.03 (dd, *J* = 50.2, 8.3 Hz, 1H, NH), 4.76 – 4.64 (m, 1H, CHP), 4.57 – 4.45 (m, 1H, CH), 3.92 – 3.81 (m, 1H, CH), 2.46 – 2.41 (m, 6H, 2xSCH$_3$), 2.41 – 2.31 (m, 1H, CH), 2.17 – 2.03 (m, 1H, CH), 1.76 – 1.47 (m, 7H, 3xCH$_2$, CH), 1.41 (d, *J* = 6.7 Hz, 9H, Boc), 1.34 – 1.23 (m, 2H, CH$_2$), 1.13 – 1.00 (m, 8H, 2xCH$_3$, CH$_2$), 0.93 – 0.80 (m, 8H, 2xCH$_3$, CH$_2$).

HRMS: $C_{37}H_{56}N_3O_7PS_2$ [M+Na]$^+$ calc. 772.3195 found 772.3164

HPLC: purity >98%; retention time 20.510 and 20.840 min

Chromatogram

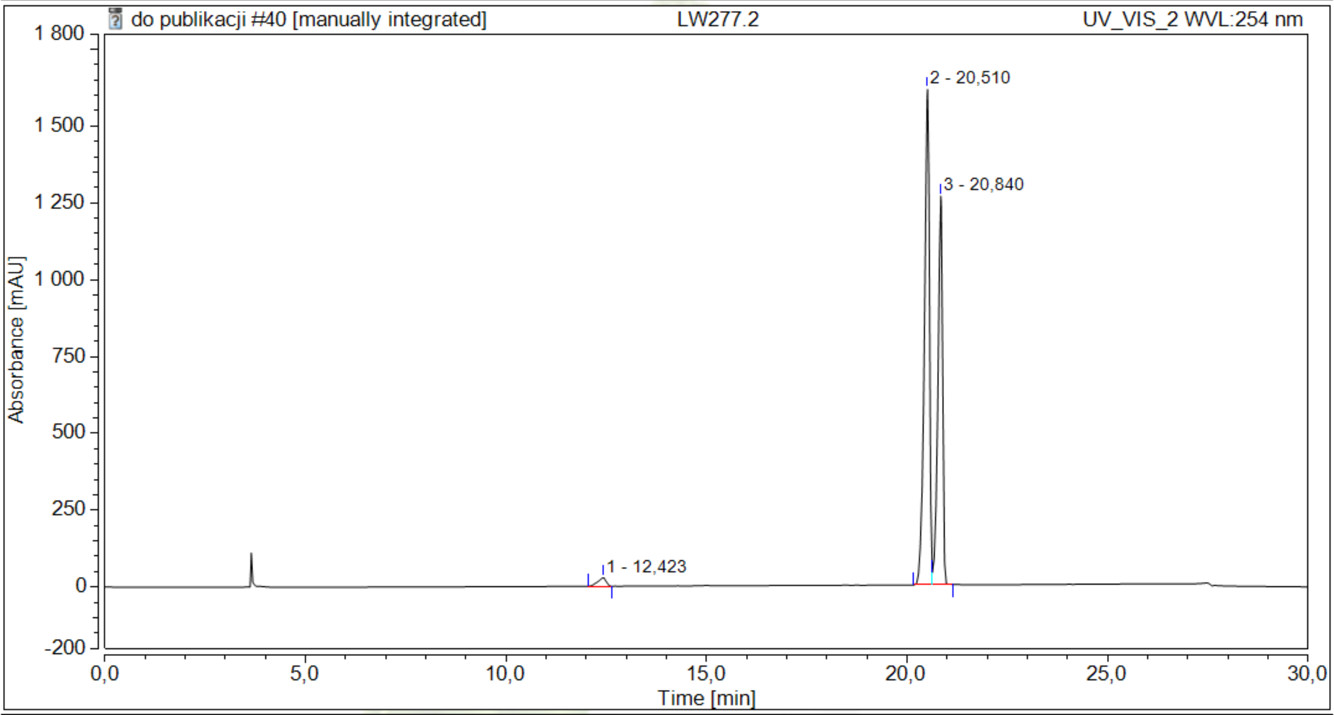

Integration Results

No.	Peak Name	Retention Time min	Area mAU*min	Height mAU	Relative Area %	Relative Height %	Amount n.a.
1		12,423	6,650	28,147	1,62	0,97	n.a.
2		20,510	235,122	1612,276	57,11	55,50	n.a.
3		20,840	169,933	1264,335	41,28	43,53	n.a.
**Total:**			**411,706**	**2904,757**	**100,00**	**100,00**	

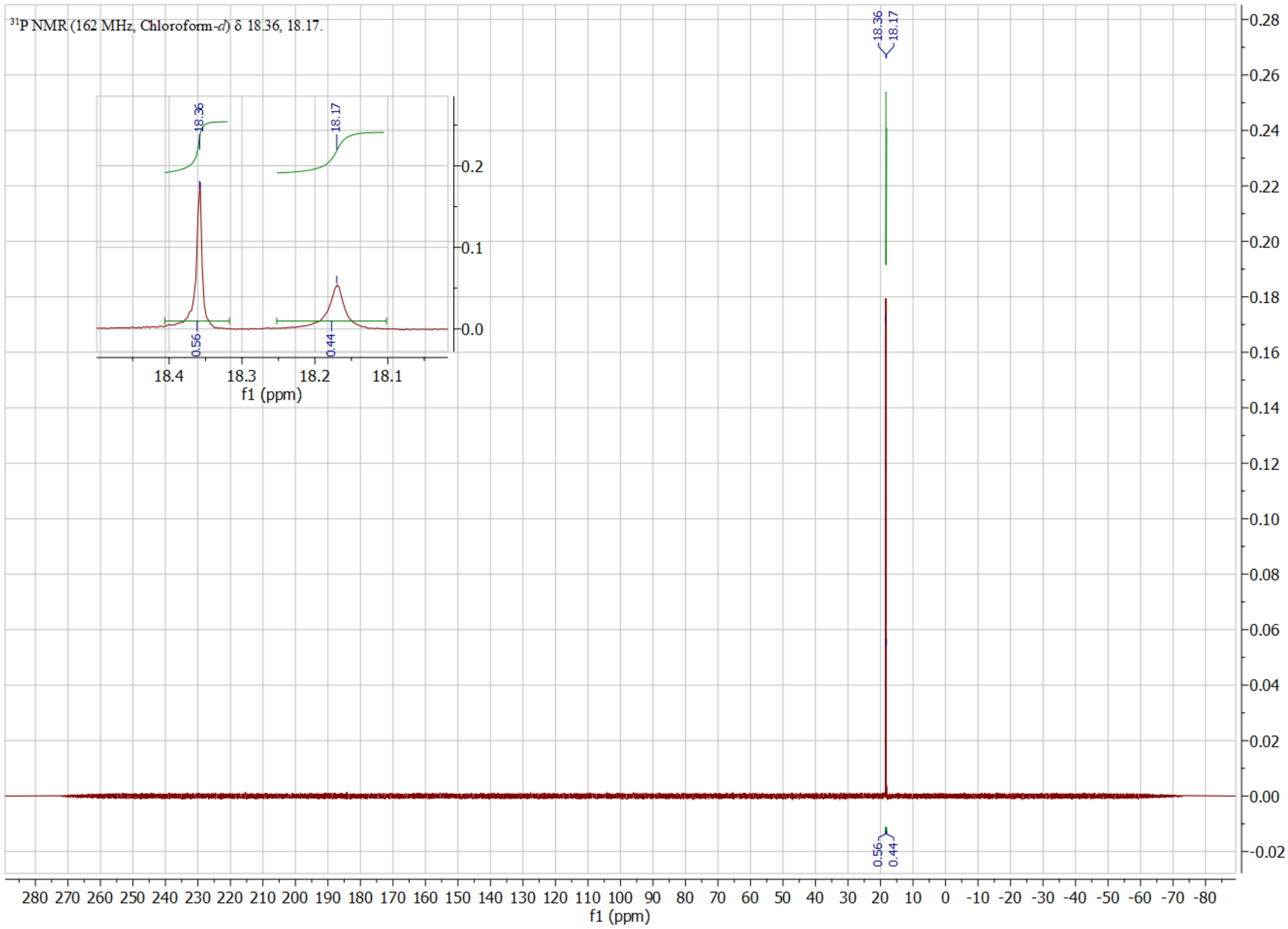
31P NMR (162 MHz, Chloroform-d) δ 18.36, 18.17.
18.36
18.17
0.56
0.44
f1 (ppm)

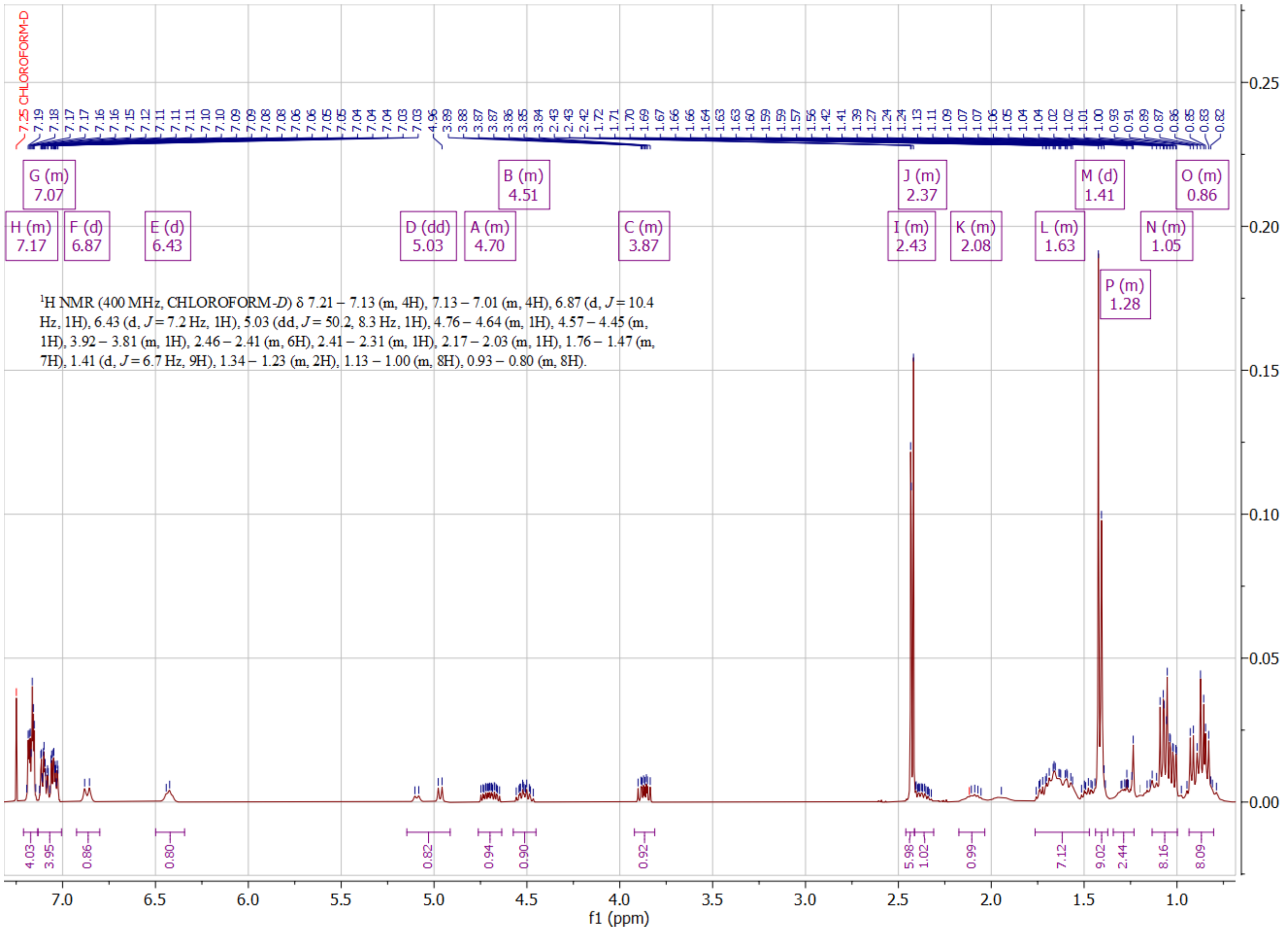
7.25 CHLOROFORM-D
G (m) 7.07
H (m) 7.17
F (d) 6.87
E (d) 6.43
D (dd) 5.03
A (m) 4.70
B (m) 4.51
C (m) 3.87
J (m) 2.37
I (m) 2.43
K (m) 2.08
L (m) 1.63
M (d) 1.41
P (m) 1.28
N (m) 1.05
O (m) 0.86
1H NMR (400 MHz, CHLOROFORM-D) δ 7.21 – 7.13 (m, 4H), 7.13 – 7.01 (m, 4H), 6.87 (d, J = 10.4 Hz, 1H), 6.43 (d, J = 7.2 Hz, 1H), 5.03 (dd, J = 50.2, 8.3 Hz, 1H), 4.76 – 4.64 (m, 1H), 4.57 – 4.45 (m, 1H), 3.92 – 3.81 (m, 1H), 2.46 – 2.41 (m, 6H), 2.41 – 2.31 (m, 1H), 2.17 – 2.03 (m, 1H), 1.76 – 1.47 (m, 7H), 1.41 (d, J = 6.7 Hz, 9H), 1.34 – 1.23 (m, 2H), 1.13 – 1.00 (m, 8H), 0.93 – 0.80 (m, 8H).
f1 (ppm)

(41) Boc-L-Val-D-Igl-Val^{P}(OPh-4-SCH$_3$)$_2$

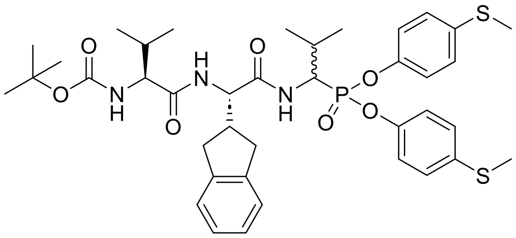

Name: *tert*-butyl ((2S)-1-(((1S)-2-((1-(bis(4-(methylthio)phenoxy)phosphoryl)-2-methylpropyl)amino)-1-(2,3-dihydro-1H-inden-2-yl)-2-oxoethyl)amino)-3-methyl-1-oxobutan-2-yl)carbamate

^{31}P NMR (162 MHz, Chloroform-d) δ 18.32 (s, 42%), 18.27 (s, 58%).

^{1}H NMR (400 MHz, Chloroform-d) δ 7.24 – 6.91 (m, 13H, 12xArH, NH), 6.64 (td, J = 9.1, 9.1 Hz, 1H, NH), 5.30 – 5.01 (m, 1H, NH), 4.82 – 4.59 (m, 2H, CHP, CH), 3.88 – 3.78 (m, 1H, CH), 3.13 – 2.88 (m, 3H, CH$_2$, CH), 2.82 – 2.64 (m, 2H, CH$_2$), 2.45 – 2.39 (m, 6H, 2xSCH$_3$), 2.40 – 2.24 (m, 1H, CH), 2.17 – 2.02 (m, 1H, CH), 1.41 (d, J = 15.9 Hz, 9H, Boc), 1.13 – 0.98 (m, 6H, 2xCH$_3$), 0.92 – 0.75 (m, 6H, 2xCH$_3$)

HRMS: $C_{39}H_{52}N_3O_7PS_2$ [M+Na]$^+$ calc. 792.2882 found 792.2878

HPLC: purity >98%; retention time 19.860 and 20.143 min

Chromatogram

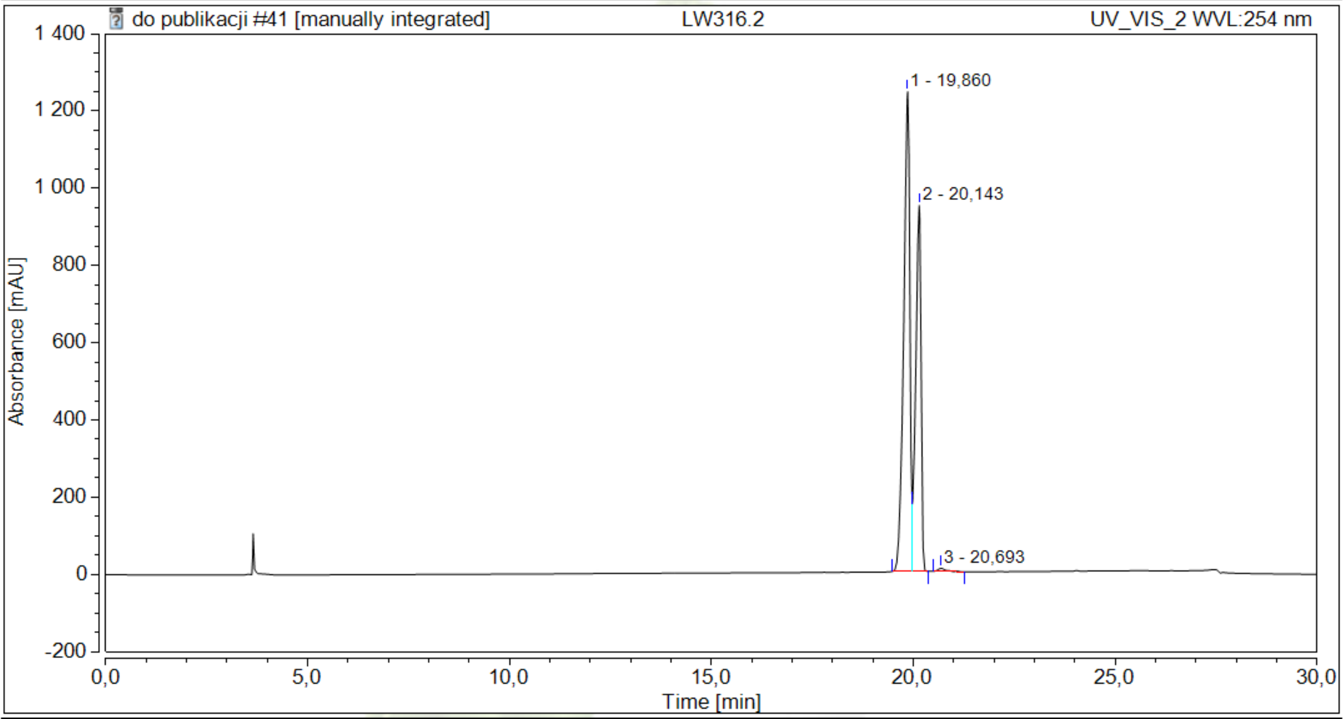

Integration Results

No.	Peak Name	Retention Time min	Area mAU*min	Height mAU	Relative Area %	Relative Height %	Amount n.a.
1		19,860	216,488	1241,757	59,59	56,51	n.a.
2		20,143	144,883	947,324	39,88	43,11	n.a.
3		20,693	1,897	8,412	0,52	0,38	n.a.
**Total:**			**363,267**	**2197,493**	**100,00**	**100,00**	

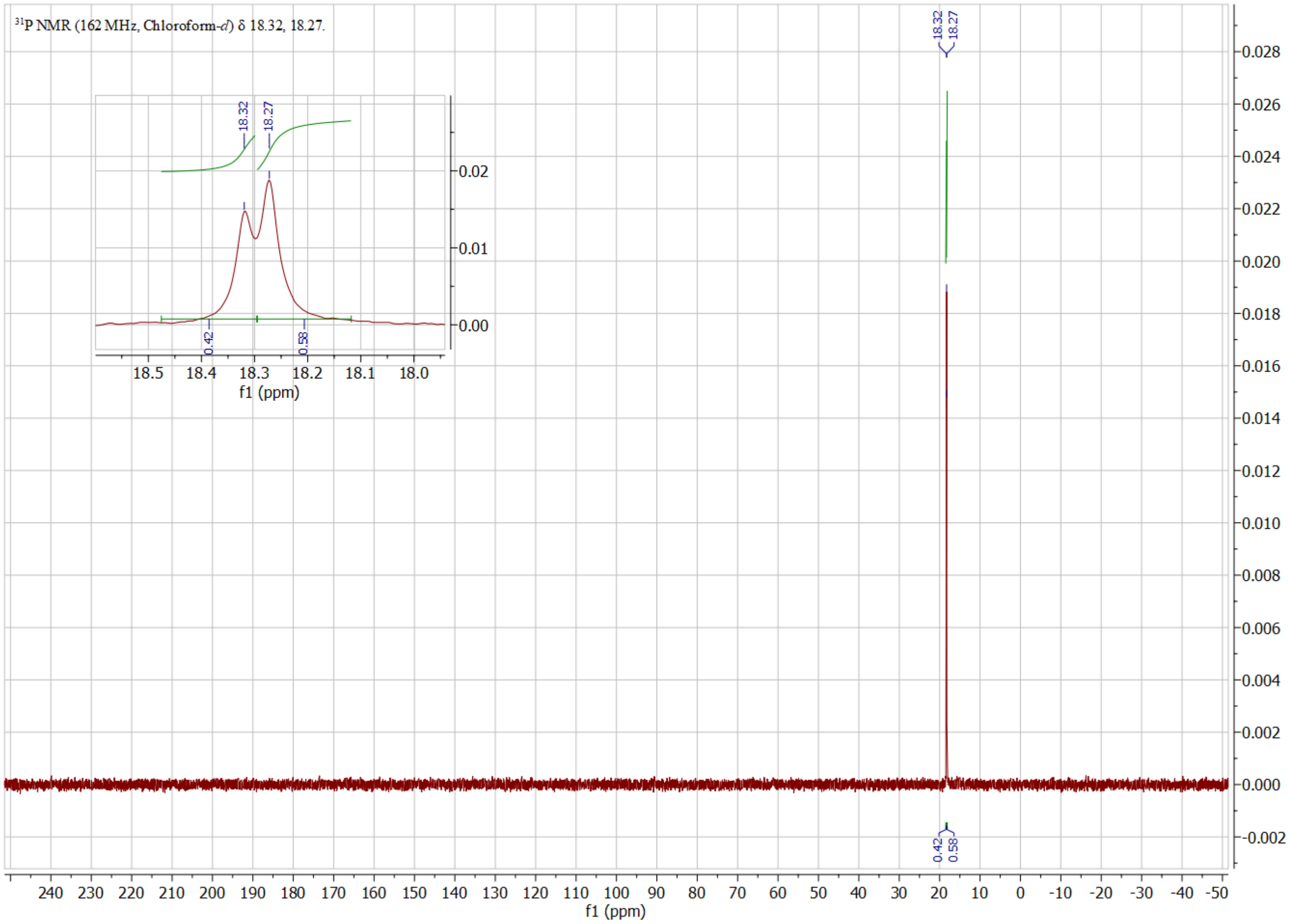
31P NMR (162 MHz, Chloroform-d) δ 18.32, 18.27.
18.32
18.27
0.42
0.58
f1 (ppm)

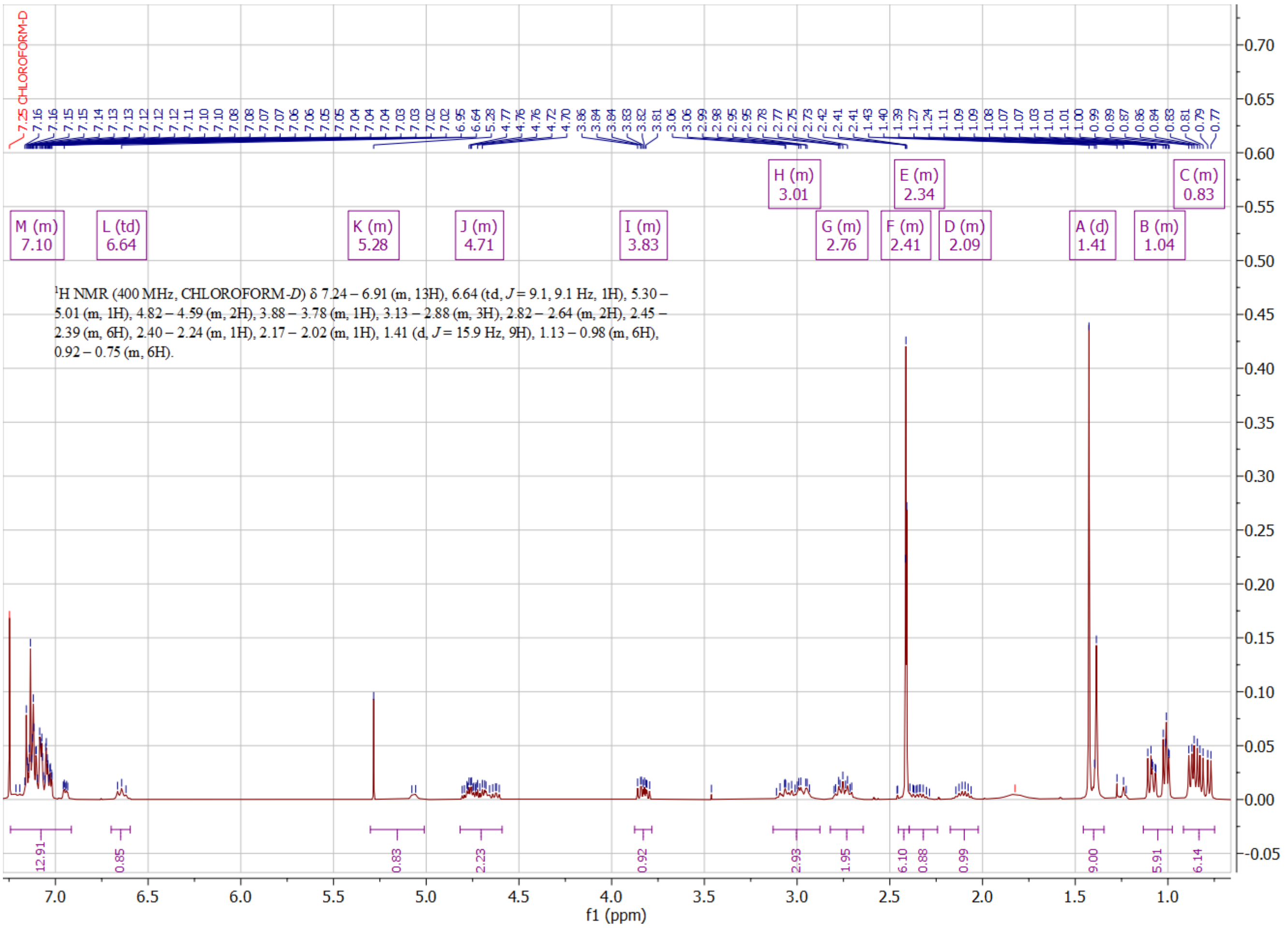
1H NMR (400 MHz, CHLOROFORM-D) δ 7.24 – 6.91 (m, 13H), 6.64 (td, J = 9.1, 9.1 Hz, 1H), 5.30 – 5.01 (m, 1H), 4.82 – 4.59 (m, 2H), 3.88 – 3.78 (m, 1H), 3.13 – 2.88 (m, 3H), 2.82 – 2.64 (m, 2H), 2.45 – 2.39 (m, 6H), 2.40 – 2.24 (m, 1H), 2.17 – 2.02 (m, 1H), 1.41 (d, J = 15.9 Hz, 9H), 1.13 – 0.98 (m, 6H), 0.92 – 0.75 (m, 6H).
7.25 CHLOROFORM-D
M (m) 7.10
L (td) 6.64
K (m) 5.28
J (m) 4.71
I (m) 3.83
H (m) 3.01
G (m) 2.76
F (m) 2.41
E (m) 2.34
D (m) 2.09
A (d) 1.41
B (m) 1.04
C (m) 0.83
f1 (ppm)

(42) Boc-L-Val-D-Igl-Val^P(OPh-4-SCH$_3$)$_2$

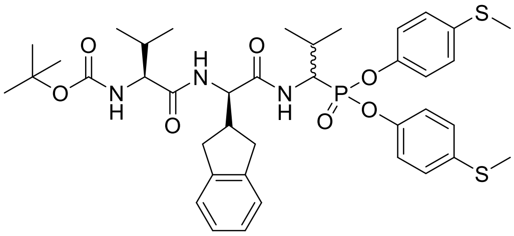

Name: *tert*-butyl ((2S)-1-(((1R)-2-((1-(bis(4-(methylthio)phenoxy)phosphoryl)-2-methylpropyl)amino)-1-(2,3-dihydro-1H-inden-2-yl)-2-oxoethyl)amino)-3-methyl-1-oxobutan-2-yl)carbamate

^{31}P NMR 162 MHz, Chloroform-d) δ 18.41 (s, 60%), 18.14 (s, 40%).

^{1}H NMR (400 MHz, Chloroform-d) δ 7.25 – 7.00 (m, 13H, 12xArH, NH), 6.81 (dd, J = 122.2, 6.1 Hz, 1H, NH), 5.09 (dd, J = 97.1, 6.7 Hz, 1H, NH), 4.79 – 4.67 (m, 1H, CHP), 4.65 – 4.55 (m, 1H, CH), 3.95 – 3.80 (m, 1H, CH), 3.14 – 2.83 (m, 3H, CH$_2$, CH), 2.83 – 2.63 (m, 2H, CH$_2$), 2.47 – 2.40 (m, 6H, 2xSCH$_3$), 2.40 – 2.22 (m, 1H, CH), 2.16 – 1.96 (m, 1H, CH), 1.38 (d, J = 15.3 Hz, 9H, Boc), 1.11 – 0.95 (m, 6H, 2xCH$_3$), 0.92 – 0.70 (m, 6H, 2xCH$_3$).

HRMS: $C_{39}H_{52}N_3O_7PS_2$ [M+Na]$^+$ calc. 792.2882 found 792.2873

HPLC: purity >98%; retention time 19.963 and 20.223 min

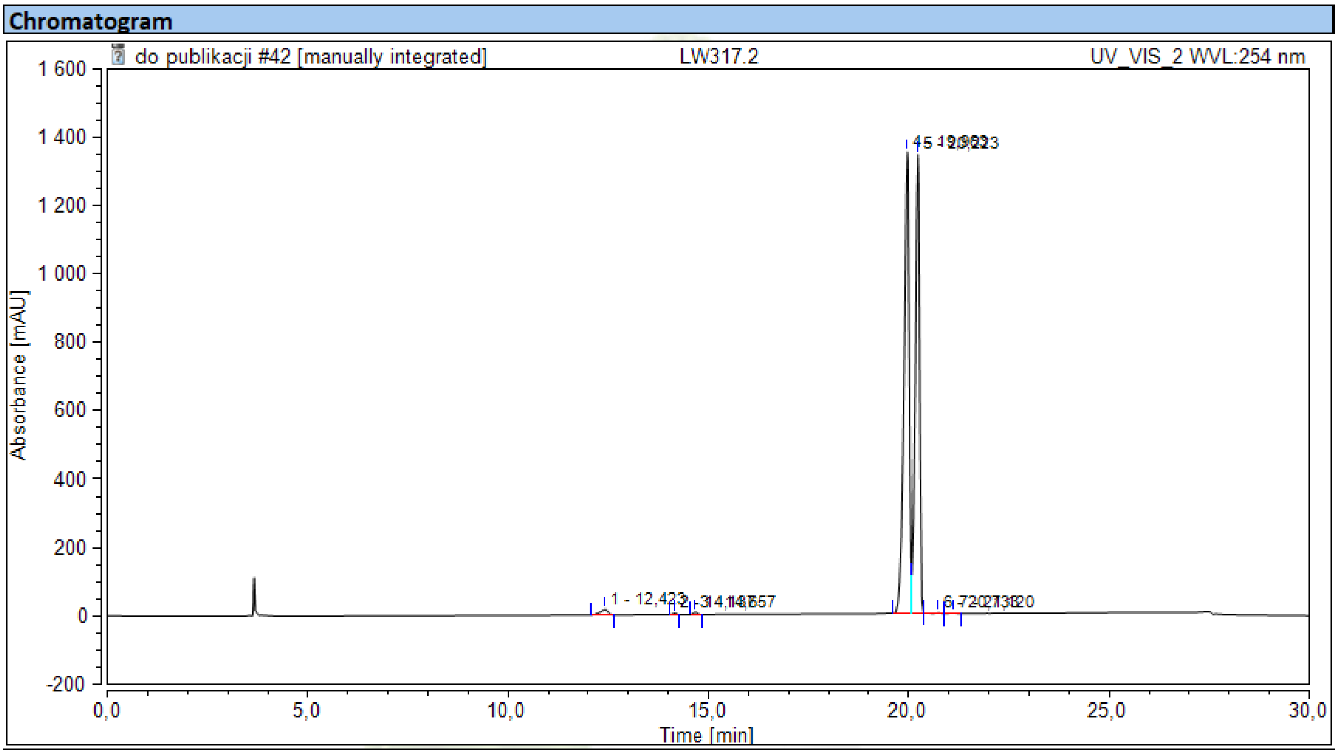

Integration Results

No.	Peak Name	Retention Time min	Area mAU*min	Height mAU	Relative Area %	Relative Height %	Amount n.a.
1		12,423	3,662	16,318	0,95	0,60	n.a.
2		14,137	0,520	4,604	0,14	0,17	n.a.
3		14,657	0,907	6,915	0,24	0,25	n.a.
4		19,963	206,125	1350,546	53,74	49,58	n.a.
5		20,223	171,893	1343,027	44,82	49,30	n.a.
6		20,733	0,273	1,536	0,07	0,06	n.a.
7		21,120	0,182	1,140	0,05	0,04	n.a.
**Total:**			**383,560**	**2724,086**	**100,00**	**100,00**	

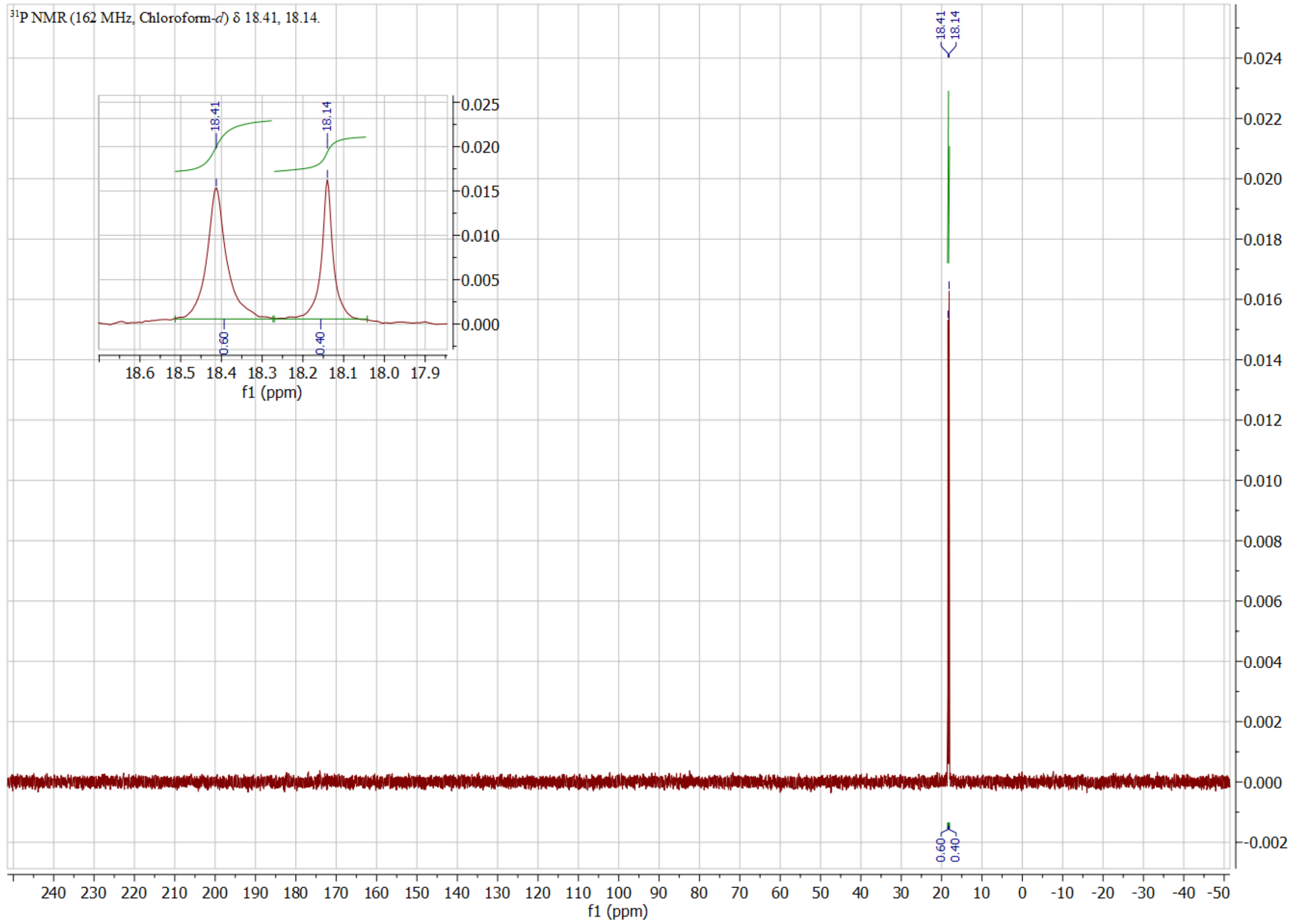
31P NMR (162 MHz, Chloroform-d) δ 18.41, 18.14.
f1 (ppm)

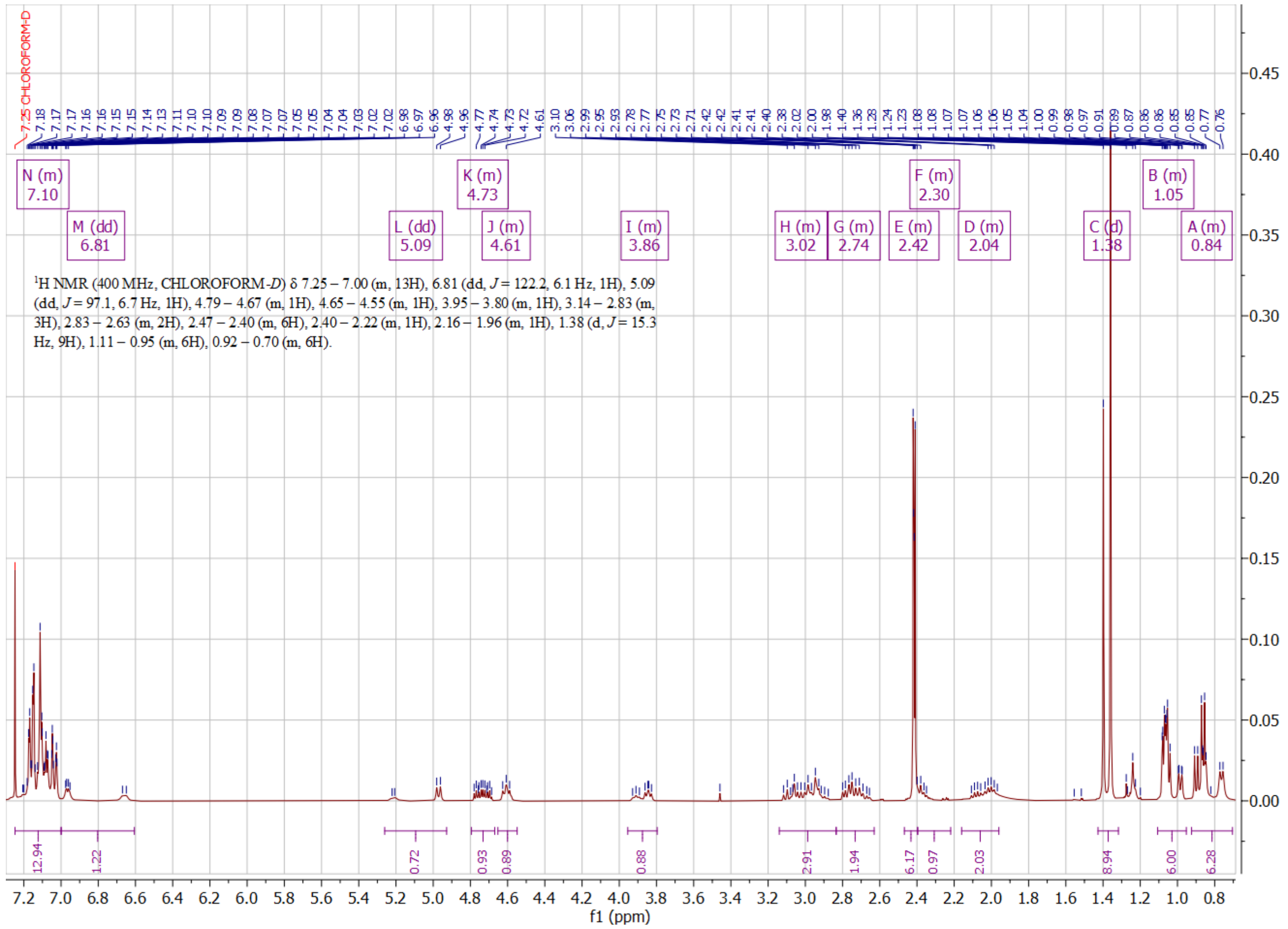
1H NMR (400 MHz, CHLOROFORM-D) δ 7.25 – 7.00 (m, 13H), 6.81 (dd, J = 122.2, 6.1 Hz, 1H), 5.09 (dd, J = 97.1, 6.7 Hz, 1H), 4.79 – 4.67 (m, 1H), 4.65 – 4.55 (m, 1H), 3.95 – 3.80 (m, 1H), 3.14 – 2.83 (m, 3H), 2.83 – 2.63 (m, 2H), 2.47 – 2.40 (m, 6H), 2.40 – 2.22 (m, 1H), 2.16 – 1.96 (m, 1H), 1.38 (d, J = 15.3 Hz, 9H), 1.11 – 0.95 (m, 6H), 0.92 – 0.70 (m, 6H).
f1 (ppm)

(43) Boc-L-Val-L-Trp- Val^P(OPh-4-SCH$_3$)$_2$

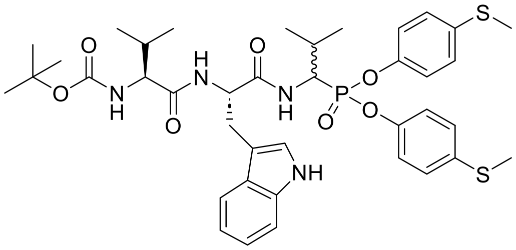

Name: *tert*-butyl ((2S)-1-(((2S)-1-((1-(bis(4-(methylthio)phenoxy)phosphoryl)-2-methylpropyl)amino)-3-(1H-indol-3-yl)-1-oxopropan-2-yl)amino)-3-methyl-1-oxobutan-2-yl)carbamate

^{31}P NMR (162 MHz, Chloroform-d) δ 17.96 (s, 48%), 17.74 (s, 52%).

^{1}H NMR (400 MHz, Chloroform-d) δ 8.07 (d, J = 33.2 Hz, 1H, NH), 7.71 – 7.59 (m, 1H, NH), 7.35 – 7.26 (m, 1H, NH), 7.23 – 7.09 (m, 6H, 6xArH), 7.09 – 6.68 (m, 7H, 6xArH, CH), 4.95 – 4.77 (m, 2H, CHP, NH), 4.75 – 4.63 (m, 1H, CH), 3.94 – 3.81 (m, 1H, CH), 3.34 – 3.07 (m, 2H, CH$_2$), 2.48 – 2.35 (m, 6H, 2xSCH$_3$), 2.36 – 2.19 (m, 1H, CH), 2.18 – 1.99 (m, 1H, CH), 1.35 (d, J = 5.8 Hz, 9H, Boc), 0.99 – 0.61 (m, 12H, 4xCH$_3$).

HRMS: $C_{39}H_{51}N_4O_7PS_2$ [M+Na]$^+$ calc. 805.2834 found 805.2843

HPLC: purity >99%; retention time 18.993 min

Chromatogram

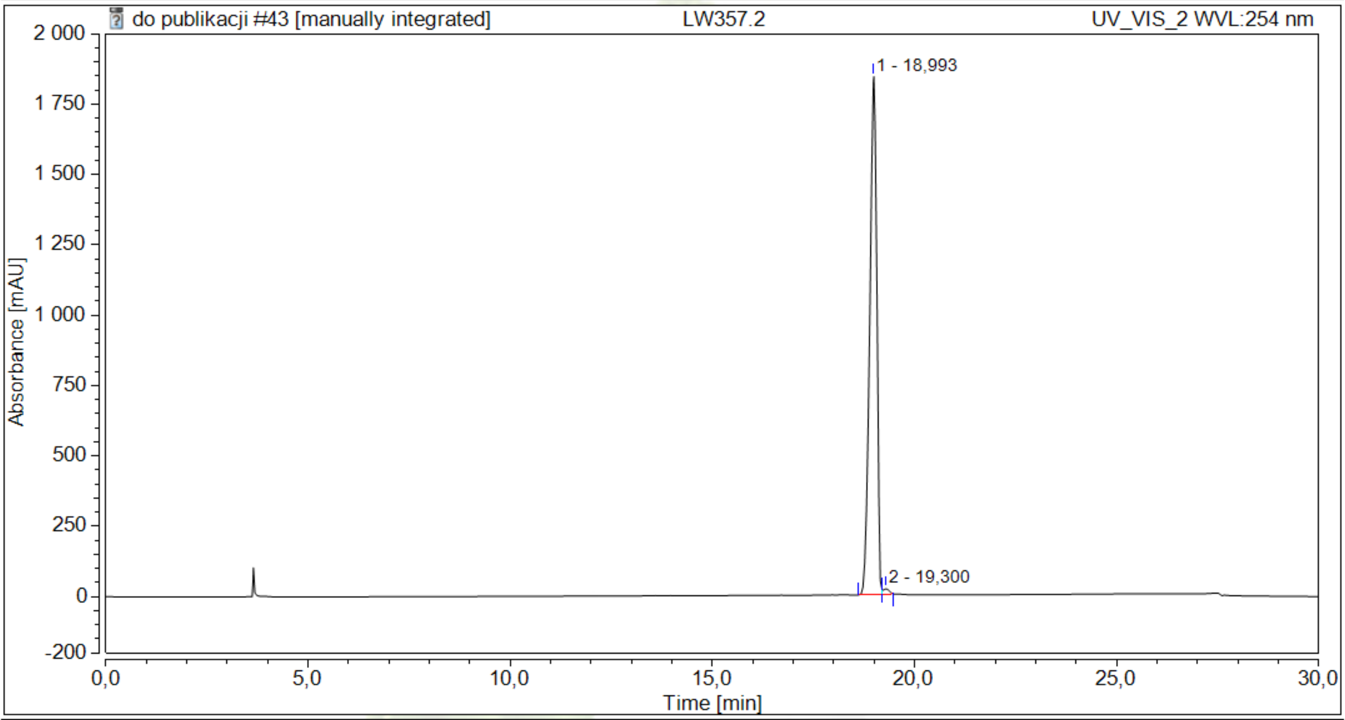

Integration Results

No.	Peak Name	Retention Time min	Area mAU*min	Height mAU	Relative Area %	Relative Height %	Amount n.a.
1		18,993	372,330	1839,923	99,15	98,97	n.a.
2		19,300	3,177	19,106	0,85	1,03	n.a.
**Total:**			**375,508**	**1859,029**	**100,00**	**100,00**	

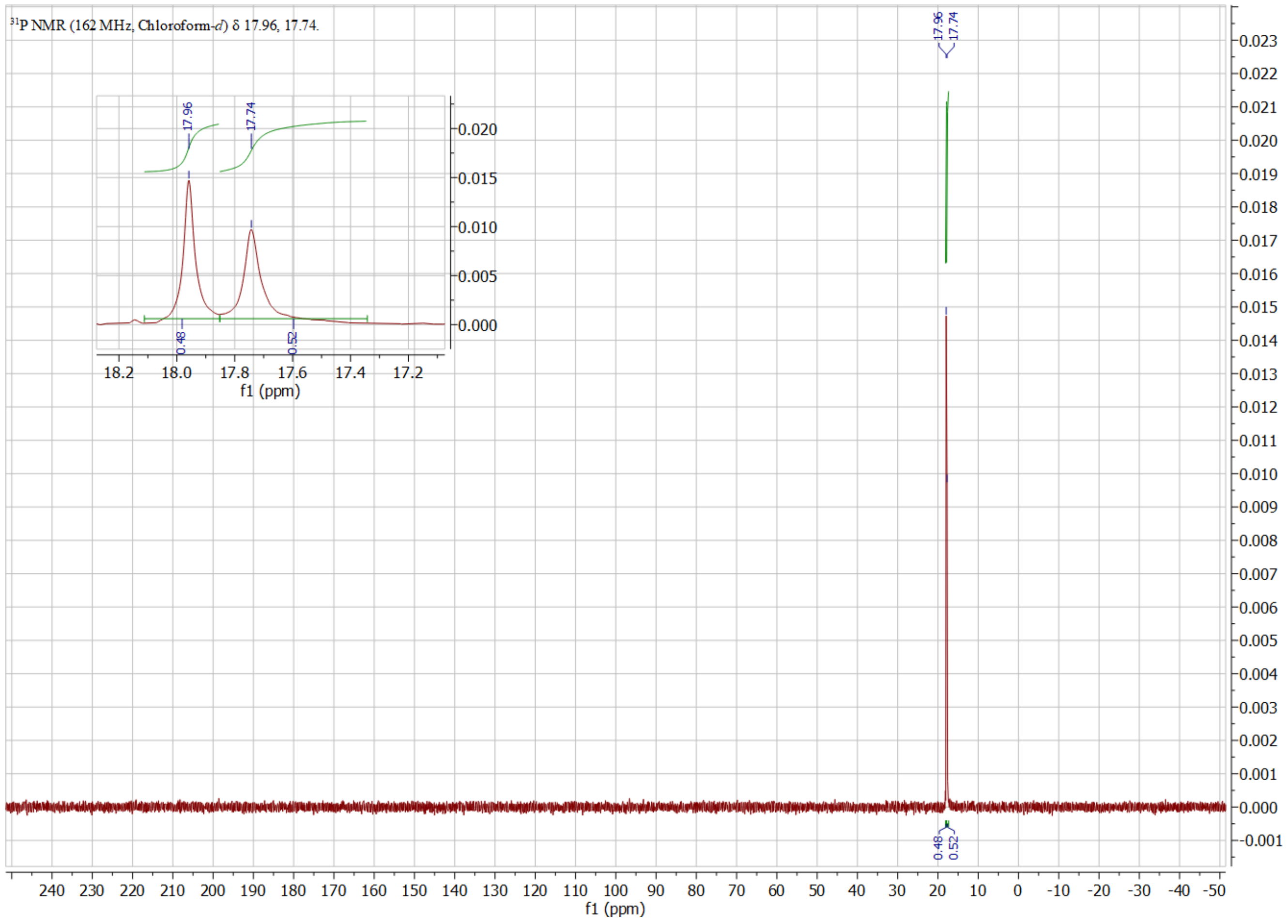
^{31}P NMR (162 MHz, Chloroform-*d*) δ 17.96, 17.74.
f1 (ppm)

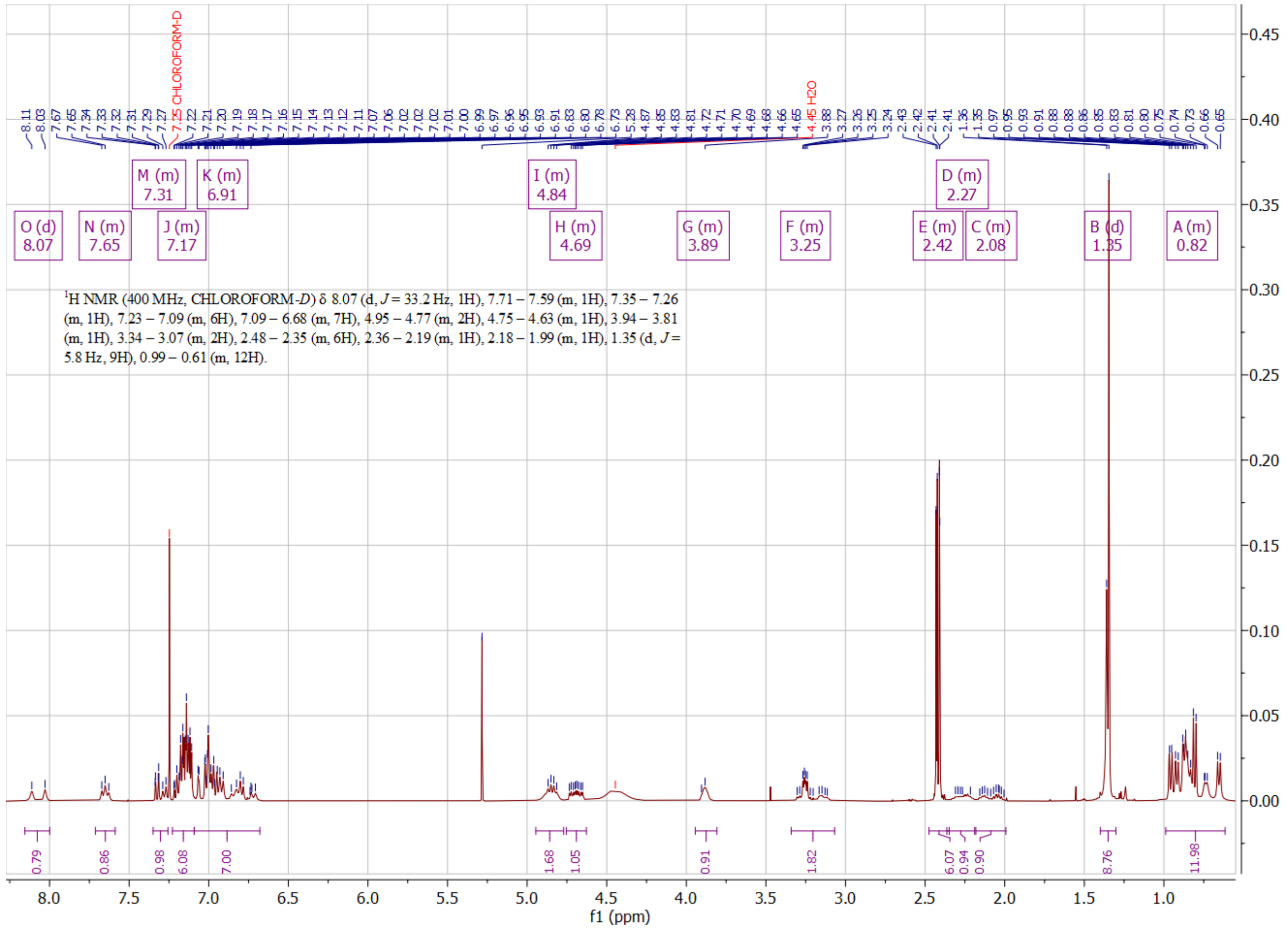
^{1}H NMR (400 MHz, CHLOROFORM-*D*) δ 8.07 (d, *J* = 33.2 Hz, 1H), 7.71 – 7.59 (m, 1H), 7.35 – 7.26 (m, 1H), 7.23 – 7.09 (m, 6H), 7.09 – 6.68 (m, 7H), 4.95 – 4.77 (m, 2H), 4.75 – 4.63 (m, 1H), 3.94 – 3.81 (m, 1H), 3.34 – 3.07 (m, 2H), 2.48 – 2.35 (m, 6H), 2.36 – 2.19 (m, 1H), 2.18 – 1.99 (m, 1H), 1.35 (d, *J* = 5.8 Hz, 9H), 0.99 – 0.61 (m, 12H).
CHLOROFORM-D
H2O
f1 (ppm)

(44) Boc-L-Val-D-Trp-Val^{P}(OPh-4-SCH$_3$)$_2$

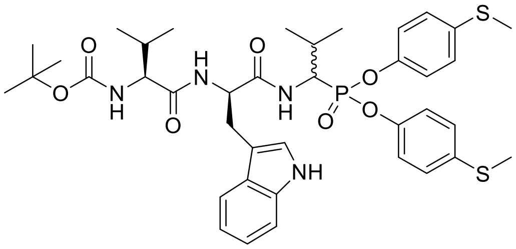

Name: *tert*-butyl ((2S)-1-(((2R)-1-((1-(bis(4-(methylthio)phenoxy)phosphoryl)-2-methylpropyl)amino)-3-(1H-indol-3-yl)-1-oxopropan-2-yl)amino)-3-methyl-1-oxobutan-2-yl)carbamate
^{31}P NMR (162 MHz, Chloroform-d) δ 18.03 (s, 46%), 17.80 (s, 54%).
^{1}H NMR (400 MHz, Chloroform-d) δ 8.06 (d, J = 38.8 Hz, 1H), 7.70 – 7.61 (m, 1H, NH), 7.36 – 7.25 (m, 1H, NH), 7.21 – 7.07 (m, 6H, 6xArH), 7.07 – 6.58 (m, 7H, 6xArH, CH), 5.11 (dd, J = 54.0, 8.4 Hz, 1H, NH), 4.95 – 4.81 (m, 1H, CH), 4.77 – 4.63 (m, 1H, CHP), 3.89 – 3.71 (m, 1H, CH), 3.33 – 3.06 (m, 2H, CH$_2$), 2.41 (dd, J = 10.6, 2.7 Hz, 6H, 2xSCH$_3$), 2.38 – 2.17 (m, 1H, CH), 2.01 – 1.78 (m, 1H, CH), 1.37 (d, J = 8.2 Hz, 9H, Boc), 1.01 – 0.63 (m, 12H, 4xCH$_3$).
HRMS: $C_{39}H_{51}N_4O_7PS_2$ [M+Na]+ calc. 805.2834 found 805.2846
HPLC: purity >99%; retention time 18.973 and 19.133 min

Chromatogram

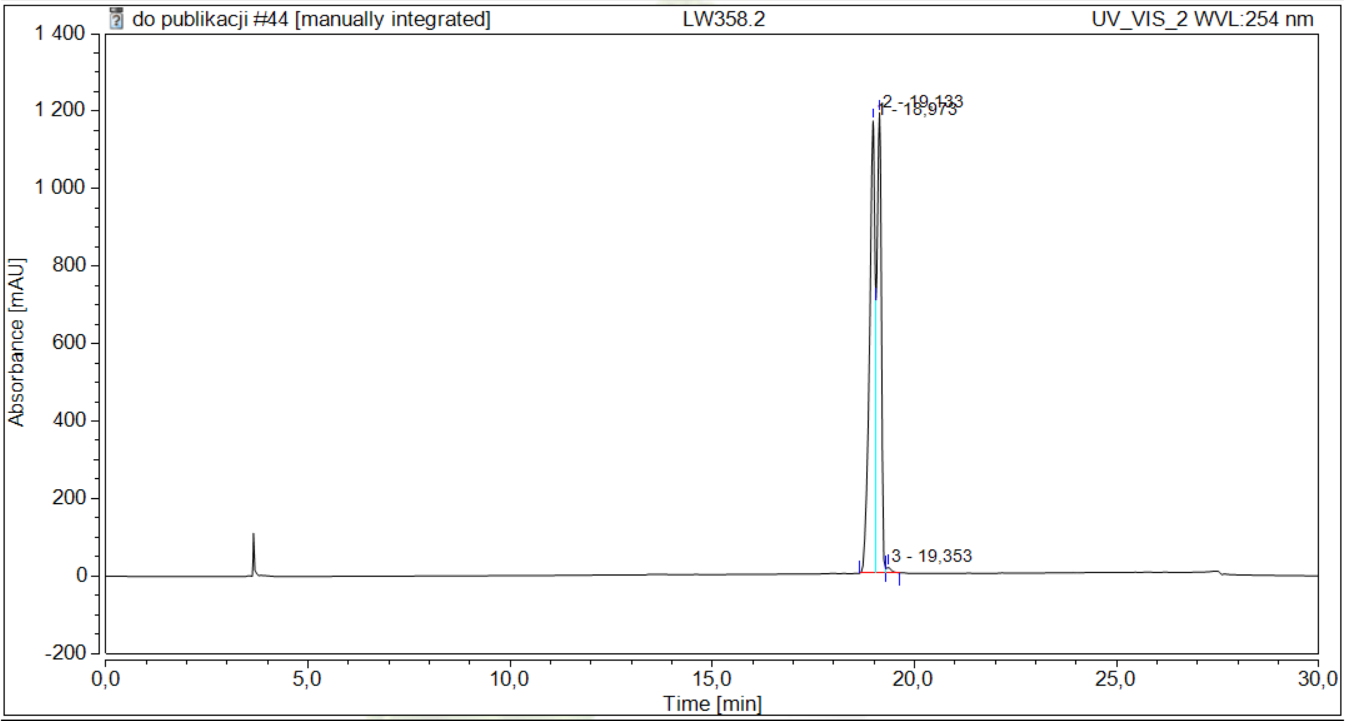

Integration Results

No.	Peak Name	Retention Time min	Area mAU*min	Height mAU	Relative Area %	Relative Height %	Amount n.a.
1		18,973	185,737	1167,393	55,31	49,26	n.a.
2		19,133	147,970	1188,364	44,06	50,14	n.a.
3		19,353	2,117	14,194	0,63	0,60	n.a.
**Total:**			**335,825**	**2369,951**	**100,00**	**100,00**	

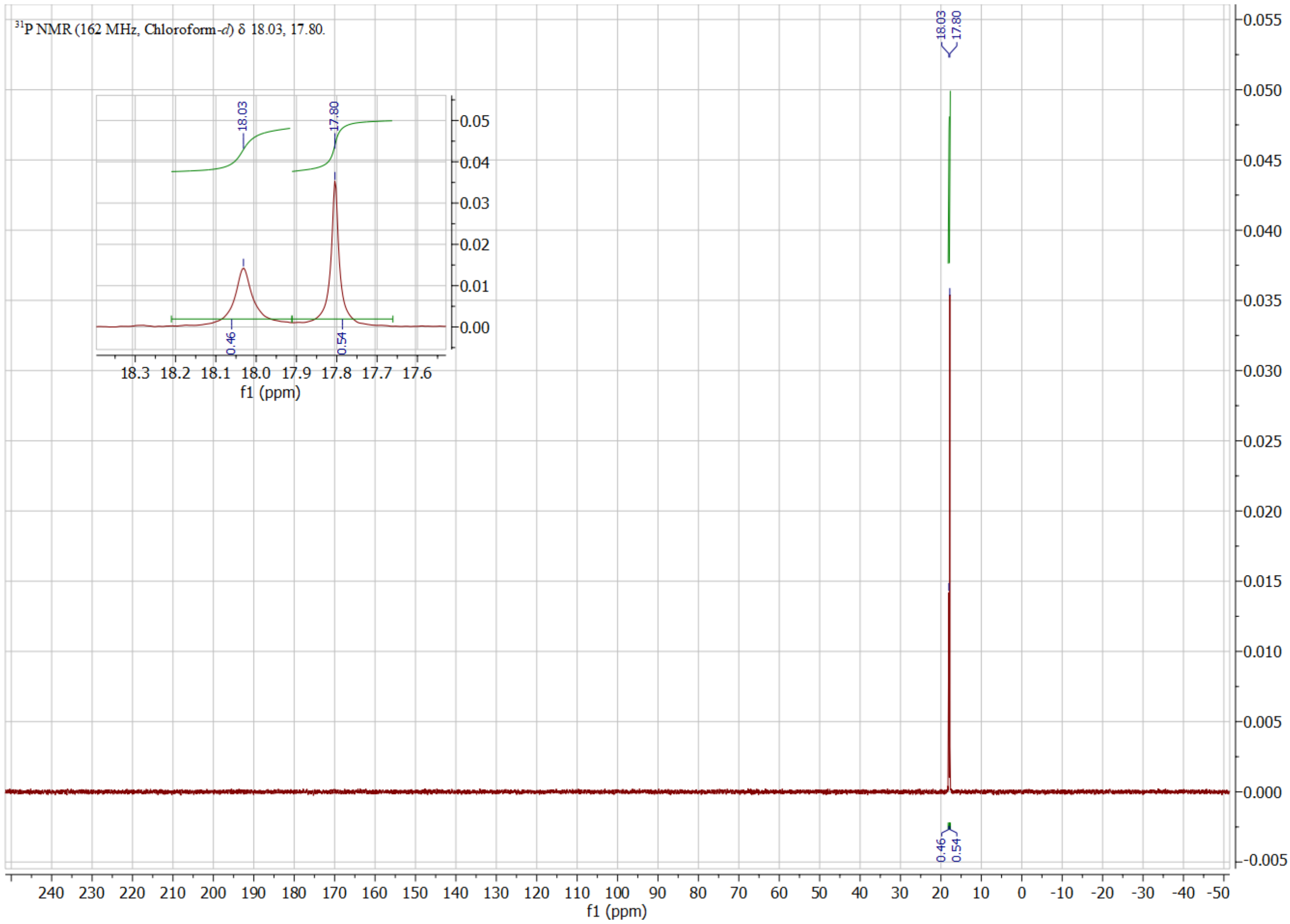
31P NMR (162 MHz, Chloroform-d) δ 18.03, 17.80.
f1 (ppm)

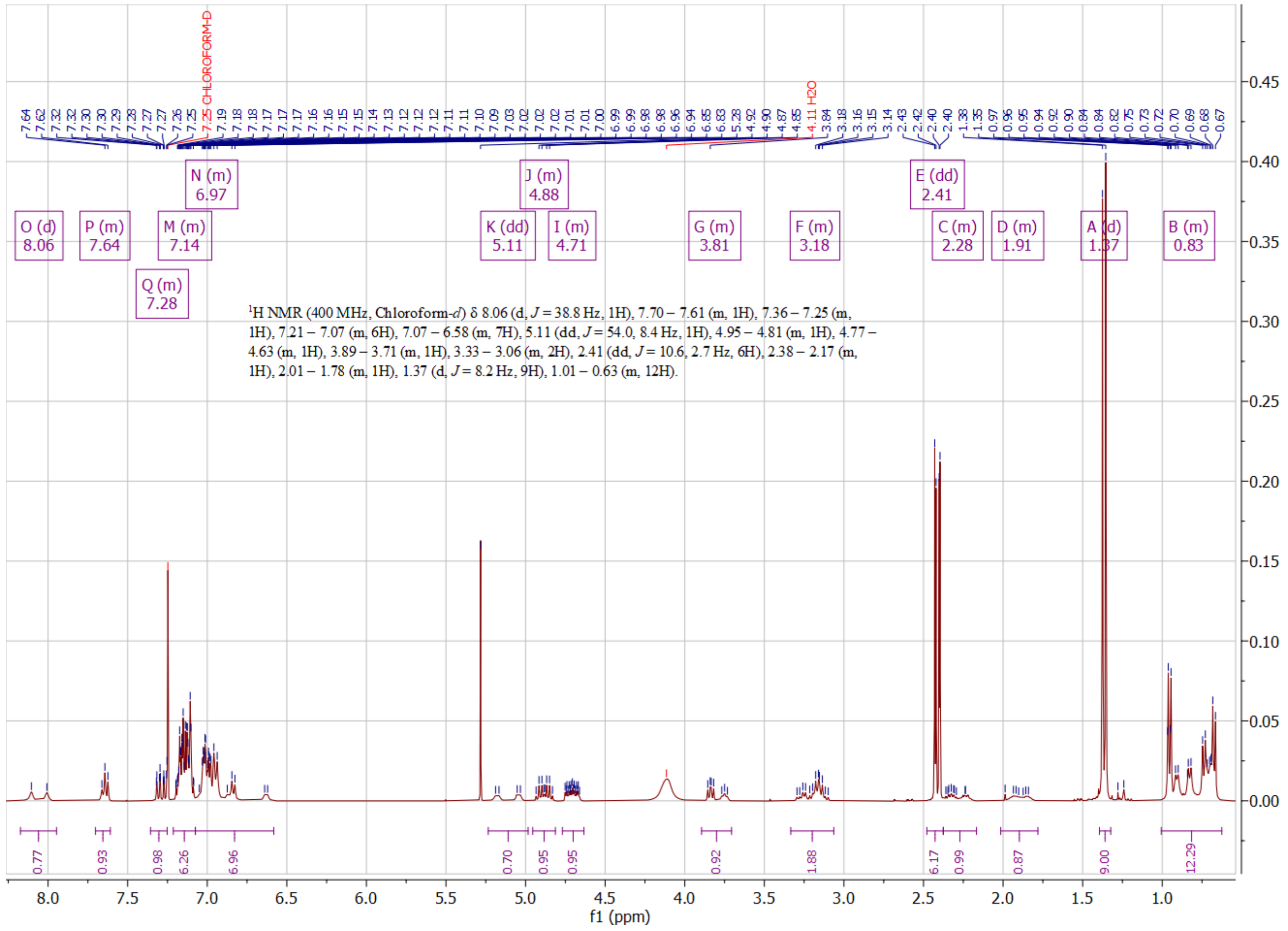
1H NMR (400 MHz, Chloroform-d) δ 8.06 (d, J = 38.8 Hz, 1H), 7.70 – 7.61 (m, 1H), 7.36 – 7.25 (m, 1H), 7.21 – 7.07 (m, 6H), 7.07 – 6.58 (m, 7H), 5.11 (dd, J = 54.0, 8.4 Hz, 1H), 4.95 – 4.81 (m, 1H), 4.77 – 4.63 (m, 1H), 3.89 – 3.71 (m, 1H), 3.33 – 3.06 (m, 2H), 2.41 (dd, J = 10.6, 2.7 Hz, 6H), 2.38 – 2.17 (m, 1H), 2.01 – 1.78 (m, 1H), 1.37 (d, J = 8.2 Hz, 9H), 1.01 – 0.63 (m, 12H).
f1 (ppm)

(45) Boc-L-Val-L-Asp(Bzl)-Val^{P}(OPh-4-SCH$_3$)$_2$

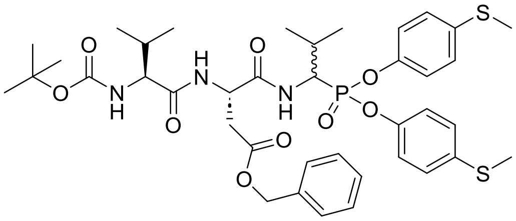

Name: benzyl (3S)-4-((1-(bis(4-(methylthio)phenoxy)phosphoryl)-2-methylpropyl)amino)-3-((S)-2-((*tert*-butoxycarbonyl)amino)-3-methylbutanamido)-4-oxobutanoate

^{31}P NMR (162 MHz, Chloroform-d) δ 18.03 (s, 50%), 17.99 (s, 50%).

^{1}H NMR (400 MHz, Chloroform-d) δ 7.36 – 7.25 (m, 6H, 5xArH, NH), 7.24 – 7.10 (m, 7H, 6xArH, NH), 7.07 – 7.00 (m, 2H, 2xArH), 5.15 – 5.02 (m, 2H, CH$_2$), 4.99 – 4.92 (m, 1H, NH), 4.89 – 4.79 (m, 1H, CH), 4.72 – 4.59 (m, 1H, CHP), 3.99 – 3.88 (m, 1H, CH), 3.10 – 2.98 (m, 1H, 0.5xCH$_2$), 2.78 – 2.59 (m, 1H, 0.5xCH$_2$), 2.44 – 2.40 (m, 6H, 2xSCH$_3$), 2.40 – 2.31 (m, 1H, CH), 2.22 – 2.04 (m, 1H, CH), 1.41 (d, J = 12.9 Hz, 9H, Boc), 1.10 – 0.97 (m, 6H, 2xCH$_3$), 0.96 – 0.81 (m, 6H, 2xCH$_3$).

HRMS: $C_{39}H_{52}N_3O_9PS_2$ [M+Na]$^+$ calc. 824.2780 found 824.2771

HPLC: purity >98%; retention time 19.943 min

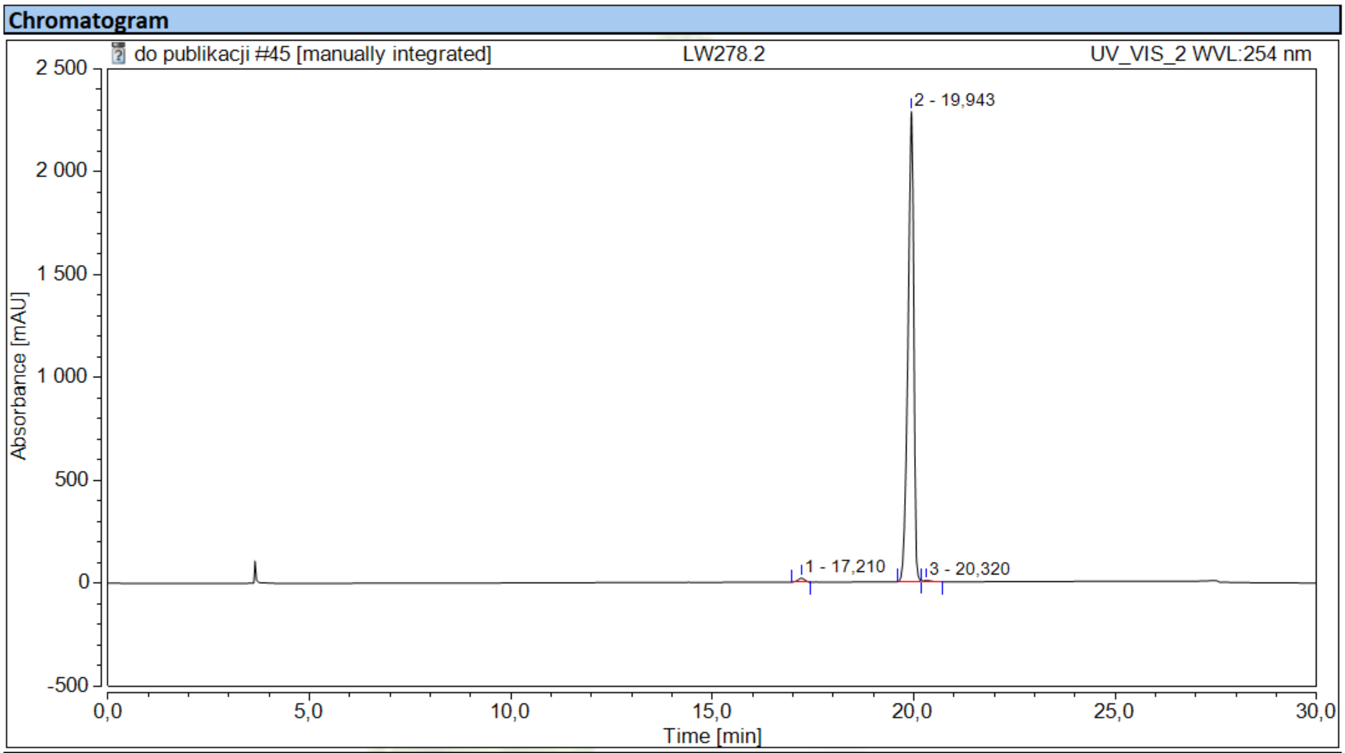

Integration Results

No.	Peak Name	Retention Time min	Area mAU*min	Height mAU	Relative Area %	Relative Height %	Amount n.a.
1		17,210	3,677	19,560	0,96	0,85	n.a.
2		19,943	379,325	2284,511	98,66	98,88	n.a.
3		20,320	1,470	6,313	0,38	0,27	n.a.
**Total:**			**384,472**	**2310,385**	**100,00**	**100,00**	

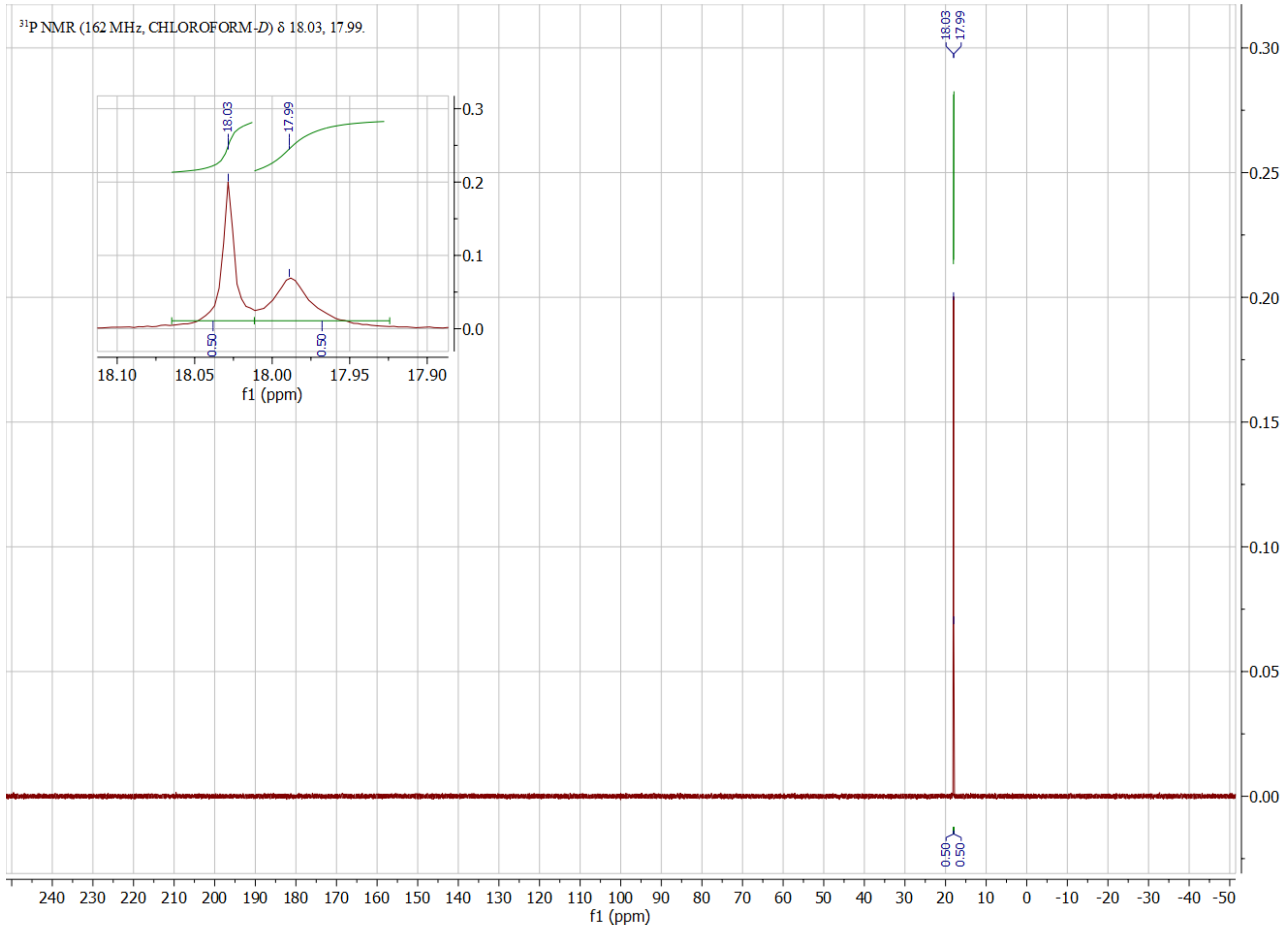
31P NMR (162 MHz, CHLOROFORM-D) δ 18.03, 17.99.
f1 (ppm)

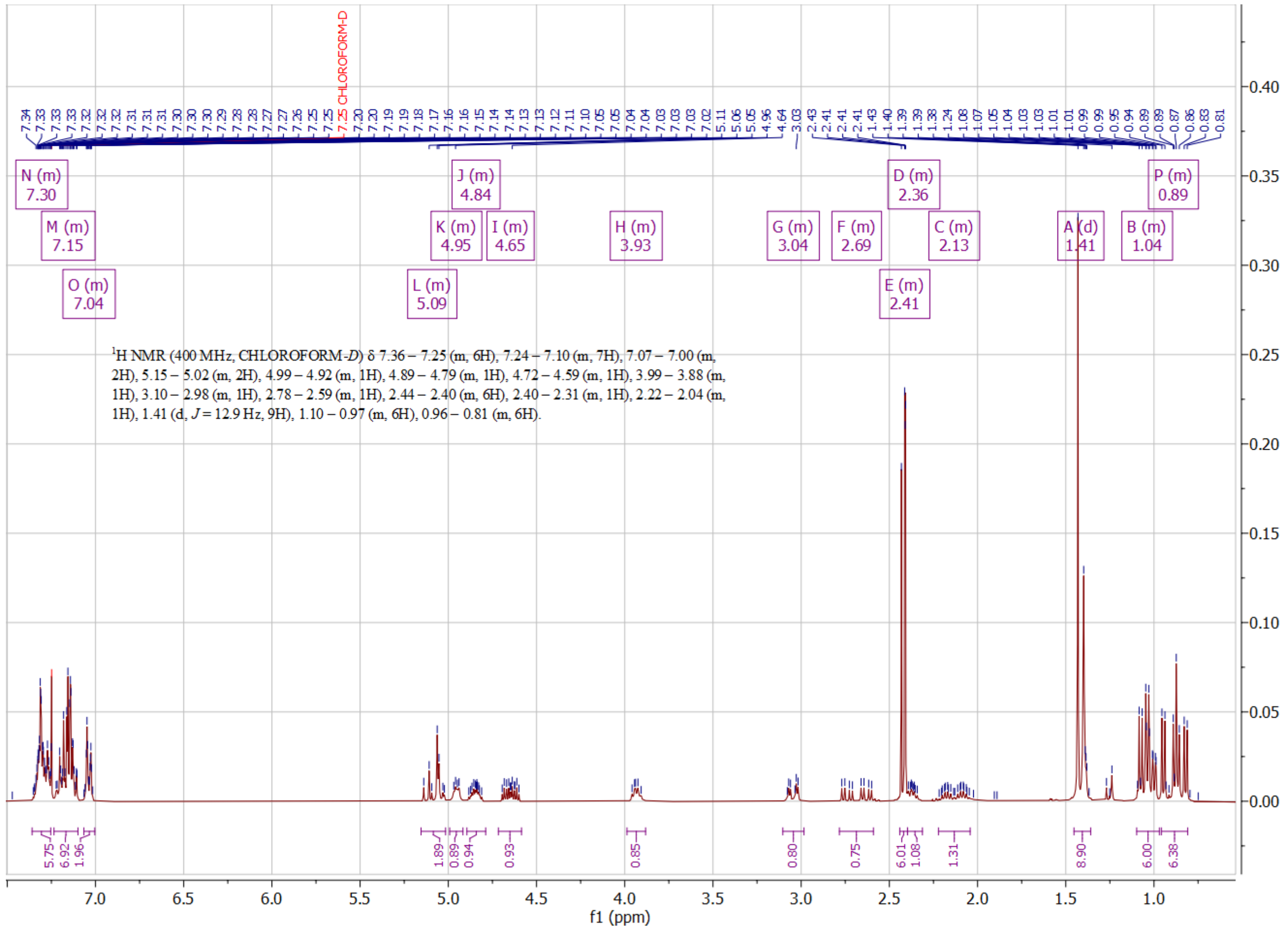
1H NMR (400 MHz, CHLOROFORM-D) δ 7.36 – 7.25 (m, 6H), 7.24 – 7.10 (m, 7H), 7.07 – 7.00 (m, 2H), 5.15 – 5.02 (m, 2H), 4.99 – 4.92 (m, 1H), 4.89 – 4.79 (m, 1H), 4.72 – 4.59 (m, 1H), 3.99 – 3.88 (m, 1H), 3.10 – 2.98 (m, 1H), 2.78 – 2.59 (m, 1H), 2.44 – 2.40 (m, 6H), 2.40 – 2.31 (m, 1H), 2.22 – 2.04 (m, 1H), 1.41 (d, J = 12.9 Hz, 9H), 1.10 – 0.97 (m, 6H), 0.96 – 0.81 (m, 6H).
7.25 CHLOROFORM-D
f1 (ppm)

(46) Boc-L-Val-D-Asp(Bzl)-Val^P(OPh-4-SCH$_3$)$_2$

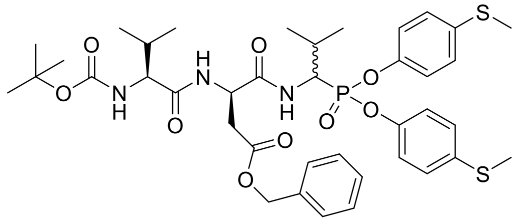

Name: benzyl (3R)-4-((1-(bis(4-(methylthio)phenoxy)phosphoryl)-2-methylpropyl)amino)-3-((S)-2-((*tert*-butoxycarbonyl)amino)-3-methylbutanamido)-4-oxobutanoate

^{31}P NMR (162 MHz, Chloroform-d) δ 18.14 (s, 54%), 17.96 (s, 46%).

^{1}H NMR (400 MHz, Chloroform-d) δ 7.36 – 7.25 (m, 6H, 5xArH, NH), 7.24 – 7.10 (m, 7H, 6xArH, NH), 7.09 – 6.99 (m, 2H, 2xArH), 5.12 – 4.80 (m, 4H, CH2, NH, CH), 4.70 – 4.58 (m, 1H, CHP), 3.92 – 3.77 (m, 1H, CH), 3.11 – 2.94 (m, 1H, 0.5xCH$_2$), 2.75 – 2.56 (m, 1H, 0.5xCH$_2$), 2.46 – 2.30 (m, 7H, 2xSCH$_3$, CH), 2.15 – 2.00 (m, 1H, CH), 1.38 (d, J = 1.8 Hz, 9H, Boc), 1.12 – 0.95 (m, 6H, 2xCH$_3$), 0.93 – 0.81 (m, 6H, 2xCH$_3$).

HRMS: $C_{39}H_{52}N_3O_9PS_2$ [M+Na]$^+$ calc. 824.2780 found 824.2800

HPLC: purity >98%; retention time 19.940 and 20.073 min

Chromatogram

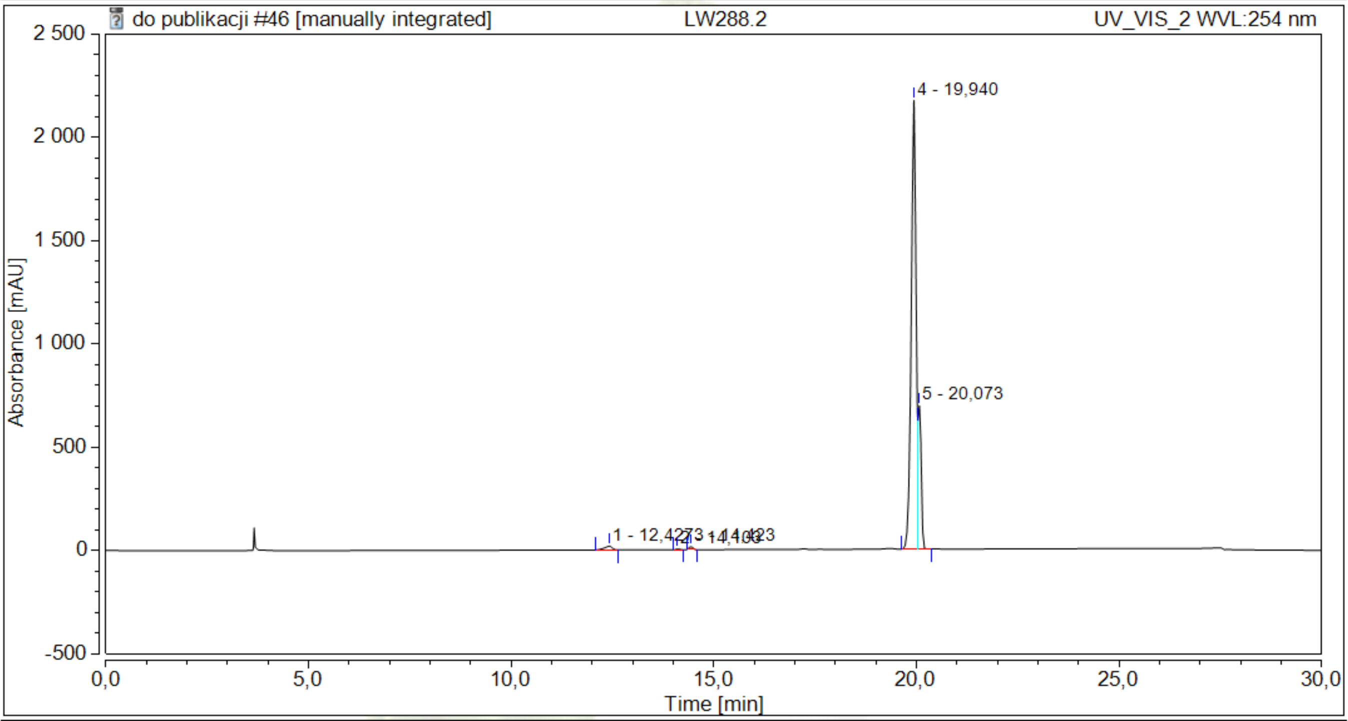

Integration Results

No.	Peak Name	Retention Time min	Area mAU*min	Height mAU	Relative Area %	Relative Height %	Amount n.a.
1		12,427	4,162	19,201	1,14	0,66	n.a.
2		14,103	0,379	3,490	0,10	0,12	n.a.
3		14,423	1,608	14,055	0,44	0,48	n.a.
4		19,940	298,778	2170,841	81,85	74,83	n.a.
5		20,073	60,102	693,562	16,47	23,91	n.a.
**Total:**			**365,030**	**2901,149**	**100,00**	**100,00**	

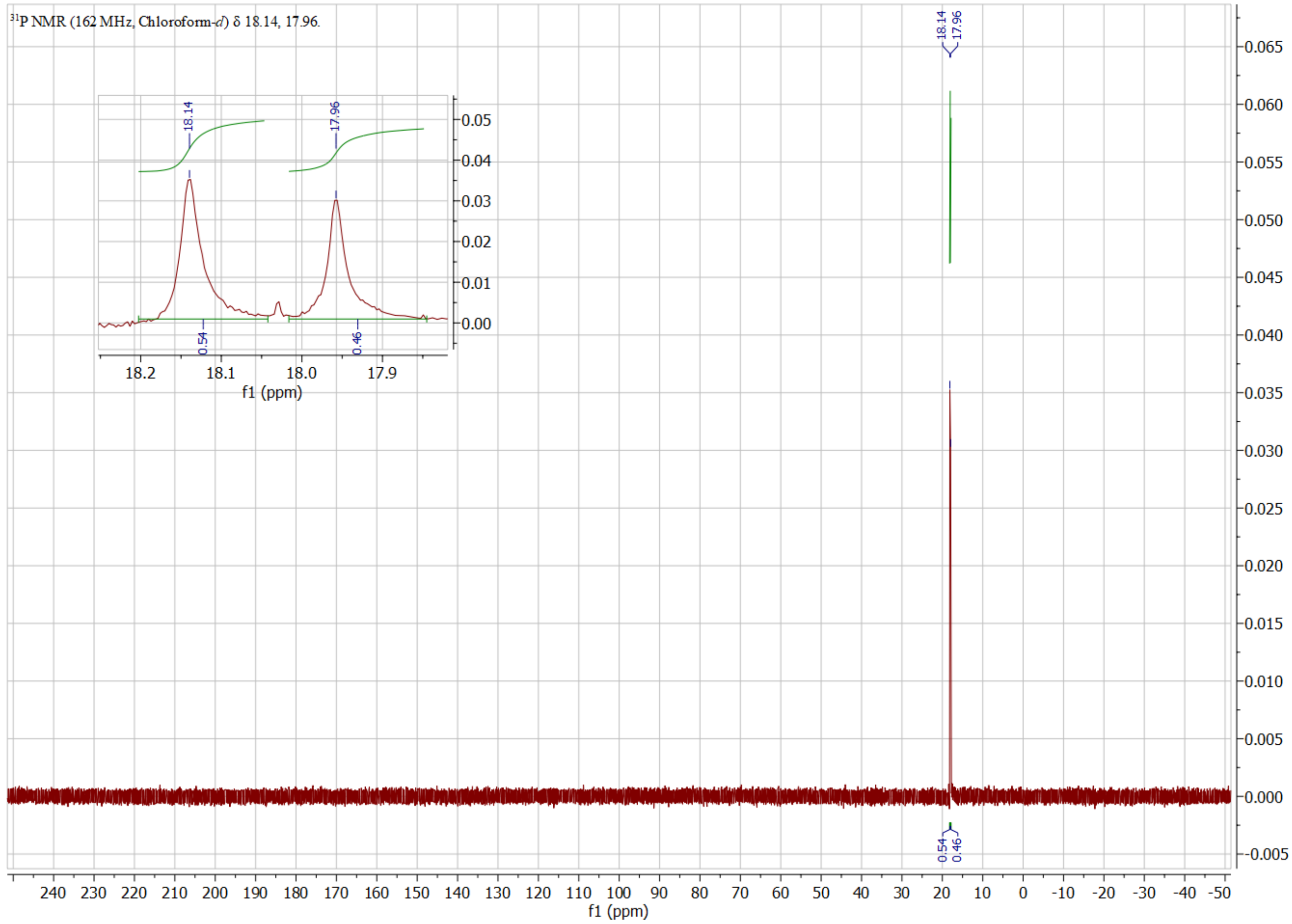

^{31}P NMR (162 MHz, Chloroform-d) δ 18.14, 17.96.

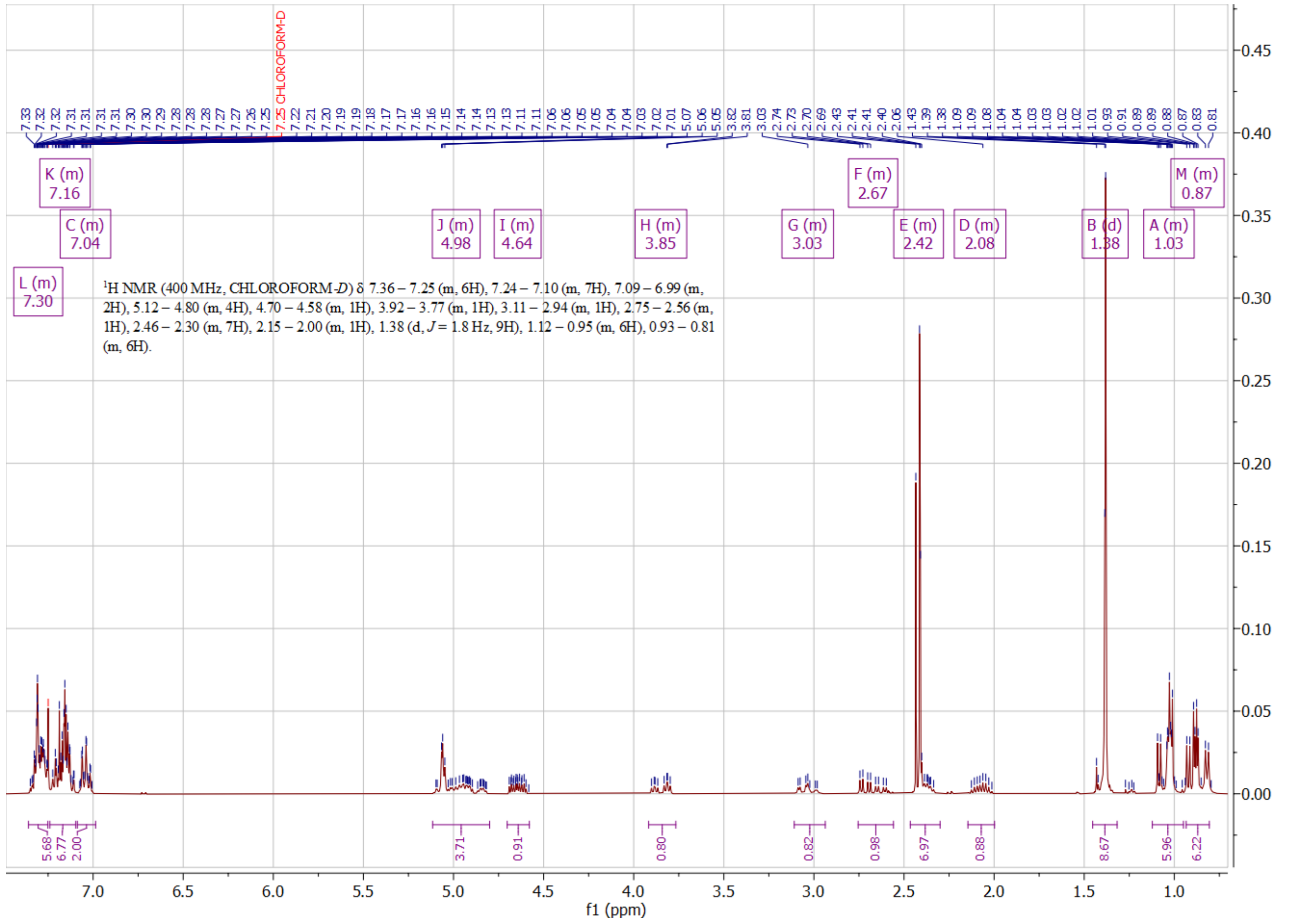

^{1}H NMR (400 MHz, CHLOROFORM-D) δ 7.36 – 7.25 (m, 6H), 7.24 – 7.10 (m, 7H), 7.09 – 6.99 (m, 2H), 5.12 – 4.80 (m, 4H), 4.70 – 4.58 (m, 1H), 3.92 – 3.77 (m, 1H), 3.11 – 2.94 (m, 1H), 2.75 – 2.56 (m, 1H), 2.46 – 2.30 (m, 7H), 2.15 – 2.00 (m, 1H), 1.38 (d, J = 1.8 Hz, 9H), 1.12 – 0.95 (m, 6H), 0.93 – 0.81 (m, 6H).

(47) Boc-L-Val-L-Cys(Bzl)-Val^{P}(OPh-4-SCH_3)$_2$

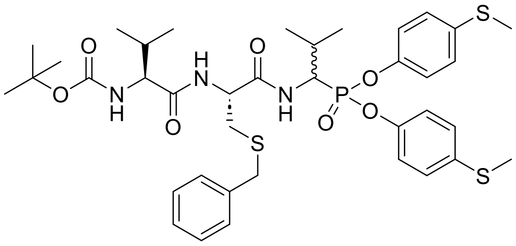

Name: *tert*-butyl ((2S)-1-(((2R)-3-(benzylthio)-1-((1-(bis(4-(methylthio)phenoxy)phosphoryl)-2-methylpropyl)amino)-1-oxopropan-2-yl)amino)-3-methyl-1-oxobutan-2-yl)carbamate

^{31}P NMR (162 MHz, Chloroform-d) δ 17.89 (s).

^{1}H NMR (400 MHz, Chloroform-d) δ 7.35 – 7.25 (m, 4H, 4xArH), 7.24 – 7.01 (m, 9H, 9xArH), 6.99 – 6.87 (m, 1H, NH), 6.82 (dd, J = 28.6, 6.9 Hz, 1H, NH), 5.07 – 4.82 (m, 1H, NH), 4.76 – 4.61 (m, 1H, CHP), 4.54 – 4.43 (m, 1H, CH), 3.96 – 3.88 (m, 1H, CH), 3.77 (d, J = 5.9 Hz, 2H, CH_2), 2.97 – 2.84 (m, 1H, 0.5xCH_2), 2.73 – 2.58 (m, 1H, 0.5xCH_2), 2.43 (dd, J = 5.3, 2.1 Hz, 6H, 2xSCH_3), 2.41 – 2.32 (m, 1H, CH), 2.23 – 2.09 (m, 1H, CH), 1.42 (d, J = 11.5 Hz, 9H, Boc), 1.14 – 0.99 (m, 6H, 2xCH_3), 0.94 (dd, J = 7.9, 6.8 Hz, 3H, CH_3), 0.86 (dd, J = 6.9, 3.5 Hz, 3H, CH_3).

HRMS: $C_{38}H_{52}N_3O_7PS_3$ $[M+Na]^+$ calc. 812.2603 found 812.2574

HPLC: purity >98%; retention time 19.977 and 20.197 min

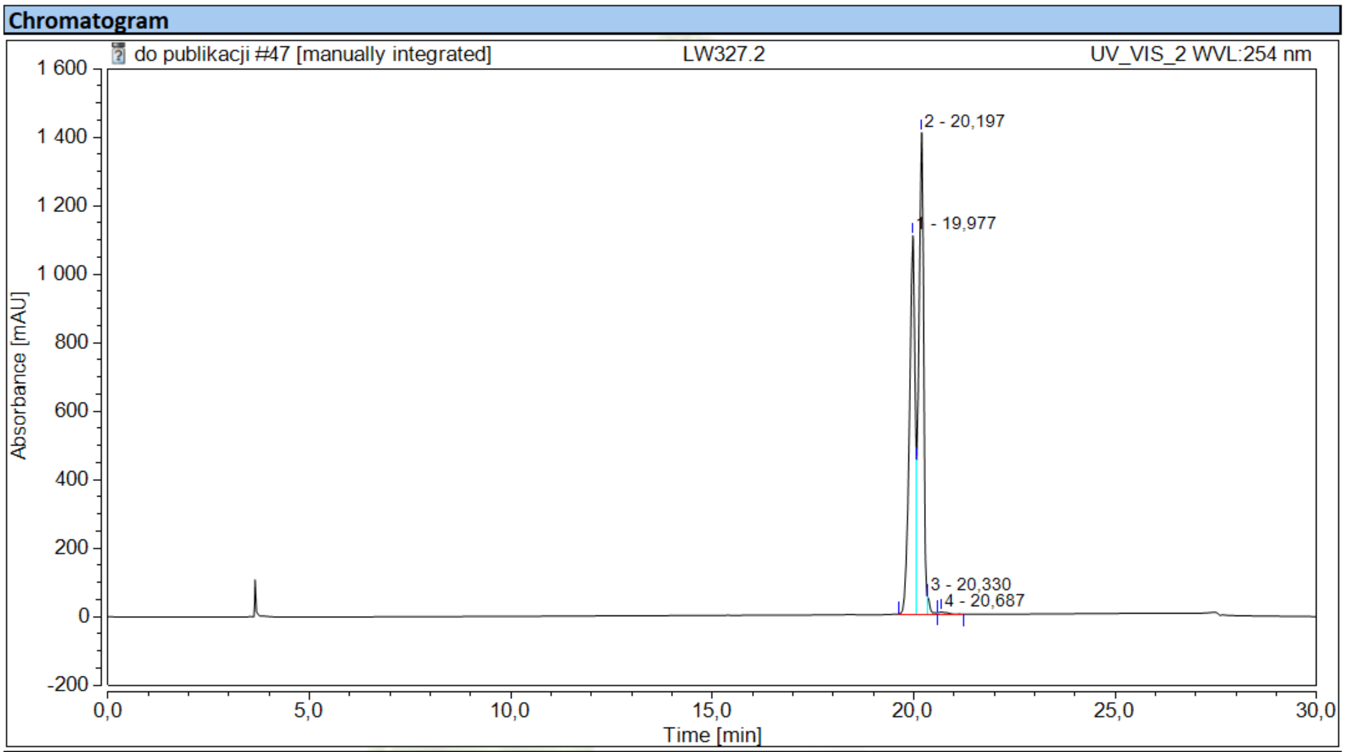

Integration Results

No.	Peak Name	Retention Time min	Area mAU*min	Height mAU	Relative Area %	Relative Height %	Amount n.a.
1		19,977	178,894	1105,174	46,95	42,99	n.a.
2		20,197	196,546	1406,187	51,58	54,70	n.a.
3		20,330	4,038	52,855	1,06	2,06	n.a.
4		20,687	1,570	6,283	0,41	0,24	n.a.
**Total:**			**381,048**	**2570,498**	**100,00**	**100,00**	

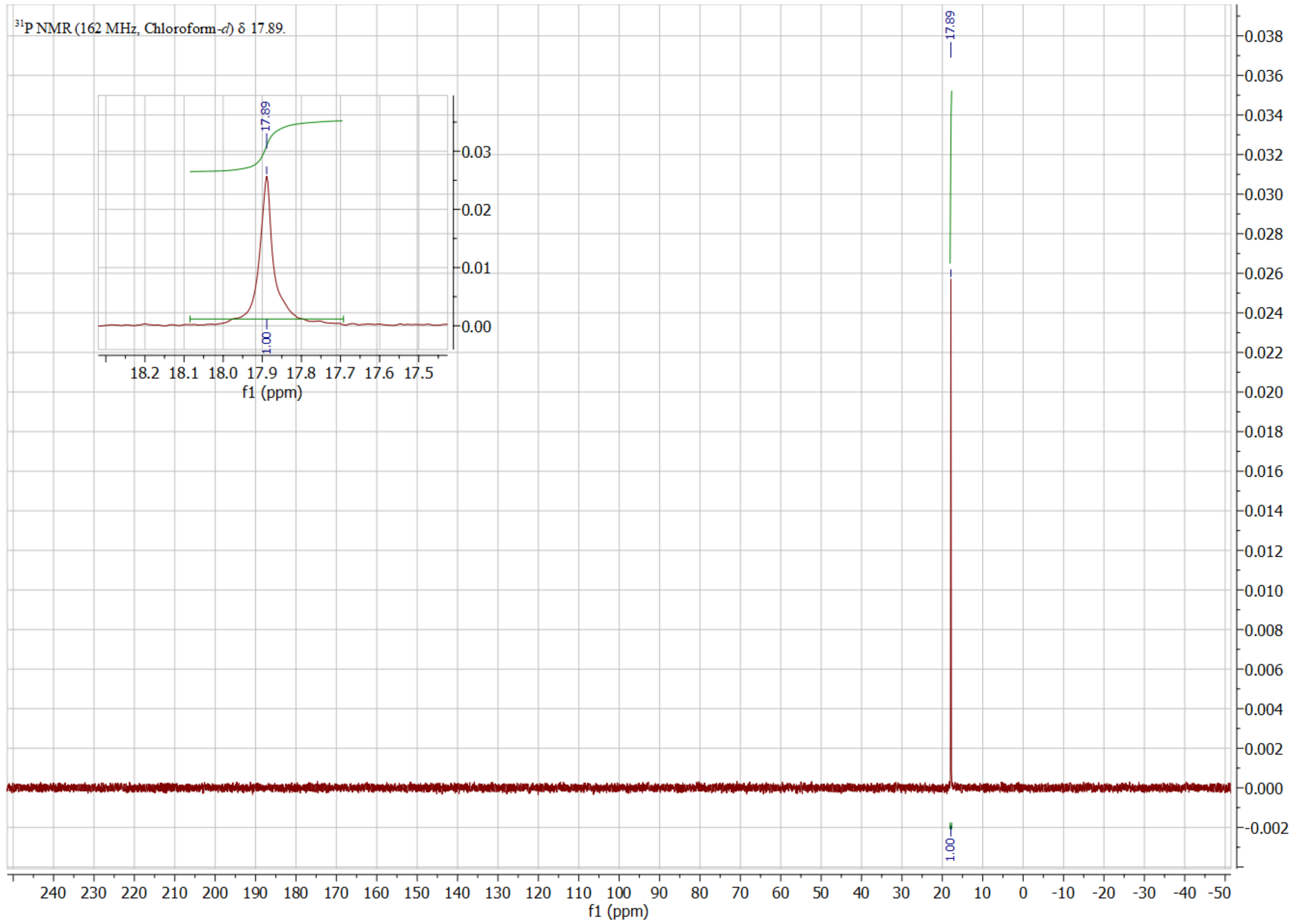
^{31}P NMR (162 MHz, Chloroform-d) δ 17.89.
f1 (ppm)

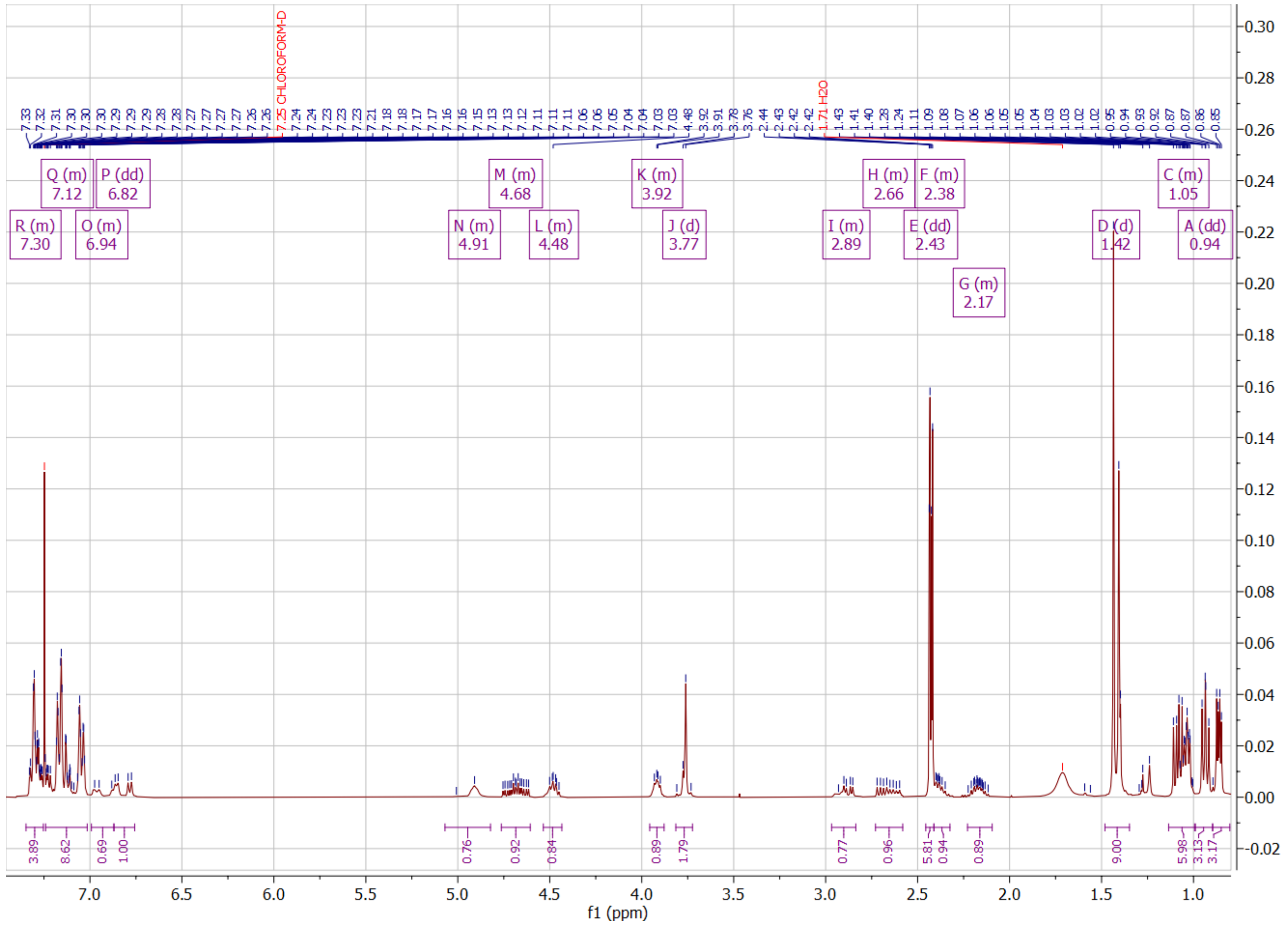
CHLOROFORM-D
H2O
Q (m) 7.12
P (dd) 6.82
R (m) 7.30
O (m) 6.94
M (m) 4.68
N (m) 4.91
L (m) 4.48
K (m) 3.92
J (d) 3.77
I (m) 2.89
H (m) 2.66
F (m) 2.38
E (dd) 2.43
G (m) 2.17
D (d) 1.42
C (m) 1.05
A (dd) 0.94
f1 (ppm)

(48) Boc-L-Val-L-Glu(Bzl)- Val^{P}(OPh-4-SCH$_3$)$_2$

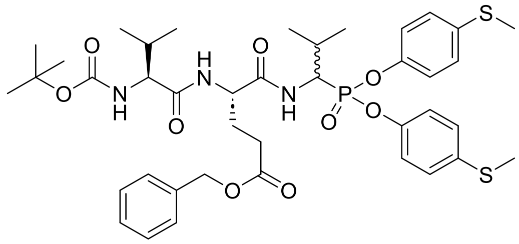

Name: benzyl (4S)-5-((1-(bis(4-(methylthio)phenoxy)phosphoryl)-2-methylpropyl)amino)-4-((S)-2-((*tert*-butoxycarbonyl)amino)-3-methylbutanamido)-5-oxopentanoate

^{31}**P NMR** (162 MHz, Chloroform-d) δ 18.23 (s, 43%), 18.17 (s, 57%).

^{1}**H NMR** (400 MHz, Chloroform-d) δ 7.37 – 7.26 (m, 5H, 5xArH), 7.24 – 6.93 (m, 10H, 8xArH, 2xNH), 5.16 – 5.05 (m, 2H, CH$_2$), 4.99 (dd, J = 26.5, 8.0 Hz, 1H, NH), 4.75 – 4.62 (m, 1H, CHP), 4.57 – 4.47 (m, 1H, CH), 3.93 – 3.85 (m, 1H, CH), 2.68 – 2.53 (m, 1H, 0.5xCH$_2$), 2.48 – 2.32 (m, 8H, 2xSCH$_3$, 0.5xCH$_2$, CH), 2.25 – 1.89 (m, 3H, CH$_2$, CH), 1.41 (d, J = 9.3 Hz, 9H, Boc), 1.11 – 1.00 (m, 6H, 2xCH$_3$), 0.94 – 0.78 (m, 6H, 2xCH$_3$).

HRMS: $C_{40}H_{54}N_3O_9PS_2$ [M+Na]$^+$ calc. 838.2937 found 838.2911

HPLC: purity >99%; retention time 19.583 and 19.860 min

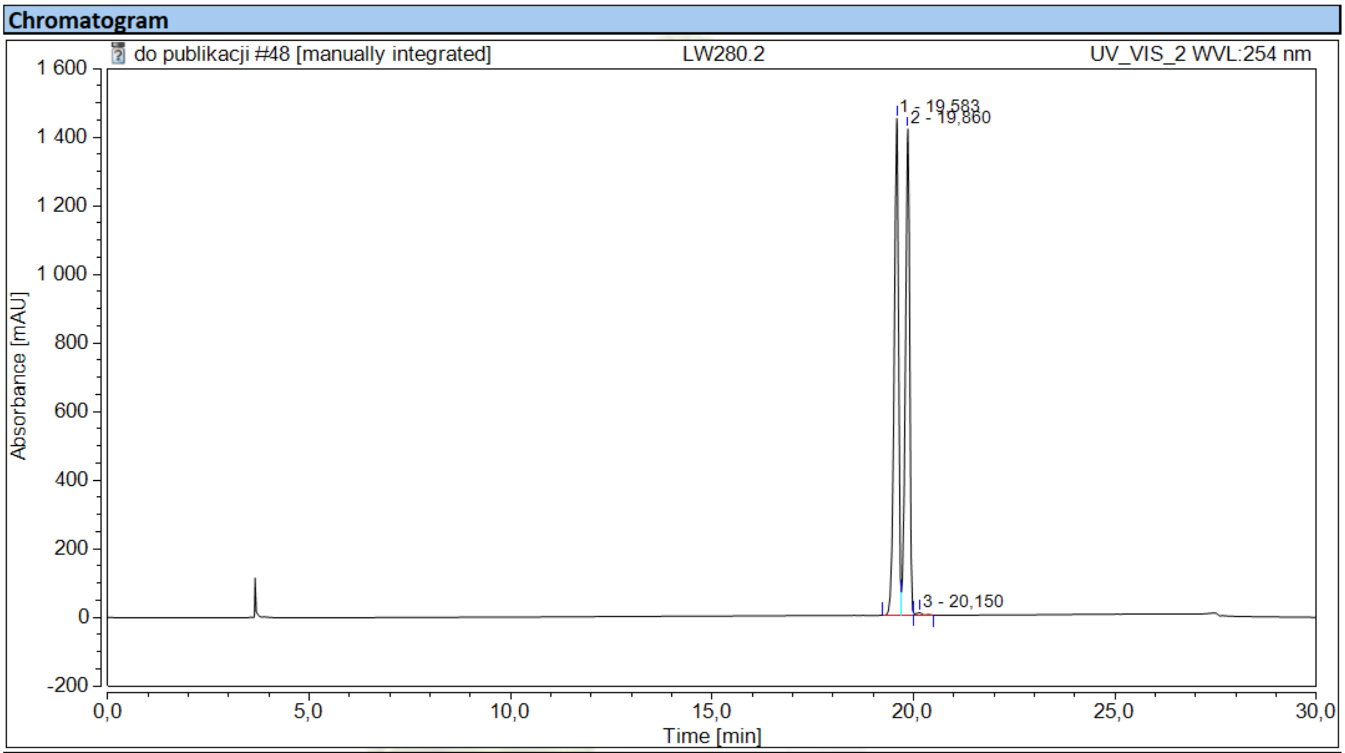

Integration Results

No.	Peak Name	Retention Time min	Area mAU*min	Height mAU	Relative Area %	Relative Height %	Amount n.a.
1		19,583	182,891	1448,310	51,26	50,41	n.a.
2		19,860	172,513	1417,250	48,35	49,33	n.a.
3		20,150	1,401	7,558	0,39	0,26	n.a.
**Total:**			**356,805**	**2873,118**	**100,00**	**100,00**	

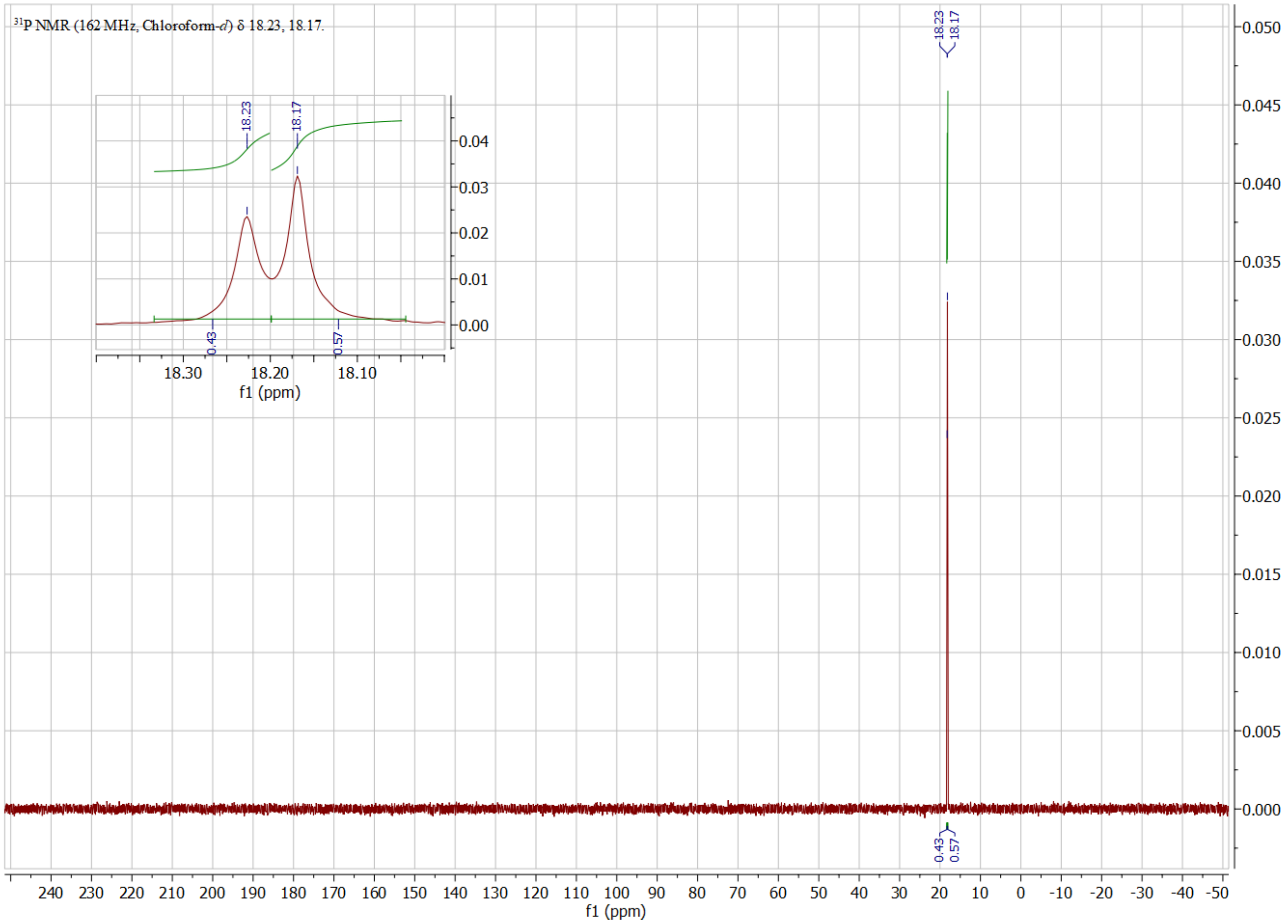

31P NMR (162 MHz, Chloroform-d) δ 18.23, 18.17.
f1 (ppm)

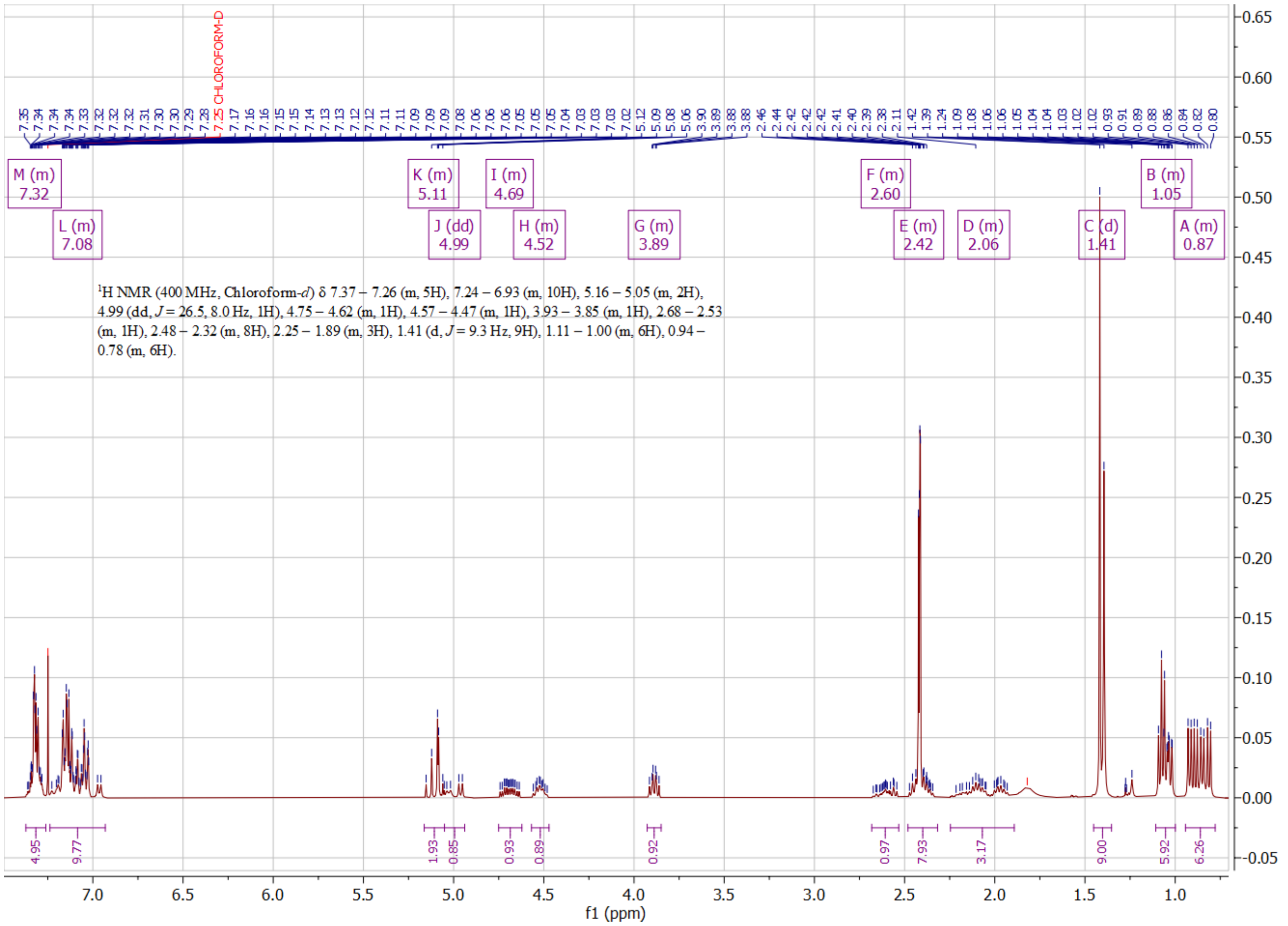

CHLOROFORM-D
1H NMR (400 MHz, Chloroform-d) δ 7.37 – 7.26 (m, 5H), 7.24 – 6.93 (m, 10H), 5.16 – 5.05 (m, 2H), 4.99 (dd, J = 26.5, 8.0 Hz, 1H), 4.75 – 4.62 (m, 1H), 4.57 – 4.47 (m, 1H), 3.93 – 3.85 (m, 1H), 2.68 – 2.53 (m, 1H), 2.48 – 2.32 (m, 8H), 2.25 – 1.89 (m, 3H), 1.41 (d, J = 9.3 Hz, 9H), 1.11 – 1.00 (m, 6H), 0.94 – 0.78 (m, 6H).
f1 (ppm)

(49) Boc-L-Val-D-Orn(Z)-Val^{P}(OPh-4-SCH$_3$)$_2$

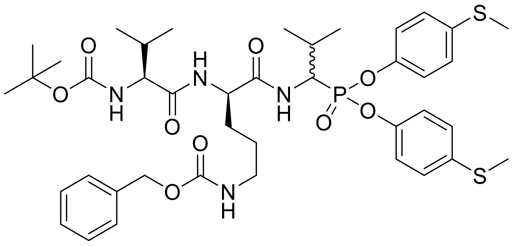

Name: *tert*-butyl ((2S)-1-(((2R)-5-(((benzyloxy)carbonyl)amino)-1-((1-(bis(4-(methylthio)phenoxy)phosphoryl)-2-methylpropyl)amino)-1-oxopentan-2-yl)amino)-3-methyl-1-oxobutan-2-yl)carbamate

^{31}P NMR (162 MHz, Chloroform-d) δ 18.23 (s, 50%), 18.11 (s, 50%).

^{1}H NMR (400 MHz, Chloroform-d) δ 7.51 (dd, J = 32.6, 10.3 Hz, 1H, NH), 7.34 – 7.22 (m, 5H, 5xArH), 7.19 – 7.01 (m, 8H, 8xArH), 7.00 – 6.90 (m, 1H, NH), 5.32 – 5.14 (m, 2H, 2xNH), 5.15 – 4.91 (m, 2H, CH_2), 4.81 – 4.55 (m, 2H, CHP, CH), 4.02 – 3.88 (m, 1H, CH), 3.47 – 3.30 (m, 1H, 0.5xCH_2), 3.17 – 2.94 (m, 1H, 0.5xCH_2), 2.45 – 2.38 (m, 6H, 2xSCH_3), 2.38 – 2.29 (m, 1H, CH), 2.16 – 2.03 (m, 1H, CH), 1.95 – 1.65 (m, 1H, 0.5x CH_2), 1.65 – 1.42 (m, 3H, 1.5 CH_2), 1.39 (d, J = 7.4 Hz, 9H, Boc), 1.16 – 0.97 (m, 6H, 2xCH_3), 0.93 – 0.80 (m, 6H, 2xCH_3).

HRMS: $C_{41}H_{57}N_4O_9PS_2$ $[M+Na]^+$ calc. 867.3202 found 867.3196

HPLC: purity >99%; retention time 19.050 and 19.183 min

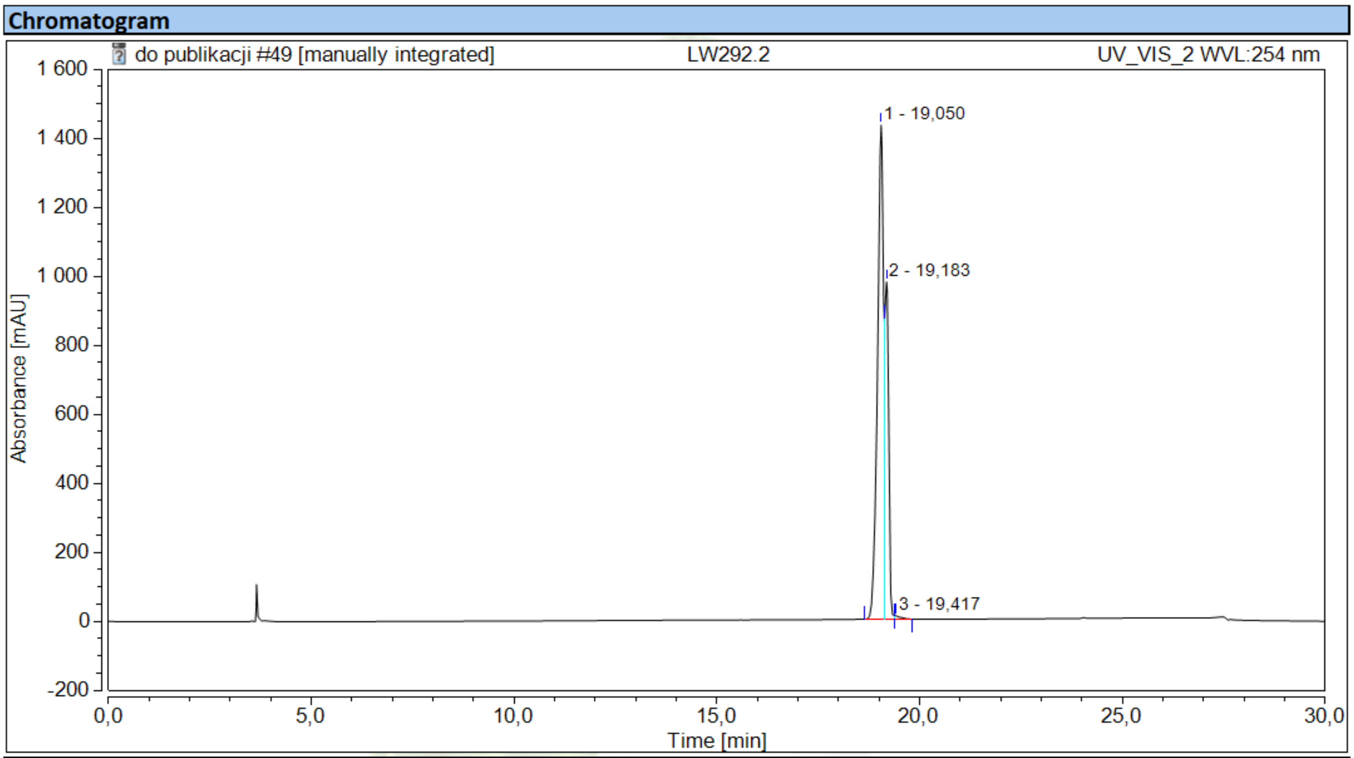

Integration Results

No.	Peak Name	Retention Time min	Area mAU*min	Height mAU	Relative Area %	Relative Height %	Amount n.a.
1		19,050	247,912	1431,932	70,35	59,19	n.a.
2		19,183	102,575	977,368	29,11	40,40	n.a.
3		19,417	1,922	9,901	0,55	0,41	n.a.
**Total:**			**352,409**	**2419,201**	**100,00**	**100,00**	

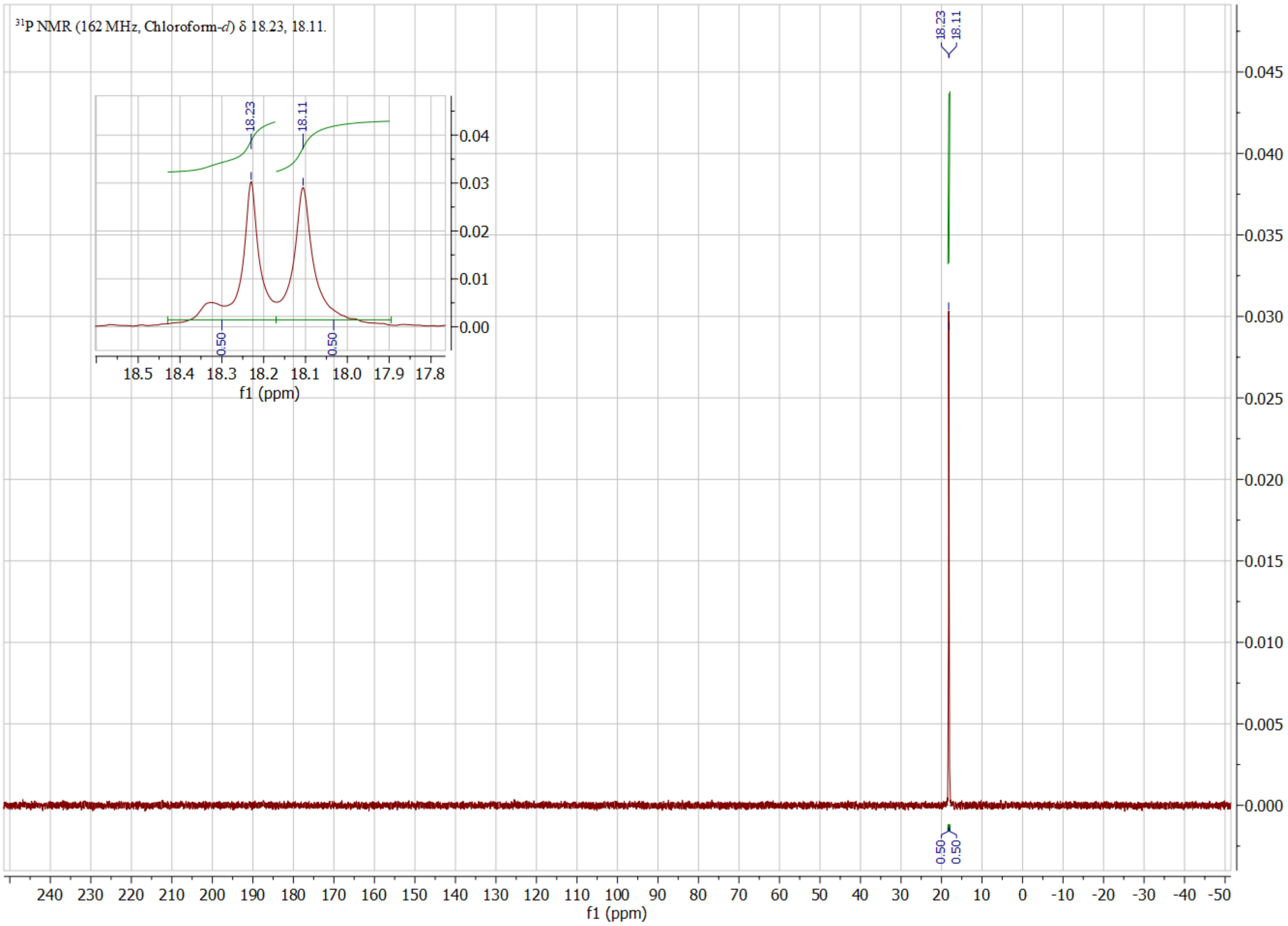
^{31}P NMR (162 MHz, Chloroform-*d*) δ 18.23, 18.11.
18.23
18.11
0.50
0.50
f1 (ppm)

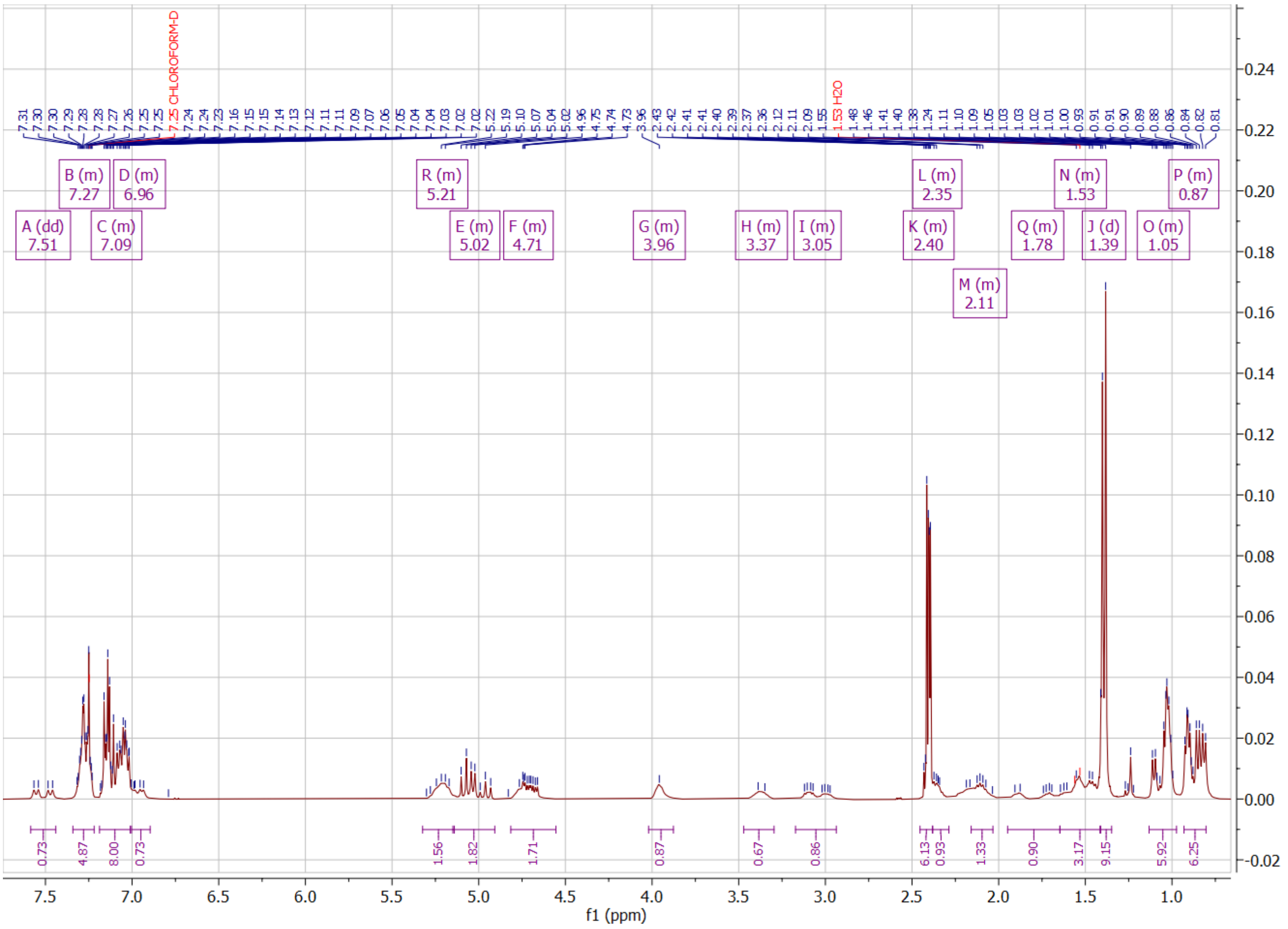
7.25 CHLOROFORM-D
1.53 H2O
A (dd) 7.51
B (m) 7.27
C (m) 7.09
D (m) 6.96
R (m) 5.21
E (m) 5.02
F (m) 4.71
G (m) 3.96
H (m) 3.37
I (m) 3.05
L (m) 2.35
K (m) 2.40
M (m) 2.11
Q (m) 1.78
N (m) 1.53
J (d) 1.39
P (m) 0.87
O (m) 1.05
f1 (ppm)

(50) Boc-L-Val-D-Tyr(Bzl)- Val^P(OPh-4-SCH$_3$)$_2$

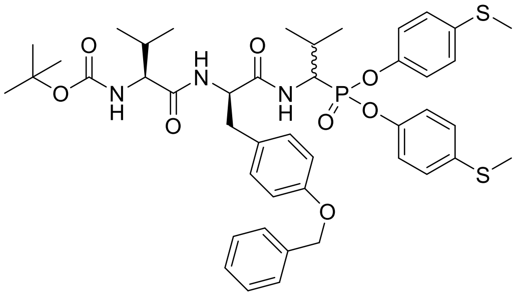

Name: *tert*-butyl ((2S)-1-(((2R)-3-(4-(benzyloxy)phenyl)-1-((1-(bis(4-(methylthio)phenoxy)phosphoryl)-2-methylpropyl)amino)-1-oxopropan-2-yl)amino)-3-methyl-1-oxobutan-2-yl)carbamate
^{31}P NMR (162 MHz, Chloroform-d) δ 18.18 (s, 57%), 17.97 (s, 43%).
^{1}H NMR (400 MHz, Chloroform-d) δ 7.44 – 7.25 (m, 5H, 5xArH), 7.21 – 6.90 (m, 11H, 10xArH, NH), 6.87 – 6.76 (m, 2H, 2xArH), 6.54 (dd, J = 114.5, 8.0 Hz, 1H, NH), 5.05 (dd, J = 41.3, 8.7 Hz, 1H, NH), 4.93 (d, J = 27.4 Hz, 2H, CH$_2$), 4.80 – 4.61 (m, 2H, CHP, CH), 3.88 – 3.72 (m, 1H, CH), 3.12 – 2.84 (m, 2H, CH$_2$), 2.44 – 2.38 (m, 6H, 2xSCH$_3$), 2.37 – 2.20 (m, 1H, CH), 2.00 – 1.83 (m, 1H, CH), 1.38 (d, J = 4.0 Hz, 9H, Boc), 1.04 – 0.64 (m, 12H, 4xCH$_3$).
HRMS: $C_{44}H_{56}N_3O_8PS_2$ [M+Na]$^+$ calc. 872.3144 found 872.3152
HPLC: purity >99%; retention time 20.607 and 20.803 min

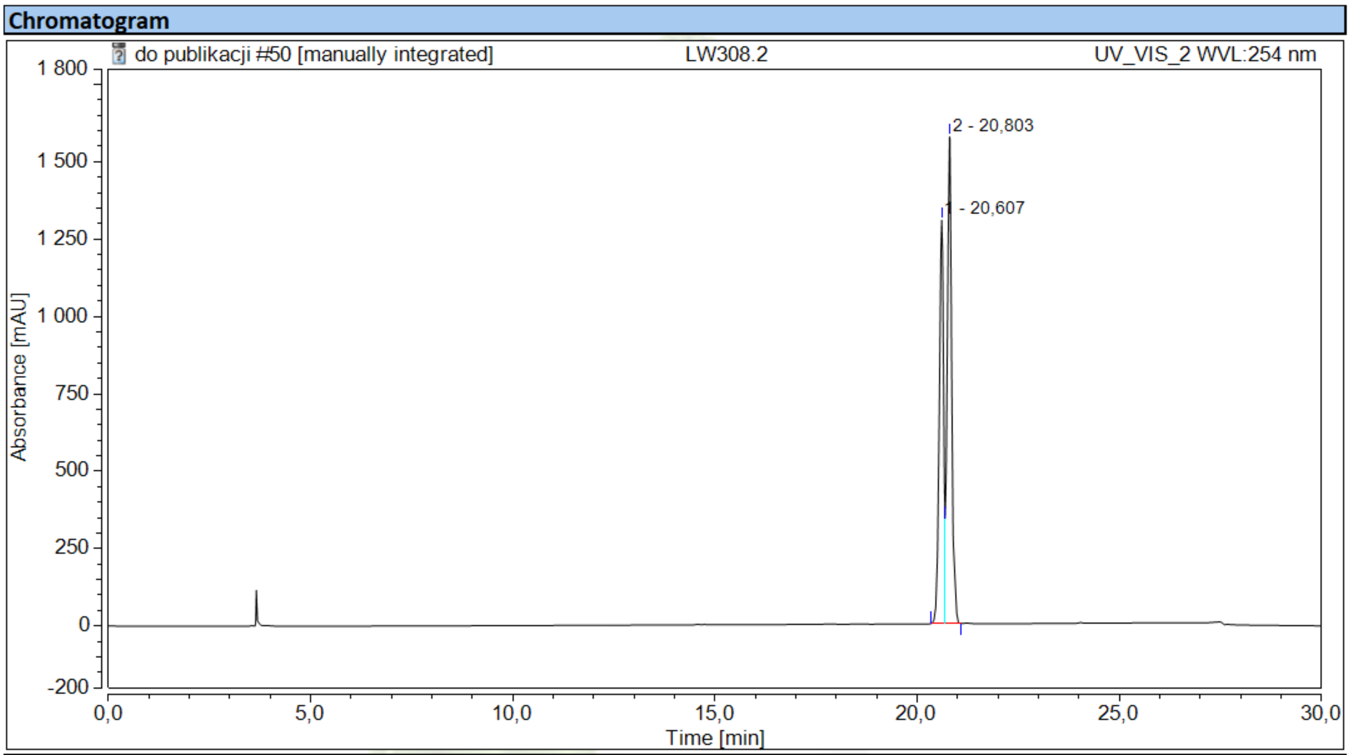

Integration Results

No.	Peak Name	Retention Time min	Area mAU*min	Height mAU	Relative Area %	Relative Height %	Amount n.a.
1		20,607	161,288	1303,204	44,52	45,32	n.a.
2		20,803	200,988	1572,320	55,48	54,68	n.a.
**Total:**			**362,275**	**2875,524**	**100,00**	**100,00**	

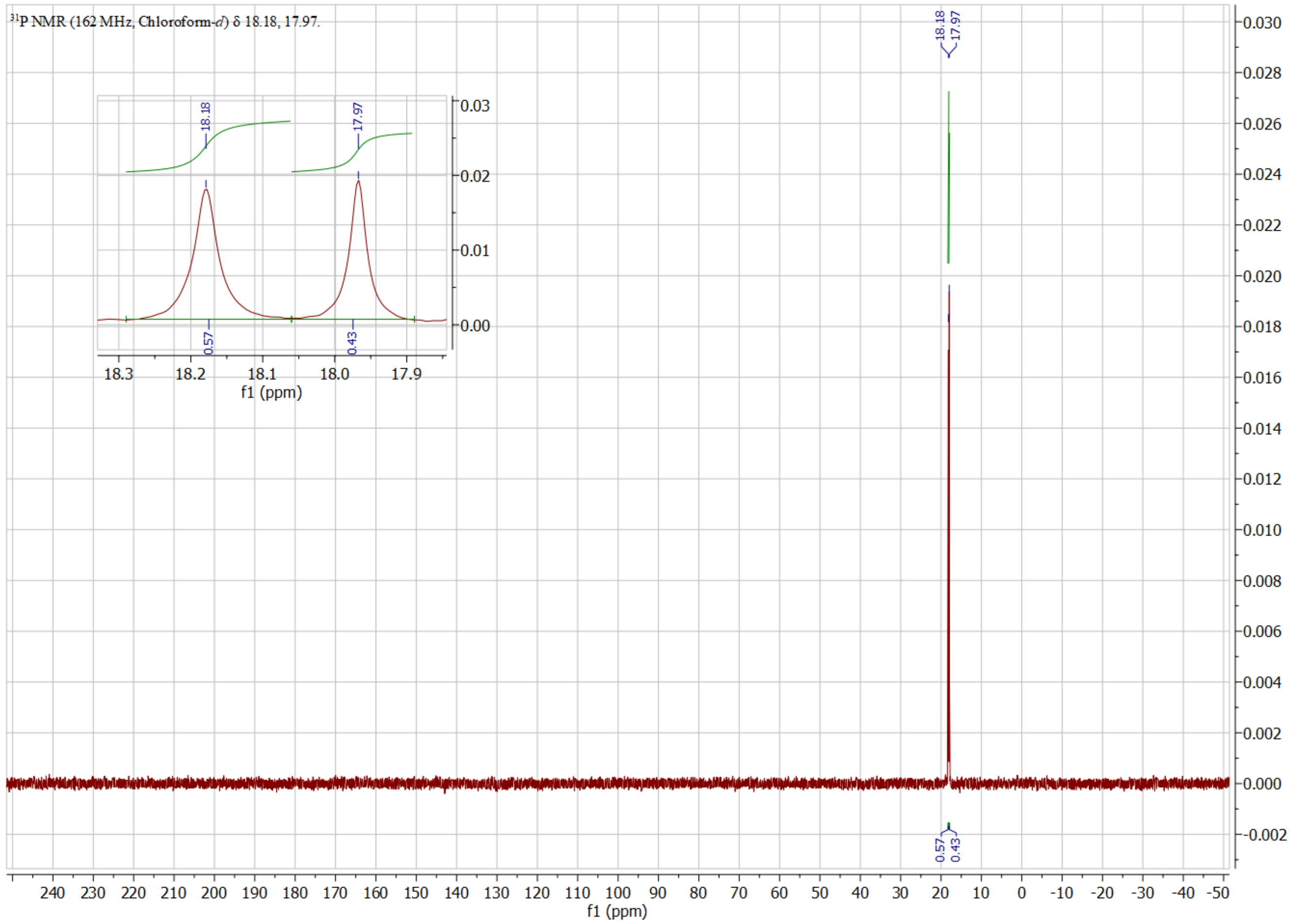
31P NMR (162 MHz, Chloroform-d) δ 18.18, 17.97.
f1 (ppm)

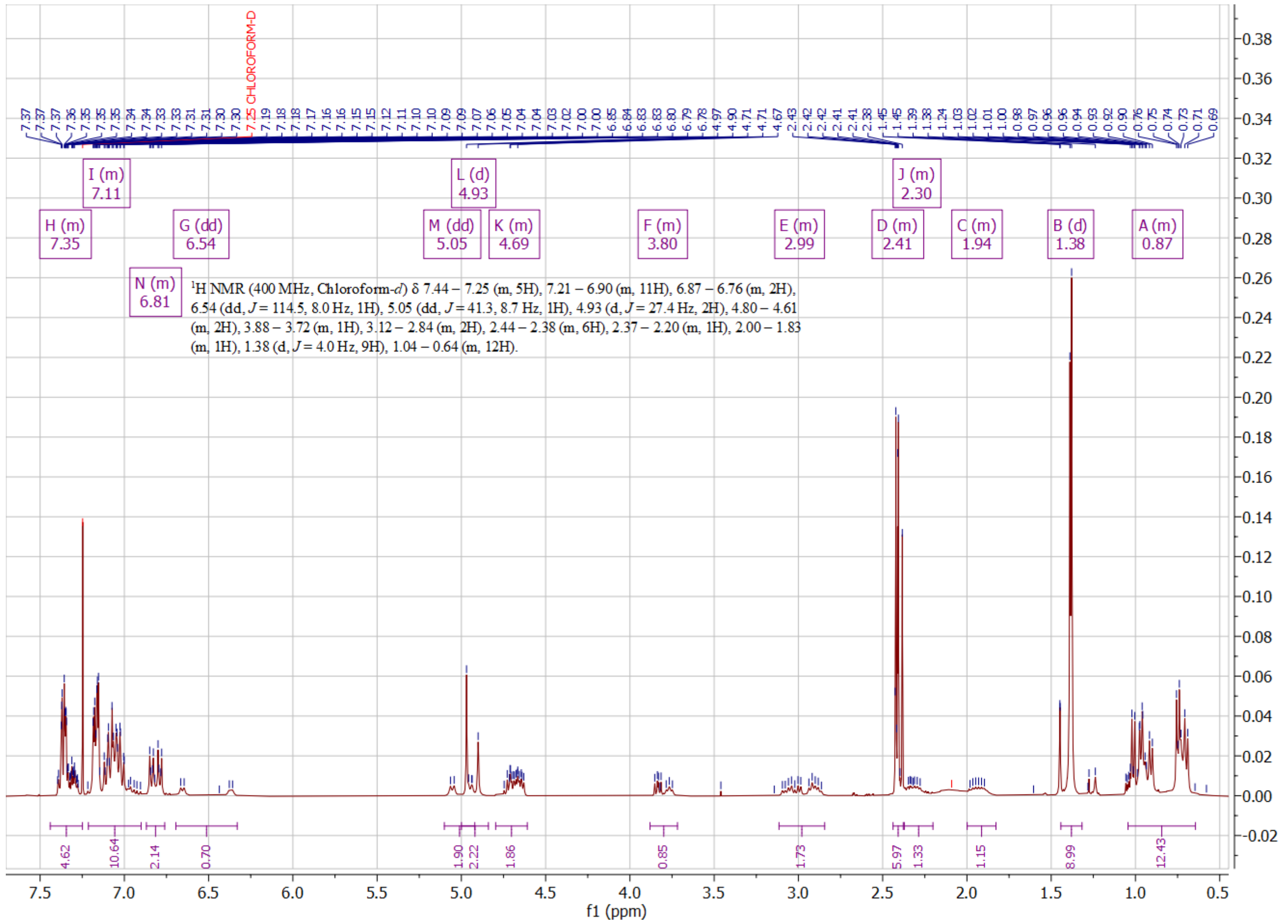
1H NMR (400 MHz, Chloroform-d) δ 7.44 – 7.25 (m, 5H), 7.21 – 6.90 (m, 11H), 6.87 – 6.76 (m, 2H), 6.54 (dd, J = 114.5, 8.0 Hz, 1H), 5.05 (dd, J = 41.3, 8.7 Hz, 1H), 4.93 (d, J = 27.4 Hz, 2H), 4.80 – 4.61 (m, 2H), 3.88 – 3.72 (m, 1H), 3.12 – 2.84 (m, 2H), 2.44 – 2.38 (m, 6H), 2.37 – 2.20 (m, 1H), 2.00 – 1.83 (m, 1H), 1.38 (d, J = 4.0 Hz, 9H), 1.04 – 0.64 (m, 12H).
CHLOROFORM-D
f1 (ppm)

(51) Boc-L-Val-L-Tyr(Bzl)-Val^P(OPh-4-SCH$_3$)$_2$

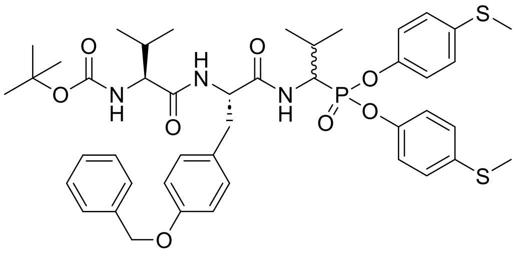

Name: *tert*-butyl ((2S)-1-(((2S)-3-(4-(benzyloxy)phenyl)-1-((1-(bis(4-(methylthio)phenoxy)phosphoryl)-2-methylpropyl)amino)-1-oxopropan-2-yl)amino)-3-methyl-1-oxobutan-2-yl)carbamate

^{31}P NMR (162 MHz, Chloroform-d) δ 18.13 (s, 42%), 18.06 (s, 58%).

^{1}H NMR (400 MHz, Chloroform-d) δ 7.41 – 7.25 (m, 5H, 5xArH), 7.24 – 6.90 (m, 11H, 10xArH, NH), 6.88 – 6.78 (m, 2H, 2xArH), 6.54 (d, J = 8.9 Hz, 1H, NH), 5.08 – 4.81 (m, 3H, CH$_2$, NH), 4.76 – 4.60 (m, 2H, CHP, CH), 3.90 – 3.81 (m, 1H, CH), 3.16 – 2.91 (m, 2H, CH$_2$), 2.44 – 2.37 (m, 6H, 2xSCH$_3$), 2.37 – 2.23 (m, 1H, CH), 2.18 – 2.02 (m, 1H, CH), 1.39 (d, J = 7.3 Hz, 9H, Boc), 1.08 – 0.90 (m, 6H, 2xCH$_3$), 0.90 – 0.71 (m, 6H, 2xCH$_3$).

HRMS: $C_{44}H_{56}N_3O_8PS_2$ $[M+Na]^+$ calc. 872.3144 found 872.3138

HPLC: purity >98%; retention time 20.450 and 20.627 min

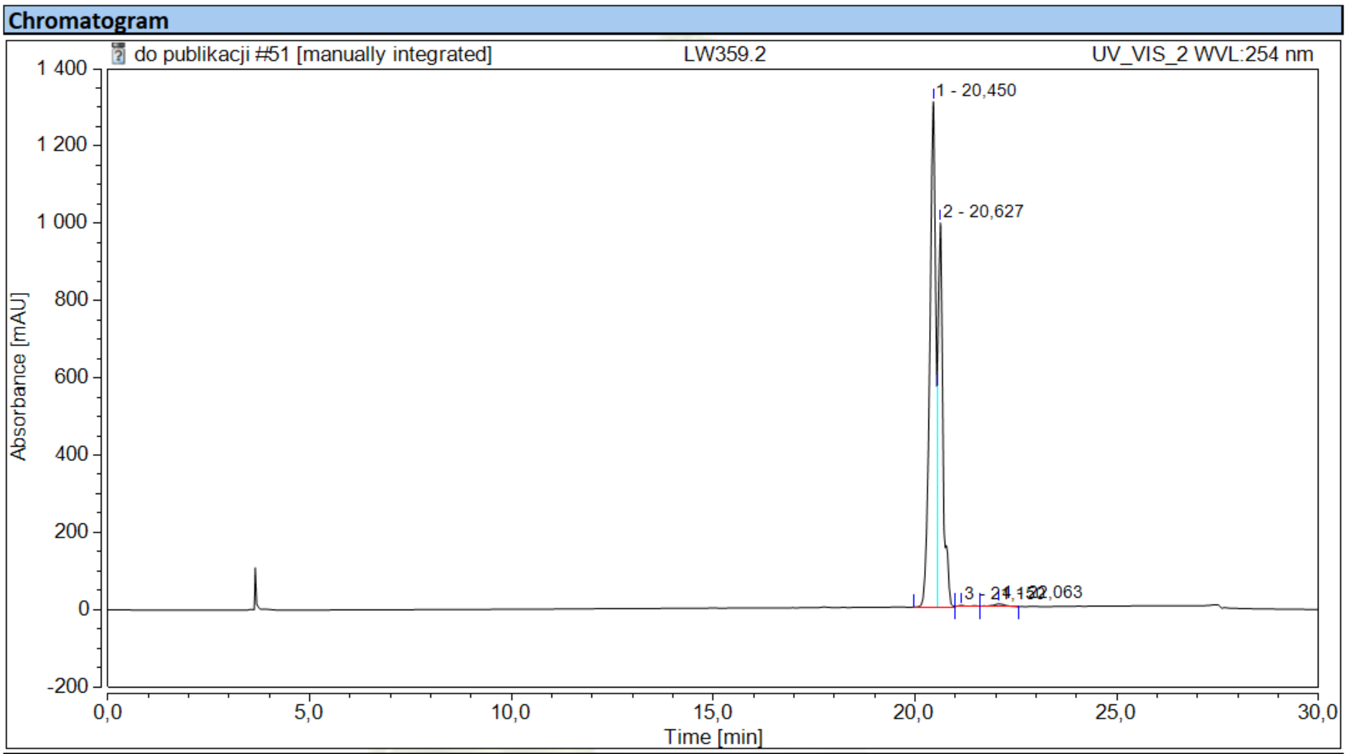

Integration Results

No.	Peak Name	Retention Time min	Area mAU*min	Height mAU	Relative Area %	Relative Height %	Amount n.a.
1		20,450	217,666	1308,190	59,62	56,54	n.a.
2		20,627	143,035	994,418	39,18	42,98	n.a.
3		21,150	1,514	3,923	0,41	0,17	n.a.
4		22,063	2,867	7,411	0,79	0,32	n.a.
**Total:**			**365,082**	**2313,942**	**100,00**	**100,00**	

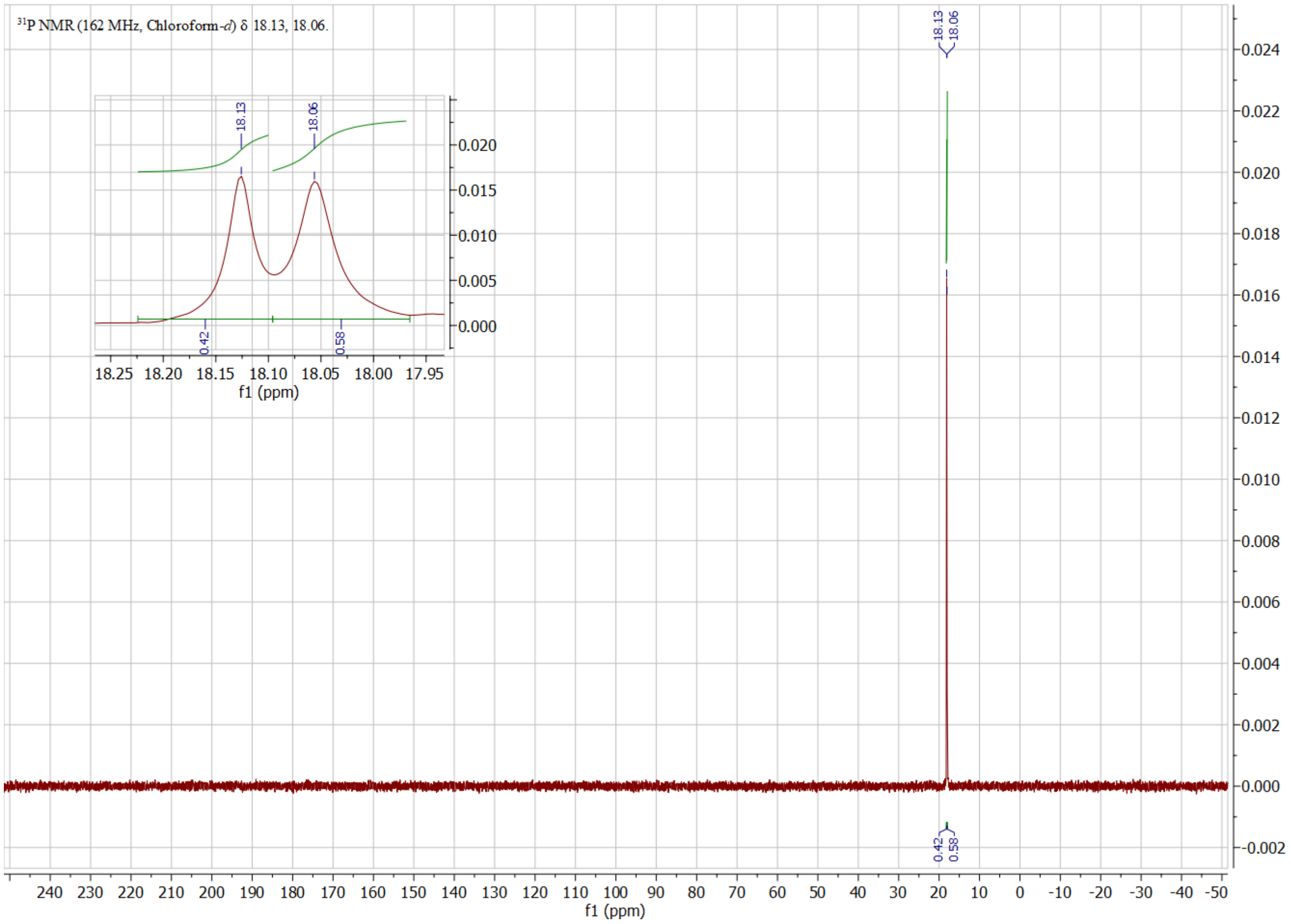

^{31}P NMR (162 MHz, Chloroform-d) δ 18.13, 18.06.
f1 (ppm)

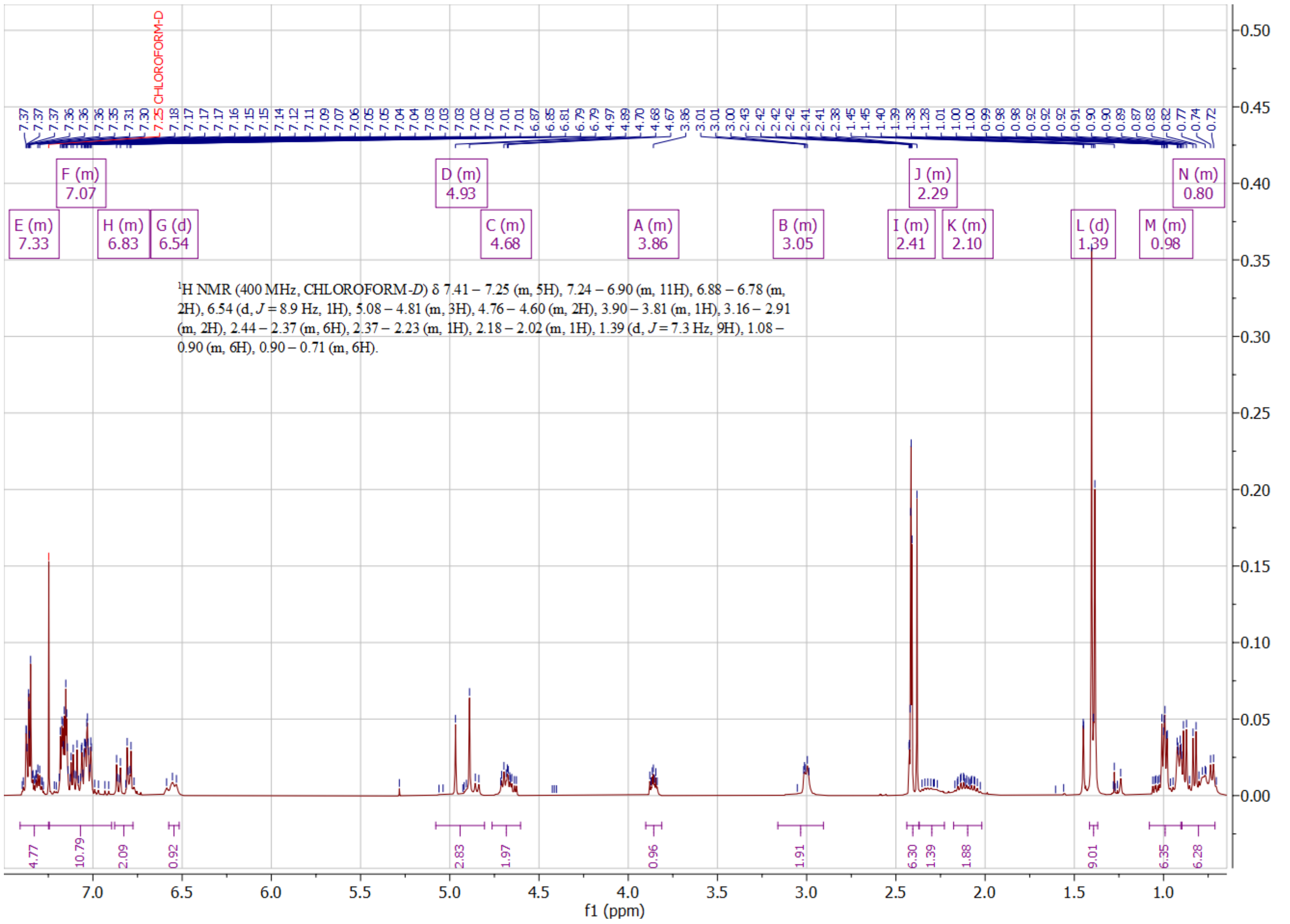

^{1}H NMR (400 MHz, CHLOROFORM-D) δ 7.41 – 7.25 (m, 5H), 7.24 – 6.90 (m, 11H), 6.88 – 6.78 (m, 2H), 6.54 (d, J = 8.9 Hz, 1H), 5.08 – 4.81 (m, 3H), 4.76 – 4.60 (m, 2H), 3.90 – 3.81 (m, 1H), 3.16 – 2.91 (m, 2H), 2.44 – 2.37 (m, 6H), 2.37 – 2.23 (m, 1H), 2.18 – 2.02 (m, 1H), 1.39 (d, J = 7.3 Hz, 9H), 1.08 – 0.90 (m, 6H), 0.90 – 0.71 (m, 6H).
CHLOROFORM-D
f1 (ppm)

(52) Boc-L-Val-L-Lys(Z)-Val^P(OPh-4-SCH$_3$)$_2$

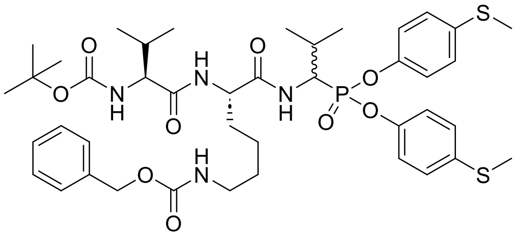

Name: *tert*-butyl ((2S)-1-(((2S)-6-(((benzyloxy)carbonyl)amino)-1-((1-(bis(4-(methylthio)phenoxy)phosphoryl)-2-methylpropyl)amino)-1-oxohexan-2-yl)amino)-3-methyl-1-oxobutan-2-yl)carbamate

^{31}P NMR (162 MHz, Chloroform-d) δ 18.21 (s, 49%), 18.12 (s, 51%).

^{1}H NMR (400 MHz, Chloroform-d) δ 7.39 – 7.26 (m, 5H, 5xArH), 7.22 – 7.12 (m, 4H, 4xArH), 7.12 – 6.95 (m, 5H, 4xArH, NH), 6.82 – 6.68 (m, 1H, NH), 5.48 – 4.97 (m, 4H, CH$_2$, 2xNH), 4.78 – 4.64 (m, 1H, CHP), 4.51 – 4.17 (m, 1H, CH), 4.00 – 3.70 (m, 1H, CH), 3.23 – 2.76 (m, 2H, CH$_2$), 2.46 – 2.30 (m, 7H, 2xSCH$_3$, CH), 2.18 – 2.02 (m, 1H, CH), 1.75 – 1.43 (m, 4H, 2xCH$_2$), 1.40 (d, J = 5.6 Hz, 9H, Boc), 1.38 – 1.25 (m, 2H, CH$_2$), 1.09 – 1.00 (m, 6H, 2xCH$_3$), 0.96 – 0.89 (m, 3H, CH$_3$), 0.88 – 0.81 (m, 3H, CH$_3$).

HRMS: $C_{42}H_{59}N_4O_9PS_2$ [M+Na]$^+$ calc. 881.3359 found 881.3330

HPLC: purity >98%; retention time 19.273 and 19.447 min

Chromatogram

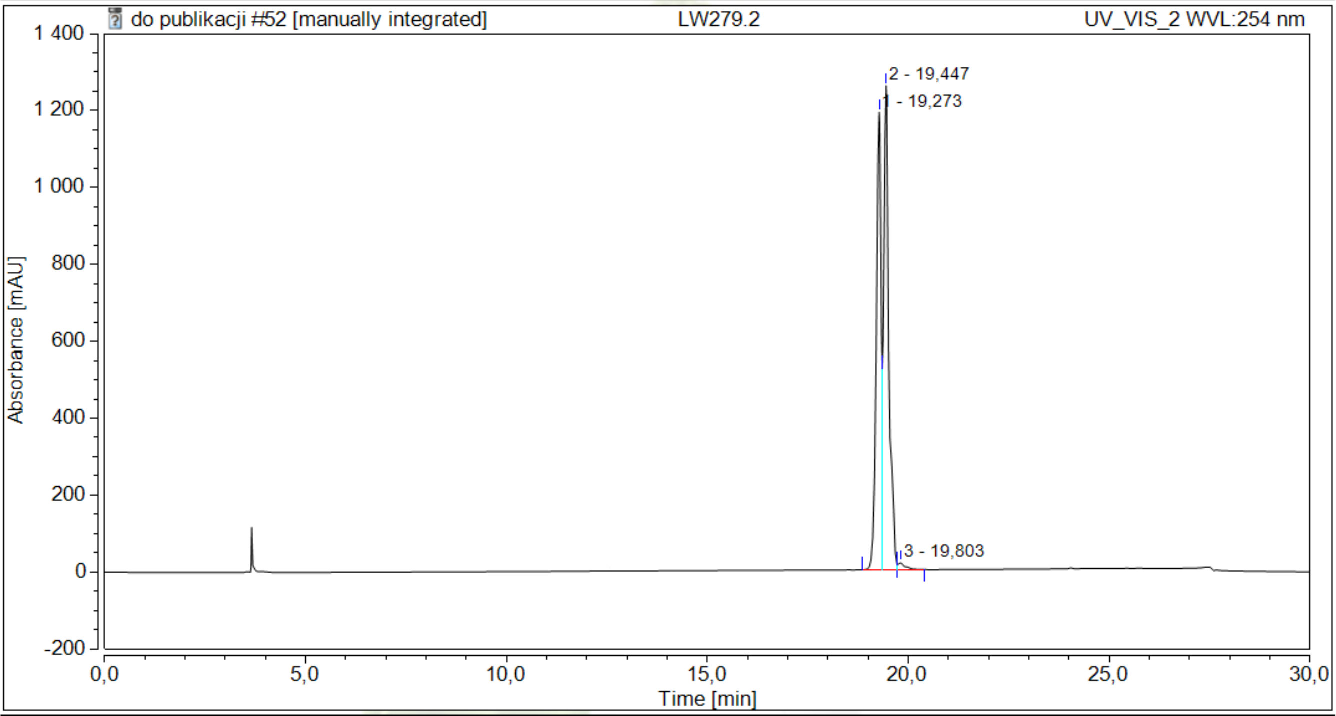

Integration Results

No.	Peak Name	Retention Time min	Area mAU*min	Height mAU	Relative Area %	Relative Height %	Amount n.a.
1		19,273	164,458	1189,753	46,12	48,24	n.a.
2		19,447	188,357	1258,740	52,82	51,03	n.a.
3		19,803	3,809	17,933	1,07	0,73	n.a.
**Total:**			**356,624**	**2466,426**	**100,00**	**100,00**	

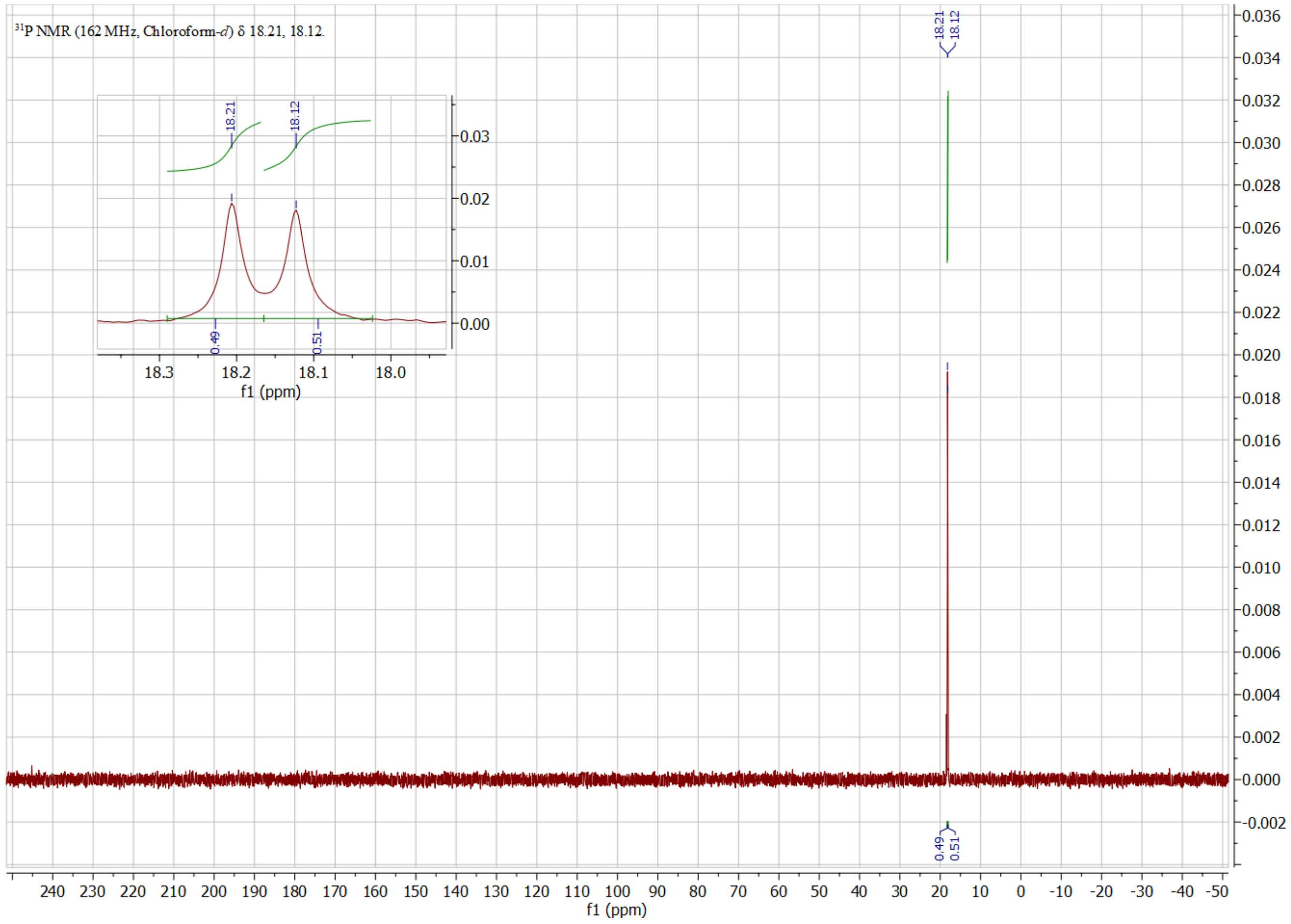

^{31}P NMR (162 MHz, Chloroform-d) δ 18.21, 18.12.
18.21
18.12
0.49
0.51
f1 (ppm)

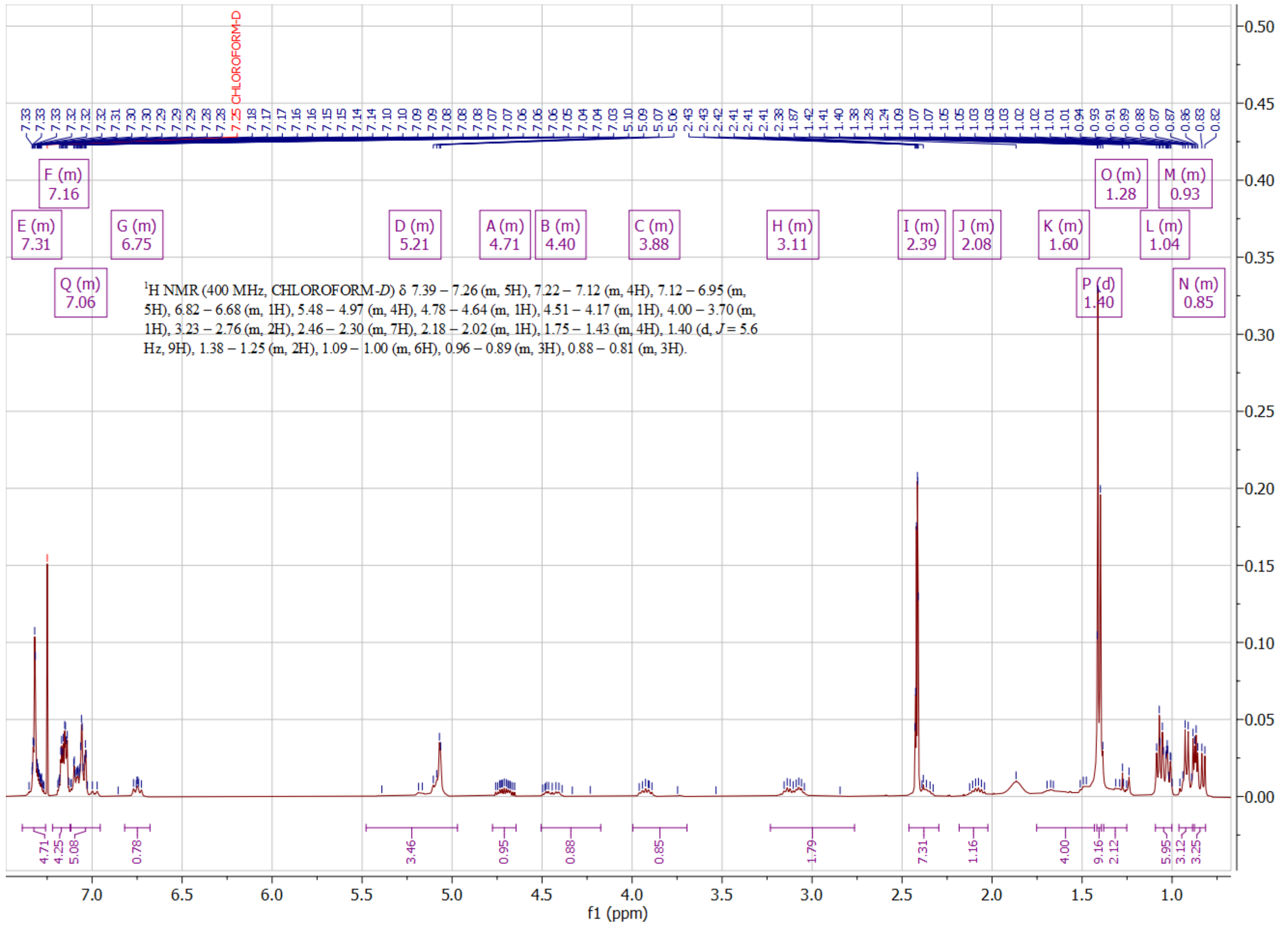

CHLOROFORM-D
E (m) 7.31
F (m) 7.16
Q (m) 7.06
G (m) 6.75
D (m) 5.21
A (m) 4.71
B (m) 4.40
C (m) 3.88
H (m) 3.11
I (m) 2.39
J (m) 2.08
K (m) 1.60
P (d) 1.40
O (m) 1.28
L (m) 1.04
M (m) 0.93
N (m) 0.85
^{1}H NMR (400 MHz, CHLOROFORM-D) δ 7.39 – 7.26 (m, 5H), 7.22 – 7.12 (m, 4H), 7.12 – 6.95 (m, 5H), 6.82 – 6.68 (m, 1H), 5.48 – 4.97 (m, 4H), 4.78 – 4.64 (m, 1H), 4.51 – 4.17 (m, 1H), 4.00 – 3.70 (m, 1H), 3.23 – 2.76 (m, 2H), 2.46 – 2.30 (m, 7H), 2.18 – 2.02 (m, 1H), 1.75 – 1.43 (m, 4H), 1.40 (d, J = 5.6 Hz, 9H), 1.38 – 1.25 (m, 2H), 1.09 – 1.00 (m, 6H), 0.96 – 0.89 (m, 3H), 0.88 – 0.81 (m, 3H).
f1 (ppm)

(53) Boc-L-Val-D-Lys(Z)-Val^P(OPh-4-SCH$_3$)$_2$

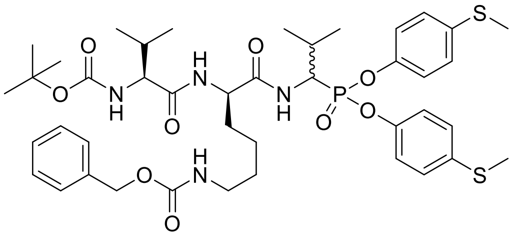

Name: *tert*-butyl ((2S)-1-(((2R)-6-(((benzyloxy)carbonyl)amino)-1-((1-(bis(4-(methylthio)phenoxy)phosphoryl)-2-methylpropyl)amino)-1-oxohexan-2-yl)amino)-3-methyl-1-oxobutan-2-yl)carbamate

^{31}P NMR (162 MHz, Chloroform-d) δ 18.14 (s, 56%), 18.00 (s, 44%).

^{1}H NMR (400 MHz, Chloroform-d) δ 7.36 – 7.26 (m, 6H, 5xArH, NH), 7.18 – 7.11 (m, 4H, 4xArH), 7.09 – 7.01 (m, 4H, 4xArH), 6.98 – 6.80 (m, 1H, NH), 5.33 – 4.98 (m, 4H, CH$_2$, 2xNH), 4.77 – 4.62 (m, 1H, CHP), 4.57 – 4.43 (m, 1H, CH), 4.02 – 3.81 (m, 1H, CH), 3.21 – 2.92 (m, 2H, CH$_2$), 2.40 (dd, J = 5.9, 3.7 Hz, 6H, 2xSCH$_3$), 2.38 – 2.29 (m, 1H, CH), 2.14 – 1.98 (m, 1H, CH), 1.92 – 1.41 (m, 4H, 2xCH$_2$), 1.38 (d, J = 6.6 Hz, 9H, Boc), 1.34 – 1.23 (m, 2H, CH$_2$), 1.05 – 0.98 (m, 6H, 2xCH$_3$), 0.90 (dd, J = 6.8, 2.3 Hz, 3H, CH$_3$), 0.83 (dd, J = 13.2, 6.8 Hz, 3H, CH$_3$).

HRMS: $C_{42}H_{59}N_4O_9PS_2$ [M+Na]$^+$ calc. 881.3359 found 881.3366

HPLC: purity >99%; retention time 19.210 and 19.393 min

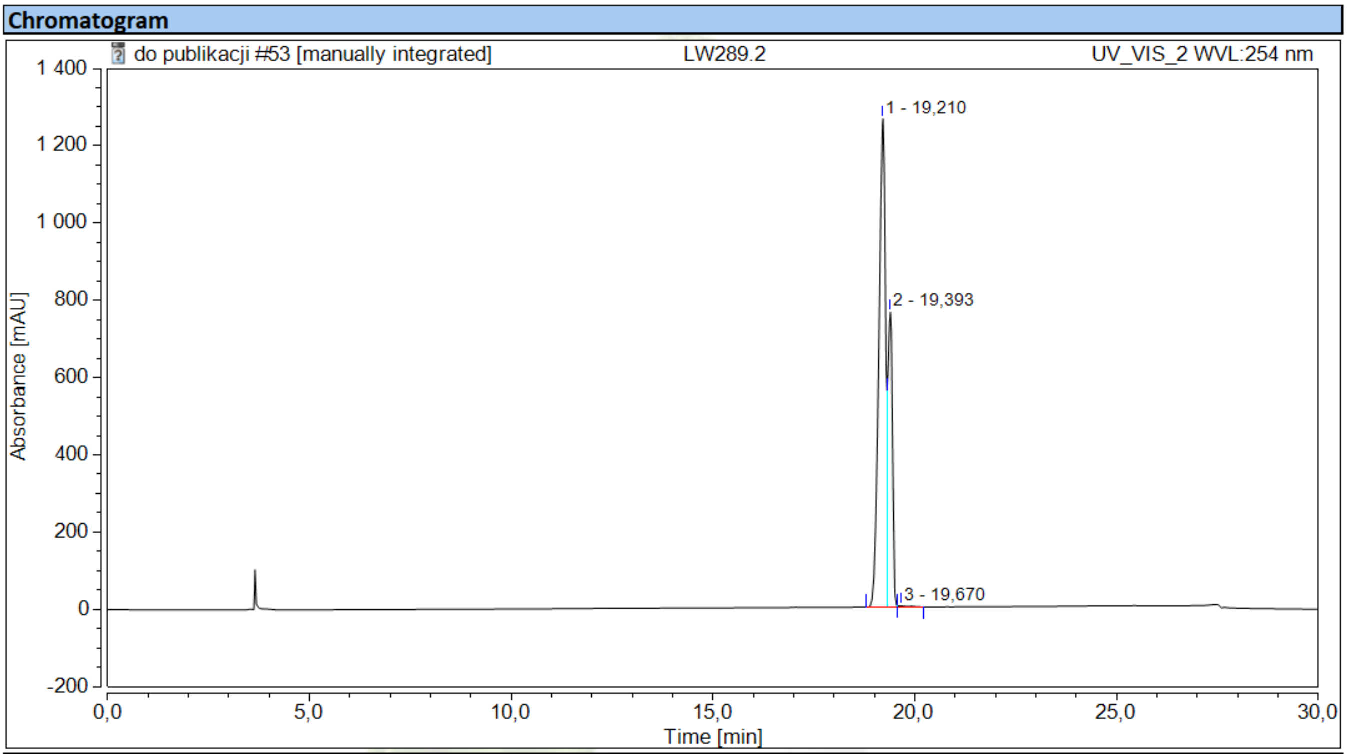

Integration Results

No.	Peak Name	Retention Time min	Area mAU*min	Height mAU	Relative Area %	Relative Height %	Amount n.a.
1		19,210	237,330	1263,966	69,01	62,23	n.a.
2		19,393	105,510	763,671	30,68	37,60	n.a.
3		19,670	1,076	3,348	0,31	0,16	n.a.
**Total:**			**343,916**	**2030,985**	**100,00**	**100,00**	

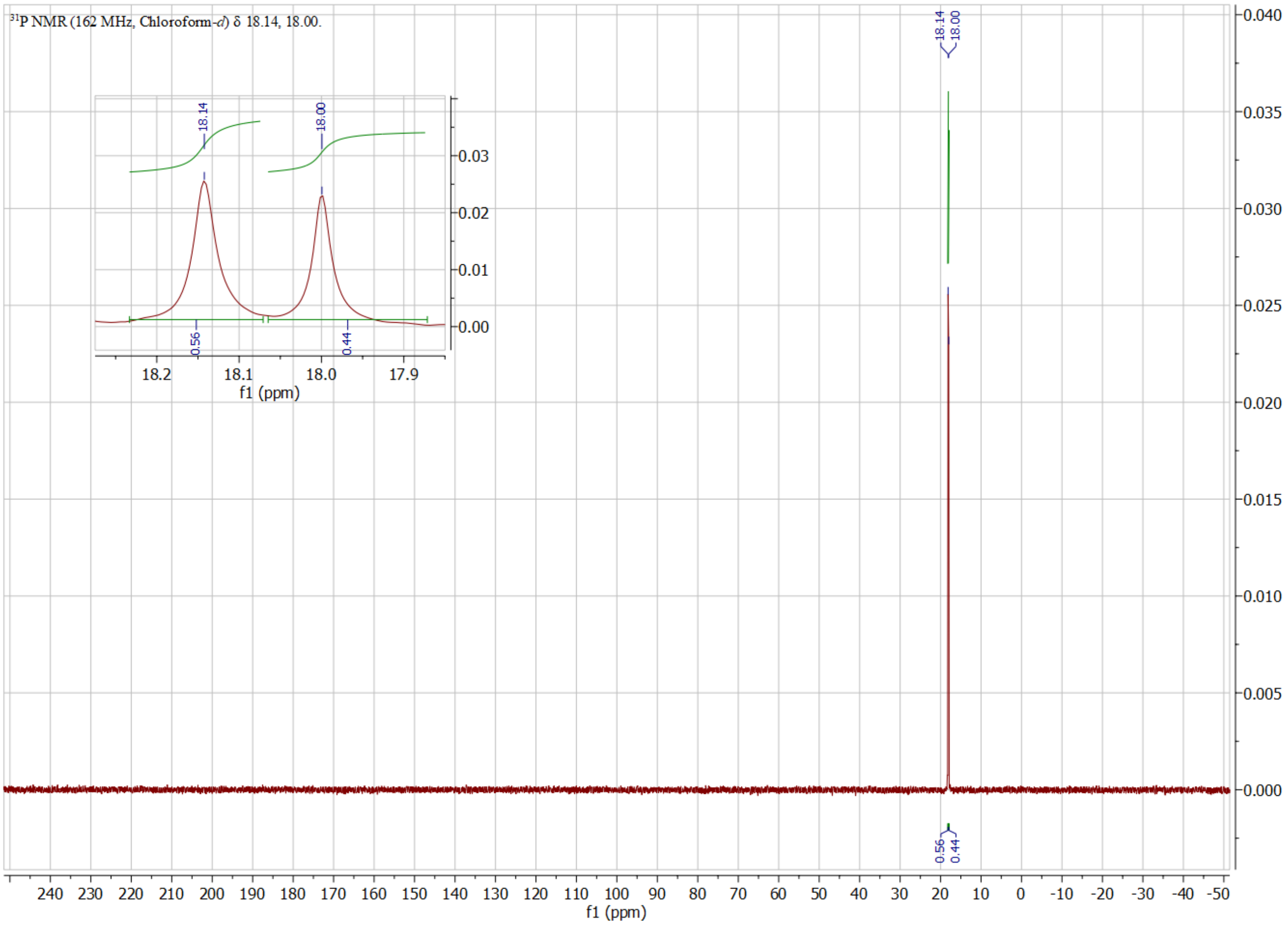

^{31}P NMR (162 MHz, Chloroform-*d*) δ 18.14, 18.00.
f1 (ppm)

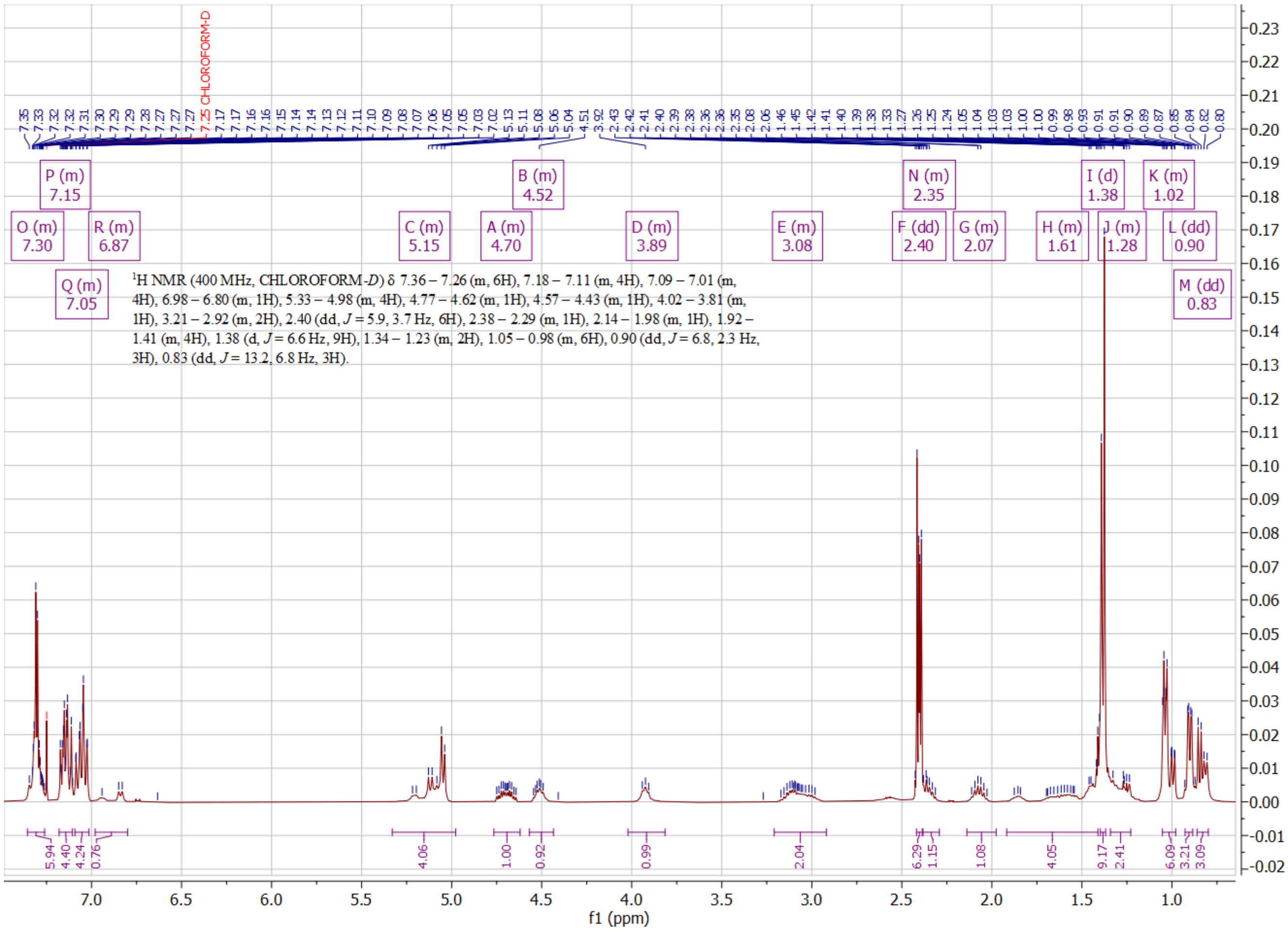

^{1}H NMR (400 MHz, CHLOROFORM-*D*) δ 7.36 – 7.26 (m, 6H), 7.18 – 7.11 (m, 4H), 7.09 – 7.01 (m, 4H), 6.98 – 6.80 (m, 1H), 5.33 – 4.98 (m, 4H), 4.77 – 4.62 (m, 1H), 4.57 – 4.43 (m, 1H), 4.02 – 3.81 (m, 1H), 3.21 – 2.92 (m, 2H), 2.40 (dd, *J* = 5.9, 3.7 Hz, 6H), 2.38 – 2.29 (m, 1H), 2.14 – 1.98 (m, 1H), 1.92 – 1.41 (m, 4H), 1.38 (d, *J* = 6.6 Hz, 9H), 1.34 – 1.23 (m, 2H), 1.05 – 0.98 (m, 6H), 0.90 (dd, *J* = 6.8, 2.3 Hz, 3H), 0.83 (dd, *J* = 13.2, 6.8 Hz, 3H).
f1 (ppm)

(54) 353.2 Boc-L-Val-L-Hyp(Bzl)-Val^P(OPh-4-SCH$_3$)$_2$

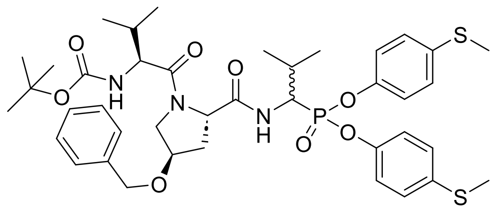

Name: *tert*-butyl ((2S)-1-((2S,4R)-4-(benzyloxy)-2-((1-(bis(4-(methylthio)phenoxy)phosphoryl)-2-methylpropyl)carbamoyl)pyrrolidin-1-yl)-3-methyl-1-oxobutan-2-yl)carbamate

^{31}P NMR (162 MHz, Chloroform-d) δ 18.31 (s, 30%), 17.83 (s, 70%).

^{1}H NMR (400 MHz, Chloroform-d) δ 7.36 – 7.25 (m, 6H, 5xArH, NH), 7.20 – 7.14 (m, 4H, 4xArH), 7.12 – 7.02 (m, 4H, 4xArH), 5.38 (dd, J = 84.6, 9.3 Hz, 1H, NH), 4.78 – 4.65 (m, 1H, CHP), 4.63 – 4.41 (m, 3H, CH$_2$, CH), 4.31 – 4.13 (m, 2H, CH$_2$), 4.11 – 3.97 (m, 1H, CH), 3.72 – 3.54 (m, 1H, CH), 2.46 – 2.40 (m, 6H, 2xSCH$_3$), 2.40 – 2.31 (m, 1H, CH), 2.30 – 2.15 (m, 1H, CH), 2.12 – 2.03 (m, 1H, 0.5xCH$_2$), 2.00 – 1.80 (m, 1H, 0.5xCH$_2$), 1.40 (d, J = 1.9 Hz, 9H, Boc), 1.12 – 1.00 (m, 6H, 2xCH$_3$), 0.96 – 0.81 (m, 6H, 2xCH$_3$).

HRMS: $C_{40}H_{54}N_3O_8PS_2$ [M+Na]$^+$ calc. 822.2988 found 822.2994

HPLC: purity >99%; retention time 20.320 and 20.553 min

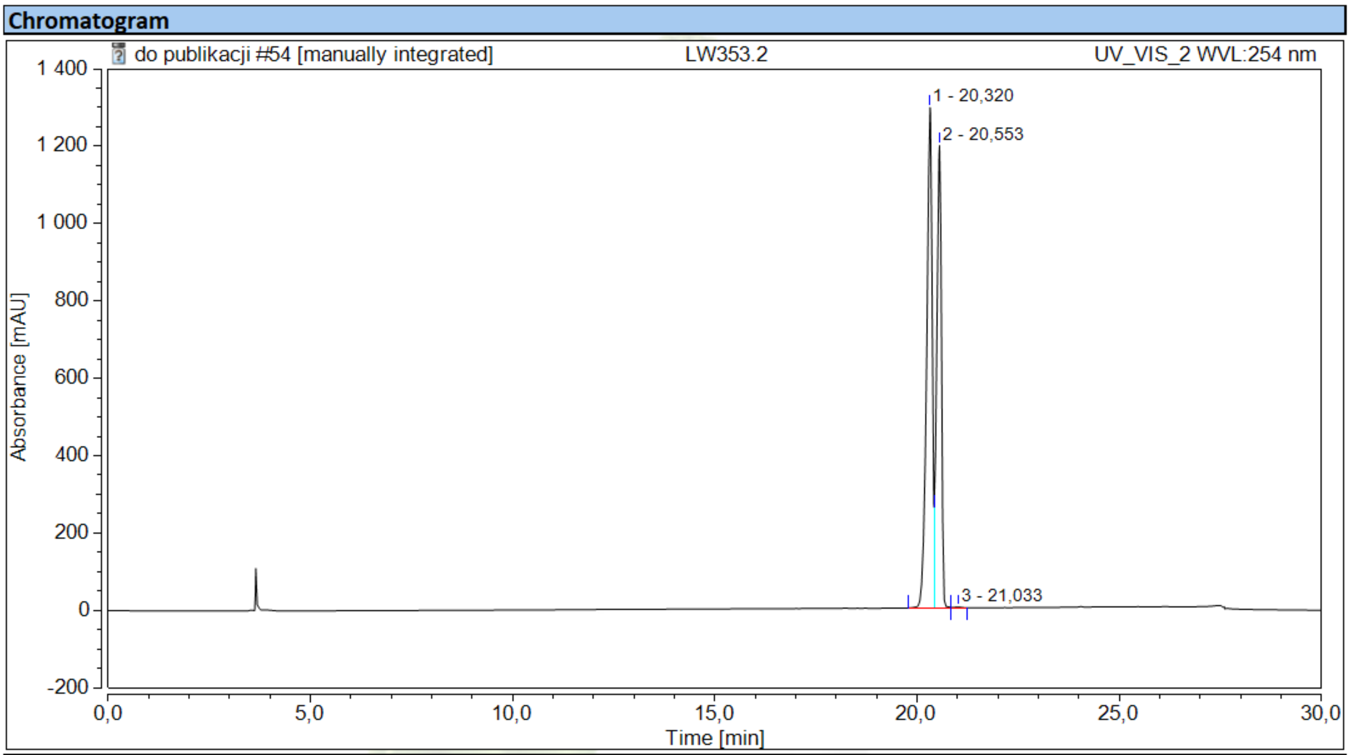

Integration Results

No.	Peak Name	Retention Time min	Area mAU*min	Height mAU	Relative Area %	Relative Height %	Amount n.a.
1		20,320	203,490	1293,574	55,66	51,93	n.a.
2		20,553	161,688	1195,335	44,23	47,99	n.a.
3		21,033	0,421	1,901	0,12	0,08	n.a.
**Total:**			**365,599**	**2490,810**	**100,00**	**100,00**	

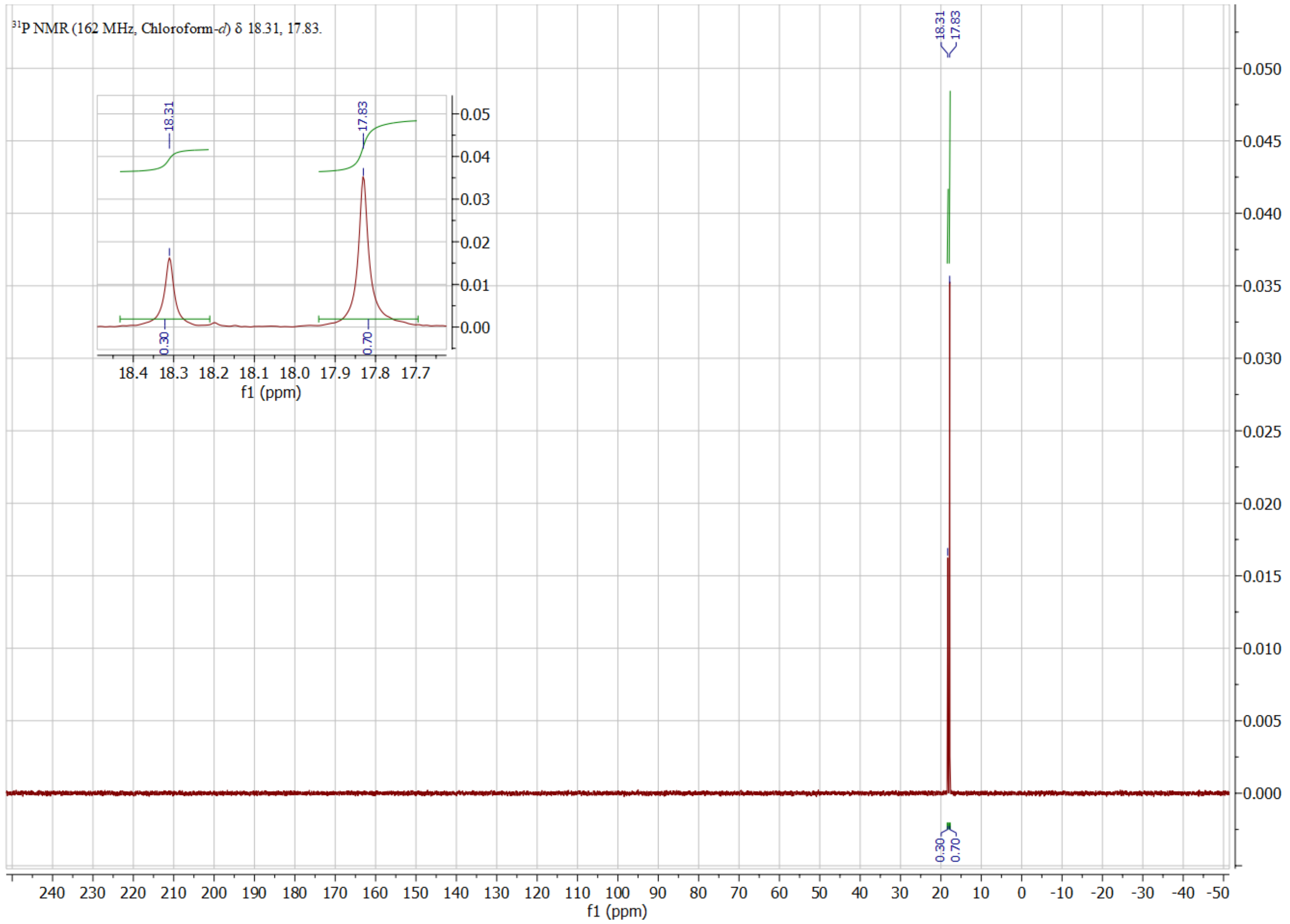
31P NMR (162 MHz, Chloroform-d) δ 18.31, 17.83.
f1 (ppm)

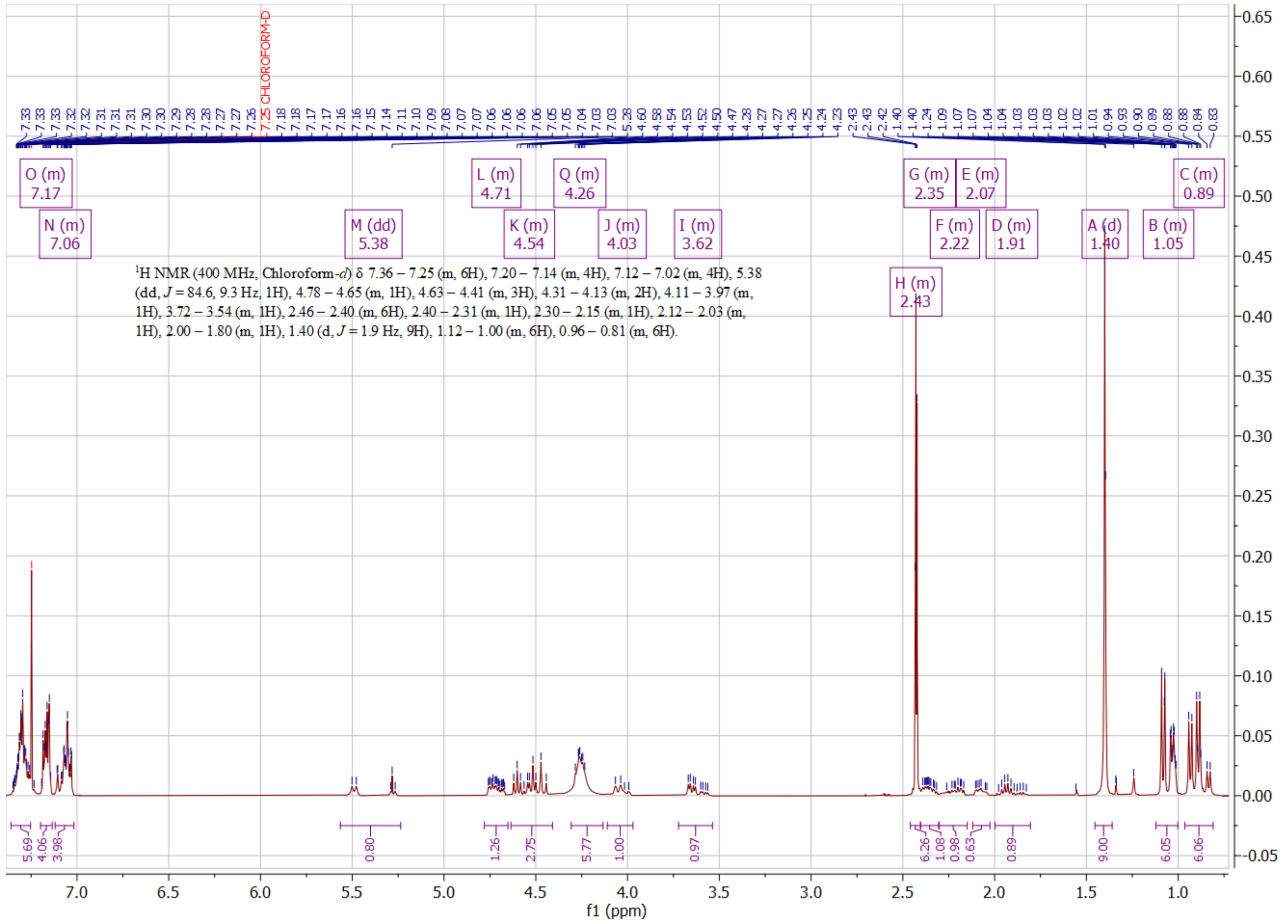
1H NMR (400 MHz, Chloroform-d) δ 7.36 – 7.25 (m, 6H), 7.20 – 7.14 (m, 4H), 7.12 – 7.02 (m, 4H), 5.38 (dd, J = 84.6, 9.3 Hz, 1H), 4.78 – 4.65 (m, 1H), 4.63 – 4.41 (m, 3H), 4.31 – 4.13 (m, 2H), 4.11 – 3.97 (m, 1H), 3.72 – 3.54 (m, 1H), 2.46 – 2.40 (m, 6H), 2.40 – 2.31 (m, 1H), 2.30 – 2.15 (m, 1H), 2.12 – 2.03 (m, 1H), 2.00 – 1.80 (m, 1H), 1.40 (d, J = 1.9 Hz, 9H), 1.12 – 1.00 (m, 6H), 0.96 – 0.81 (m, 6H).
CHLOROFORM-D
f1 (ppm)

(55) Boc-Gly-L-Pro-Val^{P}(OPh-4-SCH$_3$)$_2$

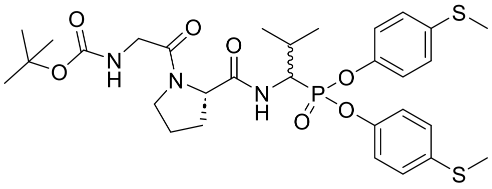

Name: *tert*-butyl (2-((2S)-2-((1-(bis(4-(methylthio)phenoxy)phosphoryl)-2-methylpropyl)carbamoyl)pyrrolidin-1-yl)-2-oxoethyl)carbamate

^{31}P NMR (162 MHz, Chloroform-d) δ 18.38 (s, 63%), 18.11 (s, 37%).

^{1}H NMR (400 MHz, Chloroform-d) δ 7.48 (dd, J = 113.1, 10.3 Hz, 1H, NH), 7.22 – 6.98 (m, 8H, 8xArH), 5.38 (d, J = 21.3 Hz, 1H, NH), 4.75 – 4.46 (m, 2H, CH$_2$), 4.05 – 3.84 (m, 1H, CHP), 3.78 – 3.21 (m, 3H, CH$_2$, CH), 2.43 (d, J = 2.8 Hz, 6H, 2xSCH$_3$), 2.41 – 2.21 (m, 2H, CH$_2$), 2.18 – 1.78 (m, 3H, CH$_2$, CH), 1.43 (s, 9H, Boc), 1.12 – 0.96 (m, 6H, 2xCH$_3$).

HRMS: $C_{30}H_{42}N_3O_7PS_2$ [M+Na]$^+$ calc. 674.2099 found 674.2091

HPLC: purity >99%; retention time 17.170 and 17.377 min

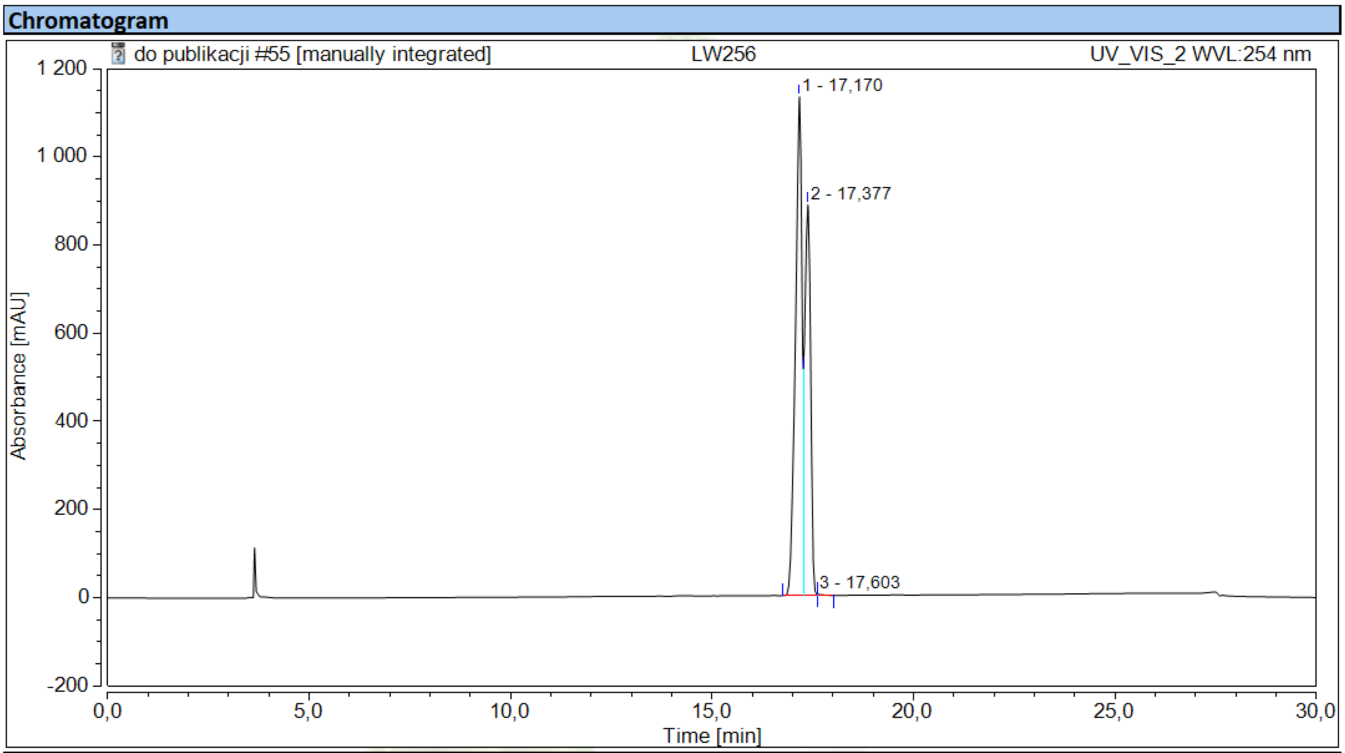

Integration Results

No.	Peak Name	Retention Time min	Area mAU*min	Height mAU	Relative Area %	Relative Height %	Amount n.a.
1		17,170	209,025	1131,362	57,83	56,02	n.a.
2		17,377	152,080	886,066	42,07	43,87	n.a.
3		17,603	0,349	2,116	0,10	0,10	n.a.
**Total:**			**361,453**	**2019,545**	**100,00**	**100,00**	

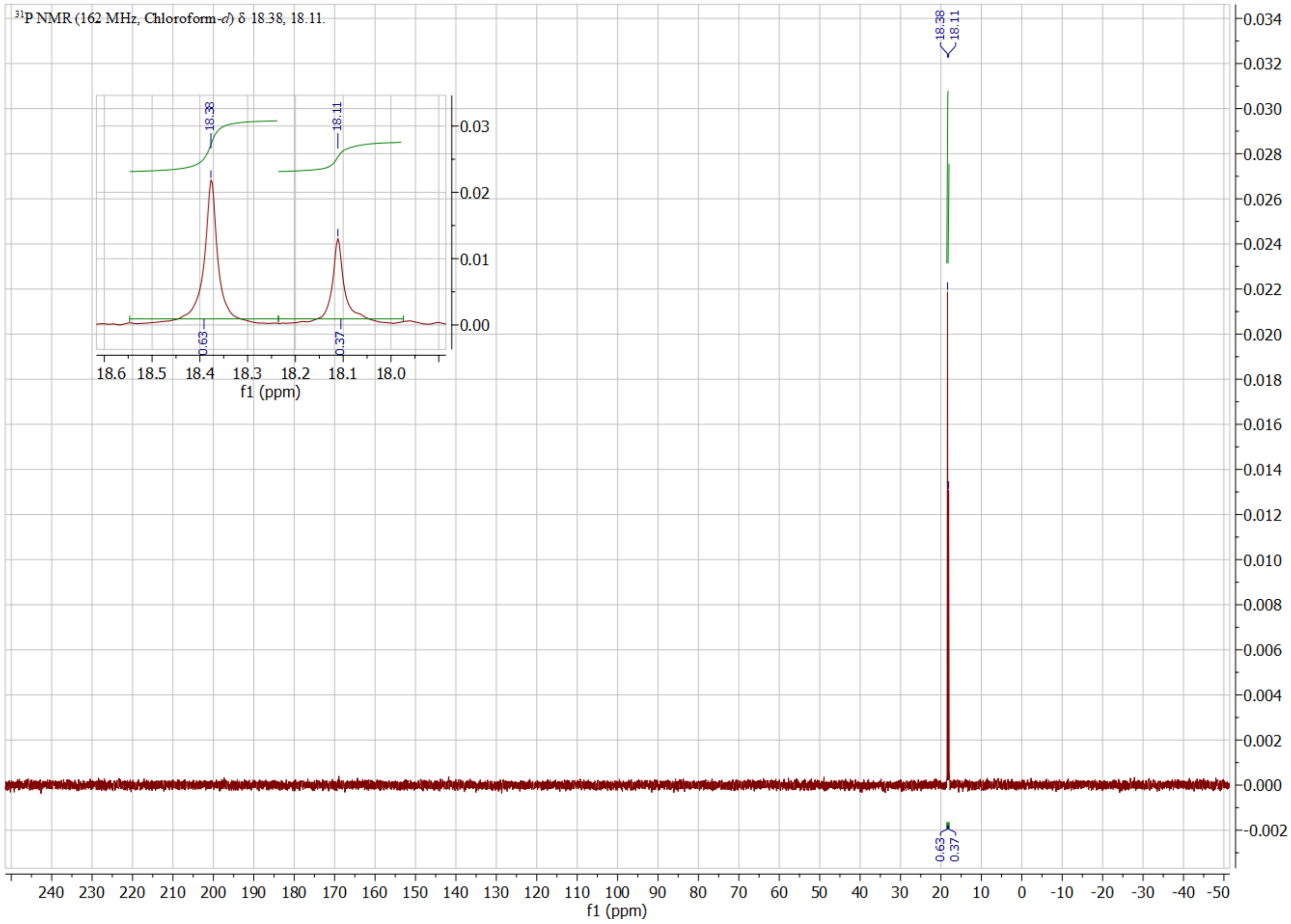
^{31}P NMR (162 MHz, Chloroform-*d*) δ 18.38, 18.11.
f1 (ppm)

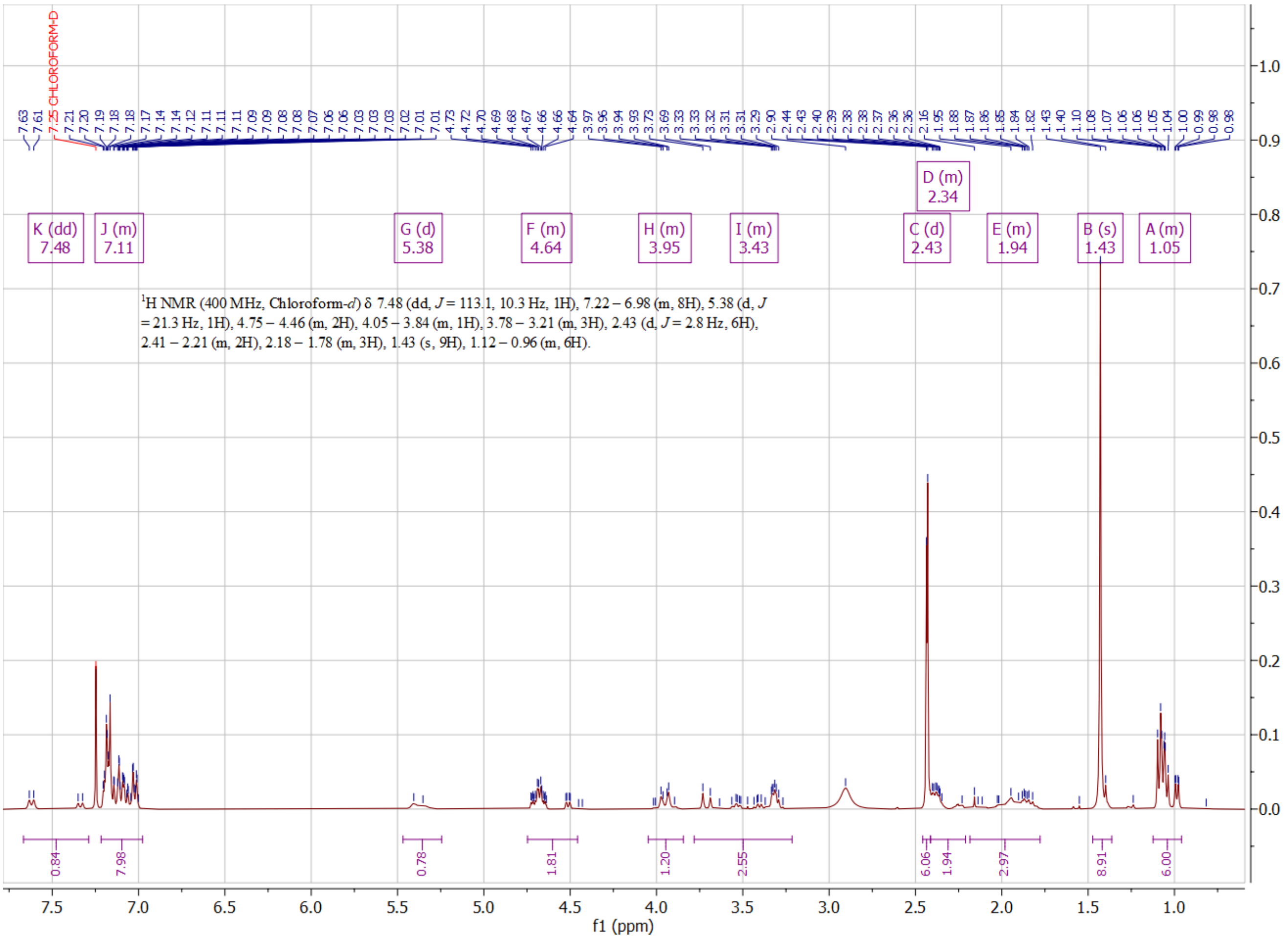
^{1}H NMR (400 MHz, Chloroform-*d*) δ 7.48 (dd, *J* = 113.1, 10.3 Hz, 1H), 7.22 – 6.98 (m, 8H), 5.38 (d, *J* = 21.3 Hz, 1H), 4.75 – 4.46 (m, 2H), 4.05 – 3.84 (m, 1H), 3.78 – 3.21 (m, 3H), 2.43 (d, *J* = 2.8 Hz, 6H), 2.41 – 2.21 (m, 2H), 2.18 – 1.78 (m, 3H), 1.43 (s, 9H), 1.12 – 0.96 (m, 6H).
f1 (ppm)

(56) Boc-L-Ala-L-Pro-Val^P(OPh-4-SCH$_3$)$_2$

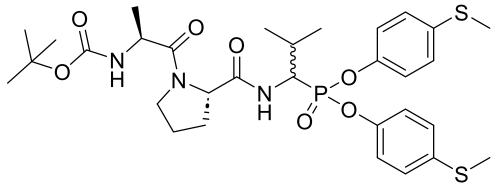

Name: *tert*-butyl ((2S)-1-((2S)-2-((1-(bis(4-(methylthio)phenoxy)phosphoryl)-2-methylpropyl)carbamoyl)pyrrolidin-1-yl)-1-oxopropan-2-yl)carbamate

^{31}P NMR (162 MHz, Chloroform-d) δ 18.31 (s, 54%), 17.97 (s, 46%).

^{1}H NMR (400 MHz, Chloroform-d) δ 7.43 (t, J = 11.4 Hz, 1H, NH), 7.23 – 7.14 (m, 4H, 4xArH), 7.14 – 7.00 (m, 4H, 4xArH), 5.40 (dd, J = 37.1, 8.1 Hz, 1H, NH), 4.77 – 4.53 (m, 2H, CHP, CH), 4.53 – 4.35 (m, 1H, CH), 3.73 – 3.59 (m, 1H, 0.5xCH$_2$), 3.59 – 3.39 (m, 1H, 0.5xCH$_2$), 2.47 – 2.42 (m, 6H, 2xSCH$_3$), 2.42 – 2.16 (m, 2H, CH$_2$), 2.17 – 1.77 (m, 3H, CH$_2$, CH), 1.41 (d, J = 3.0 Hz, 9H, Boc), 1.23 (dd, J = 66.2, 6.9 Hz, 3H, CH$_3$), 1.10 – 0.93 (m, 6H, 3xCH$_3$).

HRMS: $C_{31}H_{44}N_3O_7PS_2$ [M+Na]$^+$ calc. 688.2256 found 688.2241

HPLC: purity >99%; retention time 17.490 and 17.807 min

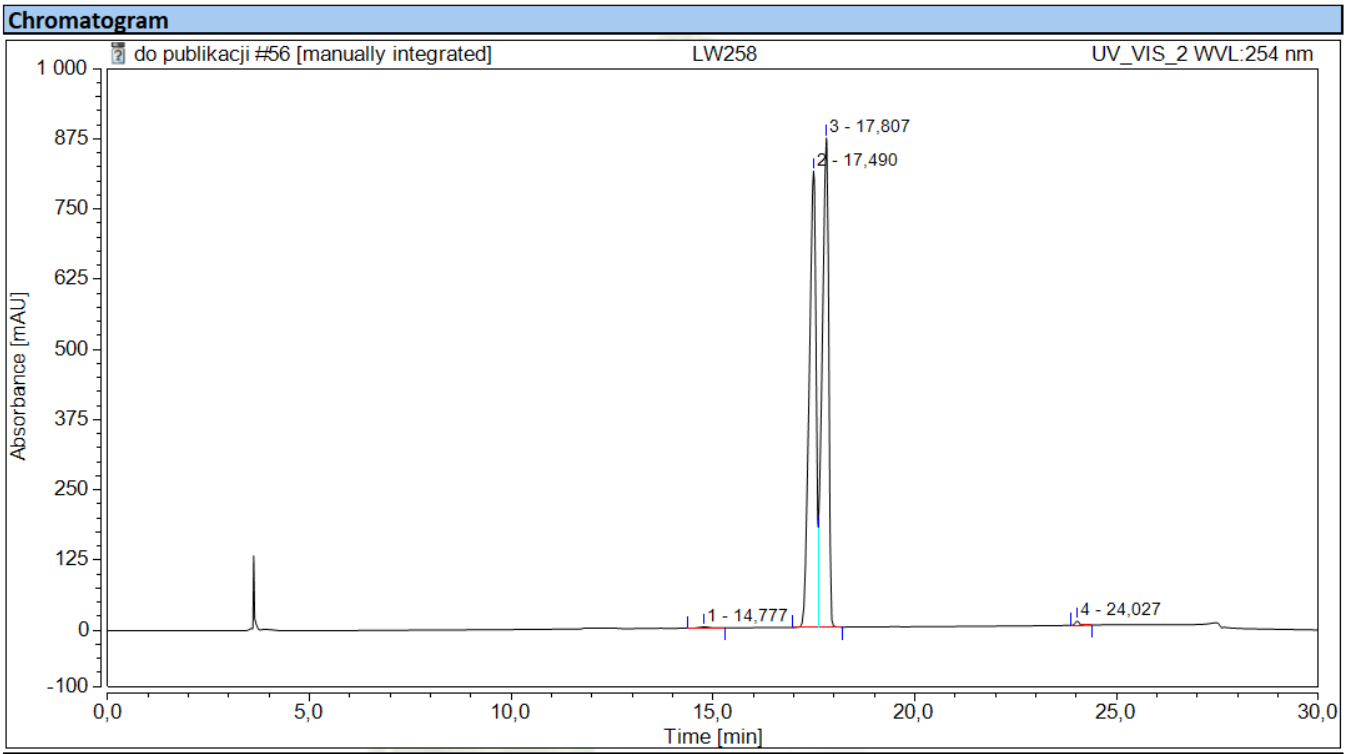

Integration Results

No.	Peak Name	Retention Time min	Area mAU*min	Height mAU	Relative Area %	Relative Height %	Amount n.a.
1		14,777	0,630	2,121	0,19	0,13	n.a.
2		17,490	159,743	812,195	49,26	47,97	n.a.
3		17,807	163,058	870,795	50,28	51,43	n.a.
4		24,027	0,861	7,912	0,27	0,47	n.a.
**Total:**			**324,292**	**1693,023**	**100,00**	**100,00**	

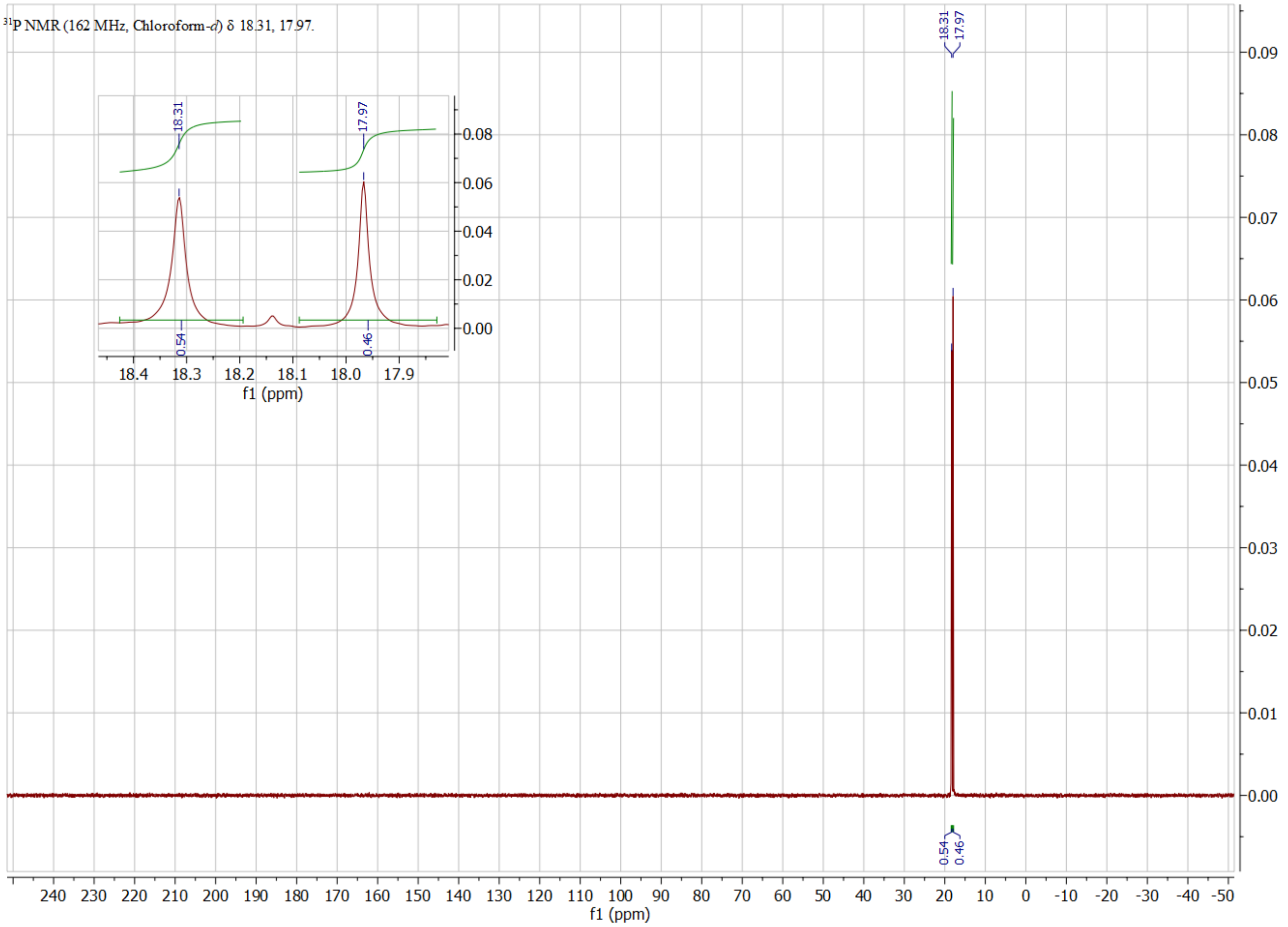
31P NMR (162 MHz, Chloroform-d) δ 18.31, 17.97.
f1 (ppm)

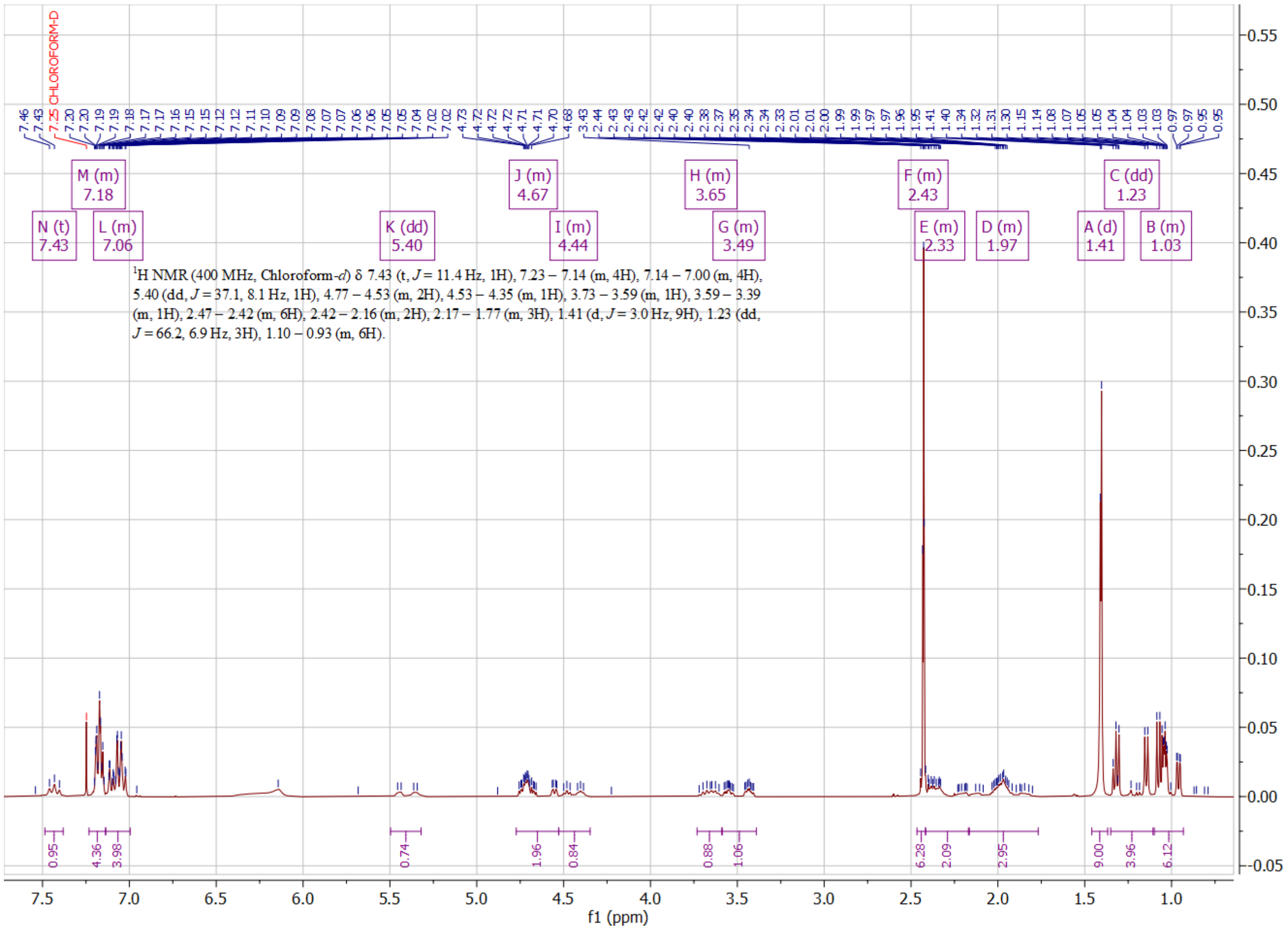
1H NMR (400 MHz, Chloroform-d) δ 7.43 (t, J = 11.4 Hz, 1H), 7.23 – 7.14 (m, 4H), 7.14 – 7.00 (m, 4H), 5.40 (dd, J = 37.1, 8.1 Hz, 1H), 4.77 – 4.53 (m, 2H), 4.53 – 4.35 (m, 1H), 3.73 – 3.59 (m, 1H), 3.59 – 3.39 (m, 1H), 2.47 – 2.42 (m, 6H), 2.42 – 2.16 (m, 2H), 2.17 – 1.77 (m, 3H), 1.41 (d, J = 3.0 Hz, 9H), 1.23 (dd, J = 66.2, 6.9 Hz, 3H), 1.10 – 0.93 (m, 6H).
CHLOROFORM-D
f1 (ppm)

(57) Boc-D-Ala-L-Pro-Val^{P}(OPh-4-SCH$_3$)$_2$

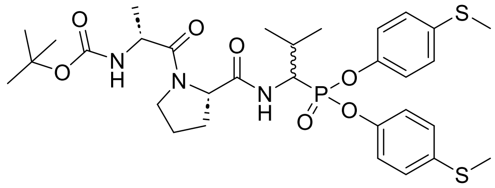

Name: *tert*-butyl ((2R)-1-((2S)-2-((1-(bis(4-(methylthio)phenoxy)phosphoryl)-2-methylpropyl)carbamoyl)pyrrolidin-1-yl)-1-oxopropan-2-yl)carbamate

^{31}P NMR (162 MHz, Chloroform-d) δ 18.57 (s, 67%), 18.25 (s, 33%).

^{1}H NMR (400 MHz, Chloroform-d) δ 7.53 (dd, J = 43.1, 10.3 Hz, 1H, NH), 7.22 – 7.10 (m, 6H, 6xArH), 7.10 – 6.97 (m, 2H, 2xArH), 5.37 – 5.27 (m, 1H, NH), 4.72 – 4.49 (m, 2H, CHP, CH), 4.49 – 4.30 (m, 1H, CH), 3.85 – 3.59 (m, 1H, 0.5xCH$_2$), 3.51 – 3.35 (m, 1H, 0.5xCH$_2$), 2.43 (d, J = 4.7 Hz, 6H, 2xSCH$_3$), 2.40 – 2.24 (m, 2H, CH$_2$), 2.15 – 1.75 (m, 3H, CH$_2$, CH), 1.46 – 1.26 (m, 12H, Boc, CH$_3$), 1.12 – 0.94 (m, 6H, 2xCH$_3$).

HRMS: $C_{31}H_{44}N_3O_7PS_2$ [M+Na]$^+$ calc. 688.2256 found 688.2244

HPLC: purity >99%; retention time 17.693 min

Chromatogram

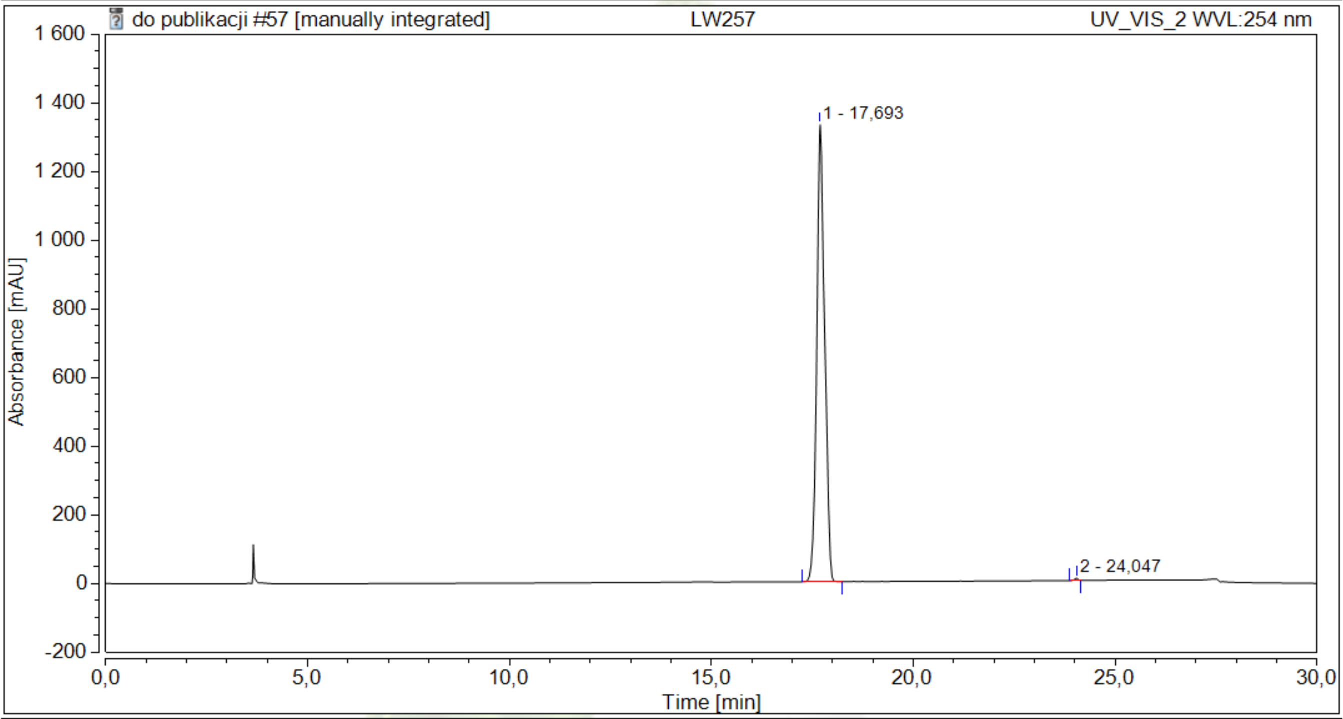

Integration Results

No.	Peak Name	Retention Time min	Area mAU*min	Height mAU	Relative Area %	Relative Height %	Amount n.a.
1		17,693	300,297	1330,908	99,76	99,45	n.a.
2		24,047	0,710	7,375	0,24	0,55	n.a.
**Total:**			**301,007**	**1338,283**	**100,00**	**100,00**	

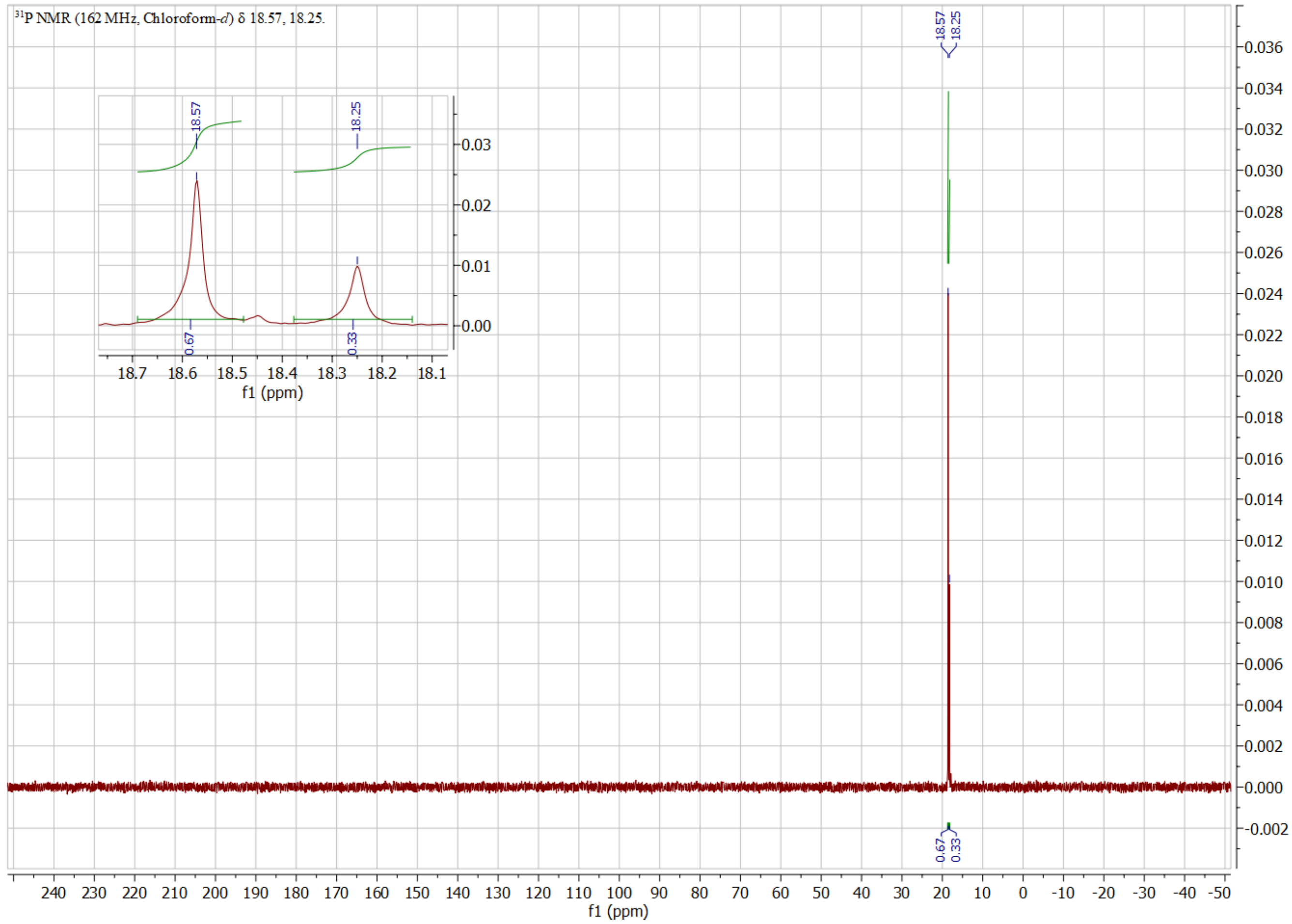

^{31}P NMR (162 MHz, Chloroform-d) δ 18.57, 18.25.
f1 (ppm)

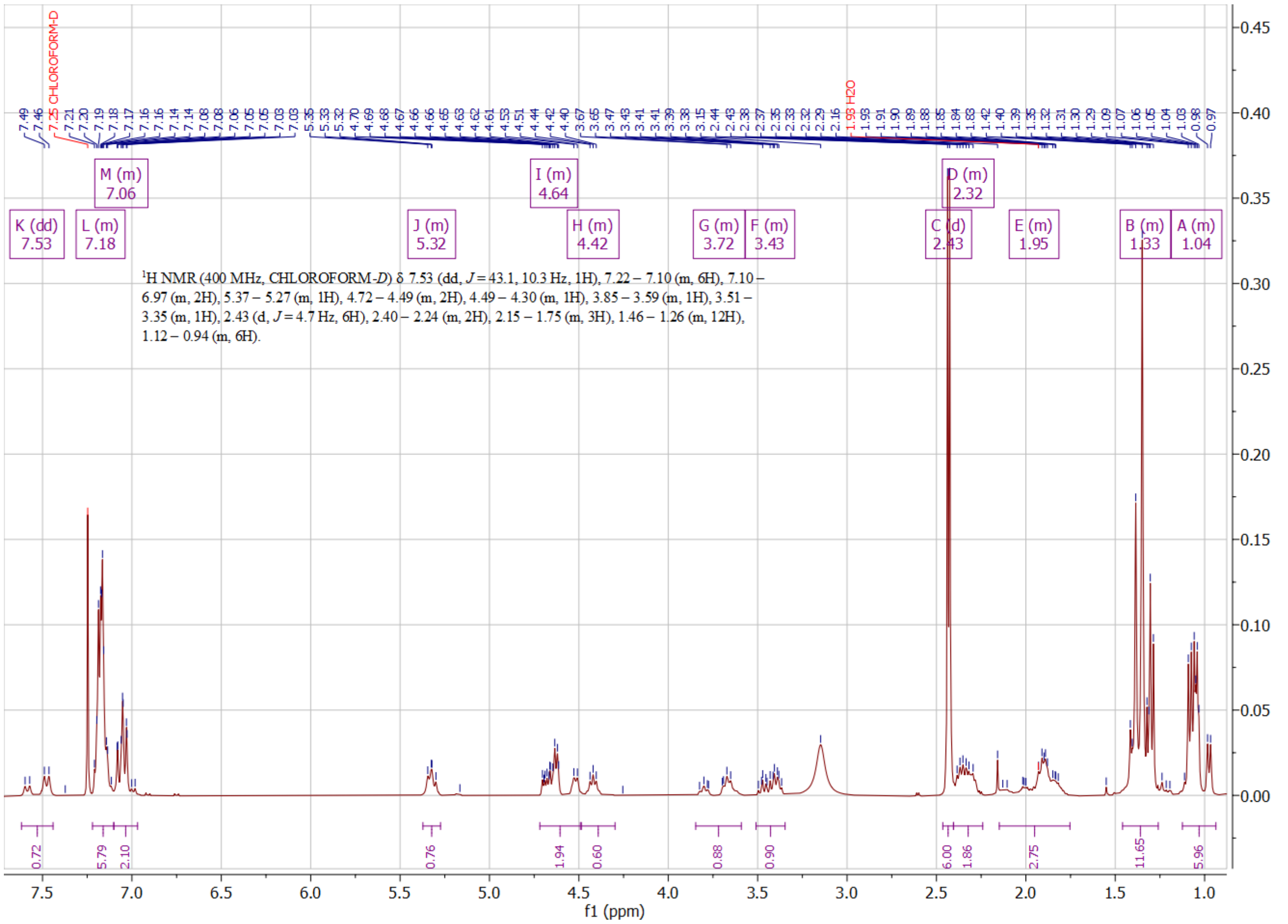

^{1}H NMR (400 MHz, CHLOROFORM-D) δ 7.53 (dd, J = 43.1, 10.3 Hz, 1H), 7.22 – 7.10 (m, 6H), 7.10 – 6.97 (m, 2H), 5.37 – 5.27 (m, 1H), 4.72 – 4.49 (m, 2H), 4.49 – 4.30 (m, 1H), 3.85 – 3.59 (m, 1H), 3.51 – 3.35 (m, 1H), 2.43 (d, J = 4.7 Hz, 6H), 2.40 – 2.24 (m, 2H), 2.15 – 1.75 (m, 3H), 1.46 – 1.26 (m, 12H), 1.12 – 0.94 (m, 6H).
f1 (ppm)

(58) Boc-Aib-L-Pro- Val^{P}(OPh-4-SCH$_3$)$_2$

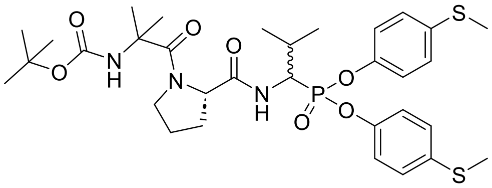

Name: tert-butyl (1-((2S)-2-((1-(bis(4-(methylthio)phenoxy)phosphoryl)-2-methylpropyl)carbamoyl)pyrrolidin-1-yl)-2-methyl-1-oxopropan-2-yl)carbamate

^{31}P NMR (162 MHz, Chloroform-d) δ 19.13 (s).1H NMR

^{1}H NMR (400 MHz, Chloroform-d) δ 7.71 (d, J = 9.6 Hz, 1H, NH), 7.21 – 7.12 (m, 4H, 4xArH), 7.10 – 6.99 (m, 4H, 4xArH), 4.79 (s, 1H, NH), 4.74 – 4.56 (m, 2H, CHP, CH), 3.84 – 3.73 (m, 1H, 0.5xCH$_2$), 3.52 – 3.41 (m, 1H, 0.5xCH$_2$), 2.48 – 2.37 (m, 7H, 2xSCH$_3$, CH), 2.26 – 2.14 (m, 1H, 0.5xCH$_2$), 1.92 – 1.65 (m, 3H, 1.5xCH$_2$), 1.50 (s, 3H, CH$_3$), 1.38 (s, 3H, CH$_3$), 1.35 (s, 9H, Boc), 1.16 (dd, J = 10.5, 6.8 Hz, 6H, 2xCH$_3$).

HRMS: $C_{32}H_{46}N_3O_7PS_2$ [M+Na]$^+$ calc. 702.2412 found 702.2403

HPLC: purity >99%; retention time 18.090 and 18.357 min

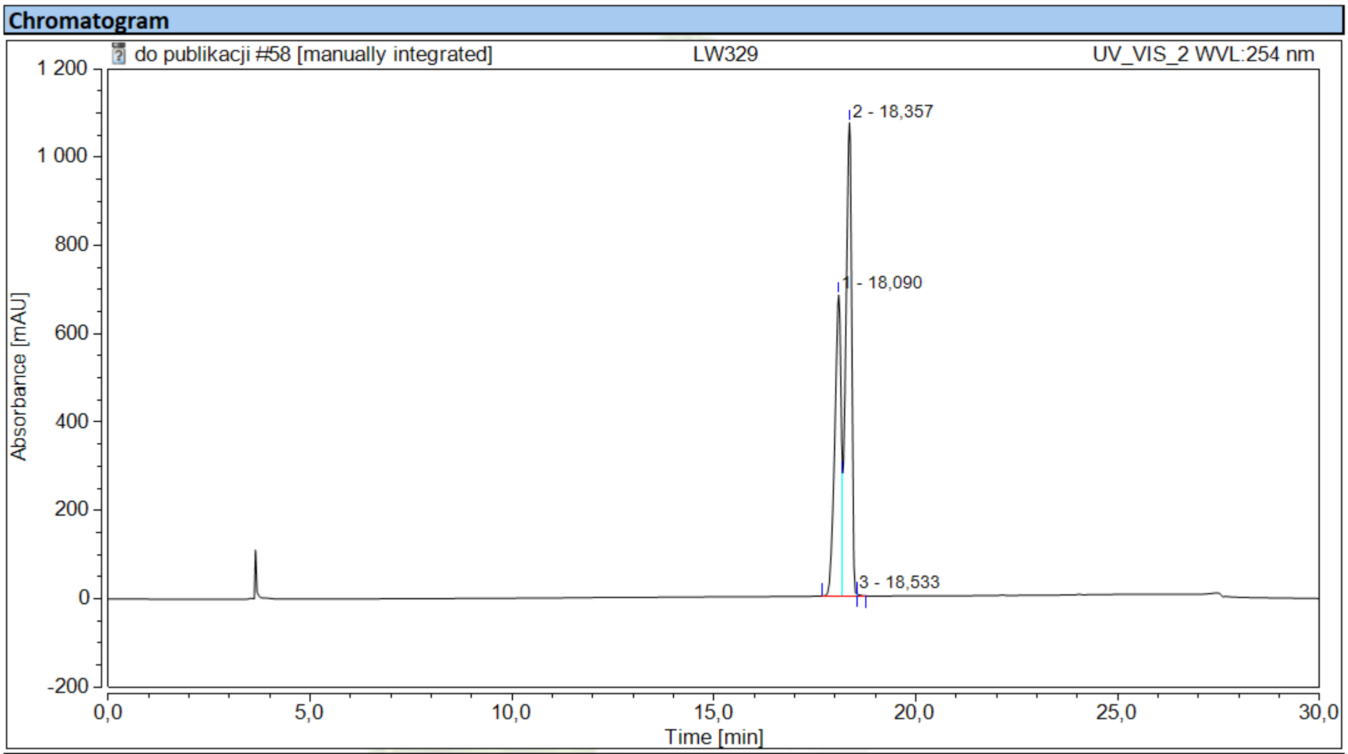

Integration Results

No.	Peak Name	Retention Time min	Area mAU*min	Height mAU	Relative Area %	Relative Height %	Amount n.a.
1		18,090	125,510	682,107	41,05	38,81	n.a.
2		18,357	179,879	1071,643	58,84	60,97	n.a.
3		18,533	0,343	3,934	0,11	0,22	n.a.
**Total:**			**305,732**	**1757,685**	**100,00**	**100,00**	

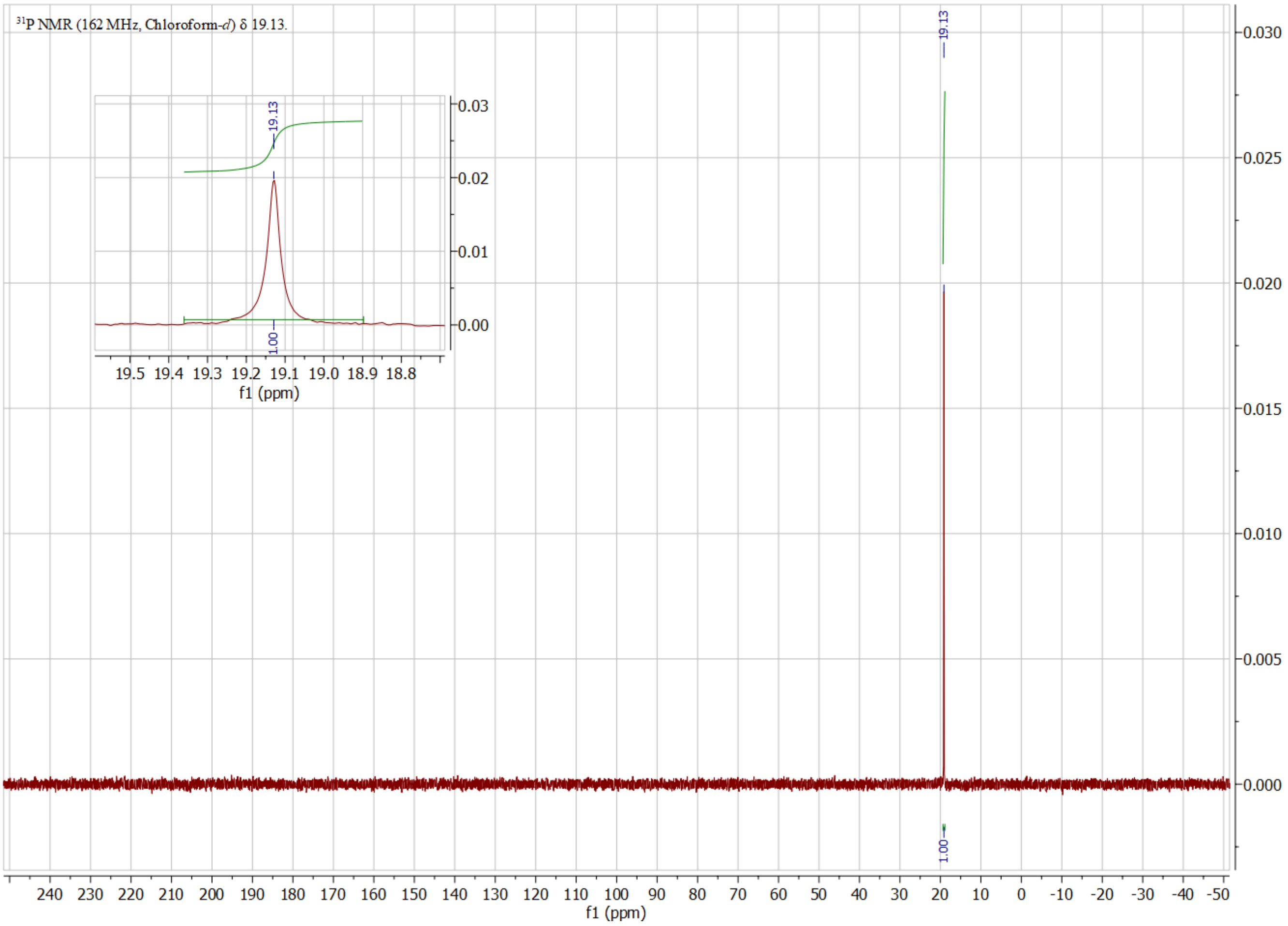
^{31}P NMR (162 MHz, Chloroform-d) δ 19.13.
f1 (ppm)

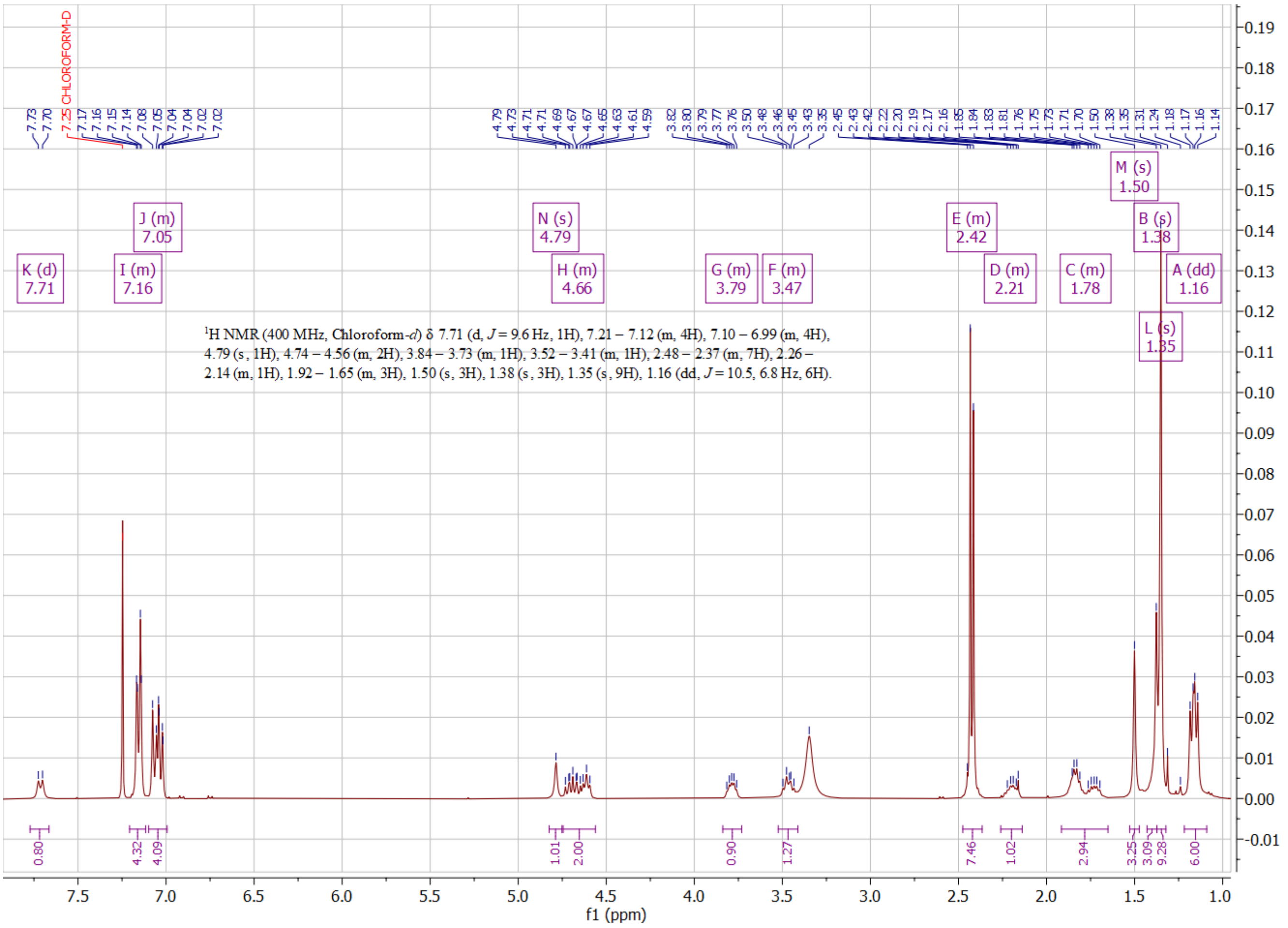
^{1}H NMR (400 MHz, Chloroform-d) δ 7.71 (d, J = 9.6 Hz, 1H), 7.21 – 7.12 (m, 4H), 7.10 – 6.99 (m, 4H), 4.79 (s, 1H), 4.74 – 4.56 (m, 2H), 3.84 – 3.73 (m, 1H), 3.52 – 3.41 (m, 1H), 2.48 – 2.37 (m, 7H), 2.26 – 2.14 (m, 1H), 1.92 – 1.65 (m, 3H), 1.50 (s, 3H), 1.38 (s, 3H), 1.35 (s, 9H), 1.16 (dd, J = 10.5, 6.8 Hz, 6H).
f1 (ppm)

(59) Boc-AC3C-L-Pro- Val^P(OPh-4-SCH$_3$)$_2$

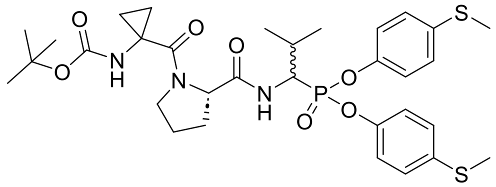

Name: *tert*-butyl (1-((2S)-2-((1-(bis(4-(methylthio)phenoxy)phosphoryl)-2-methylpropyl)carbamoyl)pyrrolidine-1-carbonyl)cyclopropyl)carbamate

^{31}P NMR (162 MHz, Chloroform-d) δ 18.71 (s, 75%), 18.27 (s, 25%).

^{1}H NMR (400 MHz, Chloroform-d) δ 7.50 (d, J = 10.1 Hz, 1, NH), 7.21 – 7.01 (m, 8H, 8xArH), 5.10 (s, 1H, NH), 4.76 – 4.60 (m, 2H, CHP, CH), 3.79 – 3.56 (m, 2H, CH$_2$), 2.43 (d, J = 3.4 Hz, 6H, 2xSCH$_3$), 2.42 – 2.32 (m, 1H, CH), 2.21 – 1.94 (m, 2H, CH$_2$), 1.96 – 1.58 (m, 3H, 1.5xCH$_2$), 1.39 (d, J = 14.6 Hz, 9H, Boc), 1.34 – 1.23 (m, 1H, 0.5xCH$_2$), 1.09 (dd, J = 17.9, 6.8 Hz, 6H, 3xCH$_3$), 1.04 – 0.78 (m, 2H, CH$_2$).

HRMS: $C_{32}H_{44}N_3O_7PS_2$ [M+Na]$^+$ calc. 700.2256 found 700.2229

HPLC: purity >99%; retention time 17.613 and 17.860 min

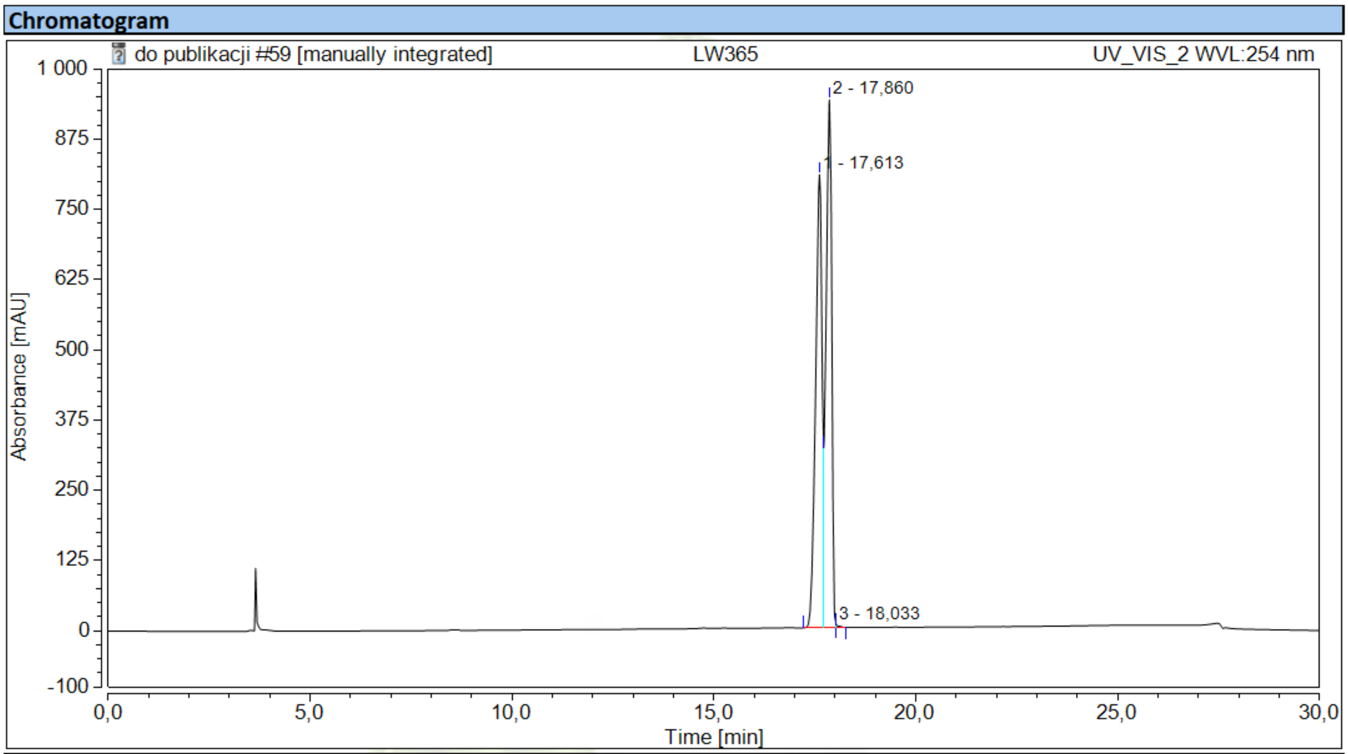

Integration Results

No.	Peak Name	Retention Time min	Area mAU*min	Height mAU	Relative Area %	Relative Height %	Amount n.a.
1		17,613	147,528	806,259	49,03	46,09	n.a.
2		17,860	152,991	939,103	50,85	53,68	n.a.
3		18,033	0,365	4,110	0,12	0,23	n.a.
**Total:**			**300,885**	**1749,472**	**100,00**	**100,00**	

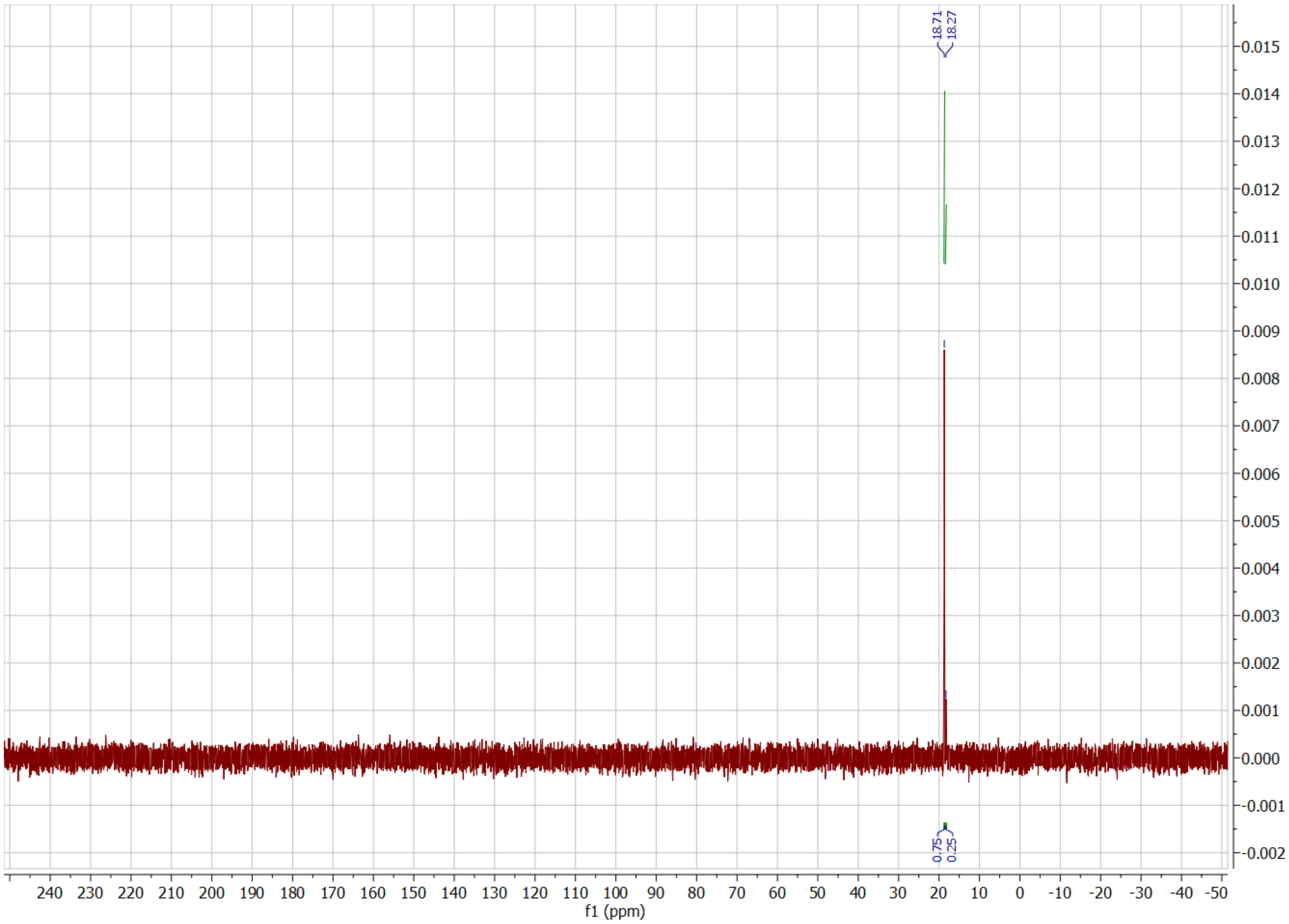
18.71
18.27
0.75
0.25
0.015
0.014
0.013
0.012
0.011
0.010
0.009
0.008
0.007
0.006
0.005
0.004
0.003
0.002
0.001
0.000
-0.001
-0.002
240 230 220 210 200 190 180 170 160 150 140 130 120 110 100 90 80 70 60 50 40 30 20 10 0 -10 -20 -30 -40 -50
f1 (ppm)

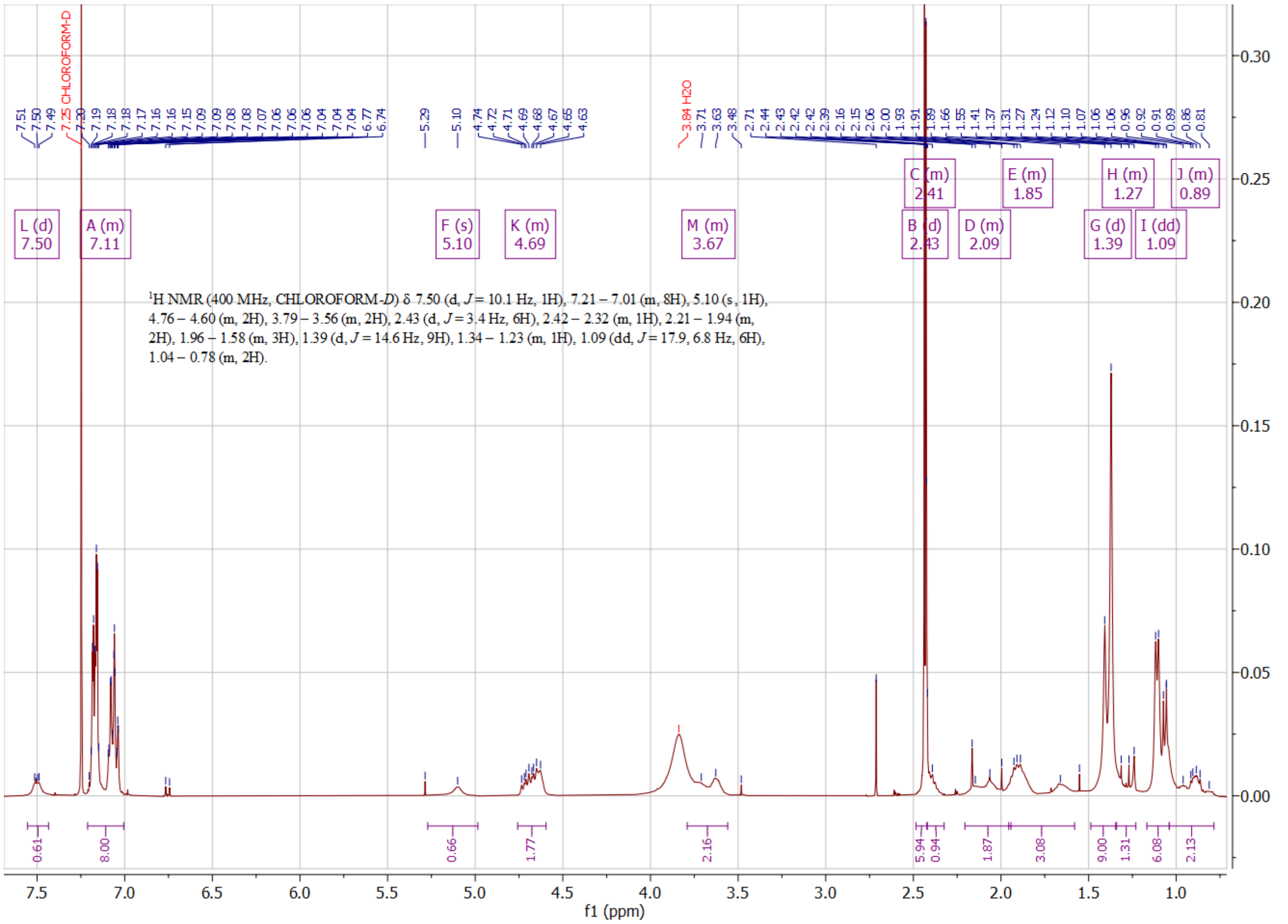
7.25 CHLOROFORM-D
3.84 H2O
L (d) 7.50
A (m) 7.11
F (s) 5.10
K (m) 4.69
M (m) 3.67
C (m) 2.41
B (d) 2.43
D (m) 2.09
E (m) 1.85
G (d) 1.39
H (m) 1.27
I (dd) 1.09
J (m) 0.89
^{1}H NMR (400 MHz, CHLOROFORM-*D*) δ 7.50 (d, *J* = 10.1 Hz, 1H), 7.21 – 7.01 (m, 8H), 5.10 (s, 1H), 4.76 – 4.60 (m, 2H), 3.79 – 3.56 (m, 2H), 2.43 (d, *J* = 3.4 Hz, 6H), 2.42 – 2.32 (m, 1H), 2.21 – 1.94 (m, 2H), 1.96 – 1.58 (m, 3H), 1.39 (d, *J* = 14.6 Hz, 9H), 1.34 – 1.23 (m, 1H), 1.09 (dd, *J* = 17.9, 6.8 Hz, 6H), 1.04 – 0.78 (m, 2H).
0.61
8.00
0.66
1.77
2.16
5.94
0.94
1.87
3.08
9.00
1.31
6.08
2.13
7.5 7.0 6.5 6.0 5.5 5.0 4.5 4.0 3.5 3.0 2.5 2.0 1.5 1.0
f1 (ppm)

(60) Boc-beta-Ala-L-Pro- Val^{P}(OPh-4-SCH$_3$)$_2$

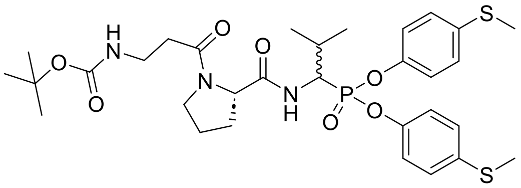

Name: *tert*-butyl (3-((2S)-2-((1-(bis(4-(methylthio)phenoxy)phosphoryl)-2-methylpropyl)carbamoyl)pyrrolidin-1-yl)-3-oxopropyl)carbamate

^{31}P NMR (162 MHz, Chloroform-d) δ 18.18 (s, 51%), 18.04 (s, 49%).

^{1}H NMR (400 MHz, Chloroform-d) δ 1H NMR (400 MHz, CHLOROFORM-D) δ 7.66 (dd, J = 101.1, 10.1 Hz, 1H, NH), 7.23 – 6.98 (m, 8H, 8xArH), 5.66 – 5.26 (m, 1H, NH), 4.78 – 4.66 (m, 1H, CHP), 4.66 – 4.50 (m, 1H, CH), 3.65 – 3.26 (m, 4H, 2xCH$_2$), 2.62 – 2.46 (m, 1H, CH), 2.46 – 2.42 (m, 6H, 2xSCH$_3$), 2.42 – 1.95 (m, 4H, 2xCH$_2$), 1.95 – 1.78 (m, 2H, CH$_2$), 1.39 (d, J = 10.6 Hz, 9H, Boc), 1.14 – 0.91 (m, 6H, 2xCH$_3$).

HRMS: $C_{31}H_{44}N_3O_7PS_2$ [M+Na]$^+$ calc. 688.2256 found 688.2258

HPLC: purity >99%; retention time 17.330 and 17.627 min

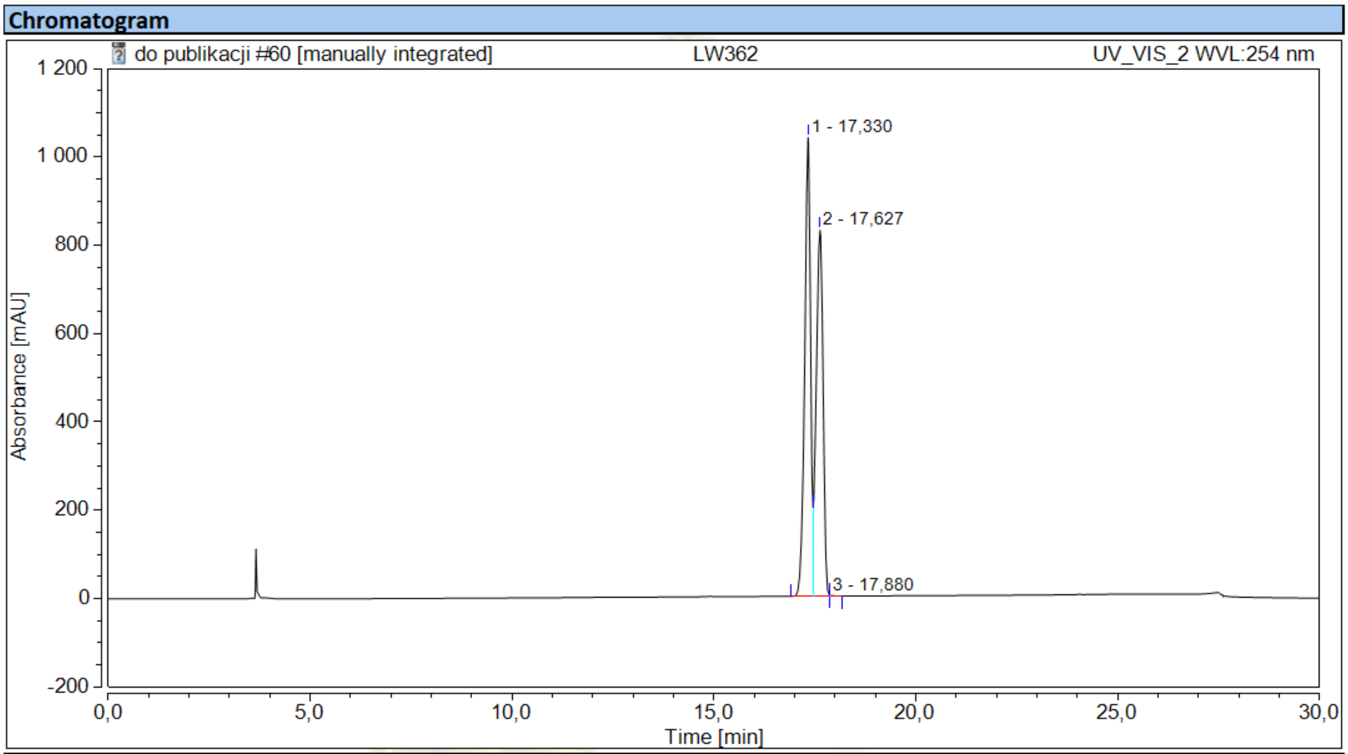

Integration Results

No.	Peak Name	Retention Time min	Area mAU*min	Height mAU	Relative Area %	Relative Height %	Amount n.a.
1		17,330	173,059	1038,616	51,36	55,59	n.a.
2		17,627	163,741	828,615	48,60	44,35	n.a.
3		17,880	0,128	1,199	0,04	0,06	n.a.
**Total:**			**336,928**	**1868,431**	**100,00**	**100,00**	

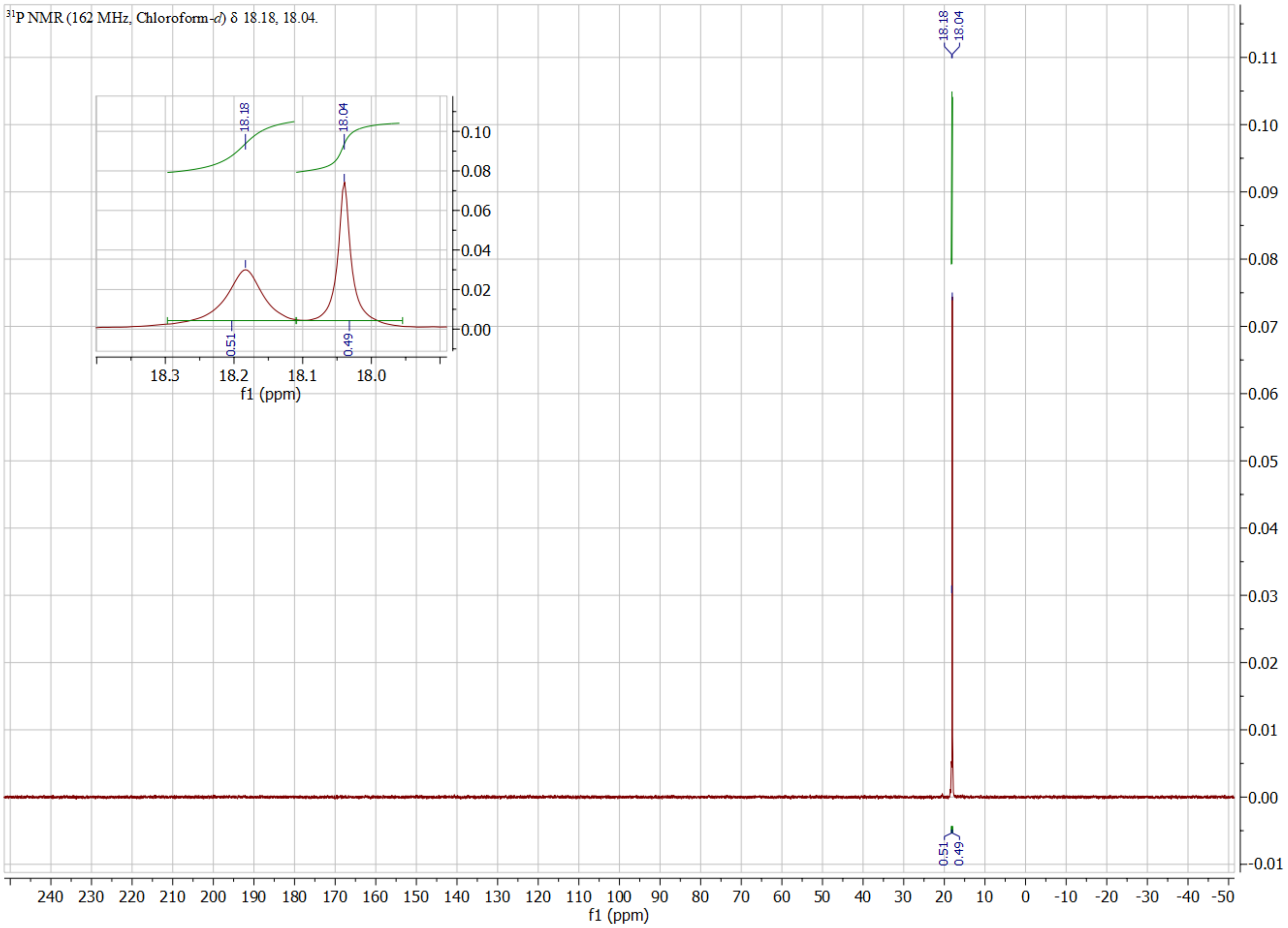
31P NMR (162 MHz, Chloroform-d) δ 18.18, 18.04.
18.18
18.04
0.51
0.49
f1 (ppm)

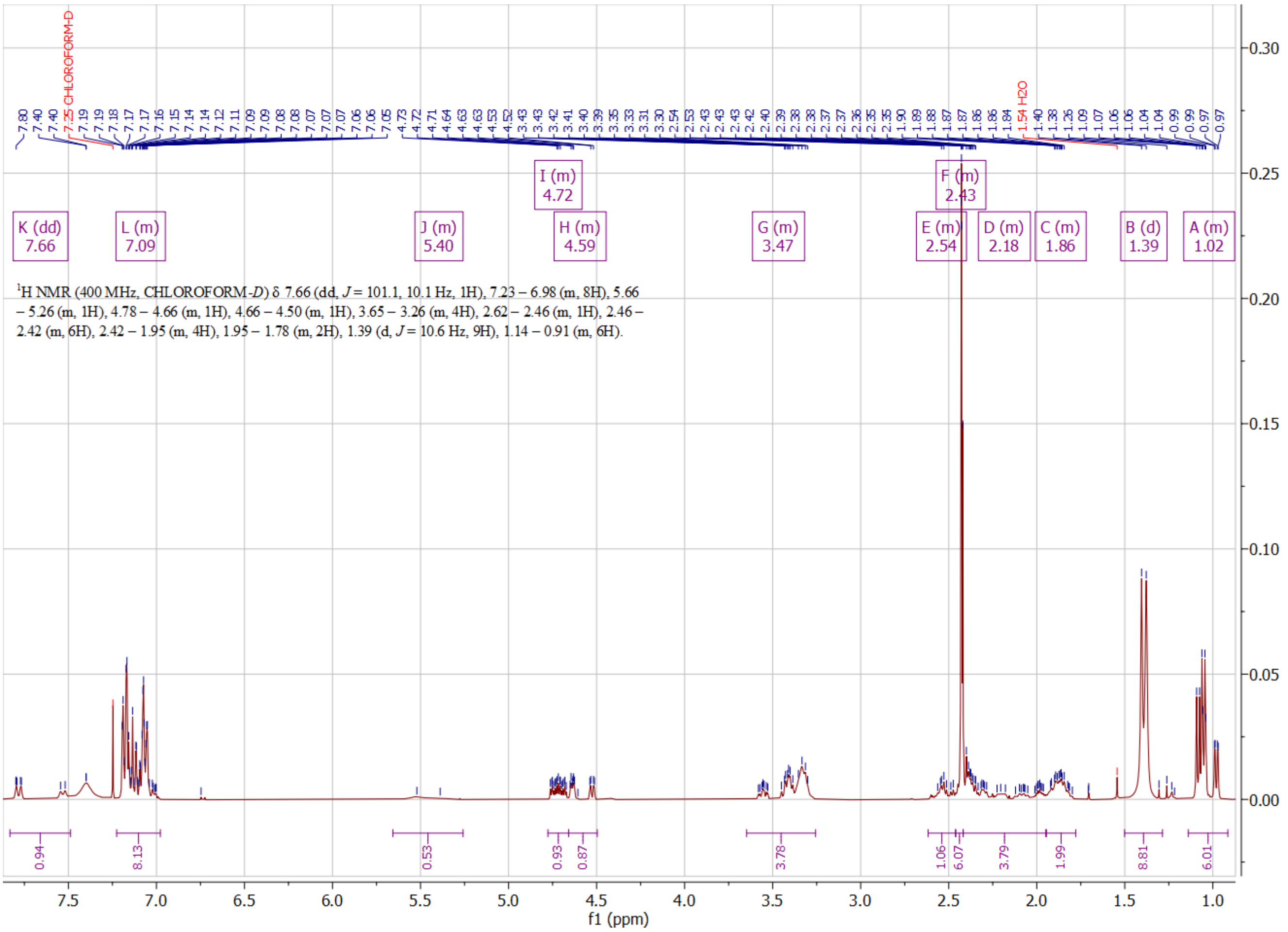
1H NMR (400 MHz, CHLOROFORM-D) δ 7.66 (dd, J = 101.1, 10.1 Hz, 1H), 7.23 – 6.98 (m, 8H), 5.66 – 5.26 (m, 1H), 4.78 – 4.66 (m, 1H), 4.66 – 4.50 (m, 1H), 3.65 – 3.26 (m, 4H), 2.62 – 2.46 (m, 1H), 2.46 – 2.42 (m, 6H), 2.42 – 1.95 (m, 4H), 1.95 – 1.78 (m, 2H), 1.39 (d, J = 10.6 Hz, 9H), 1.14 – 0.91 (m, 6H).
7.25 CHLOROFORM-D
1.54 H2O
K (dd) 7.66
L (m) 7.09
J (m) 5.40
I (m) 4.72
H (m) 4.59
G (m) 3.47
F (m) 2.43
E (m) 2.54
D (m) 2.18
C (m) 1.86
B (d) 1.39
A (m) 1.02
f1 (ppm)

(61) Boc-GABA-L-Pro-Val^P(OPh-4-SCH$_3$)$_2$

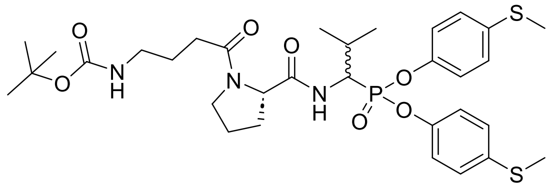

Name: *tert*-butyl (4-((2S)-2-((1-(bis(4-(methylthio)phenoxy)phosphoryl)-2-methylpropyl)carbamoyl)pyrrolidin-1-yl)-4-oxobutyl)carbamate

^{31}P NMR (162 MHz, Chloroform-d) δ 18.44 (s, 73%), 18.25 (s,27%).

^{1}H NMR (400 MHz, Chloroform-d) δ 1H NMR (400 MHz, CHLOROFORM-D) δ 7.67 (dd, J = 95.8, 10.3 Hz, 1H, NH), 7.24 – 6.97 (m, 8H, 8xArH), 4.91 (s, 1H, NH), 4.80 – 4.36 (m, 2H, CHP, CH), 3.72 – 3.07 (m, 4H, 2xCH$_2$), 2.43 (d, J = 2.1 Hz, 6H, 2xSCH$_3$), 2.41 – 2.33 (m, 2H, CH$_2$), 2.33 – 1.94 (m, 3H, 2xCH$_2$, CH), 1.94 – 1.61 (m, 4H, 2xCH$_2$), 1.48 – 1.22 (m, 9H, Boc), 1.13 – 0.96 (m, 6H, 2xCH$_3$).

HRMS: $C_{32}H_{46}N_3O_7PS_2$ [M+Na]$^+$ calc. 702.2412 found 702.2399

HPLC: purity >99%; retention time 17.360 and 17.567 min

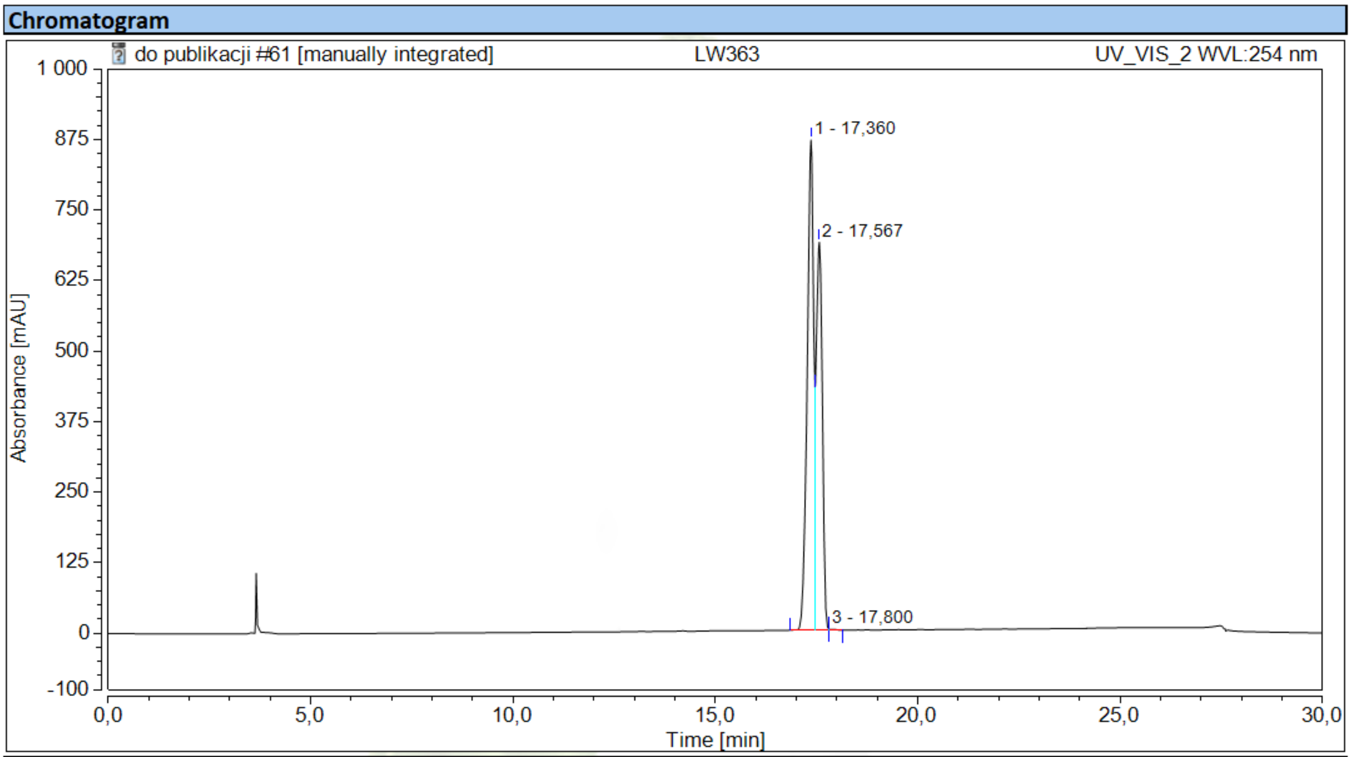

Integration Results

No.	Peak Name	Retention Time min	Area mAU*min	Height mAU	Relative Area %	Relative Height %	Amount n.a.
1		17,360	157,500	868,493	56,40	55,75	n.a.
2		17,567	121,485	687,522	43,50	44,13	n.a.
3		17,800	0,268	1,886	0,10	0,12	n.a.
**Total:**			**279,252**	**1557,901**	**100,00**	**100,00**	

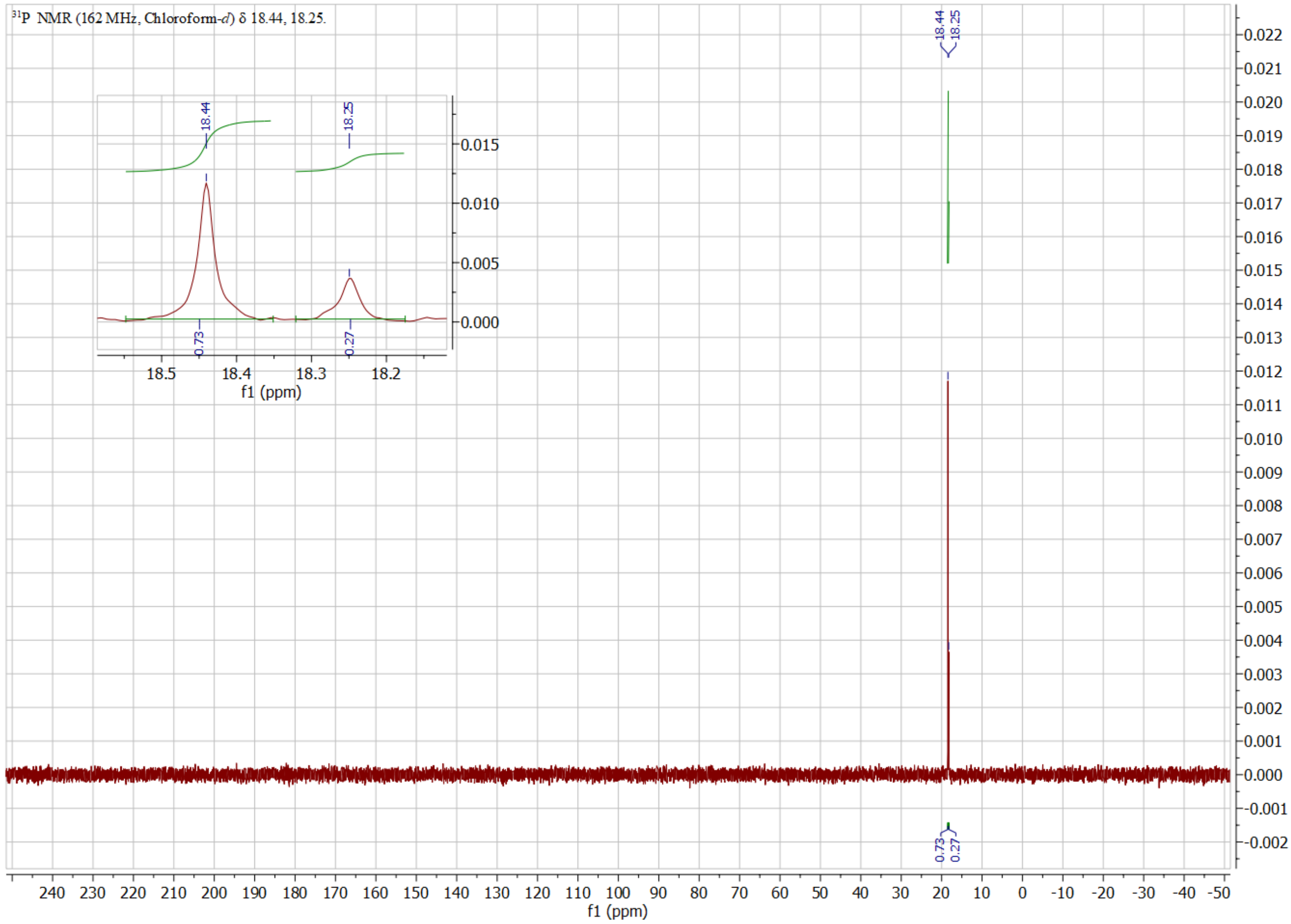
^{31}P NMR (162 MHz, Chloroform-*d*) δ 18.44, 18.25.
f1 (ppm)

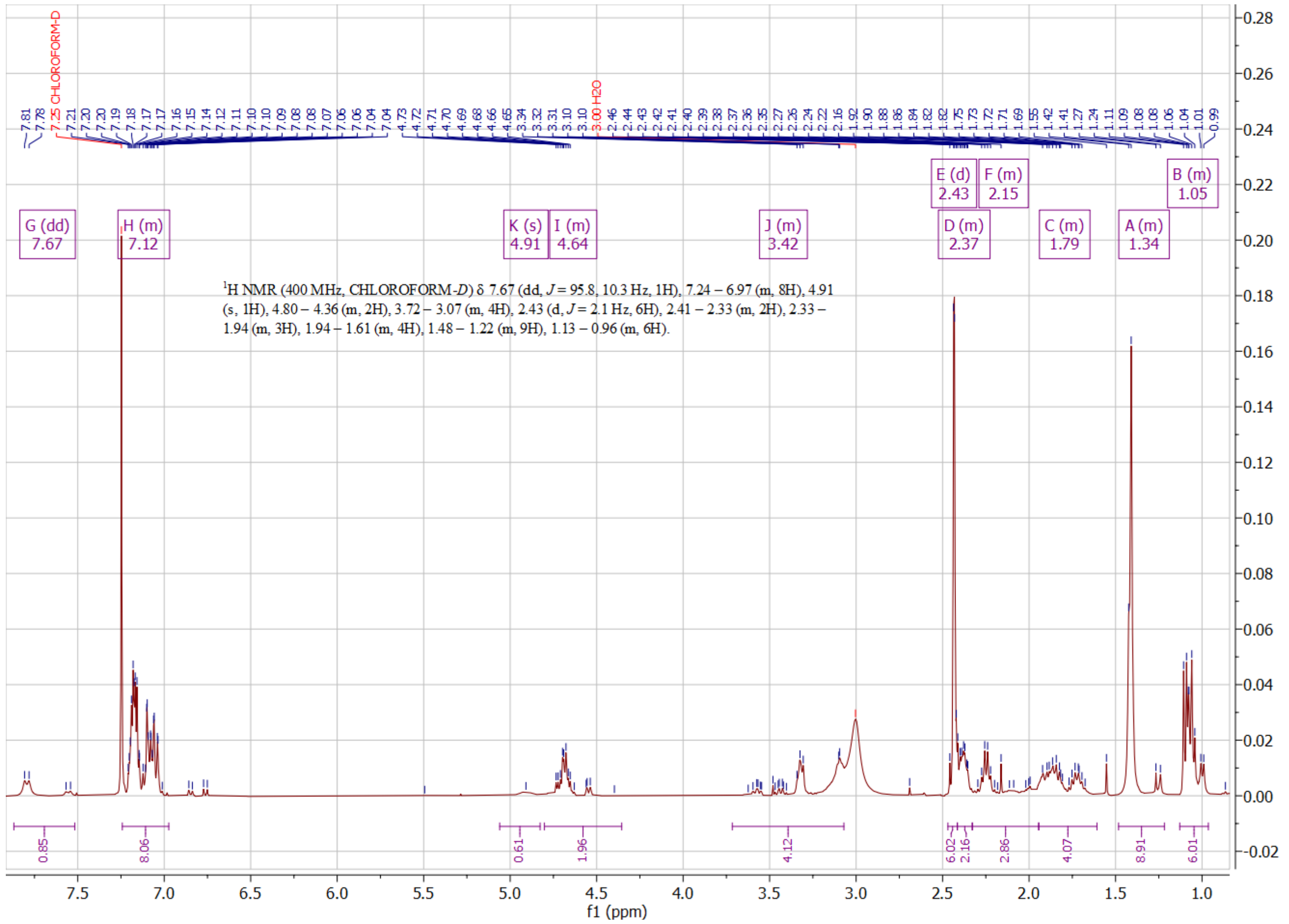
^{1}H NMR (400 MHz, CHLOROFORM-*D*) δ 7.67 (dd, *J* = 95.8, 10.3 Hz, 1H), 7.24 – 6.97 (m, 8H), 4.91 (s, 1H), 4.80 – 4.36 (m, 2H), 3.72 – 3.07 (m, 4H), 2.43 (d, *J* = 2.1 Hz, 6H), 2.41 – 2.33 (m, 2H), 2.33 – 1.94 (m, 3H), 1.94 – 1.61 (m, 4H), 1.48 – 1.22 (m, 9H), 1.13 – 0.96 (m, 6H).
f1 (ppm)

(62) Boc-L-Abu-L-Pro-Val^{P}(OPh-4-SCH$_3$)$_2$

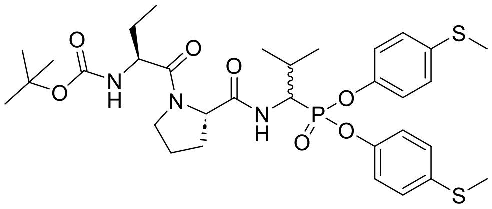

Name: *tert*-butyl ((2S)-1-((2S)-2-((1-(bis(4-(methylthio)phenoxy)phosphoryl)-2-methylpropyl)carbamoyl)pyrrolidin-1-yl)-1-oxobutan-2-yl)carbamate
^{31}P NMR (162 MHz, Chloroform-d) δ 18.49 (s, 59%), 18.14 (s, 41%).
^{1}H NMR (400 MHz, Chloroform-d) δ 1H NMR (400 MHz, CHLOROFORM-D) δ 7.41 (dd, J = 28.0, 10.5 Hz, 1H, NH), 7.21 – 6.70 (m, 8H, 8xArH), 5.22 (dd, J = 40.6, 8.8 Hz, 1H, NH), 4.77 – 3.96 (m, 3H, CHP, 2xCH), 3.85 – 3.32 (m, 2H, CH$_2$), 2.47 – 2.39 (m, 6H, 2xSCH$_3$), 2.39 – 2.27 (m, 1H, CH), 2.27 – 2.13 (m, 1H, 0.5xCH$_2$), 2.13 – 1.85 (m, 3H, 1.5xCH$_2$), 1.85 – 1.46 (m, 2H, CH$_2$), 1.46 – 1.22 (m, 9H, Boc), 1.11 – 0.80 (m, 9H, 3xCH$_3$).
HRMS: $C_{32}H_{46}N_3O_7PS_2$ [M+Na]$^+$ calc. 702.2412 found 702.2396
HPLC: purity >99%; retention time 18.143 and 18.427 min

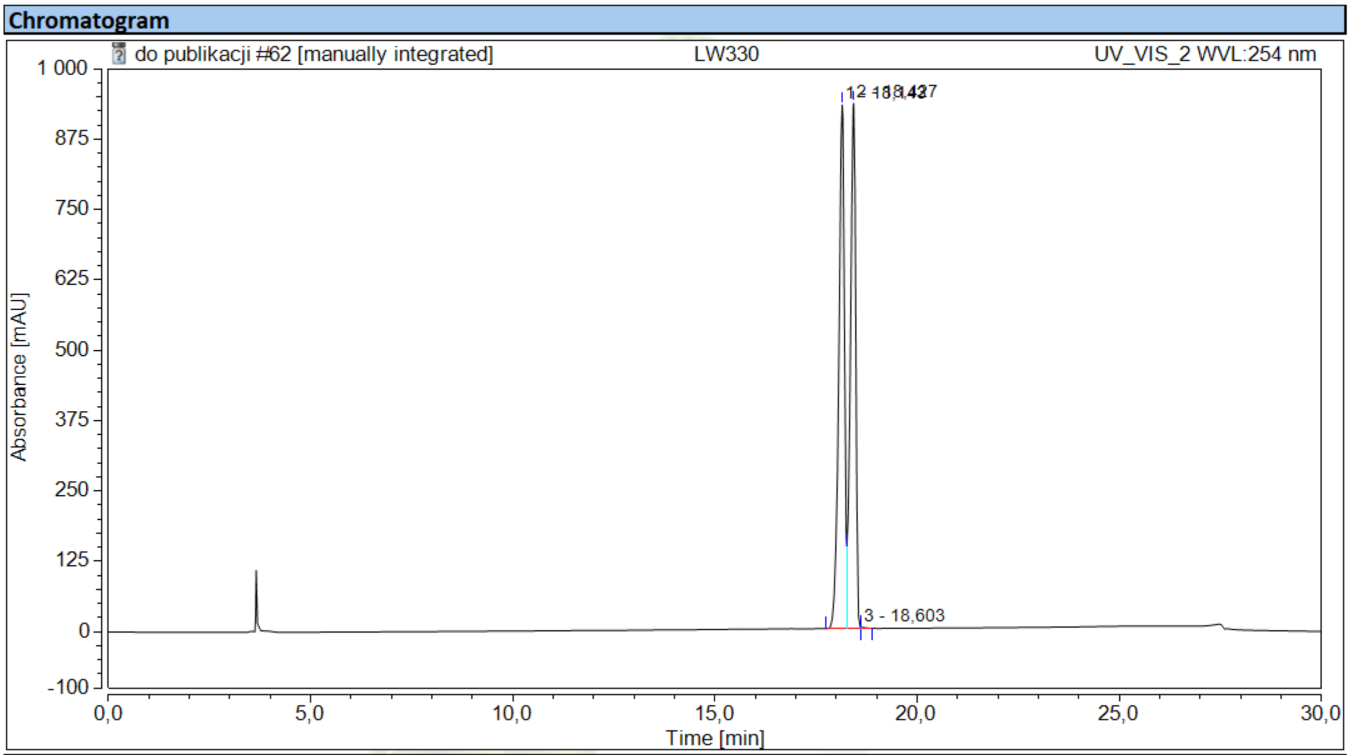

Integration Results

No.	Peak Name	Retention Time min	Area mAU*min	Height mAU	Relative Area %	Relative Height %	Amount n.a.
1		18,143	150,403	930,113	51,36	49,89	n.a.
2		18,427	142,241	932,688	48,57	50,02	n.a.
3		18,603	0,192	1,695	0,07	0,09	n.a.
**Total:**			**292,836**	**1864,496**	**100,00**	**100,00**	

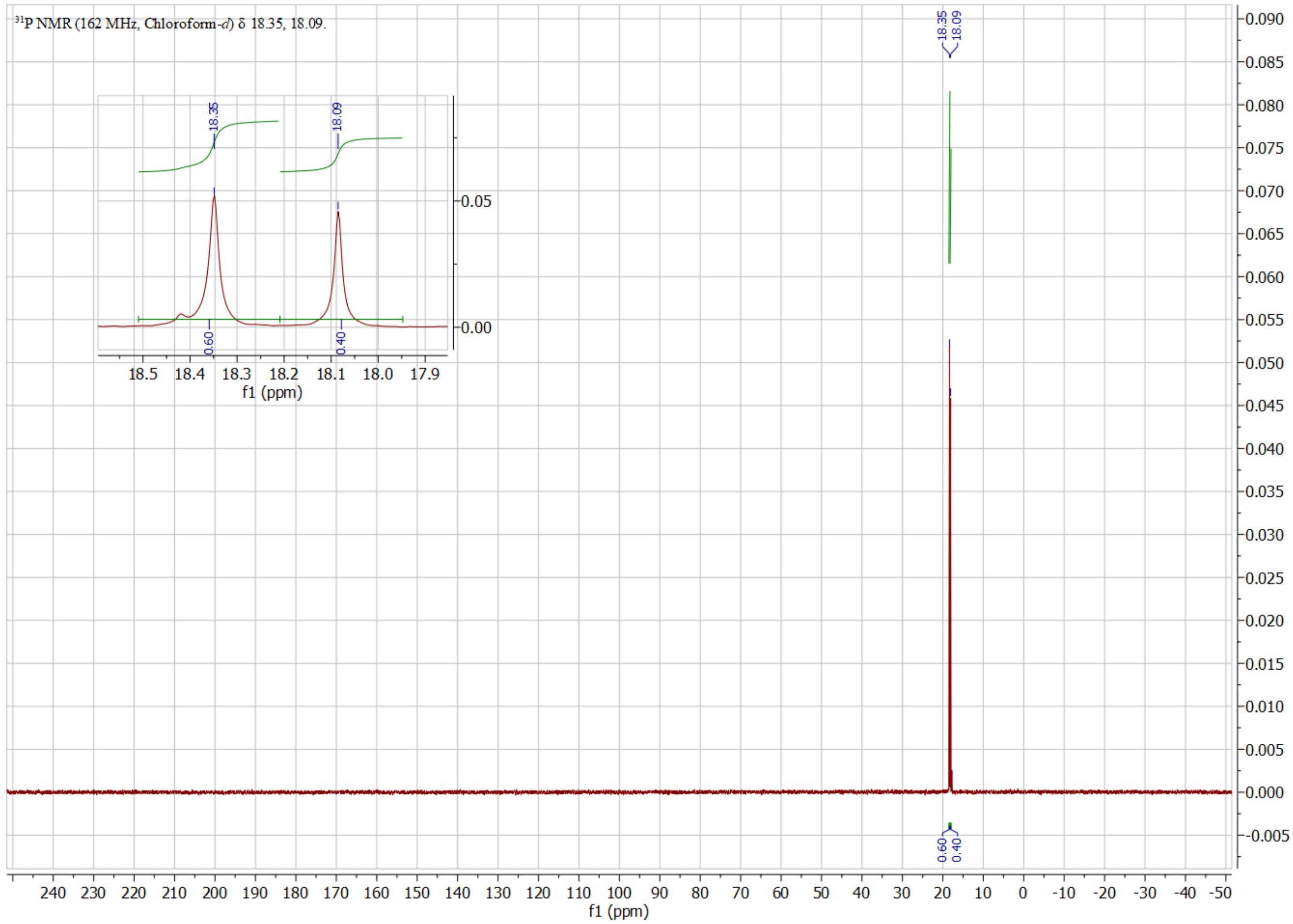
^{31}P NMR (162 MHz, Chloroform-*d*) δ 18.35, 18.09.
f1 (ppm)

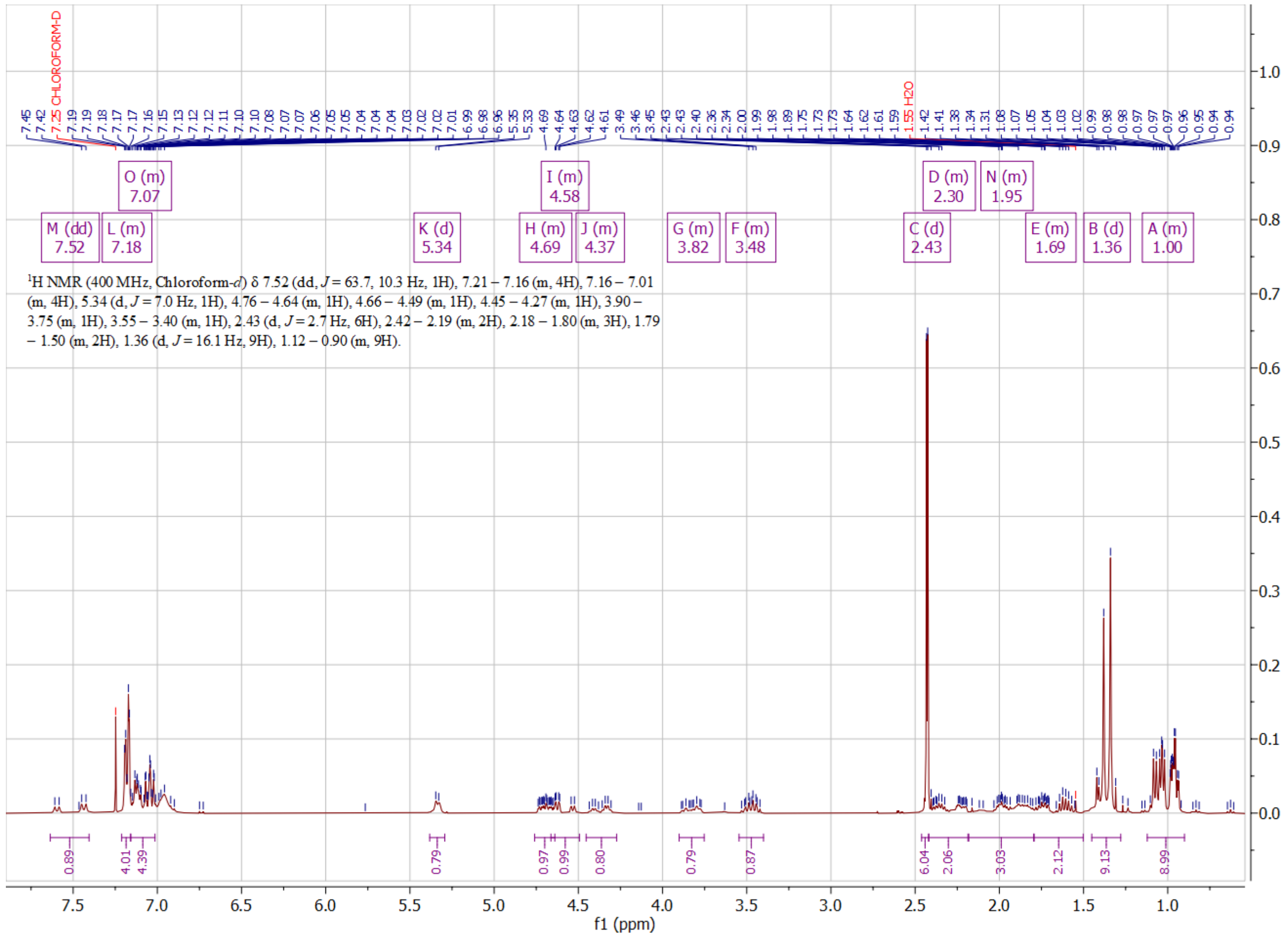
^{1}H NMR (400 MHz, Chloroform-*d*) δ 7.52 (dd, *J* = 63.7, 10.3 Hz, 1H), 7.21 – 7.16 (m, 4H), 7.16 – 7.01 (m, 4H), 5.34 (d, *J* = 7.0 Hz, 1H), 4.76 – 4.64 (m, 1H), 4.66 – 4.49 (m, 1H), 4.45 – 4.27 (m, 1H), 3.90 – 3.75 (m, 1H), 3.55 – 3.40 (m, 1H), 2.43 (d, *J* = 2.7 Hz, 6H), 2.42 – 2.19 (m, 2H), 2.18 – 1.80 (m, 3H), 1.79 – 1.50 (m, 2H), 1.36 (d, *J* = 16.1 Hz, 9H), 1.12 – 0.90 (m, 9H).
f1 (ppm)

(63) Boc-D-Abu-L-Pro-Val^{P}(OPh-4-SCH$_3$)$_2$

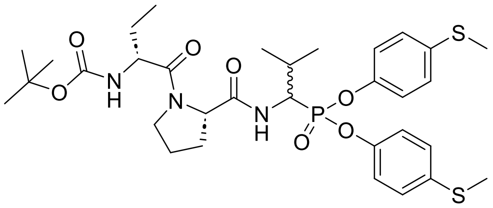

Name: *tert*-butyl ((2R)-1-((2S)-2-((1-(bis(4-(methylthio)phenoxy)phosphoryl)-2-methylpropyl)carbamoyl)pyrrolidin-1-yl)-1-oxobutan-2-yl)carbamate
^{31}P NMR (162 MHz, Chloroform-d) δ 18.35 (s, 60%), 18.09 (s, 40%).
^{1}H NMR (400 MHz, Chloroform-d) δ 7.52 (dd, J = 63.7, 10.3 Hz, 1H, NH), 7.21 – 7.16 (m, 4H, 4xArH), 7.16 – 7.01 (m, 4H, 4xArH), 5.34 (d, J = 7.0 Hz, 1H, NH), 4.76 – 4.64 (m, 1H, CHP), 4.66 – 4.49 (m, 1H, CH), 4.45 – 4.27 (m, 1H, CH), 3.90 – 3.75 (m, 1H, 0.5xCH$_2$), 3.55 – 3.40 (m, 1H, 0.5xCH$_2$), 2.43 (d, J = 2.7 Hz, 6H, 2xSCH$_3$), 2.42 – 2.19 (m, 2H, 0.5xCH$_2$, CH), 2.18 – 1.80 (m, 3H, 1.5xCH$_2$), 1.79 – 1.50 (m, 2H, CH$_2$), 1.36 (d, J = 16.1 Hz, 9H, Boc), 1.12 – 0.90 (m, 9H, 3xCH$_3$).
HRMS: $C_{32}H_{46}N_3O_7PS_2$ [M+Na]$^+$ calc. 702.2412 found 702.2405
HPLC: purity >99%; retention time 18.293 min

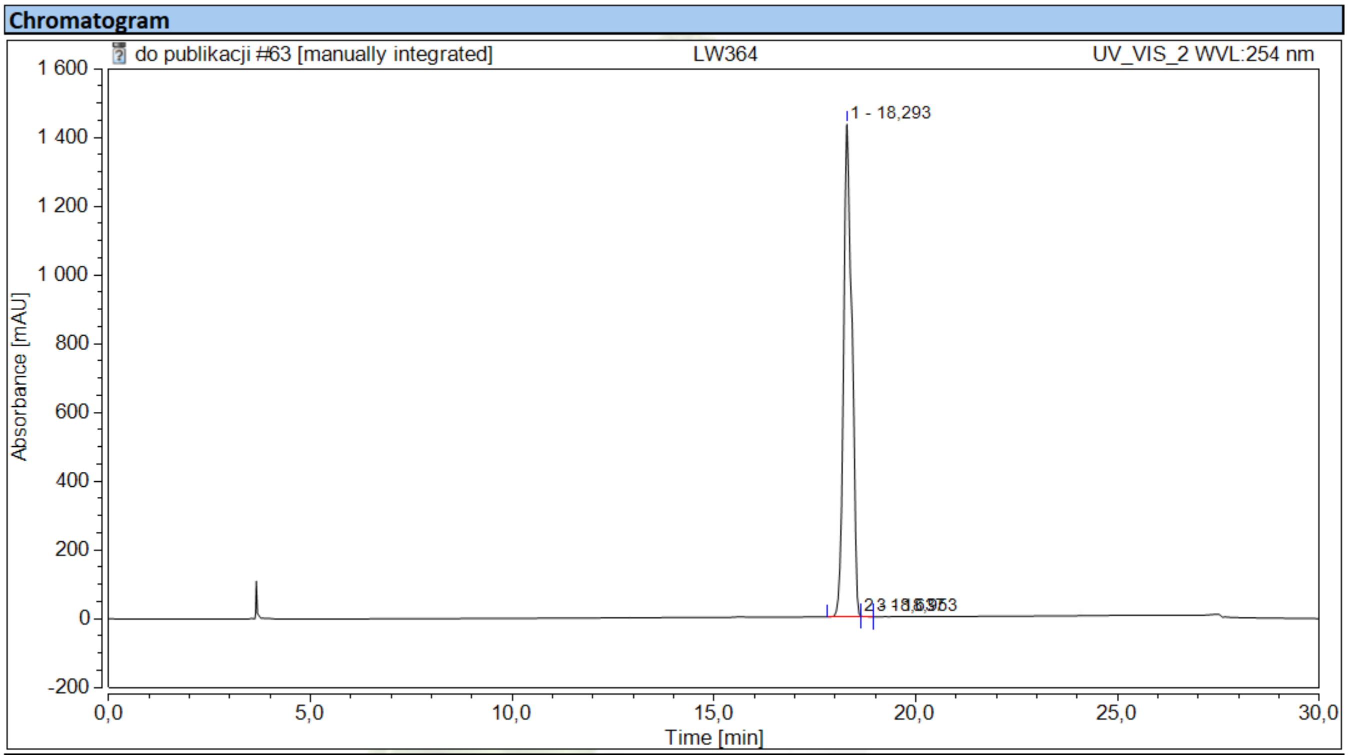

Integration Results

No.	Peak Name	Retention Time min	Area mAU*min	Height mAU	Relative Area %	Relative Height %	Amount n.a.
1		18,293	340,133	1432,720	99,95	99,88	n.a.
2		18,637	0,167	1,753	0,05	0,12	n.a.
3		18,953	0,000	0,000	0,00	0,00	n.a.
**Total:**			**340,300**	**1434,472**	**100,00**	**100,00**	

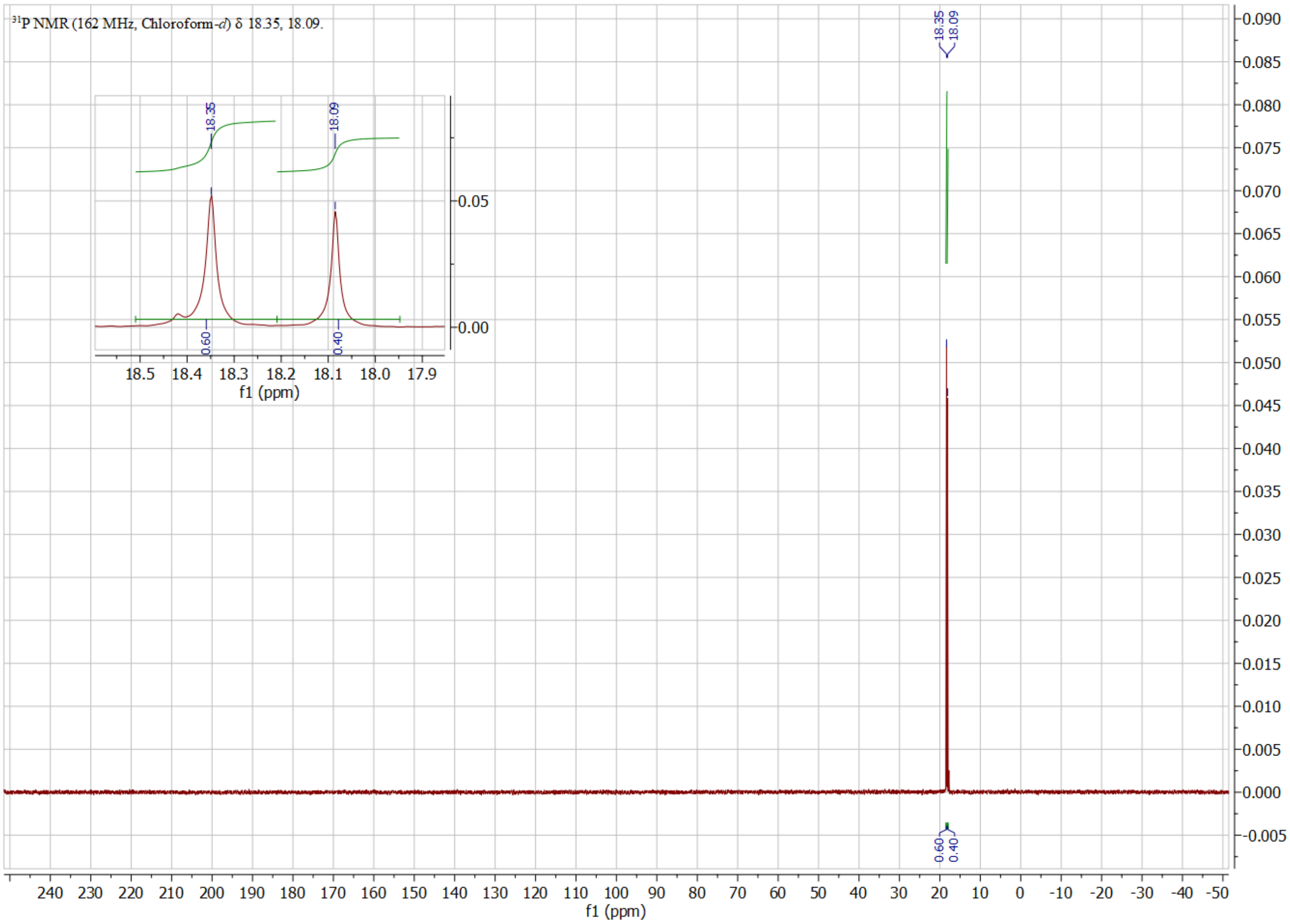
31P NMR (162 MHz, Chloroform-d) δ 18.35, 18.09.
f1 (ppm)

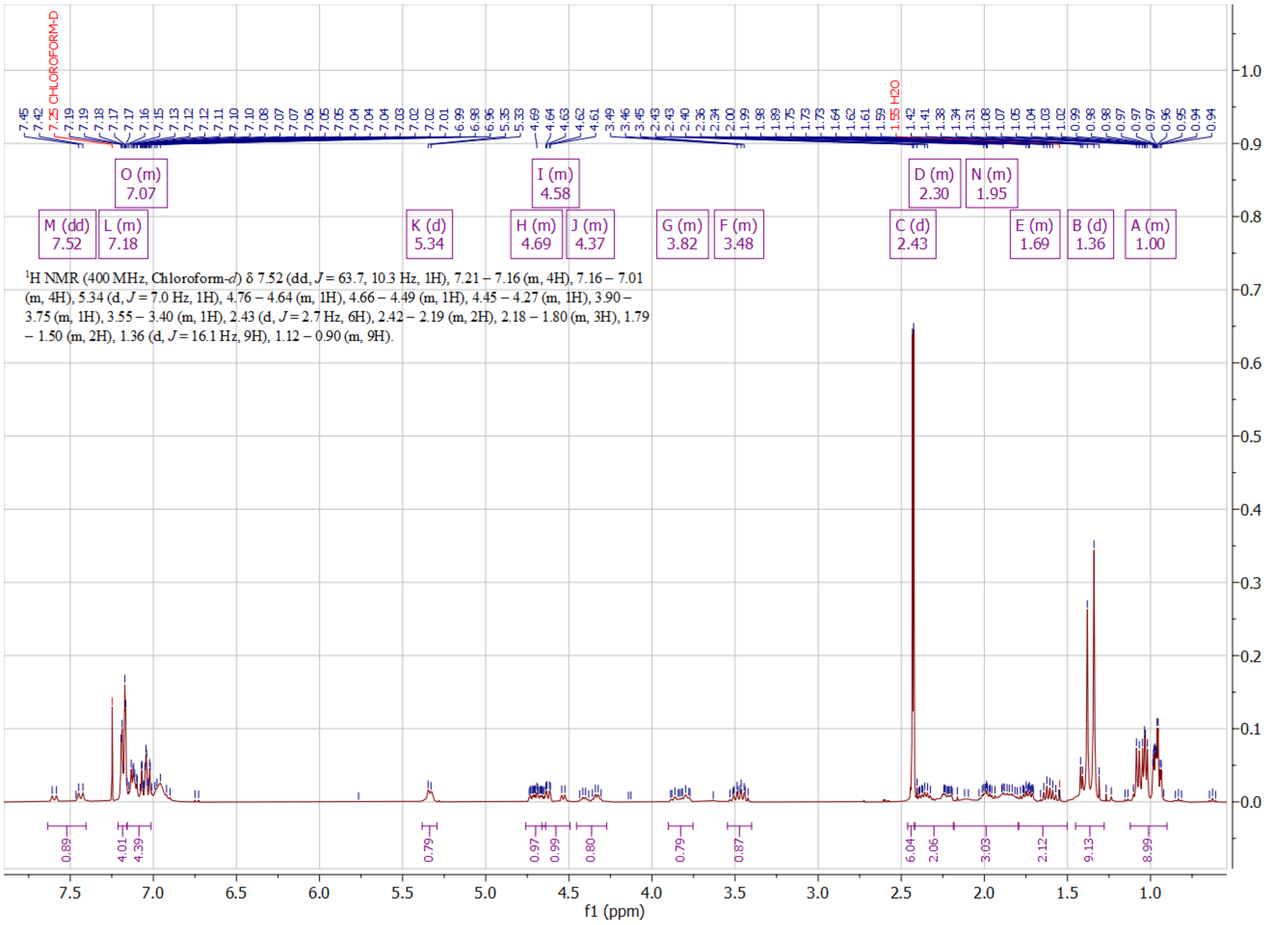
1H NMR (400 MHz, Chloroform-d) δ 7.52 (dd, J = 63.7, 10.3 Hz, 1H), 7.21 – 7.16 (m, 4H), 7.16 – 7.01 (m, 4H), 5.34 (d, J = 7.0 Hz, 1H), 4.76 – 4.64 (m, 1H), 4.66 – 4.49 (m, 1H), 4.45 – 4.27 (m, 1H), 3.90 – 3.75 (m, 1H), 3.55 – 3.40 (m, 1H), 2.43 (d, J = 2.7 Hz, 6H), 2.42 – 2.19 (m, 2H), 2.18 – 1.80 (m, 3H), 1.79 – 1.50 (m, 2H), 1.36 (d, J = 16.1 Hz, 9H), 1.12 – 0.90 (m, 9H).
f1 (ppm)

(64) Boc-AC4C-L-Pro-Val^P(OPh-4-SCH$_3$)$_2$

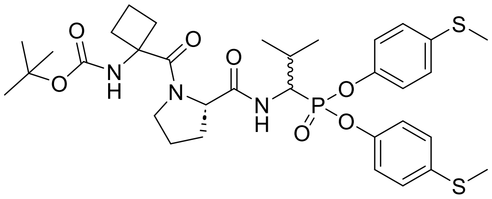

Name: *tert*-butyl (1-((2S)-2-((1-(bis(4-(methylthio)phenoxy)phosphoryl)-2-methylpropyl)carbamoyl)pyrrolidine-1-carbonyl)cyclobutyl)carbamate

^{31}P NMR (162 MHz, Chloroform-d) δ 18.78 (s, 75%), 18.06 (s, 25%).

^{1}H NMR (400 MHz, Chloroform-d) δ 7.72 (d, J = 7.8 Hz, 1H, NH), 7.21 – 6.98 (m, 8H, 8xArH), 5.27 – 5.15 (m, 1H, NH), 4.81 – 4.58 (m, 2H, CHP, CH), 3.62 – 3.31 (m, 2H, CH$_2$), 3.08 – 2.88 (m, 1H, 0.5xCH$_2$), 2.54 – 2.34 (m, 8H, 2xSCH$_3$, 0.5xCH$_2$, CH), 2.29 – 1.93 (m, 4H, 2xCH$_2$), 1.93 – 1.73 (m, 4H, 2xCH$_2$), 1.37 (d, J = 7.9 Hz, 9H, Boc), 1.22 – 0.99 (m, 6H, 2xCH$_3$).

HRMS: $C_{33}H_{46}N_3O_7PS_2$ [M+Na]$^+$ calc. 714,2412 found 714.2411

HPLC: purity >99%; retention time 18.170 and 18.337 min

Chromatogram

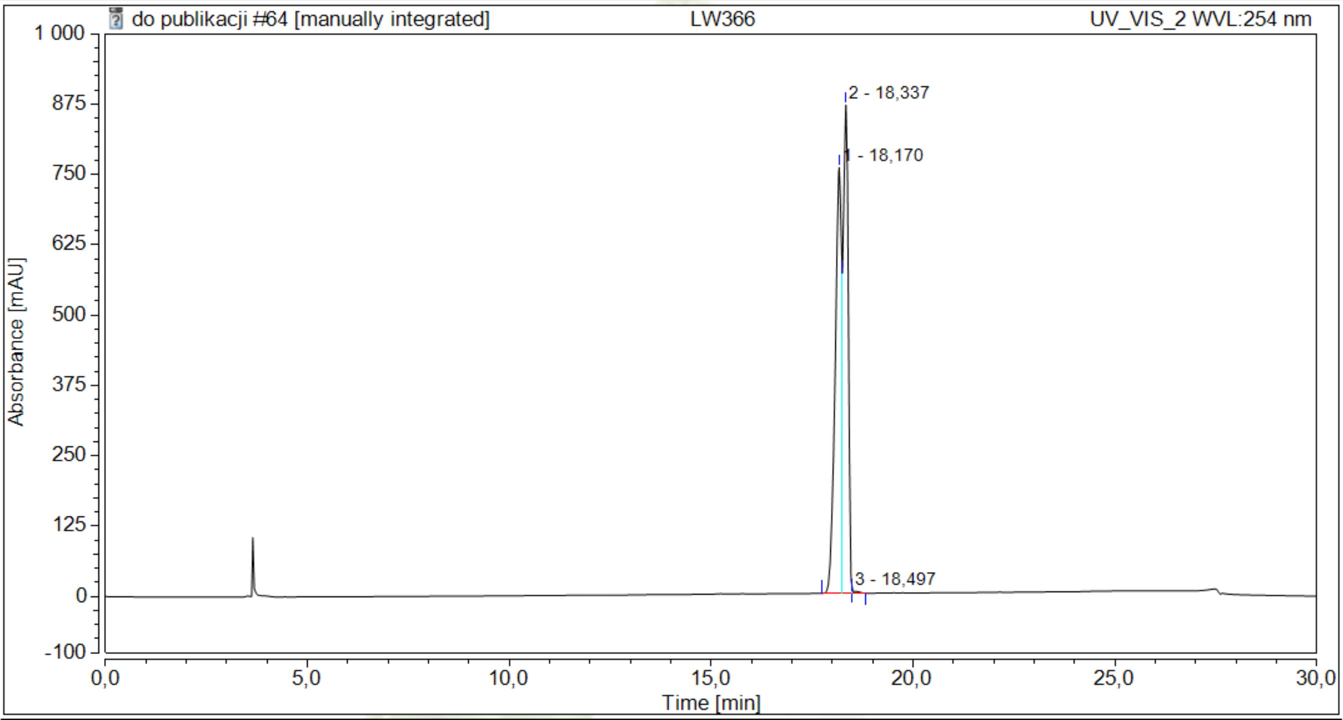

Integration Results

No.	Peak Name	Retention Time min	Area mAU*min	Height mAU	Relative Area %	Relative Height %	Amount n.a.
1		18,170	136,879	756,613	51,63	46,46	n.a.
2		18,337	127,577	867,601	48,12	53,27	n.a.
3		18,497	0,657	4,437	0,25	0,27	n.a.
**Total:**			**265,113**	**1628,651**	**100,00**	**100,00**	

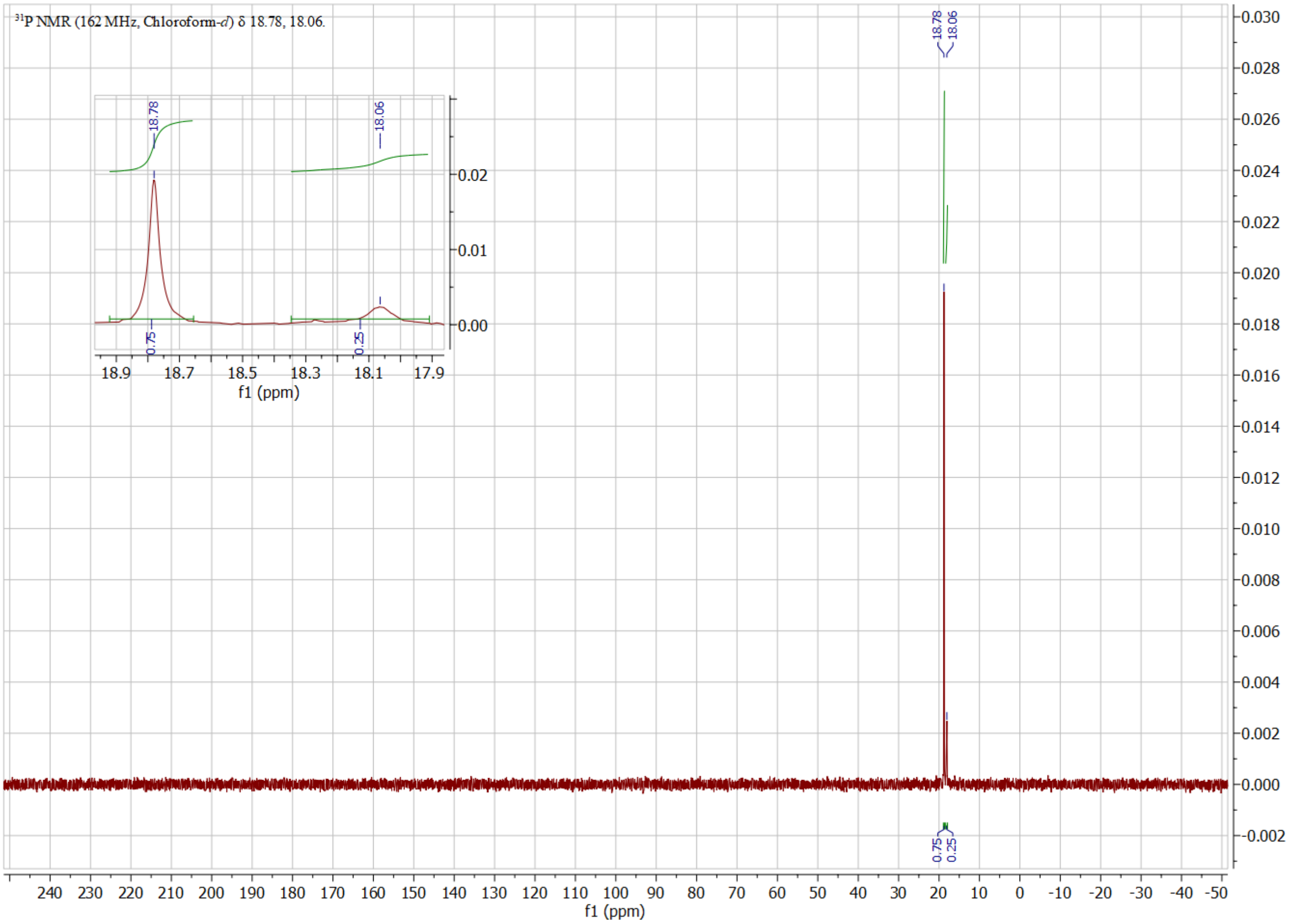

^{31}P NMR (162 MHz, Chloroform-*d*) δ 18.78, 18.06.
f1 (ppm)

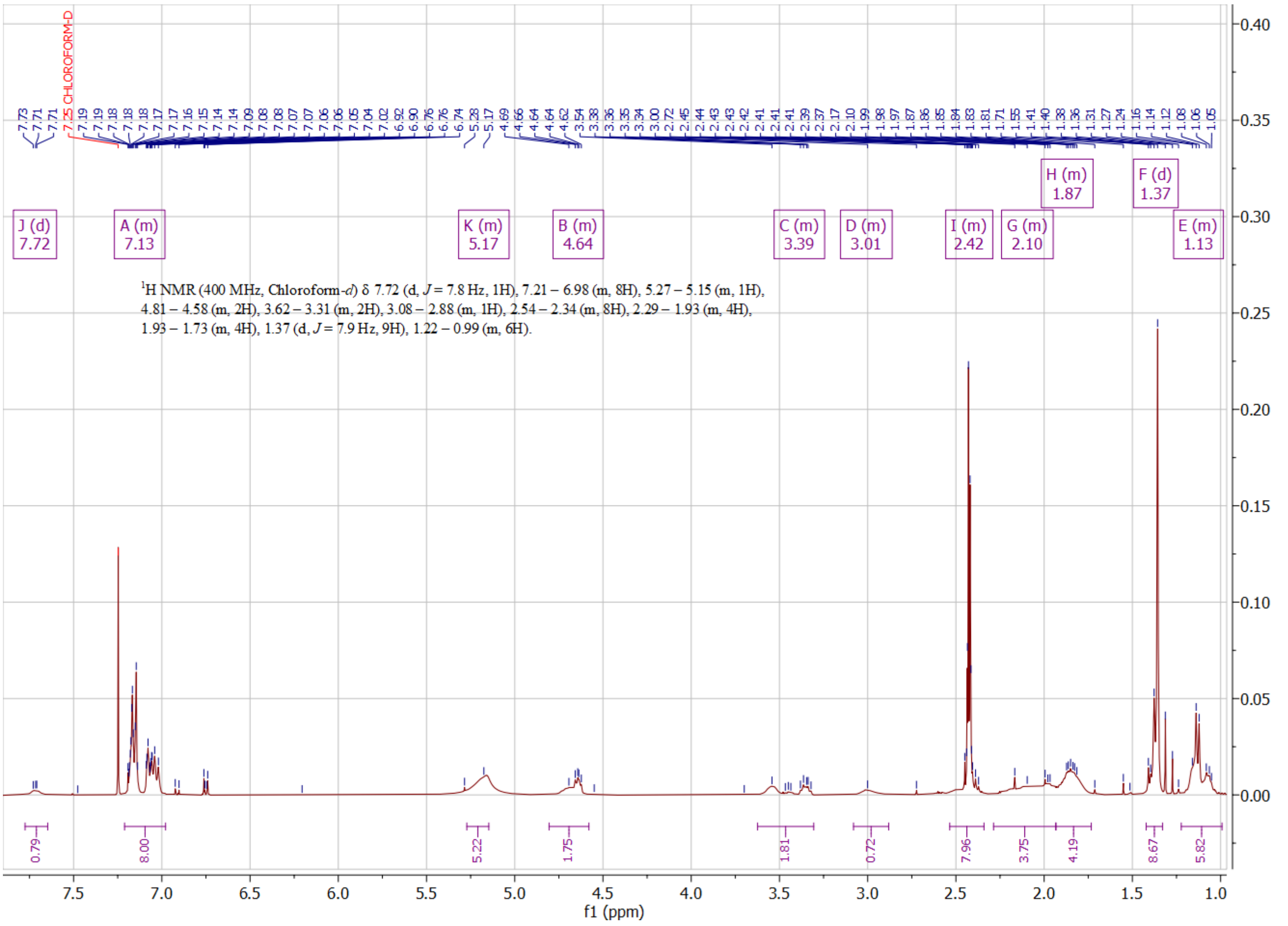

^{1}H NMR (400 MHz, Chloroform-*d*) δ 7.72 (d, *J* = 7.8 Hz, 1H), 7.21 – 6.98 (m, 8H), 5.27 – 5.15 (m, 1H), 4.81 – 4.58 (m, 2H), 3.62 – 3.31 (m, 2H), 3.08 – 2.88 (m, 1H), 2.54 – 2.34 (m, 8H), 2.29 – 1.93 (m, 4H), 1.93 – 1.73 (m, 4H), 1.37 (d, *J* = 7.9 Hz, 9H), 1.22 – 0.99 (m, 6H).
f1 (ppm)

(65) Boc-L-Nva-L-Pro- Val^{P}(OPh-4-SCH$_3$)$_2$

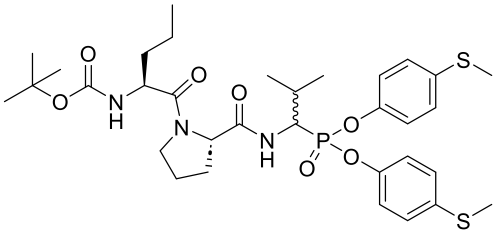

Name: *tert*-butyl ((2S)-1-((2S)-2-((1-(bis(4-(methylthio)phenoxy)phosphoryl)-2-methylpropyl)carbamoyl)pyrrolidin-1-yl)-1-oxopentan-2-yl)carbamate

^{31}P NMR (162 MHz, Chloroform-d) δ 18.37 (s, 53%), 17.97 (s, 47%).

^{1}H NMR (400 MHz, Chloroform-d) δ 7.39 (dd, J = 27.7, 10.4 Hz, 1H, NH), 7.23 – 7.14 (m, 4H, 4xArH), 7.13 – 7.01 (m, 4H, 4xArH), 5.29 (dd, J = 43.7, 8.8 Hz, 1H, NH), 4.77 – 4.48 (m, 2H, CHP, CH), 4.46 – 4.32 (m, 1H, CH), 3.77 – 3.66 (m, 1H, 0.5xCH$_2$), 3.61 – 3.43 (m, 1H, 0.5xCH$_2$), 2.43 (d, J = 1.6 Hz, 6H, 2xSCH$_3$), 2.41 – 2.27 (m, 2H, CH$_2$), 2.23 – 1.81 (m, 3H, CH$_2$, CH), 1.68 – 1.43 (m, 2H, CH$_2$), 1.41 (d, J = 2.2 Hz, 9H, Boc), 1.39 – 1.15 (m, 2H, CH$_2$), 1.09 – 0.93 (m, 6H, 2xCH$_3$), 0.82 (dt, J = 65.7, 7.2 Hz, 3H, CH$_3$).

HRMS: $C_{33}H_{48}N_3O_7PS_2$ [M+Na]$^+$ calc. 716.2569 found 716.2538

HPLC: purity >99%; retention time 18.870 and 19.060 min

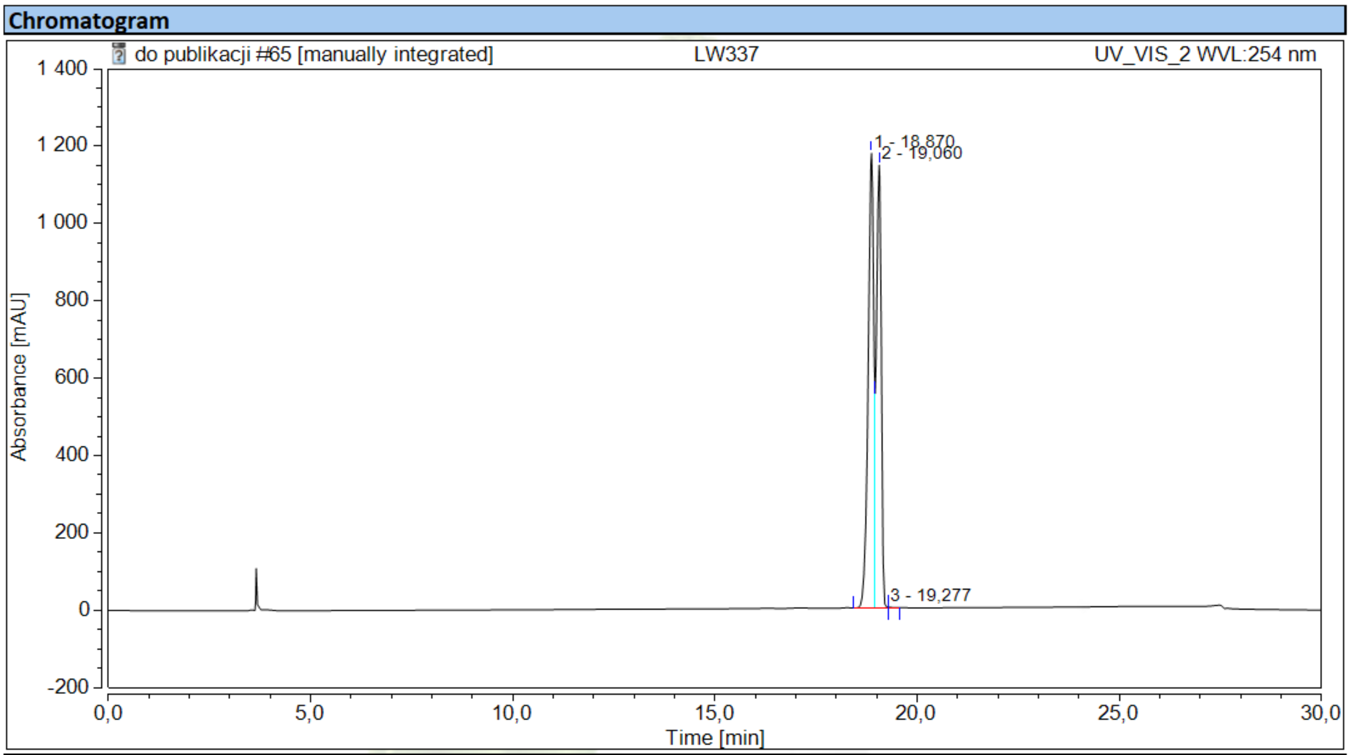

Integration Results

No.	Peak Name	Retention Time min	Area mAU*min	Height mAU	Relative Area %	Relative Height %	Amount n.a.
1		18,870	190,967	1175,642	54,73	50,62	n.a.
2		19,060	157,749	1144,759	45,21	49,29	n.a.
3		19,277	0,233	1,940	0,07	0,08	n.a.
**Total:**			**348,948**	**2322,342**	**100,00**	**100,00**	

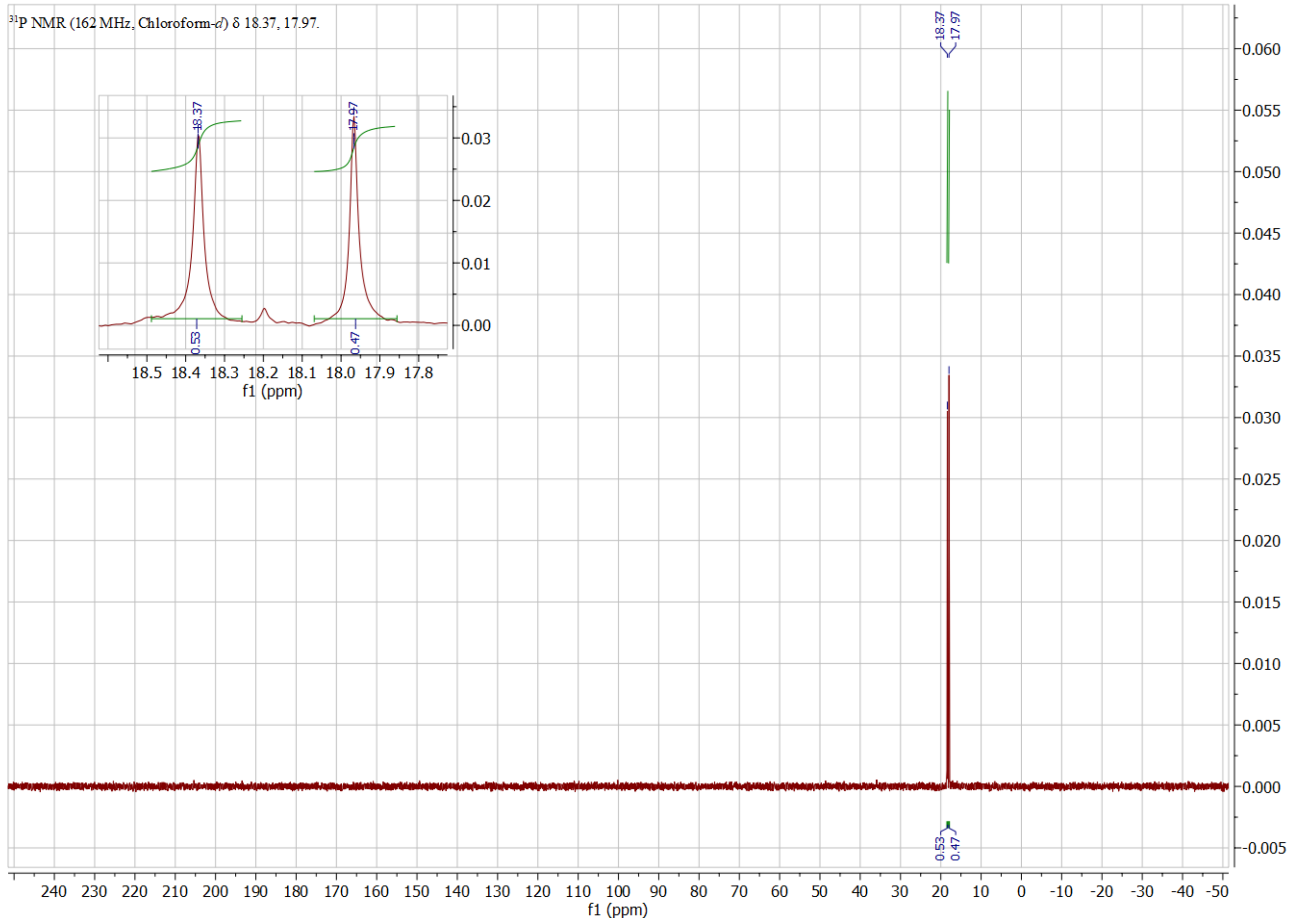
31P NMR (162 MHz, Chloroform-d) δ 18.37, 17.97.
f1 (ppm)

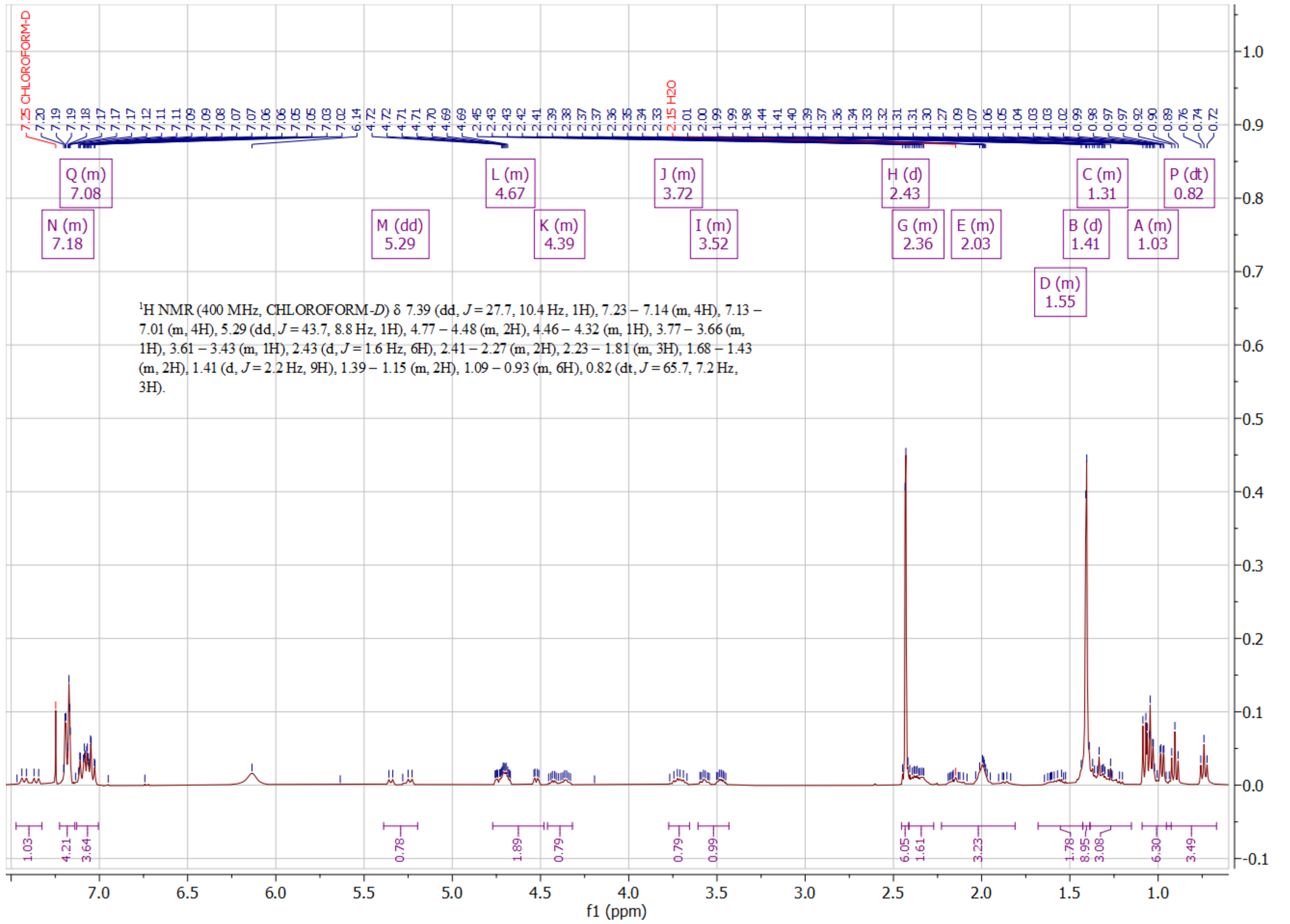
1H NMR (400 MHz, CHLOROFORM-D) δ 7.39 (dd, J = 27.7, 10.4 Hz, 1H), 7.23 – 7.14 (m, 4H), 7.13 – 7.01 (m, 4H), 5.29 (dd, J = 43.7, 8.8 Hz, 1H), 4.77 – 4.48 (m, 2H), 4.46 – 4.32 (m, 1H), 3.77 – 3.66 (m, 1H), 3.61 – 3.43 (m, 1H), 2.43 (d, J = 1.6 Hz, 6H), 2.41 – 2.27 (m, 2H), 2.23 – 1.81 (m, 3H), 1.68 – 1.43 (m, 2H), 1.41 (d, J = 2.2 Hz, 9H), 1.39 – 1.15 (m, 2H), 1.09 – 0.93 (m, 6H), 0.82 (dt, J = 65.7, 7.2 Hz, 3H).
f1 (ppm)

(66) Boc-D-Nva-L-Pro-Val^{P}(OPh-4-SCH$_3$)$_2$

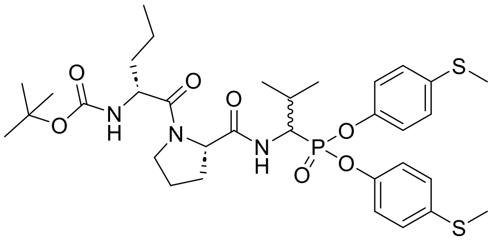

Name: *tert*-butyl ((2R)-1-((2S)-2-((1-(bis(4-(methylthio)phenoxy)phosphoryl)-2-methylpropyl)carbamoyl)pyrrolidin-1-yl)-1-oxopentan-2-yl)carbamate

^{31}P NMR (162 MHz, Chloroform-d) δ 18.46 (s, 50%), 18.17 (s, 50%).

^{1}H NMR (400 MHz, Chloroform-d) δ 7.54 (dd, J = 82.9, 10.3 Hz, 1H, NH), 7.22 – 7.10 (m, 6H, 6xArH), 7.09 – 7.00 (m, 2H, 2xArH), 5.27 (t, J = 7.3 Hz, 1H, NH), 4.73 – 4.64 (m, 1H, CHP), 4.64 – 4.51 (m, 1H, CH), 4.51 – 4.34 (m, 1H, CH), 3.89 – 3.74 (m, 1H, 0.5xCH$_2$), 3.53 – 3.39 (m, 1H, 0.5xCH$_2$), 2.43 (d, J = 4.0 Hz, 6H, 2xSCH$_3$), 2.41 – 2.20 (m, 2H, CH$_2$), 2.17 – 1.76 (m, 3H, CH$_2$, CH), 1.69 – 1.50 (m, 2H, CH$_2$), 1.49 – 1.30 (m, 2H, CH$_2$), 1.36 (d, J = 17.4 Hz, 9H, Boc), 1.12 – 0.96 (m, 6H, 2xCH$_3$), 0.96 – 0.90 (m, 3H, CH$_3$).

HRMS: $C_{33}H_{48}N_3O_7PS$ [M+Na]$^+$ calc. 716.2569 found 716.2576

HPLC: purity >99%; retention time 18.947 min

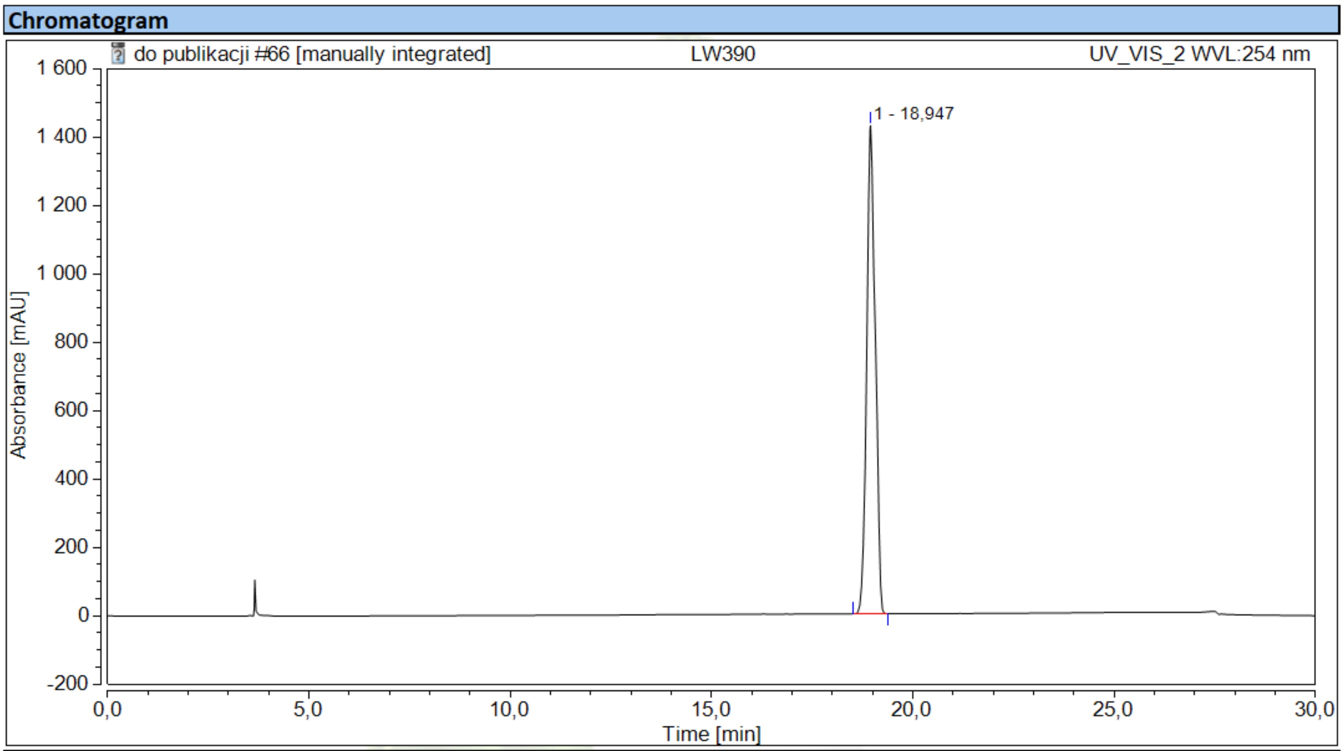

Integration Results

No.	Peak Name	Retention Time min	Area mAU*min	Height mAU	Relative Area %	Relative Height %	Amount n.a.
1		18,947	350,989	1427,156	100,00	100,00	n.a.
**Total:**			**350,989**	**1427,156**	**100,00**	**100,00**	

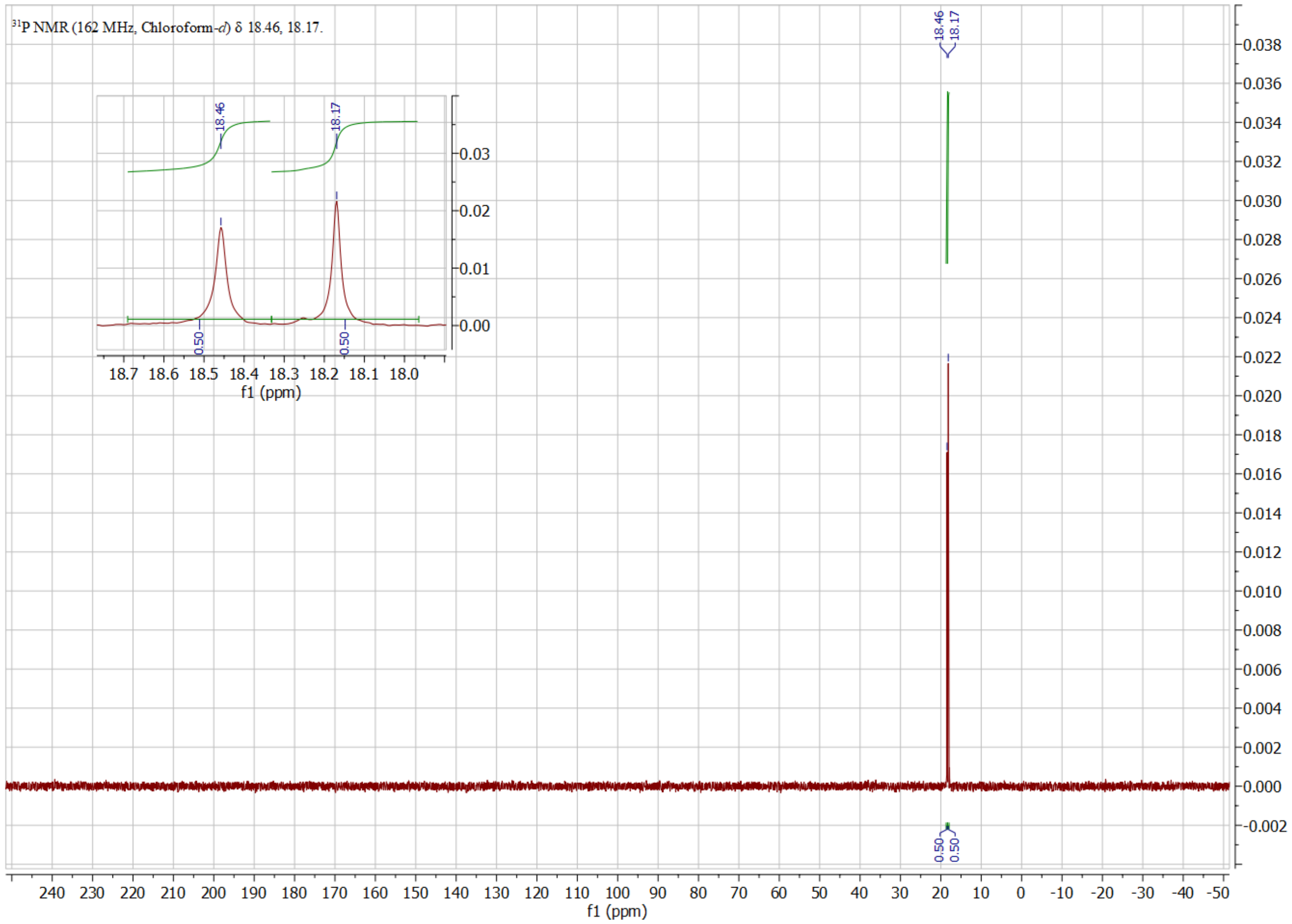

31P NMR (162 MHz, Chloroform-d) δ 18.46, 18.17.
18.46
18.17
0.50
0.50
f1 (ppm)

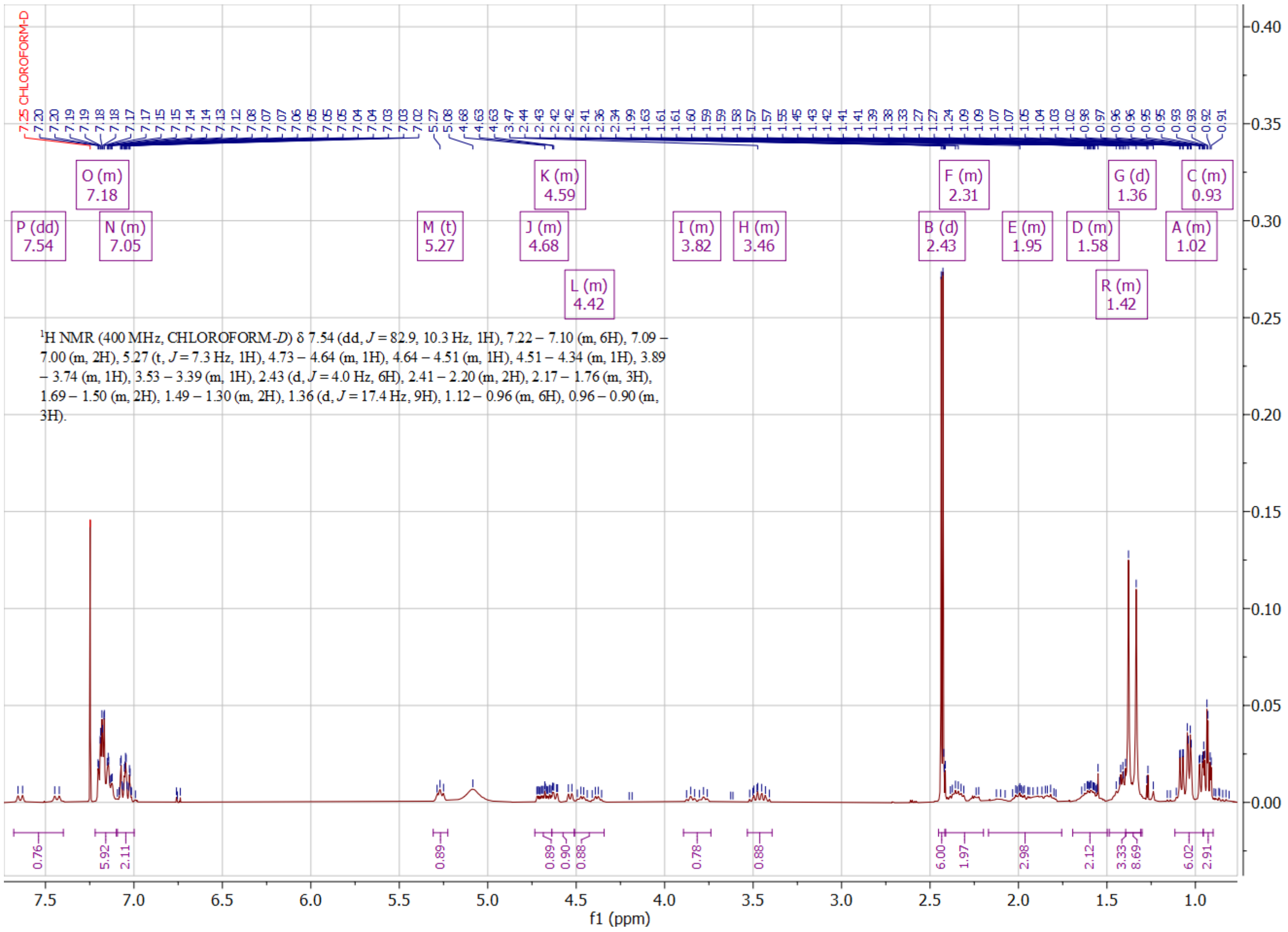

7.25 CHLOROFORM-D
P (dd) 7.54
O (m) 7.18
N (m) 7.05
M (t) 5.27
K (m) 4.59
J (m) 4.68
L (m) 4.42
I (m) 3.82
H (m) 3.46
F (m) 2.31
B (d) 2.43
E (m) 1.95
D (m) 1.58
R (m) 1.42
G (d) 1.36
C (m) 0.93
A (m) 1.02
1H NMR (400 MHz, CHLOROFORM-D) δ 7.54 (dd, J = 82.9, 10.3 Hz, 1H), 7.22 – 7.10 (m, 6H), 7.09 – 7.00 (m, 2H), 5.27 (t, J = 7.3 Hz, 1H), 4.73 – 4.64 (m, 1H), 4.64 – 4.51 (m, 1H), 4.51 – 4.34 (m, 1H), 3.89 – 3.74 (m, 1H), 3.53 – 3.39 (m, 1H), 2.43 (d, J = 4.0 Hz, 6H), 2.41 – 2.20 (m, 2H), 2.17 – 1.76 (m, 3H), 1.69 – 1.50 (m, 2H), 1.49 – 1.30 (m, 2H), 1.36 (d, J = 17.4 Hz, 9H), 1.12 – 0.96 (m, 6H), 0.96 – 0.90 (m, 3H).
f1 (ppm)

(67) Boc-D-Val-L-Pro-Val^P(OPh-4-SCH$_3$)$_2$

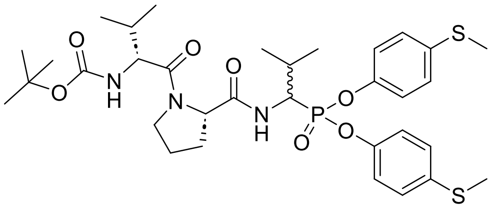

Name: *tert*-butyl ((2R)-1-((2S)-2-((1-(bis(4-(methylthio)phenoxy)phosphoryl)-2-methylpropyl)carbamoyl)pyrrolidin-1-yl)-3-methyl-1-oxobutan-2-yl)carbamate

^{31}P NMR (162 MHz, Chloroform-d) δ 18.35 (s, 54%), 18.15 (s, 46%).

^{1}H NMR (400 MHz, Chloroform-d) δ 7.56 (dd, J = 122.4, 10.3 Hz, 1H, NH), 7.22 – 7.09 (m, 6H, 6xArH), 7.09 – 6.95 (m, 2H, 2xArH), 5.27 (d, J = 9.6 Hz, 1H, NH), 4.74 – 4.63 (m, 1H, CHP), 4.58 (dd, J = 22.0, 8.0 Hz, 1H, CH), 4.31 – 4.18 (m, 1H, CH), 3.92 – 3.78 (m, 1H, 0.5xCH$_2$), 3.59 – 3.44 (m, 1H, 0.5xCH$_2$), 2.46 – 2.40 (m, 6H, 2xSCH$_3$), 2.40 – 2.28 (m, 1H, CH), 2.26 – 2.03 (m, 1H, CH), 2.03 – 1.74 (m, 4H, 2xCH$_2$), 1.35 (d, J = 23.8 Hz, 9H, Boc), 1.09 – 0.89 (m, 12H, 4xCH$_3$).

HRMS: $C_{33}H_{48}N_3O_7PS_2$ [M+Na]$^+$ calc. 716.2569 found 716.2557

HPLC: purity >99%; retention time 18.970 min

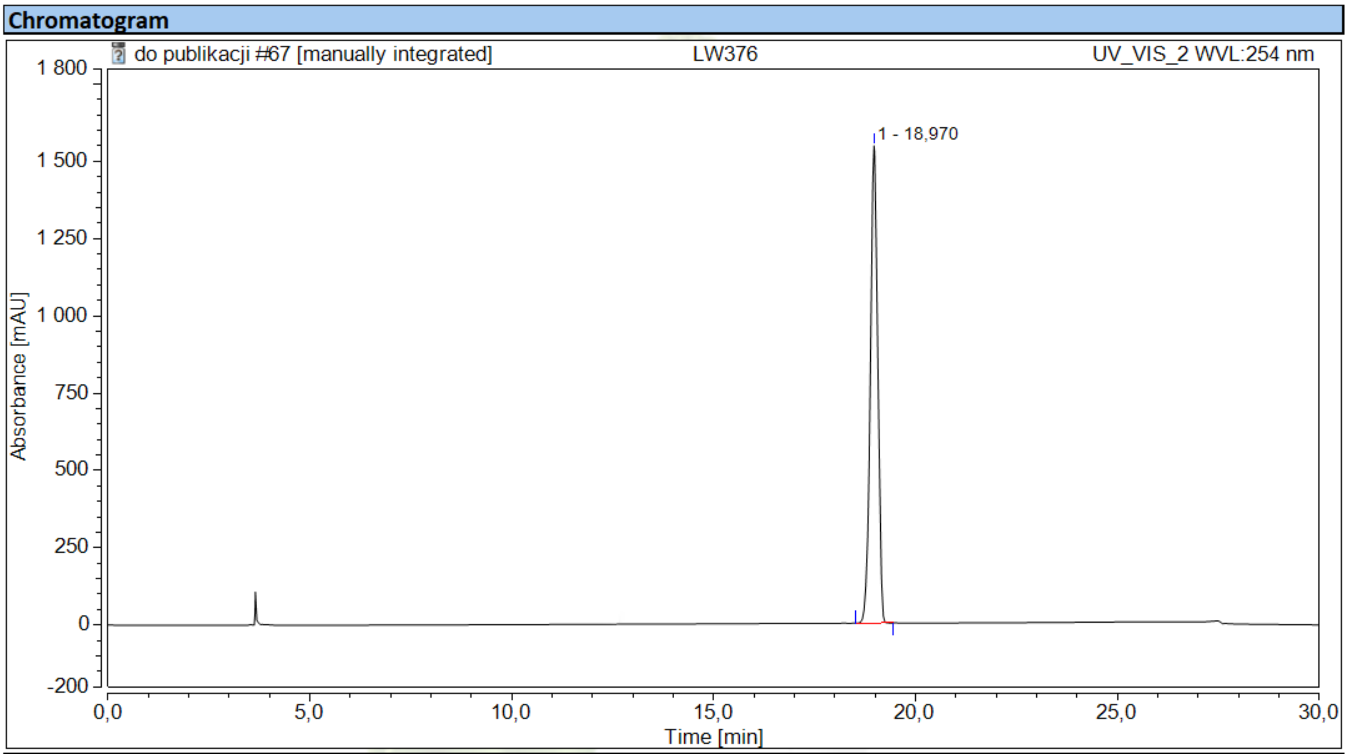

Integration Results

No.	Peak Name	Retention Time min	Area mAU*min	Height mAU	Relative Area %	Relative Height %	Amount n.a.
1		18,970	327,375	1544,305	100,00	100,00	n.a.
**Total:**			**327,375**	**1544,305**	**100,00**	**100,00**	

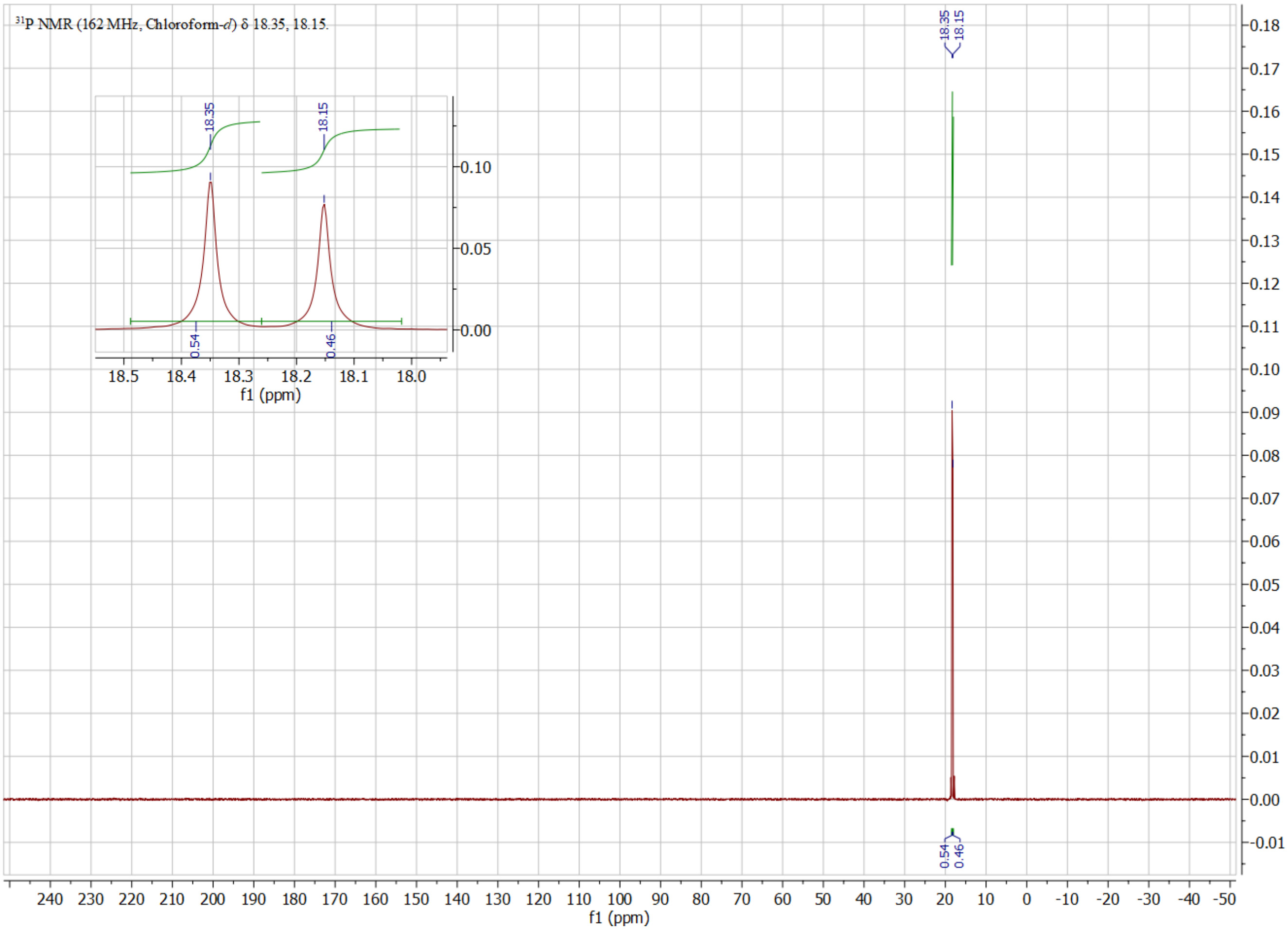
31P NMR (162 MHz, Chloroform-d) δ 18.35, 18.15.
f1 (ppm)

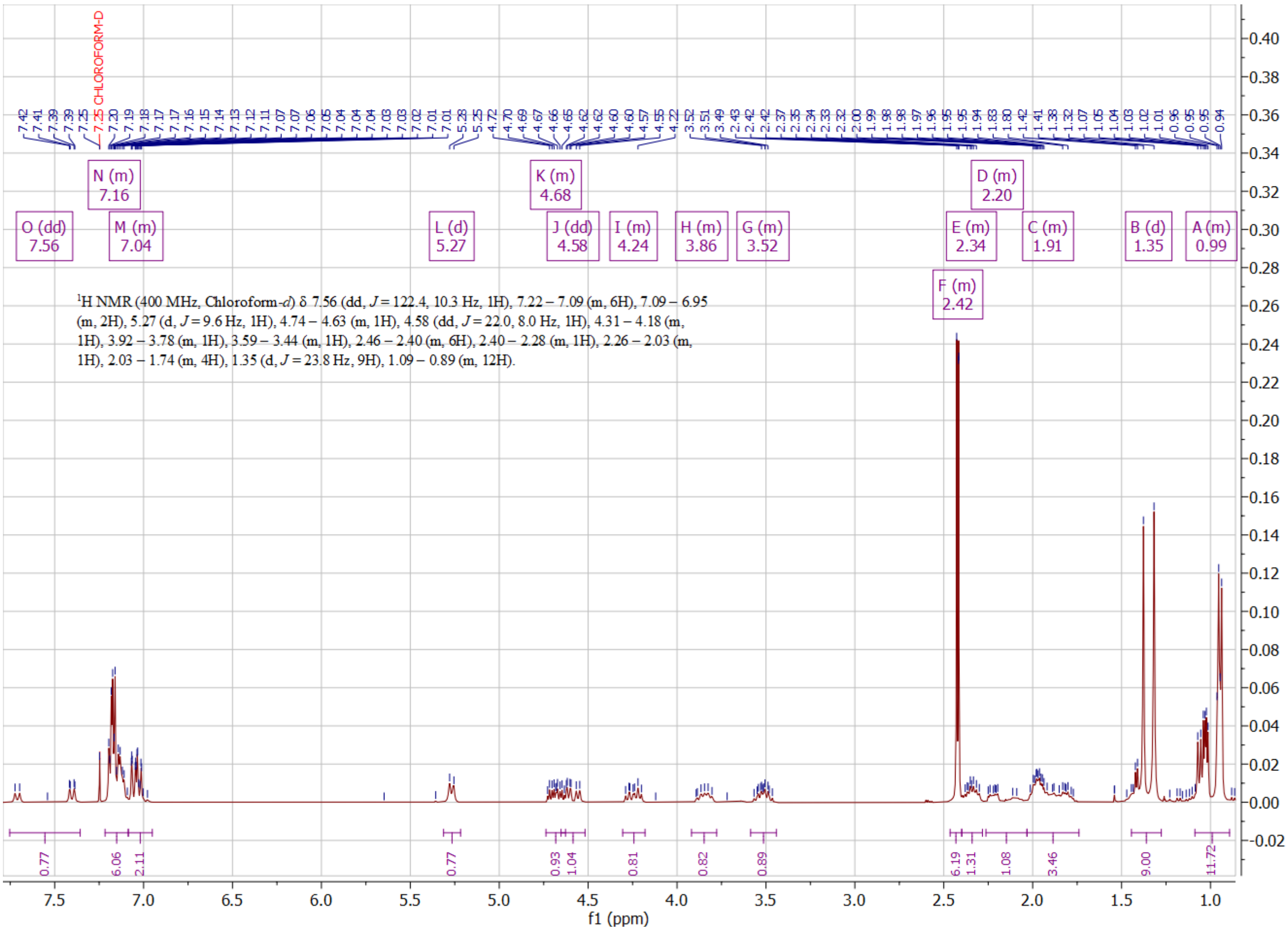
1H NMR (400 MHz, Chloroform-d) δ 7.56 (dd, J = 122.4, 10.3 Hz, 1H), 7.22 – 7.09 (m, 6H), 7.09 – 6.95 (m, 2H), 5.27 (d, J = 9.6 Hz, 1H), 4.74 – 4.63 (m, 1H), 4.58 (dd, J = 22.0, 8.0 Hz, 1H), 4.31 – 4.18 (m, 1H), 3.92 – 3.78 (m, 1H), 3.59 – 3.44 (m, 1H), 2.46 – 2.40 (m, 6H), 2.40 – 2.28 (m, 1H), 2.26 – 2.03 (m, 1H), 2.03 – 1.74 (m, 4H), 1.35 (d, J = 23.8 Hz, 9H), 1.09 – 0.89 (m, 12H).
7.25 CHLOROFORM-D
f1 (ppm)

(68) Boc-L-Tle-L-Pro-Val^P(OPh-4-SCH$_3$)$_2$

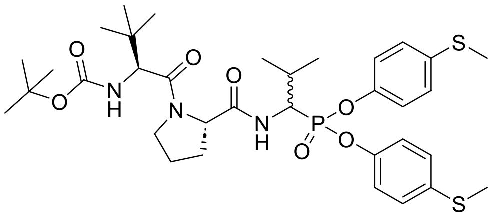

Name: *tert*-butyl ((2S)-1-((2S)-2-((1-(bis(4-(methylthio)phenoxy)phosphoryl)-2-methylpropyl)carbamoyl)pyrrolidin-1-yl)-3,3-dimethyl-1-oxobutan-2-yl)carbamate
^{31}P NMR (162 MHz, Chloroform-d) δ 18.70 (s, 35%), 18.16 (s, 65%).
^{1}H NMR (400 MHz, Chloroform-d) δ 7.28 (dd, J = 28.3, 9.8 Hz, 1H, NH), 7.20 – 7.13 (m, 4H, 4xArH), 7.13 – 7.02 (m, 4H, 4xArH), 5.30 (dd, J = 41.6, 9.9 Hz, 1H, NH), 4.76 – 4.65 (m, 1H, CHP), 4.47 (dd, J = 8.1, 3.4 Hz, 1H, CH), 4.35 – 4.25 (m, 1H, CH), 3.90 – 3.54 (m, 2H, CH$_2$), 2.46 – 2.41 (m, 6H, 2xSCH$_3$), 2.40 – 2.29 (m, 1H, CH), 2.22 – 1.77 (m, 4H, 2xCH$_2$), 1.41 (d, J = 2.6 Hz, 9H, Boc), 1.13 – 0.91 (m, 15H, 5xCH$_3$).
HRMS: $C_{34}H_{50}N_3O_7PS_2$ [M+Na]$^+$ calc. 730.2725 found 730.2714
HPLC: purity >99%; retention time 19.713 min

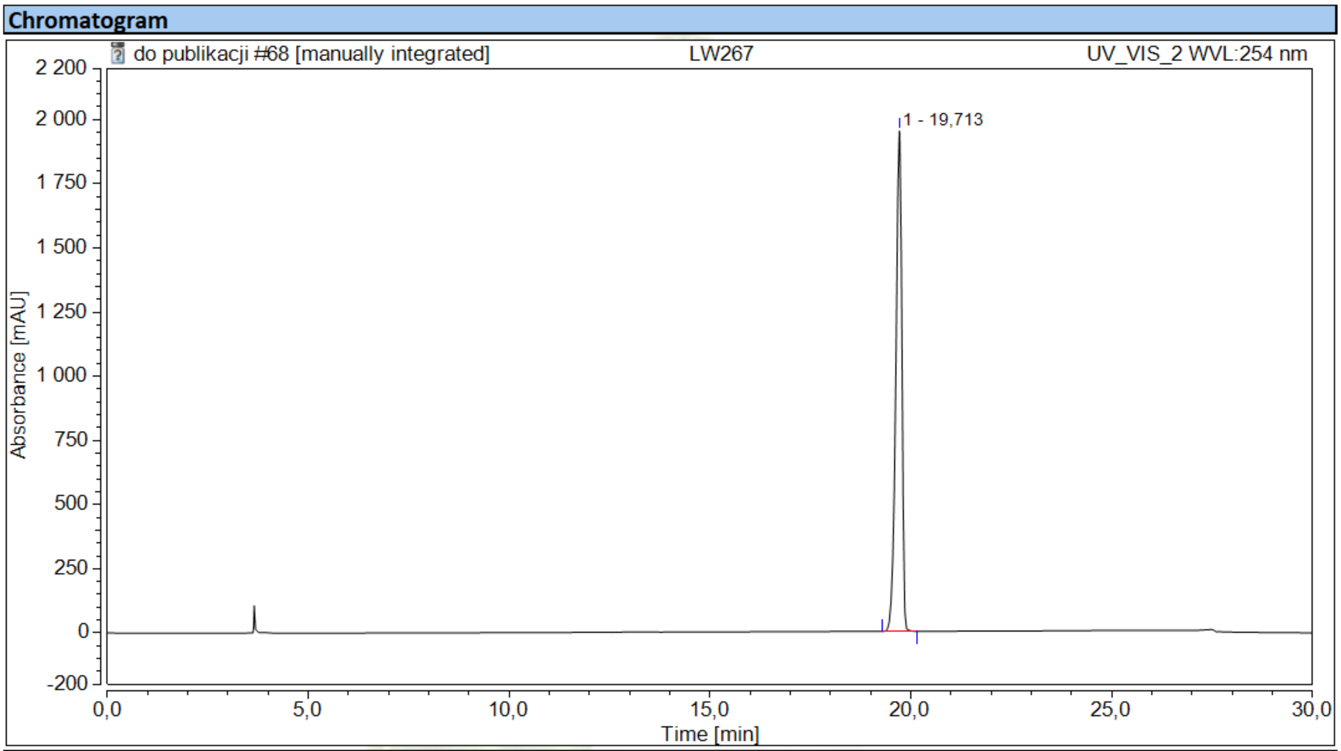

Integration Results

No.	Peak Name	Retention Time min	Area mAU*min	Height mAU	Relative Area %	Relative Height %	Amount n.a.
1		19,713	339,869	1949,761	100,00	100,00	n.a.
**Total:**			**339,869**	**1949,761**	**100,00**	**100,00**	

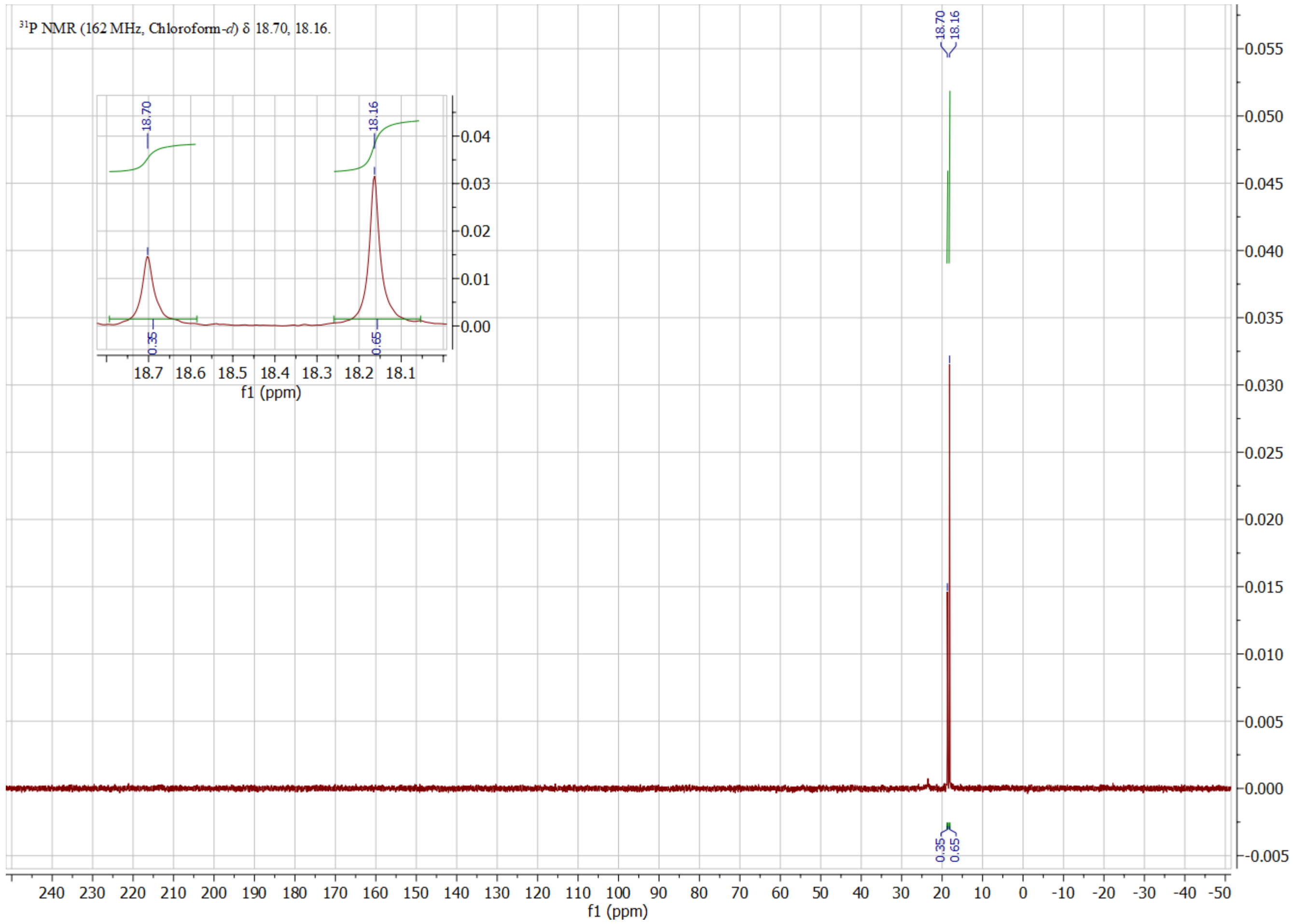

^{31}P NMR (162 MHz, Chloroform-*d*) δ 18.70, 18.16.
f1 (ppm)

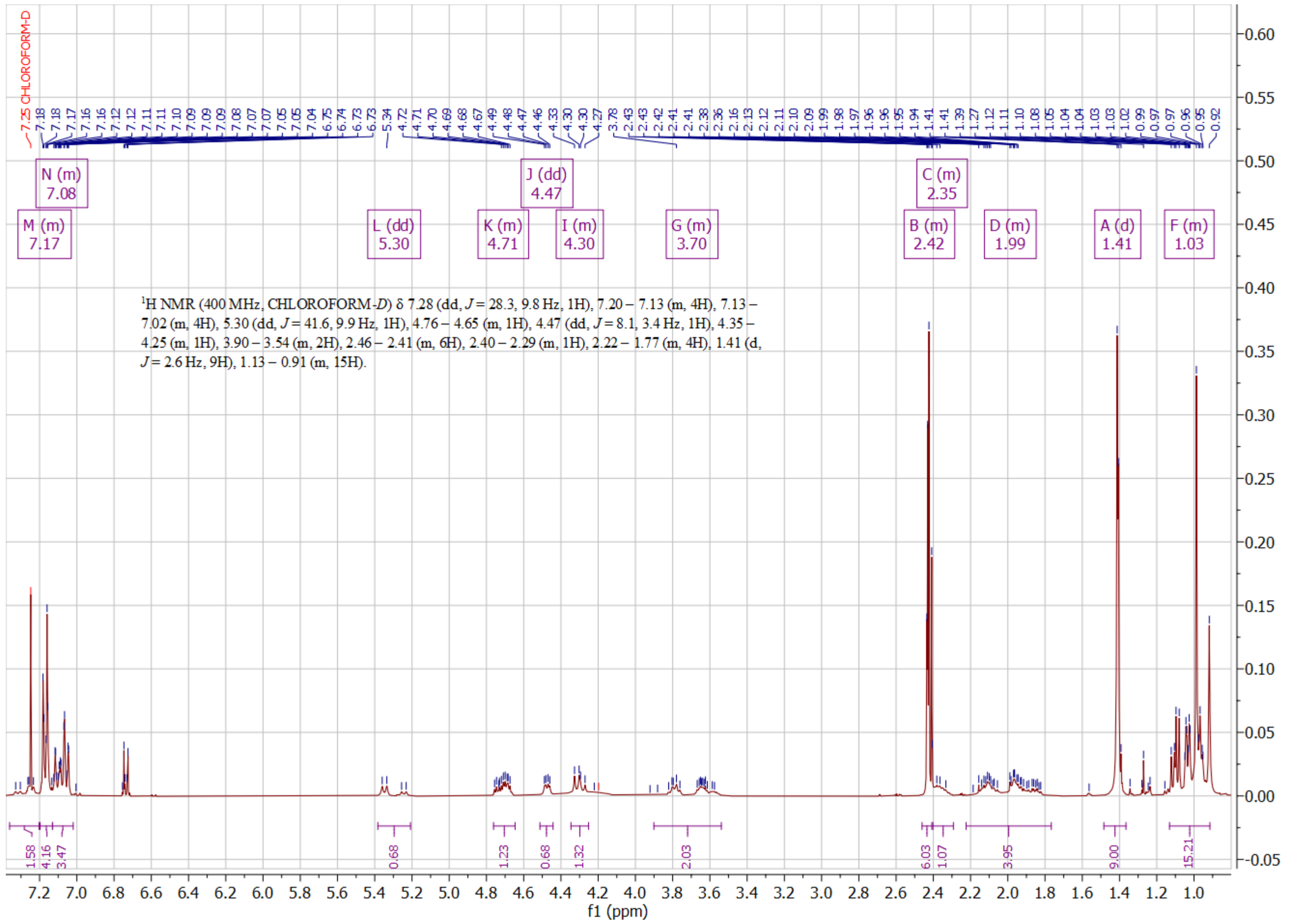

^{1}H NMR (400 MHz, CHLOROFORM-*D*) δ 7.28 (dd, *J* = 28.3, 9.8 Hz, 1H), 7.20 – 7.13 (m, 4H), 7.13 – 7.02 (m, 4H), 5.30 (dd, *J* = 41.6, 9.9 Hz, 1H), 4.76 – 4.65 (m, 1H), 4.47 (dd, *J* = 8.1, 3.4 Hz, 1H), 4.35 – 4.25 (m, 1H), 3.90 – 3.54 (m, 2H), 2.46 – 2.41 (m, 6H), 2.40 – 2.29 (m, 1H), 2.22 – 1.77 (m, 4H), 1.41 (d, *J* = 2.6 Hz, 9H), 1.13 – 0.91 (m, 15H).
7.25 CHLOROFORM-D
f1 (ppm)

(69) Boc-D-Tle-L-Pro- Val^{P}(OPh-4-SCH$_3$)$_2$

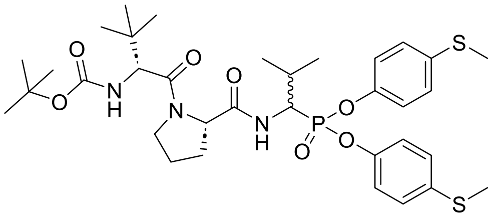

Name: *tert*-butyl ((2R)-1-((2S)-2-((1-(bis(4-(methylthio)phenoxy)phosphoryl)-2-methylpropyl)carbamoyl)pyrrolidin-1-yl)-3,3-dimethyl-1-oxobutan-2-yl)carbamate

^{31}P NMR (162 MHz, Chloroform-d) δ 18.27 (s, 54%), 18.12 (s, 46%).

^{1}H NMR (400 MHz, Chloroform-d) δ 7.59 (dd, J = 197.2, 10.6 Hz, 1H, NH), 7.23 – 7.09 (m, 6H, 6xArH), 7.09 – 6.94 (m, 2H, 2xArH), 5.30 (d, J = 9.4 Hz, 1H, NH), 4.75 – 4.63 (m, 1H, CHP), 4.63 – 4.55 (m, 1H, CH), 4.33 (dd, J = 23.3, 9.2 Hz, 1H, CH), 3.93 – 3.80 (m, 1H, 0.5xCH$_2$), 3.66 – 3.53 (m, 1H, 0.5xCH$_2$), 2.43 (d, J = 4.7 Hz, 6H, 2xSCH$_3$), 2.41 – 2.16 (m, 2H, 0.5xCH$_2$, CH), 2.16 – 1.73 (m, 3H, 1.5xCH$_2$), 1.35 (d, J = 27.9 Hz, 9H, Boc), 1.10 – 0.93 (m, 15H, 5xCH$_3$).

HRMS: $C_{34}H_{50}N_3O_7PS_2$ [M+Na]$^+$ calc. 730.2725 found 730.2716

HPLC: purity >99%; retention time 19.860 min

Chromatogram

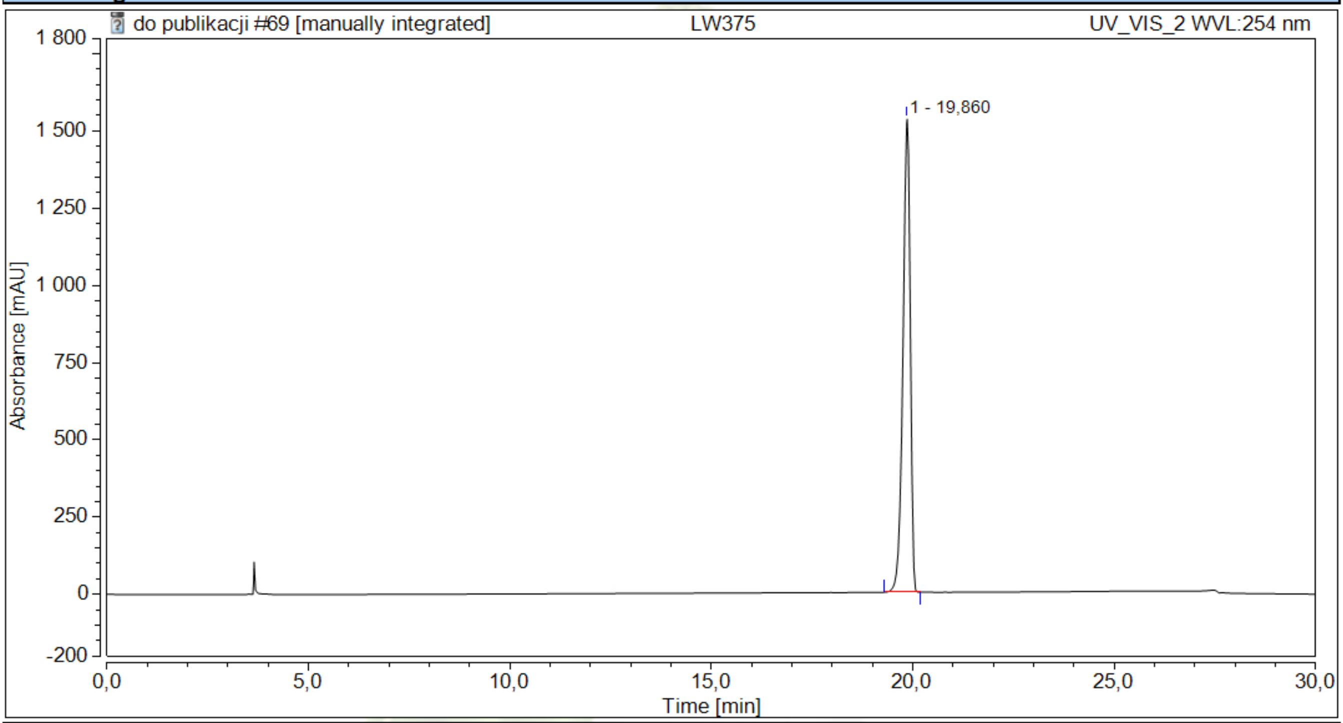

Integration Results

No.	Peak Name	Retention Time min	Area mAU*min	Height mAU	Relative Area %	Relative Height %	Amount n.a.
1		19,860	320,962	1531,024	100,00	100,00	n.a.
**Total:**			**320,962**	**1531,024**	**100,00**	**100,00**	

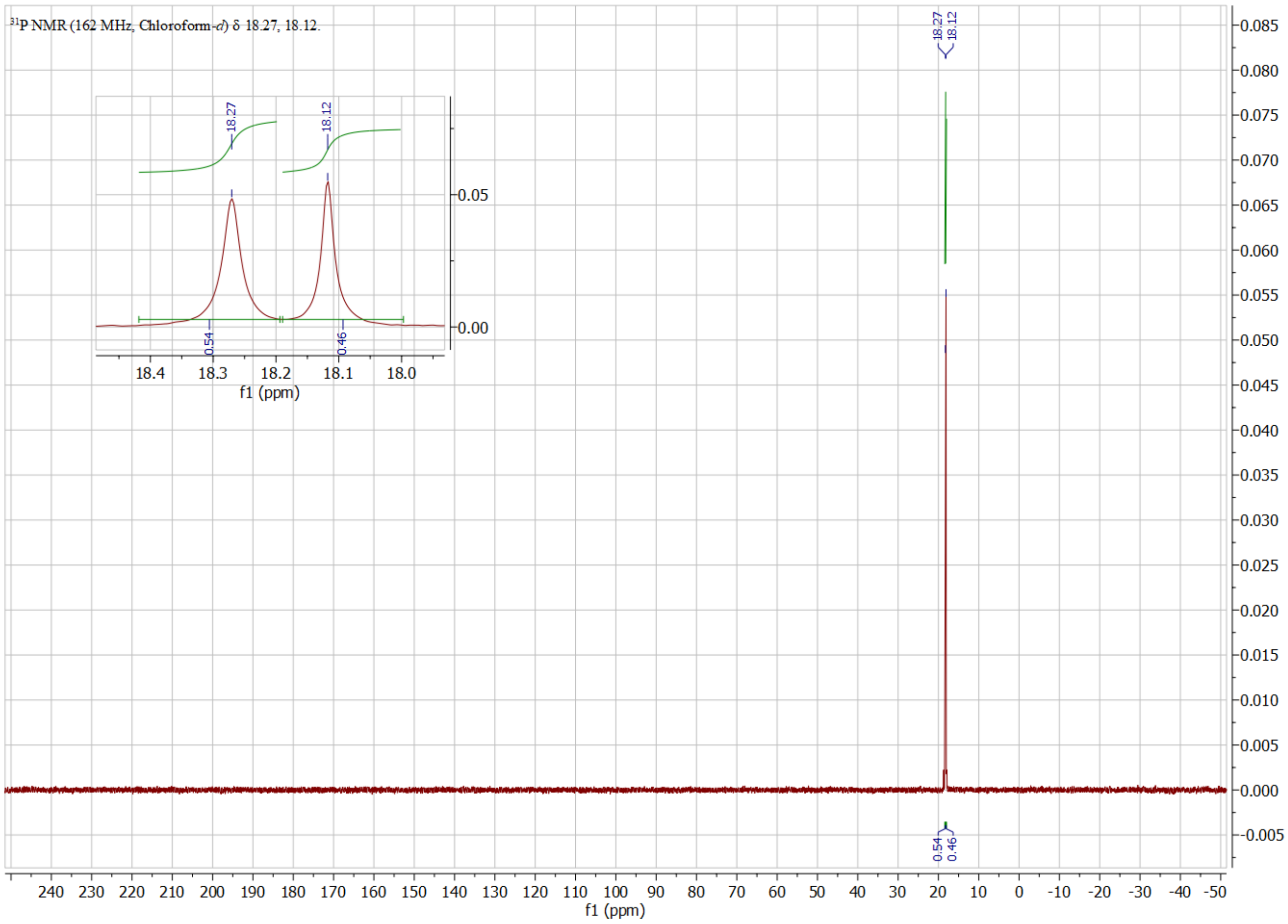
31P NMR (162 MHz, Chloroform-d) δ 18.27, 18.12.
18.27
18.12
0.54
0.46
f1 (ppm)

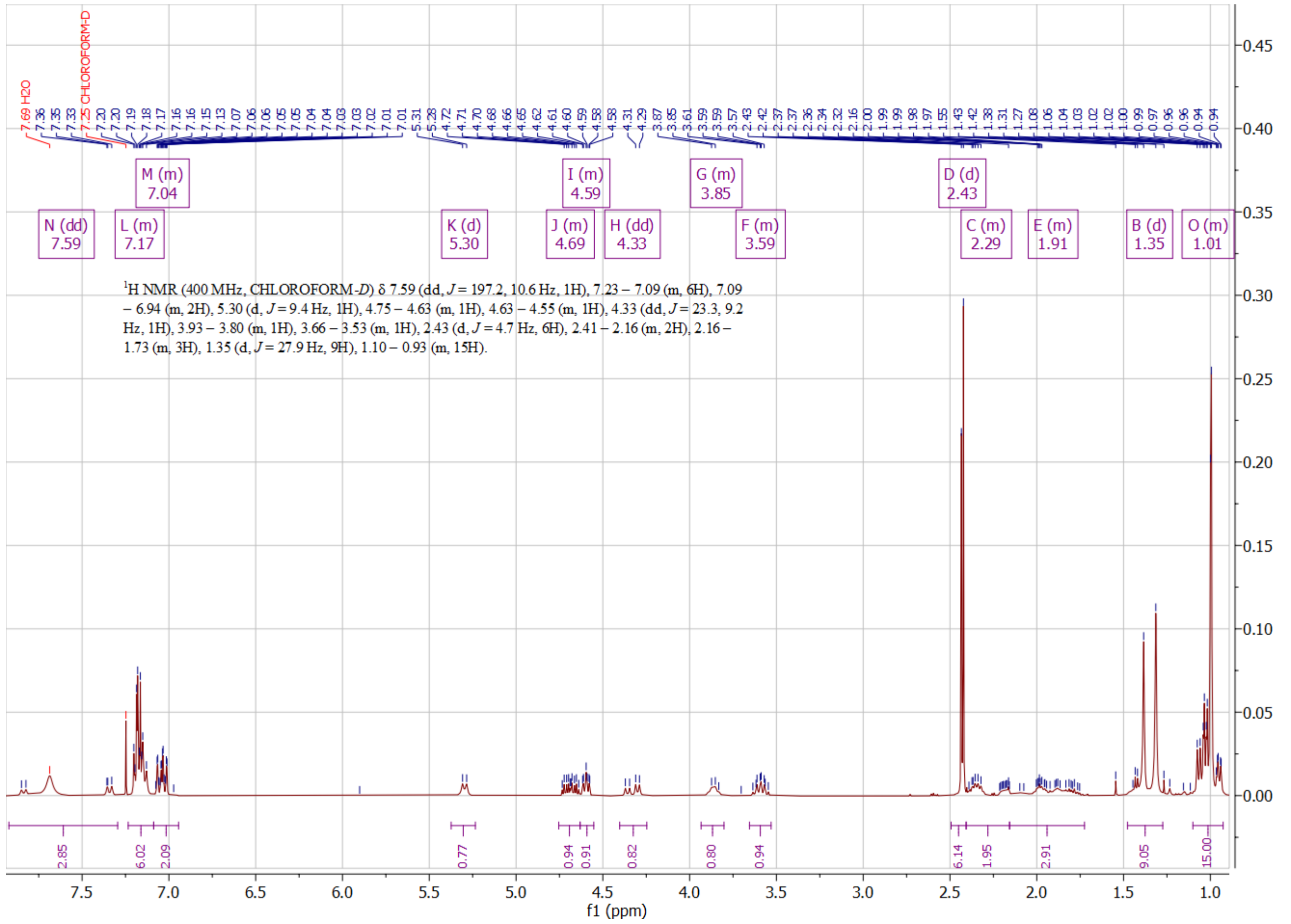
1H NMR (400 MHz, CHLOROFORM-D) δ 7.59 (dd, J = 197.2, 10.6 Hz, 1H), 7.23 – 7.09 (m, 6H), 7.09 – 6.94 (m, 2H), 5.30 (d, J = 9.4 Hz, 1H), 4.75 – 4.63 (m, 1H), 4.63 – 4.55 (m, 1H), 4.33 (dd, J = 23.3, 9.2 Hz, 1H), 3.93 – 3.80 (m, 1H), 3.66 – 3.53 (m, 1H), 2.43 (d, J = 4.7 Hz, 6H), 2.41 – 2.16 (m, 2H), 2.16 – 1.73 (m, 3H), 1.35 (d, J = 27.9 Hz, 9H), 1.10 – 0.93 (m, 15H).
7.69 H2O
7.25 CHLOROFORM-D
N (dd) 7.59
M (m) 7.04
L (m) 7.17
K (d) 5.30
I (m) 4.59
J (m) 4.69
H (dd) 4.33
G (m) 3.85
F (m) 3.59
D (d) 2.43
C (m) 2.29
E (m) 1.91
B (d) 1.35
O (m) 1.01
f1 (ppm)

(70) Boc-L-Nle-L-Pro-Val^{P}(OPh-4-SCH$_3$)$_2$

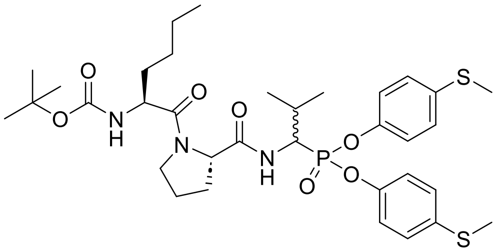

Name: *tert*-butyl ((2S)-1-((2S)-2-((1-(bis(4-(methylthio)phenoxy)phosphoryl)-2-methylpropyl)carbamoyl)pyrrolidin-1-yl)-1-oxohexan-2-yl)carbamate
^{31}P NMR (162 MHz, Chloroform-d) δ 18.46 (s, 50%), 17.99 (s, 50%).
^{1}H NMR (400 MHz, Chloroform-d) δ 7.40 (dd, J = 17.8, 10.3 Hz, 1H, NH), 7.23 – 6.99 (m, 8H, 8xArH), 5.27 (dd, J = 47.8, 8.7 Hz, 1H, NH), 4.77 – 4.49 (m, 2H, CHP, CH), 4.46 – 4.29 (m, 1H, CH), 3.80 – 3.42 (m, 2H, CH$_2$), 2.43 (d, J = 2.4 Hz, 6H, 2xSCH$_3$), 2.40 – 2.32 (m, 1H, CH), 2.22 – 1.80 (m, 3H, 1.5xCH$_2$), 1.70 – 1.44 (m, 2H, CH$_2$), 1.41 (d, J = 1.8 Hz, 9H, Boc), 1.38 – 1.09 (m, 5H, 2.5xCH$_2$), 1.09 – 0.94 (m, 6H, 2xCH$_3$), 0.81 (dt, J = 45.9, 7.1 Hz, 3H, CH$_3$).
HRMS: $C_{34}H_{50}N_3O_7PS_2$ [M+Na]$^+$ calc. 730.2725 found 730.2683
HPLC: purity >99%; retention time 19.643 min

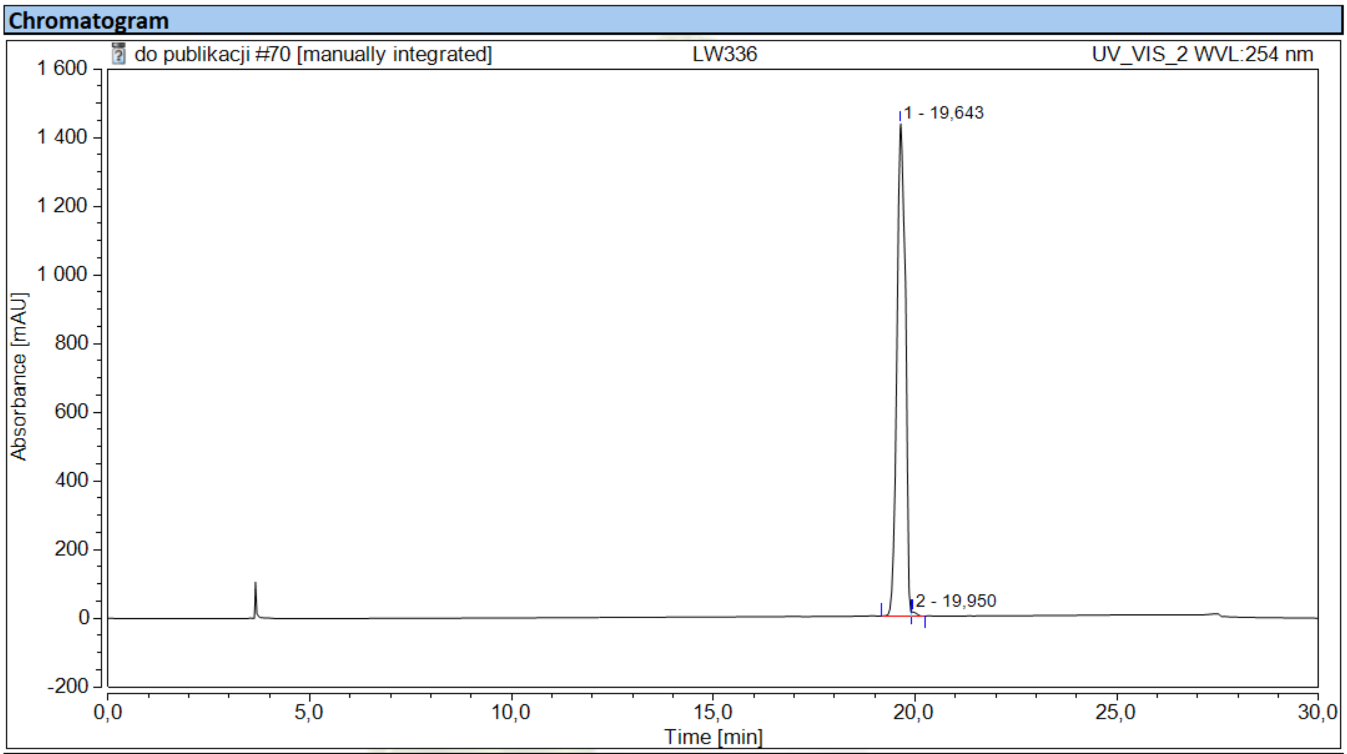

Integration Results

No.	Peak Name	Retention Time min	Area mAU*min	Height mAU	Relative Area %	Relative Height %	Amount n.a.
1		19,643	348,981	1433,133	99,53	99,20	n.a.
2		19,950	1,632	11,530	0,47	0,80	n.a.
**Total:**			**350,614**	**1444,662**	**100,00**	**100,00**	

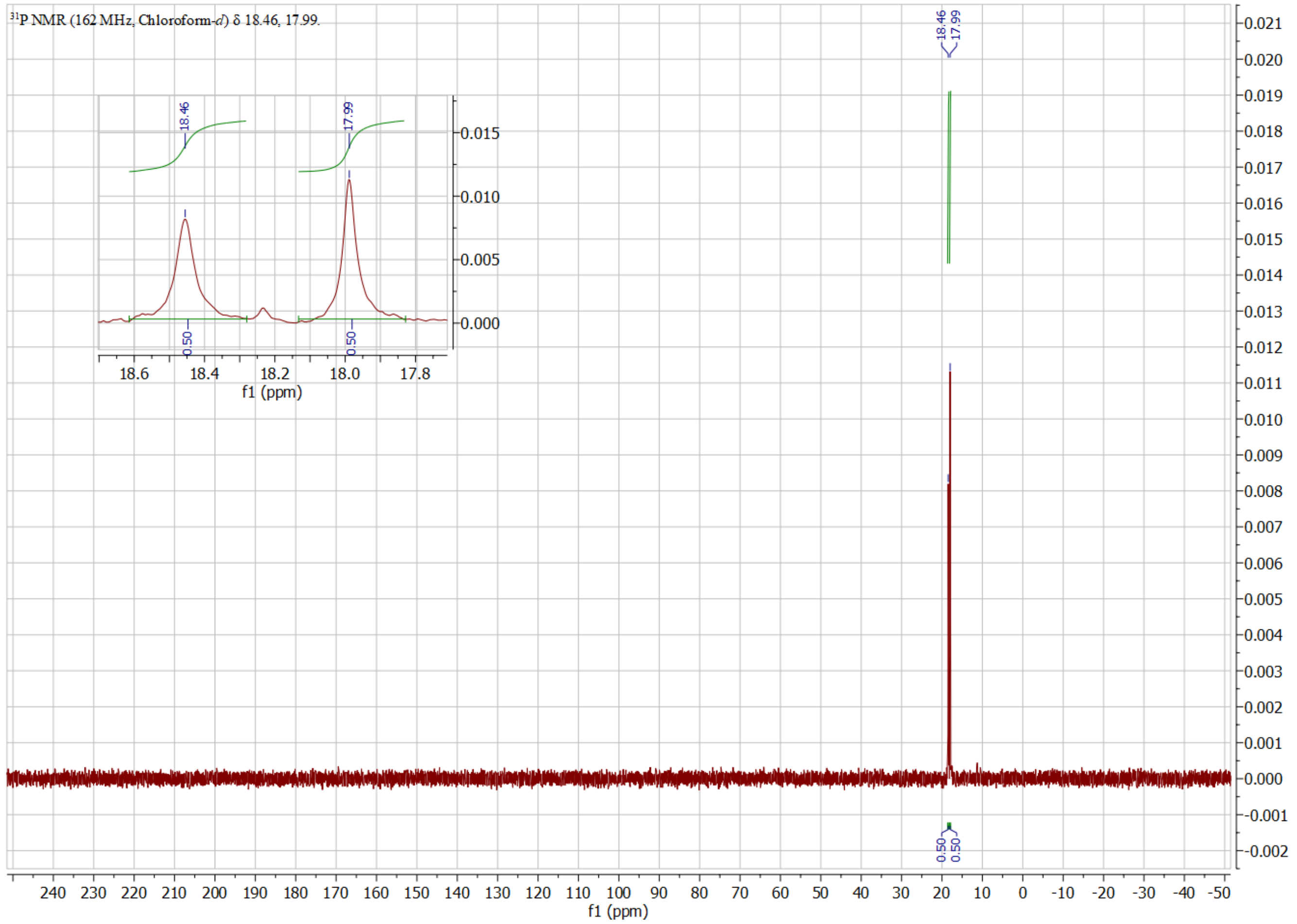

31P NMR (162 MHz, Chloroform-d) δ 18.46, 17.99.
18.46
17.99
f1 (ppm)

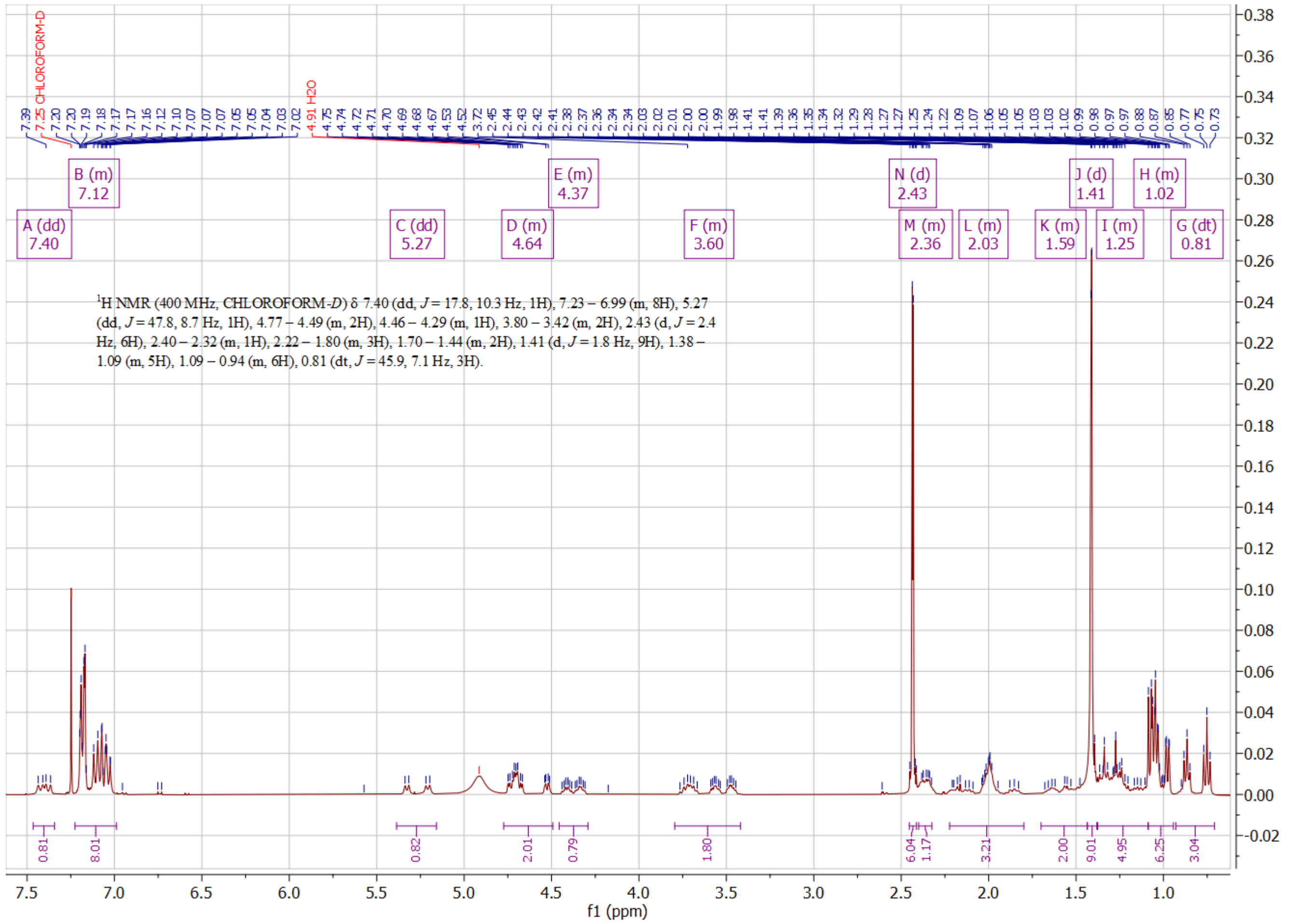

7.25 CHLOROFORM-D
4.91 H2O
A (dd) 7.40
B (m) 7.12
C (dd) 5.27
D (m) 4.64
E (m) 4.37
F (m) 3.60
N (d) 2.43
M (m) 2.36
L (m) 2.03
K (m) 1.59
J (d) 1.41
I (m) 1.25
H (m) 1.02
G (dt) 0.81
1H NMR (400 MHz, CHLOROFORM-D) δ 7.40 (dd, J = 17.8, 10.3 Hz, 1H), 7.23 – 6.99 (m, 8H), 5.27 (dd, J = 47.8, 8.7 Hz, 1H), 4.77 – 4.49 (m, 2H), 4.46 – 4.29 (m, 1H), 3.80 – 3.42 (m, 2H), 2.43 (d, J = 2.4 Hz, 6H), 2.40 – 2.32 (m, 1H), 2.22 – 1.80 (m, 3H), 1.70 – 1.44 (m, 2H), 1.41 (d, J = 1.8 Hz, 9H), 1.38 – 1.09 (m, 5H), 1.09 – 0.94 (m, 6H), 0.81 (dt, J = 45.9, 7.1 Hz, 3H).
f1 (ppm)

(71) Boc-D-Nle-L-Pro-Val^P(OPh-4-SCH$_3$)$_2$

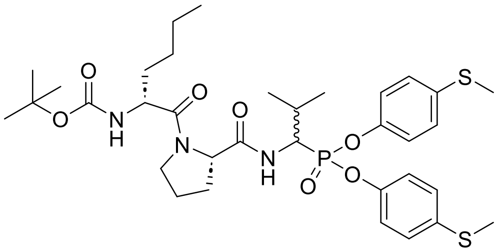

Name: *tert*-butyl ((2R)-1-((2S)-2-((1-(bis(4-(methylthio)phenoxy)phosphoryl)-2-methylpropyl)carbamoyl)pyrrolidin-1-yl)-1-oxohexan-2-yl)carbamate

^{31}P NMR (162 MHz, Chloroform-d) δ 18.63 (s, 51%), 18.31 (s, 49%).

^{1}H NMR (400 MHz, Chloroform-d) δ 7.58 (dd, J = 89.6, 10.0 Hz, 1H, NH), 7.22 – 7.12 (m, 6H, 6xArH), 7.09 – 7.01 (m, 2H, 2xArH), 5.20 (dd, J = 12.0, 8.4 Hz, 1H, NH), 4.70 – 4.51 (m, 2H, CHP, CH), 4.49 – 4.32 (m, 1H, CH), 3.87 – 3.69 (m, 1H, 0.5xCH$_2$), 3.52 – 3.38 (m, 1H, 0.5xCH$_2$), 2.43 (d, J = 5.7 Hz, 6H, 2xSCH$_3$), 2.41 – 2.20 (m, 2H, 0.5xCH$_2$, CH), 2.18 – 1.74 (m, 3H, 1.5xCH$_2$), 1.73 – 1.41 (m, 3H, 1.5xCH$_2$), 1.35 (d, J = 20.5 Hz, 9H, Boc), 1.32 – 1.20 (m, 3H, 1.5xCH$_2$), 1.11 – 0.94 (m, 6H, 2xCH$_3$), 0.92 – 0.84 (m, 3H, CH$_3$).

HRMS: $C_{34}H_{50}N_3O_7PS_2$ [M+Na]$^+$ calc. 730.2725 found 730.2729

HPLC: purity >99%; retention time 19.620 min

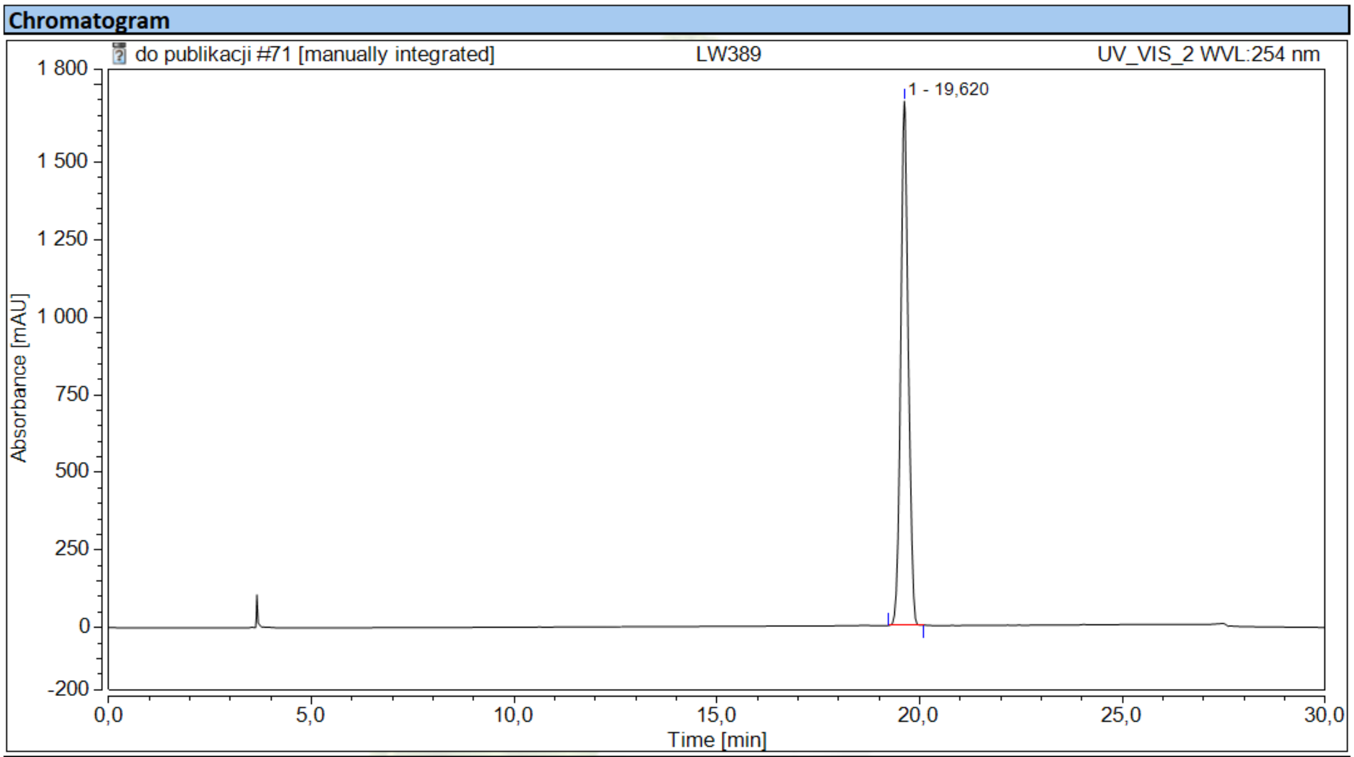

Integration Results

No.	Peak Name	Retention Time min	Area mAU*min	Height mAU	Relative Area %	Relative Height %	Amount n.a.
1		19,620	365,230	1688,818	100,00	100,00	n.a.
**Total:**			**365,230**	**1688,818**	**100,00**	**100,00**	

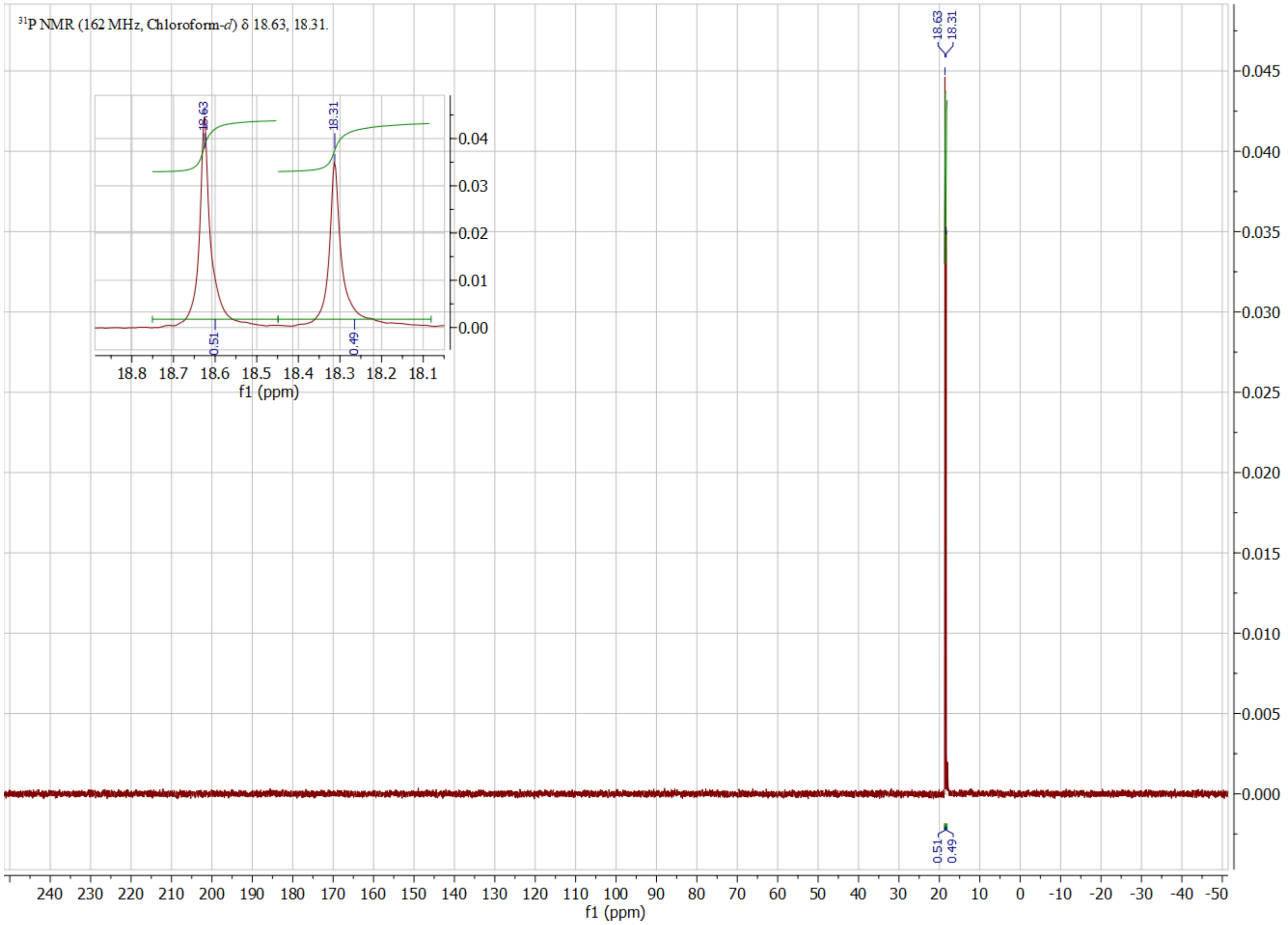
31P NMR (162 MHz, Chloroform-d) δ 18.63, 18.31.
18.63
18.31
0.51
0.49
f1 (ppm)

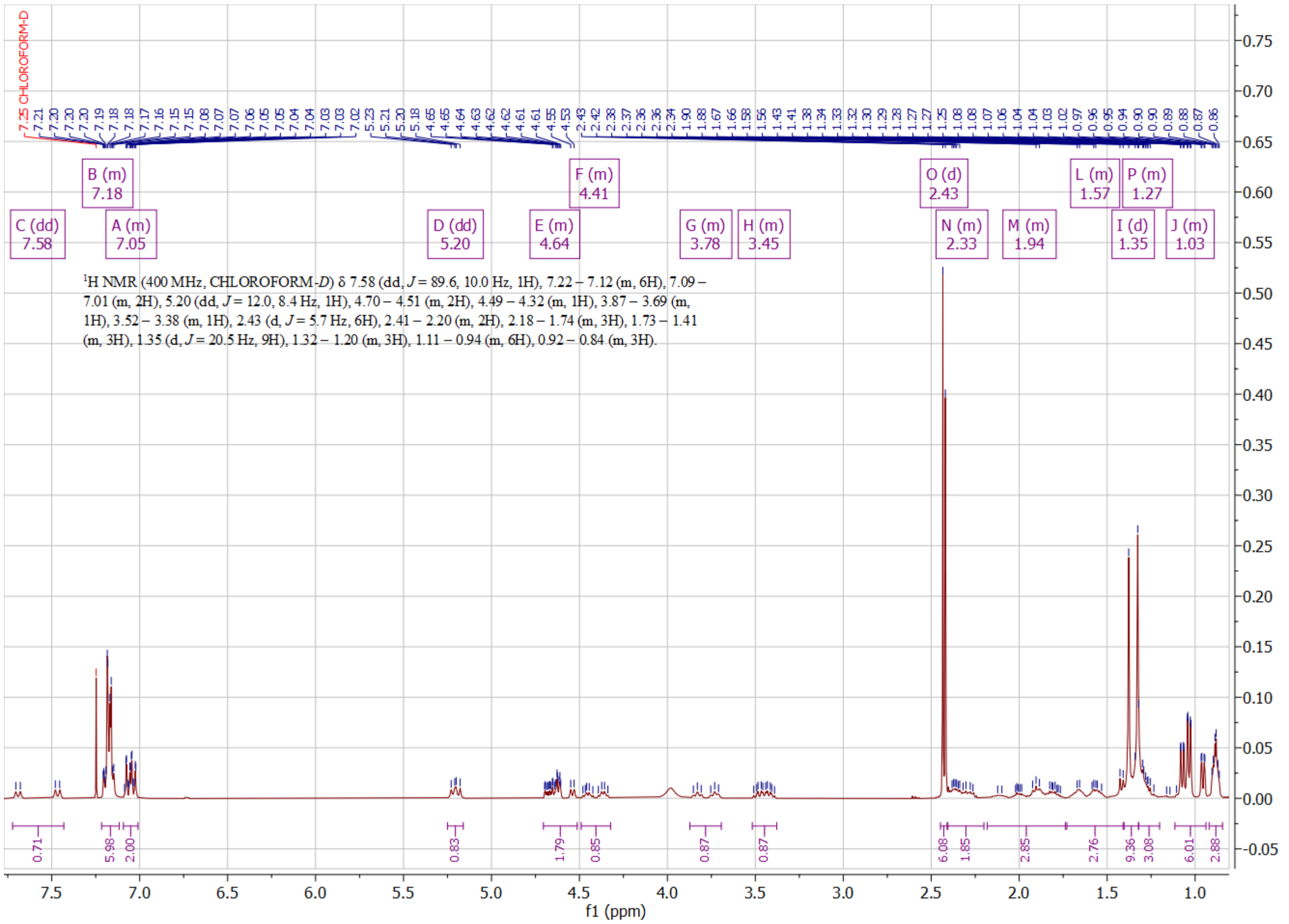
7.25 CHLOROFORM-D
C (dd) 7.58
B (m) 7.18
A (m) 7.05
D (dd) 5.20
E (m) 4.64
F (m) 4.41
G (m) 3.78
H (m) 3.45
O (d) 2.43
N (m) 2.33
M (m) 1.94
L (m) 1.57
I (d) 1.35
P (m) 1.27
J (m) 1.03
1H NMR (400 MHz, CHLOROFORM-D) δ 7.58 (dd, J = 89.6, 10.0 Hz, 1H), 7.22 – 7.12 (m, 6H), 7.09 – 7.01 (m, 2H), 5.20 (dd, J = 12.0, 8.4 Hz, 1H), 4.70 – 4.51 (m, 2H), 4.49 – 4.32 (m, 1H), 3.87 – 3.69 (m, 1H), 3.52 – 3.38 (m, 1H), 2.43 (d, J = 5.7 Hz, 6H), 2.41 – 2.20 (m, 2H), 2.18 – 1.74 (m, 3H), 1.73 – 1.41 (m, 3H), 1.35 (d, J = 20.5 Hz, 9H), 1.32 – 1.20 (m, 3H), 1.11 – 0.94 (m, 6H), 0.92 – 0.84 (m, 3H).
f1 (ppm)

(72) Boc-L-Leu-L-Pro- Val^{P}(OPh-4-SCH$_3$)$_2$

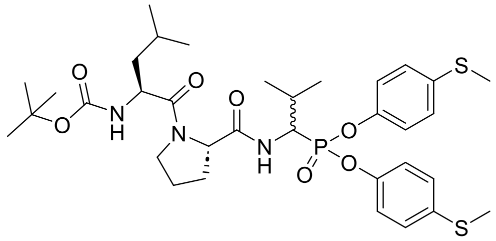

Name: *tert*-butyl ((2S)-1-((2S)-2-((1-(bis(4-(methylthio)phenoxy)phosphoryl)-2-methylpropyl)carbamoyl)pyrrolidin-1-yl)-4-methyl-1-oxopentan-2-yl)carbamate

^{31}P NMR (162 MHz, Chloroform-d) δ 18.51 (s, 55%), 18.04 (s, 45%).

^{1}H NMR (400 MHz, Chloroform-d) δ 7.43 (dd, J = 36.3, 9.9 Hz, 1H, NH), 7.24 – 7.15 (m, 4H, 4xArH), 7.15 – 7.00 (m, 4H, 4xArH), 5.18 (dd, J = 49.4, 8.9 Hz, 1H, NH), 4.76 – 4.69 (m, 1H, CHP), 4.69 – 4.51 (m, 1H, CH), 4.51 – 4.36 (m, 1H, CH), 3.78 – 3.64 (m, 1H, 0.5xCH$_2$), 3.58 – 3.39 (m, 1H, 0.5xCH$_2$), 2.43 (d, J = 3.2 Hz, 6H, 2xSCH$_3$), 2.39 – 2.31 (m, 1H, CH), 2.26 – 2.04 (m, 1H, CH), 2.04 – 1.85 (m, 2H, CH$_2$), 1.85 – 1.45 (m, 2H, CH$_2$), 1.41 (d, J = 1.4 Hz, 9H, Boc), 1.36 – 1.11 (m, 2H, CH$_2$), 1.06 – 0.93 (m, 6H, 2xCH$_3$), 0.99 (dd, J = 68.2, 6.8 Hz, 3H, CH$_3$), 0.76 (dd, J = 58.0, 6.6 Hz, 3H, CH$_3$).

HRMS: $C_{34}H_{50}N_3O_7PS_2$ $[M+Na]^+$ calc. 730.2725 found 730.2708

HPLC: purity >99%; retention time 19.523 and 19.667 min

Chromatogram

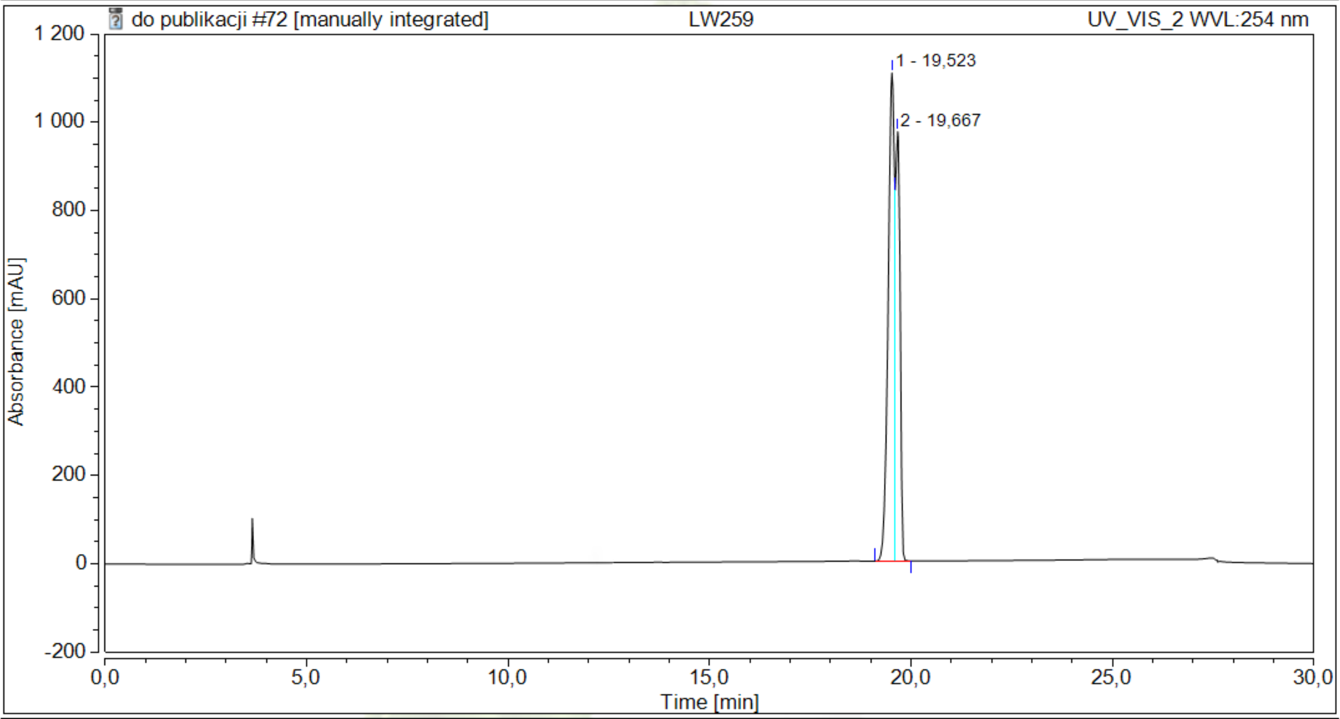

Integration Results

No.	Peak Name	Retention Time min	Area mAU*min	Height mAU	Relative Area %	Relative Height %	Amount n.a.
1		19,523	199,505	1106,050	61,21	53,20	n.a.
2		19,667	126,434	972,904	38,79	46,80	n.a.
**Total:**			**325,939**	**2078,953**	**100,00**	**100,00**	

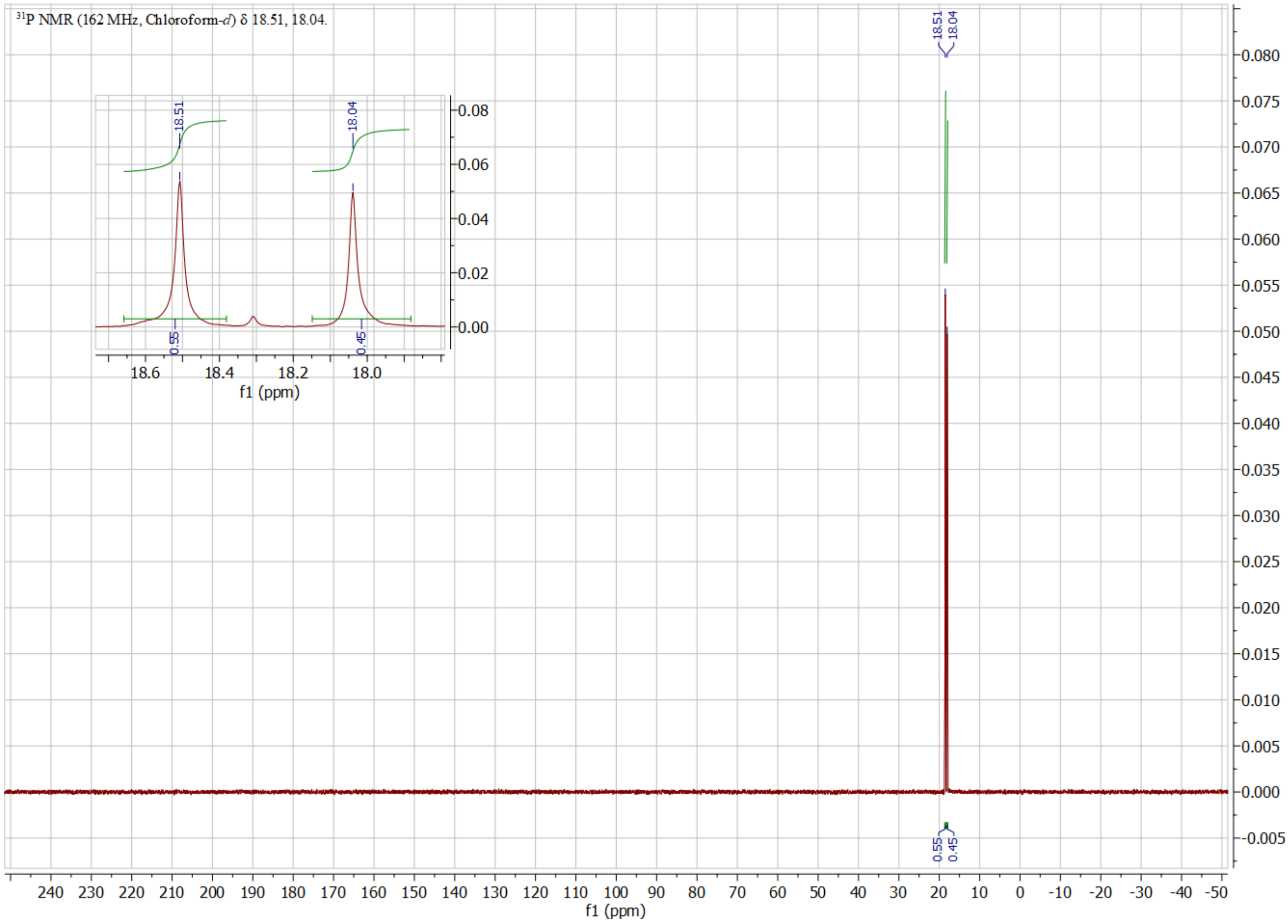

³¹P NMR (162 MHz, Chloroform-d) δ 18.51, 18.04.
f1 (ppm)

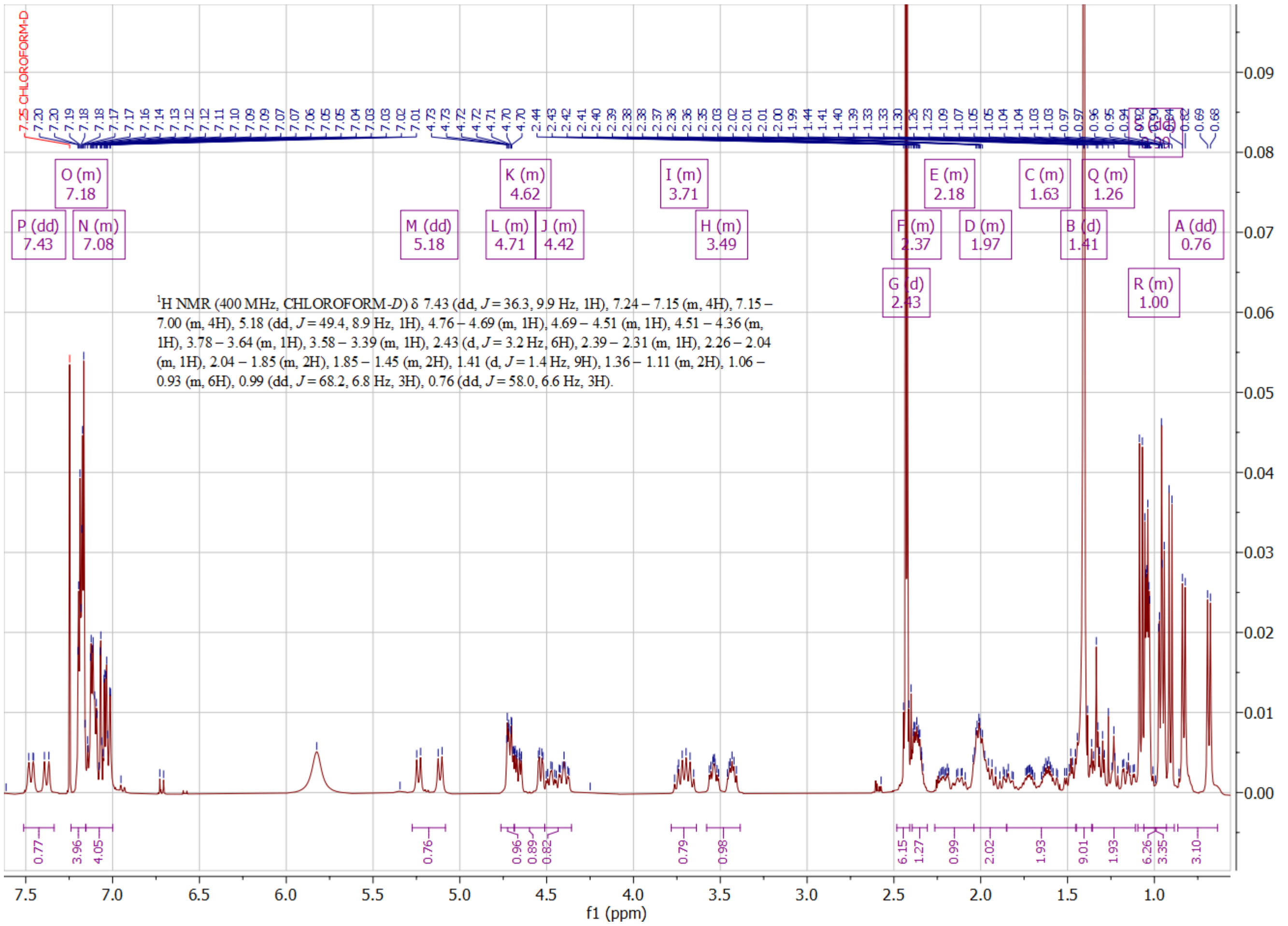

CHLOROFORM-D
^{1}H NMR (400 MHz, CHLOROFORM-D) δ 7.43 (dd, J = 36.3, 9.9 Hz, 1H), 7.24 – 7.15 (m, 4H), 7.15 – 7.00 (m, 4H), 5.18 (dd, J = 49.4, 8.9 Hz, 1H), 4.76 – 4.69 (m, 1H), 4.69 – 4.51 (m, 1H), 4.51 – 4.36 (m, 1H), 3.78 – 3.64 (m, 1H), 3.58 – 3.39 (m, 1H), 2.43 (d, J = 3.2 Hz, 6H), 2.39 – 2.31 (m, 1H), 2.26 – 2.04 (m, 1H), 2.04 – 1.85 (m, 2H), 1.85 – 1.45 (m, 2H), 1.41 (d, J = 1.4 Hz, 9H), 1.36 – 1.11 (m, 2H), 1.06 – 0.93 (m, 6H), 0.99 (dd, J = 68.2, 6.8 Hz, 3H), 0.76 (dd, J = 58.0, 6.6 Hz, 3H).
f1 (ppm)

(73) Boc-D-Leu-L-Pro-Val^{P}(OPh-4-SCH$_3$)$_2$

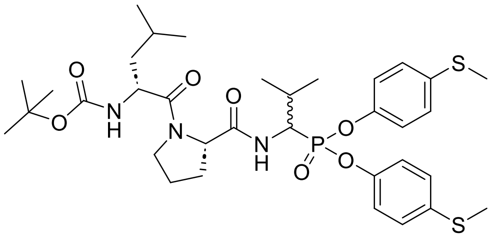

Name: *tert*-butyl ((2R)-1-((2S)-2-((1-(bis(4-(methylthio)phenoxy)phosphoryl)-2-methylpropyl)carbamoyl)pyrrolidin-1-yl)-4-methyl-1-oxopentan-2-yl)carbamate

^{31}P NMR (162 MHz, Chloroform-d) δ 18.54 (s, 64%), 18.23 (s, 36%).

^{1}H NMR (400 MHz, Chloroform-d) δ 7.56 (dd, J = 111.5, 10.2 Hz, 1H, NH), 7.23 – 7.10 (m, 6H, 6xArH), 7.10 – 7.00 (m, 2H, 2xArH), 5.19 (td, J = 8.3, 8.3 Hz, 1H, NH), 4.73 – 4.62 (m, 1H, CHP), 4.62 – 4.53 (m, 1H, CH), 4.53 – 4.37 (m, 1H, CH), 3.90 – 3.75 (m, 1H, 0.5xCH$_2$), 3.51 – 3.36 (m, 1H, 0.5xCH$_2$), 2.43 (d, J = 4.7 Hz, 6H, 2xSCH$_3$), 2.41 – 2.28 (m, 1H, CH), 2.28 – 2.07 (m, 1H, CH), 2.05 – 1.82 (m, 2H, 2xCH$_2$), 1.82 – 1.39 (m, 4H, 2xCH$_2$), 1.35 (d, J = 17.5 Hz, 9H, Boc), 1.10 – 0.90 (m, 12H, 4xCH$_3$).

HRMS: $C_{34}H_{50}N_3O_7PS_2$ [M+Na]$^+$ calc. 730.2725 found 730.2708

HPLC: purity >99%; retention time 19.597 and 19.780 min

Chromatogram

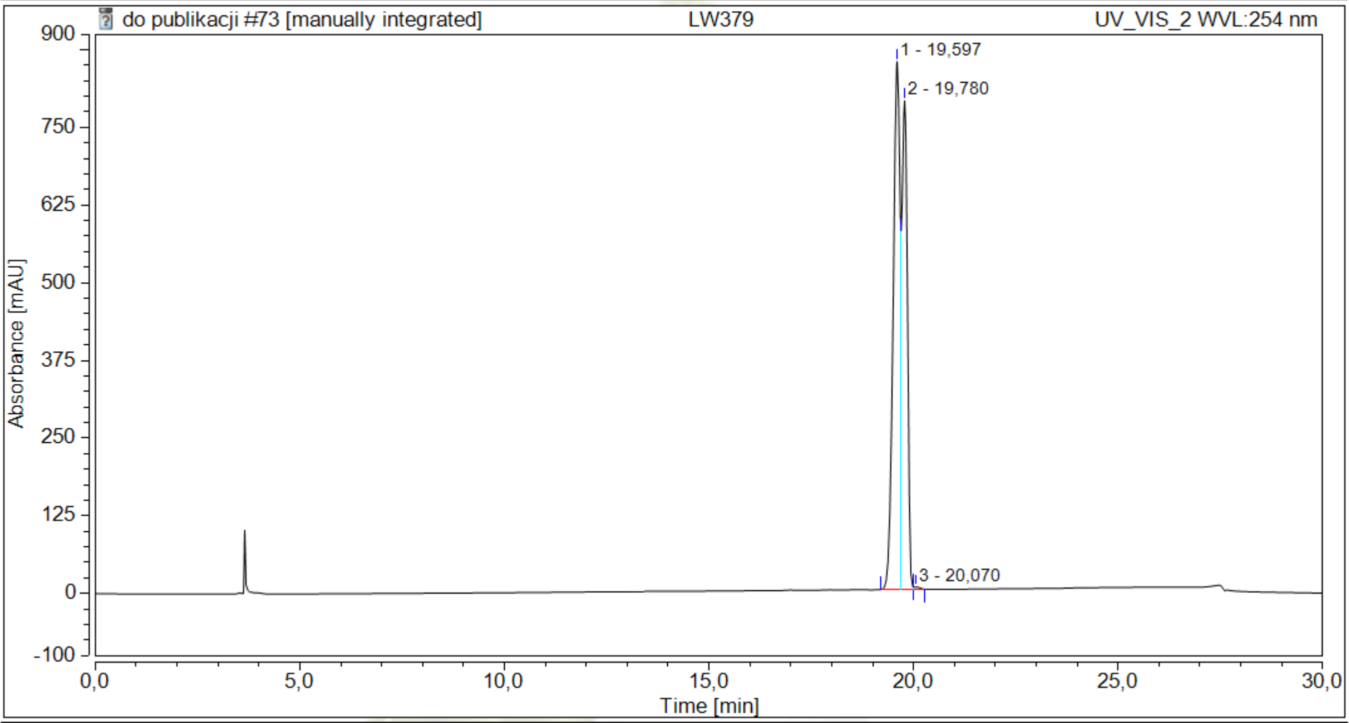

Integration Results

No.	Peak Name	Retention Time min	Area mAU*min	Height mAU	Relative Area %	Relative Height %	Amount n.a.
1		19,597	164,217	849,469	56,93	51,81	n.a.
2		19,780	123,581	786,363	42,84	47,96	n.a.
3		20,070	0,679	3,862	0,24	0,24	n.a.
**Total:**			**288,476**	**1639,694**	**100,00**	**100,00**	

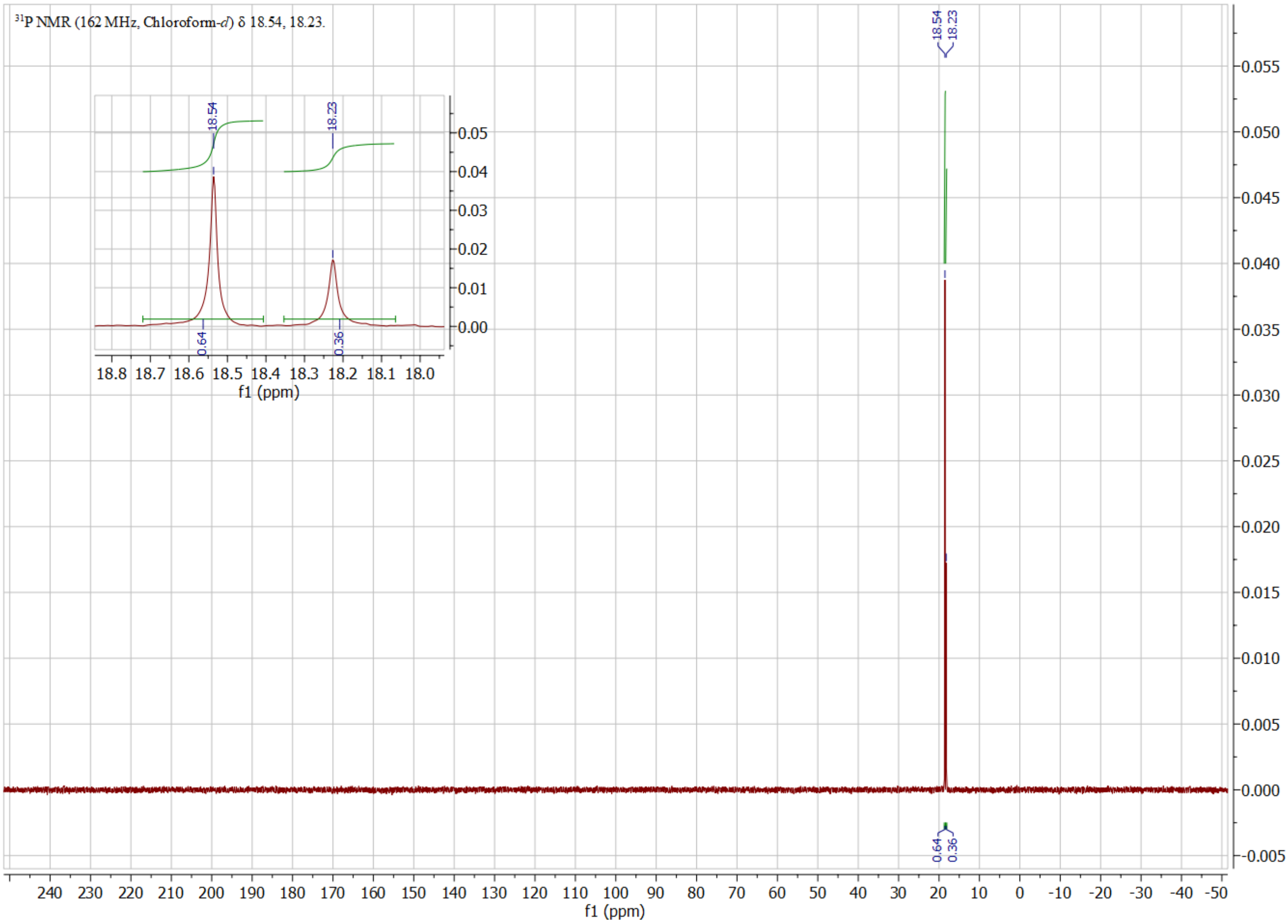
^{31}P NMR (162 MHz, Chloroform-*d*) δ 18.54, 18.23.
f1 (ppm)

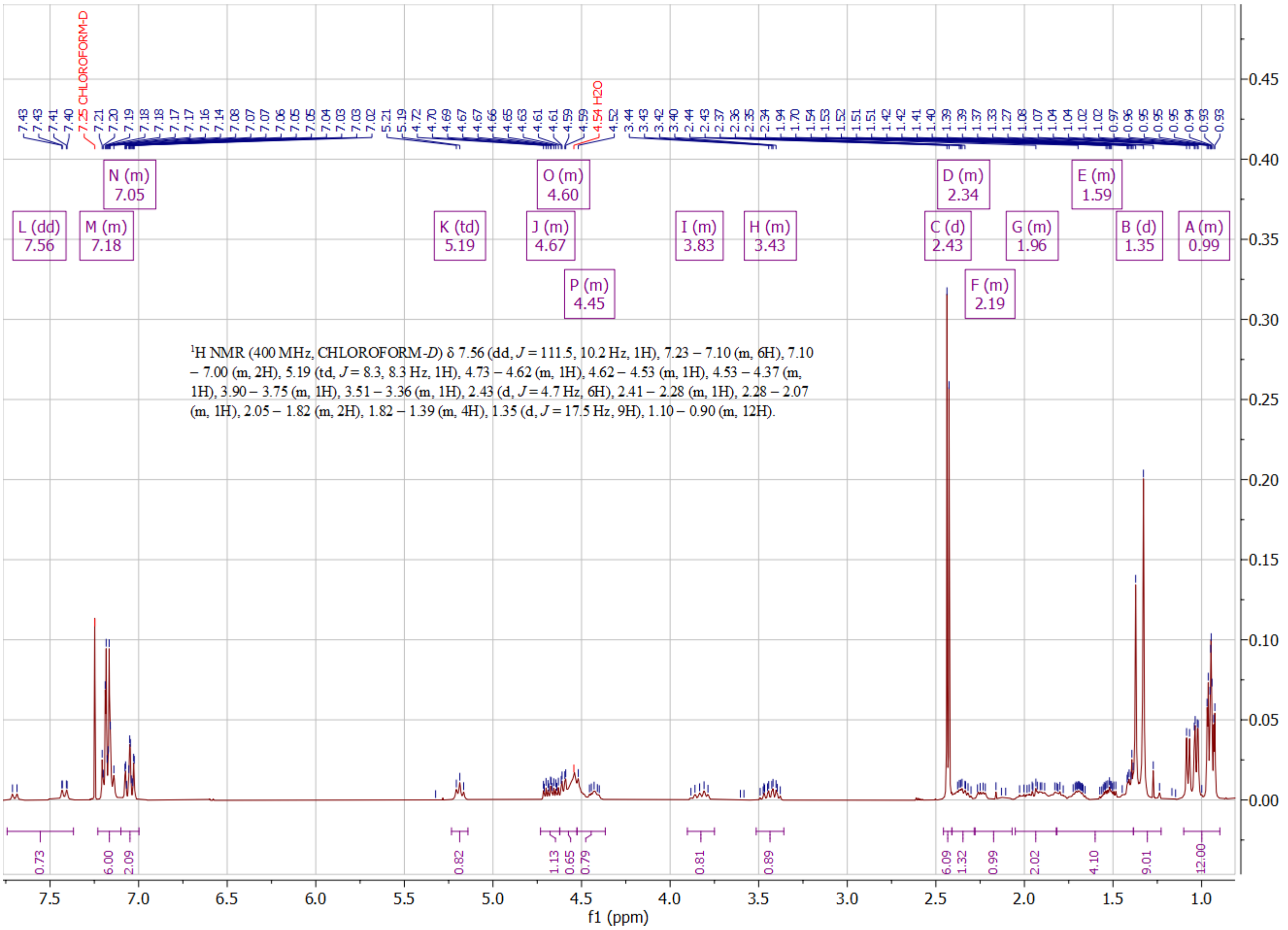
^{1}H NMR (400 MHz, CHLOROFORM-*D*) δ 7.56 (dd, *J* = 111.5, 10.2 Hz, 1H), 7.23 – 7.10 (m, 6H), 7.10 – 7.00 (m, 2H), 5.19 (td, *J* = 8.3, 8.3 Hz, 1H), 4.73 – 4.62 (m, 1H), 4.62 – 4.53 (m, 1H), 4.53 – 4.37 (m, 1H), 3.90 – 3.75 (m, 1H), 3.51 – 3.36 (m, 1H), 2.43 (d, *J* = 4.7 Hz, 6H), 2.41 – 2.28 (m, 1H), 2.28 – 2.07 (m, 1H), 2.05 – 1.82 (m, 2H), 1.82 – 1.39 (m, 4H), 1.35 (d, *J* = 17.5 Hz, 9H), 1.10 – 0.90 (m, 12H).
f1 (ppm)

(74) Boc-L-NptGly-L-Pro-Val^{P}(OPh-4-SCH$_3$)$_2$

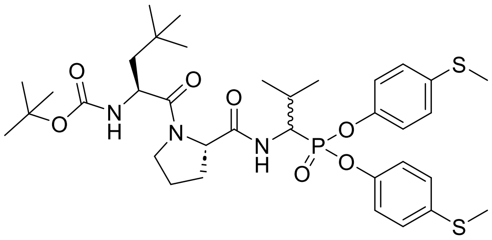

Name: *tert*-butyl ((2S)-1-((2S)-2-((1-(bis(4-(methylthio)phenoxy)phosphoryl)-2-methylpropyl)carbamoyl)pyrrolidin-1-yl)-4,4-dimethyl-1-oxopentan-2-yl)carbamate

^{31}P NMR (162 MHz, Chloroform-d) δ 18.56 (s, 55%), 18.00 (s, 45%).

^{1}H NMR (400 MHz, Chloroform-d) δ 7.41 (dd, J = 60.7, 10.2 Hz, 1H, NH), 7.23 – 7.15 (m, 4H, 4xArH), 7.15 – 7.09 (m, 2H, 2xArH), 7.09 – 6.99 (m, 2H, 2xArH), 5.18 (dd, J = 52.7, 9.1 Hz, 1H, NH), 4.76 – 4.62 (m, 2H, CHP, CH), 4.58 – 4.41 (m, 1H, CH), 3.78 – 3.67 (m, 1H, 0.5xCH$_2$), 3.58 – 3.42 (m, 1H, 0.5xCH$_2$), 2.43 (d, J = 3.7 Hz, 6H, 2xSCH$_3$), 2.42 – 2.30 (m, 2H, 0.5xCH$_2$, CH), 2.21 – 1.80 (m, 3H, 1.5xCH$_2$), 1.40 (s, 9H, Boc), 1.35 – 1.22 (m, 2H, CH$_2$), 1.11 – 0.98 (m, 6H, 2xCH$_3$), 0.89 (d, J = 65.0 Hz, 9H, 3xCH$_3$)

HRMS: $C_{35}H_{52}N_3O_7PS_2$ [M+Na]$^+$ calc. 744.2882 found 744.2855

HPLC: purity >99%; retention time 20.033 and 20.220 min

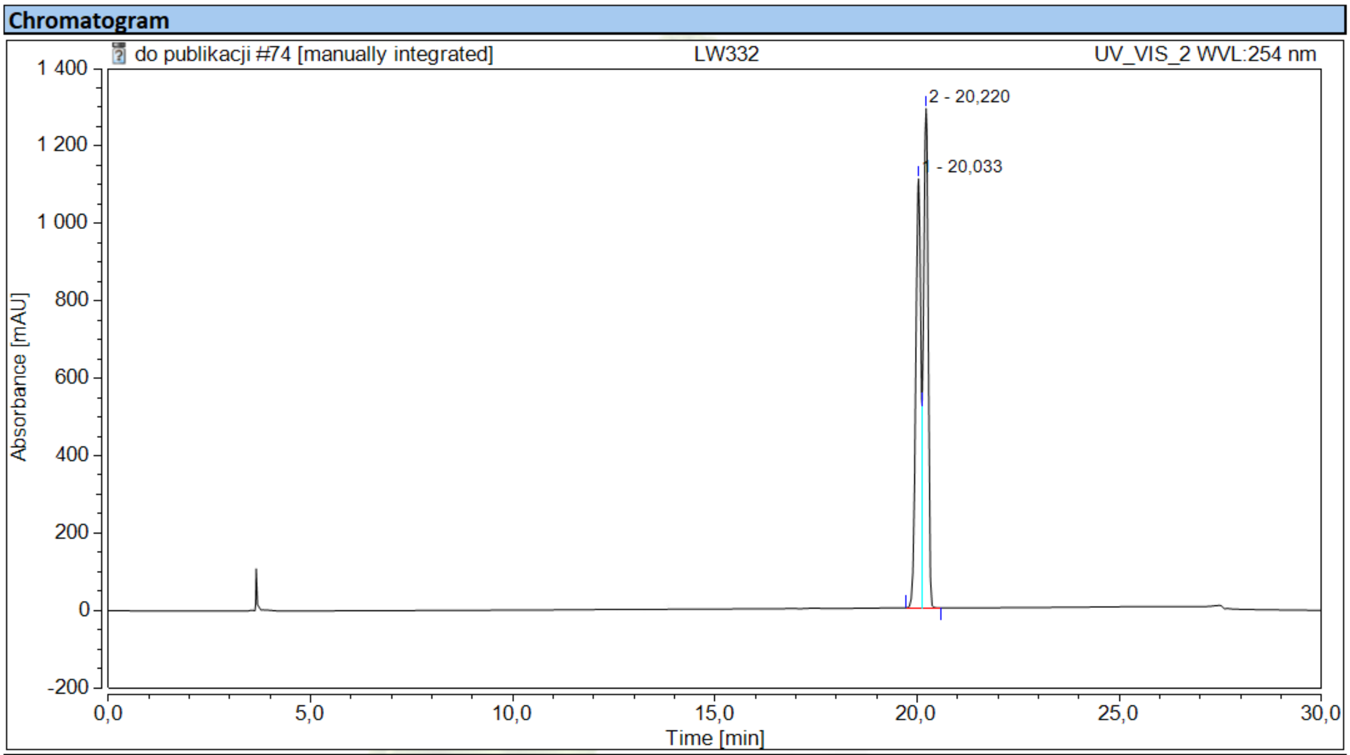

Integration Results

No.	Peak Name	Retention Time min	Area mAU*min	Height mAU	Relative Area %	Relative Height %	Amount n.a.
1		20,033	160,914	1108,590	48,31	46,22	n.a.
2		20,220	172,163	1290,159	51,69	53,78	n.a.
**Total:**			**333,077**	**2398,750**	**100,00**	**100,00**	

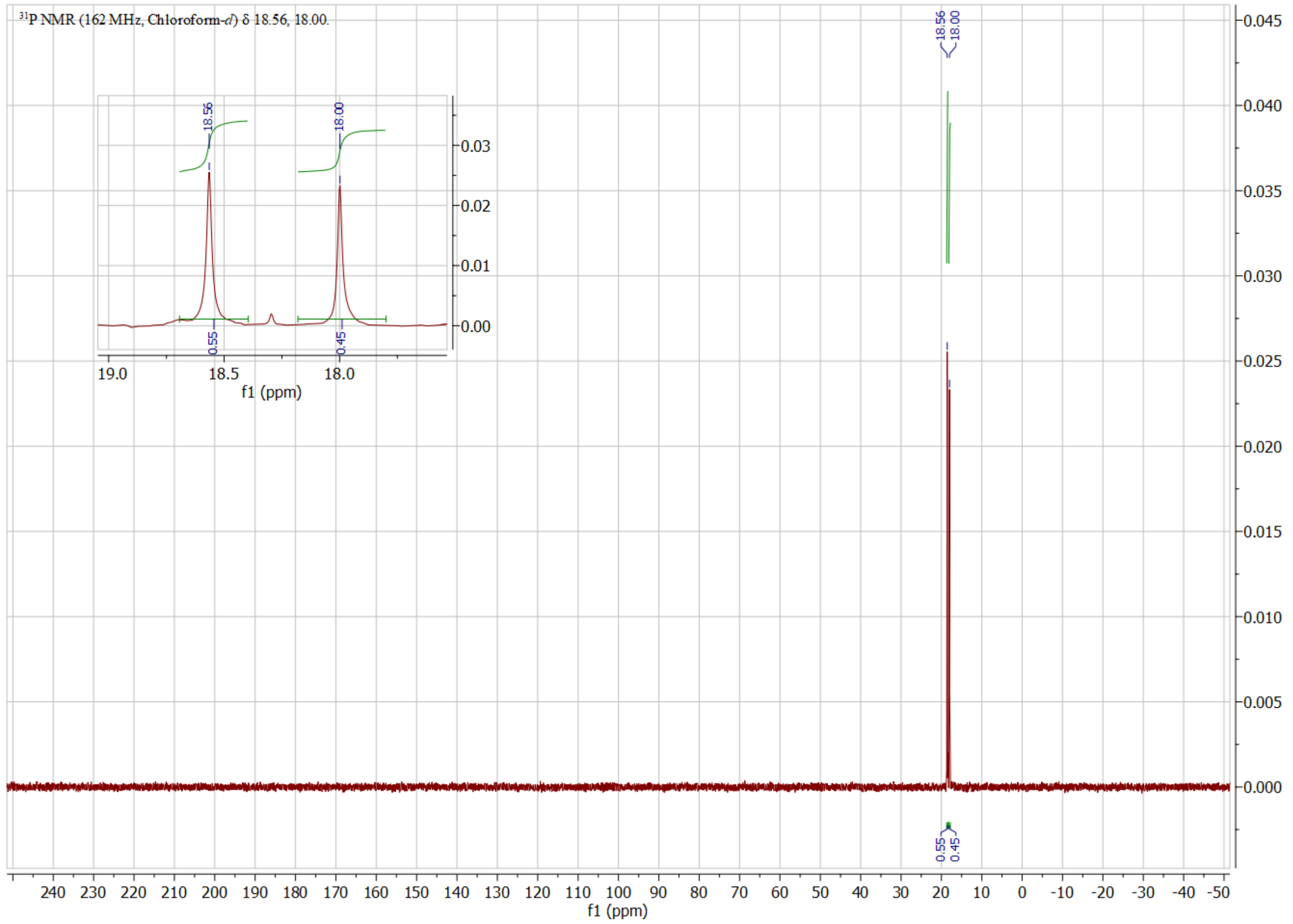

^{31}P NMR (162 MHz, Chloroform-d) δ 18.56, 18.00.
f1 (ppm)

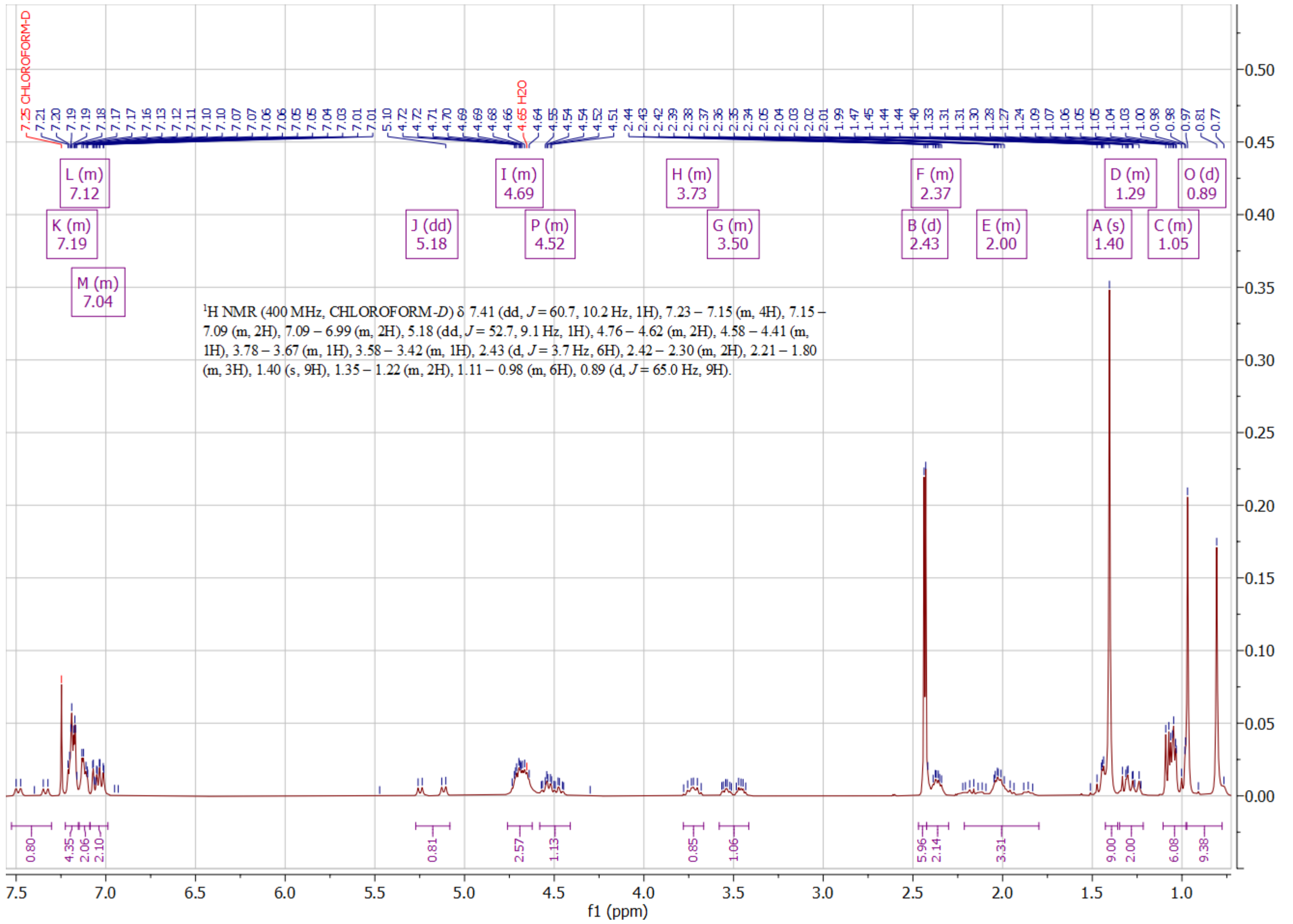

^{1}H NMR (400 MHz, CHLOROFORM-D) δ 7.41 (dd, J = 60.7, 10.2 Hz, 1H), 7.23 – 7.15 (m, 4H), 7.15 – 7.09 (m, 2H), 7.09 – 6.99 (m, 2H), 5.18 (dd, J = 52.7, 9.1 Hz, 1H), 4.76 – 4.62 (m, 2H), 4.58 – 4.41 (m, 1H), 3.78 – 3.67 (m, 1H), 3.58 – 3.42 (m, 1H), 2.43 (d, J = 3.7 Hz, 6H), 2.42 – 2.30 (m, 2H), 2.21 – 1.80 (m, 3H), 1.40 (s, 9H), 1.35 – 1.22 (m, 2H), 1.11 – 0.98 (m, 6H), 0.89 (d, J = 65.0 Hz, 9H).
f1 (ppm)

(75) Boc-L-Met-L-Pro- Val^{P}(OPh-4-SCH$_3$)$_2$

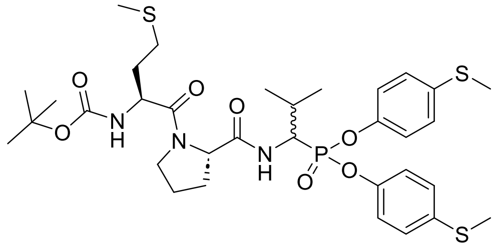

Name: *tert*-butyl ((2S)-1-((2S)-2-((1-(bis(4-(methylthio)phenoxy)phosphoryl)-2-methylpropyl)carbamoyl)pyrrolidin-1-yl)-4-(methylthio)-1-oxobutan-2-yl)carbamate
^{31}P NMR (162 MHz, Chloroform-d) δ 18.46 (s, 53%), 17.96 (s, 47%).
^{1}H NMR (400 MHz, Chloroform-d) δ 7.33 (dd, J = 90.0, 10.0 Hz, 1H, NH), 7.21 – 7.00 (m, 8H, 8xArH), 5.33 (dd, J = 45.1, 8.8 Hz, 1H, NH), 4.77 – 4.45 (m, 3H, CHP, 2xCH), 3.81 – 3.51 (m, 2H, CH$_2$), 2.62 – 2.49 (m, 1H, CH), 2.46 – 2.43 (m, 6H, 2xSCH$_3$), 2.43 – 2.29 (m, 2H, CH$_2$), 2.19 – 1.62 (m, 8H, CH$_3$, 2.5xCH$_2$), 1.50 – 1.31 (m, 9H, Boc), 1.31 – 1.22 (m, 1H, 0.5xCH$_2$), 1.11 – 0.96 (m, 6H, 2xCH$_3$).
HRMS: $C_{33}H_{48}N_3O_7PS_3$ [M+Na]$^+$ calc. 748.2290 found 748.2264
HPLC: purity >99%; retention time 18.557 and 18.837 min

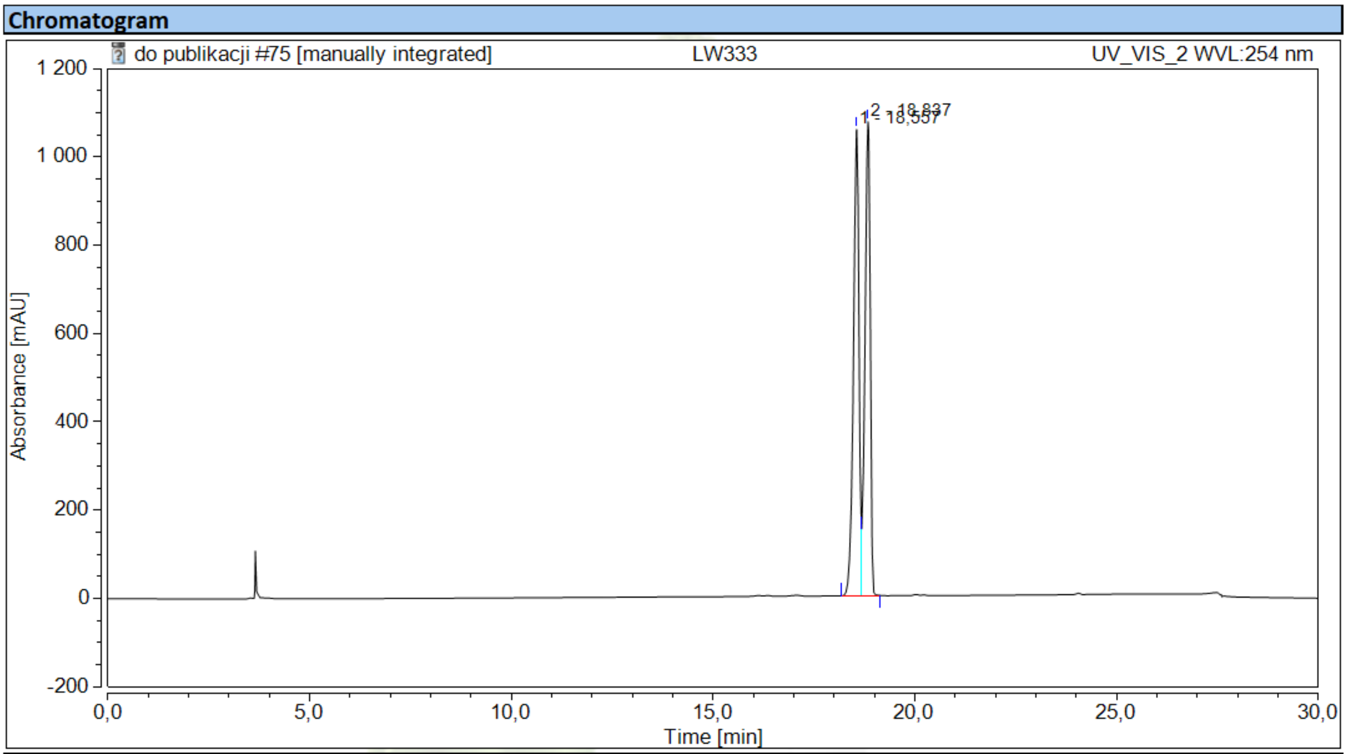

Integration Results

No.	Peak Name	Retention Time min	Area mAU*min	Height mAU	Relative Area %	Relative Height %	Amount n.a.
1		18,557	167,954	1055,893	50,88	49,59	n.a.
2		18,837	162,154	1073,161	49,12	50,41	n.a.
**Total:**			**330,108**	**2129,054**	**100,00**	**100,00**	

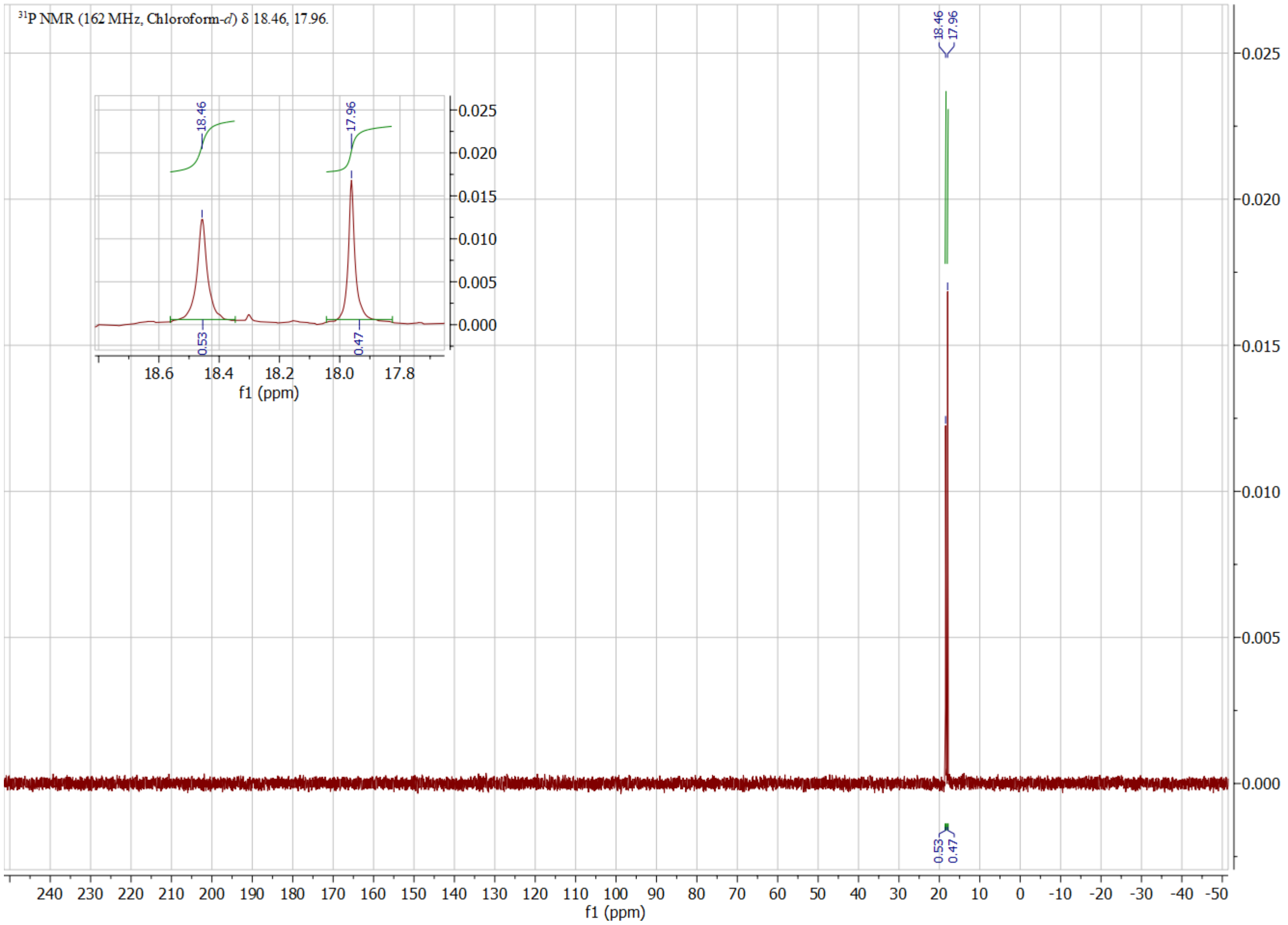

31P NMR (162 MHz, Chloroform-d) δ 18.46, 17.96.

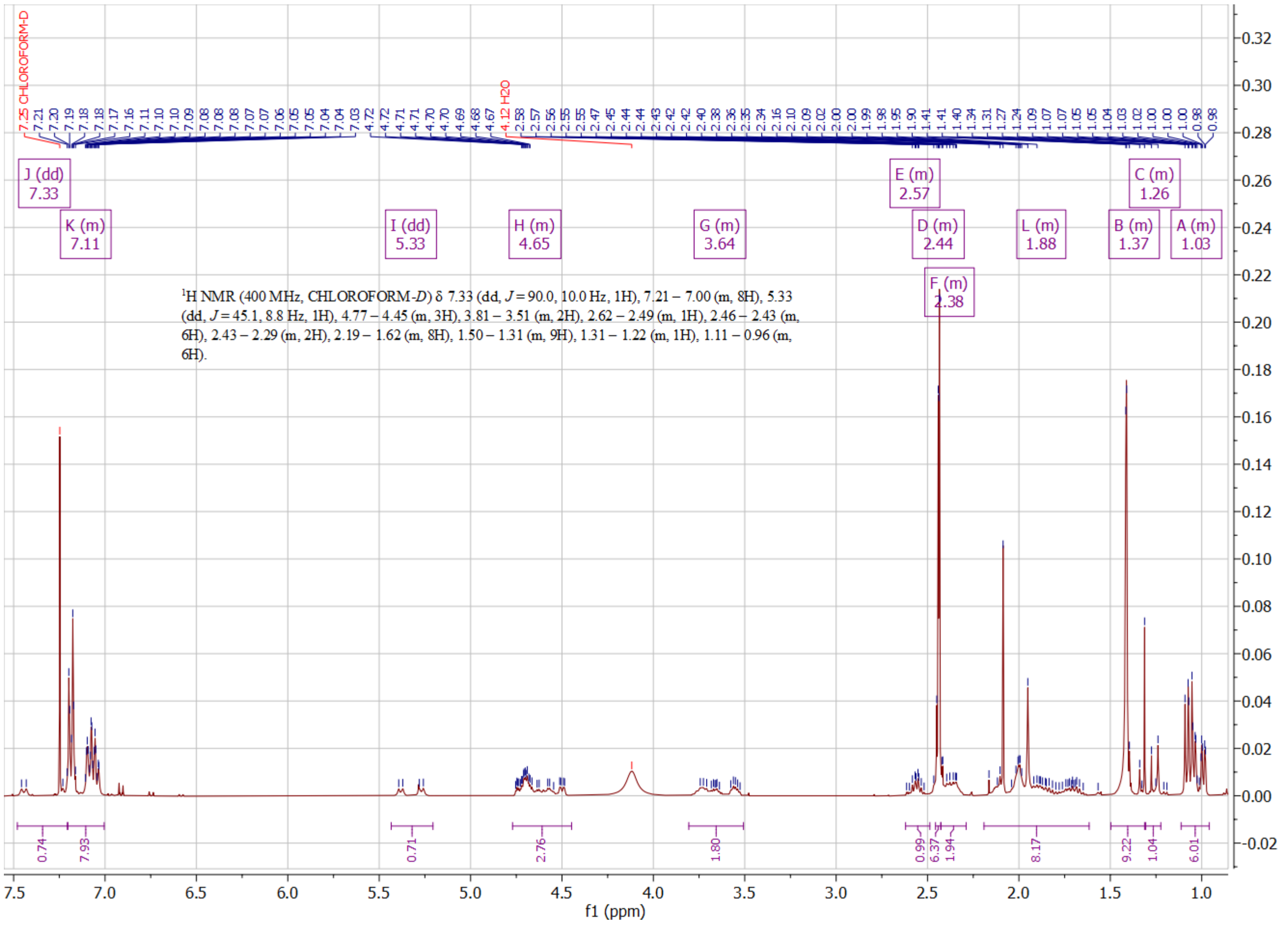

1H NMR (400 MHz, CHLOROFORM-D) δ 7.33 (dd, J = 90.0, 10.0 Hz, 1H), 7.21 – 7.00 (m, 8H), 5.33 (dd, J = 45.1, 8.8 Hz, 1H), 4.77 – 4.45 (m, 3H), 3.81 – 3.51 (m, 2H), 2.62 – 2.49 (m, 1H), 2.46 – 2.43 (m, 6H), 2.43 – 2.29 (m, 2H), 2.19 – 1.62 (m, 8H), 1.50 – 1.31 (m, 9H), 1.31 – 1.22 (m, 1H), 1.11 – 0.96 (m, 6H).

(76) Boc-D-Met-L-Pro-Val^P(OPh-4-SCH$_3$)$_2$

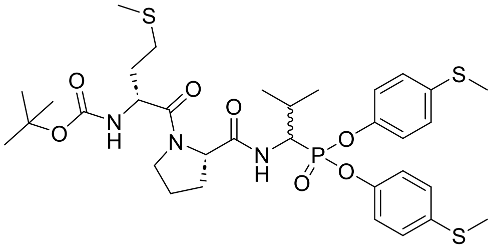

Name: *tert*-butyl ((2R)-1-((2S)-2-((1-(bis(4-(methylthio)phenoxy)phosphoryl)-2-methylpropyl)carbamoyl)pyrrolidin-1-yl)-4-(methylthio)-1-oxobutan-2-yl)carbamate

^{31}P NMR (162 MHz, Chloroform-d) δ 18.46 (s, 59%), 18.15 (s, 41%).

^{1}H NMR (400 MHz, Chloroform-d) δ 7.53 (dd, J = 39.7, 10.0 Hz, 1H, NH), 7.22 – 7.10 (m, 6H, 6xArH), 7.10 – 7.00 (m, 2H, 2xArH), 5.35 (dd, J = 30.3, 8.5 Hz, 1H, NH), 4.73 – 4.59 (m, 2H, CHP, CH), 4.59 – 4.49 (m, 1H, CH), 3.89 – 3.68 (m, 1H, 0.5xCH$_2$), 3.66 – 3.46 (m, 1H, 0.5xCH$_2$), 2.59 – 2.50 (m, 2H, CH$_2$), 2.43 (d, J = 3.7 Hz, 6H, 2xSCH$_3$), 2.41 – 2.21 (m, 2H, 0.5xCH$_2$, CH), 2.09 (d, J = 3.1 Hz, 3H, CH$_3$), 2.04 – 1.77 (m, 5H, 2.5xCH$_2$), 1.36 (d, J = 19.1 Hz, 9H, Boc), 1.10 – 0.94 (m, 6H, 2xCH$_3$).

HRMS: $C_{33}H_{48}N_3O_7PS_3$ $[M+Na]^+$ calc. 748.2290 found 748.2278

HPLC: purity >99%; retention time 18.687 min

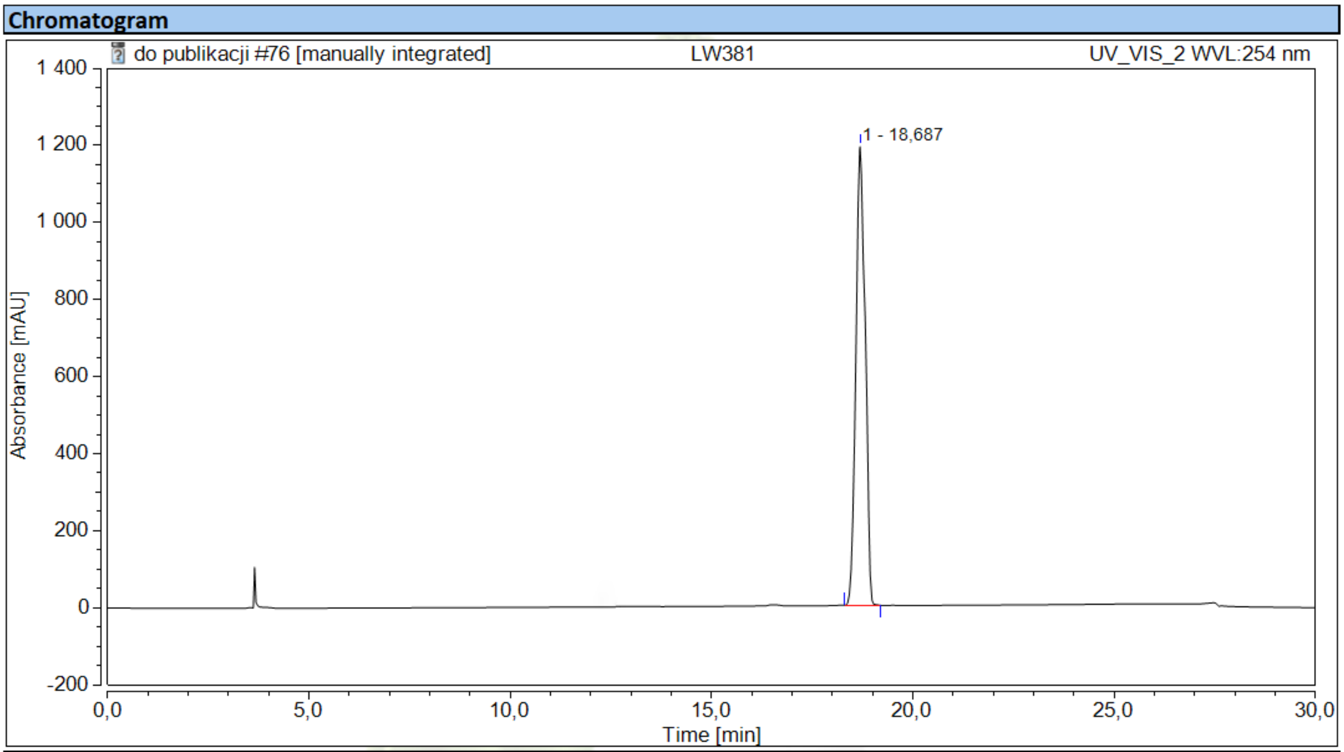

Integration Results

No.	Peak Name	Retention Time min	Area mAU*min	Height mAU	Relative Area %	Relative Height %	Amount n.a.
1		18,687	319,783	1190,076	100,00	100,00	n.a.
**Total:**			**319,783**	**1190,076**	**100,00**	**100,00**	

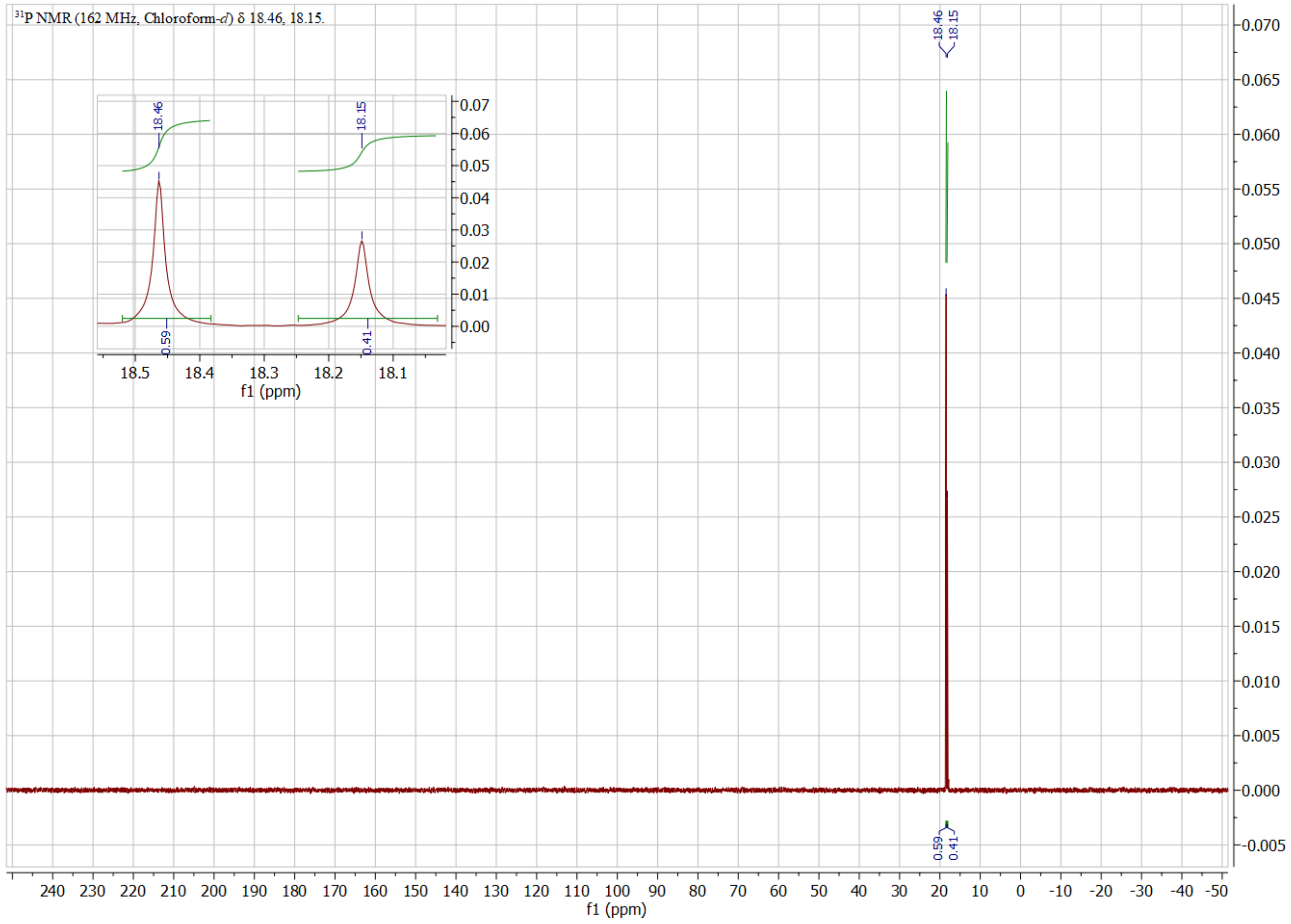

^{31}P NMR (162 MHz, Chloroform-*d*) δ 18.46, 18.15.
18.46
18.15
0.59
0.41
f1 (ppm)

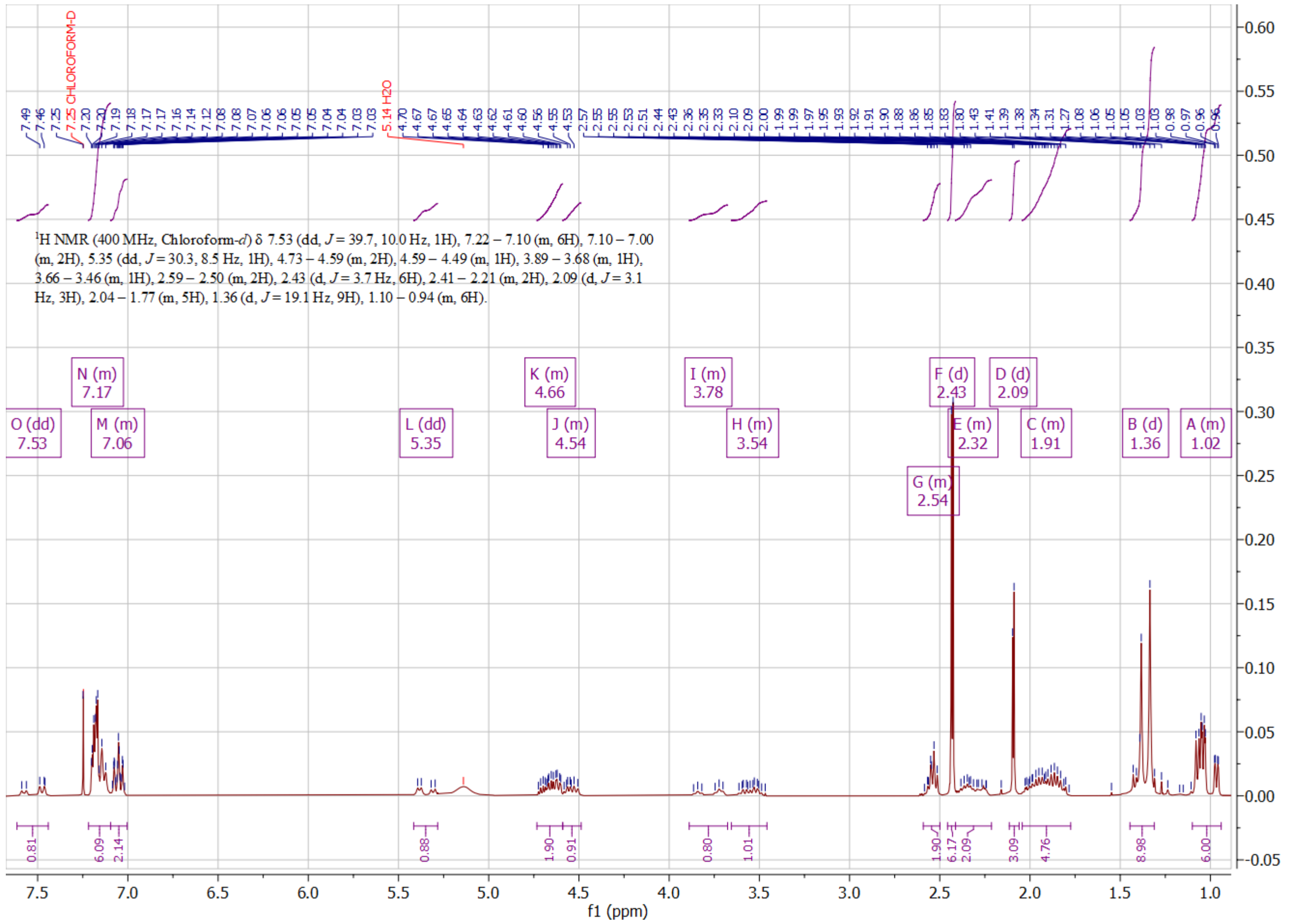

CHLOROFORM-D
H2O
^{1}H NMR (400 MHz, Chloroform-*d*) δ 7.53 (dd, *J* = 39.7, 10.0 Hz, 1H), 7.22 – 7.10 (m, 6H), 7.10 – 7.00 (m, 2H), 5.35 (dd, *J* = 30.3, 8.5 Hz, 1H), 4.73 – 4.59 (m, 2H), 4.59 – 4.49 (m, 1H), 3.89 – 3.68 (m, 1H), 3.66 – 3.46 (m, 1H), 2.59 – 2.50 (m, 2H), 2.43 (d, *J* = 3.7 Hz, 6H), 2.41 – 2.21 (m, 2H), 2.09 (d, *J* = 3.1 Hz, 3H), 2.04 – 1.77 (m, 5H), 1.36 (d, *J* = 19.1 Hz, 9H), 1.10 – 0.94 (m, 6H).
O (dd) 7.53
N (m) 7.17
M (m) 7.06
L (dd) 5.35
K (m) 4.66
J (m) 4.54
I (m) 3.78
H (m) 3.54
G (m) 2.54
F (d) 2.43
E (m) 2.32
D (d) 2.09
C (m) 1.91
B (d) 1.36
A (m) 1.02
f1 (ppm)

(77) Boc-L-Met(O)-L-Pro-Val^{P}(OPh-4-SCH$_3$)$_2$

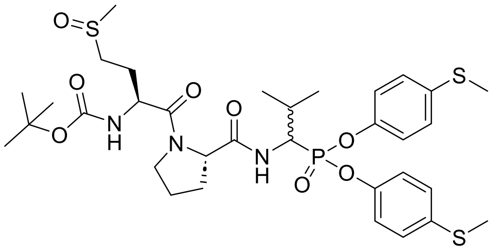

Name: *tert*-butyl ((2S)-1-((2S)-2-((1-(bis(4-(methylthio)phenoxy)phosphoryl)-2-methylpropyl)carbamoyl)pyrrolidin-1-yl)-4-(methylsulfinyl)-1-oxobutan-2-yl)carbamate

^{31}P NMR (162 MHz, Chloroform-d) δ 18.37 (s, 27%), 18.16 (s, 19%), 17.94 (d, J = 2.2 Hz, 52%).

^{1}H NMR (400 MHz, Chloroform-d) δ 7.51 (dd, J = 33.7, 10.3 Hz, 1H, NH), 7.22 – 7.14 (m, 4H, 4xArH), 7.12 – 6.96 (m, 4H, 4xArH), 5.58 – 5.35 (m, 1H, NH), 4.77 – 4.44 (m, 3H, CHP, 2xCH), 3.77 – 3.38 (m, 2H, CH_2), 2.93 – 2.68 (m, 2H, CH_2), 2.69 – 2.51 (m, 3H, CH_3), 2.44 (d, J = 2.5 Hz, 6H, $2xSCH_3$), 2.42 – 2.20 (m, 2H, $0.5xCH_2$, CH), 2.20 – 1.77 (m, 5H, $2.5xCH_2$), 1.41 (s, 9H, Boc), 1.13 – 0.98 (m, 6H, $2xCH_3$).

HRMS: $C_{33}H_{48}N_3O_8PS_3$ $[M+Na]^+$ calc. 764.2239 found 764.2211

HPLC: purity >99%; retention time 16.127 and 16.370 min

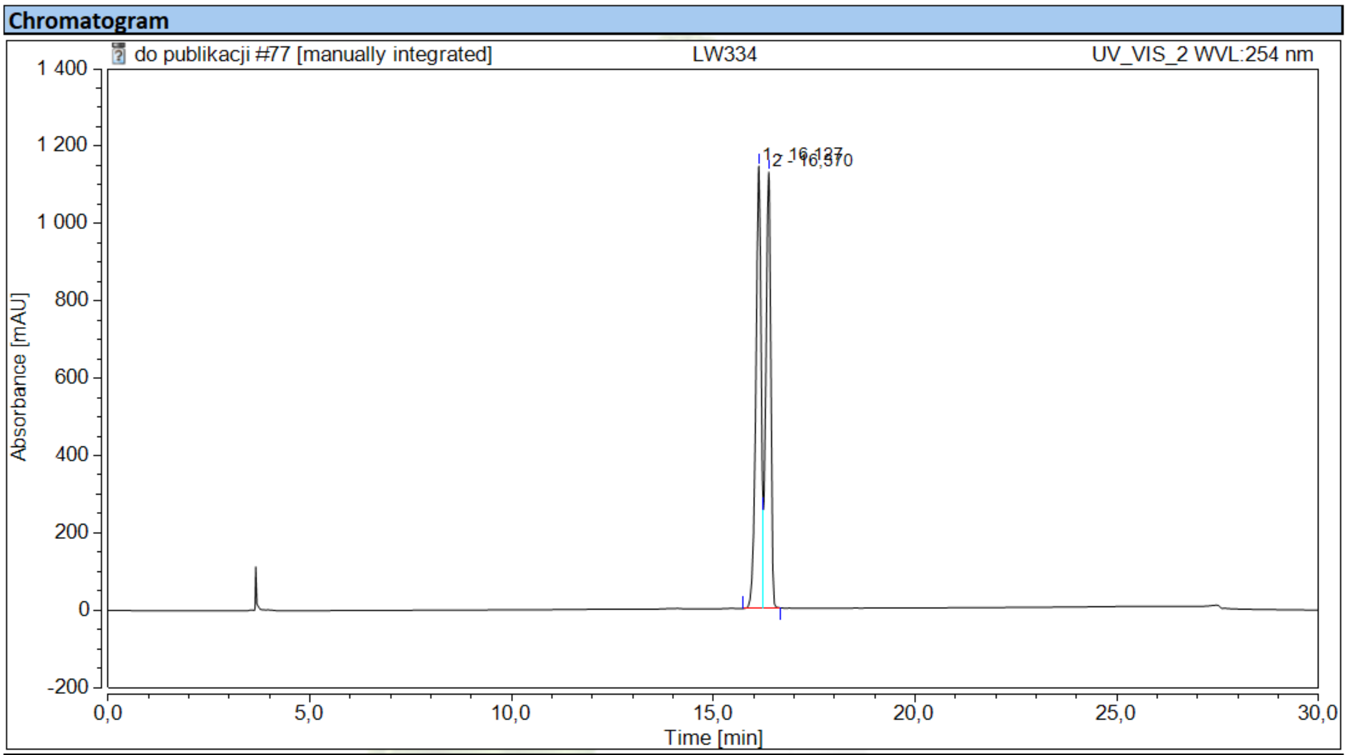

Integration Results

No.	Peak Name	Retention Time min	Area mAU*min	Height mAU	Relative Area %	Relative Height %	Amount n.a.
1		16,127	173,711	1143,549	52,21	50,35	n.a.
2		16,370	158,999	1127,545	47,79	49,65	n.a.
**Total:**			**332,711**	**2271,093**	**100,00**	**100,00**	

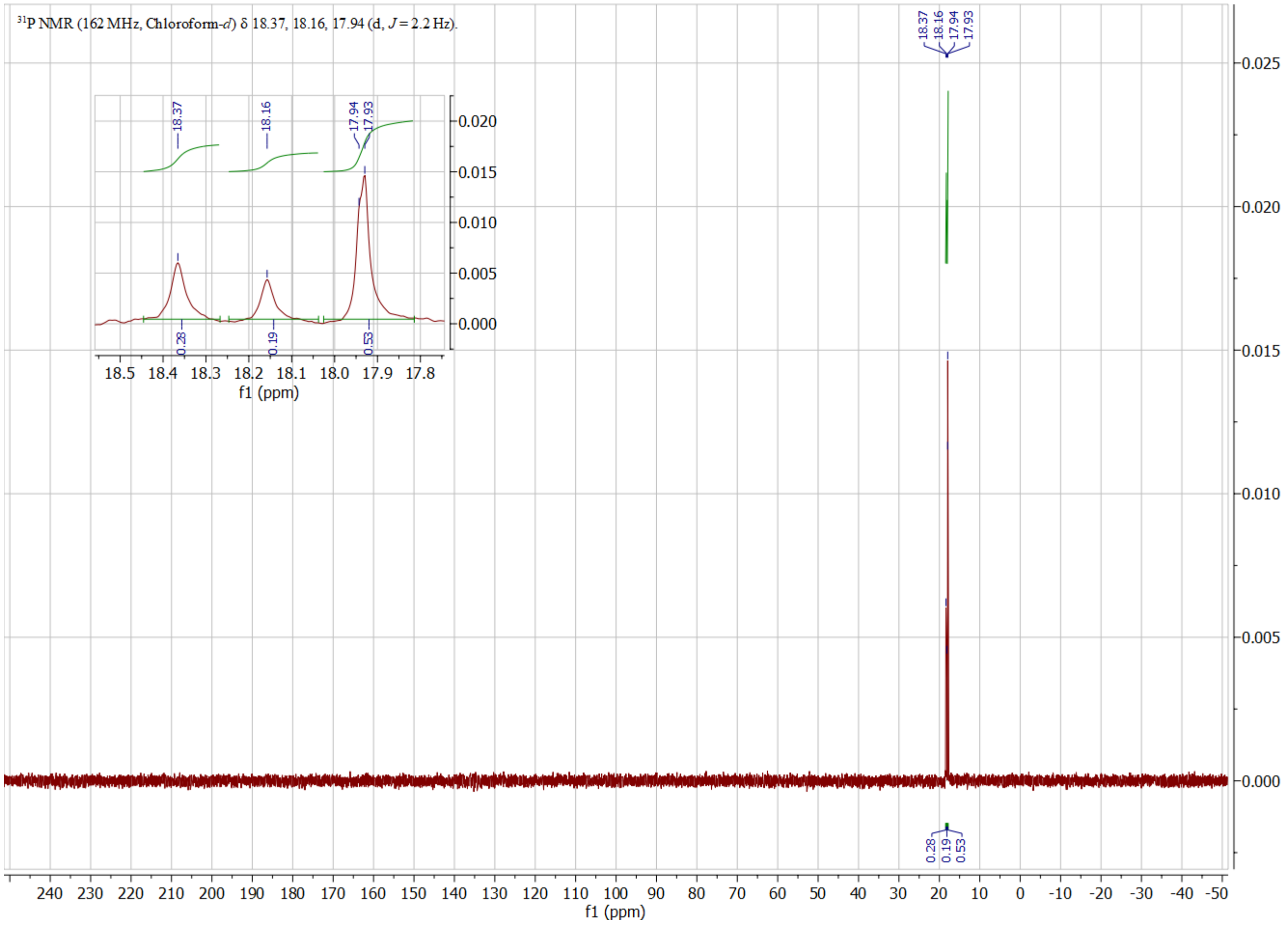

^{31}P NMR (162 MHz, Chloroform-*d*) δ 18.37, 18.16, 17.94 (d, *J* = 2.2 Hz).
f1 (ppm)

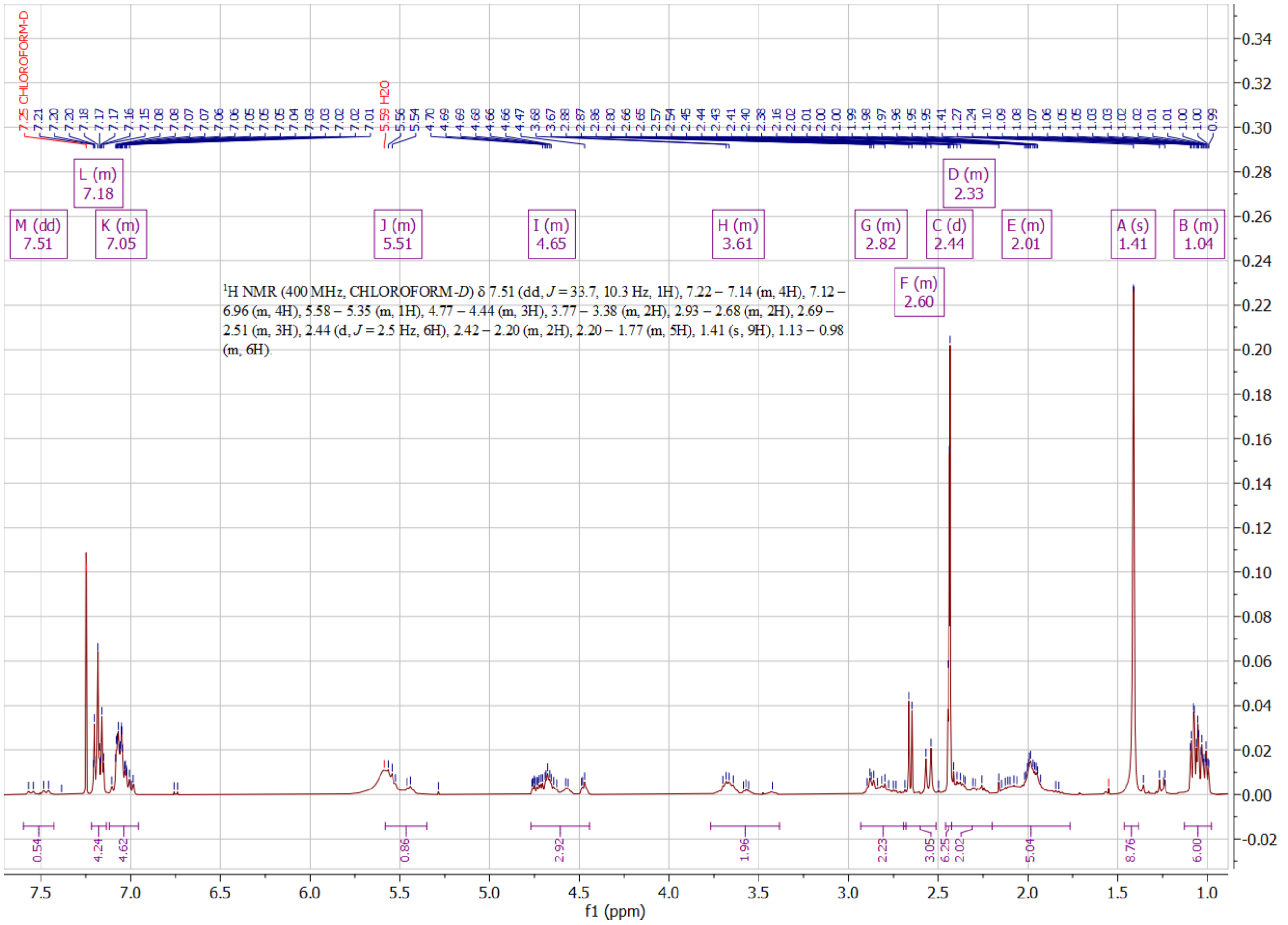

^{1}H NMR (400 MHz, CHLOROFORM-*D*) δ 7.51 (dd, *J* = 33.7, 10.3 Hz, 1H), 7.22 – 7.14 (m, 4H), 7.12 – 6.96 (m, 4H), 5.58 – 5.35 (m, 1H), 4.77 – 4.44 (m, 3H), 3.77 – 3.38 (m, 2H), 2.93 – 2.68 (m, 2H), 2.69 – 2.51 (m, 3H), 2.44 (d, *J* = 2.5 Hz, 6H), 2.42 – 2.20 (m, 2H), 2.20 – 1.77 (m, 5H), 1.41 (s, 9H), 1.13 – 0.98 (m, 6H).
f1 (ppm)

(78) Boc-L-Met(O)$_2$-L-Pro- Val^P(OPh-4-SCH$_3$)$_2$

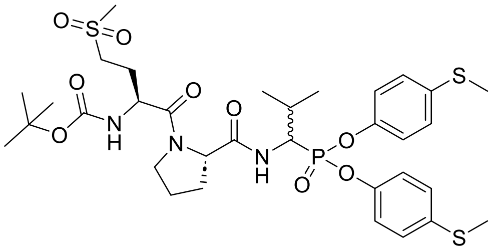

Name: *tert*-butyl ((2S)-1-((2S)-2-((1-(bis(4-(methylthio)phenoxy)phosphoryl)-2-methylpropyl)carbamoyl)pyrrolidin-1-yl)-4-(methylsulfonyl)-1-oxobutan-2-yl)carbamate

^{31}P NMR (162 MHz, Chloroform-d) δ 18.19 (s, 42%), 17.79 (s, 58%).

^{1}H NMR (400 MHz, Chloroform-d) δ 7.29 (dd, J = 142.1, 10.3 Hz, 1H, NH), 7.23 – 7.14 (m, 4H, 4xArH), 7.09 – 6.95 (m, 4H, 4xArH), 5.51 (dd, J = 25.8, 8.4 Hz, 1H, NH), 4.85 – 4.44 (m, 3H, CHP, 2xCH), 3.77 – 3.51 (m, 2H, CH$_2$), 3.19 – 2.99 (m, 2H, CH$_2$), 2.87 (d, J = 46.8 Hz, 3H, CH$_3$), 2.47 – 2.42 (m, 6H, 2xSCH$_3$), 2.43 – 2.11 (m, 3H, CH$_2$, CH), 2.11 – 1.79 (m, 4H, 2xCH$_2$), 1.41 (s, 9H, Boc), 1.11 – 0.98 (m, 6H, 2xCH$_3$).

HRMS: $C_{33}H_{48}N_3O_9PS_3$ $[M+H]^+$ 758.2369 found 758.2386.

HPLC: purity >99%; retention time 16.750 and 17.007 min

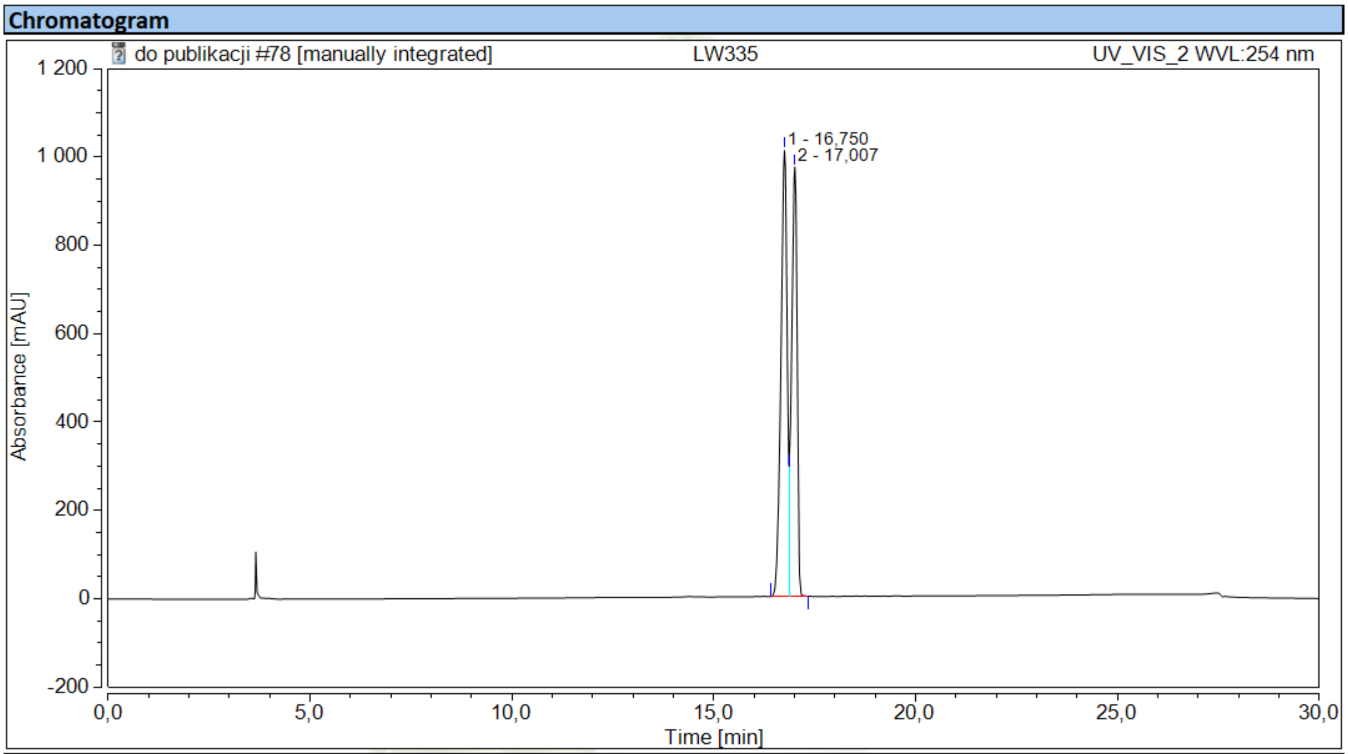

Integration Results

No.	Peak Name	Retention Time<br>min	Area<br>mAU*min	Height<br>mAU	Relative Area<br>%	Relative Height<br>%	Amount<br>n.a.
1		16,750	177,842	1009,523	53,38	50,95	n.a.
2		17,007	155,310	971,858	46,62	49,05	n.a.
**Total:**			**333,152**	**1981,381**	**100,00**	**100,00**	

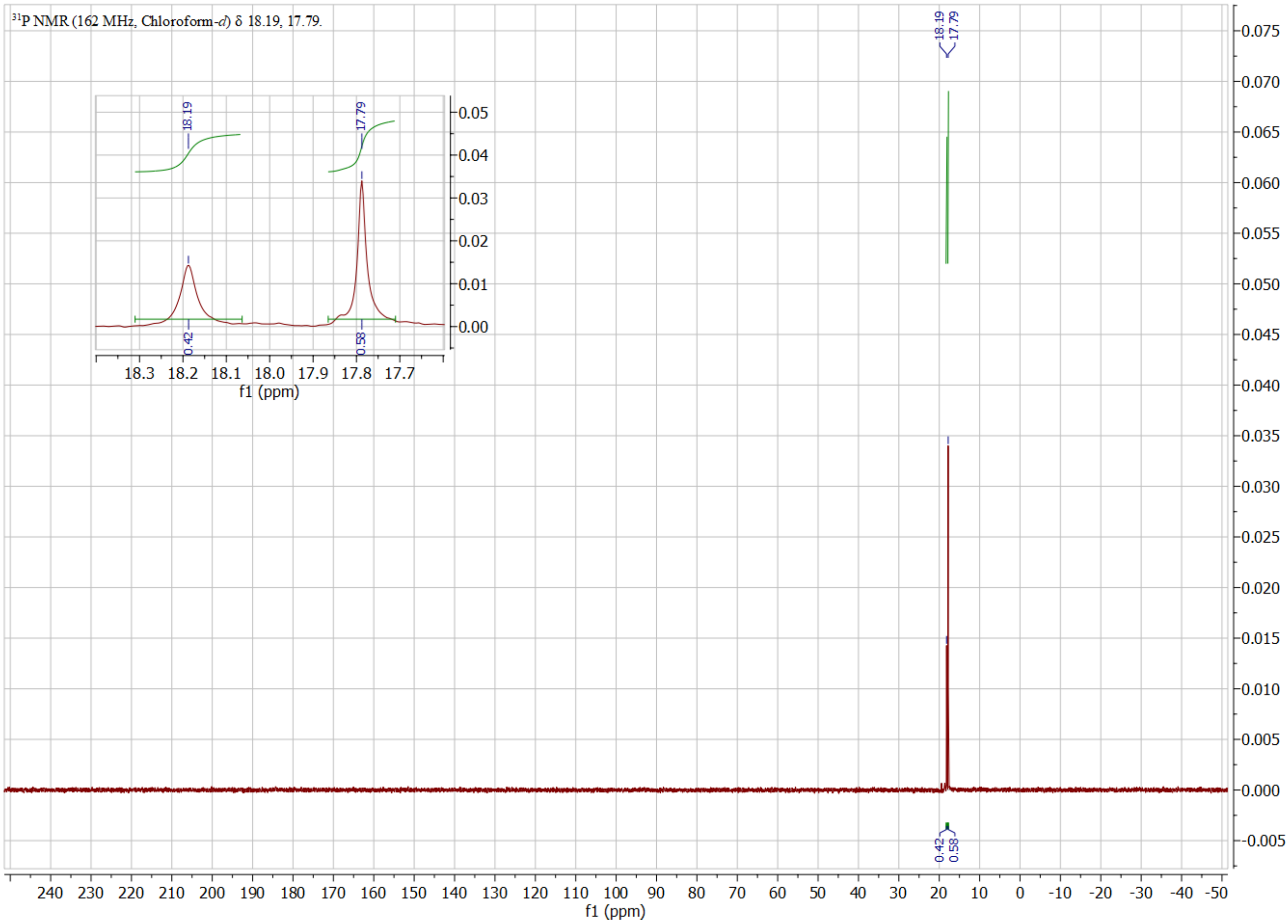
31P NMR (162 MHz, Chloroform-d) δ 18.19, 17.79.
f1 (ppm)

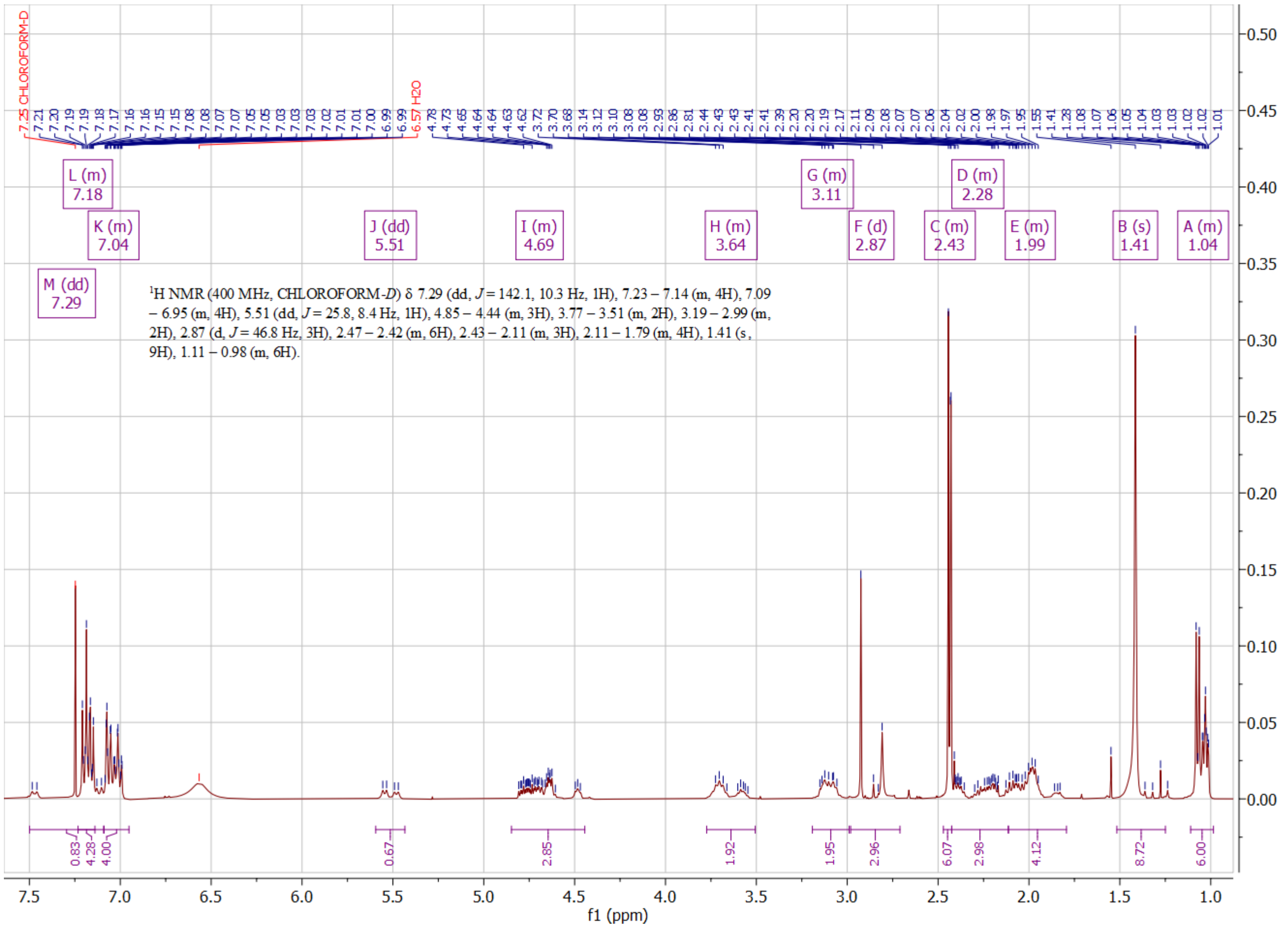
1H NMR (400 MHz, CHLOROFORM-D) δ 7.29 (dd, J = 142.1, 10.3 Hz, 1H), 7.23 – 7.14 (m, 4H), 7.09 – 6.95 (m, 4H), 5.51 (dd, J = 25.8, 8.4 Hz, 1H), 4.85 – 4.44 (m, 3H), 3.77 – 3.51 (m, 2H), 3.19 – 2.99 (m, 2H), 2.87 (d, J = 46.8 Hz, 3H), 2.47 – 2.42 (m, 6H), 2.43 – 2.11 (m, 3H), 2.11 – 1.79 (m, 4H), 1.41 (s, 9H), 1.11 – 0.98 (m, 6H).
f1 (ppm)

(79) Boc-L-Gln-L-Pro-Val^{P}(OPh-4-SCH$_3$)$_2$

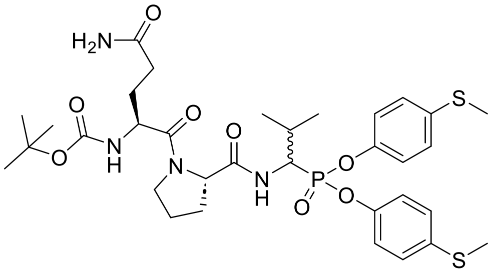

Name: *tert*-butyl ((2S)-5-amino-1-((2S)-2-((1-(bis(4-(methylthio)phenoxy)phosphoryl)-2-methylpropyl)carbamoyl)pyrrolidin-1-yl)-1,5-dioxopentan-2-yl)carbamate
^{31}P NMR (162 MHz, Chloroform-d) δ 18.15 (s, 56%), 18.00 (s, 44%).
^{1}H NMR (400 MHz, Chloroform-d) δ 7.52 (dd, J = 171.2, 10.2 Hz, 1H, NH), 7.23 – 6.44 (m, 10H, 8xArH, NH$_2$), 5.46 (dd, J = 66.2, 8.2 Hz, 1H, NH), 4.84 – 4.38 (m, 3H, CHP, 2xCH), 3.74 – 3.33 (m, 2H, CH$_2$), 2.52 – 2.35 (m, 8H, 2xSCH$_3$, CH$_2$), 2.35 – 2.20 (m, 1H, CH), 2.15 – 1.82 (m, 6H, 3xCH$_2$), 1.41 (d, J = 5.7 Hz, 9H, Boc), 1.15 – 0.99 (m, 6H, 2xCH$_3$).
HRMS: $C_{33}H_{47}N_4O_8PS_2$ [M+Na]$^+$ calc. 745.2471 found 745.2467
HPLC: purity >98%; retention time 15.847 and 16.103 min

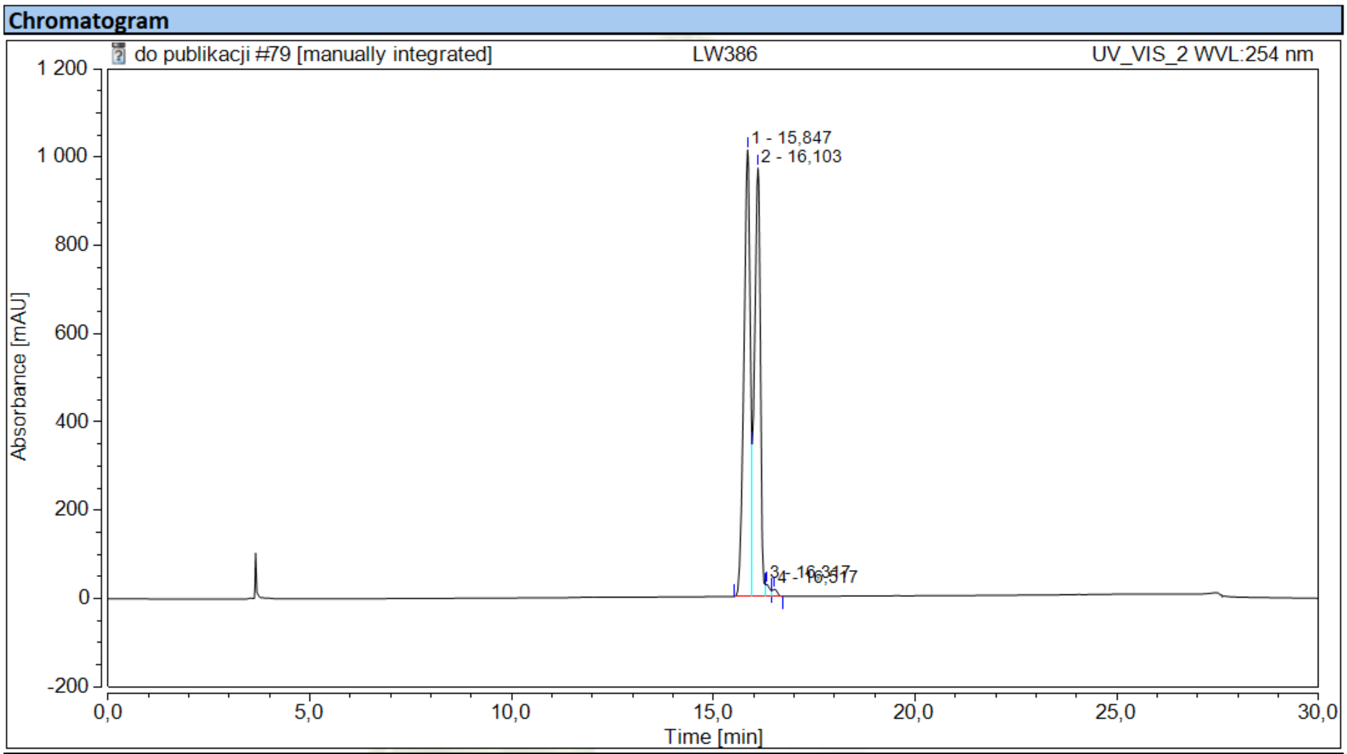

Integration Results

No.	Peak Name	Retention Time min	Area mAU*min	Height mAU	Relative Area %	Relative Height %	Amount n.a.
1		15,847	182,632	1011,941	52,23	49,95	n.a.
2		16,103	161,153	970,643	46,09	47,91	n.a.
3		16,317	3,500	27,406	1,00	1,35	n.a.
4		16,517	2,360	16,113	0,67	0,80	n.a.
**Total:**			**349,645**	**2026,103**	**100,00**	**100,00**	

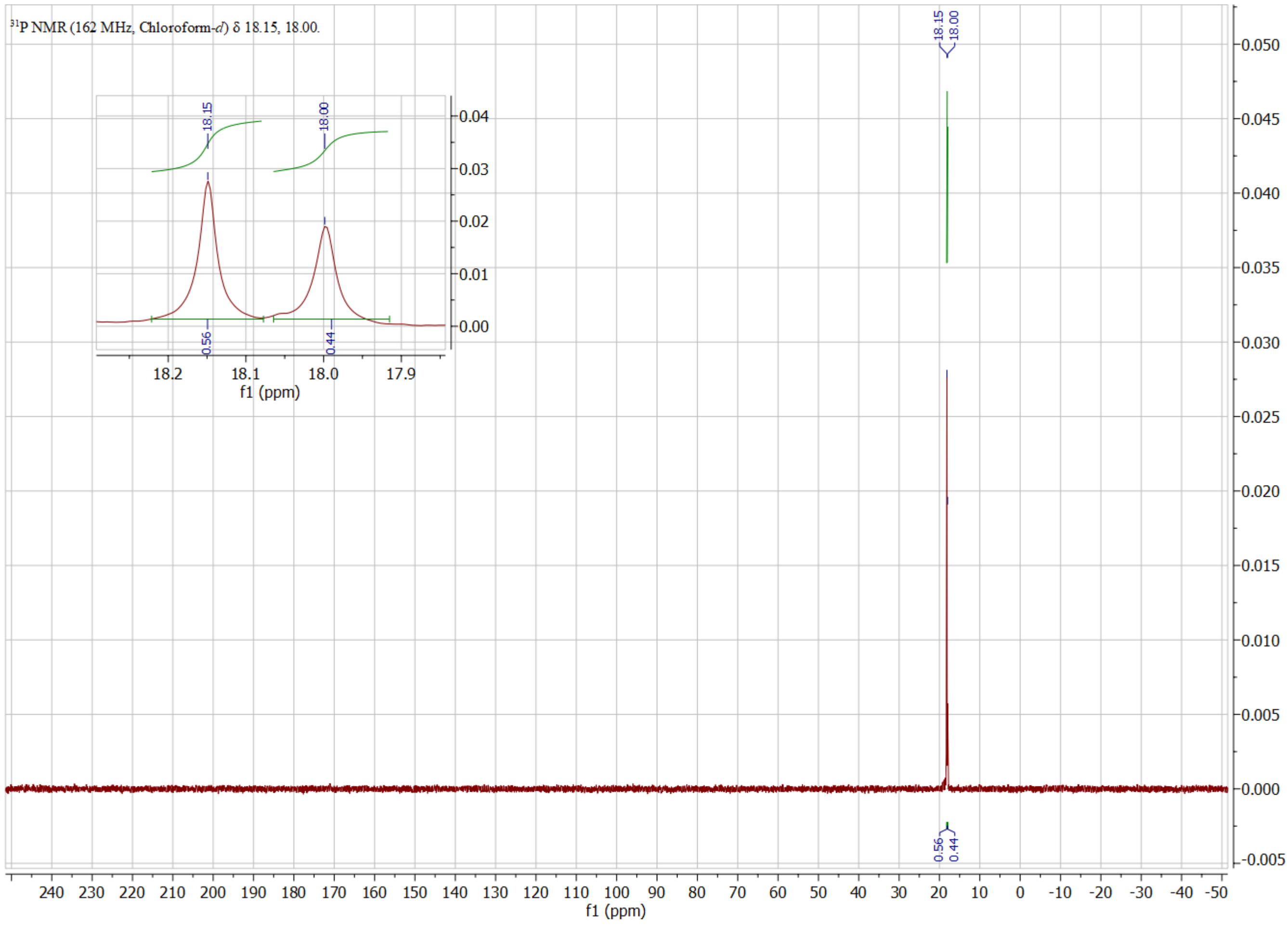
³¹P NMR (162 MHz, Chloroform-d) δ 18.15, 18.00.
18.15
18.00
0.56
0.44
f1 (ppm)

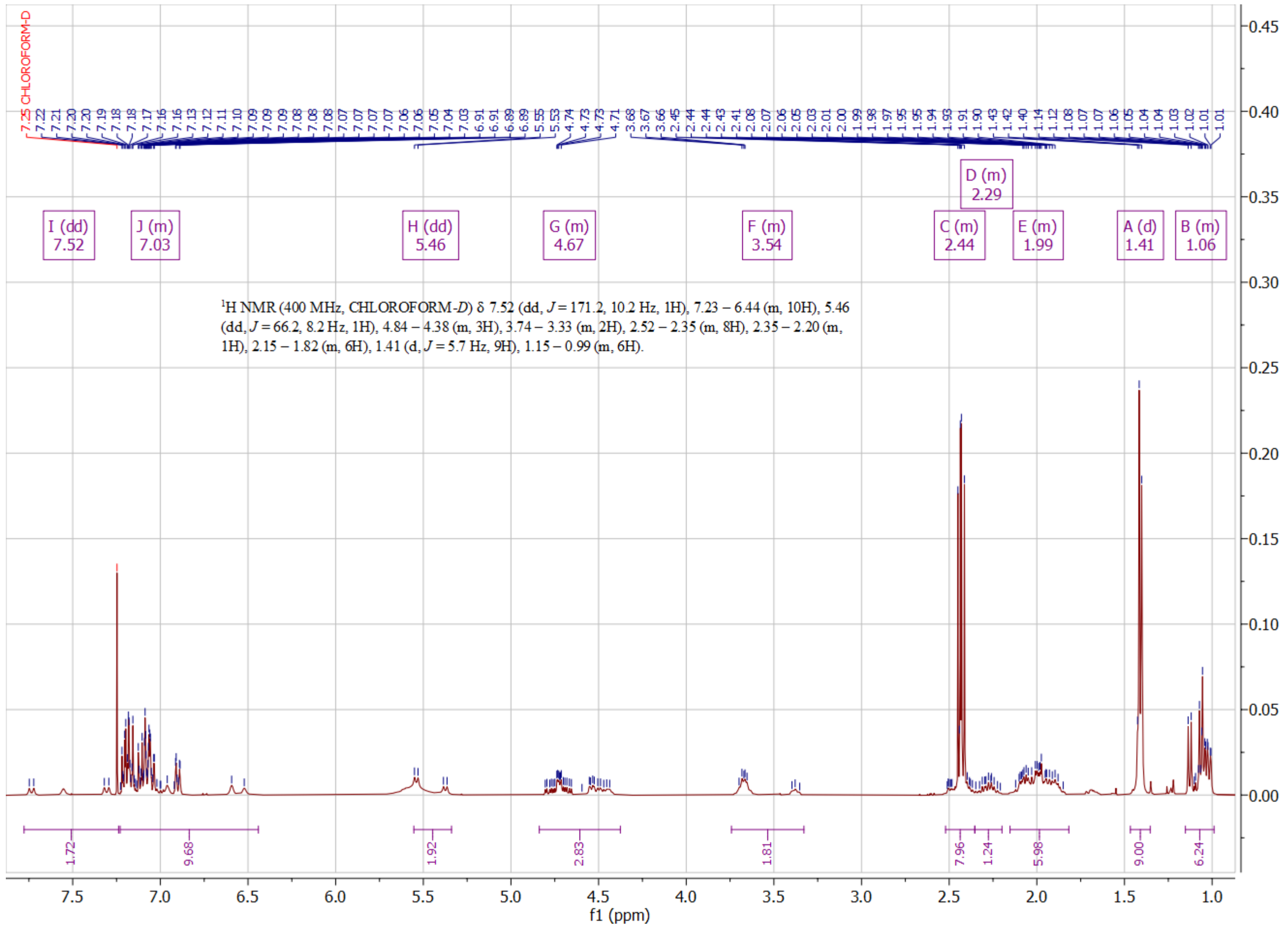
7.25 CHLOROFORM-D
I (dd) 7.52
J (m) 7.03
H (dd) 5.46
G (m) 4.67
F (m) 3.54
D (m) 2.29
C (m) 2.44
E (m) 1.99
A (d) 1.41
B (m) 1.06
¹H NMR (400 MHz, CHLOROFORM-D) δ 7.52 (dd, J = 171.2, 10.2 Hz, 1H), 7.23 – 6.44 (m, 10H), 5.46 (dd, J = 66.2, 8.2 Hz, 1H), 4.84 – 4.38 (m, 3H), 3.74 – 3.33 (m, 2H), 2.52 – 2.35 (m, 8H), 2.35 – 2.20 (m, 1H), 2.15 – 1.82 (m, 6H), 1.41 (d, J = 5.7 Hz, 9H), 1.15 – 0.99 (m, 6H).
1.72
9.68
1.92
2.83
1.81
7.96
1.24
5.98
9.00
6.24
f1 (ppm)

(80) Boc-D-Gln-L-Pro- Val^{P}(OPh-4-SCH$_3$)$_2$

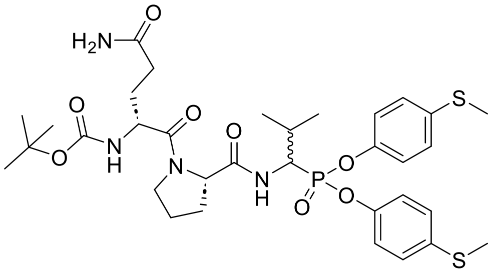

Name: *tert*-butyl ((2R)-5-amino-1-((2S)-2-((1-(bis(4-(methylthio)phenoxy)phosphoryl)-2-methylpropyl)carbamoyl)pyrrolidin-1-yl)-1,5-dioxopentan-2-yl)carbamate

^{31}P NMR (162 MHz, Chloroform-d) δ 18.38 (s, 53%), 18.18 (s, 47%).

^{1}H NMR (400 MHz, Chloroform-d) δ 7.46 (dd, J = 44.0, 10.3 Hz, 1H, NH), 7.22 – 7.04 (m, 9H, 8xArH, 0.5xNH$_2$), 6.92 – 6.05 (m, 1H, 0.5xNH$_2$), 5.64 (dd, J = 44.7, 8.2 Hz, 1H, NH), 4.73 – 4.35 (m, 3H, CHP, 2xCH), 3.74 – 3.38 (m, 2H, CH$_2$), 2.44 – 2.43 (m, 6H, 2xSCH$_3$), 2.41 – 2.29 (m, 3H, CH$_2$, CH), 2.27 – 1.73 (m, 6H, 3xCH$_2$), 1.38 (d, J = 15.4 Hz, 9H, Boc), 1.09 – 0.94 (m, 6H, 2xCH$_3$).

HRMS: $C_{33}H_{47}N_4O_8PS_2$ [M+Na]$^+$ calc. 745.2471 found 745.2472

HPLC: purity >99%; retention time 16.110 and 16.280 min

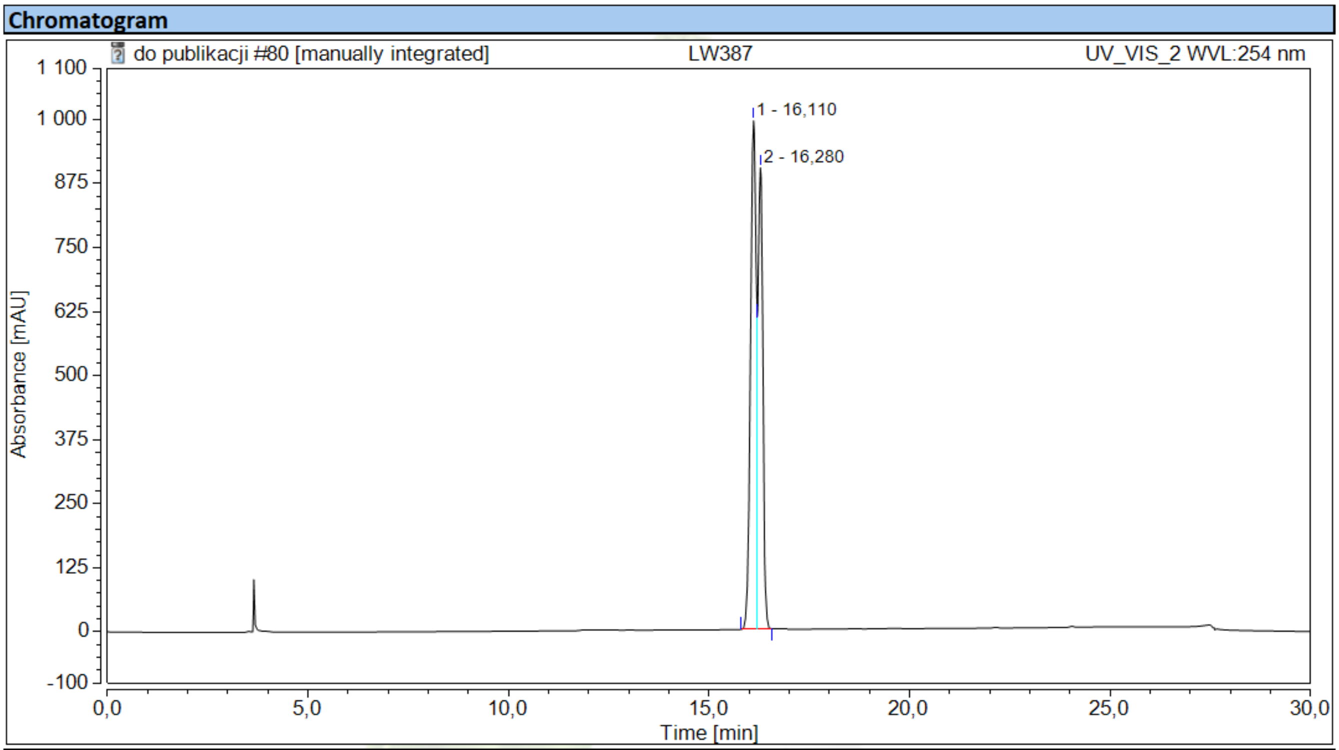

Integration Results

No.	Peak Name	Retention Time min	Area mAU*min	Height mAU	Relative Area %	Relative Height %	Amount n.a.
1		16,110	161,880	992,559	56,17	52,42	n.a.
2		16,280	126,328	900,913	43,83	47,58	n.a.
**Total:**			**288,209**	**1893,473**	**100,00**	**100,00**	

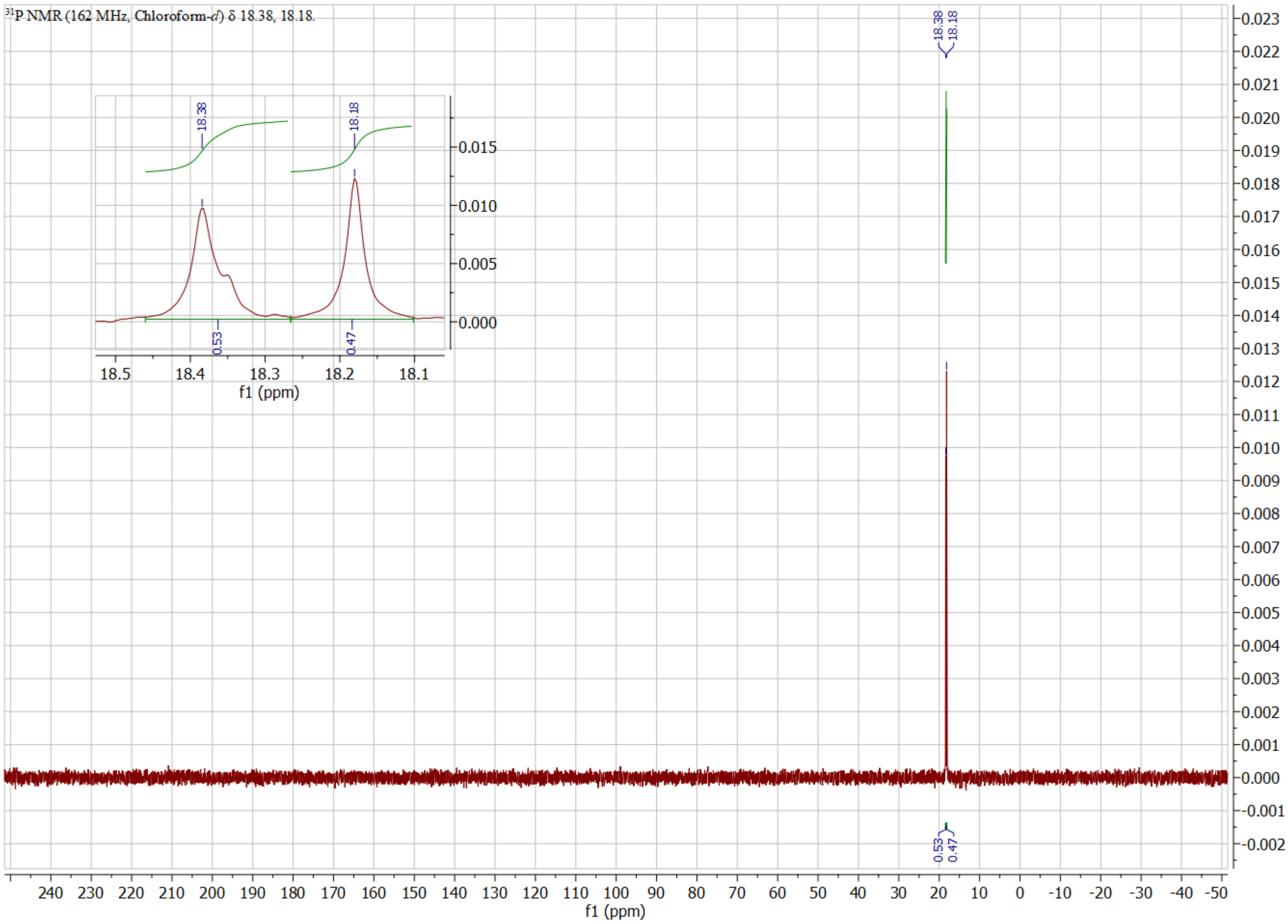
31P NMR (162 MHz, Chloroform-d) δ 18.38, 18.18.
f1 (ppm)

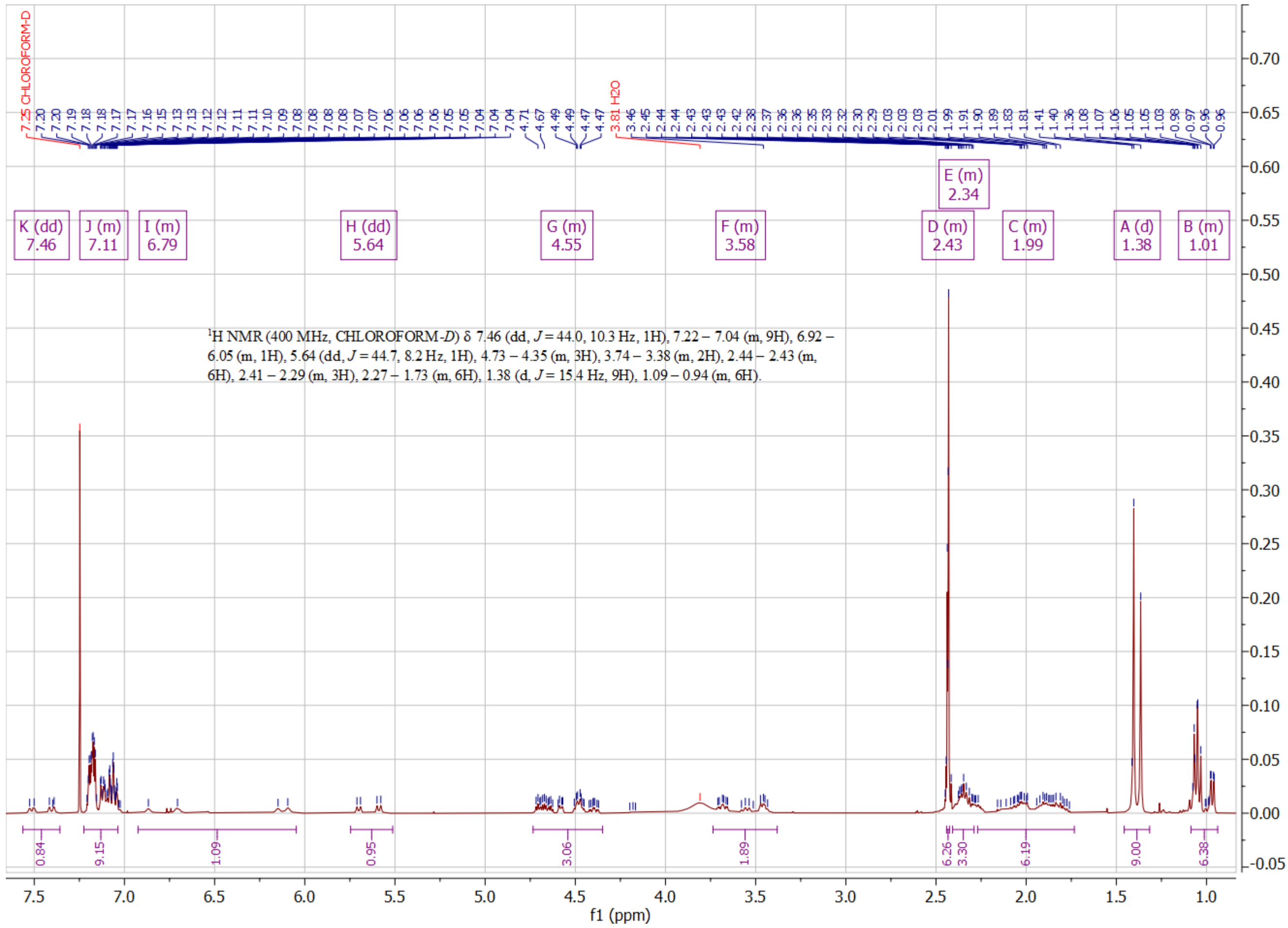
1H NMR (400 MHz, CHLOROFORM-D) δ 7.46 (dd, J = 44.0, 10.3 Hz, 1H), 7.22 – 7.04 (m, 9H), 6.92 – 6.05 (m, 1H), 5.64 (dd, J = 44.7, 8.2 Hz, 1H), 4.73 – 4.35 (m, 3H), 3.74 – 3.38 (m, 2H), 2.44 – 2.43 (m, 6H), 2.41 – 2.29 (m, 3H), 2.27 – 1.73 (m, 6H), 1.38 (d, J = 15.4 Hz, 9H), 1.09 – 0.94 (m, 6H).
f1 (ppm)

(81) Boc-L-Pro-L-Pro-Val^{P}(OPh-4-SCH$_3$)$_2$

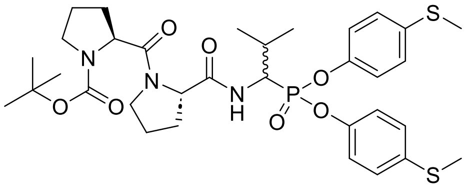

Name: *tert*-butyl (2S)-2-((2S)-2-((1-(bis(4-(methylthio)phenoxy)phosphoryl)-2-methylpropyl)carbamoyl)pyrrolidine-1-carbonyl)pyrrolidine-1-carboxylate

^{31}P NMR (162 MHz, Chloroform-d) δ 18.78 (s, 62%), 18.17 (s, 26%), 18.06 (s, 12%).

^{1}H NMR (400 MHz, Chloroform-d) δ 7.22 – 6.74 (m, 9H, 8xArH, NH), 4.83 – 4.19 (m, 3H, CHP, 2xCH), 3.79 – 3.27 (m, 4H, 2xCH$_2$), 2.50 – 2.26 (m, 7H, 2xSCH$_3$, CH), 2.25 – 1.54 (m, 8H, 4xCH$_2$), 1.47 – 1.36 (m, 9H, Boc), 1.13 – 0.95 (m, 6H, 2xCH$_3$).

HRMS: $C_{33}H_{46}N_3O_7PS_2$ [M+Na]$^+$ calc. 714.2412 found 714.2397

HPLC: purity >99%; retention time 17.957 and 18.187 min

Chromatogram

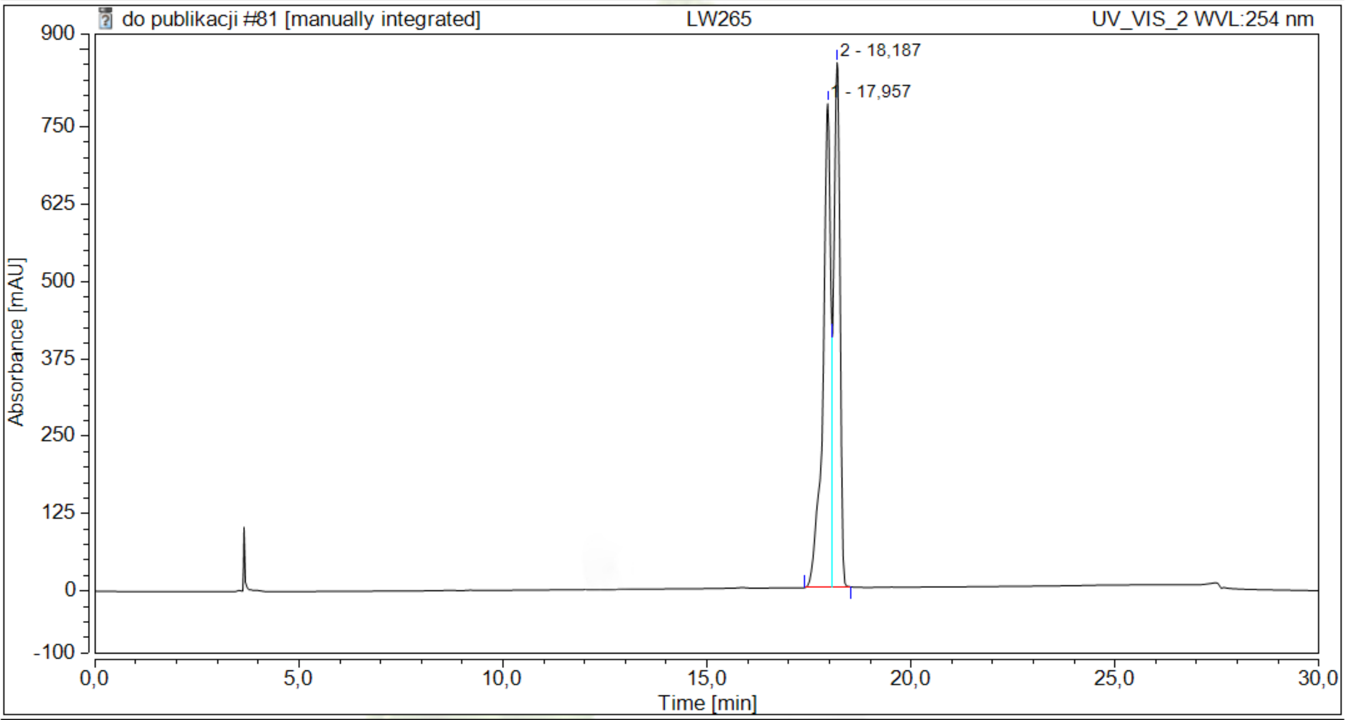

Integration Results

No.	Peak Name	Retention Time min	Area mAU*min	Height mAU	Relative Area %	Relative Height %	Amount n.a.
1		17,957	171,868	781,102	54,03	47,97	n.a.
2		18,187	146,213	847,087	45,97	52,03	n.a.
**Total:**			**318,082**	**1628,189**	**100,00**	**100,00**	

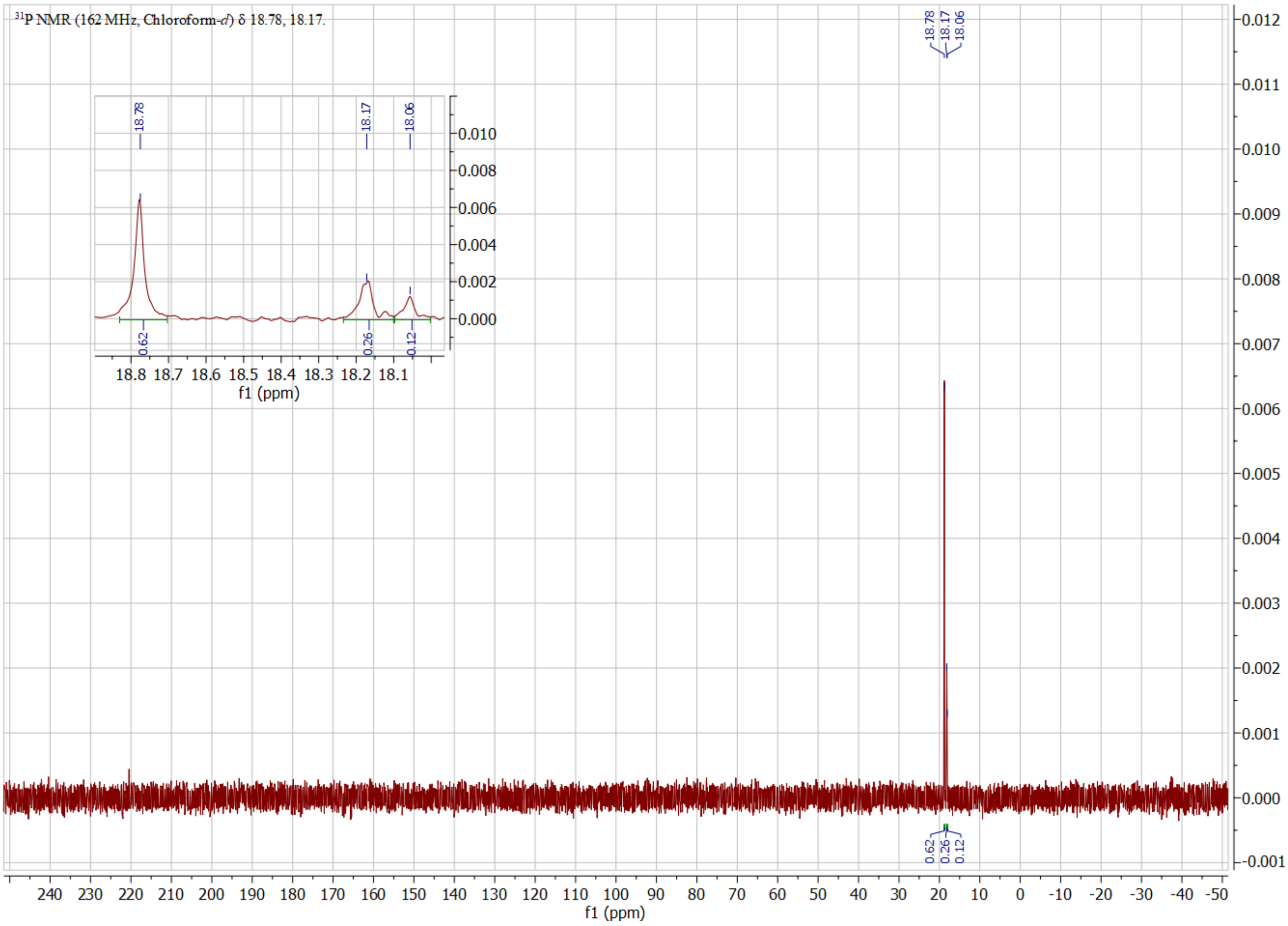
^{31}P NMR (162 MHz, Chloroform-d) δ 18.78, 18.17.
f1 (ppm)

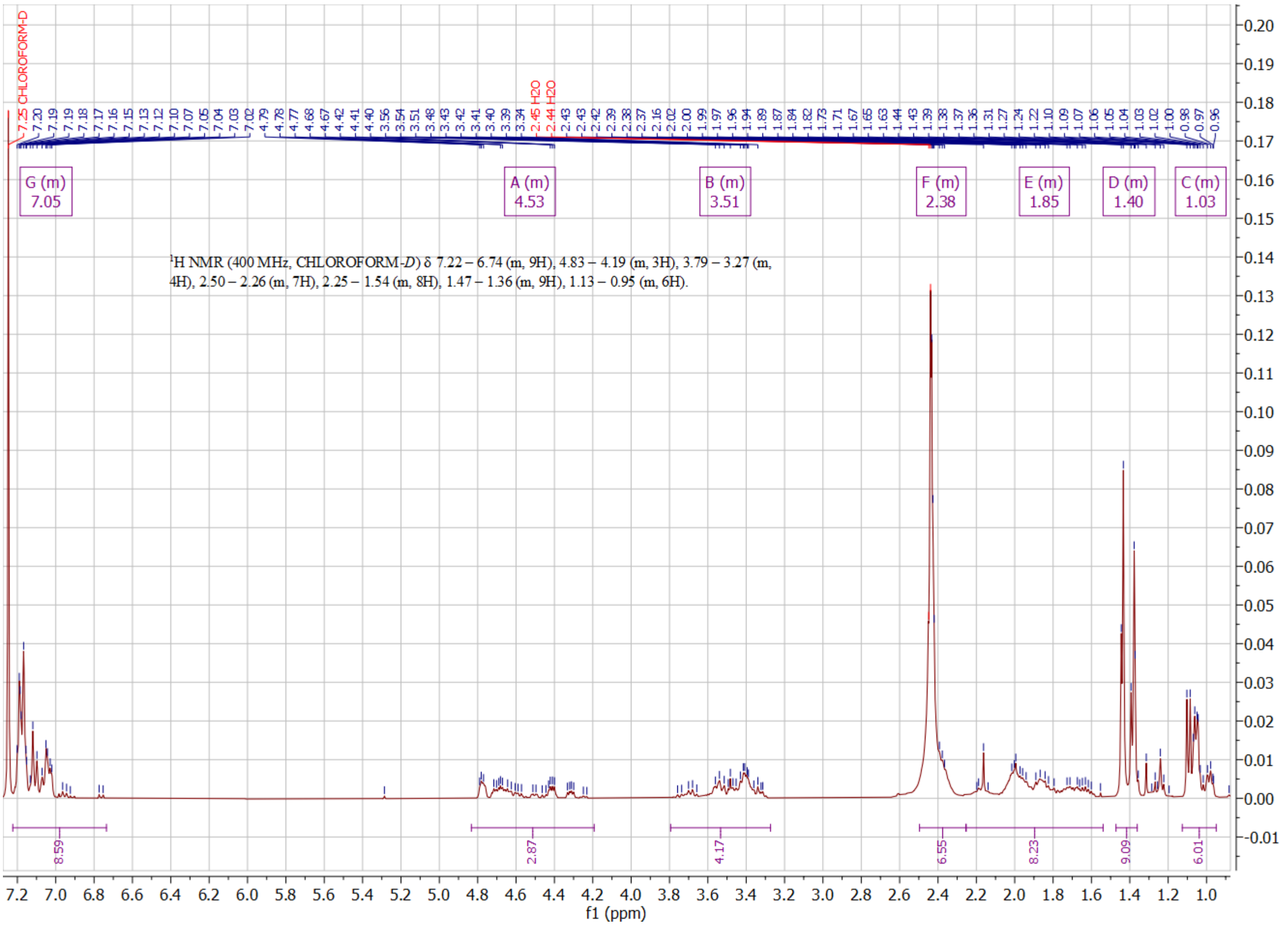
^{1}H NMR (400 MHz, CHLOROFORM-D) δ 7.22 – 6.74 (m, 9H), 4.83 – 4.19 (m, 3H), 3.79 – 3.27 (m, 4H), 2.50 – 2.26 (m, 7H), 2.25 – 1.54 (m, 8H), 1.47 – 1.36 (m, 9H), 1.13 – 0.95 (m, 6H).
G (m) 7.05
A (m) 4.53
B (m) 3.51
F (m) 2.38
E (m) 1.85
D (m) 1.40
C (m) 1.03
f1 (ppm)

(82) Boc-L-Pip-L-Pro-Val^P(OPh-4-SCH$_3$)$_2$

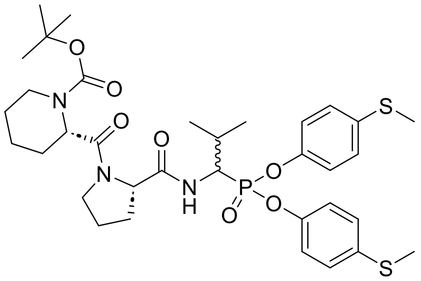

Name: *tert*-butyl (2S)-2-((2S)-2-((1-(bis(4-(methylthio)phenoxy)phosphoryl)-2-methylpropyl)carbamoyl)pyrrolidine-1-carbonyl)piperidine-1-carboxylate
^{31}P NMR (162 MHz, Chloroform-d) δ 18.58 (s, 62%), 18.15 (s, 38%).
^{1}H NMR (400 MHz, Chloroform-d) δ 7.47 (s, 1H, NH), 7.22 – 6.97 (m, 8H, 8xArH), 5.13 – 4.39 (m, 3H, CHP, 2xCH), 4.13 – 3.75 (m, 1H, 0.5xCH$_2$), 3.75 – 3.32 (m, 3H, 1.5xCH$_2$), 2.45 – 2.42 (m, 6H, 2xSCH$_3$), 2.41 – 2.22 (m, 2H, 0.5xCH$_2$, CH), 2.17 – 1.93 (m, 2H, CH$_2$), 1.92 – 1.69 (m, 2H, CH$_2$), 1.68 – 1.45 (m, 5H, 2.5xCH$_2$), 1.42 (d, J = 7.0 Hz, 9H, Boc), 1.14 – 0.95 (m, 6H, 2xCH$_3$).
HRMS: $C_{34}H_{48}N_3O_7PS_2$ [M+Na]$^+$ calc. 728.2569 found 728.2562
HPLC: purity >99%; retention time 19.393 min

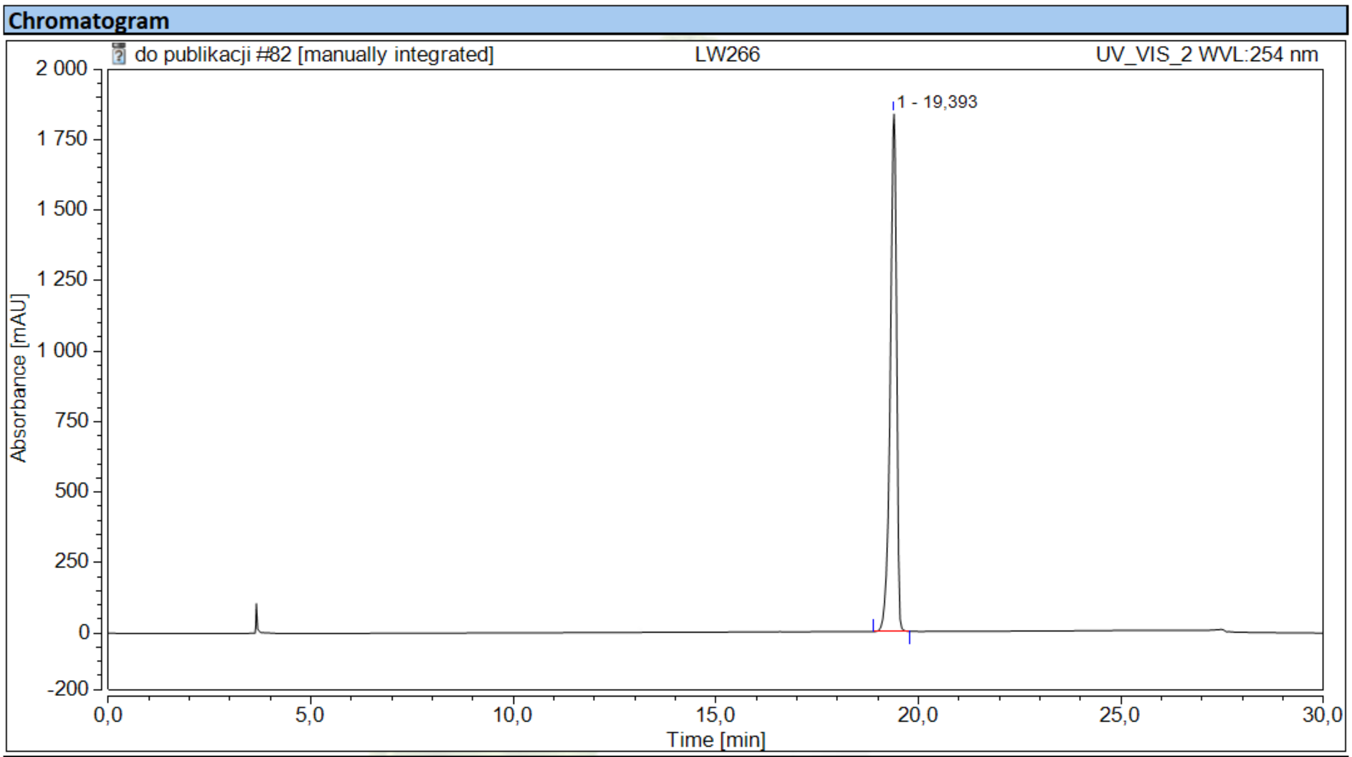

Integration Results

No.	Peak Name	Retention Time min	Area mAU*min	Height mAU	Relative Area %	Relative Height %	Amount n.a.
1		19,393	332,232	1834,347	100,00	100,00	n.a.
**Total:**			**332,232**	**1834,347**	**100,00**	**100,00**	

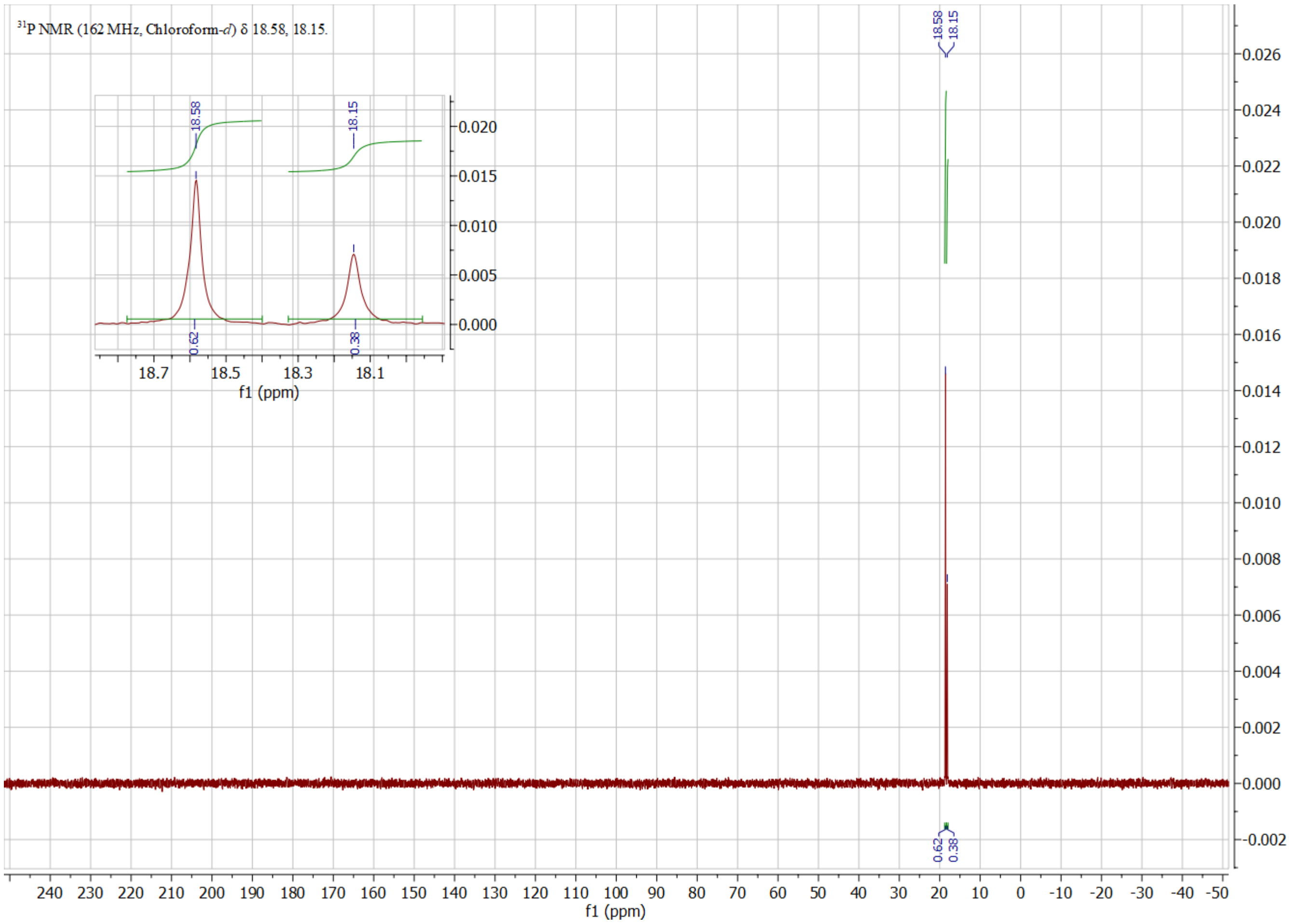
^{31}P NMR (162 MHz, Chloroform-d) δ 18.58, 18.15.
f1 (ppm)

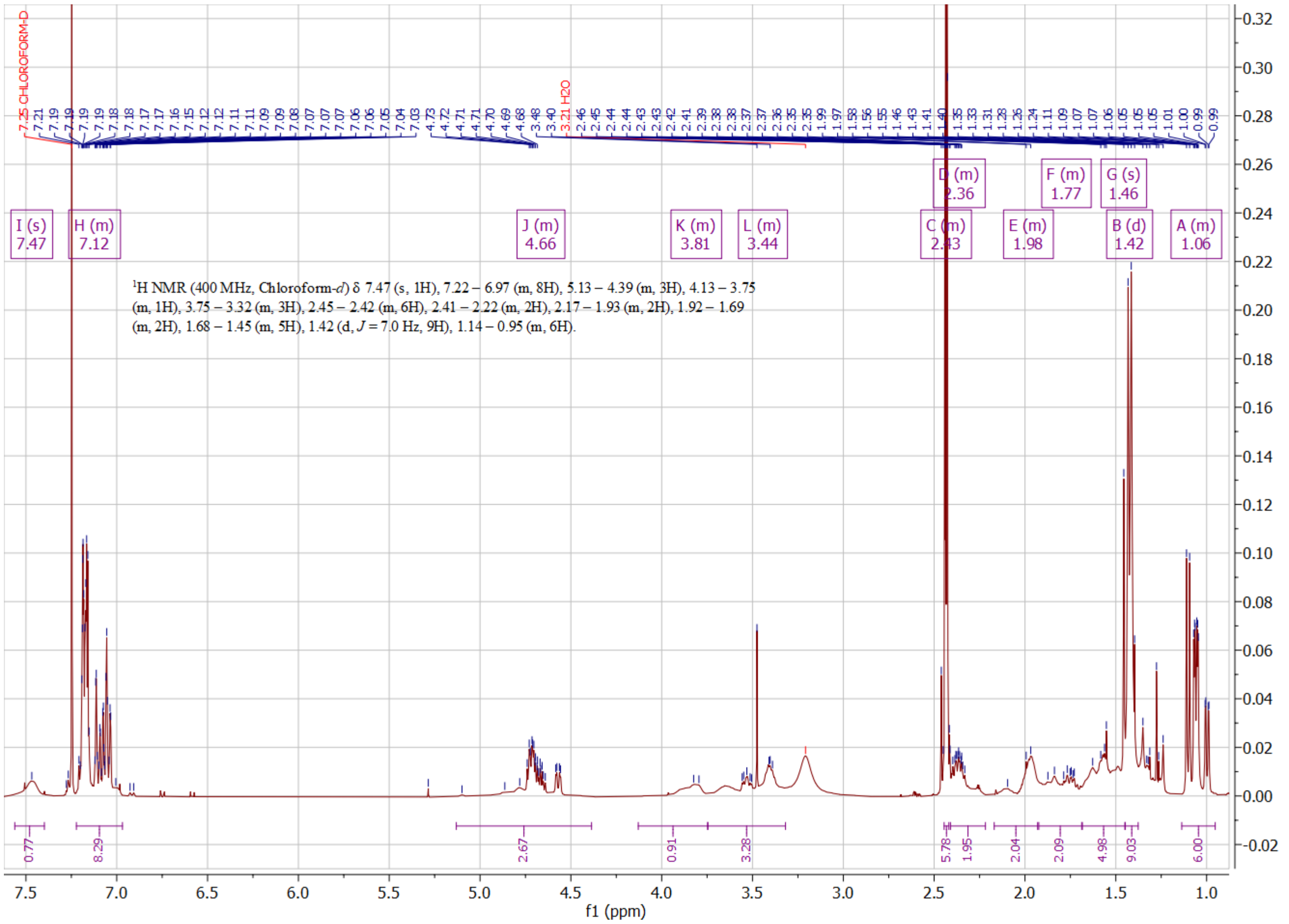
^{1}H NMR (400 MHz, Chloroform-d) δ 7.47 (s, 1H), 7.22 – 6.97 (m, 8H), 5.13 – 4.39 (m, 3H), 4.13 – 3.75 (m, 1H), 3.75 – 3.32 (m, 3H), 2.45 – 2.42 (m, 6H), 2.41 – 2.22 (m, 2H), 2.17 – 1.93 (m, 2H), 1.92 – 1.69 (m, 2H), 1.68 – 1.45 (m, 5H), 1.42 (d, J = 7.0 Hz, 9H), 1.14 – 0.95 (m, 6H).
7.25 CHLOROFORM-D
3.21 H2O
f1 (ppm)

(83) Boc-D-Pip-L-Pro- Val^{P}(OPh-4-SCH$_3$)$_2$

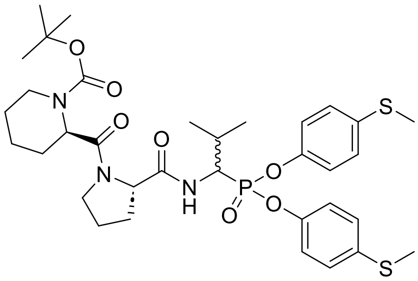

Name: *tert*-butyl (2R)-2-((2S)-2-((1-(bis(4-(methylthio)phenoxy)phosphoryl)-2-methylpropyl)carbamoyl)pyrrolidine-1-carbonyl)piperidine-1-carboxylate

^{31}P NMR (162 MHz, Chloroform-d) δ 18.78 (s, 61%), 18.24 (s, 39%).

^{1}H NMR (400 MHz, Chloroform-d) δ 7.69 – 7.25 (m, 1H, NH), 7.23 – 6.96 (m, 8H, 8xArH), 4.93 – 4.41 (m, 3H, CHP, 2xCH), 4.07 – 2.93 (m, 4H, 2xCH$_2$), 2.49 – 2.41 (m, 6H, 2xSCH$_3$), 2.41 – 2.11 (m, 2H, 0.5xCH$_2$, CH), 2.10 – 1.80 (m, 4H, 2xCH$_2$), 1.80 – 1.43 (m, 5H, 2.5xCH$_2$), 1.39 (d, J = 5.4 Hz, 9H, Boc), 1.16 – 0.97 (m, 6H, 2xCH$_3$).

HRMS: $C_{34}H_{48}N_3O_7PS_2$ [M+Na]$^+$ calc. 728.2569 found 728.2559

HPLC: purity >99%; retention time 19.637 min

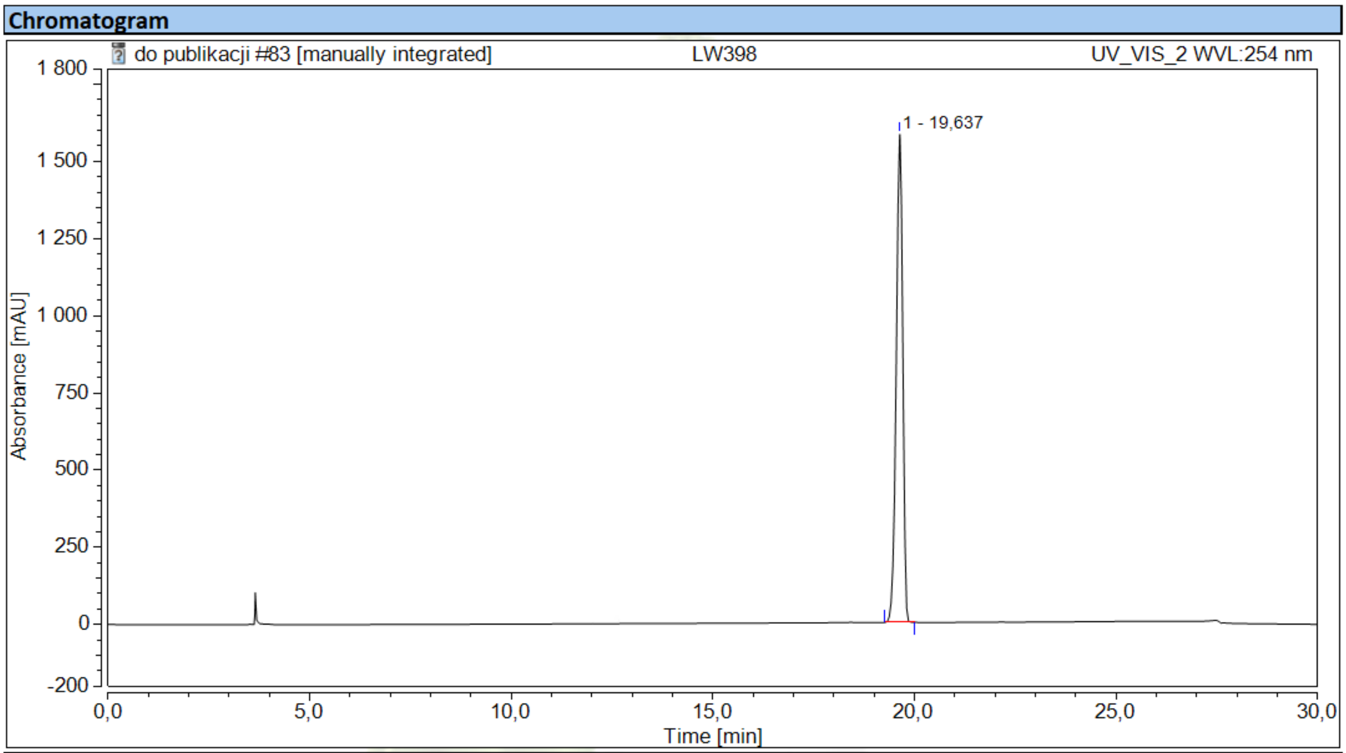

Integration Results

No.	Peak Name	Retention Time min	Area mAU*min	Height mAU	Relative Area %	Relative Height %	Amount n.a.
1		19,637	295,547	1580,758	100,00	100,00	n.a.
**Total:**			**295,547**	**1580,758**	**100,00**	**100,00**	

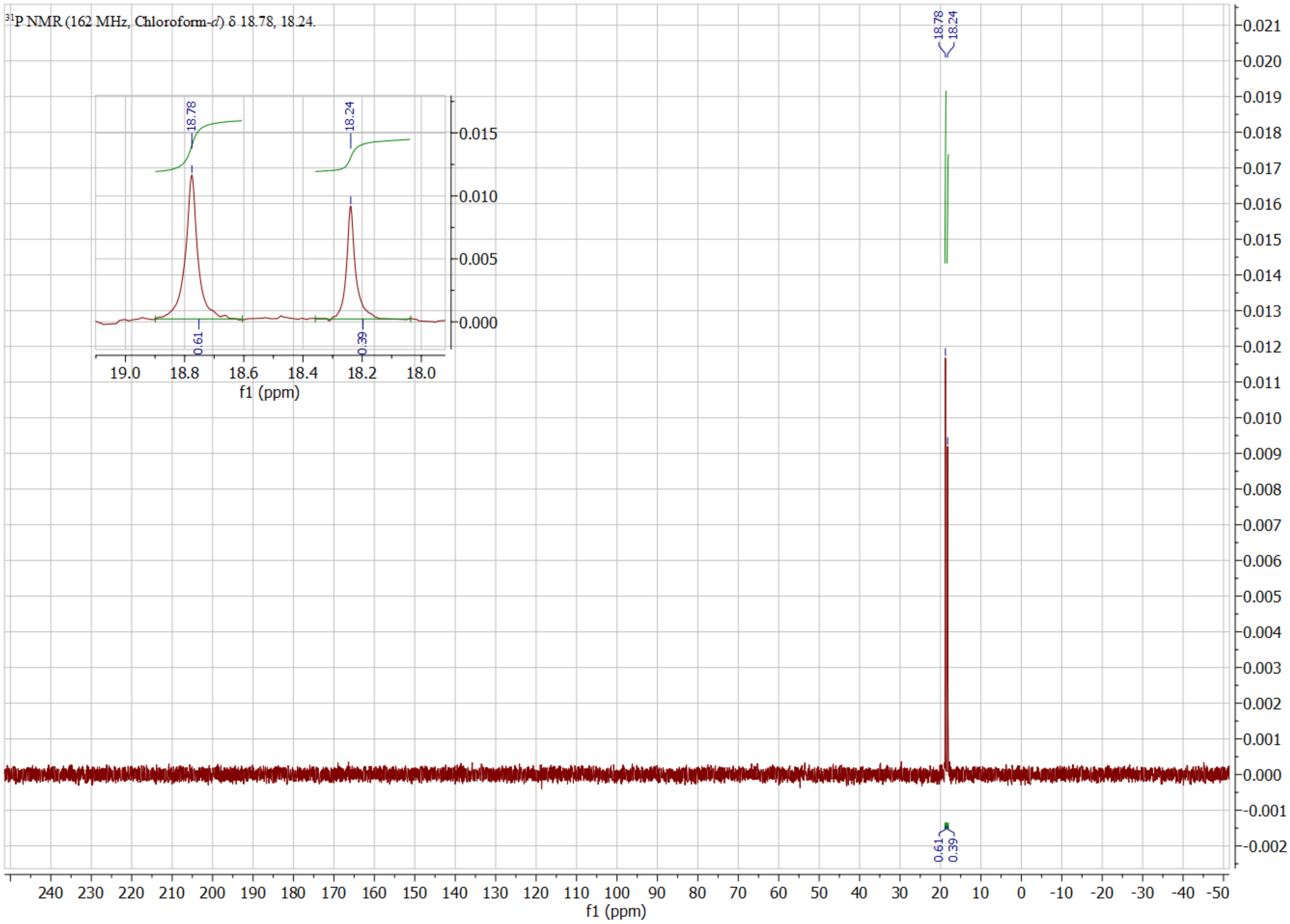

31P NMR (162 MHz, Chloroform-d) δ 18.78, 18.24.
18.78
18.24
0.61
0.39
f1 (ppm)

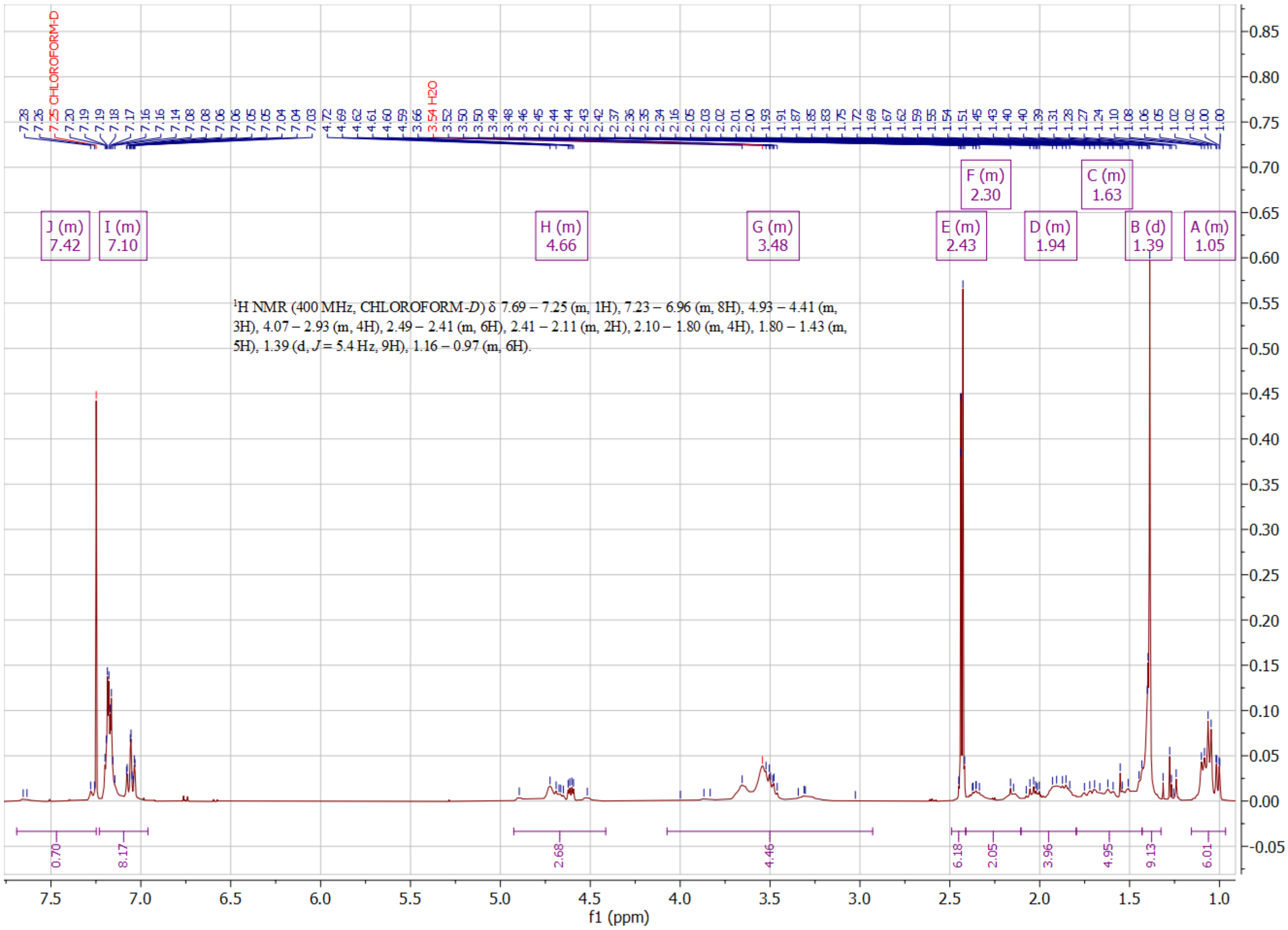

1H NMR (400 MHz, CHLOROFORM-D) δ 7.69 – 7.25 (m, 1H), 7.23 – 6.96 (m, 8H), 4.93 – 4.41 (m, 3H), 4.07 – 2.93 (m, 4H), 2.49 – 2.41 (m, 6H), 2.41 – 2.11 (m, 2H), 2.10 – 1.80 (m, 4H), 1.80 – 1.43 (m, 5H), 1.39 (d, J = 5.4 Hz, 9H), 1.16 – 0.97 (m, 6H).
CHLOROFORM-D
H2O
J (m) 7.42
I (m) 7.10
H (m) 4.66
G (m) 3.48
F (m) 2.30
E (m) 2.43
D (m) 1.94
C (m) 1.63
B (d) 1.39
A (m) 1.05
f1 (ppm)

(84) Boc-L-Nip-L-Pro- Val^{P}(OPh-4-SCH$_3$)$_2$

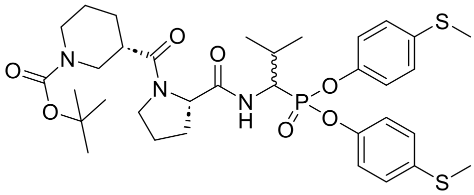

Name: *tert*-butyl (3S)-3-((2S)-2-((1-(bis(4-(methylthio)phenoxy)phosphoryl)-2-methylpropyl)carbamoyl)pyrrolidine-1-carbonyl)piperidine-1-carboxylate

^{31}P NMR (162 MHz, Chloroform-d) δ 18.61 (s, 63%), 18.20 (s, 37%).

^{1}H NMR (400 MHz, Chloroform-d) δ 7.72 (t, J = 9.9 Hz, 1H, NH), 7.24 – 6.96 (m, 8H, 8xArH), 4.81 – 4.51 (m, 2H, CHP, CH), 4.31 – 3.86 (m, 2H, CH, 0.5xCH$_2$), 3.76 – 3.33 (m, 2H, CH$_2$), 2.71 – 2.45 (m, 3H, CH$_2$, CH), 2.45 – 2.41 (m, 6H, 2xSCH$_3$), 2.41 – 2.23 (m, 2H, CH$_2$), 2.23 – 1.59 (m, 6H, 3xCH$_2$), 1.48 – 1.28 (m, 10H, Boc, 0.5xCH$_2$), 1.12 – 0.94 (m, 6H, 2xCH$_3$).

HRMS: $C_{34}H_{48}N_3O_7PS_2$ [M+Na]$^+$ calc. 728.2569 found 728.2551

HPLC: purity >99%; retention time 18.507 and 18.693 min

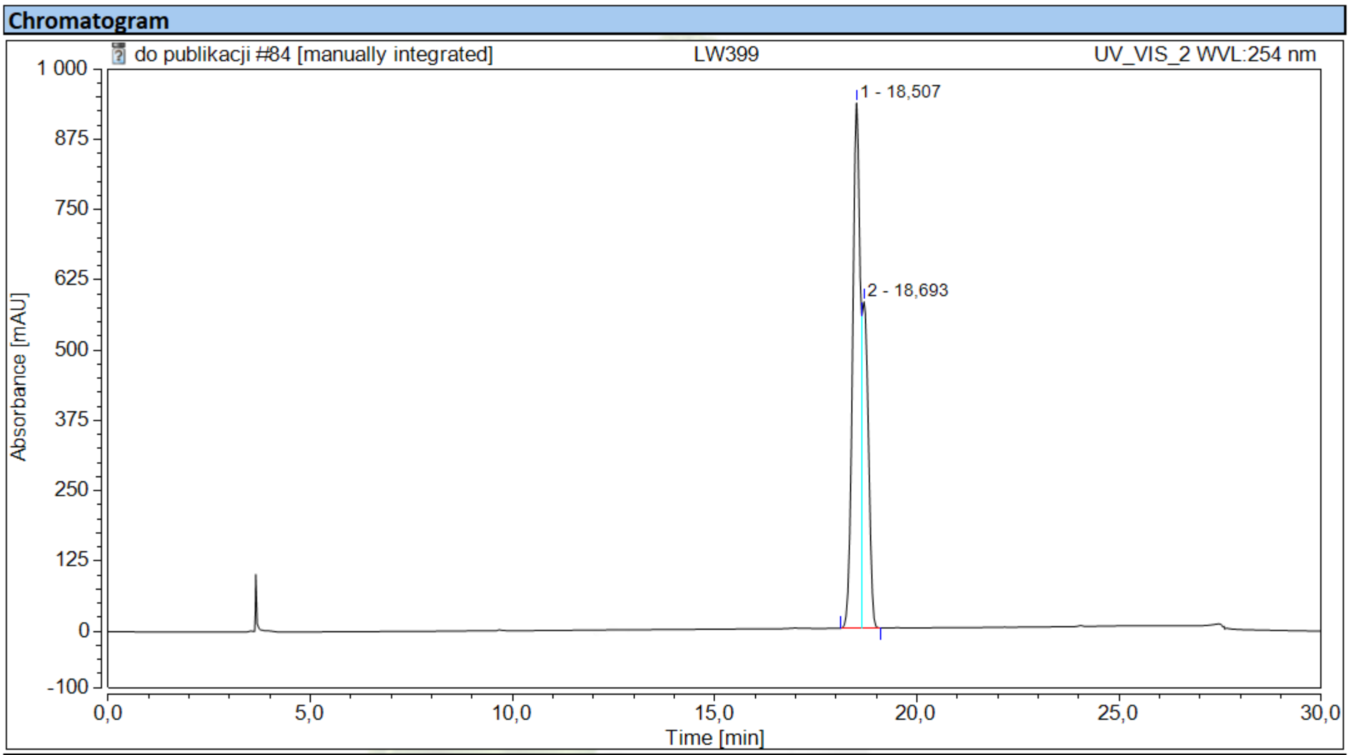

Integration Results

No.	Peak Name	Retention Time min	Area mAU*min	Height mAU	Relative Area %	Relative Height %	Amount n.a.
1		18,507	201,725	933,655	65,80	61,67	n.a.
2		18,693	104,834	580,257	34,20	38,33	n.a.
**Total:**			**306,558**	**1513,912**	**100,00**	**100,00**	

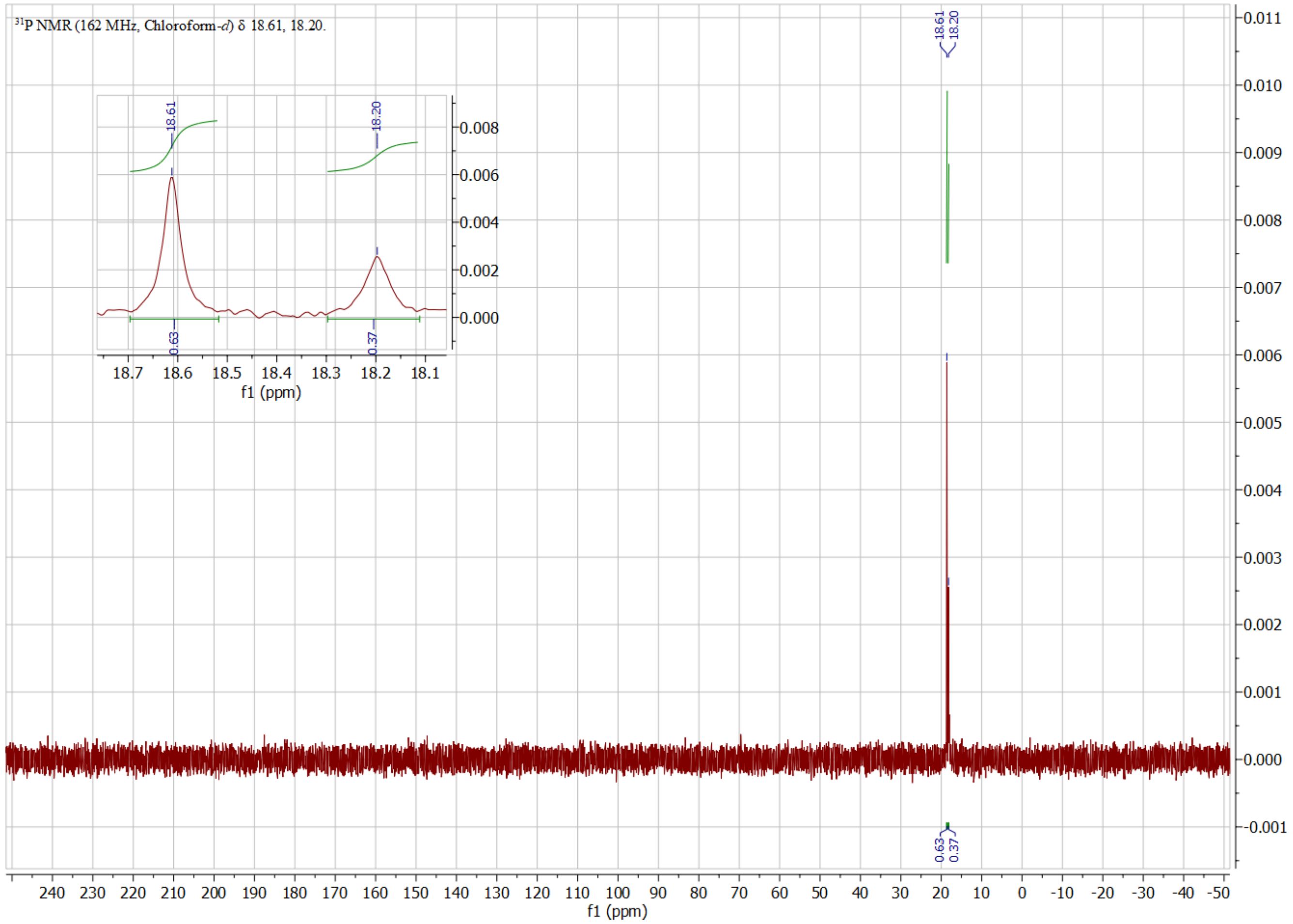

31P NMR (162 MHz, Chloroform-d) δ 18.61, 18.20.
f1 (ppm)

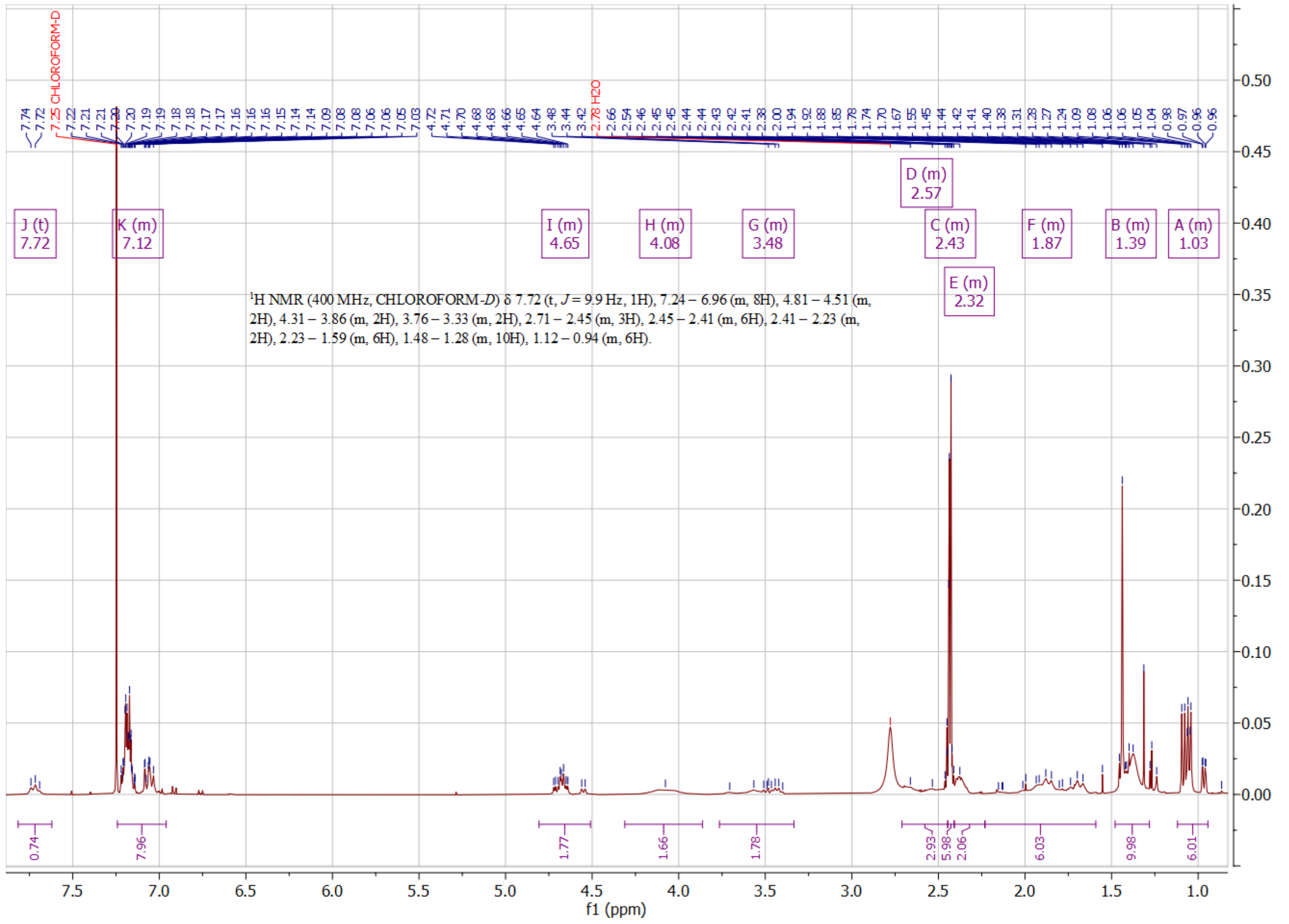

1H NMR (400 MHz, CHLOROFORM-D) δ 7.72 (t, J = 9.9 Hz, 1H), 7.24 – 6.96 (m, 8H), 4.81 – 4.51 (m, 2H), 4.31 – 3.86 (m, 2H), 3.76 – 3.33 (m, 2H), 2.71 – 2.45 (m, 3H), 2.45 – 2.41 (m, 6H), 2.41 – 2.23 (m, 2H), 2.23 – 1.59 (m, 6H), 1.48 – 1.28 (m, 10H), 1.12 – 0.94 (m, 6H).
f1 (ppm)

(85) Boc-D-Nip-L-Pro- Val^{P}(OPh-4-SCH$_3$)$_2$

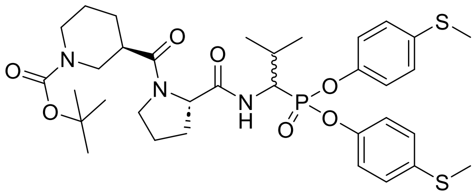

Name: *tert*-butyl (3R)-3-((2S)-2-((1-(bis(4-(methylthio)phenoxy)phosphoryl)-2-methylpropyl)carbamoyl)pyrrolidine-1-carbonyl)piperidine-1-carboxylate

^{31}P NMR (162 MHz, Chloroform-d) δ 18.42 (s, 64%), 18.16 (s, 36%).

^{1}H NMR (400 MHz, Chloroform-d) δ 7.66 (dd, J = 23.5, 10.3 Hz, 1H, NH), 7.23 – 6.95 (m, 8H, 8xArH), 4.77 – 4.52 (m, 2H, CHP, CH), 4.22 – 3.92 (m, 2H, 0.5xCH$_2$, CH), 3.55 – 3.34 (m, 2H, CH$_2$), 2.98 – 2.47 (m, 2H, CH$_2$), 2.46 – 2.42 (m, 6H, 2xSCH$_3$), 2.42 – 2.22 (m, 3H, CH$_2$, CH), 2.20 – 1.68 (m, 4H, 2xCH$_2$), 1.68 – 1.47 (m, 2H, CH$_2$), 1.44 (d, J = 5.3 Hz, 9H, Boc), 1.39 – 1.23 (m, 1H, 0.5xCH$_2$), 1.12 – 0.93 (m, 6H, 2xCH$_3$).

HRMS: $C_{34}H_{48}N_3O_7PS_2$ [M+Na]$^+$ calc. 728.2569 found 728.2559

HPLC: purity >99%; retention time 18.417 and 18.743 min

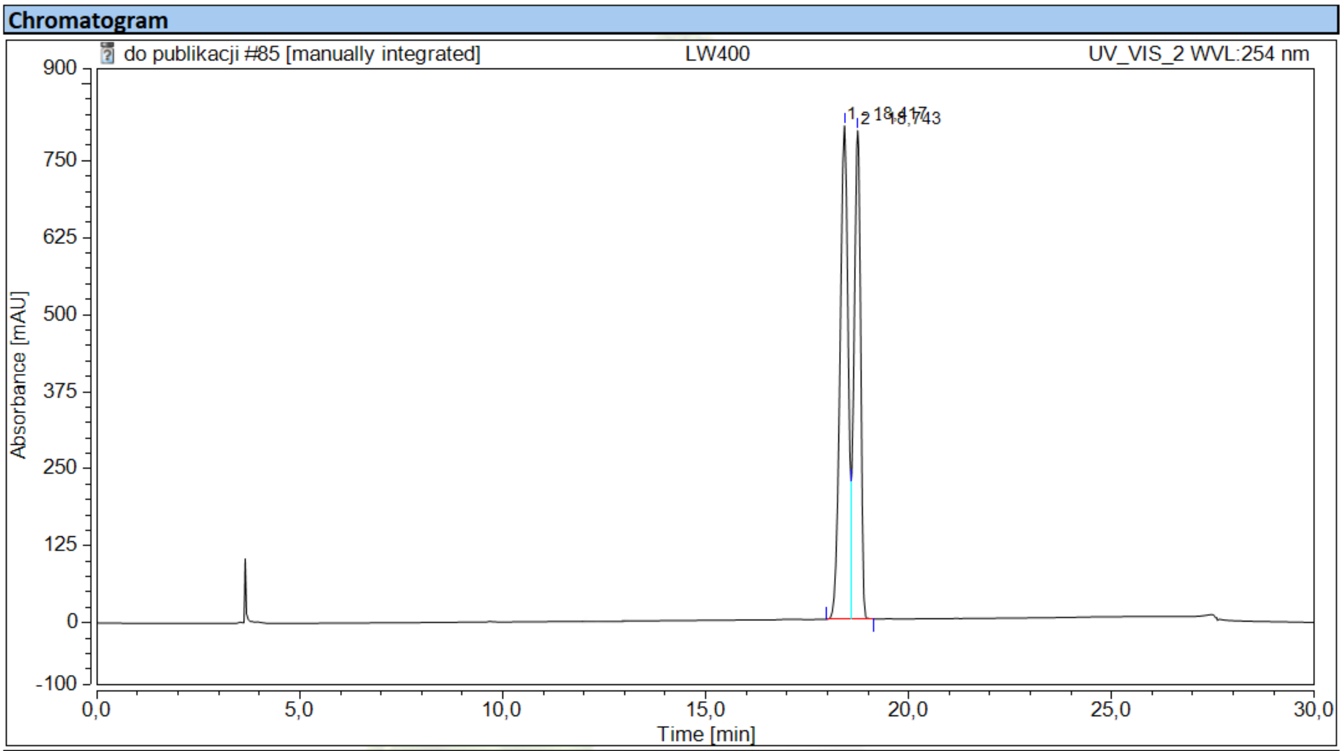

Integration Results

No.	Peak Name	Retention Time min	Area mAU*min	Height mAU	Relative Area %	Relative Height %	Amount n.a.
1		18,417	183,706	801,197	55,23	50,25	n.a.
2		18,743	148,924	793,146	44,77	49,75	n.a.
**Total:**			**332,631**	**1594,343**	**100,00**	**100,00**	

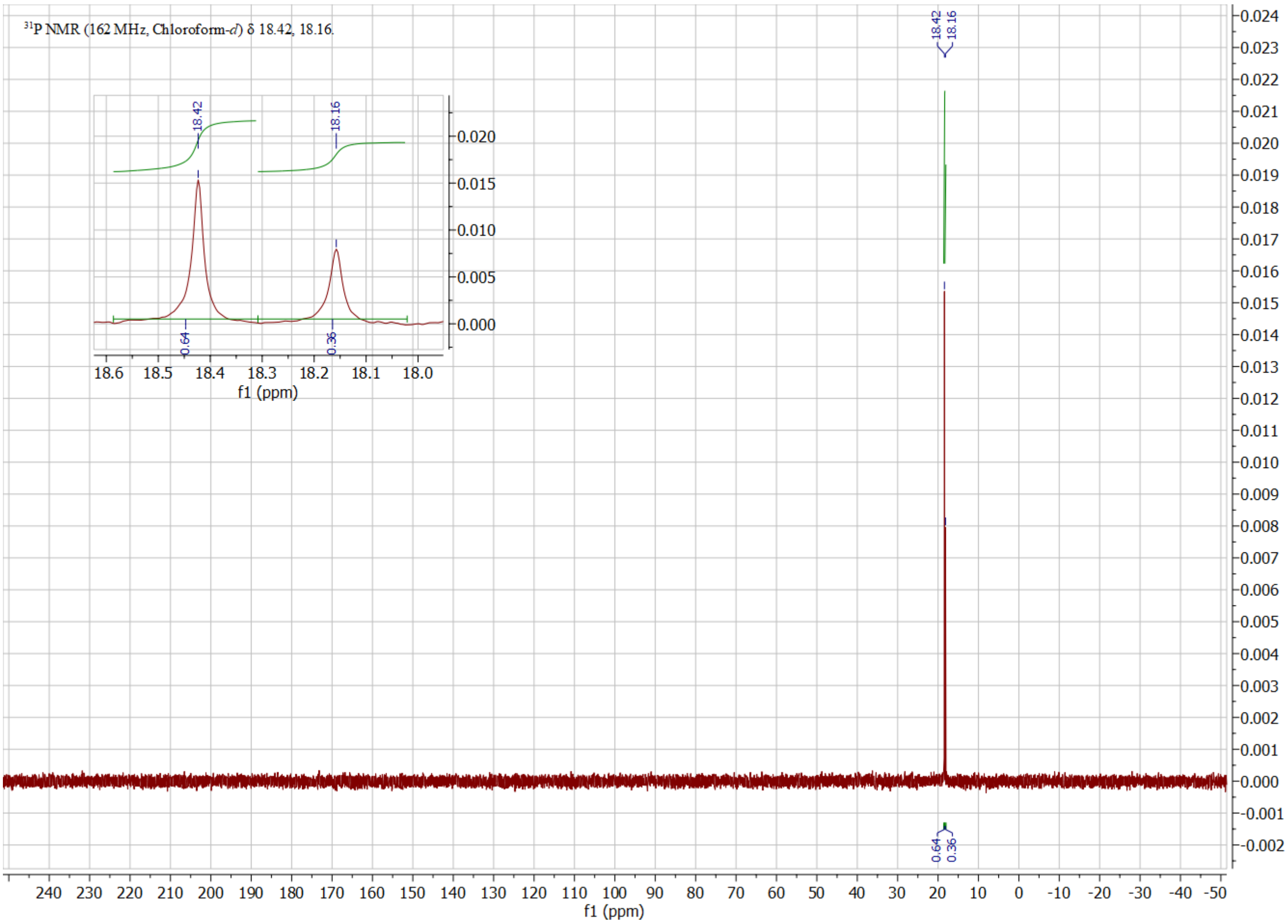
^{31}P NMR (162 MHz, Chloroform-*d*) δ 18.42, 18.16.
f1 (ppm)

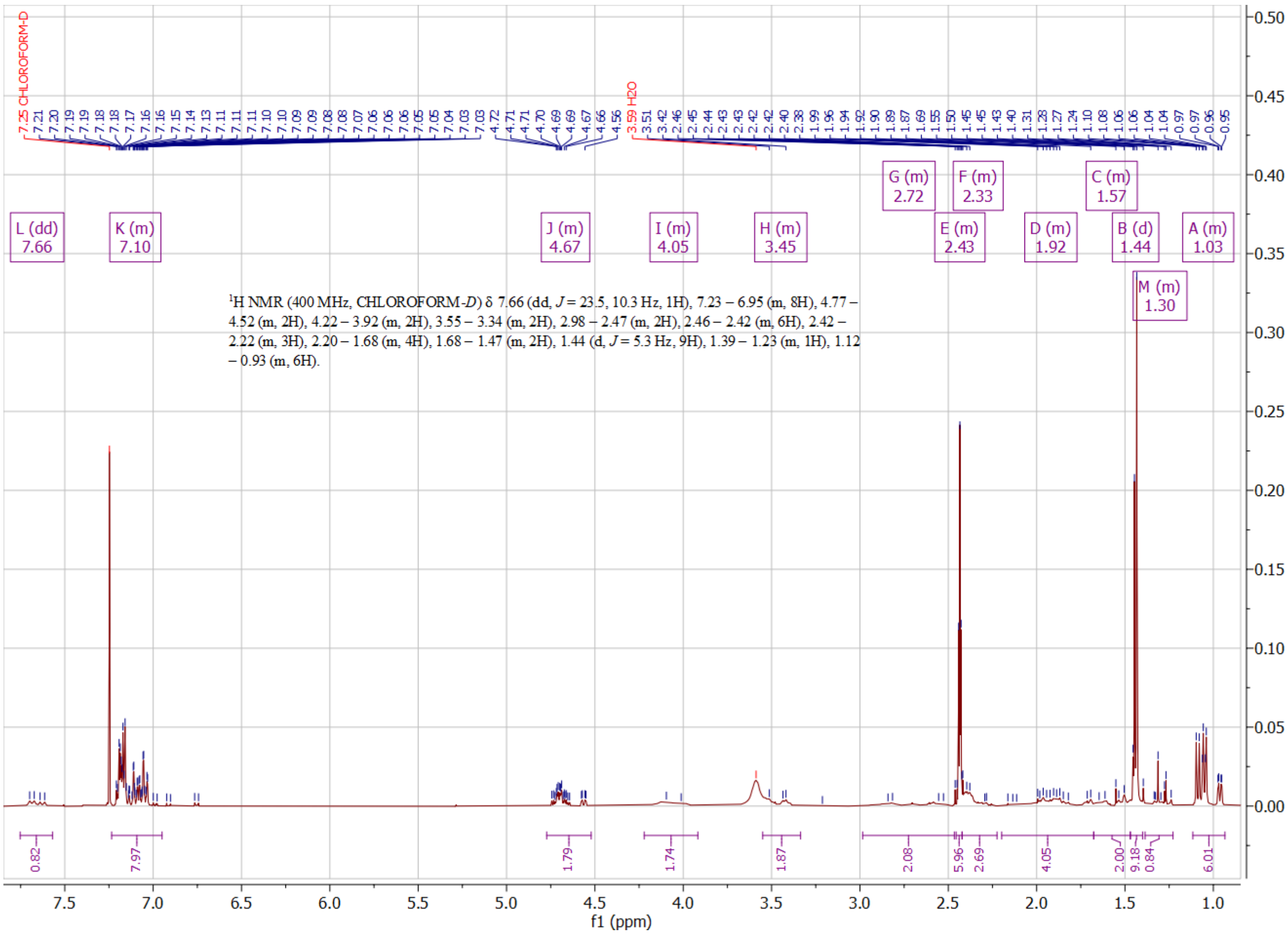
^{1}H NMR (400 MHz, CHLOROFORM-*D*) δ 7.66 (dd, *J* = 23.5, 10.3 Hz, 1H), 7.23 – 6.95 (m, 8H), 4.77 – 4.52 (m, 2H), 4.22 – 3.92 (m, 2H), 3.55 – 3.34 (m, 2H), 2.98 – 2.47 (m, 2H), 2.46 – 2.42 (m, 6H), 2.42 – 2.22 (m, 3H), 2.20 – 1.68 (m, 4H), 1.68 – 1.47 (m, 2H), 1.44 (d, *J* = 5.3 Hz, 9H), 1.39 – 1.23 (m, 1H), 1.12 – 0.93 (m, 6H).
f1 (ppm)

(86) Boc-1,4-cis-ACHC-L-Pro-Val^P(OPh-4-SCH$_3$)$_2$

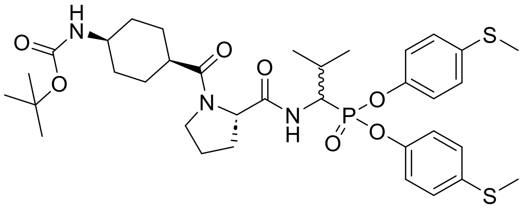

Name: *tert*-butyl ((1R,4s)-4-((2S)-2-((1-(bis(4-(methylthio)phenoxy)phosphoryl)-2-methylpropyl)carbamoyl)pyrrolidine-1-carbonyl)cyclohexyl)carbamate
^{31}P NMR (162 MHz, Chloroform-d) δ 18.01 (s, 48%), 17.96 (s, 52%).
^{1}H NMR (400 MHz, Chloroform-d) δ 7.53 (dd, J = 30.1, 10.4 Hz, 1H, NH), 7.22 – 6.89 (m, 8H, 8xArH), 4.84 – 4.47 (m, 2H, NH, CHP), 3.88 – 3.39 (m, 3H, CH, CH$_2$), 2.45 – 2.42 (m, 6H, 2xSCH$_3$), 2.42 – 1.46 (m, 15H, 6xCH$_2$, 3xCH), 1.43 (s, 9H, Boc), 1.16 – 0.95 (m, 6H, 2xCH$_3$).
HRMS: $C_{35}H_{50}N_3O_7PS_2$ [M+Na]$^+$ calc. 742.2725 found 742.2712
HPLC: purity >99%; retention time 18.063 min

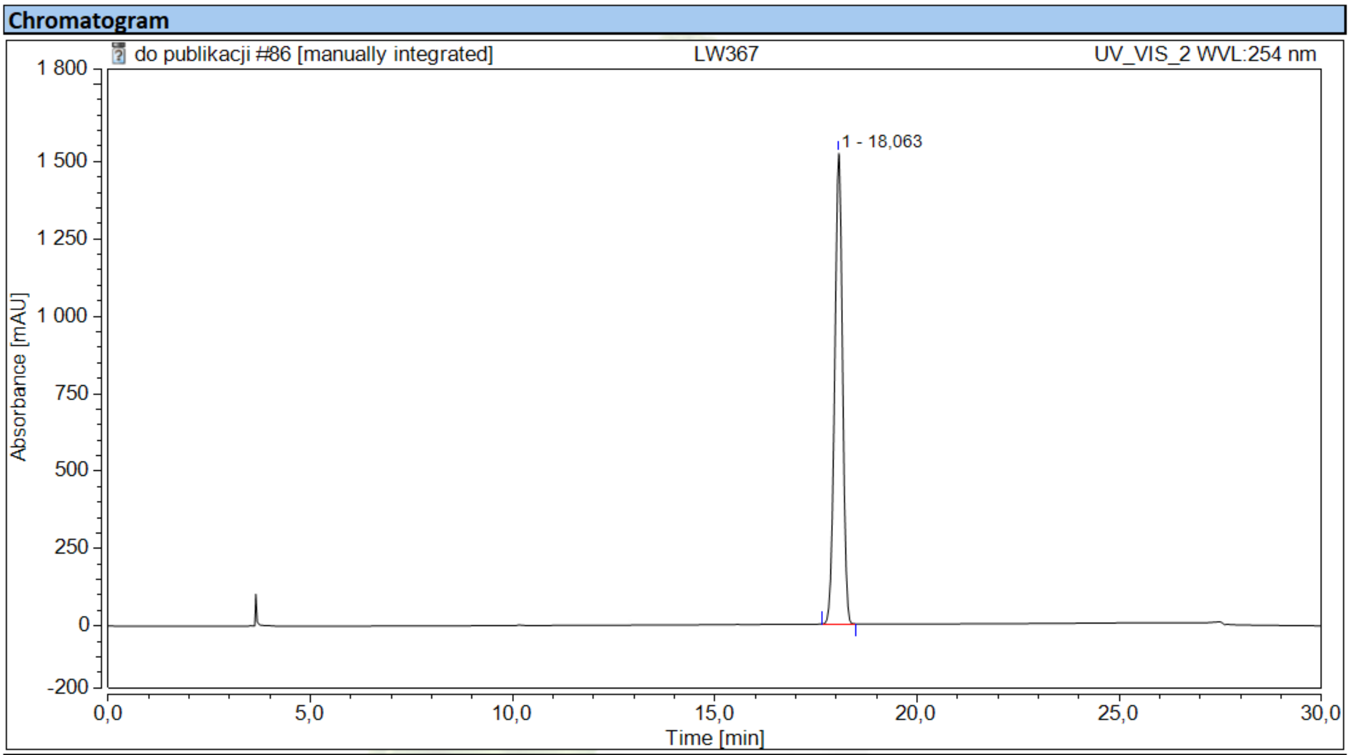

Integration Results

No.	Peak Name	Retention Time min	Area mAU*min	Height mAU	Relative Area %	Relative Height %	Amount n.a.
1		18,063	341,633	1520,614	100,00	100,00	n.a.
**Total:**			**341,633**	**1520,614**	**100,00**	**100,00**	

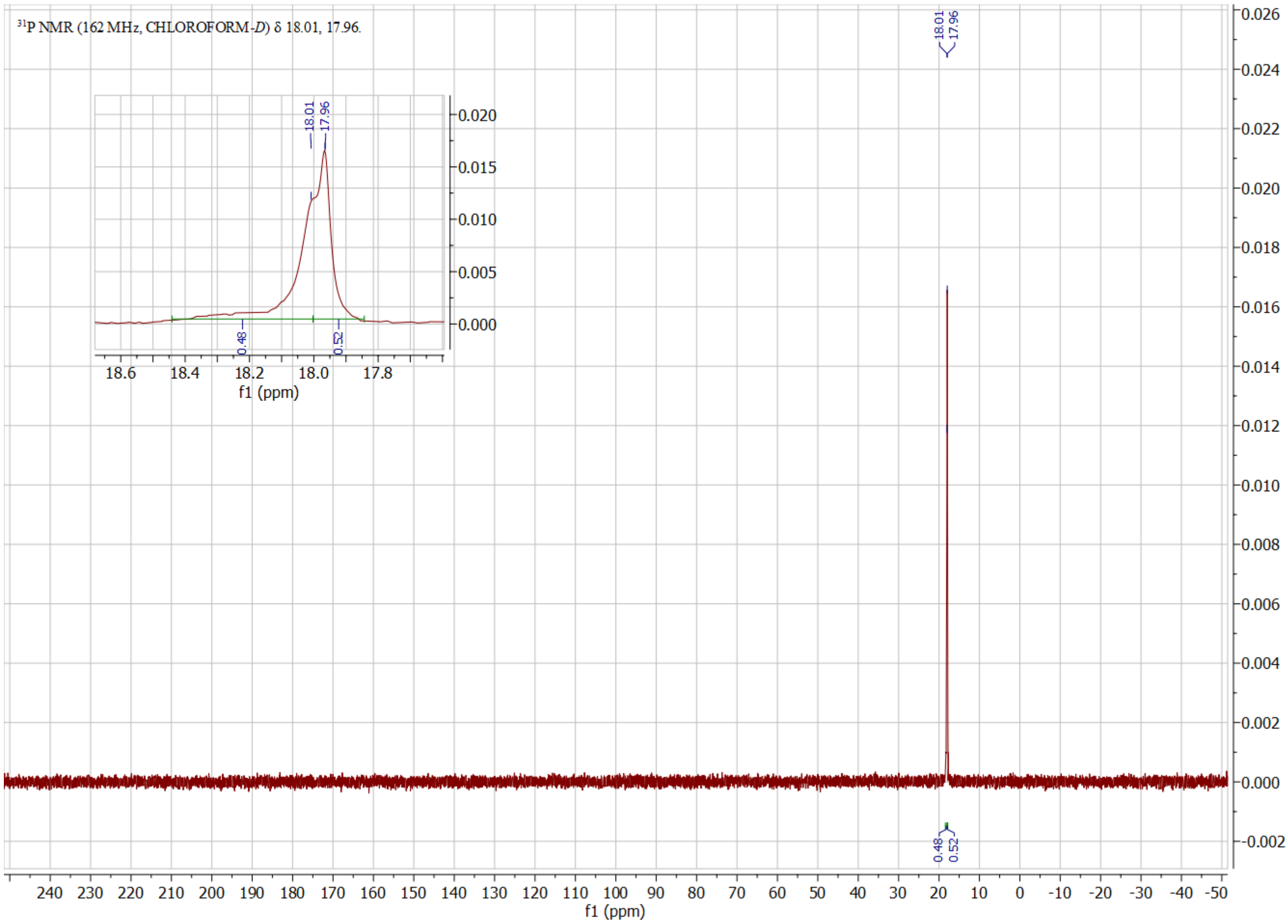
³¹P NMR (162 MHz, CHLOROFORM-D) δ 18.01, 17.96.
f1 (ppm)

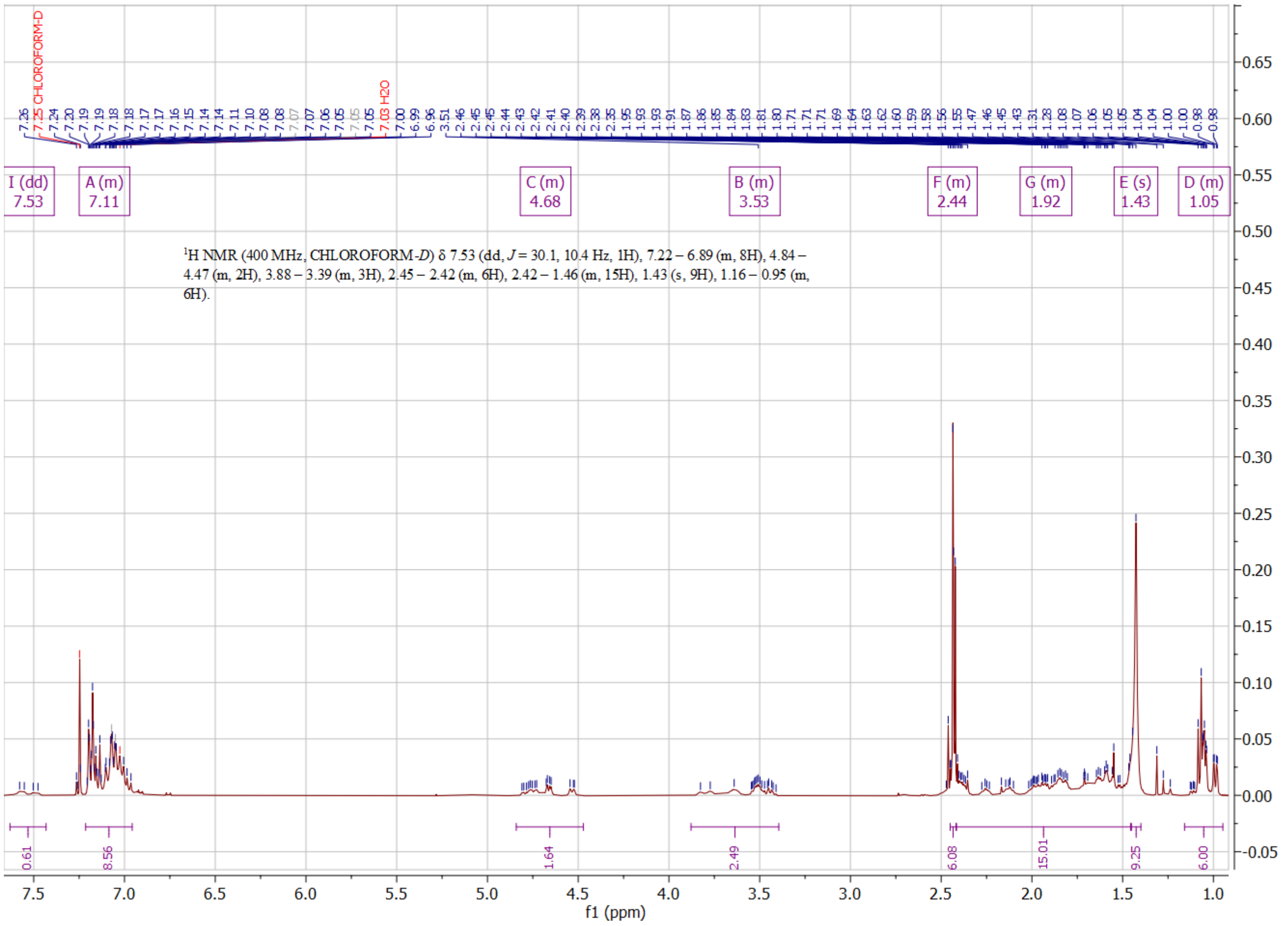
¹H NMR (400 MHz, CHLOROFORM-D) δ 7.53 (dd, J = 30.1, 10.4 Hz, 1H), 7.22 – 6.89 (m, 8H), 4.84 – 4.47 (m, 2H), 3.88 – 3.39 (m, 3H), 2.45 – 2.42 (m, 6H), 2.42 – 1.46 (m, 15H), 1.43 (s, 9H), 1.16 – 0.95 (m, 6H).
I (dd) 7.53
A (m) 7.11
C (m) 4.68
B (m) 3.53
F (m) 2.44
G (m) 1.92
E (s) 1.43
D (m) 1.05
f1 (ppm)

(87) Boc-1,4-trans-ACHC-L-Pro-Val^{P}(OPh-4-SCH$_3$)$_2$

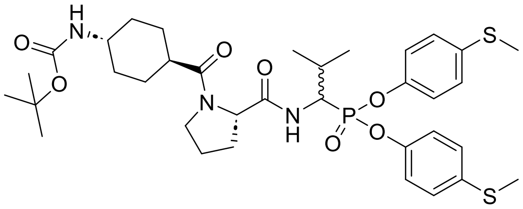

Name: *tert*-butyl ((1S,4r)-4-((2S)-2-((1-(bis(4-(methylthio)phenoxy)phosphoryl)-2-methylpropyl)carbamoyl)pyrrolidine-1-carbonyl)cyclohexyl)carbamate
^{31}**P NMR** (162 MHz, Chloroform-d) δ 18.70 (s, 49%), 18.15 (s, 51%).
^{1}**H NMR** (400 MHz, Chloroform-d) δ 1H NMR (400 MHz, CHLOROFORM-D) δ 7.75 (d, J = 10.3 Hz, 1H, NH), 7.21 – 6.94 (m, 8H, 8xArH), 4.78 – 4.30 (m, 2H, NH, CHP), 3.69 – 3.17 (m, 3H, CH_2, CH), 2.49 – 2.41 (m, 6H), 2.41 – 1.50 (m, 15H, 6xCH_2, 3xCH), 1.43 (s, 9H, Boc), 1.09 – 0.91 (m, 6H, 2xCH_3).
HRMS: $C_{35}H_{50}N_3O_7PS_2$ $[M+Na]^+$ calc. 742.2725 found 742.2707
HPLC: purity >99%; retention time 17.807 min

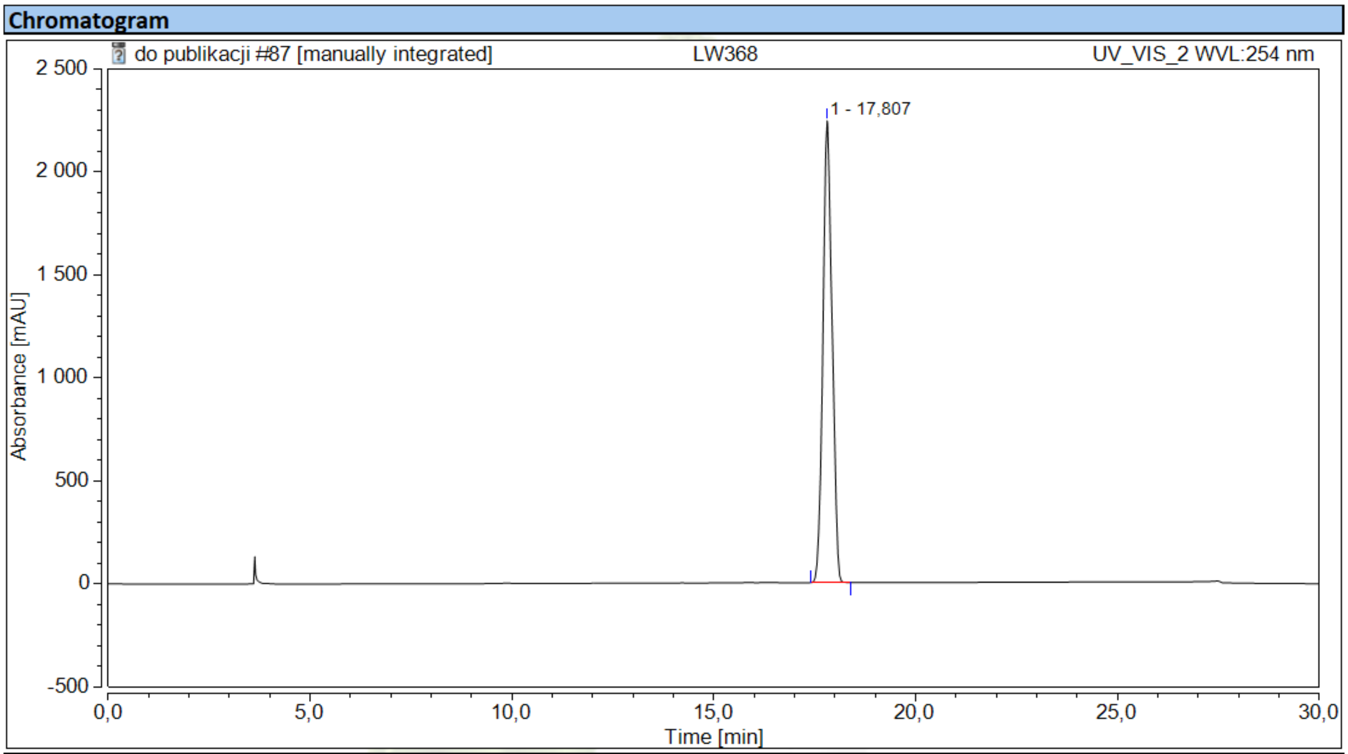

Integration Results

No.	Peak Name	Retention Time min	Area mAU*min	Height mAU	Relative Area %	Relative Height %	Amount n.a.
1		17,807	588,010	2241,698	100,00	100,00	n.a.
**Total:**			**588,010**	**2241,698**	**100,00**	**100,00**	

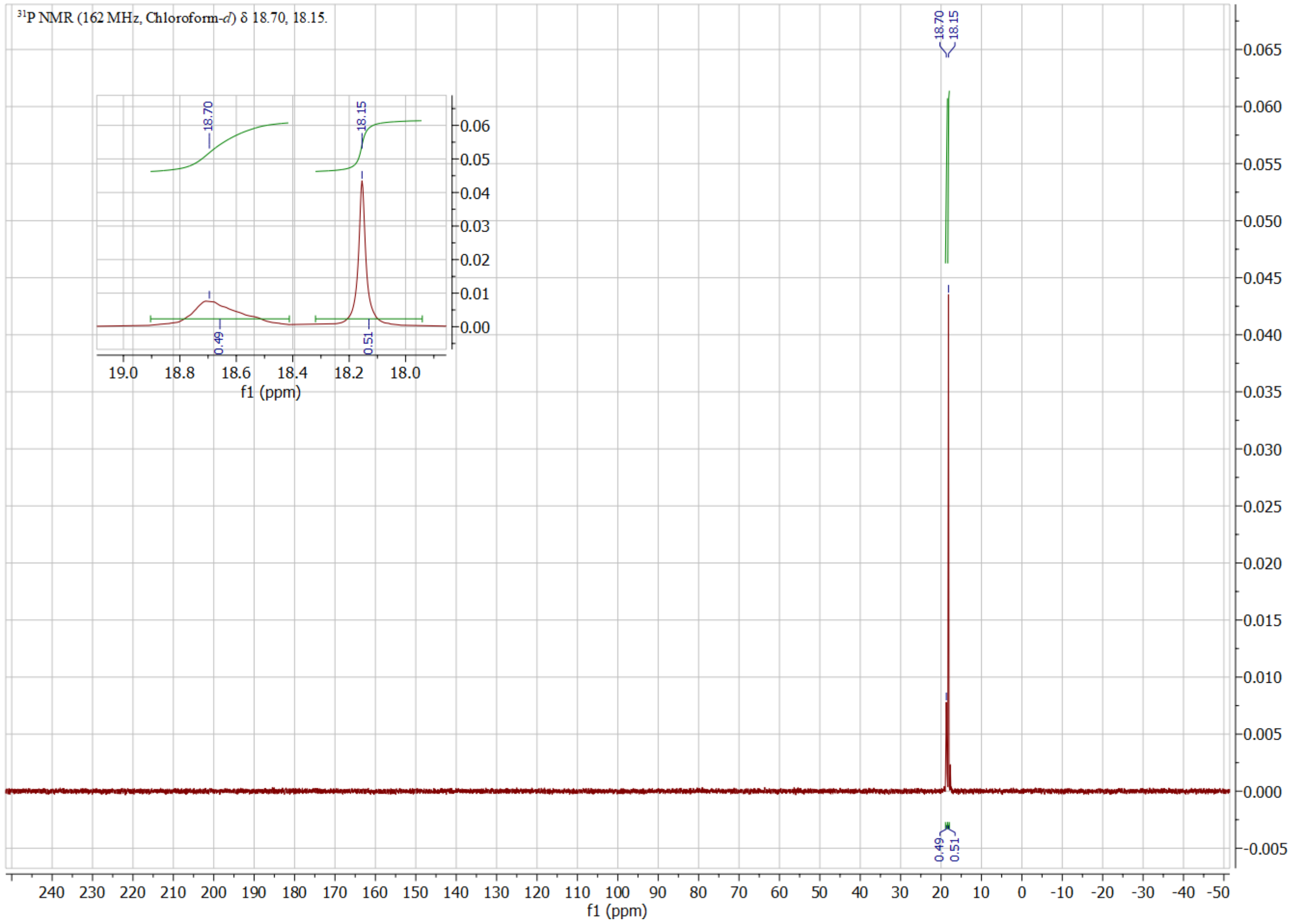

^{31}P NMR (162 MHz, Chloroform-*d*) δ 18.70, 18.15.
f1 (ppm)

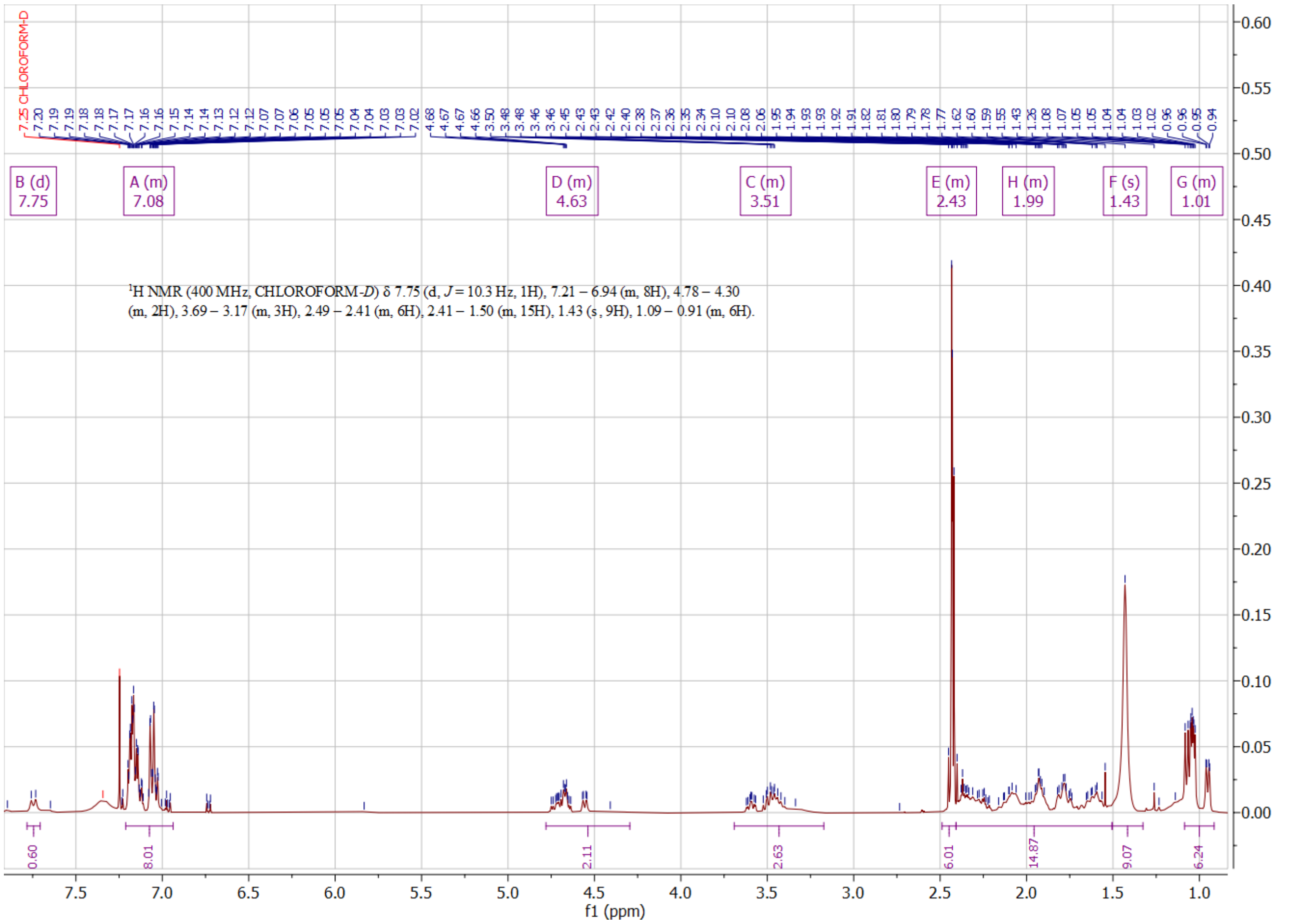

^{1}H NMR (400 MHz, CHLOROFORM-*D*) δ 7.75 (d, *J* = 10.3 Hz, 1H), 7.21 – 6.94 (m, 8H), 4.78 – 4.30 (m, 2H), 3.69 – 3.17 (m, 3H), 2.49 – 2.41 (m, 6H), 2.41 – 1.50 (m, 15H), 1.43 (s, 9H), 1.09 – 0.91 (m, 6H).
B (d) 7.75
A (m) 7.08
D (m) 4.63
C (m) 3.51
E (m) 2.43
H (m) 1.99
F (s) 1.43
G (m) 1.01
f1 (ppm)

(88) Boc-L-Cpg-L-Pro-Val^P(OPh-4-SCH$_3$)$_2$

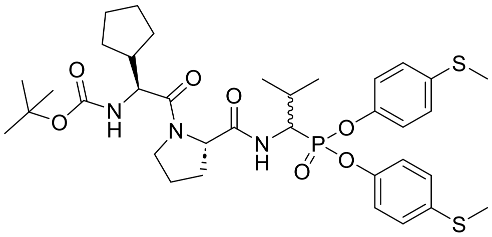

Name: *tert*-butyl ((1S)-2-((2S)-2-((1-(bis(4-(methylthio)phenoxy)phosphoryl)-2-methylpropyl)carbamoyl)pyrrolidin-1-yl)-1-cyclopentyl-2-oxoethyl)carbamate

^{31}P NMR (162 MHz, Chloroform-d) δ 18.36 (s, 48%), 17.98 (s, 52%).

^{1}H NMR (400 MHz, Chloroform-d) δ 7.34 – 7.25 (m, 1H, NH), 7.23 – 7.13 (m, 4H, 4xArH), 7.13 – 7.02 (m, 4H, 4xArH), 5.35 (dd, J = 30.9, 8.9 Hz, 1H, NH), 4.79 – 4.69 (m, 1H, CHP), 4.69 – 4.46 (m, 1H, CH), 4.40 – 4.27 (m, 1H, CH), 3.88 – 3.77 (m, 1H, 0.5xCH$_2$), 3.69 – 3.53 (m, 1H, 0.5xCH$_2$), 2.43 (d, J = 1.2 Hz, 6H, 2xSCH$_3$), 2.42 – 2.16 (m, 2H, 2xCH), 2.16 – 1.82 (m, 4H, 2xCH$_2$), 1.74 – 1.58 (m, 3H, 1.5xCH$_2$), 1.57 – 1.45 (m, 2H, CH$_2$), 1.40 (d, J = 2.3 Hz, 9H, Boc), 1.38 – 1.22 (m, 3H, 1.5xCH$_2$), 1.11 – 0.99 (m, 6H, 2xCH$_3$).

HRMS: $C_{35}H_{50}N_3O_7PS_2$ [M+Na]$^+$ calc. 742.2725 found 742.2720

HPLC: purity >99%; retention time 19.760 min

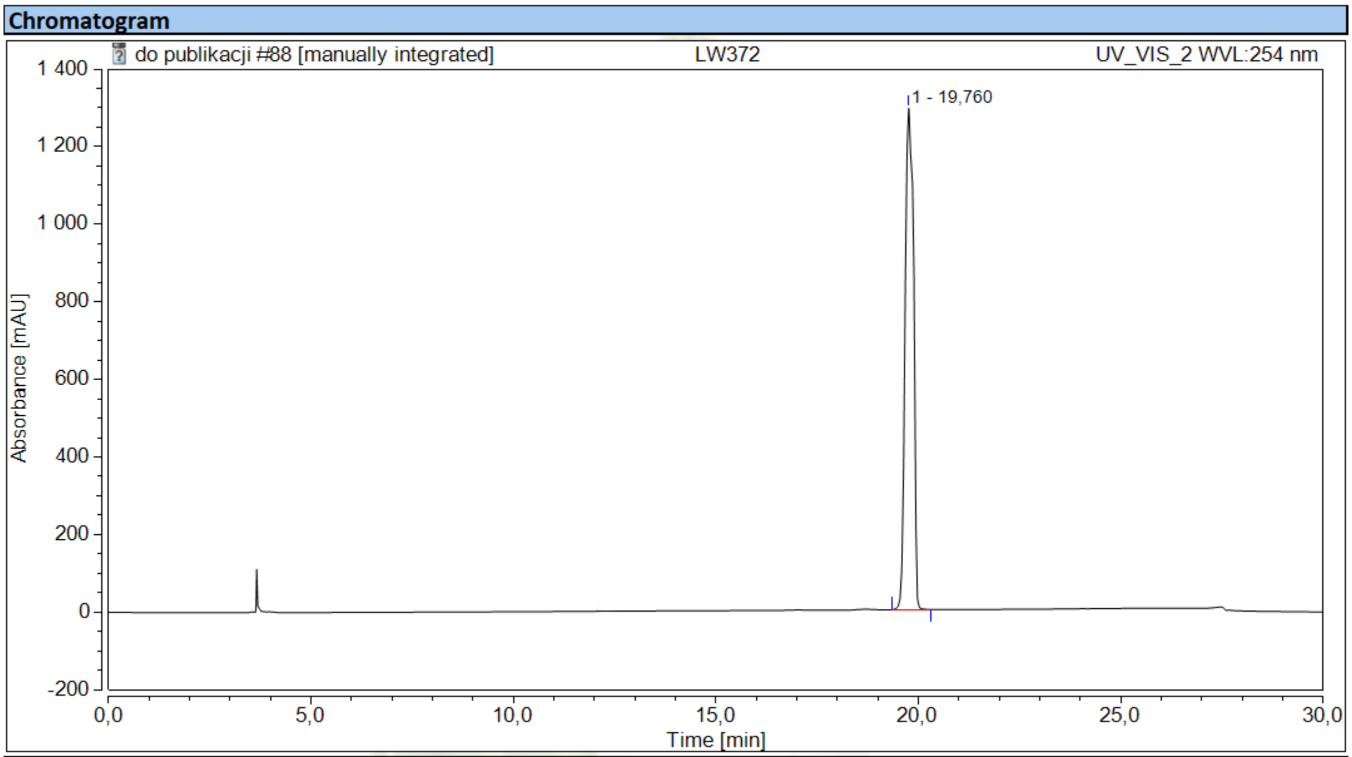

Integration Results

No.	Peak Name	Retention Time min	Area mAU*min	Height mAU	Relative Area %	Relative Height %	Amount n.a.
1		19,760	312,769	1292,252	100,00	100,00	n.a.
**Total:**			**312,769**	**1292,252**	**100,00**	**100,00**	

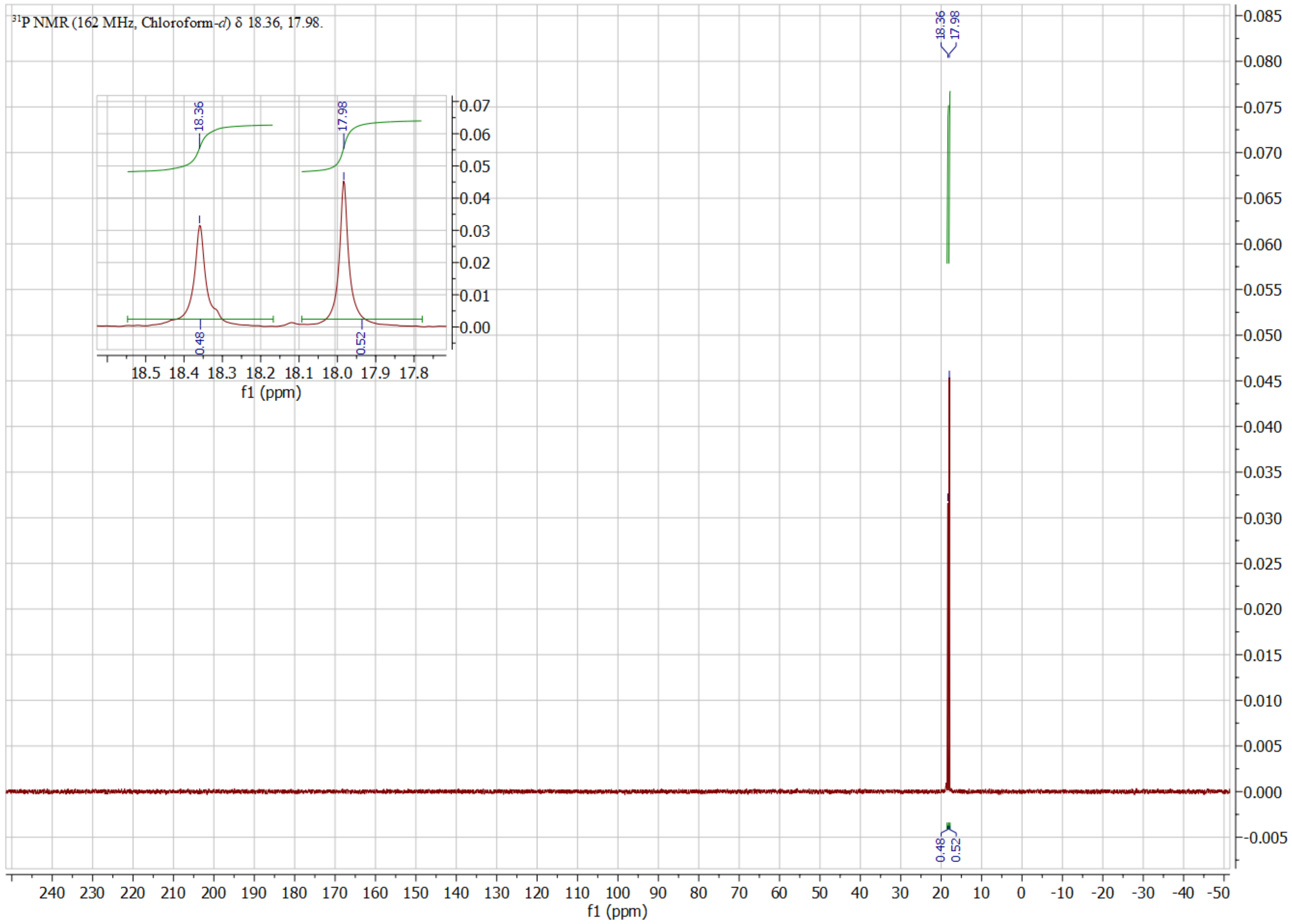
31P NMR (162 MHz, Chloroform-d) δ 18.36, 17.98.
f1 (ppm)

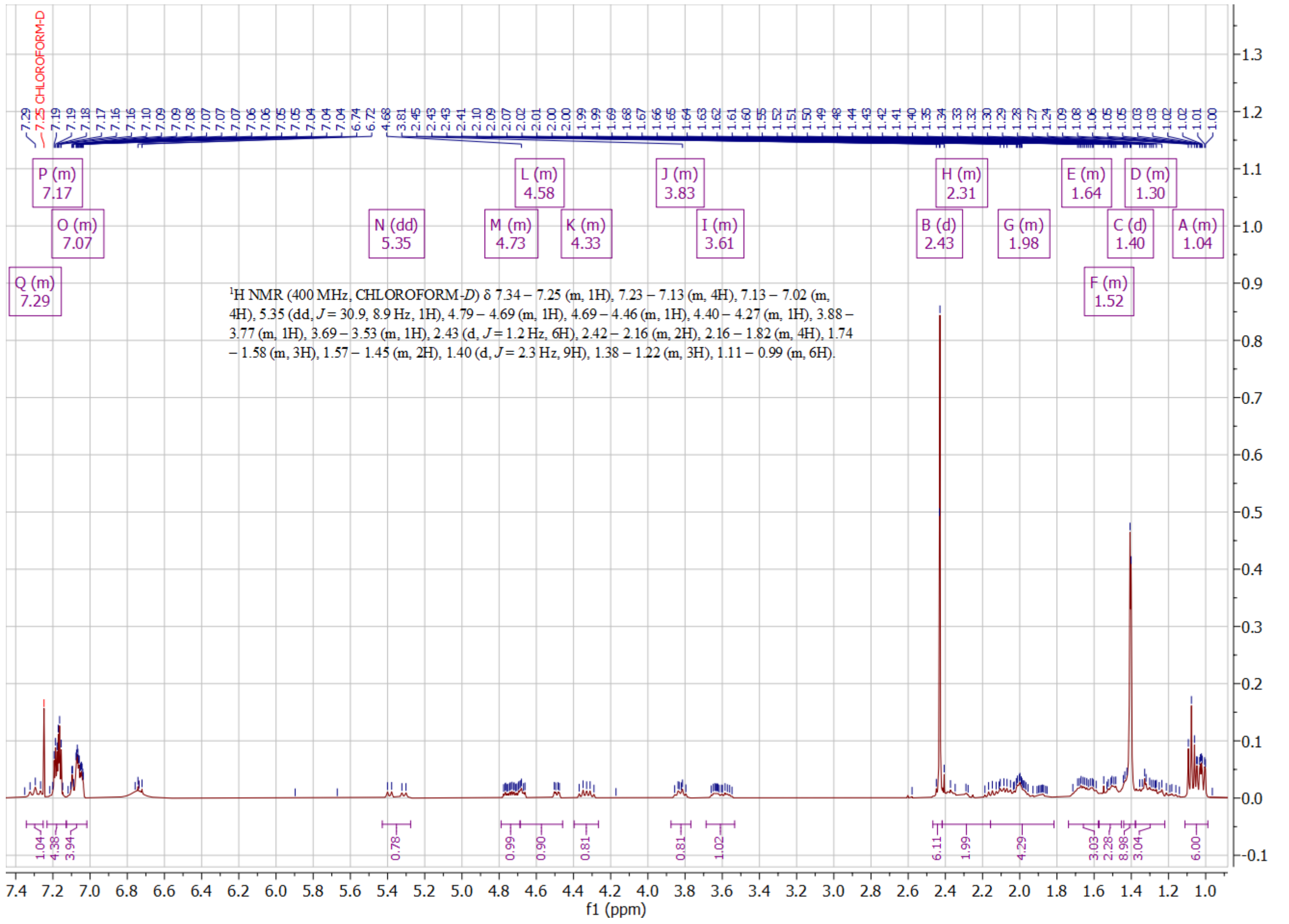
1H NMR (400 MHz, CHLOROFORM-D) δ 7.34 – 7.25 (m, 1H), 7.23 – 7.13 (m, 4H), 7.13 – 7.02 (m, 4H), 5.35 (dd, J = 30.9, 8.9 Hz, 1H), 4.79 – 4.69 (m, 1H), 4.69 – 4.46 (m, 1H), 4.40 – 4.27 (m, 1H), 3.88 – 3.77 (m, 1H), 3.69 – 3.53 (m, 1H), 2.43 (d, J = 1.2 Hz, 6H), 2.42 – 2.16 (m, 2H), 2.16 – 1.82 (m, 4H), 1.74 – 1.58 (m, 3H), 1.57 – 1.45 (m, 2H), 1.40 (d, J = 2.3 Hz, 9H), 1.38 – 1.22 (m, 3H), 1.11 – 0.99 (m, 6H).
f1 (ppm)

(89) Boc-L-Phg-L-Pro-Val^{P}(OPh-4-SCH$_3$)$_2$

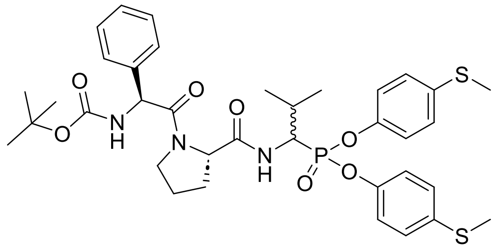

Name: *tert*-butyl ((1S)-2-((2S)-2-((1-(bis(4-(methylthio)phenoxy)phosphoryl)-2-methylpropyl)carbamoyl)pyrrolidin-1-yl)-2-oxo-1-phenylethyl)carbamate

^{31}P NMR (162 MHz, Chloroform-d) δ 18.39 (d, J = 4.2 Hz, 45%), 18.21 (s, 19%), 17.98 (s, 36%).

^{1}H NMR (400 MHz, Chloroform-d) δ 7.62 – 7.25 (m, 5H, 4xArH, NH), 7.22 – 7.11 (m, 6H, 6xArH), 7.11 – 6.96 (m, 3H, 3xArH), 5.92 – 5.72 (m, 1H, NH), 5.40 (dd, J = 31.5, 7.2 Hz, 1H, CH), 4.78 – 4.51 (m, 2H, CHP, CH), 3.76 – 3.53 (m, 1H, 0.5xCH$_2$), 3.34 – 3.01 (m, 1H, 0.5xCH$_2$), 2.46 – 2.40 (m, 6H, 2xSCH$_3$), 2.40 – 2.13 (m, 2H, 0.5xCH$_2$, CH), 2.03 – 1.62 (m, 3H, 1.5xCH$_2$), 1.45 – 1.31 (m, 9H, Boc), 1.13 – 0.85 (m, 6H, 2xCH$_3$).

HRMS: $C_{36}H_{46}N_3O_7PS_2$ [M+Na]$^+$ calc. 750.2412 found 750.2407

HPLC: purity >99%; retention time 19.180 min

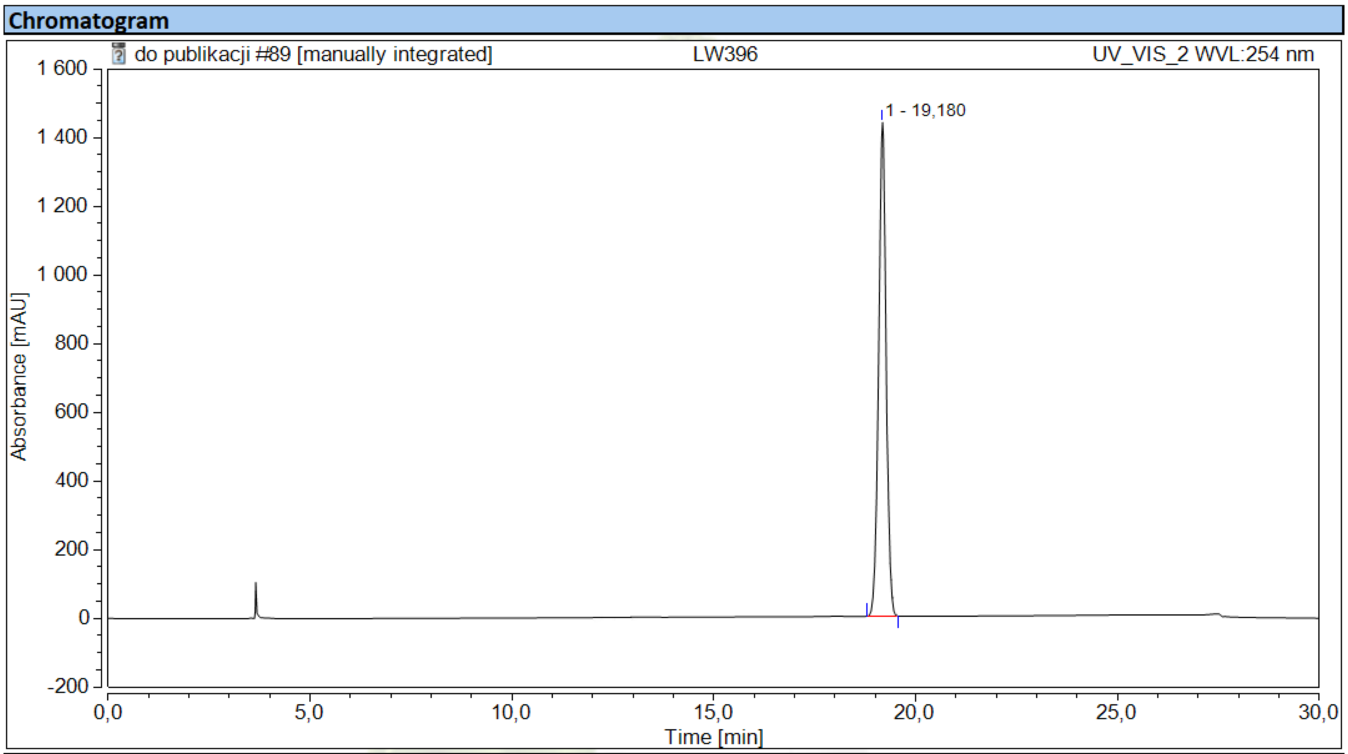

Integration Results

No.	Peak Name	Retention Time min	Area mAU*min	Height mAU	Relative Area %	Relative Height %	Amount n.a.
1		19,180	334,417	1437,657	100,00	100,00	n.a.
**Total:**			**334,417**	**1437,657**	**100,00**	**100,00**	

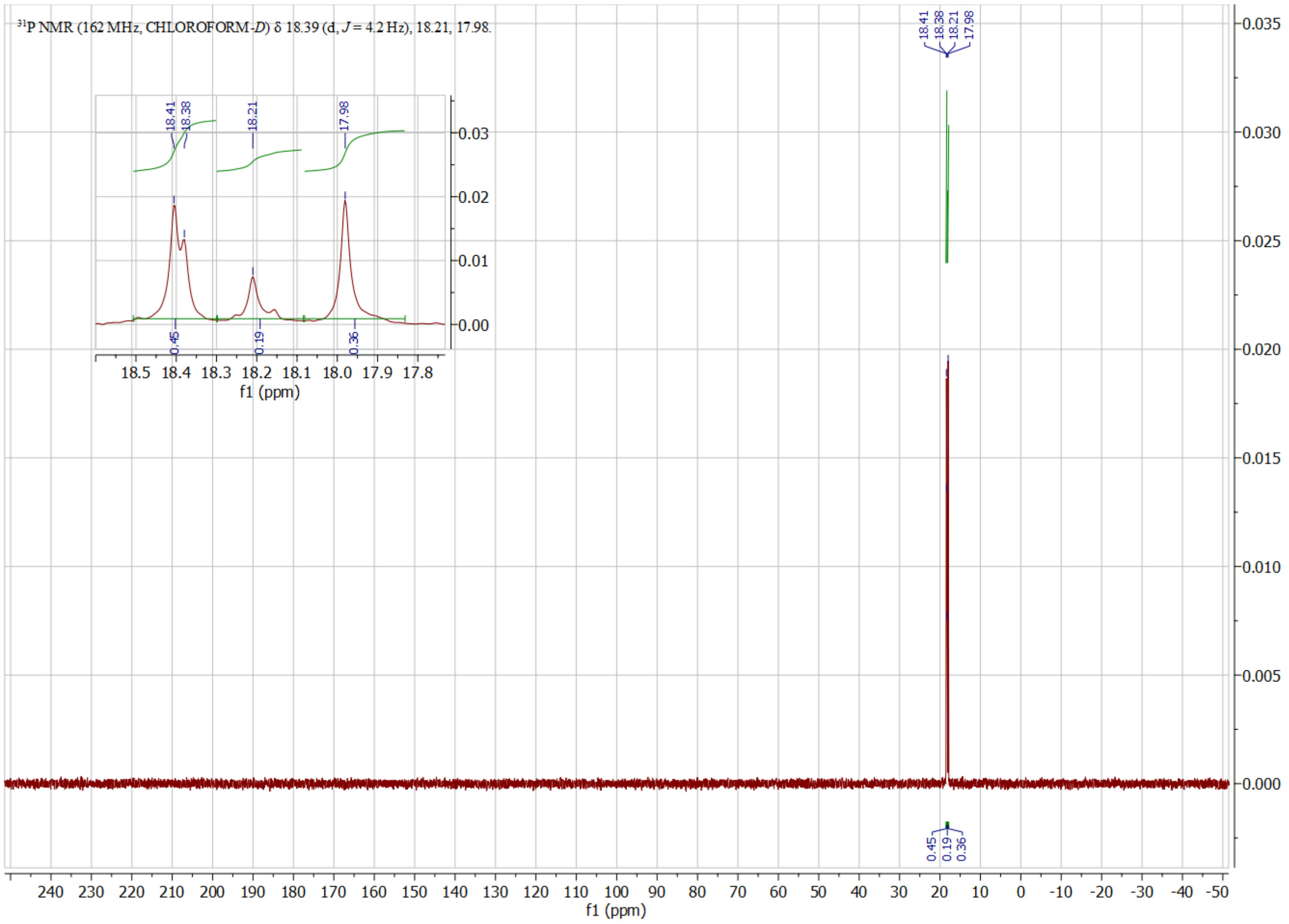

^{31}P NMR (162 MHz, CHLOROFORM-*D*) δ 18.39 (d, *J* = 4.2 Hz), 18.21, 17.98.
18.41
18.38
18.21
17.98
0.45
0.19
0.36
f1 (ppm)

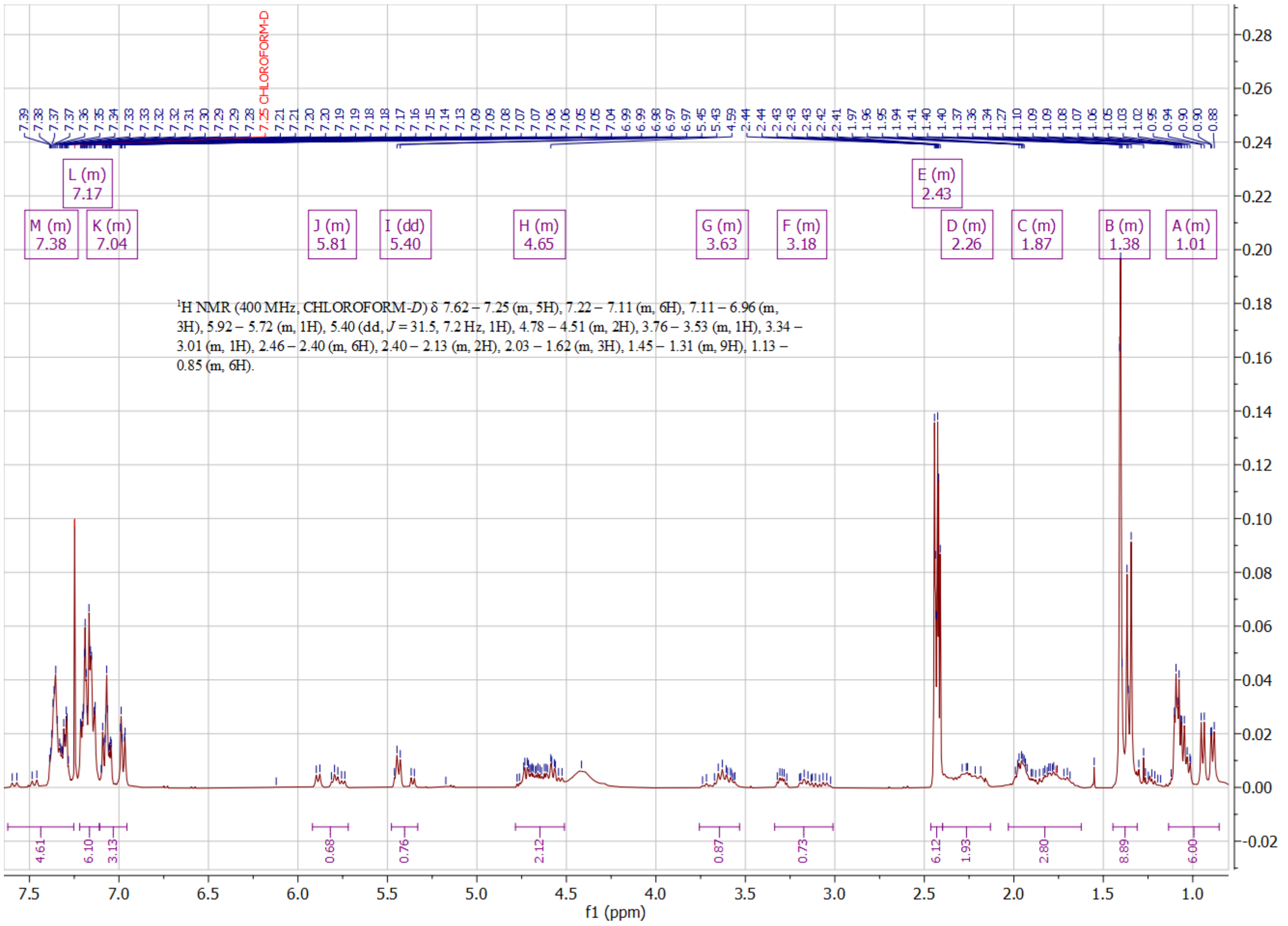

^{1}H NMR (400 MHz, CHLOROFORM-*D*) δ 7.62 – 7.25 (m, 5H), 7.22 – 7.11 (m, 6H), 7.11 – 6.96 (m, 3H), 5.92 – 5.72 (m, 1H), 5.40 (dd, *J* = 31.5, 7.2 Hz, 1H), 4.78 – 4.51 (m, 2H), 3.76 – 3.53 (m, 1H), 3.34 – 3.01 (m, 1H), 2.46 – 2.40 (m, 6H), 2.40 – 2.13 (m, 2H), 2.03 – 1.62 (m, 3H), 1.45 – 1.31 (m, 9H), 1.13 – 0.85 (m, 6H).
7.25 CHLOROFORM-D
L (m) 7.17
M (m) 7.38
K (m) 7.04
J (m) 5.81
I (dd) 5.40
H (m) 4.65
G (m) 3.63
F (m) 3.18
E (m) 2.43
D (m) 2.26
C (m) 1.87
B (m) 1.38
A (m) 1.01
4.61
6.10
3.13
0.68
0.76
2.12
0.87
0.73
6.12
1.93
2.80
8.89
6.00
f1 (ppm)

(90) Boc-D-Phg-L-Pro- Val^P(OPh-4-SCH$_3$)$_2$

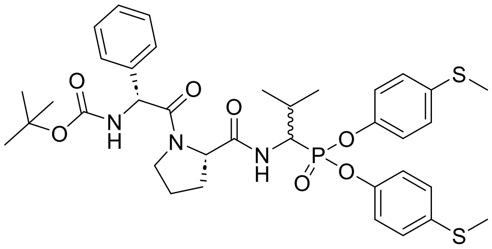

Name: *tert*-butyl ((1R)-2-((2S)-2-((1-(bis(4-(methylthio)phenoxy)phosphoryl)-2-methylpropyl)carbamoyl)pyrrolidin-1-yl)-2-oxo-1-phenylethyl)carbamate

^{31}P NMR (162 MHz, Chloroform-d) δ 18.46(s, 61%), 18.28 (s, 39%).

^{1}H NMR (400 MHz, Chloroform-d) δ 7.65 – 7.42 (m, 1H, NH), 7.42 – 7.27 (m, 5H, 5xArH), 7.22 – 7.14 (m, 6H, 6xArH), 7.13 – 7.03 (m, 2H, 2xArH), 5.77 (dd, J = 18.9, 7.6 Hz, 1H, NH), 5.40 (dd, J = 37.6, 7.5 Hz, 1H, CH), 4.79 – 4.64 (m, 1H, CHP), 4.64 – 4.50 (m, 1H, CH), 3.77 – 3.53 (m, 1H, 0.5xCH$_2$), 3.23 – 2.95 (m, 1H, 0.5xCH$_2$), 2.49 – 2.42 (m, 6H, 2xSCH$_3$), 2.42 – 1.86 (m, 3H, CH$_2$, CH), 1.86 – 1.59 (m, 2H, CH$_2$), 1.41 – 1.30 (m, 9H, Boc), 1.13 – 0.91 (m, 6H, 2xCH$_3$).

HRMS: $C_{36}H_{46}N_3O_7PS_2$ [M+Na]$^+$ calc. 750.2412 found 750.2410

HPLC: purity >99%; retention time 19.143 and 19.380 min

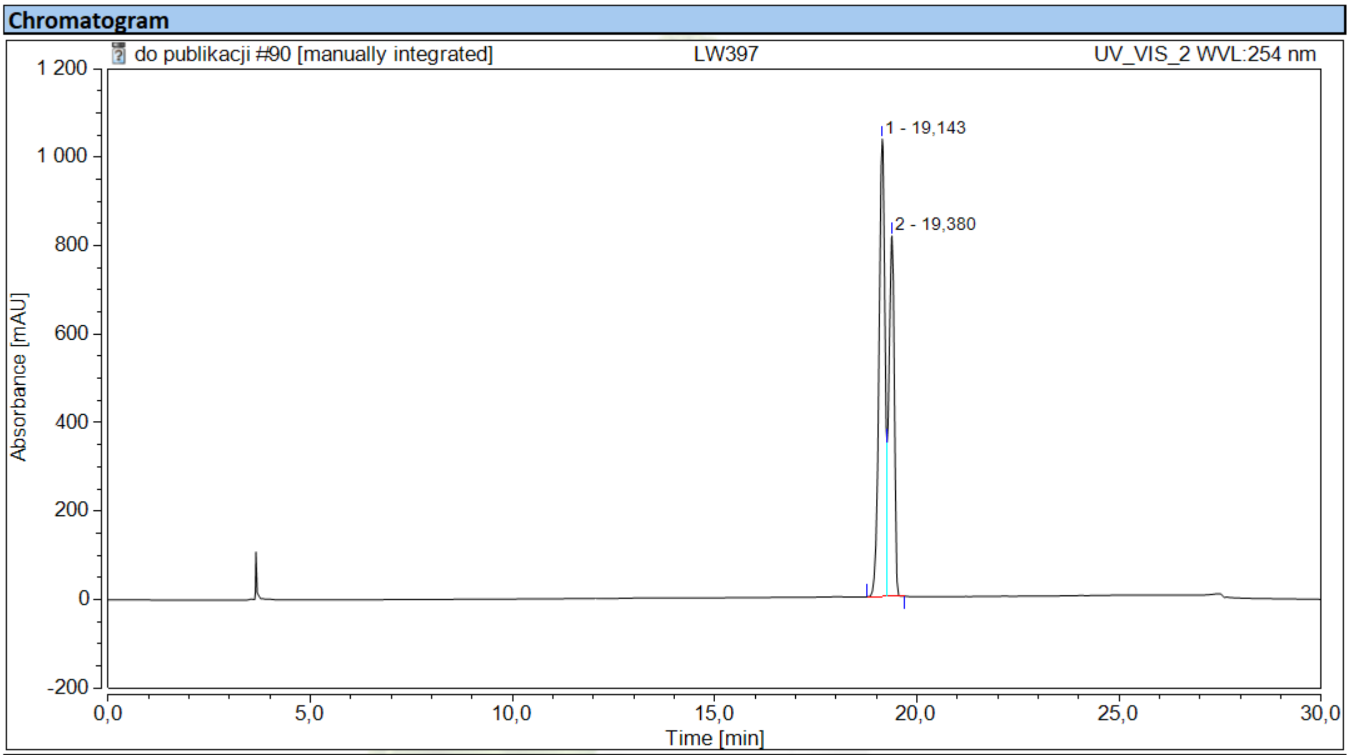

Integration Results

No.	Peak Name	Retention Time min	Area mAU*min	Height mAU	Relative Area %	Relative Height %	Amount n.a.
1		19,143	177,972	1034,360	58,38	55,93	n.a.
2		19,380	126,870	814,931	41,62	44,07	n.a.
**Total:**			**304,843**	**1849,291**	**100,00**	**100,00**	

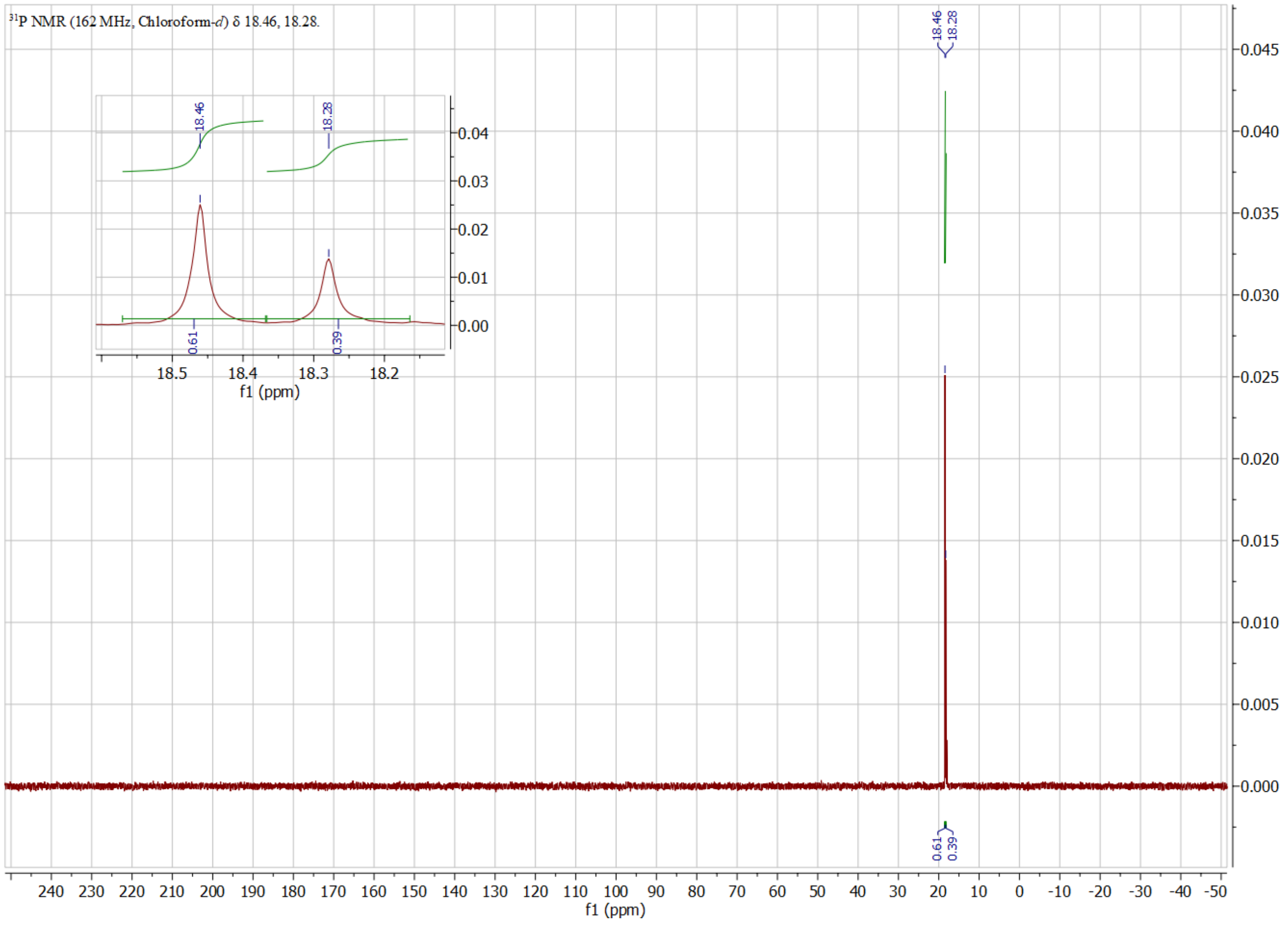
31P NMR (162 MHz, Chloroform-d) δ 18.46, 18.28.
18.46
18.28
f1 (ppm)

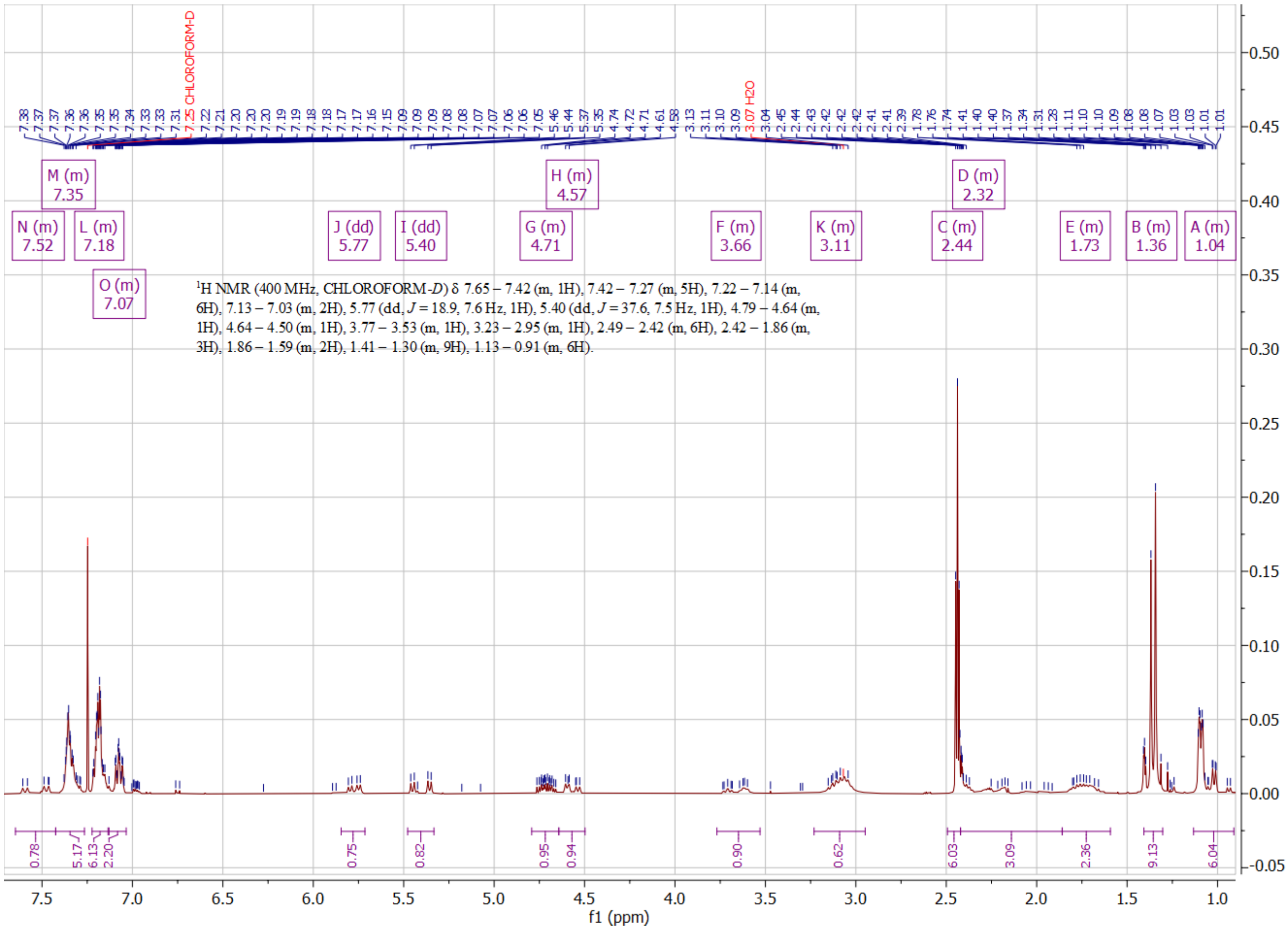
CHLOROFORM-D
H2O
M (m) 7.35
N (m) 7.52
L (m) 7.18
O (m) 7.07
J (dd) 5.77
I (dd) 5.40
H (m) 4.57
G (m) 4.71
F (m) 3.66
K (m) 3.11
D (m) 2.32
C (m) 2.44
E (m) 1.73
B (m) 1.36
A (m) 1.04
1H NMR (400 MHz, CHLOROFORM-D) δ 7.65 – 7.42 (m, 1H), 7.42 – 7.27 (m, 5H), 7.22 – 7.14 (m, 6H), 7.13 – 7.03 (m, 2H), 5.77 (dd, J = 18.9, 7.6 Hz, 1H), 5.40 (dd, J = 37.6, 7.5 Hz, 1H), 4.79 – 4.64 (m, 1H), 4.64 – 4.50 (m, 1H), 3.77 – 3.53 (m, 1H), 3.23 – 2.95 (m, 1H), 2.49 – 2.42 (m, 6H), 2.42 – 1.86 (m, 3H), 1.86 – 1.59 (m, 2H), 1.41 – 1.30 (m, 9H), 1.13 – 0.91 (m, 6H).
f1 (ppm)

(91) Boc-L-Chg-L-Pro-Val^P(OPh-4-SCH$_3$)$_2$

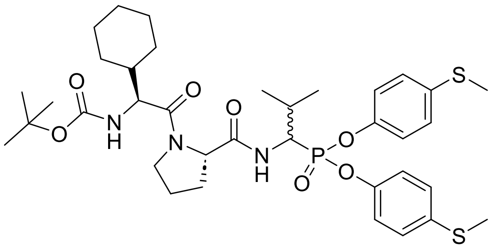

Name: *tert*-butyl ((1S)-2-((2S)-2-((1-(bis(4-(methylthio)phenoxy)phosphoryl)-2-methylpropyl)carbamoyl)pyrrolidin-1-yl)-1-cyclohexyl-2-oxoethyl)carbamate
^{31}P NMR (162 MHz, Chloroform-d) δ 18.39 (s, 46%), 17.90 (s, 54%).
^{1}H NMR (400 MHz, Chloroform-d) δ 7.35 (dd, J = 35.8, 10.1 Hz, 1H, NH), 7.23 – 7.02 (m, 8H, 8xArH), 5.35 (dd, J = 19.6, 9.2 Hz, 1H, NH), 4.79 – 4.68 (m, 1H, CHP), 4.68 – 4.47 (m, 1H, CH), 4.30 – 4.21 (m, 1H, CH), 3.86 – 3.74 (m, 1H, 0.5xCH$_2$), 3.67 – 3.55 (m, 1H, 0.5xCH$_2$), 2.47 – 2.42 (m, 6H, 2xSCH$_3$), 2.42 – 2.30 (m, 1H, CH), 2.30 – 1.98 (m, 3H, CH$_2$, CH), 1.98 – 1.47 (m, 7H, 3.5xCH$_2$), 1.41 (s, 9H, Boc), 1.26 – 0.86 (m, 11H, 2xCH$_3$, 2.5xCH$_2$).
HRMS: $C_{36}H_{52}N_3O_7PS_2$ [M+Na]$^+$ calc. 756.2882 found 756.2870
HPLC: purity >99%; retention time 20.460 min

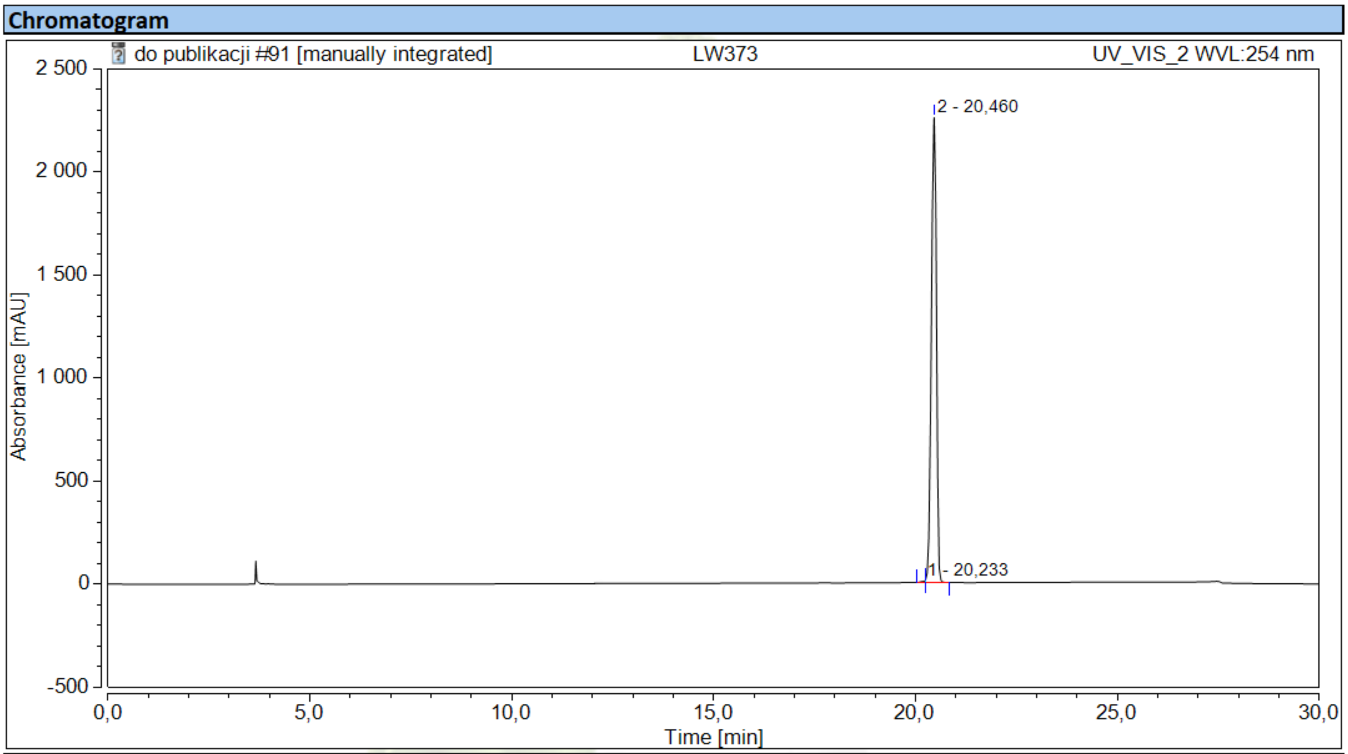

Integration Results

No.	Peak Name	Retention Time min	Area mAU*min	Height mAU	Relative Area %	Relative Height %	Amount n.a.
1		20,233	0,614	7,025	0,19	0,31	n.a.
2		20,460	329,174	2254,333	99,81	99,69	n.a.
**Total:**			**329,789**	**2261,359**	**100,00**	**100,00**	

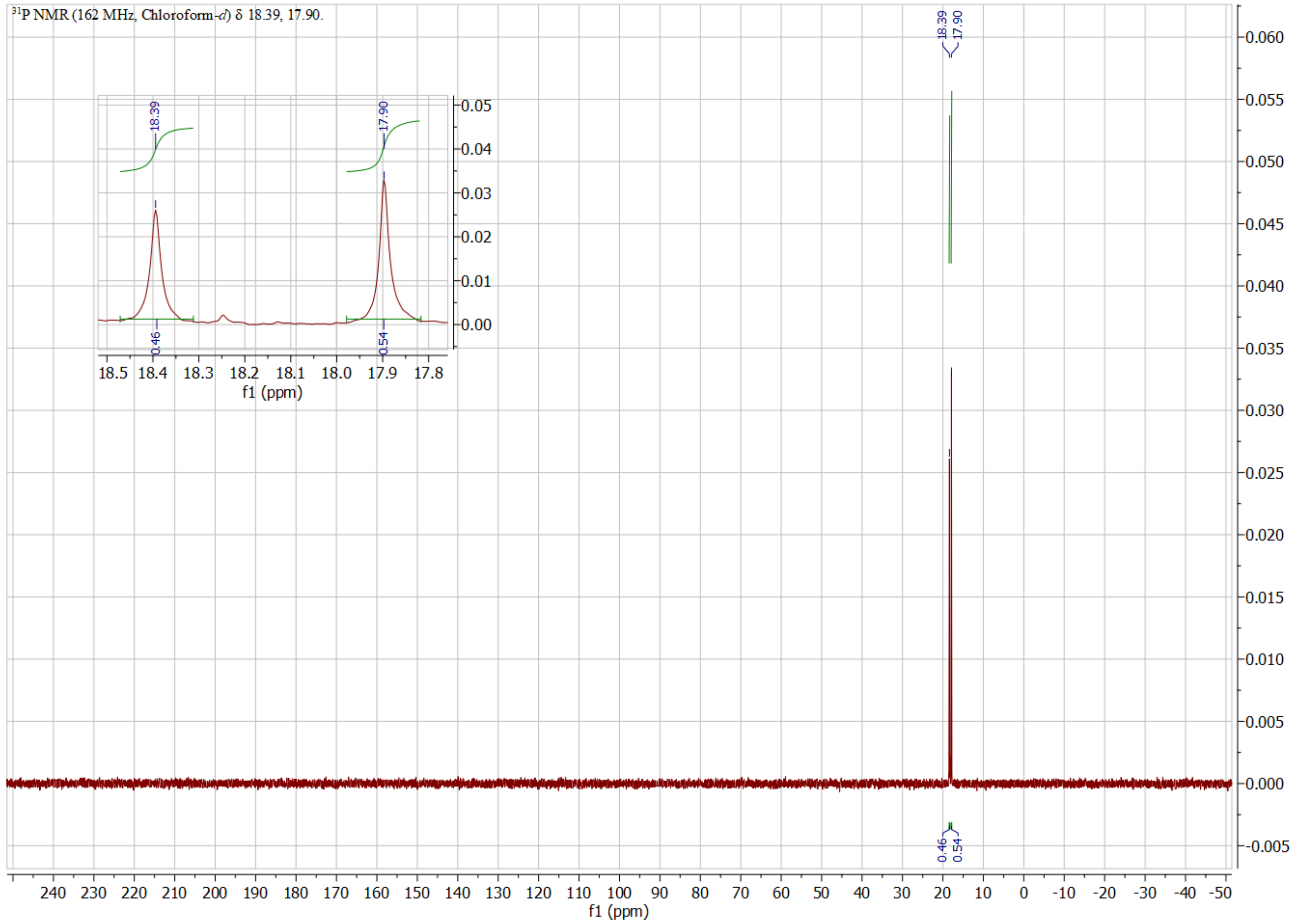

^{31}P NMR (162 MHz, Chloroform-*d*) δ 18.39, 17.90.
f1 (ppm)

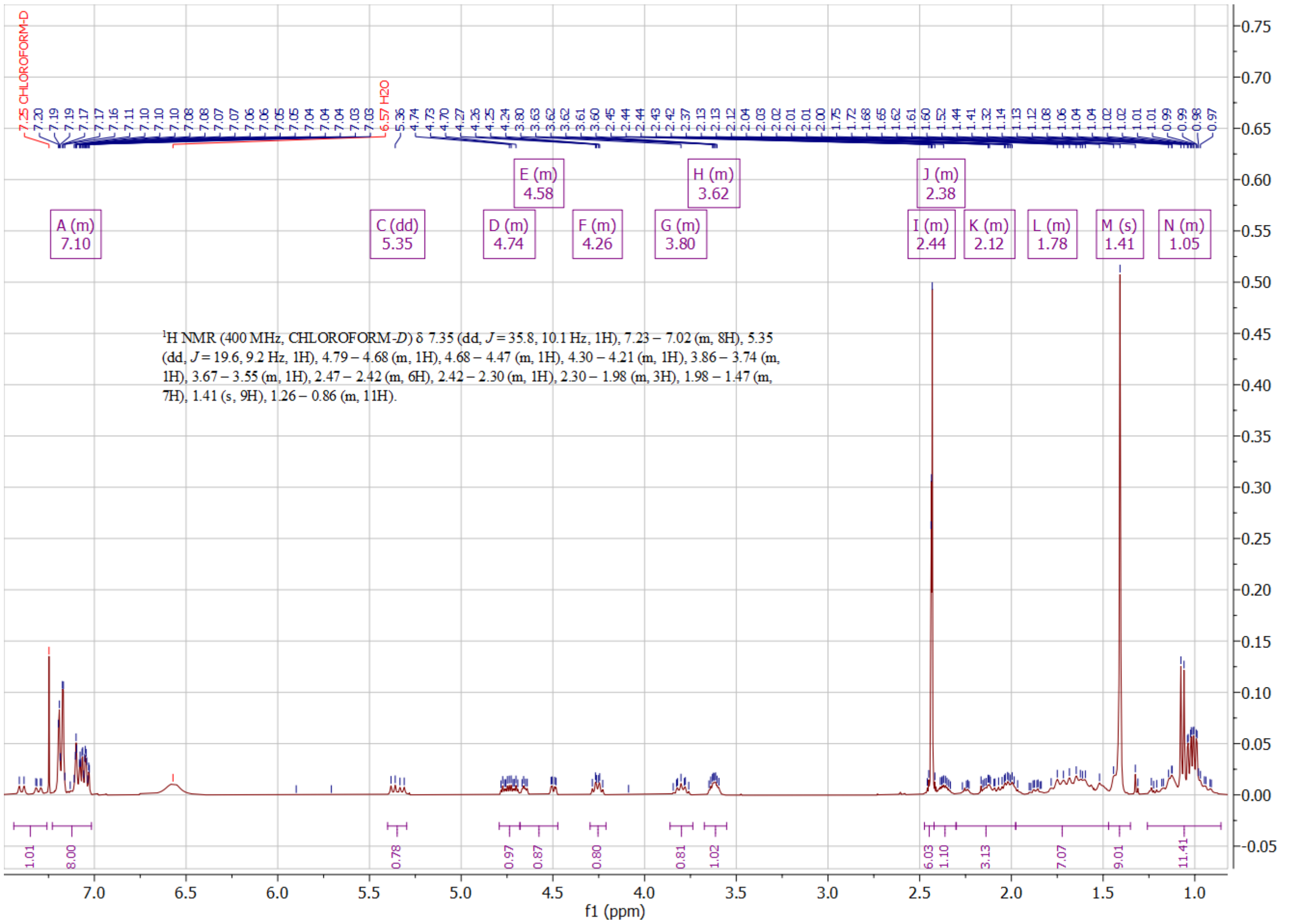

^{1}H NMR (400 MHz, CHLOROFORM-*D*) δ 7.35 (dd, *J* = 35.8, 10.1 Hz, 1H), 7.23 – 7.02 (m, 8H), 5.35 (dd, *J* = 19.6, 9.2 Hz, 1H), 4.79 – 4.68 (m, 1H), 4.68 – 4.47 (m, 1H), 4.30 – 4.21 (m, 1H), 3.86 – 3.74 (m, 1H), 3.67 – 3.55 (m, 1H), 2.47 – 2.42 (m, 6H), 2.42 – 2.30 (m, 1H), 2.30 – 1.98 (m, 3H), 1.98 – 1.47 (m, 7H), 1.41 (s, 9H), 1.26 – 0.86 (m, 11H).
f1 (ppm)

(92) Boc-D-Chg-L-Pro-Val^{P}(OPh-4-SCH$_3$)$_2$

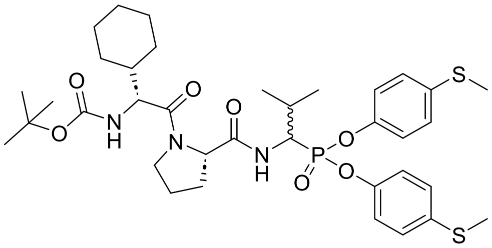

Name: *tert*-butyl ((1R)-2-((2S)-2-((1-(bis(4-(methylthio)phenoxy)phosphoryl)-2-methylpropyl)carbamoyl)pyrrolidin-1-yl)-1-cyclohexyl-2-oxoethyl)carbamate

^{31}P NMR (162 MHz, Chloroform-d) δ 18.19 (s, 55%), 18.04 (s, 45%).

^{1}H NMR (400 MHz, Chloroform-d) δ 7.54 (dd, J = 112.7, 10.2 Hz, 1H, NH), 7.23 – 7.15 (m, 4H, 4xArH), 7.14 – 6.96 (m, 4H, 4xArH), 5.37 – 5.25 (m, 1H, NH), 4.77 – 4.64 (m, 1H, CHP), 4.59 (dd, J = 26.1, 8.1 Hz, 1H, CH), 4.34 – 4.17 (m, 1H, CH), 3.95 – 3.82 (m, 1H, 0.5xCH$_2$), 3.62 – 3.46 (m, 1H, 0.5xCH$_2$), 2.43 (d, J = 3.1 Hz, 6H, 2xSCH$_3$), 2.40 – 2.24 (m, 1H, CH), 2.24 – 1.94 (m, 2H, 0.5xCH$_2$, CH), 1.94 – 1.52 (m, 8H, 4xCH$_2$), 1.46 – 1.30 (m, 9H, Boc), 1.29 – 0.93 (m, 11H, 2xCH$_3$, 2.5xCH$_2$).

HRMS: $C_{36}H_{52}N_3O_7PS_2$ $[M+Na]^+$ calc. 756.2882 found 756.2872

HPLC: purity >99%; retention time 20.387 min

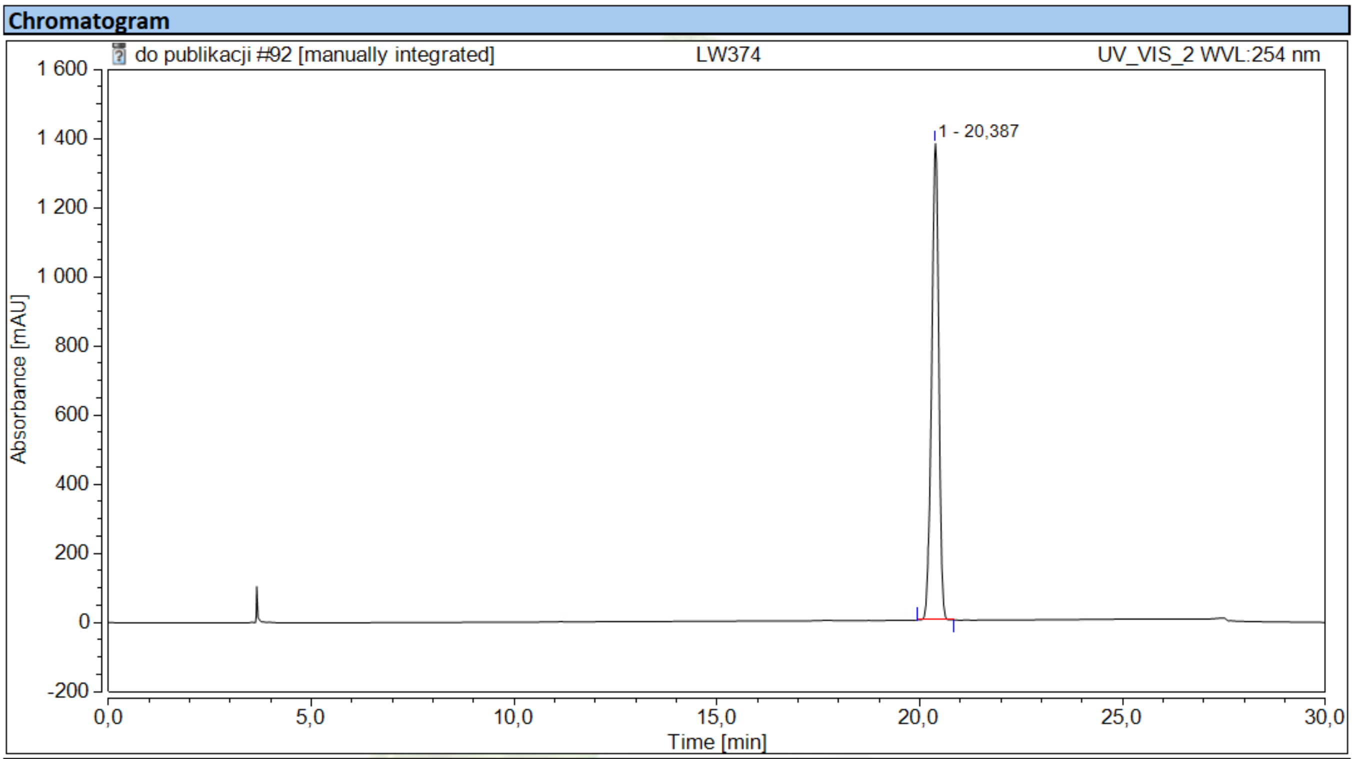

Integration Results

No.	Peak Name	Retention Time min	Area mAU*min	Height mAU	Relative Area %	Relative Height %	Amount n.a.
1		20,387	280,330	1377,415	100,00	100,00	n.a.
**Total:**			**280,330**	**1377,415**	**100,00**	**100,00**	

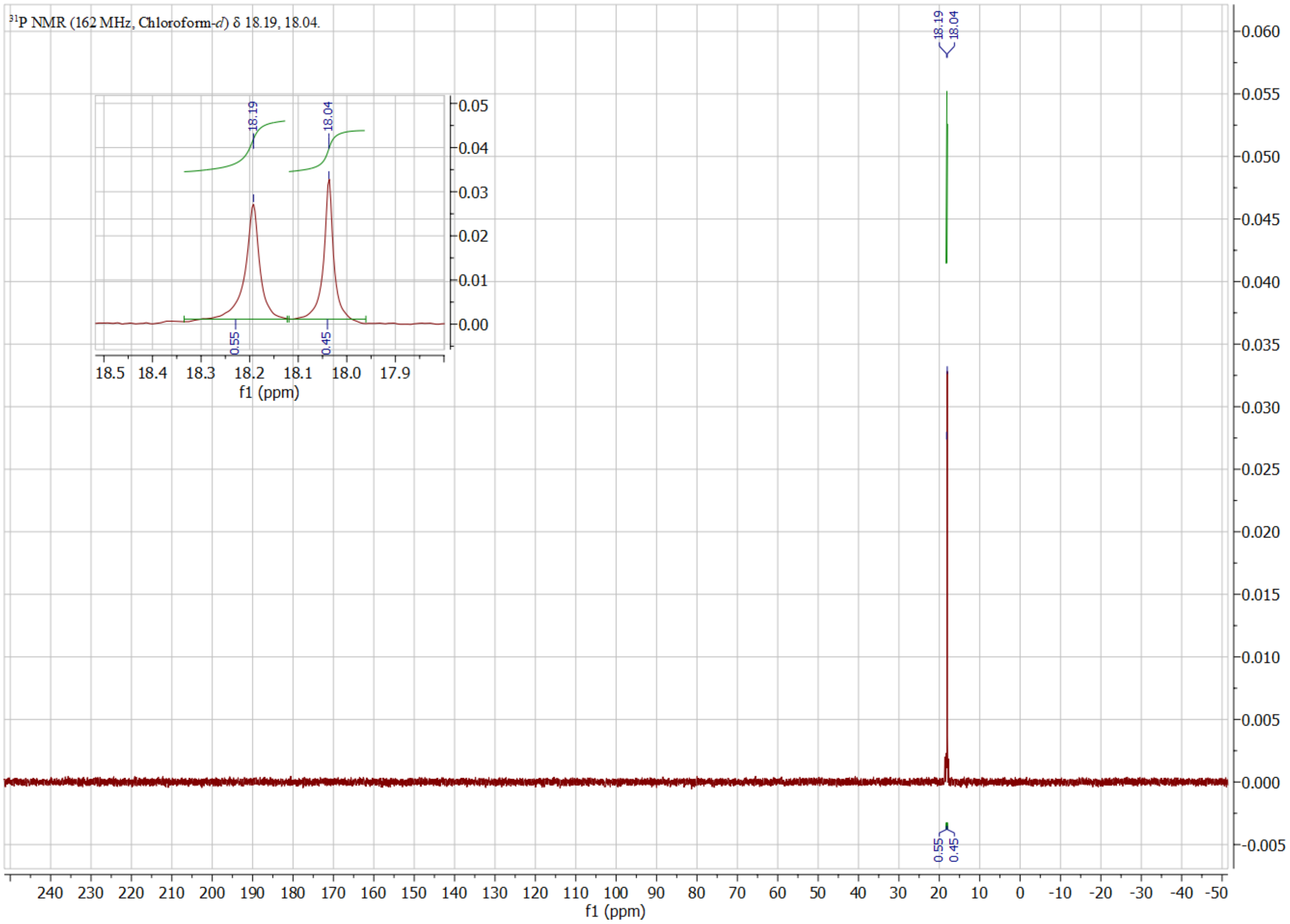

31P NMR (162 MHz, Chloroform-d) δ 18.19, 18.04.
f1 (ppm)

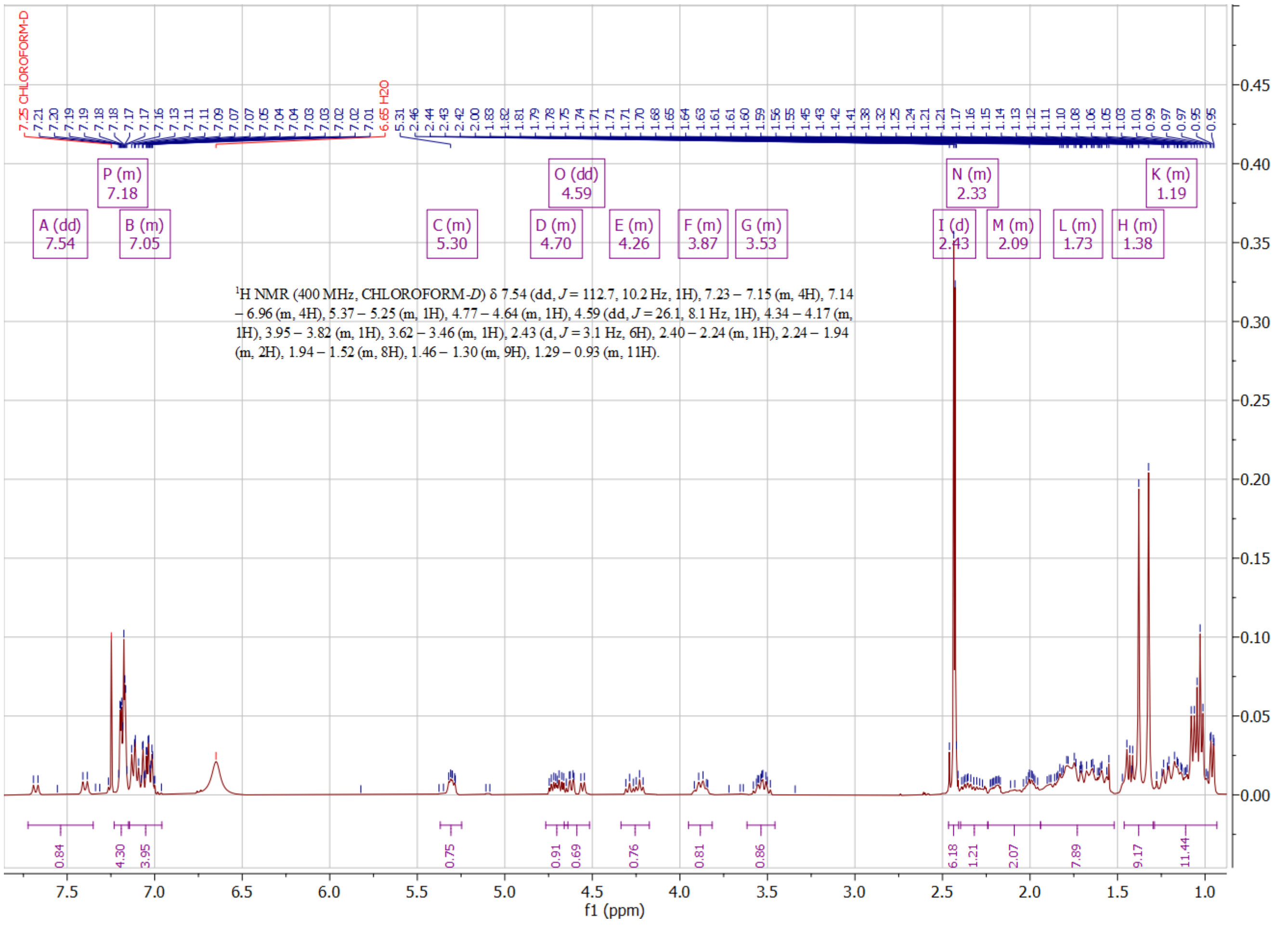

1H NMR (400 MHz, CHLOROFORM-D) δ 7.54 (dd, J = 112.7, 10.2 Hz, 1H), 7.23 – 7.15 (m, 4H), 7.14 – 6.96 (m, 4H), 5.37 – 5.25 (m, 1H), 4.77 – 4.64 (m, 1H), 4.59 (dd, J = 26.1, 8.1 Hz, 1H), 4.34 – 4.17 (m, 1H), 3.95 – 3.82 (m, 1H), 3.62 – 3.46 (m, 1H), 2.43 (d, J = 3.1 Hz, 6H), 2.40 – 2.24 (m, 1H), 2.24 – 1.94 (m, 2H), 1.94 – 1.52 (m, 8H), 1.46 – 1.30 (m, 9H), 1.29 – 0.93 (m, 11H).
7.25 CHLOROFORM-D
6.65 H2O
f1 (ppm)

(93) Boc-L-Phe-L-Pro-Val^{P}(OPh-4-SCH$_3$)$_2$

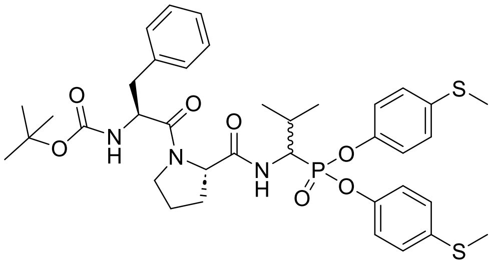

Name: *tert*-butyl ((2S)-1-((2S)-2-((1-(bis(4-(methylthio)phenoxy)phosphoryl)-2-methylpropyl)carbamoyl)pyrrolidin-1-yl)-1-oxo-3-phenylpropan-2-yl)carbamate
^{31}P NMR (162 MHz, Chloroform-d) δ 18.43 (s, 44%), 18.02 (s, 56%).
^{1}H NMR (400 MHz, Chloroform-d) δ 7.69 (dd, J = 51.4, 9.7 Hz, 1H, NH), 7.33 – 7.22 (m, 2H, 2xArH), 7.22 – 7.16 (m, 5H, 5xArH), 7.15 – 7.04 (m, 6H, 6xArH), 5.32 (dd, J = 57.6, 8.4 Hz, 1H, NH), 4.84 – 4.45 (m, 3H, CHP, 2xCH), 3.76 – 3.58 (m, 1H, 0.5xCH$_2$), 3.48 – 3.17 (m, 1H, 0.5xCH$_2$), 3.12 – 2.82 (m, 1H, 0.5xCH$_2$), 2.82 – 2.56 (m, 1H, 0.5xCH$_2$), 2.43 (d, J = 7.7 Hz, 6H, 2xSCH$_3$), 2.40 – 2.32 (m, 2H, 0.5xCH$_2$, CH), 2.11 – 1.82 (m, 3H, 1.5xCH$_2$), 1.41 – 1.28 (m, 9H, Boc), 1.16 – 1.02 (m, 6H, 2xCH$_3$).
HRMS: $C_{37}H_{48}N_3O_7PS_2$ $[M+Na]^+$ calc. 764.2569 found 764.2561
HPLC: purity >99%; retention time 19.247 and 19.457 min

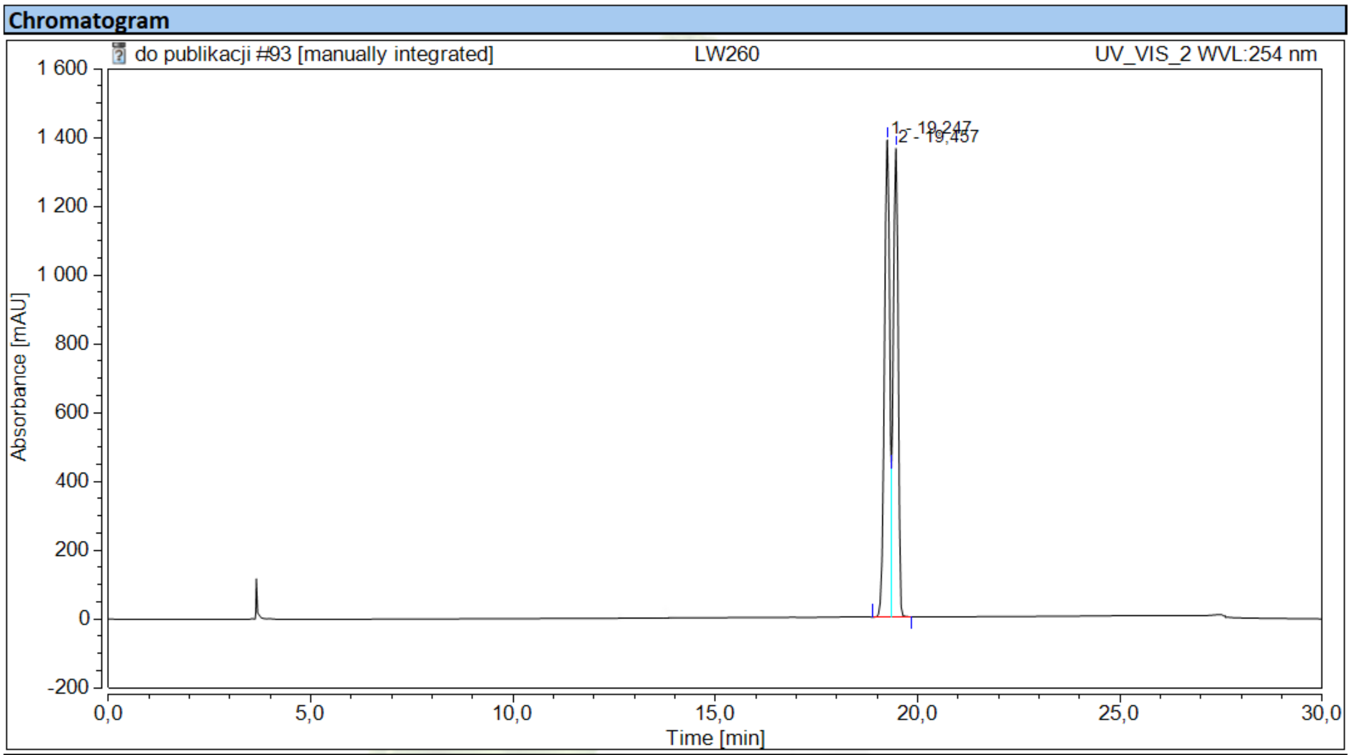

Integration Results

No.	Peak Name	Retention Time min	Area mAU*min	Height mAU	Relative Area %	Relative Height %	Amount n.a.
1		19,247	209,024	1387,055	51,91	50,45	n.a.
2		19,457	193,614	1362,307	48,09	49,55	n.a.
**Total:**			**402,639**	**2749,362**	**100,00**	**100,00**	

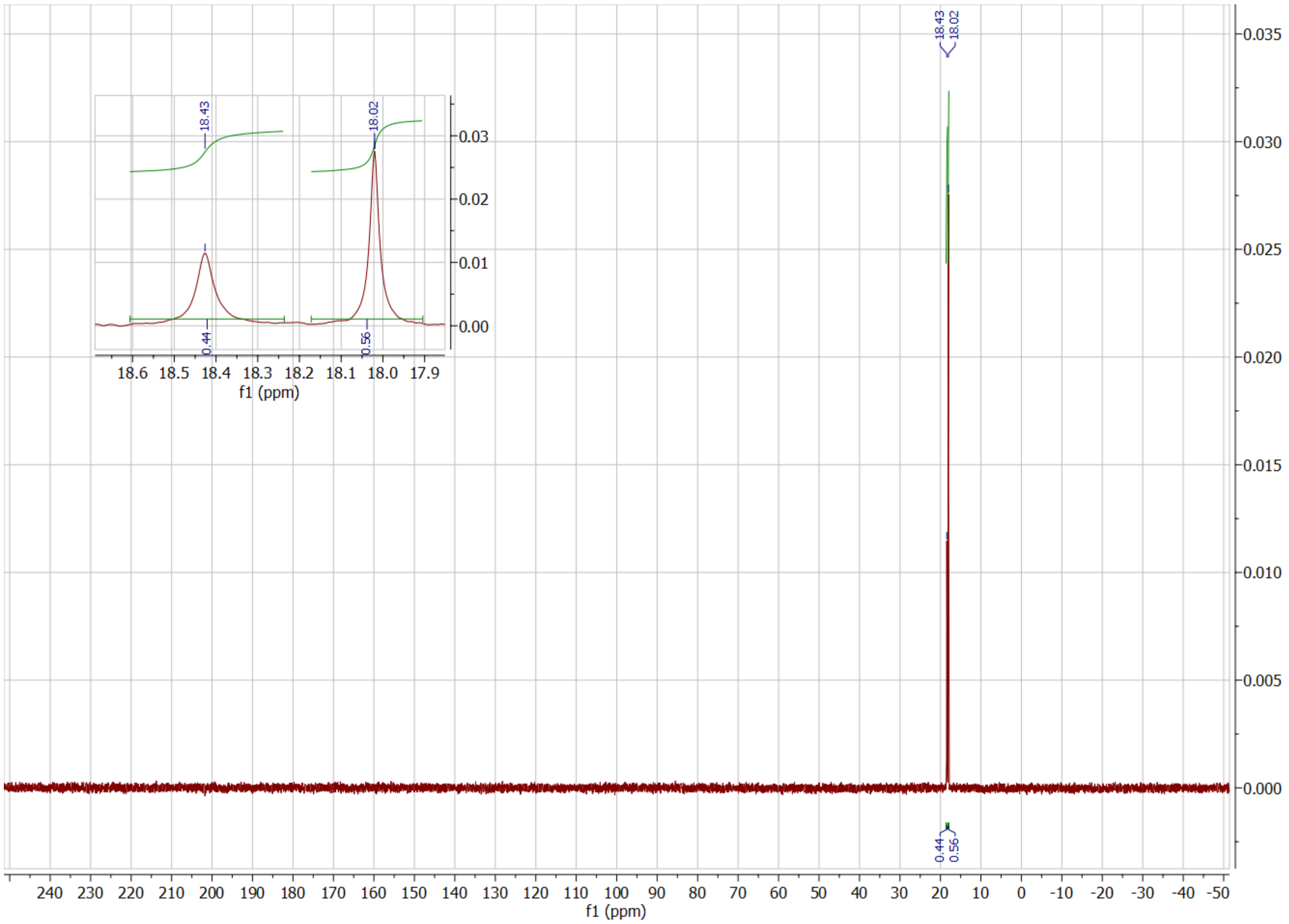
18.43
18.02
0.44
0.56
f1 (ppm)

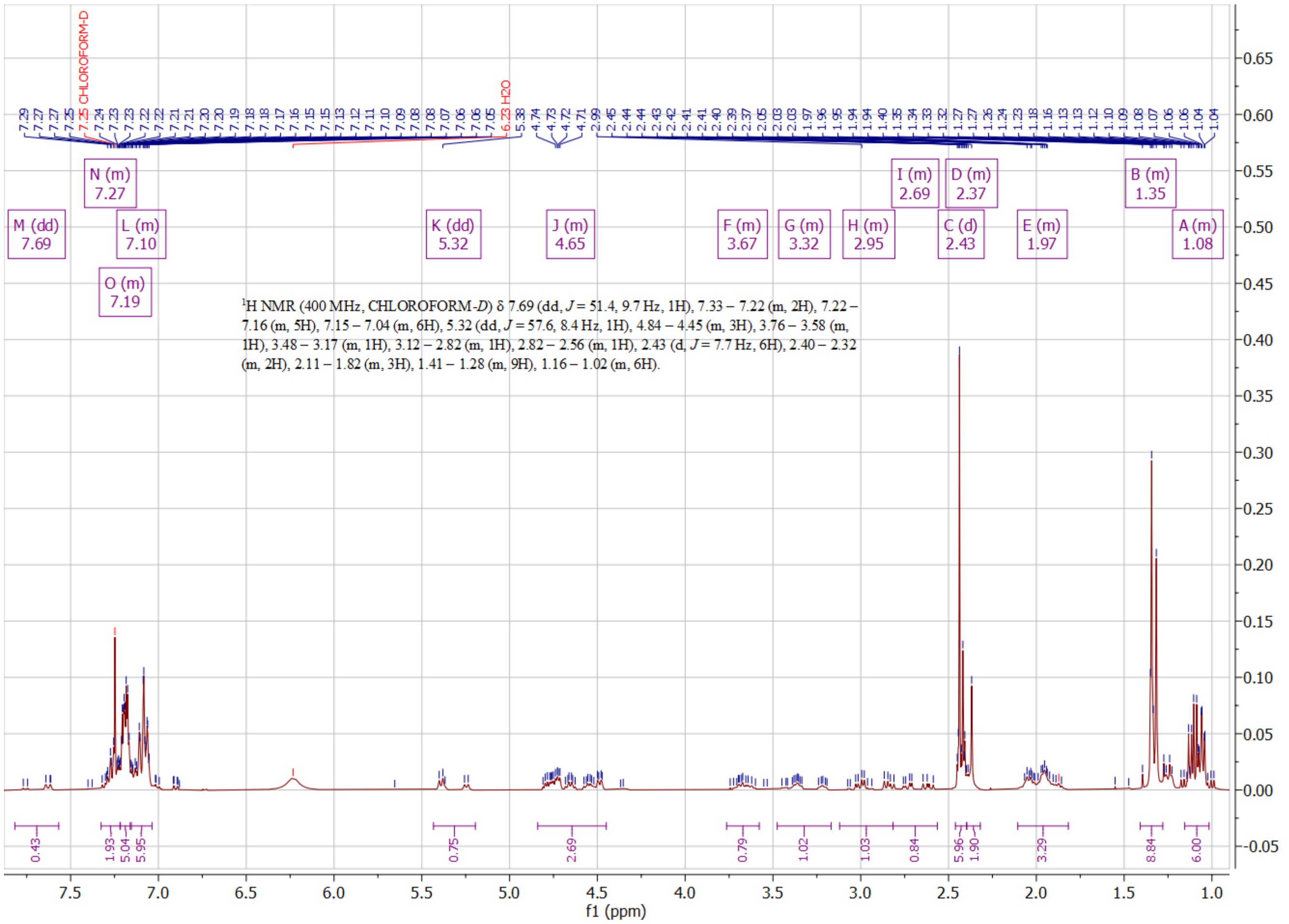
7.25 CHLOROFORM-D
6.23 H2O
M (dd) 7.69
N (m) 7.27
O (m) 7.19
L (m) 7.10
K (dd) 5.32
J (m) 4.65
F (m) 3.67
G (m) 3.32
H (m) 2.95
I (m) 2.69
D (m) 2.37
C (d) 2.43
E (m) 1.97
B (m) 1.35
A (m) 1.08
^{1}H NMR (400 MHz, CHLOROFORM-*D*) δ 7.69 (dd, *J* = 51.4, 9.7 Hz, 1H), 7.33 – 7.22 (m, 2H), 7.22 – 7.16 (m, 5H), 7.15 – 7.04 (m, 6H), 5.32 (dd, *J* = 57.6, 8.4 Hz, 1H), 4.84 – 4.45 (m, 3H), 3.76 – 3.58 (m, 1H), 3.48 – 3.17 (m, 1H), 3.12 – 2.82 (m, 1H), 2.82 – 2.56 (m, 1H), 2.43 (d, *J* = 7.7 Hz, 6H), 2.40 – 2.32 (m, 2H), 2.11 – 1.82 (m, 3H), 1.41 – 1.28 (m, 9H), 1.16 – 1.02 (m, 6H).
f1 (ppm)

(94) Boc-D-Phe-L-Pro- Val^P(OPh-4-SCH$_3$)$_2$

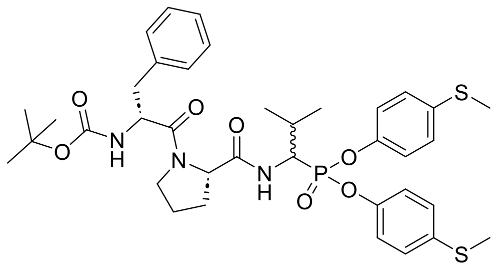

Name: *tert*-butyl ((2R)-1-((2S)-2-((1-(bis(4-(methylthio)phenoxy)phosphoryl)-2-methylpropyl)carbamoyl)pyrrolidin-1-yl)-1-oxo-3-phenylpropan-2-yl)carbamate

^{31}P NMR (162 MHz, Chloroform-d) δ 18.38 (s, 56%), 18.17 (s, 44%).

^{1}H NMR (400 MHz, Chloroform-d) δ 7.53 (dd, J = 109.7, 10.2 Hz, 1H, NH), 7.31 – 7.25 (m, 2H, 2xArH), 7.24 – 7.09 (m, 9H, 9xArH), 7.09 – 6.98 (m, 2H, 2xArH), 5.38 (d, J = 7.9 Hz, 1H, NH), 4.71 – 4.49 (m, 2H, CHP, CH), 4.49 – 4.31 (m, 1H, CH), 3.58 – 3.39 (m, 1H, 0.5xCH$_2$), 3.05 – 2.90 (m, 2H, CH$_2$), 2.62 – 2.47 (m, 1H, 0.5xCH$_2$), 2.47 – 2.41 (m, 6H, 2xSCH$_3$), 2.41 – 2.28 (m, 1H, CH), 2.20 – 2.00 (m, 1H, 0.5xCH$_2$), 1.90 – 1.39 (m, 3H, 1.5xCH$_2$), 1.35 (d, J = 17.6 Hz, 9H, Boc), 1.14 – 0.92 (m, 6H, 2xCH$_3$).

HRMS: $C_{37}H_{48}N_3O_7PS_2$ [M+Na]$^+$ calc. 764.2569 found 764.2543

HPLC: purity >99%; retention time 19.477 min

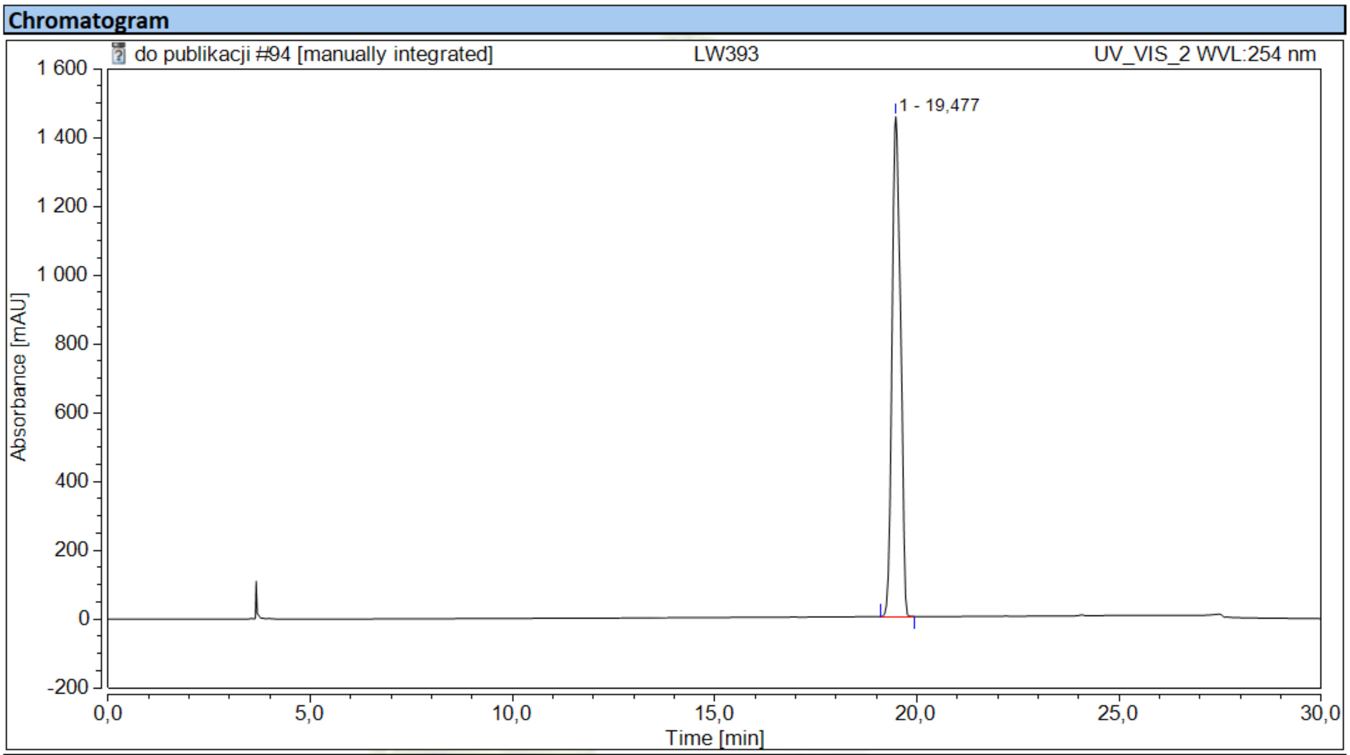

Integration Results

No.	Peak Name	Retention Time min	Area mAU*min	Height mAU	Relative Area %	Relative Height %	Amount n.a.
1		19,477	353,427	1453,217	100,00	100,00	n.a.
**Total:**			**353,427**	**1453,217**	**100,00**	**100,00**	

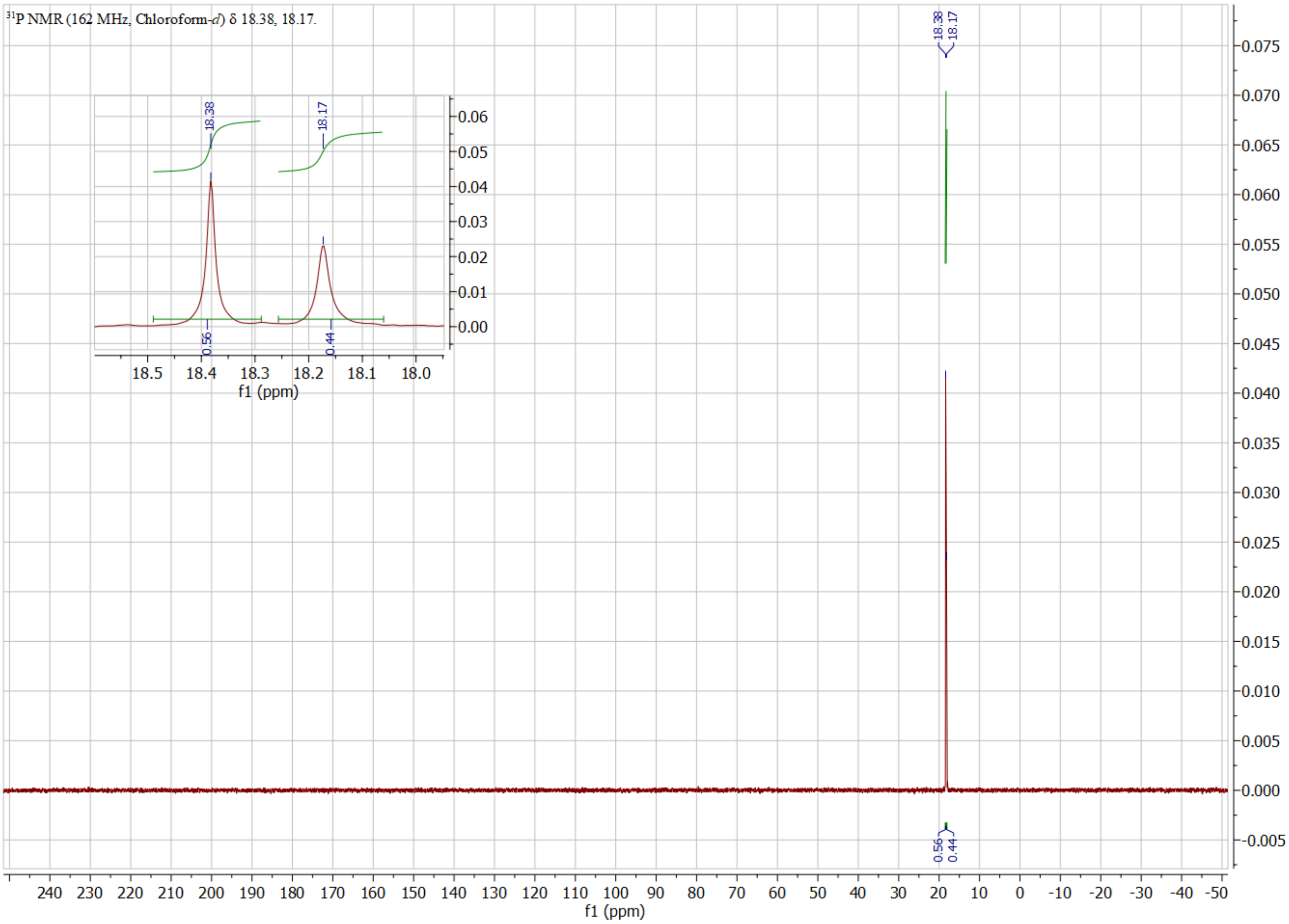

^{31}P NMR (162 MHz, Chloroform-d) δ 18.38, 18.17.
f1 (ppm)

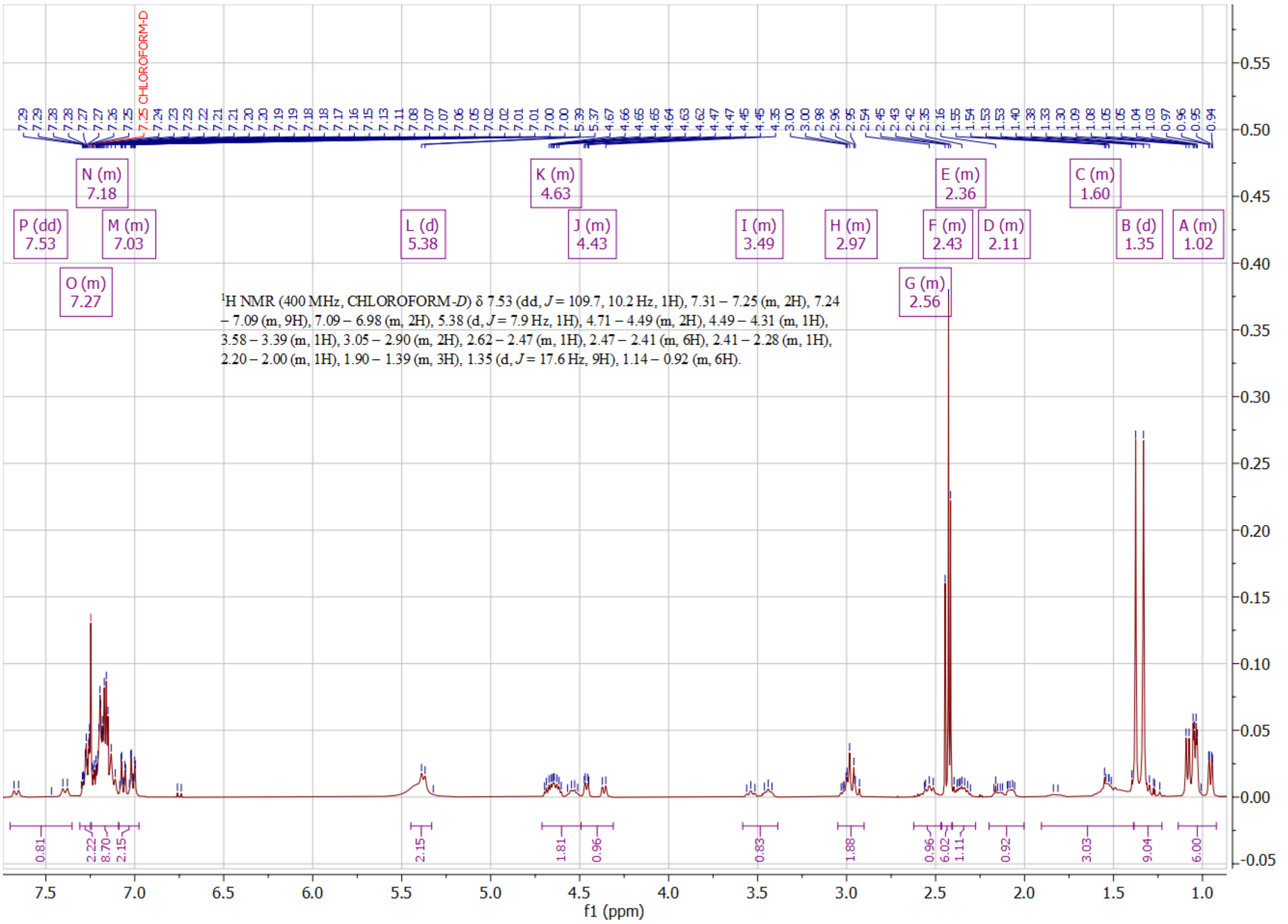

^{1}H NMR (400 MHz, CHLOROFORM-D) δ 7.53 (dd, J = 109.7, 10.2 Hz, 1H), 7.31 – 7.25 (m, 2H), 7.24 – 7.09 (m, 9H), 7.09 – 6.98 (m, 2H), 5.38 (d, J = 7.9 Hz, 1H), 4.71 – 4.49 (m, 2H), 4.49 – 4.31 (m, 1H), 3.58 – 3.39 (m, 1H), 3.05 – 2.90 (m, 2H), 2.62 – 2.47 (m, 1H), 2.47 – 2.41 (m, 6H), 2.41 – 2.28 (m, 1H), 2.20 – 2.00 (m, 1H), 1.90 – 1.39 (m, 3H), 1.35 (d, J = 17.6 Hz, 9H), 1.14 – 0.92 (m, 6H).
CHLOROFORM-D
f1 (ppm)

(95) Boc-L-Cha-L-Pro-Val^P(OPh-4-SCH$_3$)$_2$

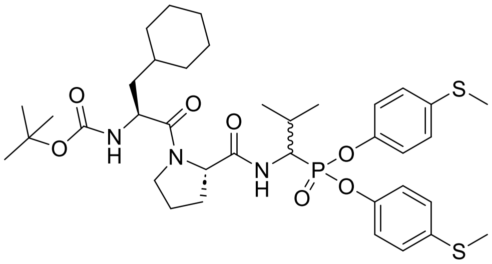

Name: *tert*-butyl ((2S)-1-((2S)-2-((1-(bis(4-(methylthio)phenoxy)phosphoryl)-2-methylpropyl)carbamoyl)pyrrolidin-1-yl)-3-cyclohexyl-1-oxopropan-2-yl)carbamate
^{31}P NMR (162 MHz, Chloroform-d) δ 18.34 (s, 49%), 17.96 (s, 51%).
^{1}H NMR (400 MHz, Chloroform-d) δ 7.40 (dd, J = 34.9, 10.8 Hz, 1H, NH), 7.22 – 7.15 (m, 4H, 4xArH), 7.15 – 6.99 (m, 4H, 4xArH), 5.23 (dd, J = 45.7, 8.8 Hz, 1H, NH), 4.78 – 4.36 (m, 3H, CHP, 2xCH), 3.78 – 3.63 (m, 1H, 0.5xCH$_2$), 3.58 – 3.41 (m, 1H, 0.5xCH$_2$), 2.45 – 2.40 (m, 6H, 2xSCH$_3$), 2.40 – 2.32 (m, 1H, CH), 2.19 – 1.89 (m, 3H, 1.5xCH$_2$), 1.89 – 1.79 (m, 1H, CH), 1.79 – 1.51 (m, 4H, 2xCH$_2$), 1.41 (d, J = 1.5 Hz, 9H, Boc), 1.40 – 1.09 (m, 7H, 3.5xCH$_2$), 1.08 – 0.96 (m, 6H, 2xCH$_3$), 0.95 – 0.57 (m, 2H, CH$_2$).
HRMS: $C_{37}H_{54}N_3O_7PS_2$ [M+Na]$^+$ calc. 770.3038 found 770.3029
HPLC: purity >97%; retention time 21.160 min

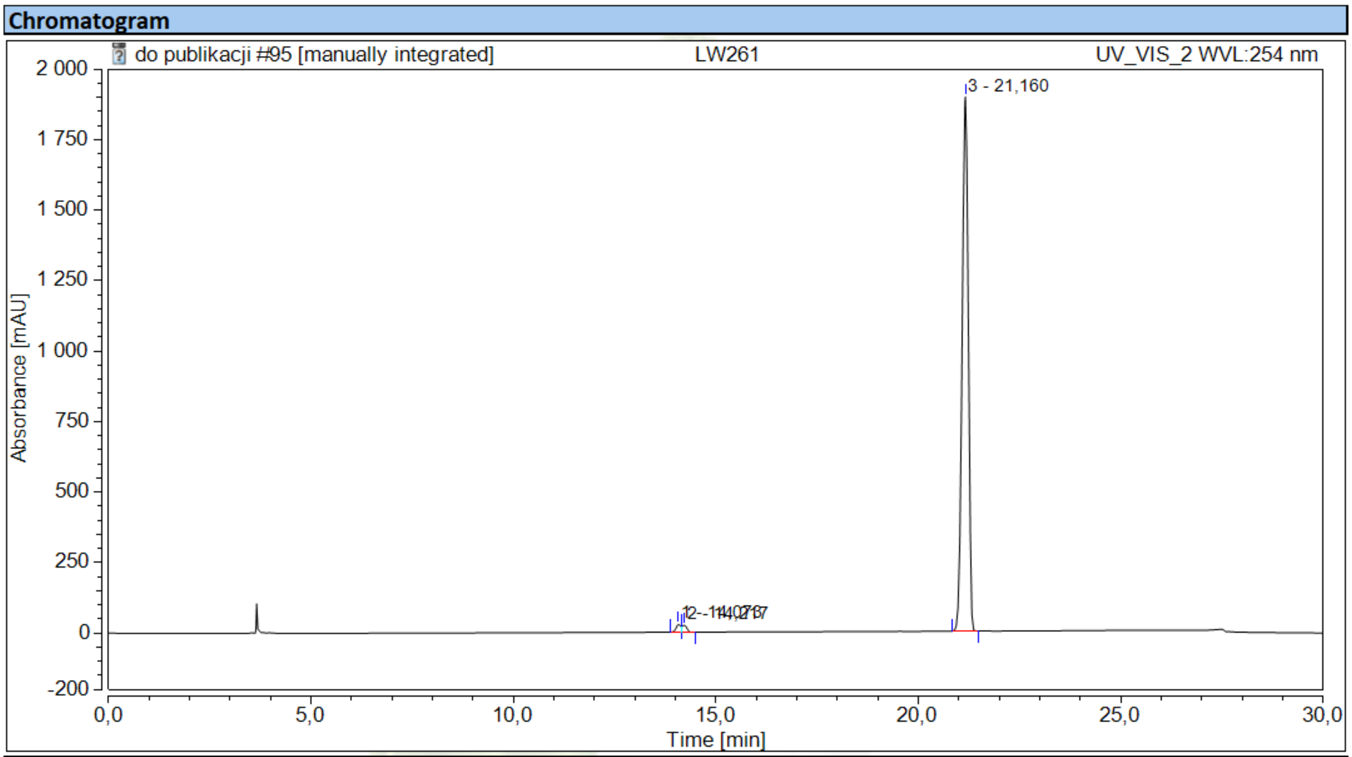

Integration Results

No.	Peak Name	Retention Time min	Area mAU*min	Height mAU	Relative Area %	Relative Height %	Amount n.a.
1		14,073	3,541	28,252	1,04	1,45	n.a.
2		14,217	3,477	24,990	1,02	1,28	n.a.
3		21,160	333,703	1894,451	97,94	97,27	n.a.
**Total:**			**340,722**	**1947,693**	**100,00**	**100,00**	

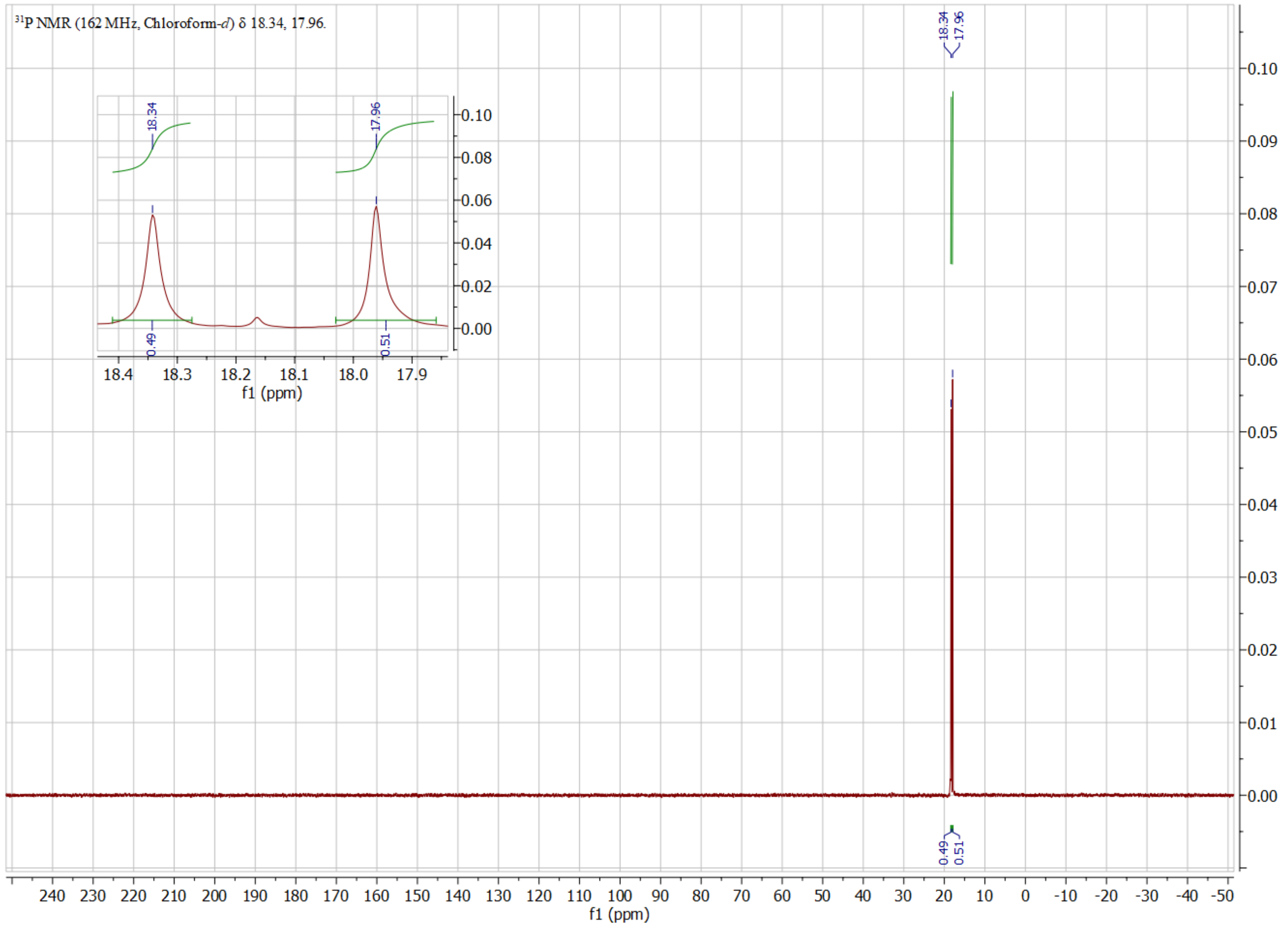
^{31}P NMR (162 MHz, Chloroform-*d*) δ 18.34, 17.96.
f1 (ppm)

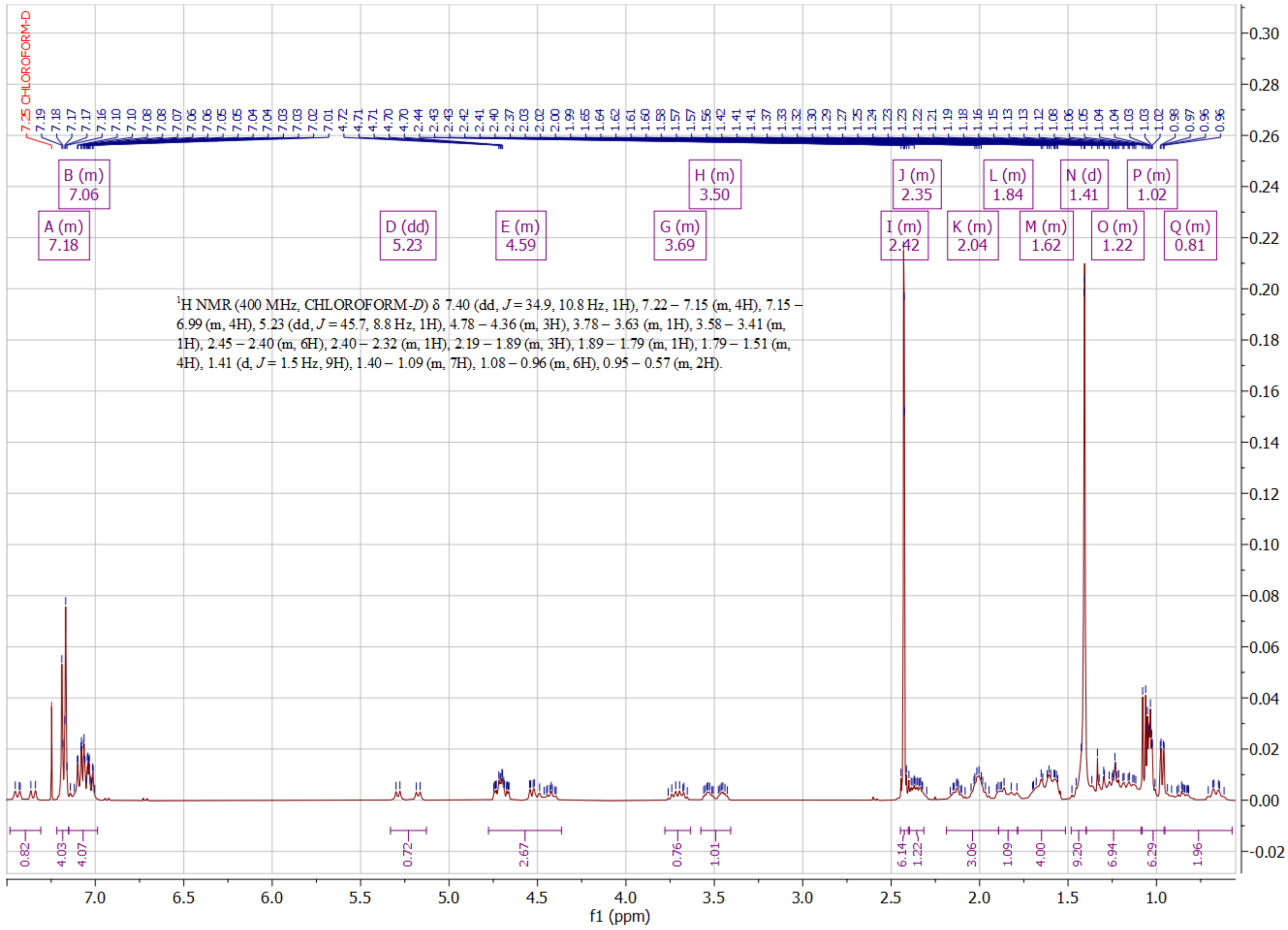
^{1}H NMR (400 MHz, CHLOROFORM-*D*) δ 7.40 (dd, *J* = 34.9, 10.8 Hz, 1H), 7.22 – 7.15 (m, 4H), 7.15 – 6.99 (m, 4H), 5.23 (dd, *J* = 45.7, 8.8 Hz, 1H), 4.78 – 4.36 (m, 3H), 3.78 – 3.63 (m, 1H), 3.58 – 3.41 (m, 1H), 2.45 – 2.40 (m, 6H), 2.40 – 2.32 (m, 1H), 2.19 – 1.89 (m, 3H), 1.89 – 1.79 (m, 1H), 1.79 – 1.51 (m, 4H), 1.41 (d, *J* = 1.5 Hz, 9H), 1.40 – 1.09 (m, 7H), 1.08 – 0.96 (m, 6H), 0.95 – 0.57 (m, 2H).
7.25 CHLOROFORM-D
f1 (ppm)

(96) Boc-L-HPhe-L-Pro-Val^{P}(OPh-4-SCH$_3$)$_2$

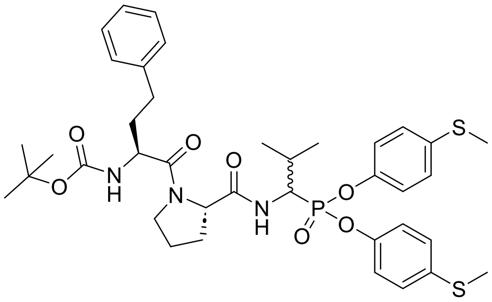

Name: *tert*-butyl ((2S)-1-((2S)-2-((1-(bis(4-(methylthio)phenoxy)phosphoryl)-2-methylpropyl)carbamoyl)pyrrolidin-1-yl)-1-oxo-4-phenylbutan-2-yl)carbamate

^{31}P NMR (162 MHz, Chloroform-d) δ 18.52 (s, 51%), 18.00 (s, 49%).

^{1}H NMR (400 MHz, Chloroform-d) δ 7.33 – 7.25 (m, 1H, NH), 7.24 – 6.90 (m, 13H, 13xArH), 5.32 (dd, J = 49.3, 8.9 Hz, 1H, NH), 4.75 – 4.30 (m, 3H, CHP, 2xCH), 3.57 – 3.41 (m, 1H, 0.5xCH$_2$), 3.33 – 3.11 (m, 1H, 0.5xCH$_2$), 2.79 – 2.49 (m, 2H, CH$_2$), 2.43 (dd, J = 5.9, 2.3 Hz, 6H, 2xSCH$_3$), 2.40 – 2.06 (m, 2H, 0.5xCH$_2$, CH), 2.06 – 1.69 (m, 5H, 2.5xCH$_2$), 1.43 (s, 9H, Boc), 1.11 – 0.94 (m, 6H, 2xCH$_3$).

HRMS: $C_{38}H_{50}N_3O_7PS_2$ [M+Na]$^+$ calc. 778.2725 found 778.2719

HPLC: purity >99%; retention time 19.787 and 19.957 min

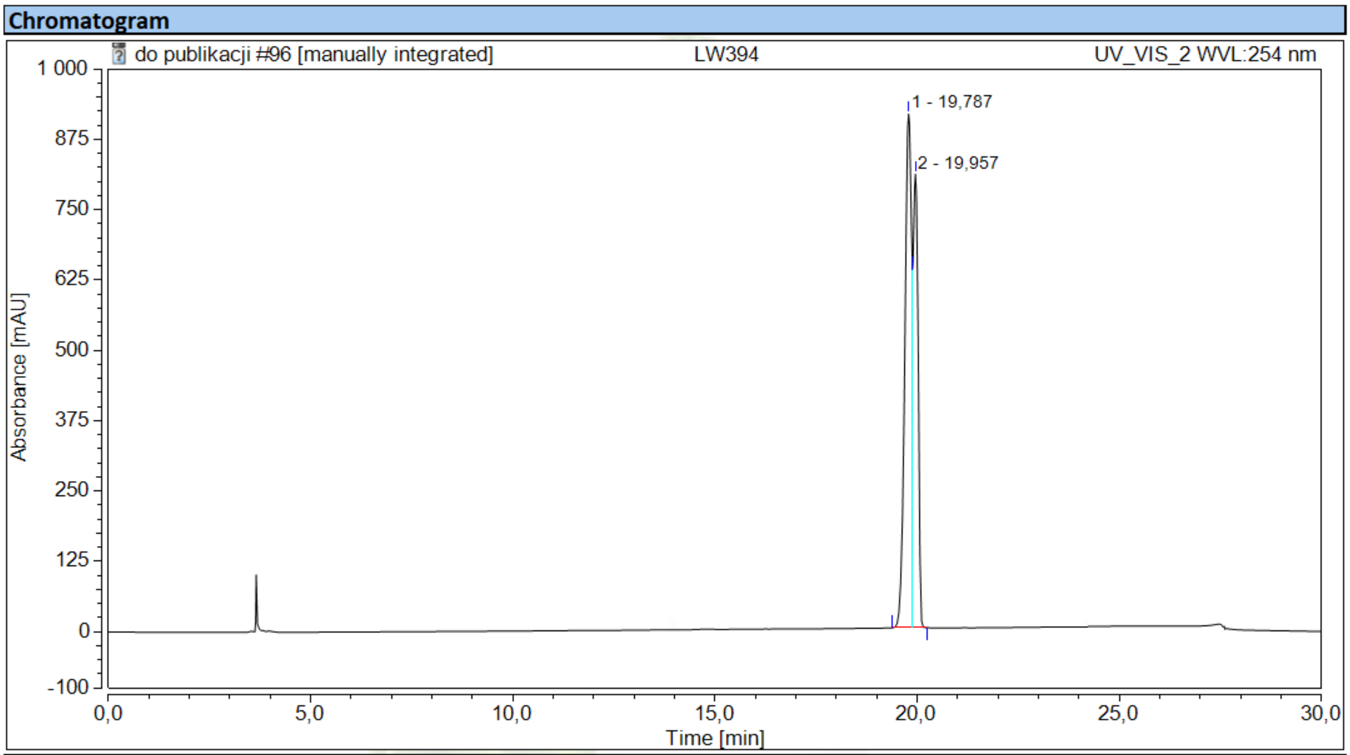

Integration Results

No.	Peak Name	Retention Time min	Area mAU*min	Height mAU	Relative Area %	Relative Height %	Amount n.a.
1		19,787	175,170	912,891	60,14	53,11	n.a.
2		19,957	116,122	805,910	39,86	46,89	n.a.
**Total:**			**291,291**	**1718,802**	**100,00**	**100,00**	

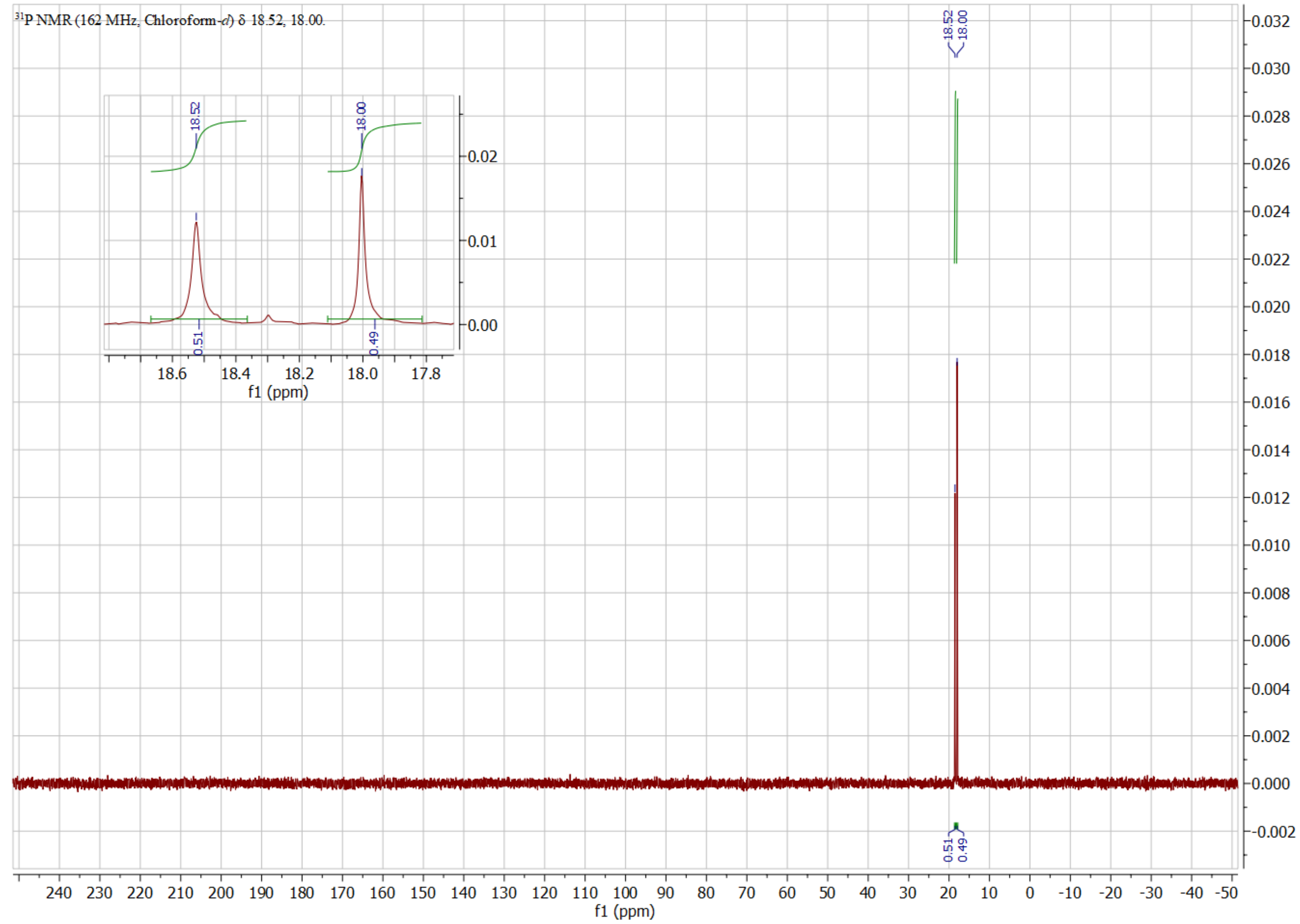
31P NMR (162 MHz, Chloroform-d) δ 18.52, 18.00.
18.52
18.00
0.51
0.49
f1 (ppm)

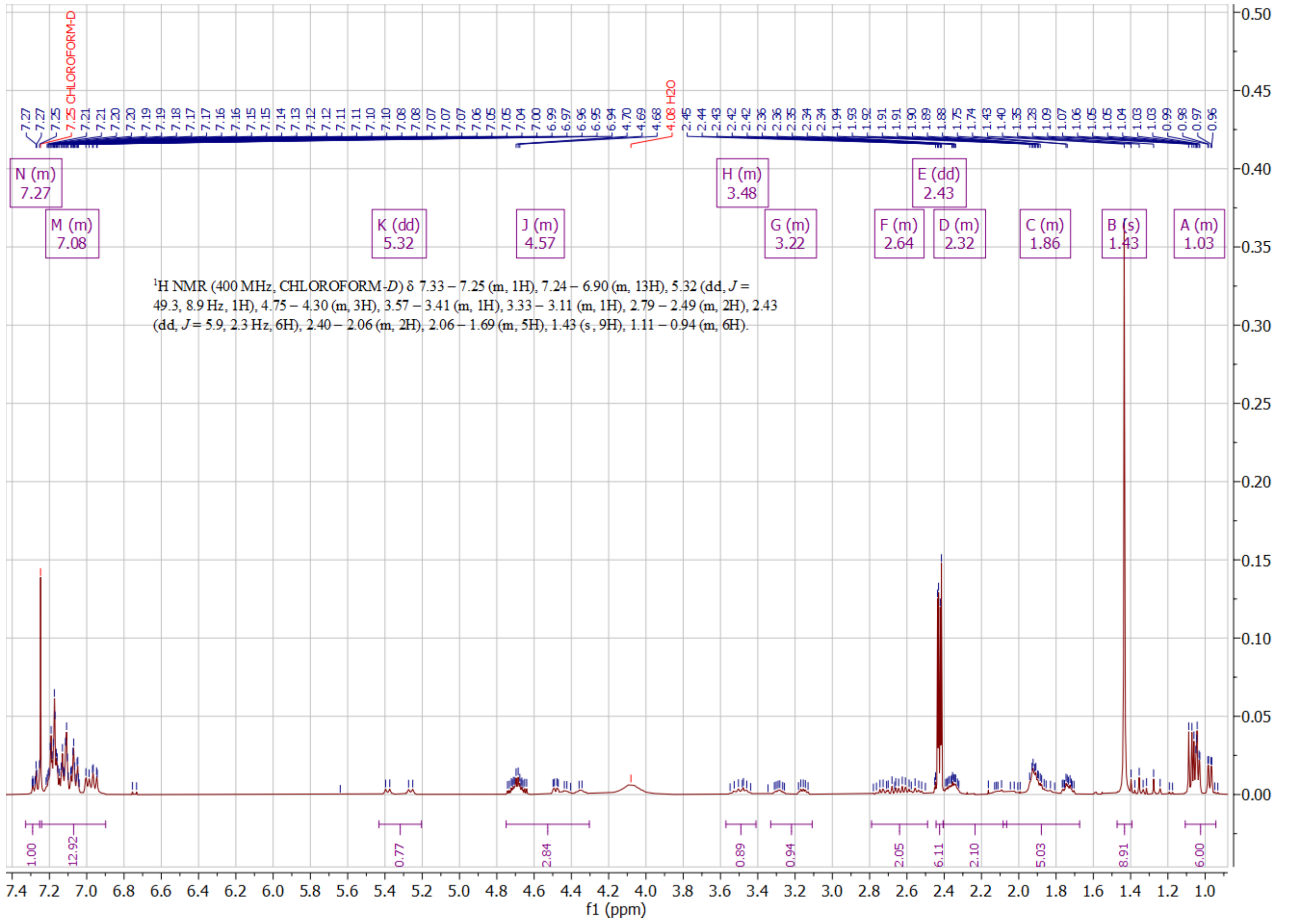
1H NMR (400 MHz, CHLOROFORM-D) δ 7.33 – 7.25 (m, 1H), 7.24 – 6.90 (m, 13H), 5.32 (dd, J = 49.3, 8.9 Hz, 1H), 4.75 – 4.30 (m, 3H), 3.57 – 3.41 (m, 1H), 3.33 – 3.11 (m, 1H), 2.79 – 2.49 (m, 2H), 2.43 (dd, J = 5.9, 2.3 Hz, 6H), 2.40 – 2.06 (m, 2H), 2.06 – 1.69 (m, 5H), 1.43 (s, 9H), 1.11 – 0.94 (m, 6H).
CHLOROFORM-D
H2O
N (m) 7.27
M (m) 7.08
K (dd) 5.32
J (m) 4.57
H (m) 3.48
G (m) 3.22
F (m) 2.64
E (dd) 2.43
D (m) 2.32
C (m) 1.86
B (s) 1.43
A (m) 1.03
f1 (ppm)

(97) Boc-D-HPhe-L-Pro-$Val^P(OPh\text{-}4\text{-}SCH_3)_2$

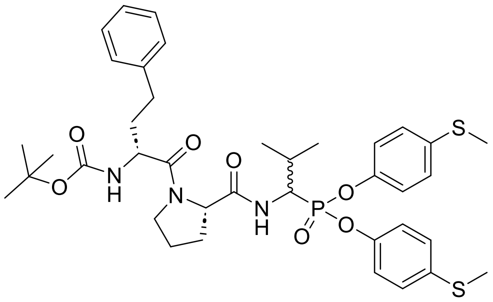

Name: *tert*-butyl ((2R)-1-((2S)-2-((1-(bis(4-(methylthio)phenoxy)phosphoryl)-2-methylpropyl)carbamoyl)pyrrolidin-1-yl)-1-oxo-4-phenylbutan-2-yl)carbamate

^{31}P NMR (162 MHz, Chloroform-d) δ 18.48 (s, 57%), 18.18 (s, 43%).

^{1}H NMR (400 MHz, Chloroform-d) δ 7.54 (dd, J = 73.6, 10.1 Hz, 1H, NH), 7.31 – 7.25 (m, 2H, 2xArH), 7.23 – 7.09 (m, 9H, 9xArH), 7.09 – 7.00 (m, 2H, 2xArH), 5.35 (dd, J = 15.8, 8.5 Hz, 1H, NH), 4.72 – 4.61 (m, 1H, CHP), 4.57 – 4.35 (m, 2H, 2xCH), 3.68 – 3.52 (m, 1H, 0.5xCH_2), 3.21 – 3.10 (m, 1H, 0.5xCH_2), 2.80 – 2.56 (m, 2H, CH_2), 2.43 (dd, J = 4.5, 1.0 Hz, 6H, 2xSCH_3), 2.41 – 2.11 (m, 2H, 0.5xCH_2, CH), 2.11 – 1.67 (m, 5H, 2.5xCH_2), 1.38 (d, J = 20.3 Hz, 9H, Boc), 1.10 – 0.92 (m, 6H, 3xCH_3).

HRMS: $C_{38}H_{50}N_3O_7PS_2$ $[M+Na]^+$ calc. 778.2725 found 778.2717

HPLC: purity >98%; retention time 19.870 min

Chromatogram

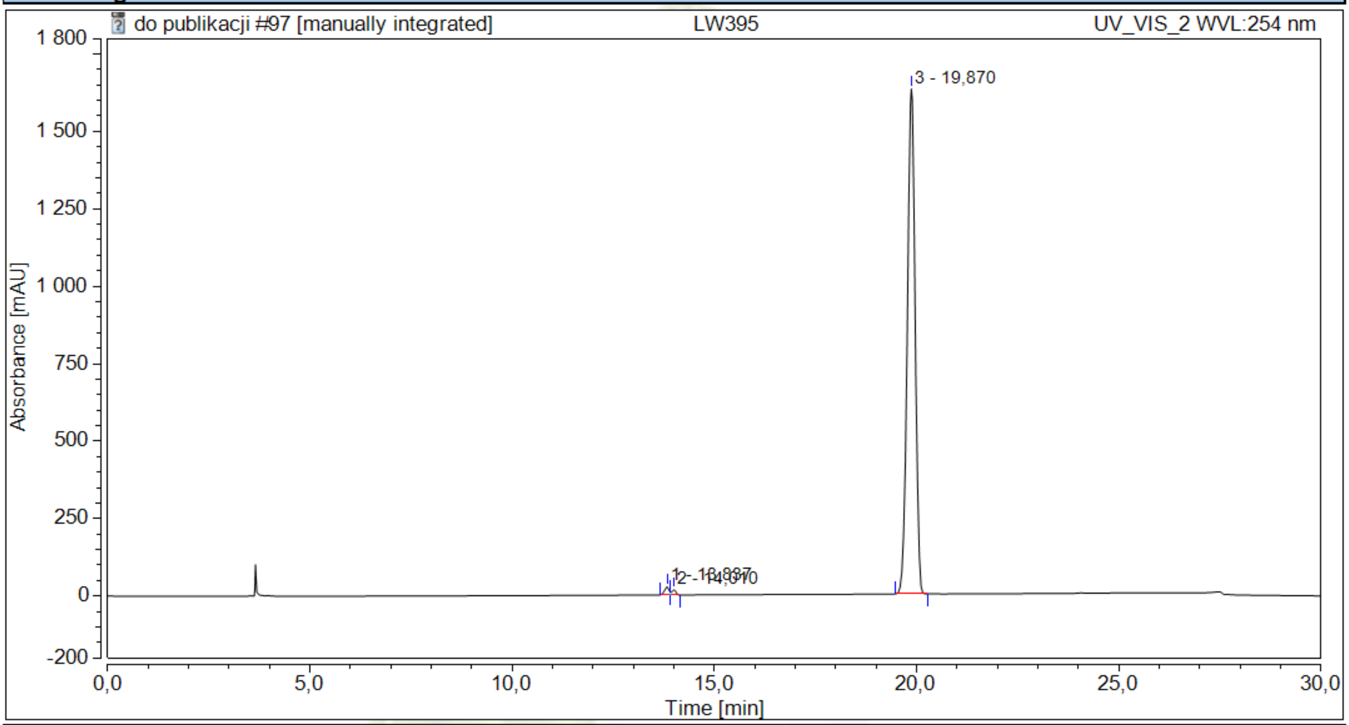

Integration Results

No.	Peak Name	Retention Time min	Area mAU*min	Height mAU	Relative Area %	Relative Height %	Amount n.a.
1		13,837	3,329	27,341	0,91	1,63	n.a.
2		14,010	1,939	17,184	0,53	1,03	n.a.
3		19,870	361,799	1630,562	98,56	97,34	n.a.
**Total:**			**367,067**	**1675,087**	**100,00**	**100,00**	

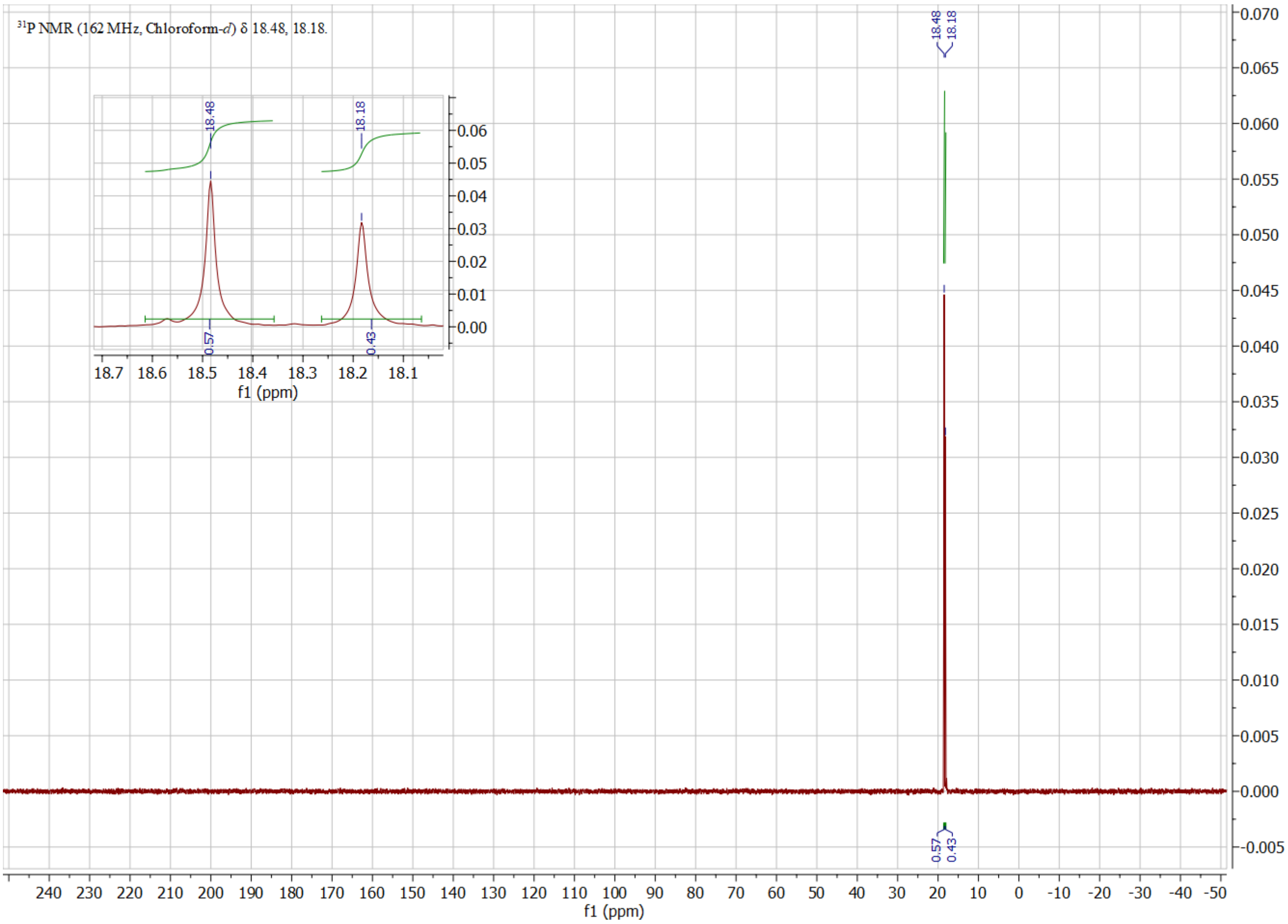

31P NMR (162 MHz, Chloroform-d) δ 18.48, 18.18.
f1 (ppm)

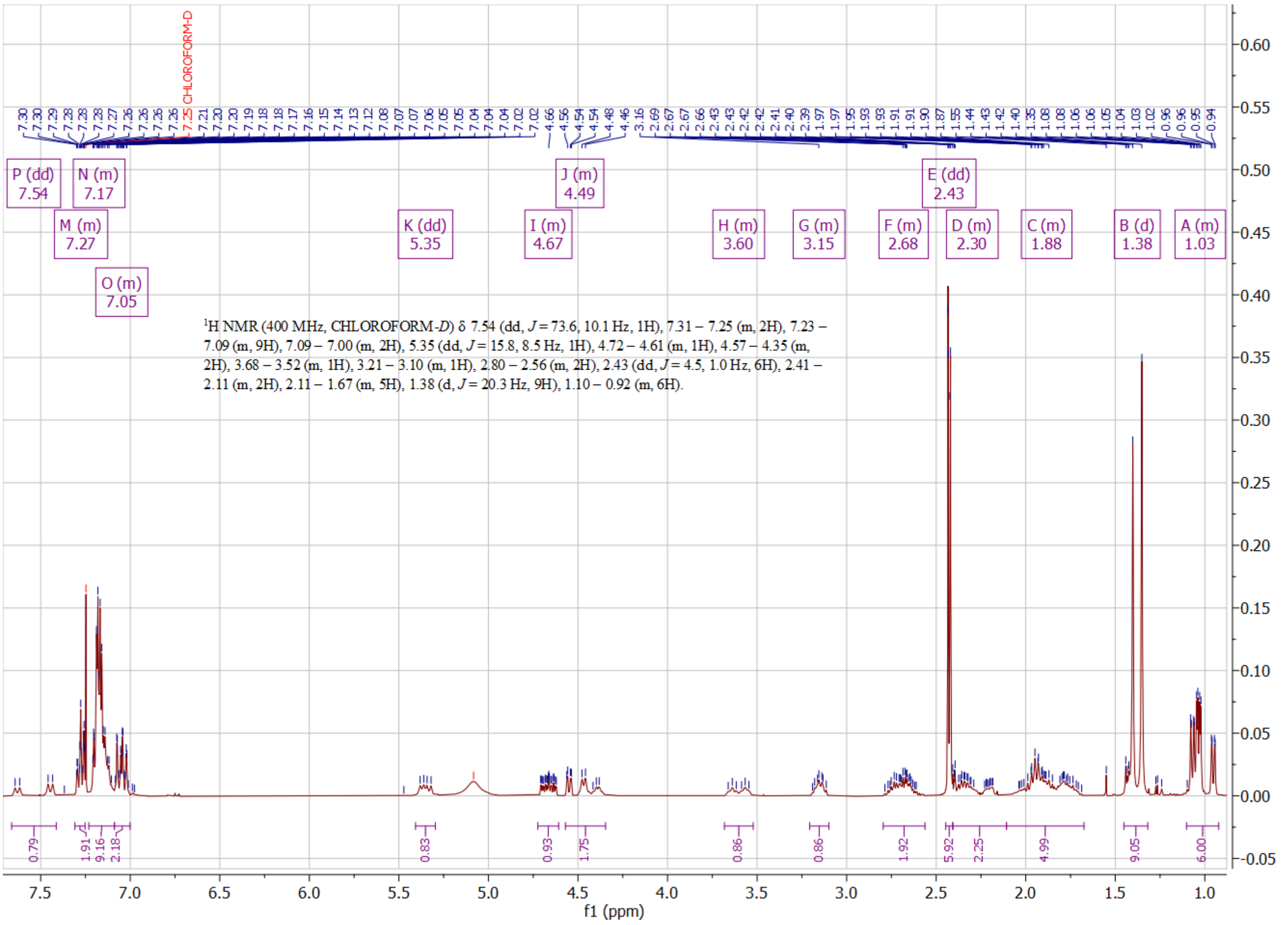

1H NMR (400 MHz, CHLOROFORM-D) δ 7.54 (dd, J = 73.6, 10.1 Hz, 1H), 7.31 – 7.25 (m, 2H), 7.23 – 7.09 (m, 9H), 7.09 – 7.00 (m, 2H), 5.35 (dd, J = 15.8, 8.5 Hz, 1H), 4.72 – 4.61 (m, 1H), 4.57 – 4.35 (m, 2H), 3.68 – 3.52 (m, 1H), 3.21 – 3.10 (m, 1H), 2.80 – 2.56 (m, 2H), 2.43 (dd, J = 4.5, 1.0 Hz, 6H), 2.41 – 2.11 (m, 2H), 2.11 – 1.67 (m, 5H), 1.38 (d, J = 20.3 Hz, 9H), 1.10 – 0.92 (m, 6H).
CHLOROFORM-D
f1 (ppm)

(98) Boc-L-Igl-L-Pro-Val^{P}(OPh-4-SCH$_3$)$_2$

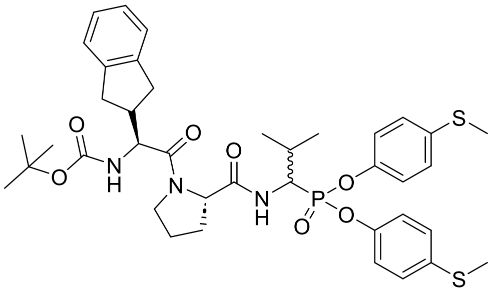

Name: tert-butyl ((1S)-2-((2S)-2-((1-(bis(4-(methylthio)phenoxy)phosphoryl)-2-methylpropyl)carbamoyl)pyrrolidin-1-yl)-1-(2,3-dihydro-1H-inden-2-yl)-2-oxoethyl)carbamate
^{31}P NMR (162 MHz, Chloroform-d) δ 18.20 (s, 46%), 17.92 (s, 54%).
^{1}H NMR (400 MHz, Chloroform-d) δ 1H NMR (400 MHz, CHLOROFORM-D) δ 7.34 (dd, J = 80.7, 10.3 Hz, 1H, NH), 7.22 – 6.97 (m, 12H, 12xArH), 5.48 (dd, J = 37.9, 8.9 Hz, 1H, NH), 4.84 – 4.74 (m, 1H, CHP), 4.71 – 4.52 (m, 1H, CH), 4.52 – 4.46 (m, 1H, CH), 3.84 – 3.73 (m, 1H, 0.5xCH$_2$), 3.63 – 3.47 (m, 1H, 0.5xCH$_2$), 3.05 – 2.53 (m, 5H, 2xCH$_2$, CH), 2.47 – 2.34 (m, 7H, 2xSCH$_3$, CH), 2.34 – 1.86 (m, 4H, 2xCH$_2$), 1.40 (d, J = 2.6 Hz, 9H, Boc), 1.14 – 1.02 (m, 6H, 2xCH$_3$).
HRMS: $C_{39}H_{50}N_3O_7PS_2$ [M+Na]$^+$ calc. 790.2725 found 790.2709
HPLC: purity >99%; retention time 20.087 min

Chromatogram

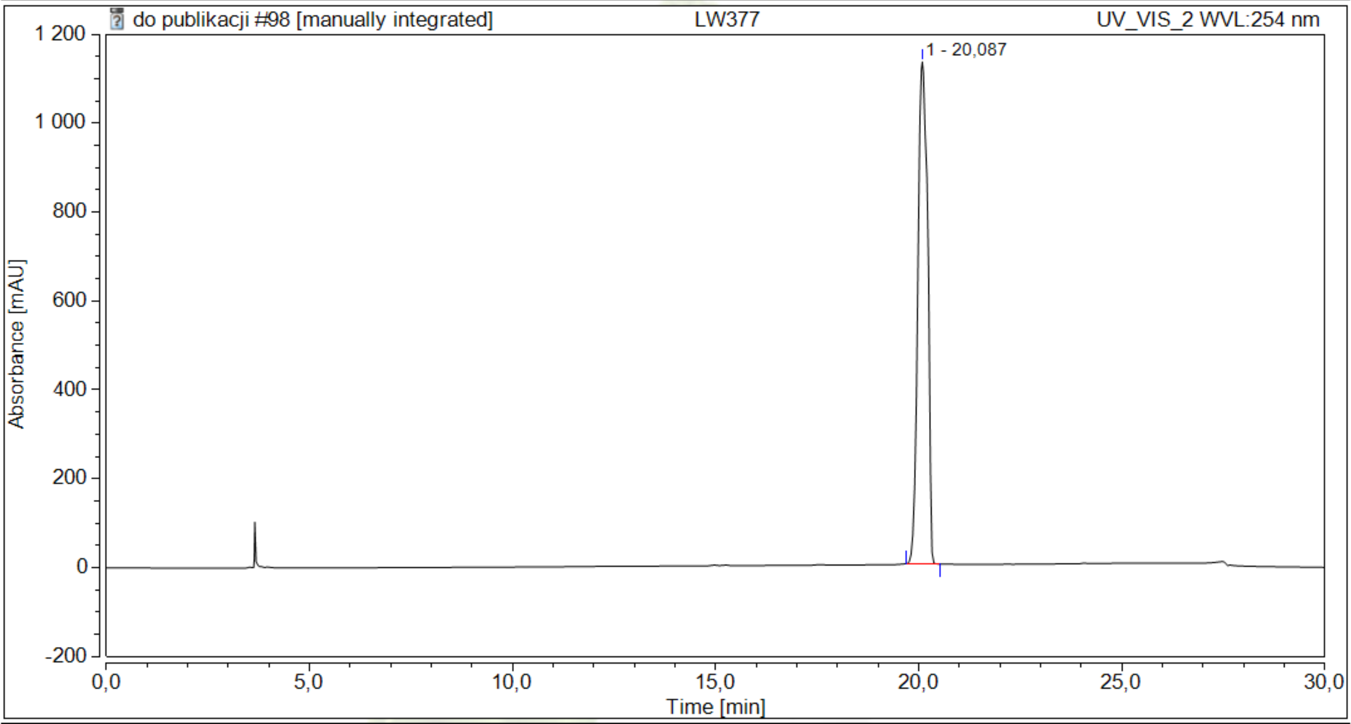

Integration Results

No.	Peak Name	Retention Time min	Area mAU*min	Height mAU	Relative Area %	Relative Height %	Amount n.a.
1		20,087	314,259	1129,711	100,00	100,00	n.a.
**Total:**			**314,259**	**1129,711**	**100,00**	**100,00**	

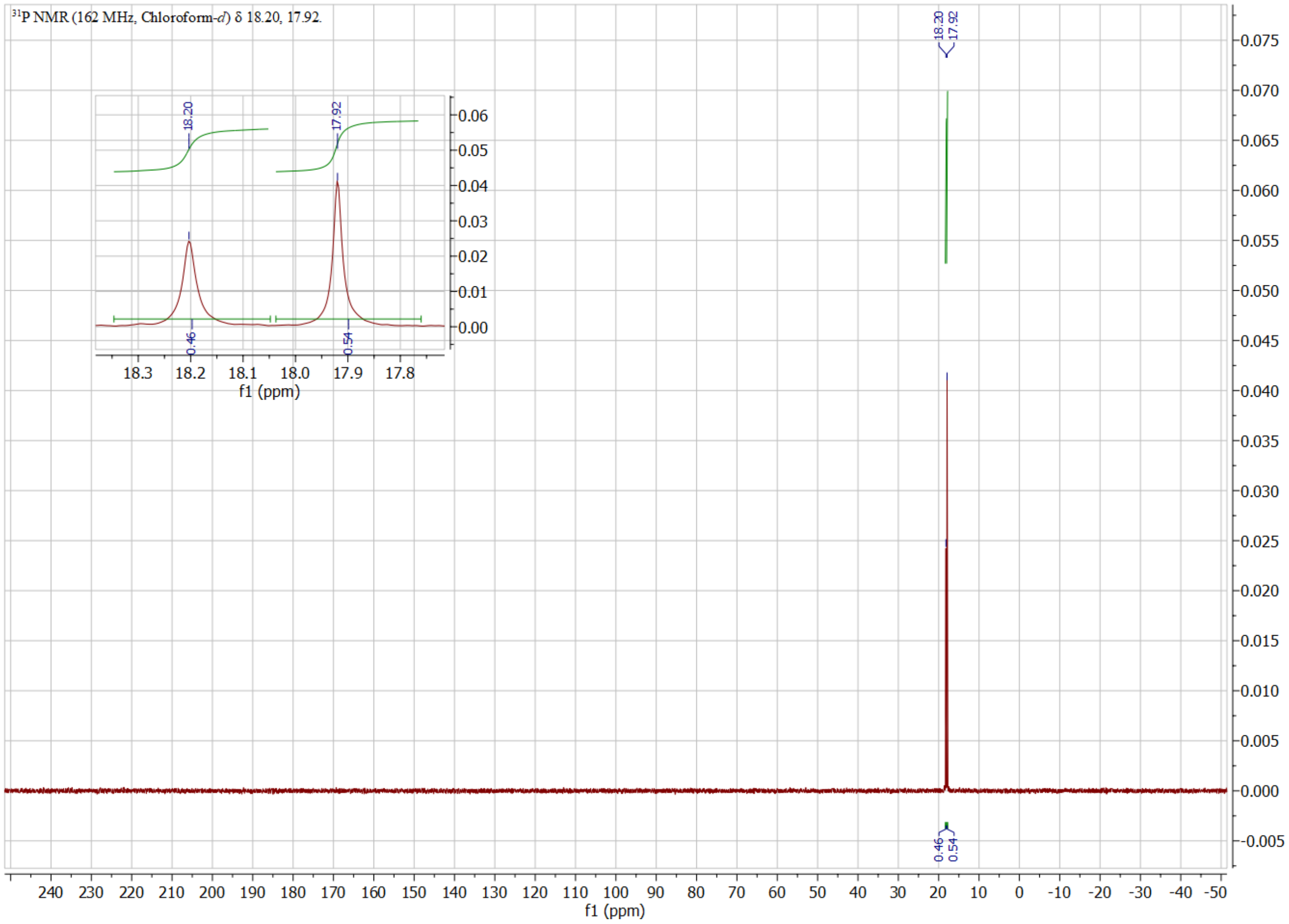
^{31}P NMR (162 MHz, Chloroform-*d*) δ 18.20, 17.92.
f1 (ppm)

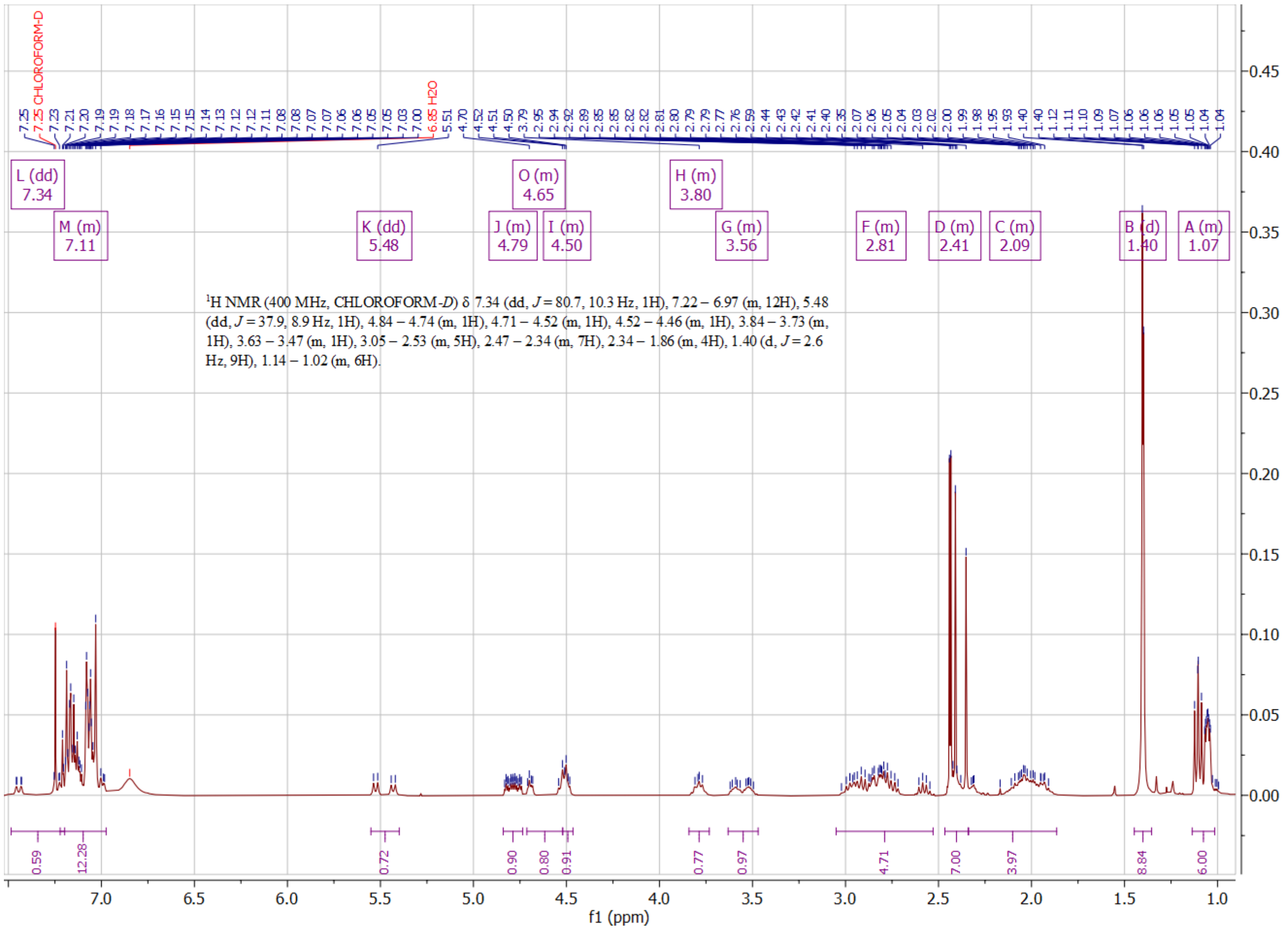
^{1}H NMR (400 MHz, CHLOROFORM-*D*) δ 7.34 (dd, *J* = 80.7, 10.3 Hz, 1H), 7.22 – 6.97 (m, 12H), 5.48 (dd, *J* = 37.9, 8.9 Hz, 1H), 4.84 – 4.74 (m, 1H), 4.71 – 4.52 (m, 1H), 4.52 – 4.46 (m, 1H), 3.84 – 3.73 (m, 1H), 3.63 – 3.47 (m, 1H), 3.05 – 2.53 (m, 5H), 2.47 – 2.34 (m, 7H), 2.34 – 1.86 (m, 4H), 1.40 (d, *J* = 2.6 Hz, 9H), 1.14 – 1.02 (m, 6H).
f1 (ppm)

(99) Boc-D-Igl-L-Pro-Val^{P}(OPh-4-SCH$_3$)$_2$

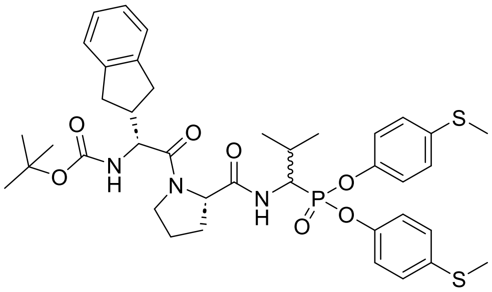

Name: tert-butyl ((1R)-2-((2S)-2-((1-(bis(4-(methylthio)phenoxy)phosphoryl)-2-methylpropyl)carbamoyl)pyrrolidin-1-yl)-1-(2,3-dihydro-1H-inden-2-yl)-2-oxoethyl)carbamate

^{31}P NMR (162 MHz, Chloroform-d) δ 18.33 (s, 50%), 18.07 (s, 50%).

^{1}H NMR (400 MHz, Chloroform-d) δ 7.52 (dd, J = 80.8, 10.4 Hz, 1H, NH), 7.22 – 7.11 (m, 9H, 9xArH), 7.11 – 7.01 (m, 3H, 3xArH), 5.40 (t, J = 8.8 Hz, 1H, NH), 4.77 – 4.67 (m, 1H, CHP), 4.67 – 4.58 (m, 1H, CH), 4.58 – 4.47 (m, 1H, CH), 3.90 – 3.77 (m, 1H, 0.5xCH$_2$), 3.54 – 3.41 (m, 1H, 0.5xCH$_2$), 3.05 – 2.91 (m, 2H, CH$_2$), 2.91 – 2.76 (m, 2H, CH$_2$), 2.74 – 2.63 (m, 1H, CH), 2.43 (dd, J = 3.1, 2.1 Hz, 6H, 2xSCH$_3$), 2.42 – 2.20 (m, 2H, 0.5xCH$_2$, CH), 2.17 – 1.81 (m, 3H, 1.5xCH$_2$), 1.36 (d, J = 23.4 Hz, 9H, Boc), 1.12 – 0.94 (m, 6H, 2xCH$_3$).

HRMS: $C_{39}H_{50}N_3O_7PS_2$ [M+Na]$^+$ calc. 790.2725 found 790.2712

HPLC: purity >99%; retention time 20.110 min

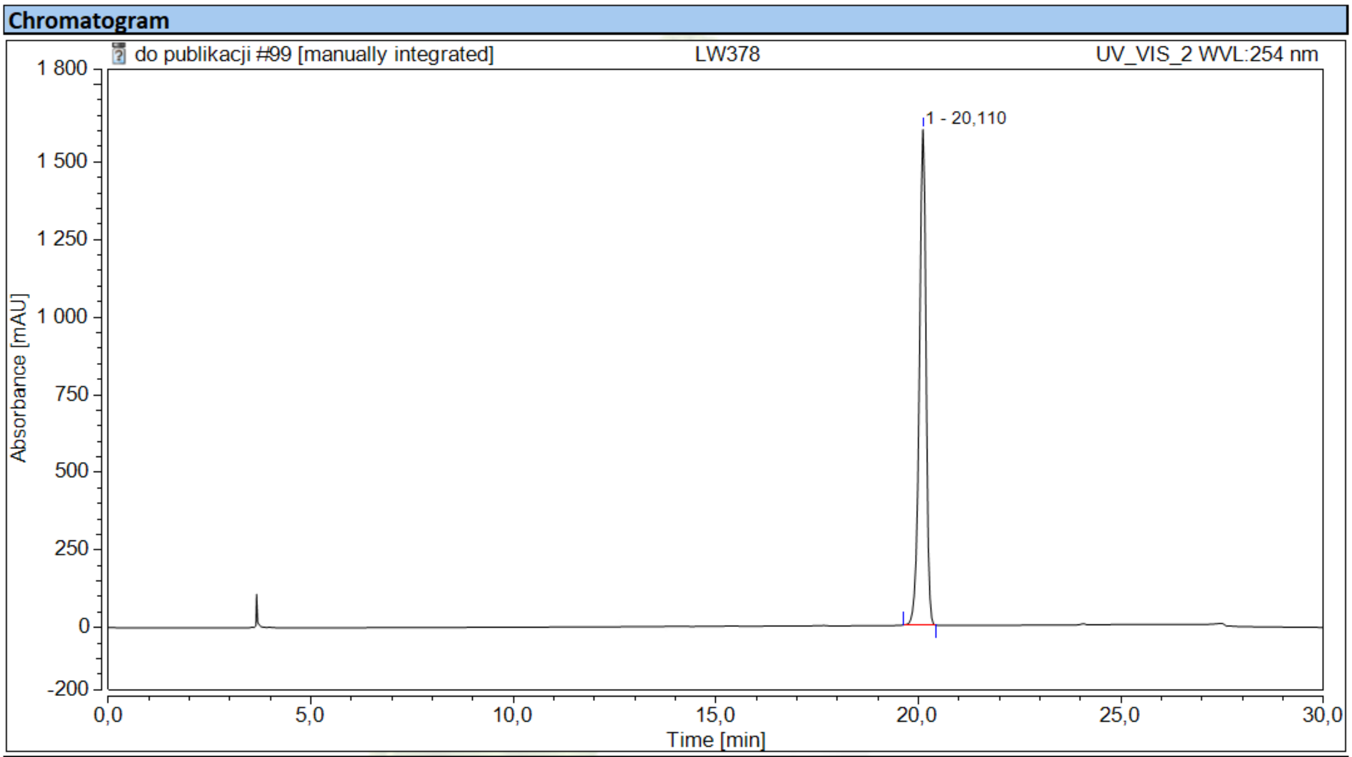

Integration Results

No.	Peak Name	Retention Time min	Area mAU*min	Height mAU	Relative Area %	Relative Height %	Amount n.a.
1		20,110	307,149	1596,423	100,00	100,00	n.a.
**Total:**			**307,149**	**1596,423**	**100,00**	**100,00**	

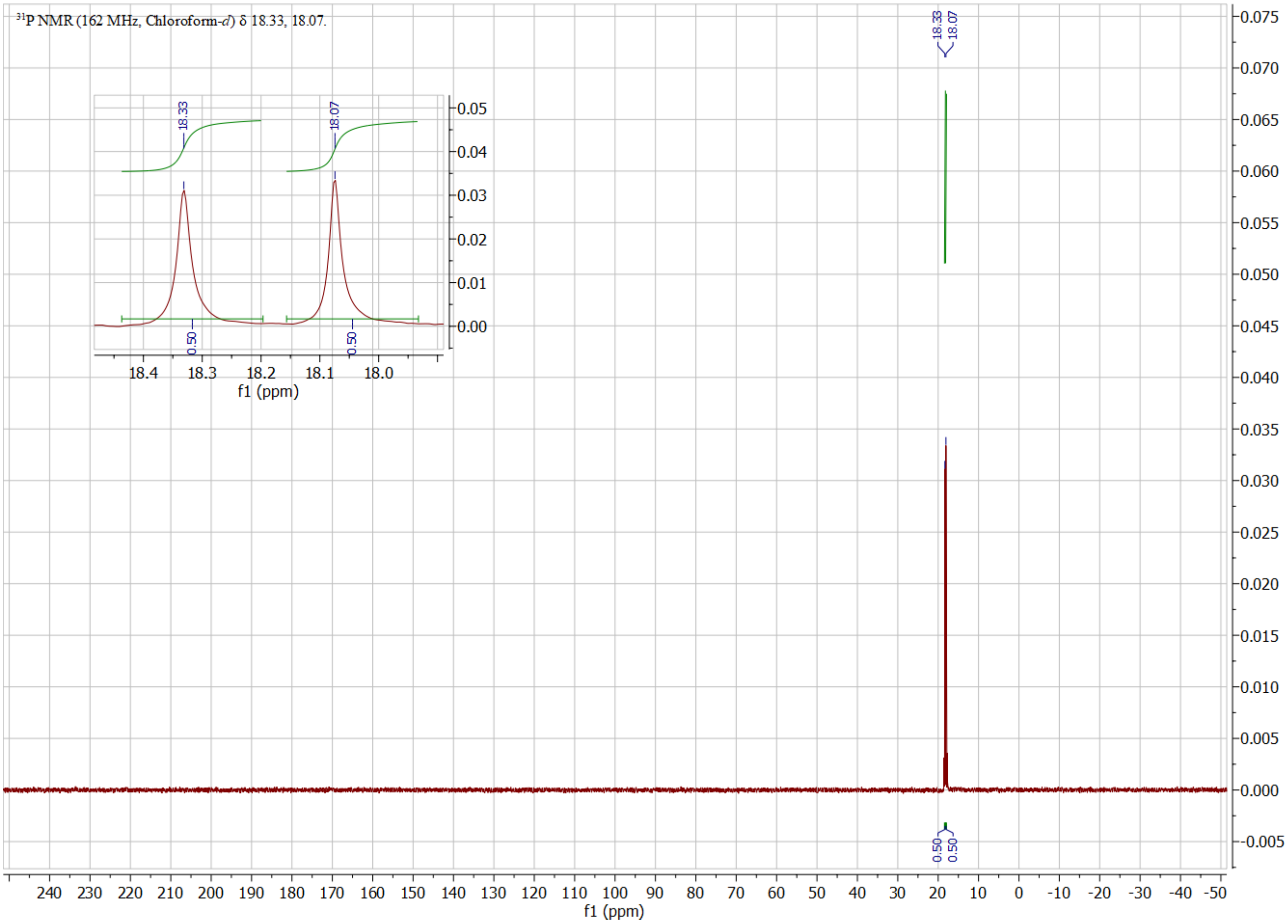

^{31}P NMR (162 MHz, Chloroform-d) δ 18.33, 18.07.

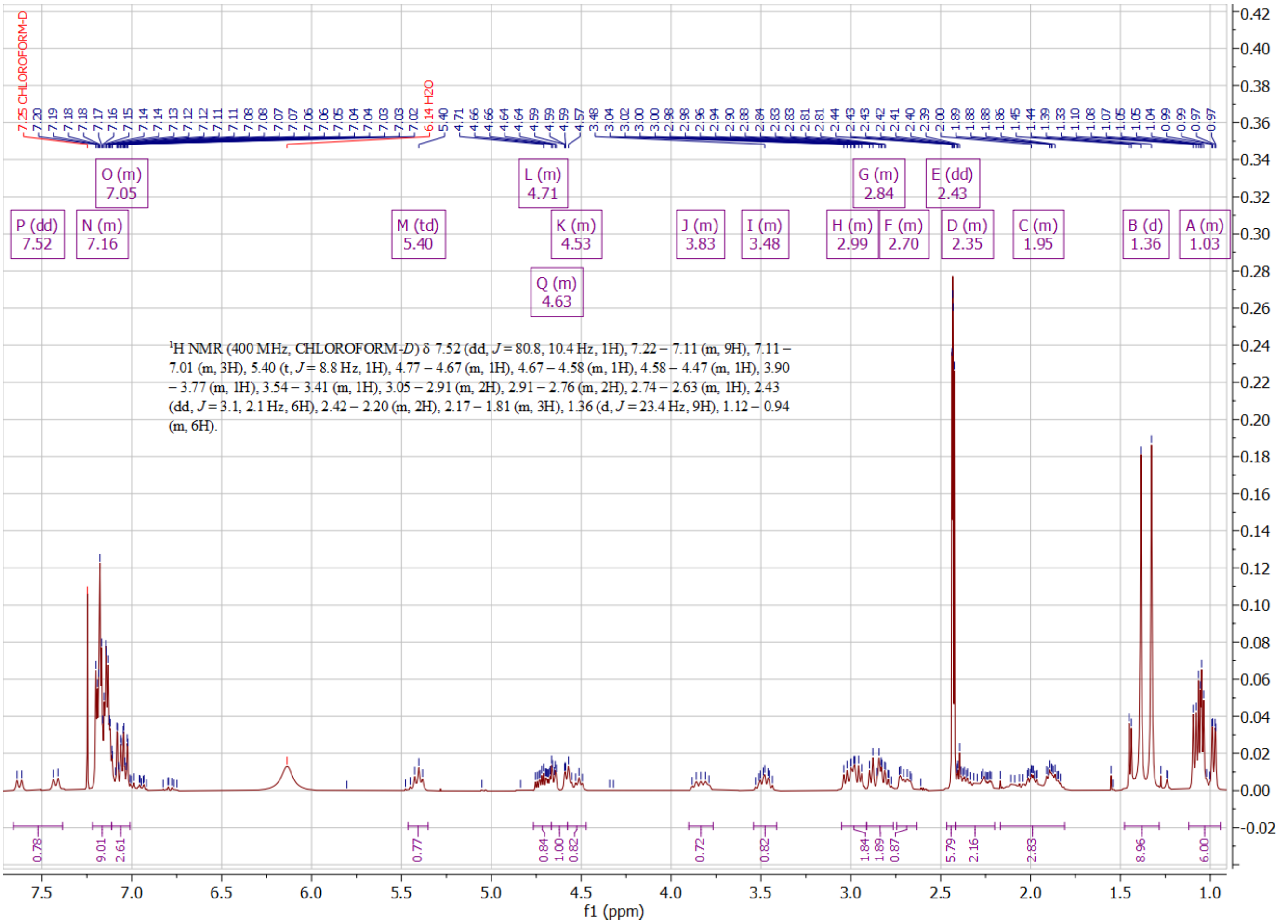

^{1}H NMR (400 MHz, CHLOROFORM-D) δ 7.52 (dd, J = 80.8, 10.4 Hz, 1H), 7.22 – 7.11 (m, 9H), 7.11 – 7.01 (m, 3H), 5.40 (t, J = 8.8 Hz, 1H), 4.77 – 4.67 (m, 1H), 4.67 – 4.58 (m, 1H), 4.58 – 4.47 (m, 1H), 3.90 – 3.77 (m, 1H), 3.54 – 3.41 (m, 1H), 3.05 – 2.91 (m, 2H), 2.91 – 2.76 (m, 2H), 2.74 – 2.63 (m, 1H), 2.43 (dd, J = 3.1, 2.1 Hz, 6H), 2.42 – 2.20 (m, 2H), 2.17 – 1.81 (m, 3H), 1.36 (d, J = 23.4 Hz, 9H), 1.12 – 0.94 (m, 6H).

(100) Boc-L-Thr(Bzl)-L-Pro- Val^{P}(OPh-4-SCH$_3$)$_2$

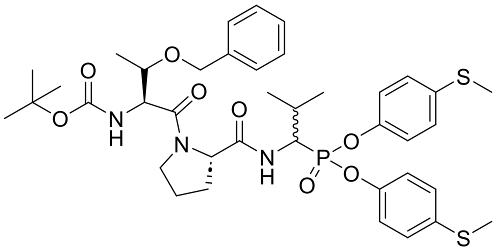

Name: *tert*-butyl ((2S,3S)-3-(benzyloxy)-1-((2S)-2-((1-(bis(4-(methylthio)phenoxy)phosphoryl)-2-methylpropyl)carbamoyl)pyrrolidin-1-yl)-1-oxobutan-2-yl)carbamate

^{31}P NMR (162 MHz, Chloroform-d) δ 18.59 (s, 46%), 18.11 (s, 54%).

^{1}H NMR (400 MHz, Chloroform-d) δ 7.36 – 7.22 (m, 5H, 4xArH, NH), 7.22 – 7.01 (m, 9H, 9xArH), 5.44 (dd, J = 47.0, 8.2 Hz, 1H, NH), 4.83 – 4.26 (m, 5H, CH$_2$, CHP, 2xCH), 3.92 – 3.43 (m, 3H, CH$_2$, CH), 2.49 – 2.39 (m, 6H, 2xSCH$_3$), 2.39 – 2.23 (m, 1H, CH), 2.23 – 1.97 (m, 2H, CH$_2$), 1.97 – 1.78 (m, 2H, CH$_2$), 1.40 (d, J = 6.6 Hz, 9H, Boc), 1.18 (dd, J = 22.2, 6.3 Hz, 3H, CH$_3$), 1.09 (td, J = 7.3, 7.3 Hz, 3H, CH$_3$), 1.00 (dd, J = 14.5, 6.8 Hz, 3H, CH$_3$).

HRMS: $C_{39}H_{52}N_3O_8PS_2$ [M+Na]$^+$ calc. 808.2831 found 808.2800

HPLC: purity >99%; retention time 20.043 min

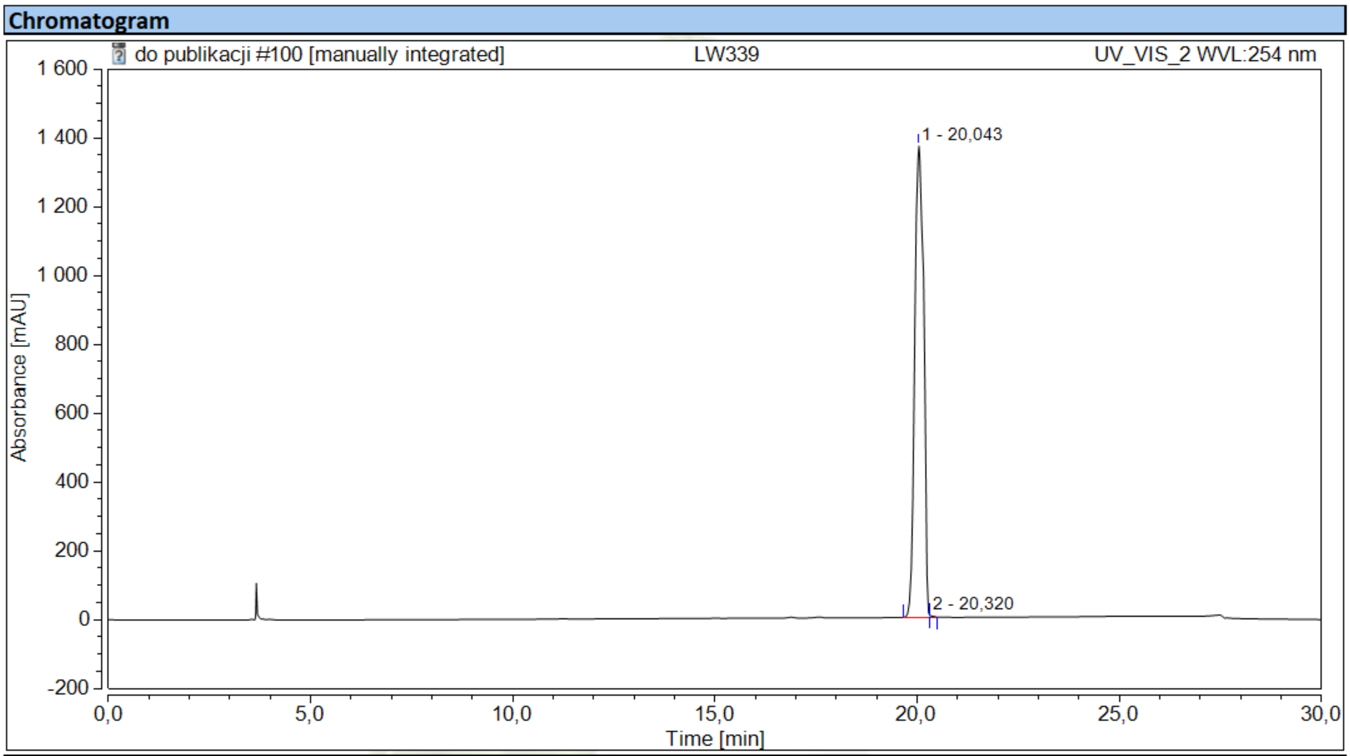

Integration Results

No.	Peak Name	Retention Time min	Area mAU*min	Height mAU	Relative Area %	Relative Height %	Amount n.a.
1		20,043	351,154	1369,252	99,88	99,70	n.a.
2		20,320	0,421	4,074	0,12	0,30	n.a.
**Total:**			**351,575**	**1373,326**	**100,00**	**100,00**	

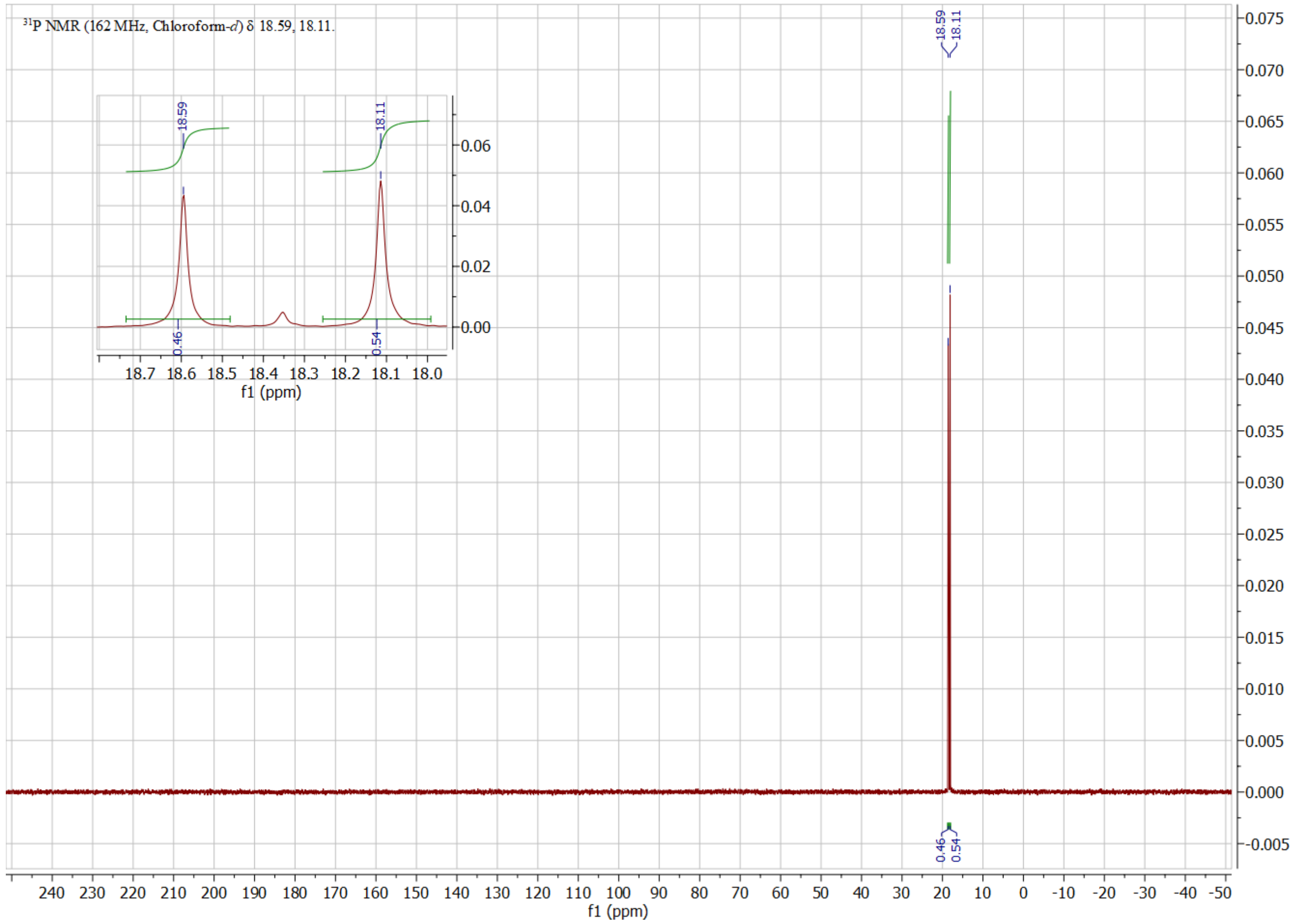
^{31}P NMR (162 MHz, Chloroform-*d*) δ 18.59, 18.11.
f1 (ppm)

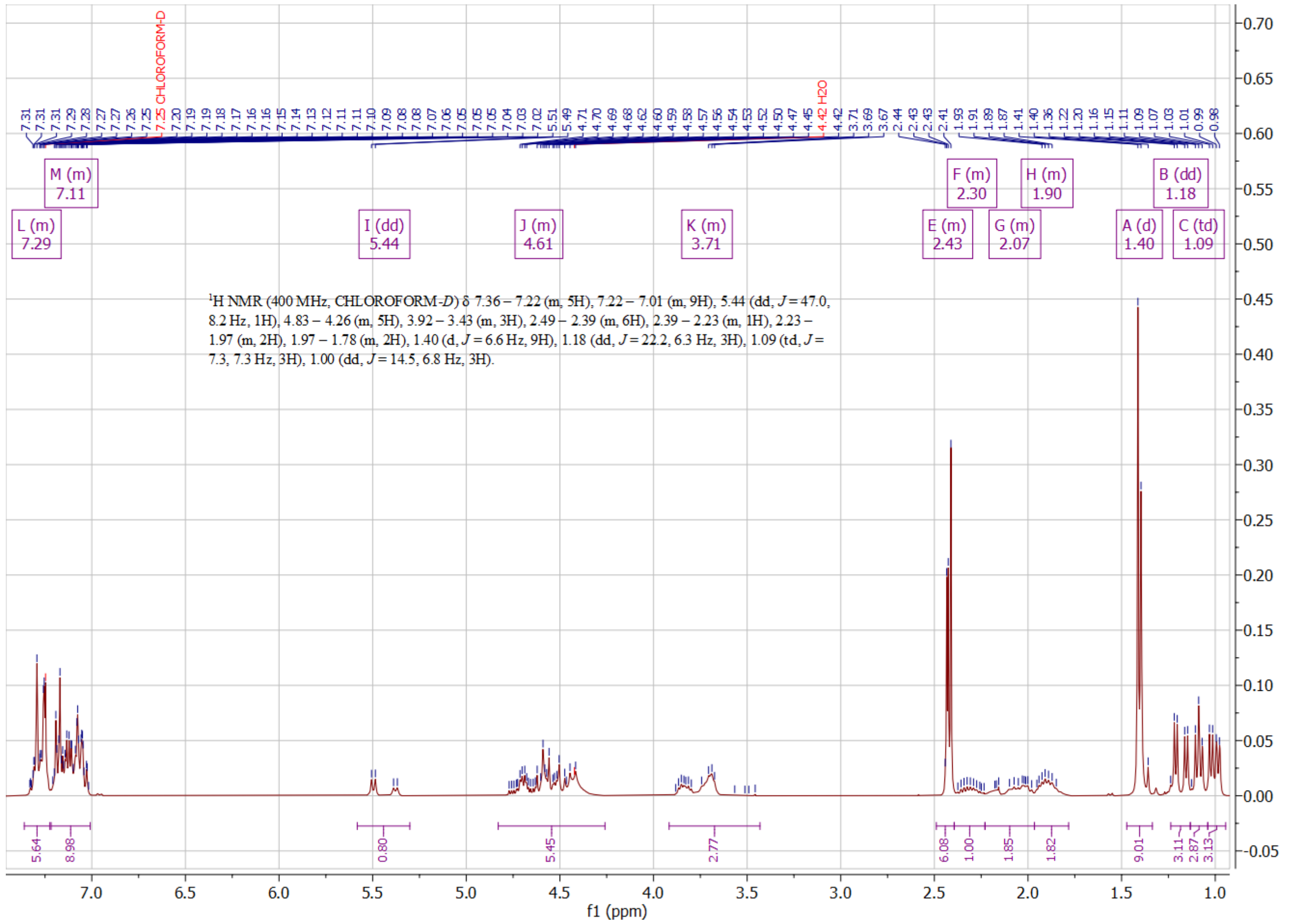
^{1}H NMR (400 MHz, CHLOROFORM-*D*) δ 7.36 – 7.22 (m, 5H), 7.22 – 7.01 (m, 9H), 5.44 (dd, *J* = 47.0, 8.2 Hz, 1H), 4.83 – 4.26 (m, 5H), 3.92 – 3.43 (m, 3H), 2.49 – 2.39 (m, 6H), 2.39 – 2.23 (m, 1H), 2.23 – 1.97 (m, 2H), 1.97 – 1.78 (m, 2H), 1.40 (d, *J* = 6.6 Hz, 9H), 1.18 (dd, *J* = 22.2, 6.3 Hz, 3H), 1.09 (td, *J* = 7.3, 7.3 Hz, 3H), 1.00 (dd, *J* = 14.5, 6.8 Hz, 3H).
f1 (ppm)

(101) Boc-L-Cys(Bzl)-L-Pro- Val^{P}(OPh-4-SCH$_3$)$_2$

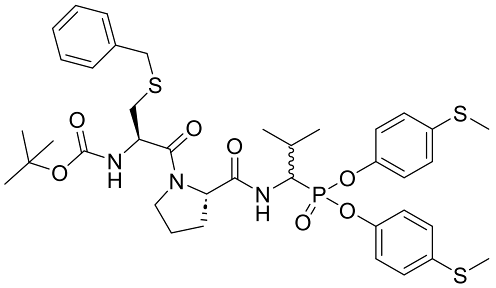

Name: *tert*-butyl ((2R)-3-(benzylthio)-1-((2S)-2-((1-(bis(4-(methylthio)phenoxy)phosphoryl)-2-methylpropyl)carbamoyl)pyrrolidin-1-yl)-1-oxopropan-2-yl)carbamate

^{31}P NMR (162 MHz, Chloroform-d) δ 18.44 (s, 54%), 17.92 (s, 46%).

^{1}H NMR (400 MHz, Chloroform-d) δ 7.35 – 7.25 (m, 3H, 2xArH, NH), 7.25 – 7.14 (m, 7H, 7xArH), 7.14 – 6.98 (m, 4H, 4xArH), 5.34 (dd, J = 22.6, 8.7 Hz, 1H, NH), 4.76 – 4.49 (m, 3H, CHP, 2xCH), 3.75 – 3.55 (m, 3H, 1.5xCH$_2$), 3.50 – 3.34 (m, 1H, 0.5xCH$_2$), 2.75 – 2.50 (m, 2H, CH$_2$), 2.46 – 2.41 (m, 6H, 2xSCH$_3$), 2.41 – 2.28 (m, 1H, CH), 2.28 – 2.15 (m, 1H, 0.5xCH$_2$), 2.11 – 1.78 (m, 3H, 1.5xCH$_2$), 1.42 (d, J = 4.5 Hz, 9H, Boc), 1.11 – 0.95 (m, 6H, 2xCH$_3$).

HRMS: $C_{38}H_{50}N_3O_7PS_3$ [M+Na]$^+$ calc. 810.2446 found 810.2424

HPLC: purity >99%; retention time 19.920 min

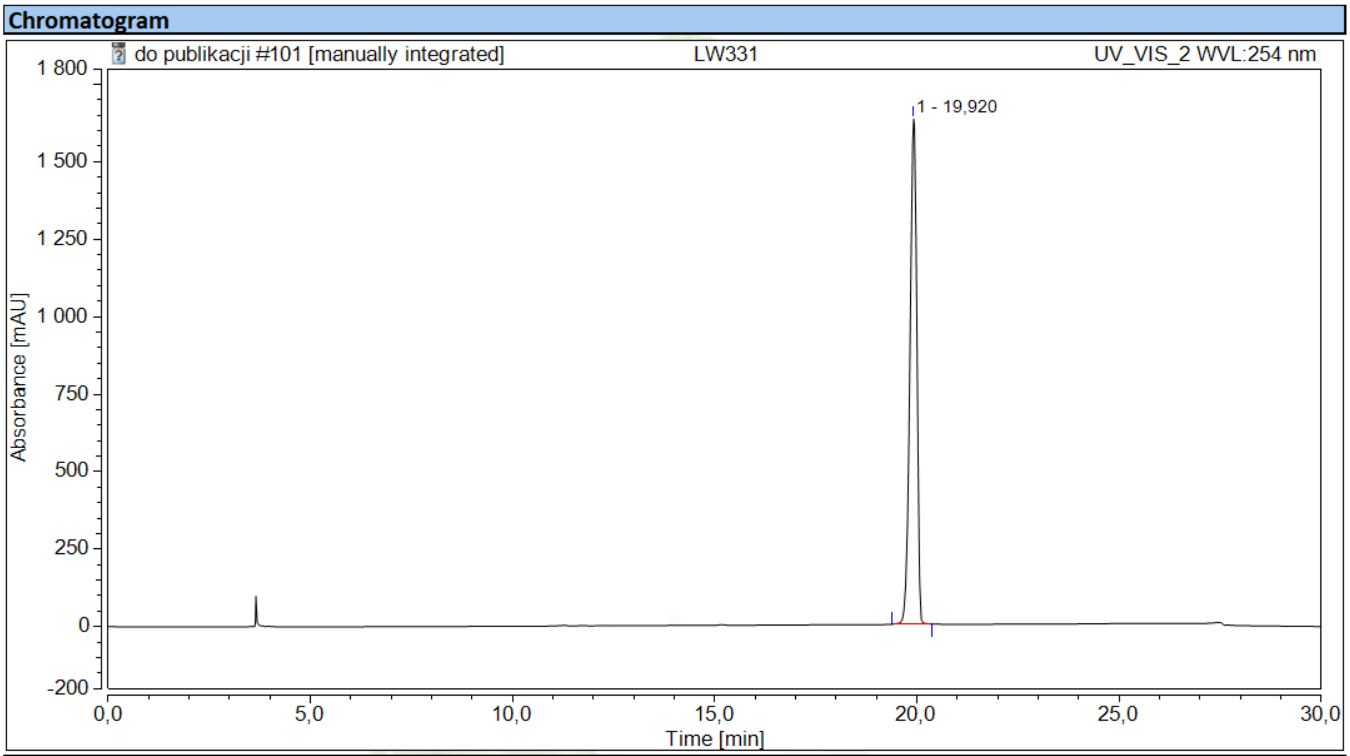

Integration Results

No.	Peak Name	Retention Time min	Area mAU*min	Height mAU	Relative Area %	Relative Height %	Amount n.a.
1		19,920	320,986	1630,093	100,00	100,00	n.a.
**Total:**			**320,986**	**1630,093**	**100,00**	**100,00**	

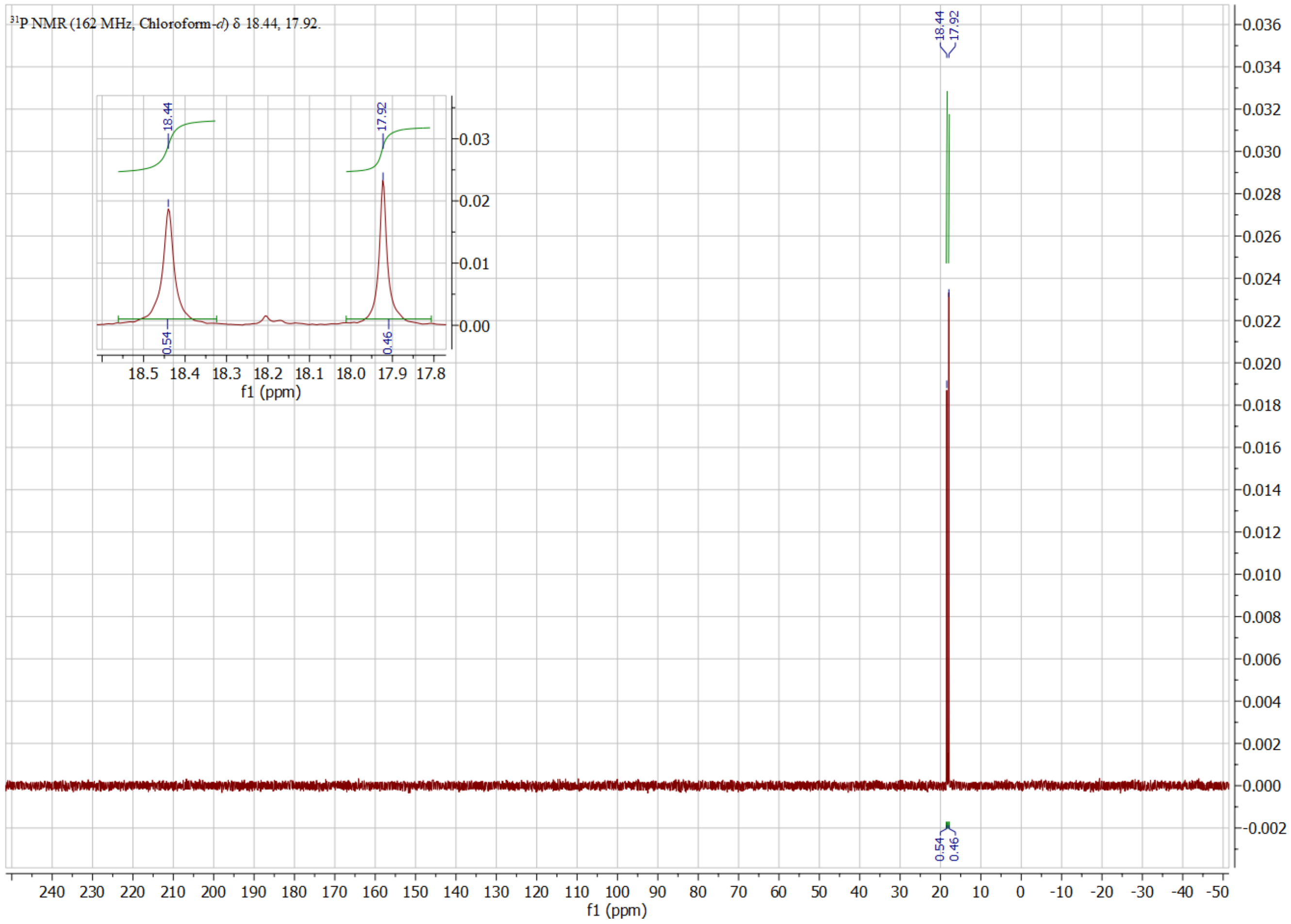
31P NMR (162 MHz, Chloroform-d) δ 18.44, 17.92.
f1 (ppm)

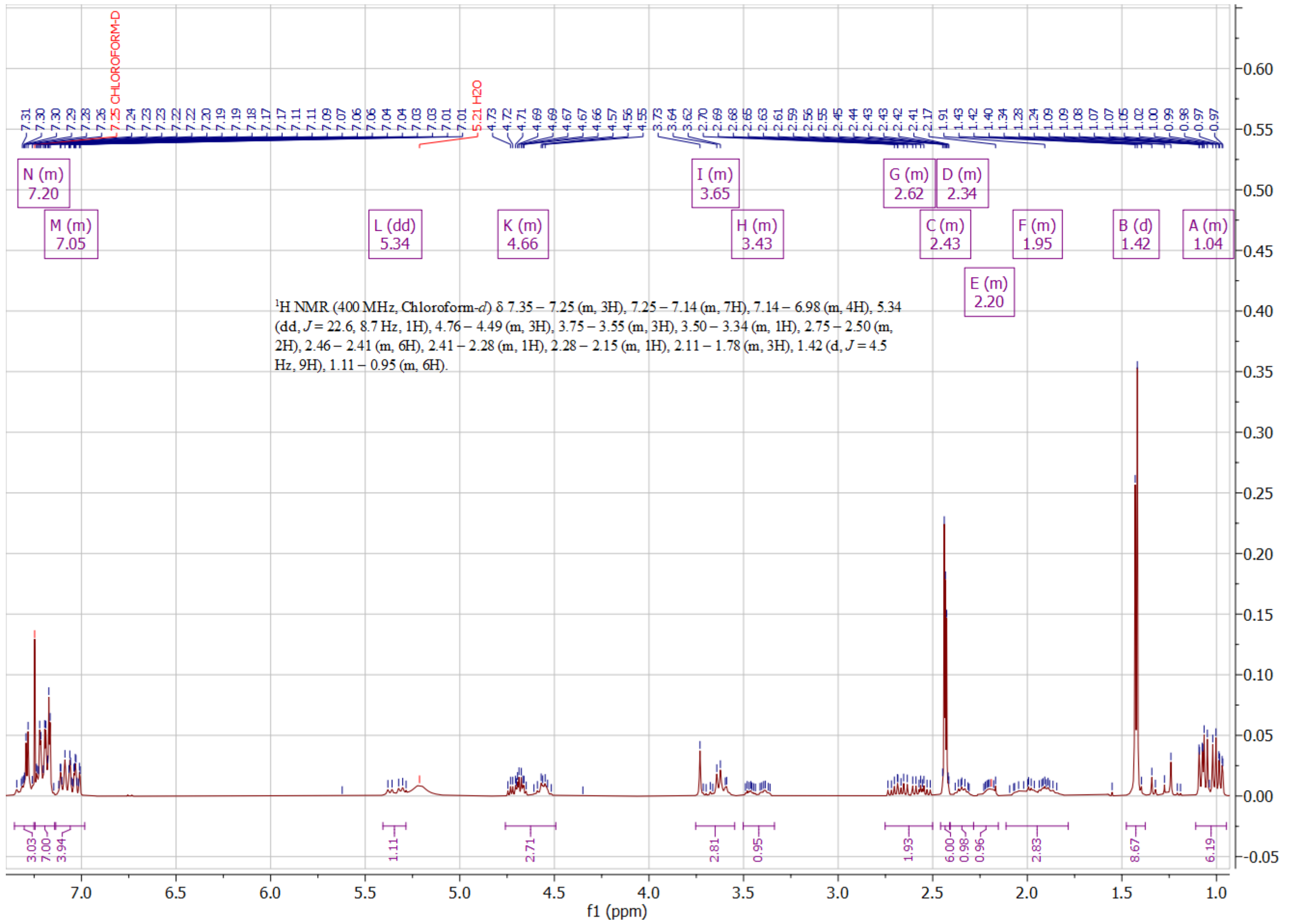
1H NMR (400 MHz, Chloroform-d) δ 7.35 – 7.25 (m, 3H), 7.25 – 7.14 (m, 7H), 7.14 – 6.98 (m, 4H), 5.34 (dd, J = 22.6, 8.7 Hz, 1H), 4.76 – 4.49 (m, 3H), 3.75 – 3.55 (m, 3H), 3.50 – 3.34 (m, 1H), 2.75 – 2.50 (m, 2H), 2.46 – 2.41 (m, 6H), 2.41 – 2.28 (m, 1H), 2.28 – 2.15 (m, 1H), 2.11 – 1.78 (m, 3H), 1.42 (d, J = 4.5 Hz, 9H), 1.11 – 0.95 (m, 6H).
f1 (ppm)

(102) Boc-D-Cys(Bzl)-L-Pro-Val^P(OPh-4-SCH$_3$)$_2$

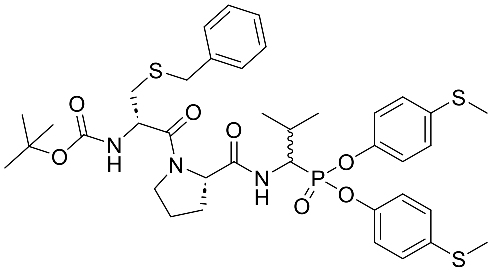

Name: *tert*-butyl ((2S)-3-(benzylthio)-1-((2S)-2-((1-(bis(4-(methylthio)phenoxy)phosphoryl)-2-methylpropyl)carbamoyl)pyrrolidin-1-yl)-1-oxopropan-2-yl)carbamate

^{31}P NMR (162 MHz, Chloroform-d) δ 18.72 (s, 52%), 18.28 (s, 48%).

^{1}H NMR (400 MHz, Chloroform-d) δ 7.64 – 7.51 (m, 1H, NH), 7.35 – 7.25 (m, 4H, 4xArH), 7.24 – 7.09 (m, 7H, 7xArH), 7.09 – 6.99 (m, 2H, 2xArH), 5.36 (dd, *J* = 87.1, 8.5 Hz, 1H, NH), 4.75 – 4.35 (m, 3H, CHP, 2xCH), 3.76 – 3.66 (m, 2H, CH$_2$), 3.58 – 3.24 (m, 2H, CH$_2$), 2.81 – 2.58 (m, 2H, CH$_2$), 2.45 – 2.39 (m, 6H, 2xSCH$_3$), 2.38 – 2.30 (m, 2H, 0.5xCH$_2$, CH), 2.09 – 1.96 (m, 1H, 0.5xCH$_2$), 1.95 – 1.73 (m, 2H, CH$_2$), 1.37 (d, *J* = 22.5 Hz, 9H, Boc), 1.09 – 0.93 (m, 6H, 2xCH$_3$).

HRMS: $C_{38}H_{50}N_3O_7PS_3$ [M+Na]$^+$ calc. 810.2446 found 810.2432

HPLC: purity >99%; retention time 20.047 min

Chromatogram

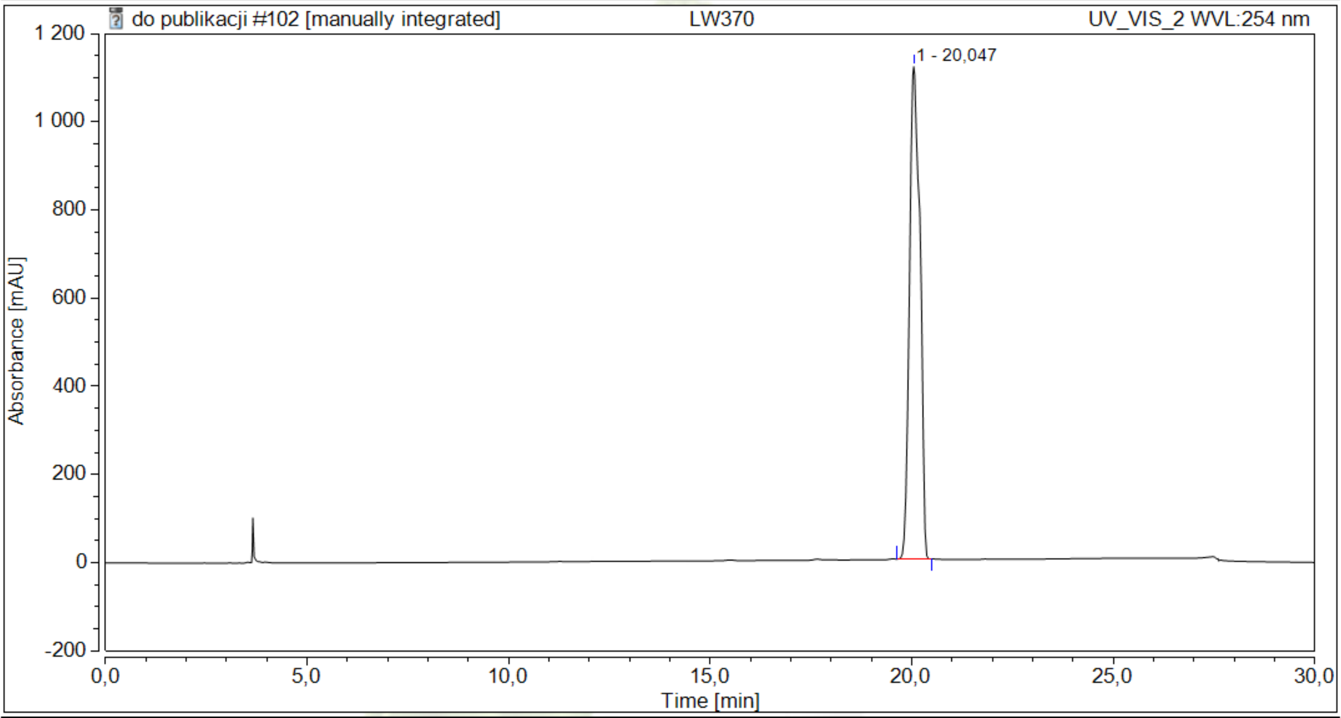

Integration Results

No.	Peak Name	Retention Time min	Area mAU*min	Height mAU	Relative Area %	Relative Height %	Amount n.a.
1		20,047	336,444	1116,895	100,00	100,00	n.a.
**Total:**			**336,444**	**1116,895**	**100,00**	**100,00**	

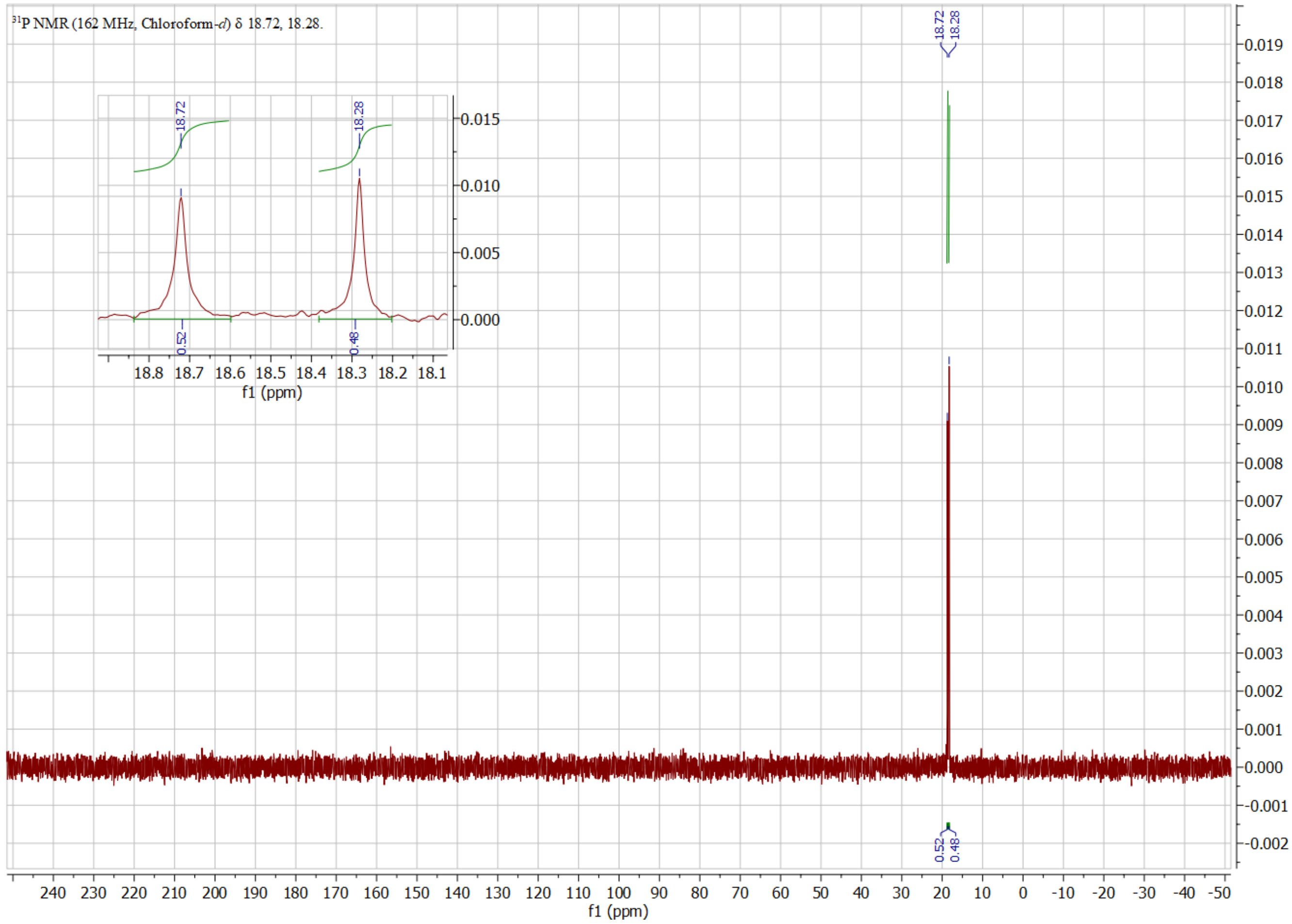

³¹P NMR (162 MHz, Chloroform-d) δ 18.72, 18.28.
f1 (ppm)

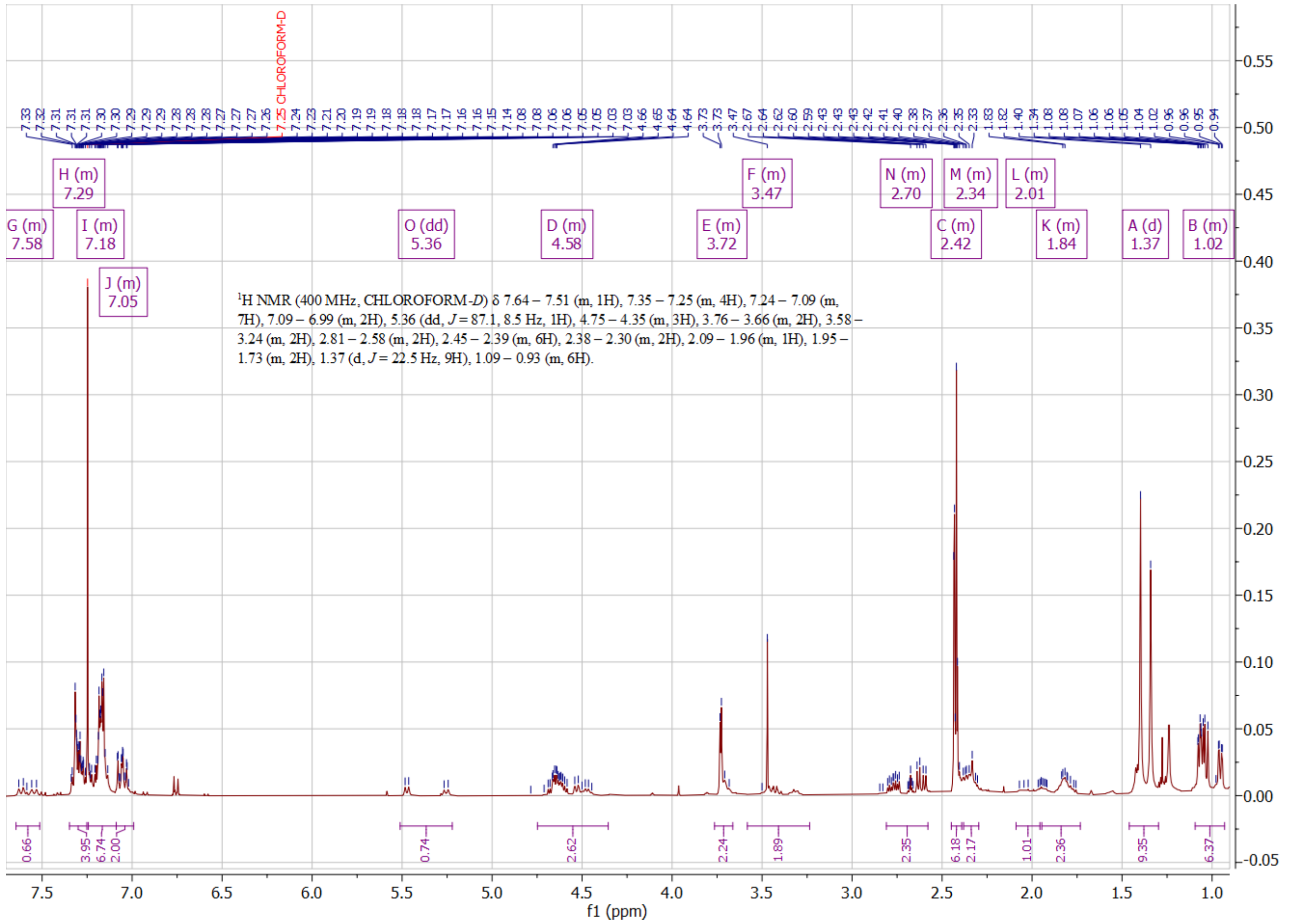

CHLOROFORM-D
¹H NMR (400 MHz, CHLOROFORM-D) δ 7.64 – 7.51 (m, 1H), 7.35 – 7.25 (m, 4H), 7.24 – 7.09 (m, 7H), 7.09 – 6.99 (m, 2H), 5.36 (dd, J = 87.1, 8.5 Hz, 1H), 4.75 – 4.35 (m, 3H), 3.76 – 3.66 (m, 2H), 3.58 – 3.24 (m, 2H), 2.81 – 2.58 (m, 2H), 2.45 – 2.39 (m, 6H), 2.38 – 2.30 (m, 2H), 2.09 – 1.96 (m, 1H), 1.95 – 1.73 (m, 2H), 1.37 (d, J = 22.5 Hz, 9H), 1.09 – 0.93 (m, 6H).
f1 (ppm)

(103) Boc-L-Asp(Bzl)-L-Pro- Val^{P}(OPh-4-SCH$_3$)$_2$

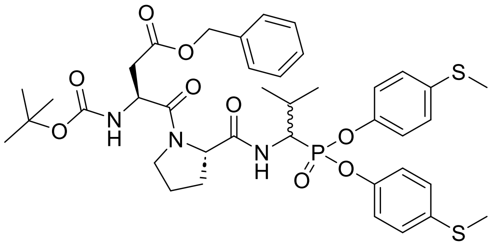

Name: benzyl (3S)-4-((2S)-2-((1-(bis(4-(methylthio)phenoxy)phosphoryl)-2-methylpropyl)carbamoyl)pyrrolidin-1-yl)-3-((*tert*-butoxycarbonyl)amino)-4-oxobutanoate

^{31}P NMR (162 MHz, Chloroform-d) δ 18.58 (s, 48%), 18.19 (s, 52%).

^{1}H NMR (400 MHz, Chloroform-d) δ 7.45 – 7.32 (m, 1H, NH), 7.32 – 7.27 (m, 3H, 3xArH), 7.22 – 7.10 (m, 8H, 8xArH), 7.06 – 6.97 (m, 2H, 2xArH), 5.26 (dd, J = 21.4, 9.3 Hz, 1H, NH), 5.12 – 4.85 (m, 2H, CH$_2$), 4.85 – 4.77 (m, 1H, CHP), 4.75 – 4.62 (m, 2H, 2xCH), 3.86 – 3.69 (m, 2H, CH$_2$), 2.97 – 2.86 (m, 1H, 0.5xCH$_2$), 2.72 – 2.62 (m, 1H, 0.5xCH$_2$), 2.43 – 2.38 (m, 6H, 2xSCH$_3$), 2.40 – 2.17 (m, 2H, 0.5xCH$_2$, CH), 2.12 – 1.81 (m, 3H, 1.5xCH$_2$), 1.41 (d, J = 1.4 Hz, 9H, Boc), 1.13 – 0.93 (m, 6H, 2xCH$_3$).

HRMS: $C_{39}H_{50}N_3O_9PS_2$ $[M+Na]^+$ calc. 822.2624 found 822.2603

HPLC: purity >99%; retention time 19.520 min

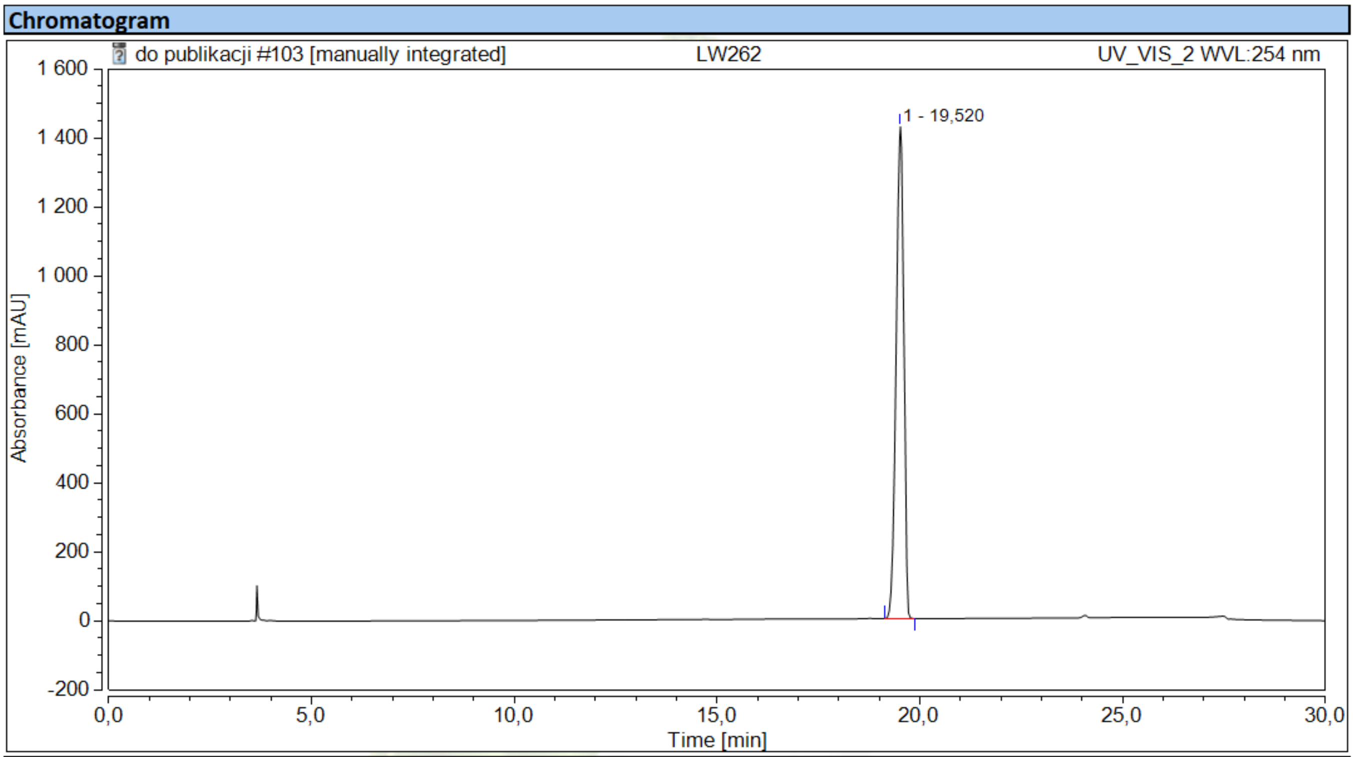

Integration Results

No.	Peak Name	Retention Time min	Area mAU*min	Height mAU	Relative Area %	Relative Height %	Amount n.a.
1		19,520	312,780	1425,797	100,00	100,00	n.a.
**Total:**			**312,780**	**1425,797**	**100,00**	**100,00**	

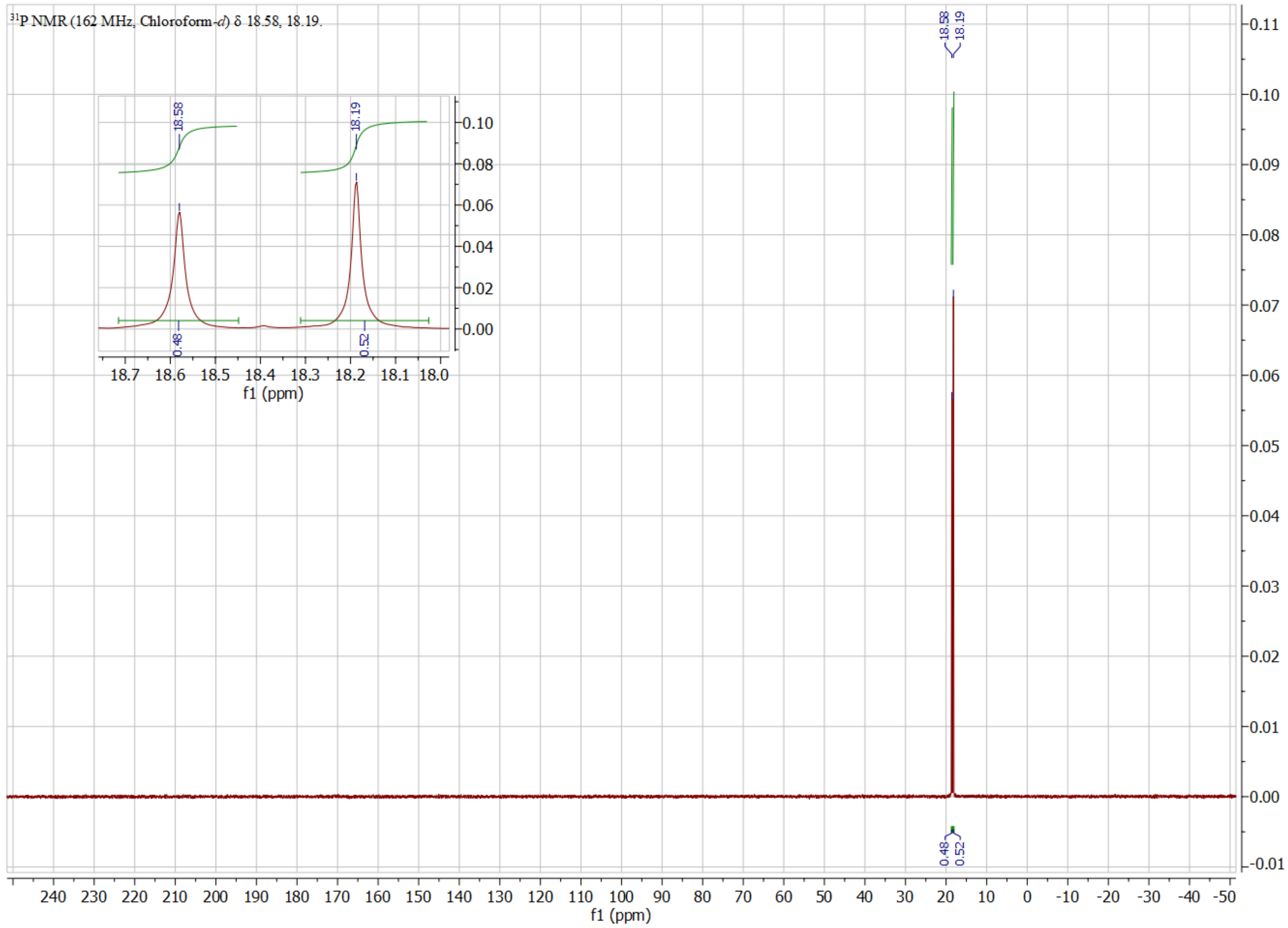

31P NMR (162 MHz, Chloroform-d) δ 18.58, 18.19.
f1 (ppm)

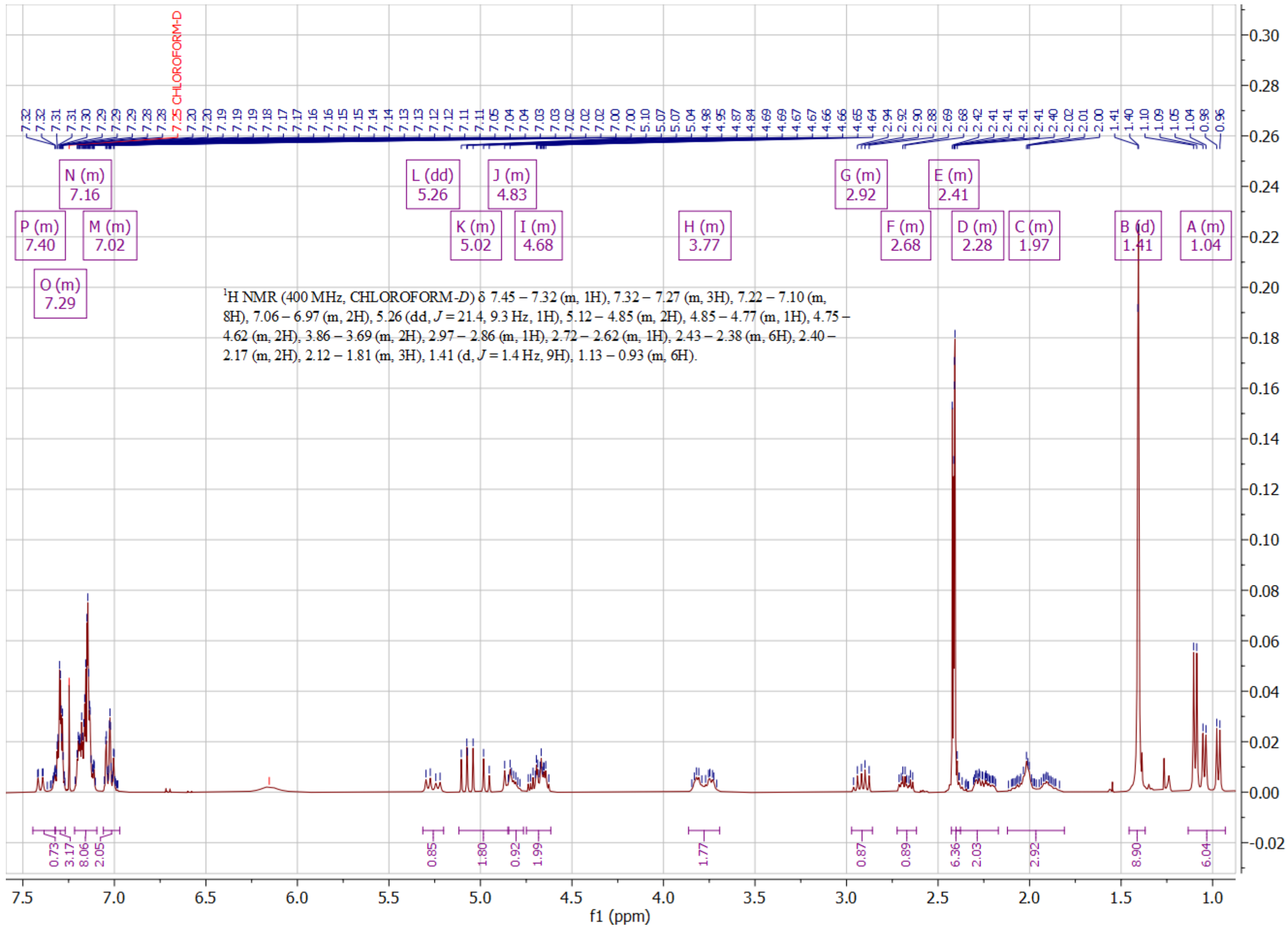

1H NMR (400 MHz, CHLOROFORM-D) δ 7.45 – 7.32 (m, 1H), 7.32 – 7.27 (m, 3H), 7.22 – 7.10 (m, 8H), 7.06 – 6.97 (m, 2H), 5.26 (dd, J = 21.4, 9.3 Hz, 1H), 5.12 – 4.85 (m, 2H), 4.85 – 4.77 (m, 1H), 4.75 – 4.62 (m, 2H), 3.86 – 3.69 (m, 2H), 2.97 – 2.86 (m, 1H), 2.72 – 2.62 (m, 1H), 2.43 – 2.38 (m, 6H), 2.40 – 2.17 (m, 2H), 2.12 – 1.81 (m, 3H), 1.41 (d, J = 1.4 Hz, 9H), 1.13 – 0.93 (m, 6H).
f1 (ppm)

(104) Boc-D-Asp(Bzl)-L-Pro- Val^P(OPh-4-SCH$_3$)$_2$

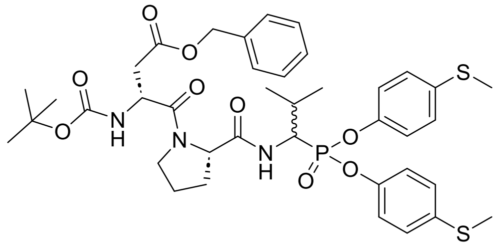

Name: benzyl (3R)-4-((2S)-2-((1-(bis(4-(methylthio)phenoxy)phosphoryl)-2-methylpropyl)carbamoyl)pyrrolidin-1-yl)-3-((*tert*-butoxycarbonyl)amino)-4-oxobutanoate

^{31}P NMR (162 MHz, Chloroform-d) δ 18.78 (s, 47%) 18.24 (s, 53%).

^{1}H NMR (400 MHz, Chloroform-d) δ 7.57 (dd, J = 131.1, 10.3 Hz, 1H, NH), 7.38 – 7.27 (m, 5H, 5xArH), 7.21 – 7.11 (m, 6H, 6xArH), 7.10 – 7.03 (m, 2H, 2xArH), 5.52 (dd, J = 127.1, 8.9 Hz, 1H, NH), 5.15 – 4.97 (m, 2H, CH$_2$), 4.93 – 4.45 (m, 3H, CHP, 2xCH), 3.84 – 3.60 (m, 1H, 0.5xCH$_2$), 3.59 – 3.44 (m, 1H, 0.5xCH$_2$), 2.99 – 2.86 (m, 1H, 0.5xCH$_2$), 2.75 – 2.64 (m, 1H, 0.5xCH$_2$), 2.45 – 2.40 (m, 6H, 2xSCH$_3$), 2.40 – 2.22 (m, 2H, 0.5xCH$_2$, CH), 2.10 – 1.90 (m, 1H, 0.5xCH$_2$), 1.90 – 1.70 (m, 2H, CH$_2$), 1.35 (d, J = 22.4 Hz, 9H, Boc), 1.09 – 0.95 (m, 6H, 2xCH$_3$).

HRMS: $C_{39}H_{50}N_3O_9PS_2$ [M+Na]$^+$ calc. 822.2624 found 822.2617

HPLC: purity >98%; retention time 19.503 and 19.667 min

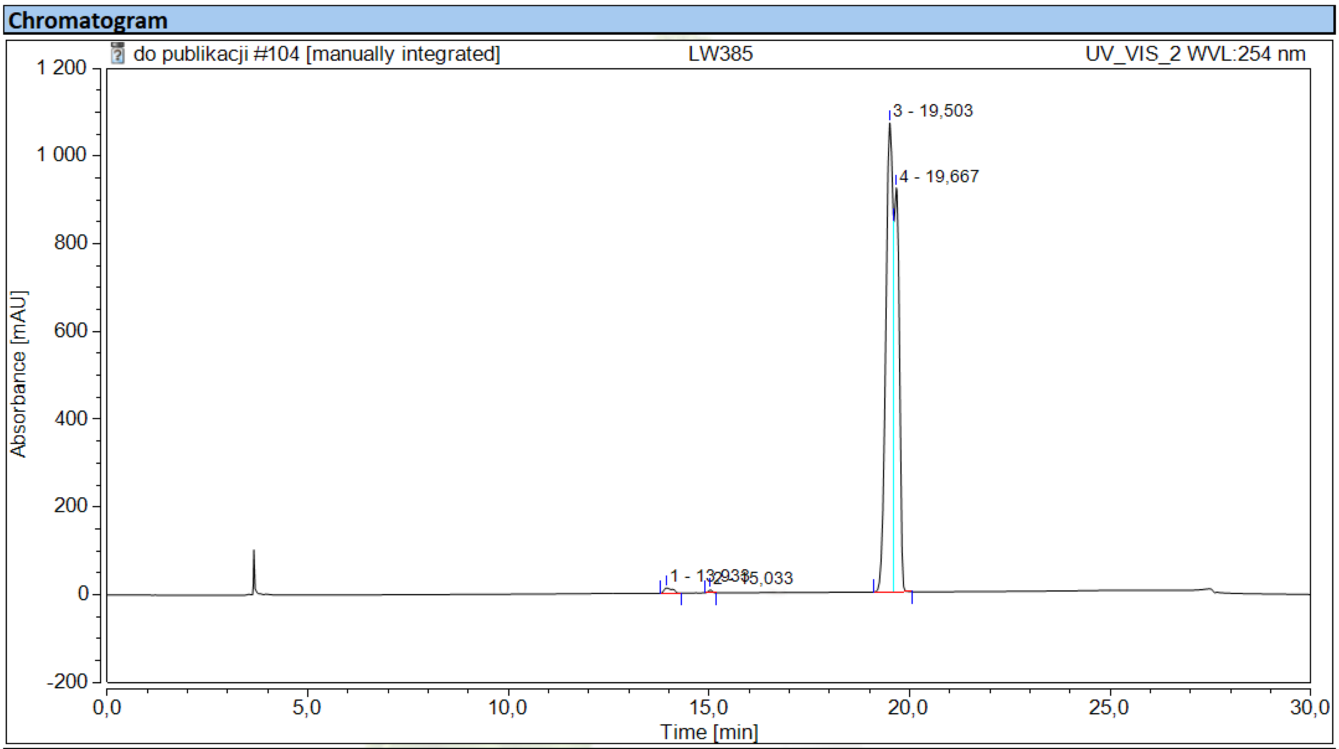

Integration Results

No.	Peak Name	Retention Time min	Area mAU*min	Height mAU	Relative Area %	Relative Height %	Amount n.a.
1		13,933	3,023	12,309	0,83	0,61	n.a.
2		15,033	0,779	6,792	0,21	0,34	n.a.
3		19,503	217,680	1069,098	59,74	53,21	n.a.
4		19,667	142,872	921,015	39,21	45,84	n.a.
**Total:**			**364,354**	**2009,214**	**100,00**	**100,00**	

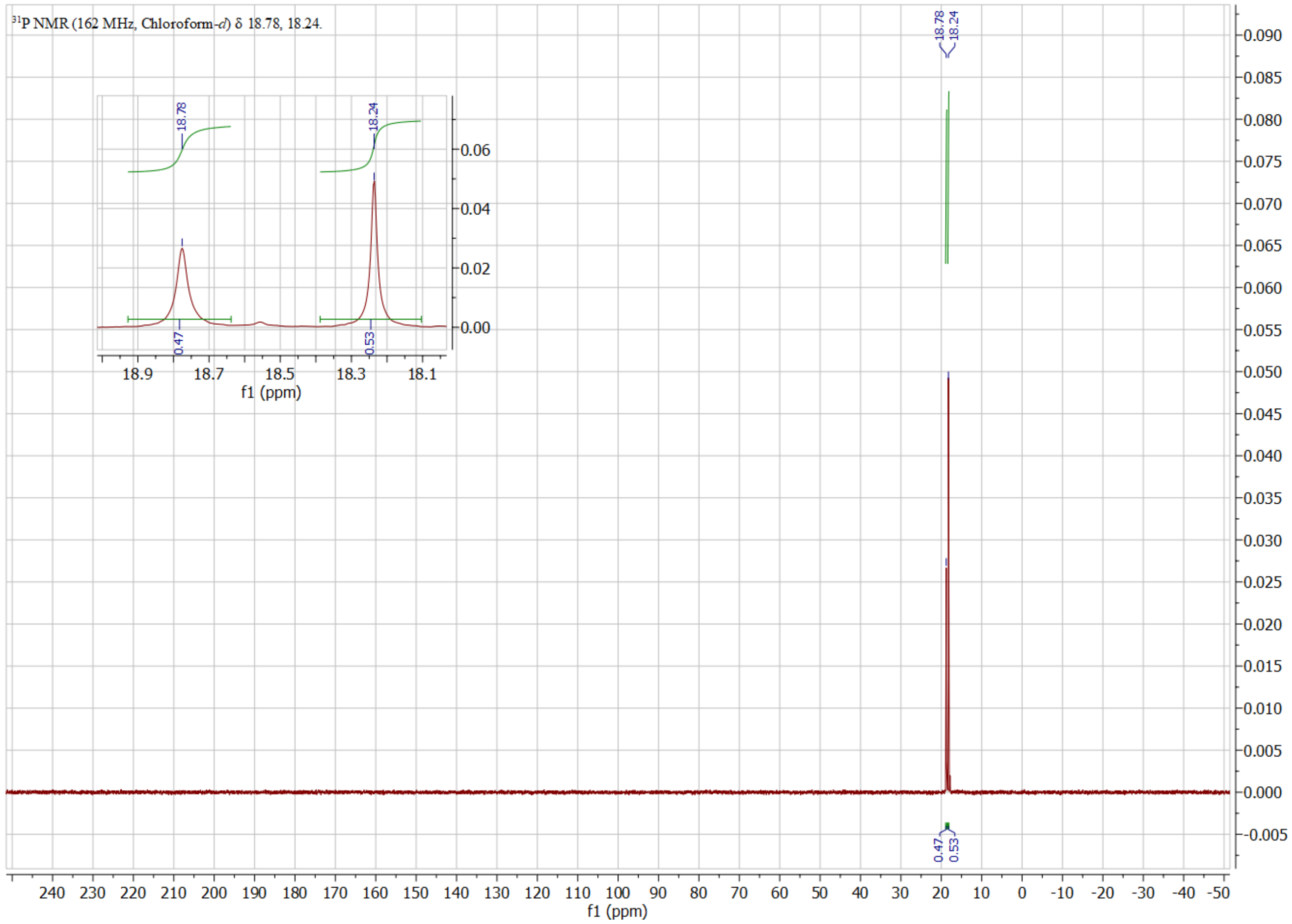

31P NMR (162 MHz, Chloroform-d) δ 18.78, 18.24.
f1 (ppm)

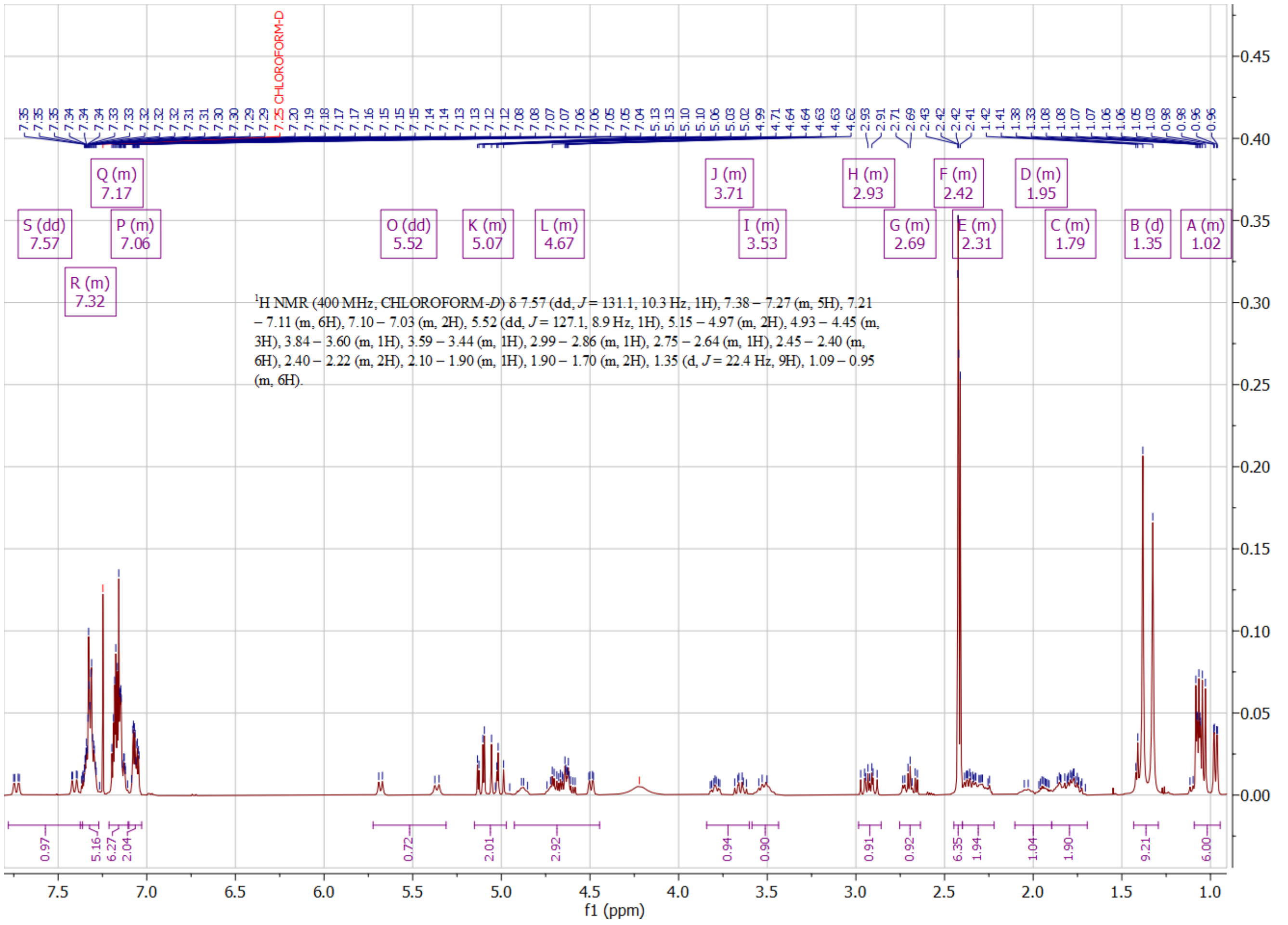

1H NMR (400 MHz, CHLOROFORM-D) δ 7.57 (dd, J = 131.1, 10.3 Hz, 1H), 7.38 – 7.27 (m, 5H), 7.21 – 7.11 (m, 6H), 7.10 – 7.03 (m, 2H), 5.52 (dd, J = 127.1, 8.9 Hz, 1H), 5.15 – 4.97 (m, 2H), 4.93 – 4.45 (m, 3H), 3.84 – 3.60 (m, 1H), 3.59 – 3.44 (m, 1H), 2.99 – 2.86 (m, 1H), 2.75 – 2.64 (m, 1H), 2.45 – 2.40 (m, 6H), 2.40 – 2.22 (m, 2H), 2.10 – 1.90 (m, 1H), 1.90 – 1.70 (m, 2H), 1.35 (d, J = 22.4 Hz, 9H), 1.09 – 0.95 (m, 6H).
CHLOROFORM-D
f1 (ppm)

(105) Boc-L-Glu(Bzl)-L-Pro- Val^P(OPh-4-SCH$_3$)$_2$

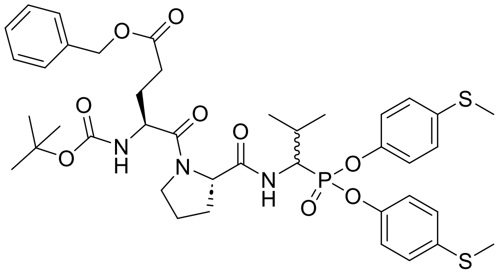

Name: benzyl (4S)-5-((2S)-2-((1-(bis(4-(methylthio)phenoxy)phosphoryl)-2-methylpropyl)carbamoyl)pyrrolidin-1-yl)-4-((*tert*-butoxycarbonyl)amino)-5-oxopentanoate

^{31}P NMR (162 MHz, Chloroform-d) δ 18.26 (s, 50%), 18.06 (s, 50%).

^{1}H NMR (400 MHz, Chloroform-d) δ 7.47 (dd, J = 36.8, 10.6 Hz, 1H, NH), 7.36 – 7.28 (m, 5H, 5xArH), 7.21 – 7.14 (m, 4H, 4xArH), 7.14 – 7.01 (m, 4H, 4xArH), 5.41 (dd, J = 20.2, 7.7 Hz, 1H, NH), 5.11 (s, 2H, CH$_2$), 4.76 – 4.64 (m, 1H, CHP), 4.60 – 4.39 (m, 2H, 2xCH), 3.79 – 3.45 (m, 2H, CH$_2$), 2.59 – 2.44 (m, 2H, CH$_2$), 2.44 – 2.40 (m, 6H, 2xSCH$_3$), 2.40 – 2.30 (m, 1H, CH), 2.27 – 2.15 (m, 1H, 0.5xCH$_2$), 2.15 – 1.88 (m, 3H, 1.5xCH$_2$), 1.88 – 1.75 (m, 2H, CH$_2$), 1.36 (d, J = 16.4 Hz, 9H, Boc), 1.12 – 0.93 (m, 6H, 2xCH$_3$).

HRMS: $C_{40}H_{52}N_3O_9PS_2$ $[M+Na]^+$ calc. 836.2780 found 836.2769

HPLC: purity >99%; retention time 19.603 min

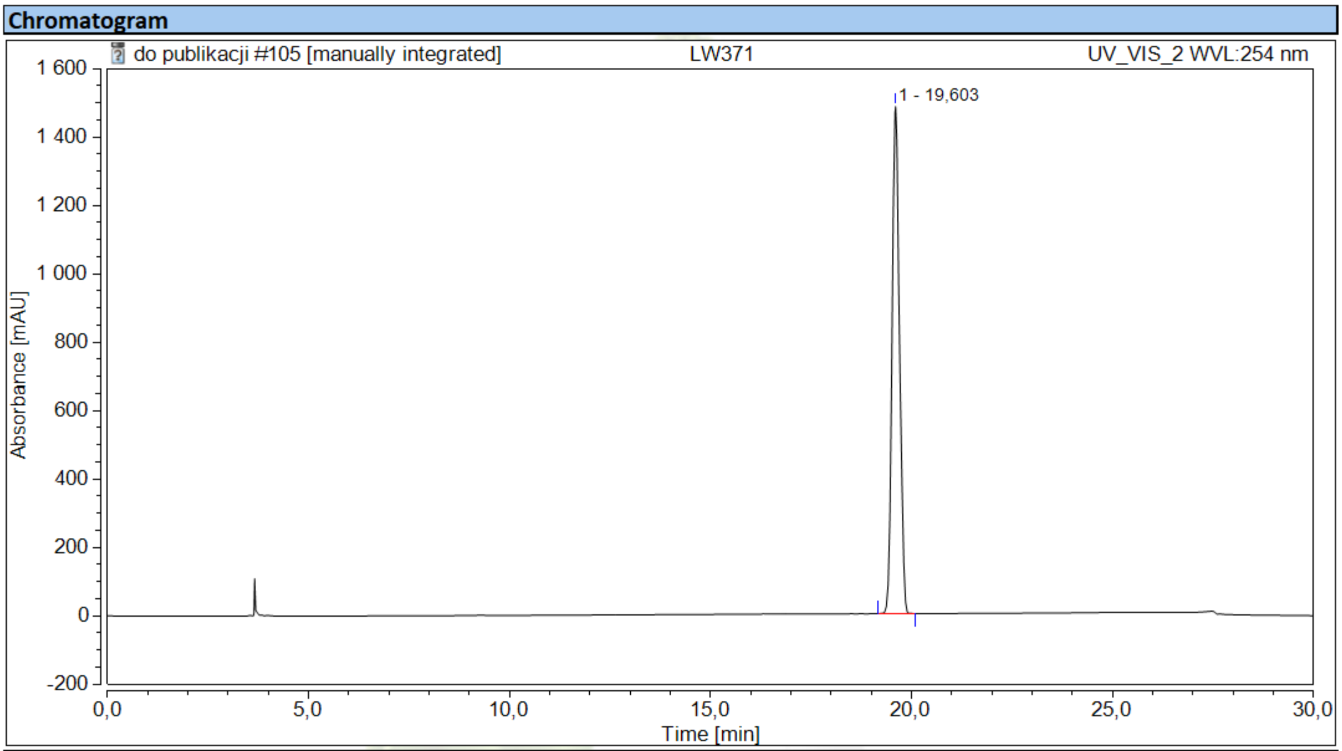

Integration Results

No.	Peak Name	Retention Time min	Area mAU*min	Height mAU	Relative Area %	Relative Height %	Amount n.a.
1		19,603	316,657	1482,385	100,00	100,00	n.a.
**Total:**			**316,657**	**1482,385**	**100,00**	**100,00**	

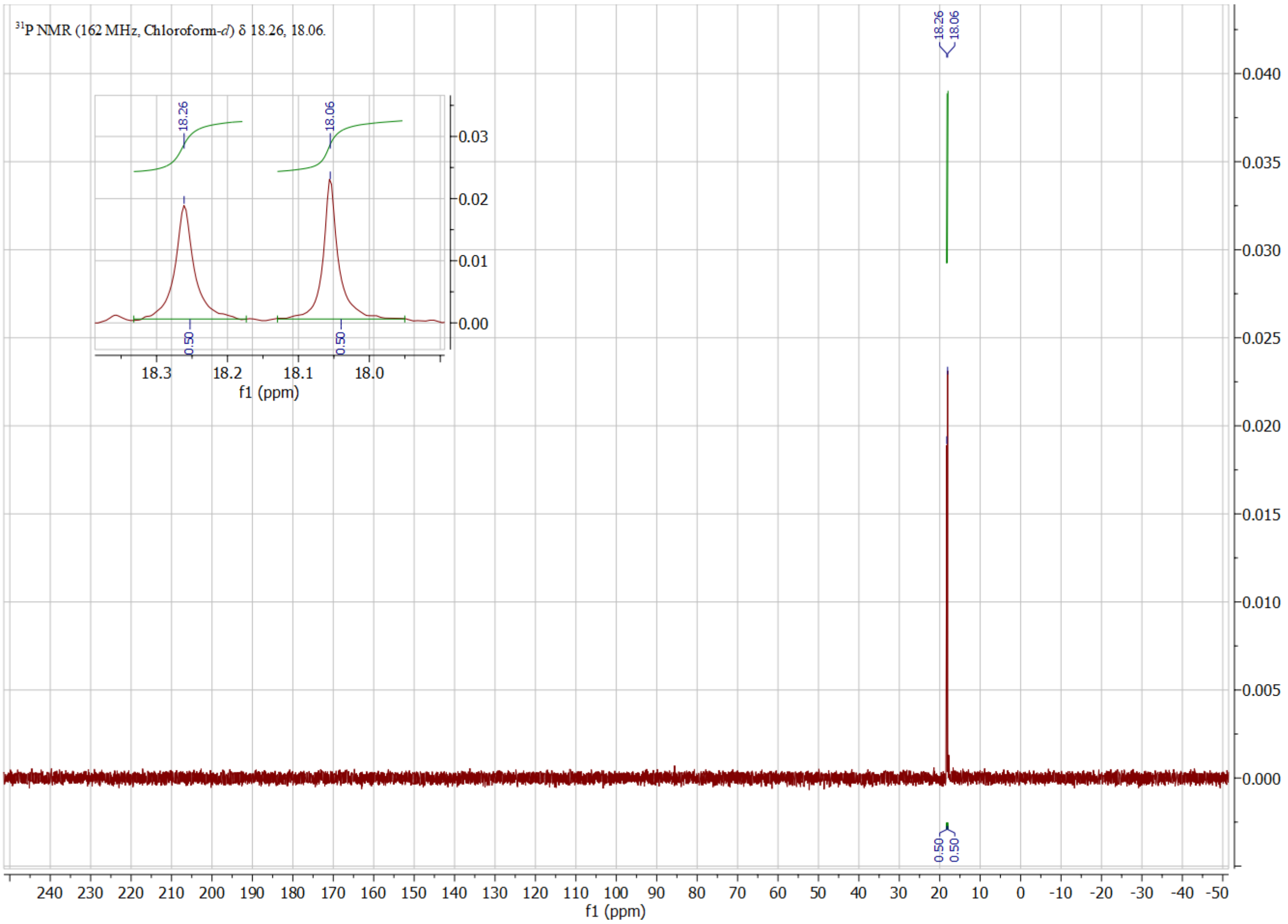

^{31}P NMR (162 MHz, Chloroform-*d*) δ 18.26, 18.06.
18.26
18.06
0.50
0.50
f1 (ppm)

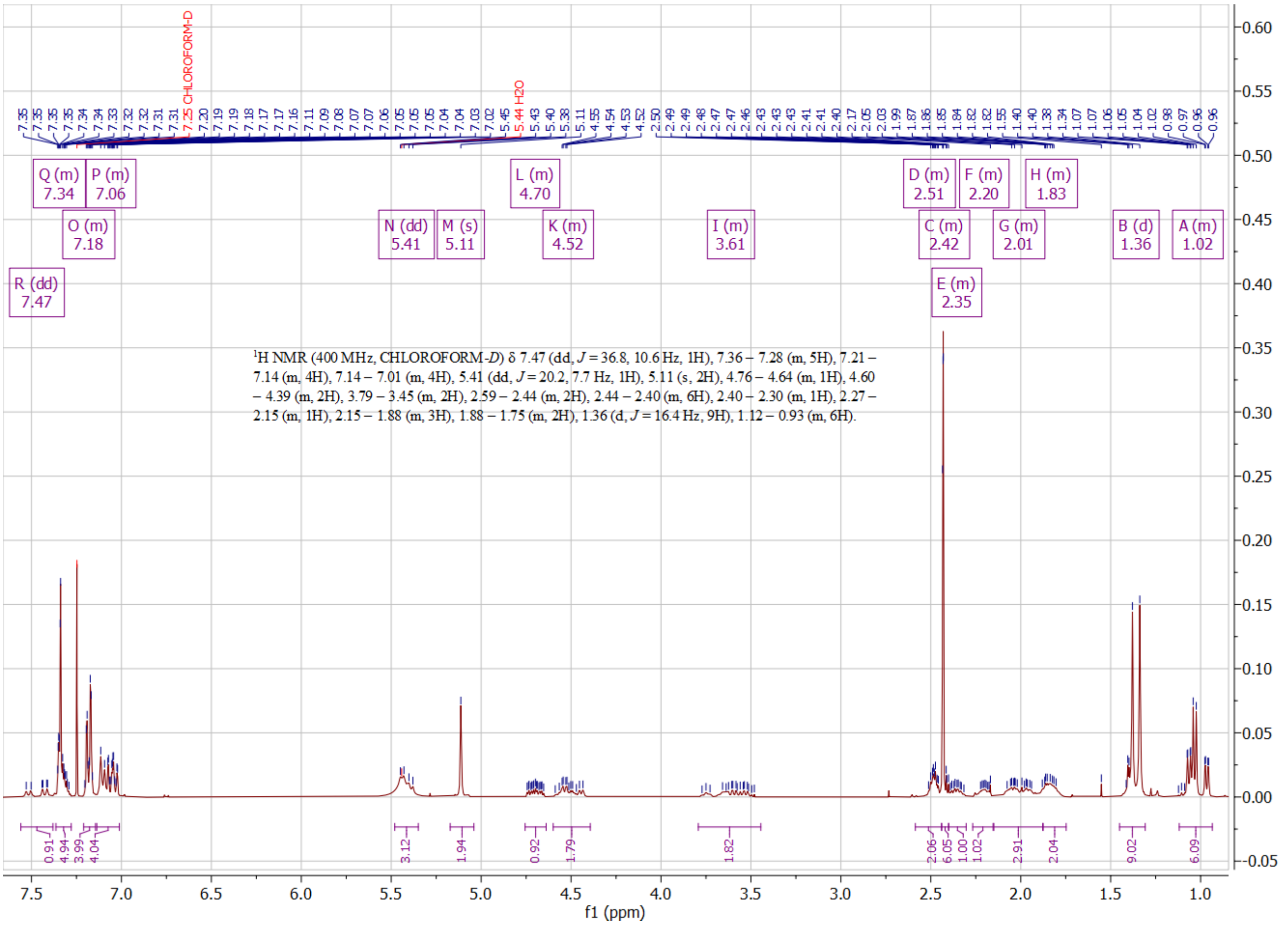

7.25 CHLOROFORM-D
5.44 H2O
Q (m) 7.34
P (m) 7.06
O (m) 7.18
R (dd) 7.47
N (dd) 5.41
M (s) 5.11
L (m) 4.70
K (m) 4.52
I (m) 3.61
D (m) 2.51
F (m) 2.20
H (m) 1.83
C (m) 2.42
G (m) 2.01
E (m) 2.35
B (d) 1.36
A (m) 1.02
^{1}H NMR (400 MHz, CHLOROFORM-*D*) δ 7.47 (dd, *J* = 36.8, 10.6 Hz, 1H), 7.36 – 7.28 (m, 5H), 7.21 – 7.14 (m, 4H), 7.14 – 7.01 (m, 4H), 5.41 (dd, *J* = 20.2, 7.7 Hz, 1H), 5.11 (s, 2H), 4.76 – 4.64 (m, 1H), 4.60 – 4.39 (m, 2H), 3.79 – 3.45 (m, 2H), 2.59 – 2.44 (m, 2H), 2.44 – 2.40 (m, 6H), 2.40 – 2.30 (m, 1H), 2.27 – 2.15 (m, 1H), 2.15 – 1.88 (m, 3H), 1.88 – 1.75 (m, 2H), 1.36 (d, *J* = 16.4 Hz, 9H), 1.12 – 0.93 (m, 6H).
f1 (ppm)

(106) Boc-L-His(Bzl)-L-Pro-Val^{P}(OPh-4-SCH$_3$)$_2$

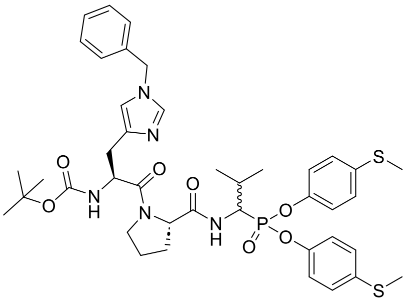

Name: *tert*-butyl ((2S)-3-(1-benzyl-1H-imidazol-4-yl)-1-((2S)-2-((1-(bis(4-(methylthio)phenoxy)phosphoryl)-2-methylpropyl)carbamoyl)pyrrolidin-1-yl)-1-oxopropan-2-yl)carbamate

^{31}P NMR (162 MHz, Chloroform-d) δ 18.55 (s,68%), 18.05 (s,32%).

^{1}H NMR (400 MHz, Chloroform-d) δ 7.43 – 7.27 (m, 4H, 3xArH, NH), 7.23 – 6.90 (m, 12H, 10xArH, 2xCH), 5.72 (dd, J = 16.9, 8.2 Hz, 1H, NH), 5.11 (d, J = 35.9 Hz, 2H, CH$_2$), 4.78 – 4.40 (m, 3H, CHP, 2xCH), 3.68 – 3.42 (m, 2H, CH$_2$), 3.18 – 2.68 (m, 2H, CH$_2$), 2.47 – 2.37 (m, 8H, 2xSCH$_3$, CH$_2$), 2.25 – 2.15 (m, 1H, CH), 2.03 – 1.83 (m, 3H, 1.5xCH$_2$), 1.32 (d, J = 6.5 Hz, 9H, Boc), 1.12 – 1.00 (m, 6H, 2xCH$_3$).

HRMS: $C_{41}H_{52}N_5O_7PS_2$ [M+Na]$^+$ calc. 844.2943 found 844.2914

HPLC: purity >99%; retention time 14.860 and 15.187 min

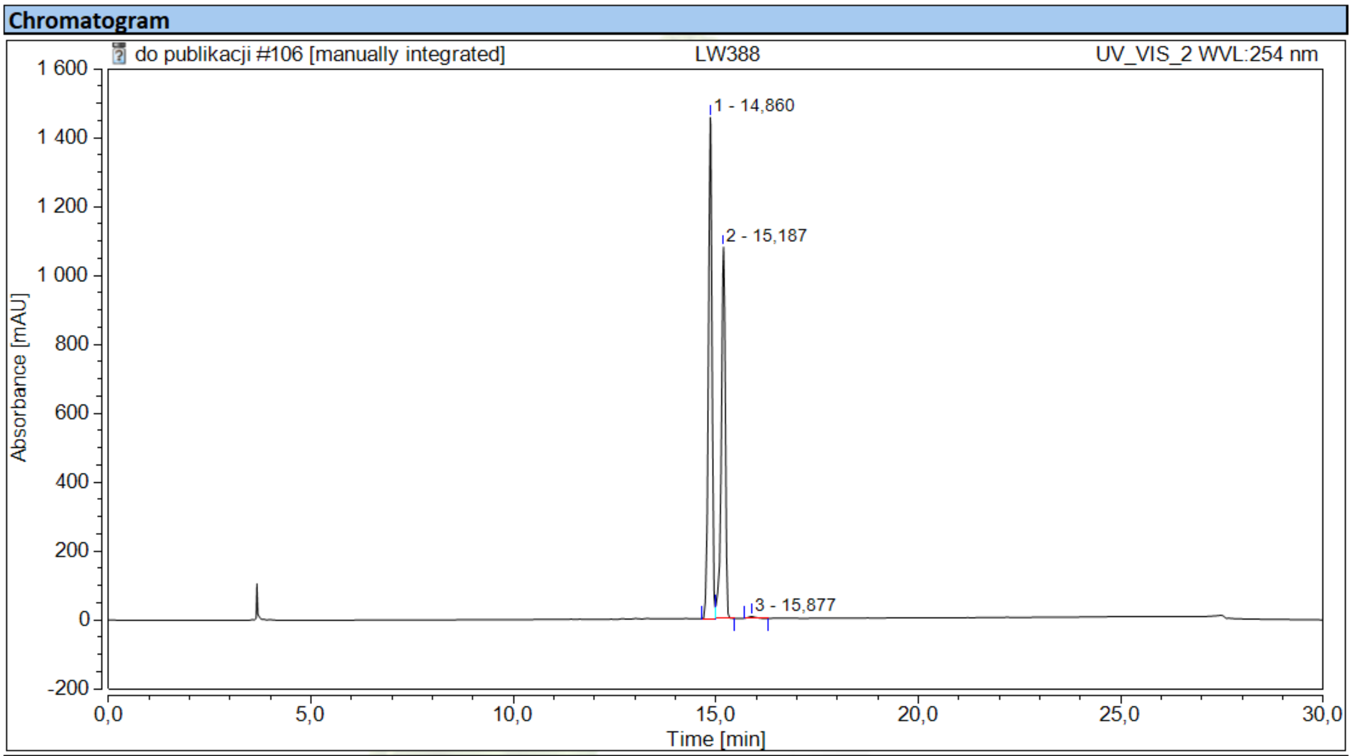

Integration Results

No.	Peak Name	Retention Time min	Area mAU*min	Height mAU	Relative Area %	Relative Height %	Amount n.a.
1		14,860	151,070	1455,037	54,65	57,33	n.a.
2		15,187	124,343	1077,908	44,98	42,47	n.a.
3		15,877	1,032	5,198	0,37	0,20	n.a.
**Total:**			**276,446**	**2538,143**	**100,00**	**100,00**	

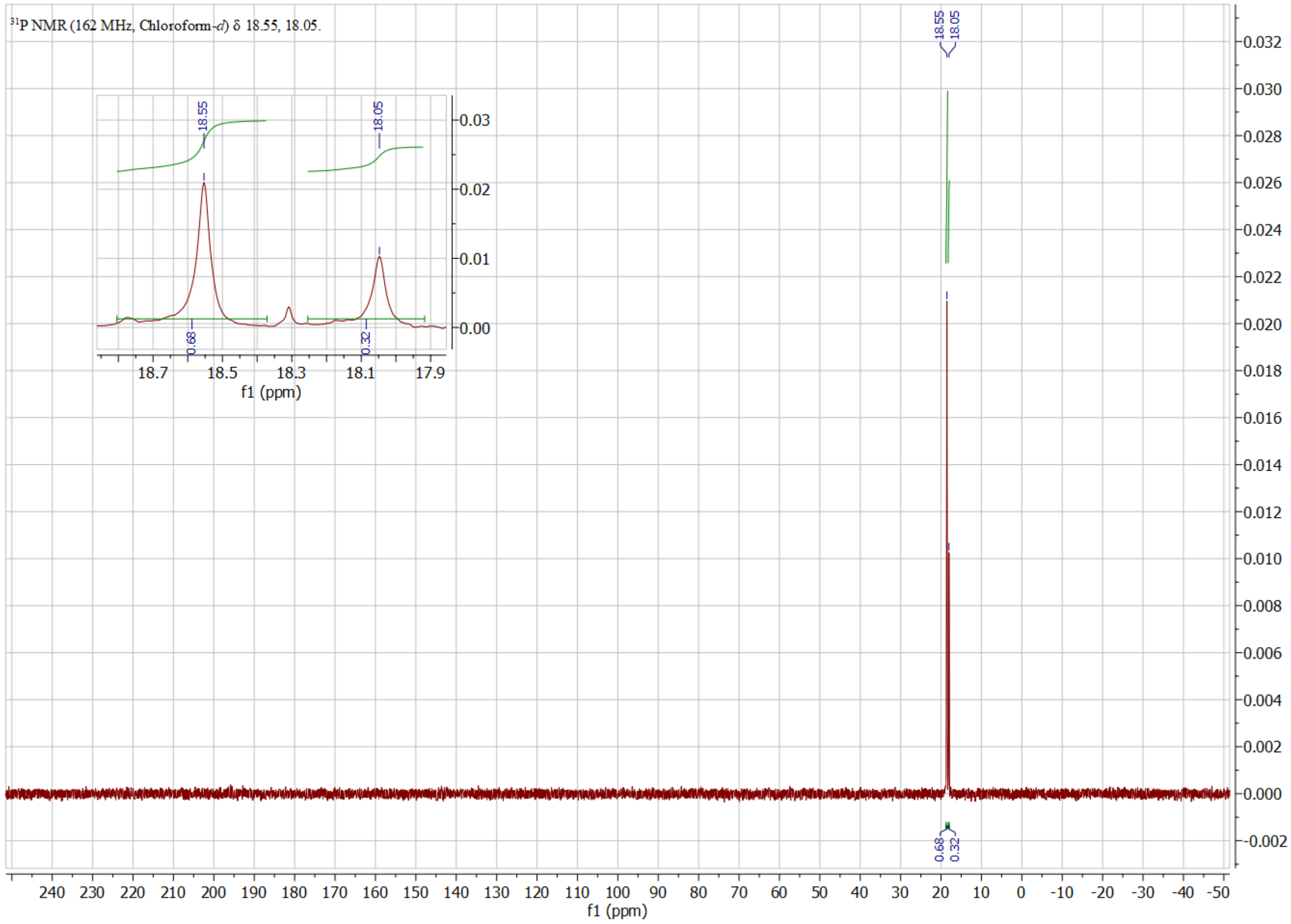

^{31}P NMR (162 MHz, Chloroform-*d*) δ 18.55, 18.05.
f1 (ppm)

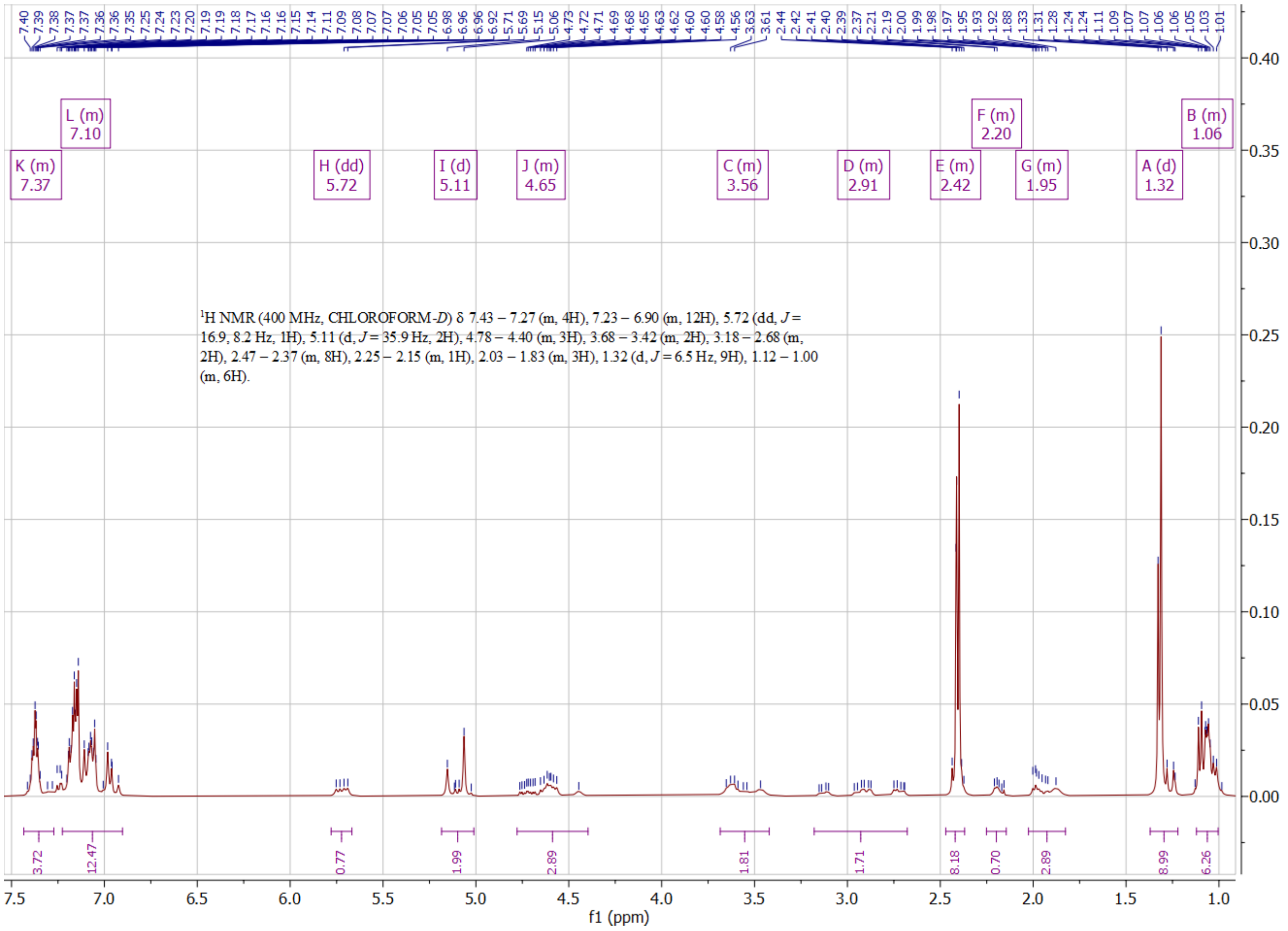

^{1}H NMR (400 MHz, CHLOROFORM-*D*) δ 7.43 – 7.27 (m, 4H), 7.23 – 6.90 (m, 12H), 5.72 (dd, *J* = 16.9, 8.2 Hz, 1H), 5.11 (d, *J* = 35.9 Hz, 2H), 4.78 – 4.40 (m, 3H), 3.68 – 3.42 (m, 2H), 3.18 – 2.68 (m, 2H), 2.47 – 2.37 (m, 8H), 2.25 – 2.15 (m, 1H), 2.03 – 1.83 (m, 3H), 1.32 (d, *J* = 6.5 Hz, 9H), 1.12 – 1.00 (m, 6H).
f1 (ppm)

(107) Boc-L-Orn(Z)-L-Pro-Val^P(OPh-4-SCH_3)$_2$

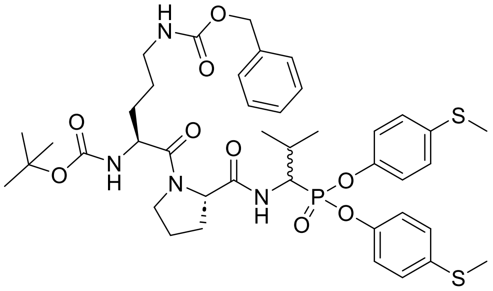

Name: benzyl *tert*-butyl ((4S)-5-((2S)-2-((1-(bis(4-(methylthio)phenoxy)phosphoryl)-2-methylpropyl)carbamoyl)pyrrolidin-1-yl)-5-oxopentane-1,4-diyl)dicarbamate
^{31}P NMR (162 MHz, Chloroform-d) δ 17.90 (s, 48%), 17.73 (s, 52%).
^{1}H NMR (400 MHz, Chloroform-d) δ 7.51 – 7.25 (m, 6H, 5xArH, NH), 7.23 – 6.91 (m, 8H, 8xArH), 5.32 (dd, J = 50.4, 8.6 Hz, 1H, NH), 5.17 – 4.99 (m, 2H, CH_2), 4.80 – 4.60 (m, 2H, CHP, NH), 4.55 – 4.44 (m, 1H, CH), 4.44 – 4.31 (m, 1H, CH), 3.75 – 3.29 (m, 2H, CH_2), 3.26 – 2.88 (m, 2H, CH_2), 2.47 – 2.38 (m, 7H, 2xSCH_3, CH), 2.38 – 1.81 (m, 4H, 2xCH_2), 1.78 – 1.43 (m, 3H, 1.5xCH_2), 1.41 (d, J = 2.2 Hz, 9H, Boc), 1.39 – 1.32 (m, 1H, 0.5xCH_2), 1.12 – 0.94 (m, 6H, 2xCH_3).
HRMS: $C_{41}H_{55}N_4O_9PS_2$ $[M+Na]^+$ calc. 865.3046 found 865.3028
HPLC: purity >99%; retention time 18.633 and 18.970 min

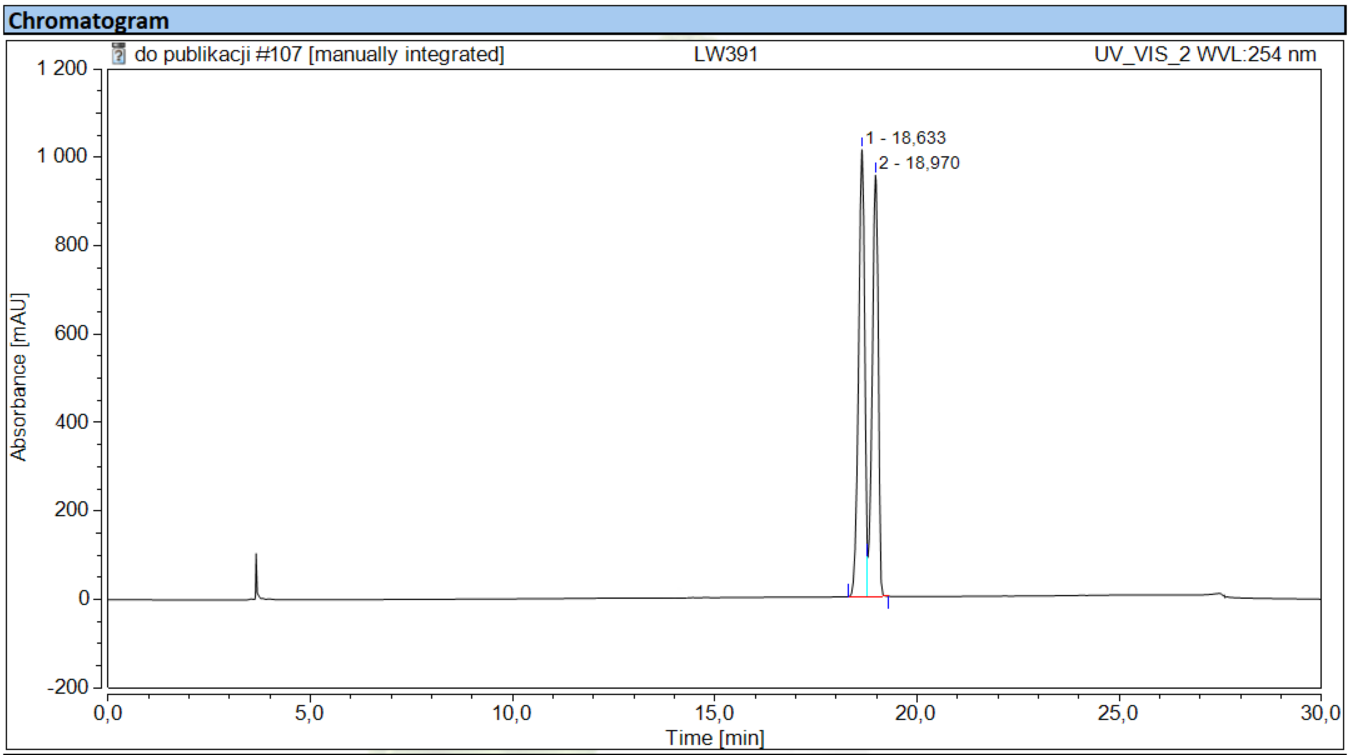

Integration Results

No.	Peak Name	Retention Time min	Area mAU*min	Height mAU	Relative Area %	Relative Height %	Amount n.a.
1		18,633	178,560	1010,931	52,03	51,47	n.a.
2		18,970	164,594	953,133	47,97	48,53	n.a.
**Total:**			**343,154**	**1964,063**	**100,00**	**100,00**	

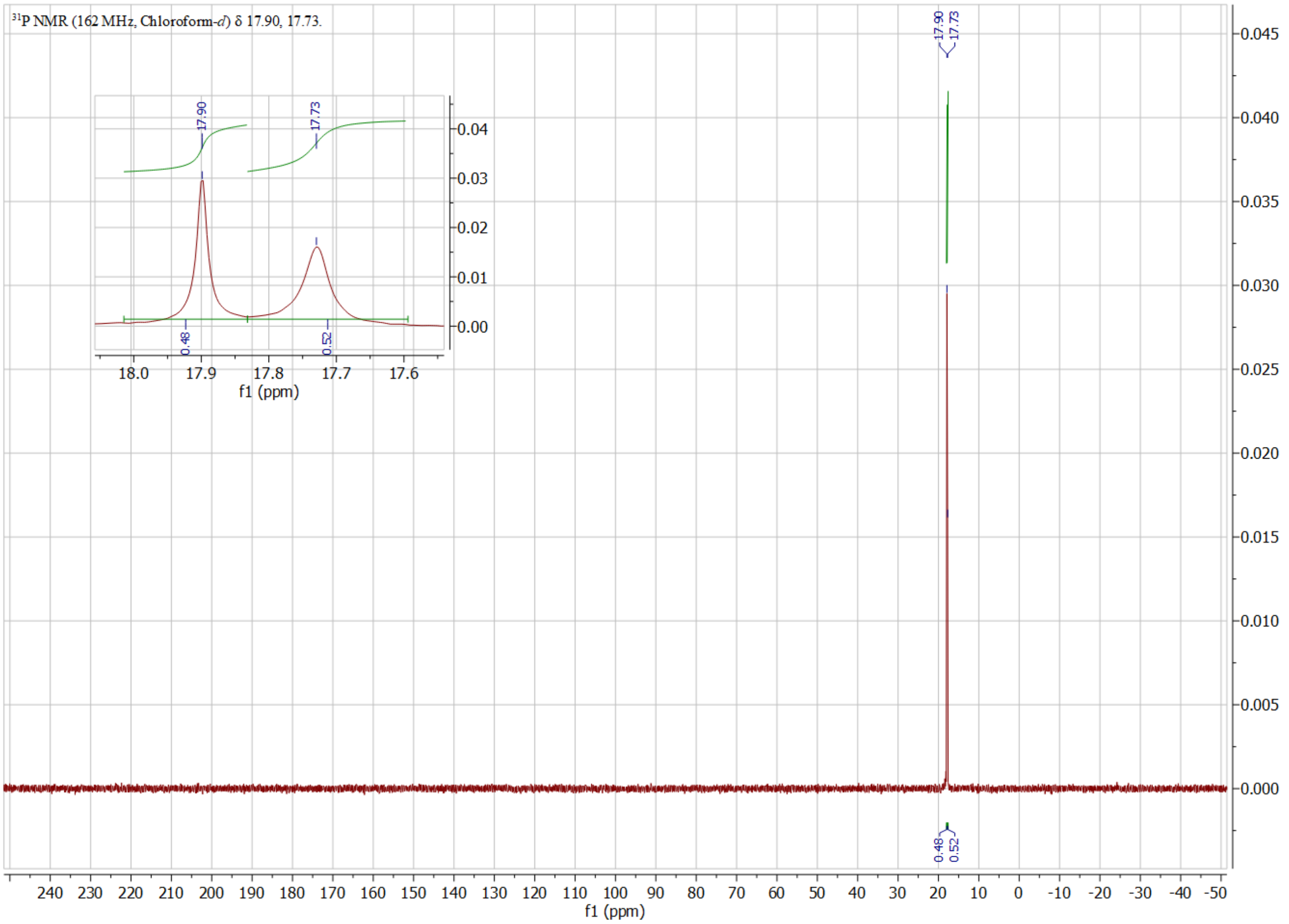

31P NMR (162 MHz, Chloroform-d) δ 17.90, 17.73.
17.90
17.73
0.48
0.52
f1 (ppm)

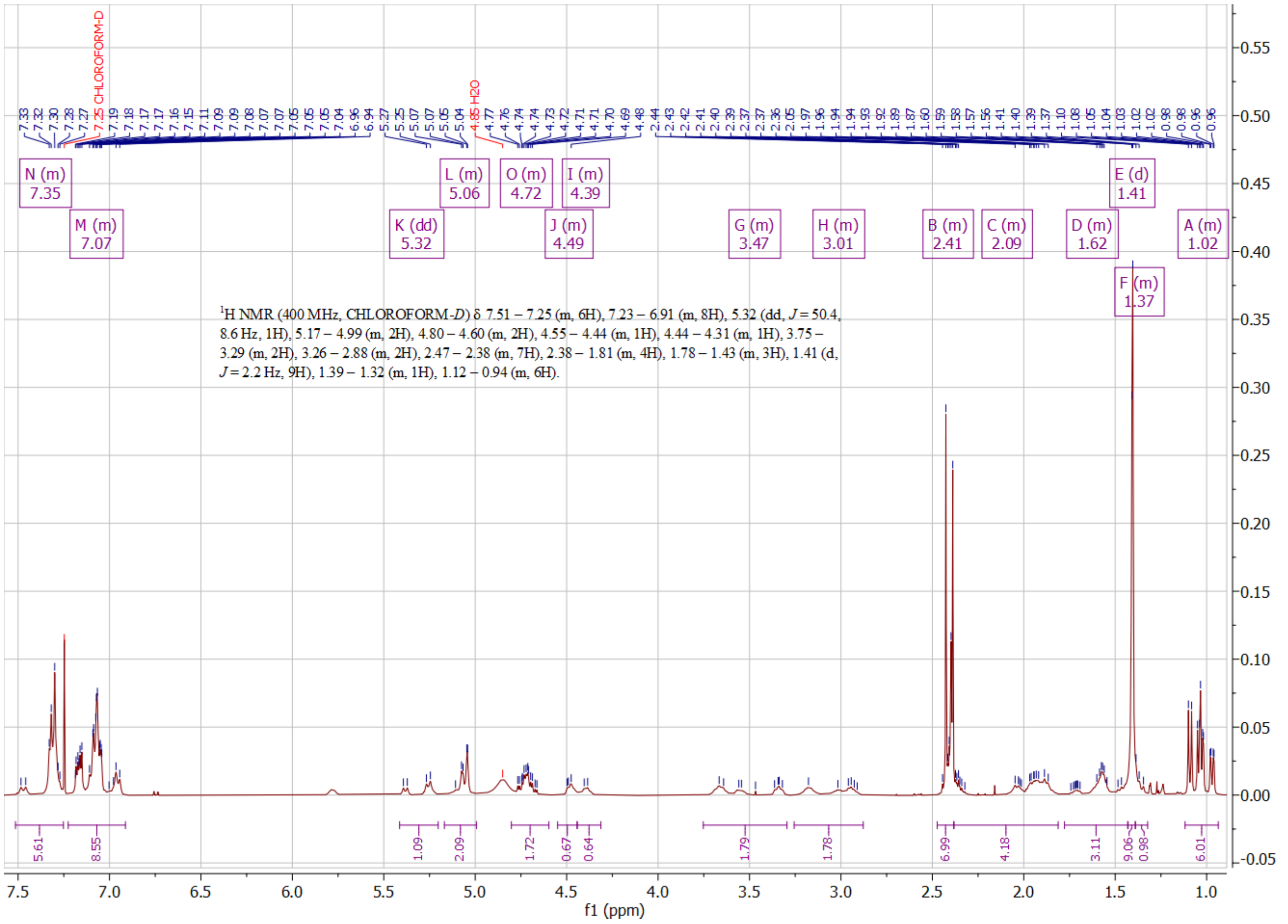

1H NMR (400 MHz, CHLOROFORM-D) δ 7.51 – 7.25 (m, 6H), 7.23 – 6.91 (m, 8H), 5.32 (dd, J = 50.4, 8.6 Hz, 1H), 5.17 – 4.99 (m, 2H), 4.80 – 4.60 (m, 2H), 4.55 – 4.44 (m, 1H), 4.44 – 4.31 (m, 1H), 3.75 – 3.29 (m, 2H), 3.26 – 2.88 (m, 2H), 2.47 – 2.38 (m, 7H), 2.38 – 1.81 (m, 4H), 1.78 – 1.43 (m, 3H), 1.41 (d, J = 2.2 Hz, 9H), 1.39 – 1.32 (m, 1H), 1.12 – 0.94 (m, 6H).
7.25 CHLOROFORM-D
4.85 H2O
N (m) 7.35
M (m) 7.07
K (dd) 5.32
L (m) 5.06
O (m) 4.72
J (m) 4.49
I (m) 4.39
G (m) 3.47
H (m) 3.01
B (m) 2.41
C (m) 2.09
D (m) 1.62
E (d) 1.41
F (m) 1.37
A (m) 1.02
f1 (ppm)

(108) Boc-D-Orn(Z)-L-Pro-Val^P(OPh-4-SCH_3)$_2$

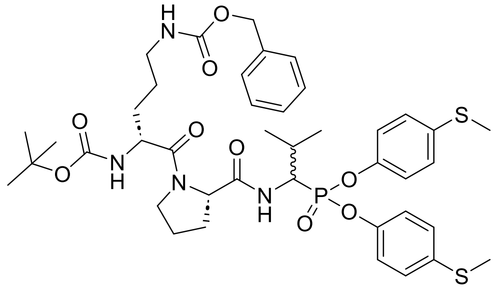

Name: benzyl *tert*-butyl ((4R)-5-((2S)-2-((1-(bis(4-(methylthio)phenoxy)phosphoryl)-2-methylpropyl)carbamoyl)pyrrolidin-1-yl)-5-oxopentane-1,4-diyl)dicarbamate
^{31}P NMR (162 MHz, Chloroform-d) δ 18.51 (s, 52%), 18.19 (s, 48%).
^{1}H NMR (400 MHz, Chloroform-d) δ 7.52 (dd, J = 29.9, 9.4 Hz, 1H, NH), 7.40 – 7.26 (m, 5H, 5xArH), 7.23 – 7.00 (m, 8H, 8xArH), 5.34 (dd, J = 29.7, 8.4 Hz, 1H, NH), 5.17 – 5.01 (m, 2H, CH_2), 4.98 – 4.87 (m, 1H, NH), 4.74 – 4.62 (m, 1H, CHP), 4.62 – 4.49 (m, 1H, CH), 4.50 – 4.34 (m, 1H, CH), 3.83 – 3.32 (m, 2H, CH_2), 3.27 – 3.13 (m, 2H, CH_2), 2.43 (d, J = 3.3 Hz, 6H, 2xSCH_3), 2.41 – 2.02 (m, 3H, CH_2, CH), 2.02 – 1.66 (m, 3H, 1.5xCH_2), 1.66 – 1.46 (m, 3H, 1.5xCH_2), 1.36 (d, J = 19.7 Hz, 9H, Boc), 1.13 – 0.92 (m, 6H, 2xCH_3).
HRMS: $C_{41}H_{55}N_4O_9PS_2$ $[M+Na]^+$ calc. 865.3046 found 865.3023
HPLC: purity >99%; retention time 18.770 min

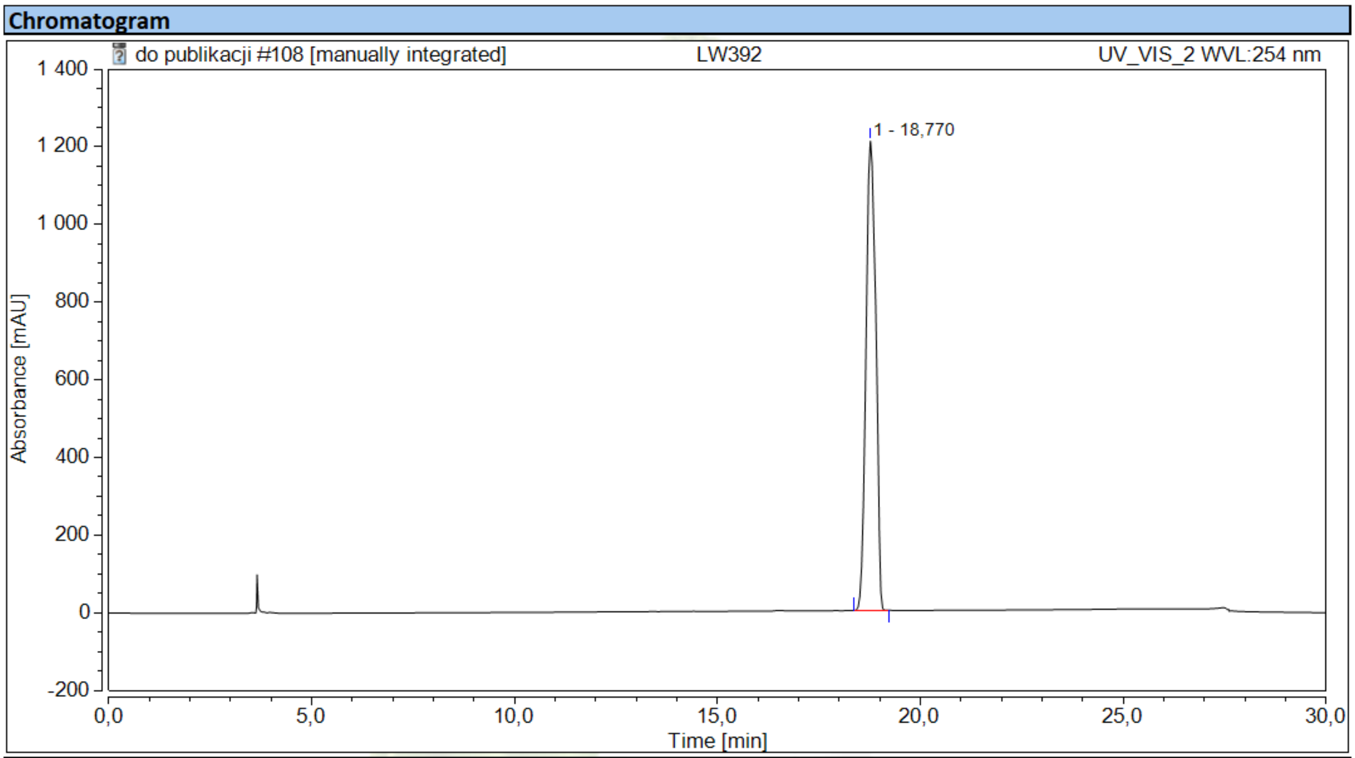

Integration Results

No.	Peak Name	Retention Time min	Area mAU*min	Height mAU	Relative Area %	Relative Height %	Amount n.a.
1		18,770	338,477	1208,222	100,00	100,00	n.a.
**Total:**			**338,477**	**1208,222**	**100,00**	**100,00**	

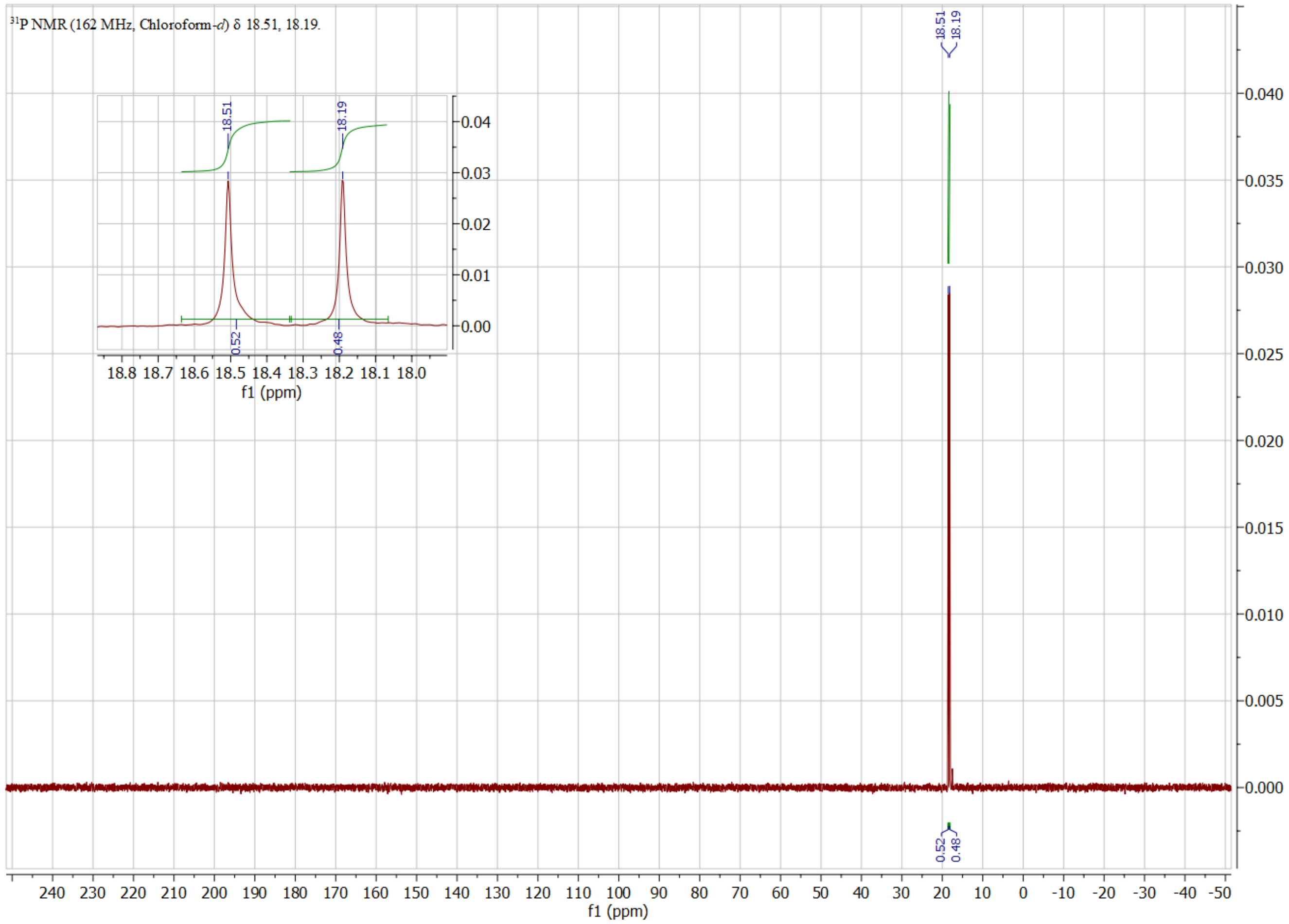
31P NMR (162 MHz, Chloroform-d) δ 18.51, 18.19.
f1 (ppm)

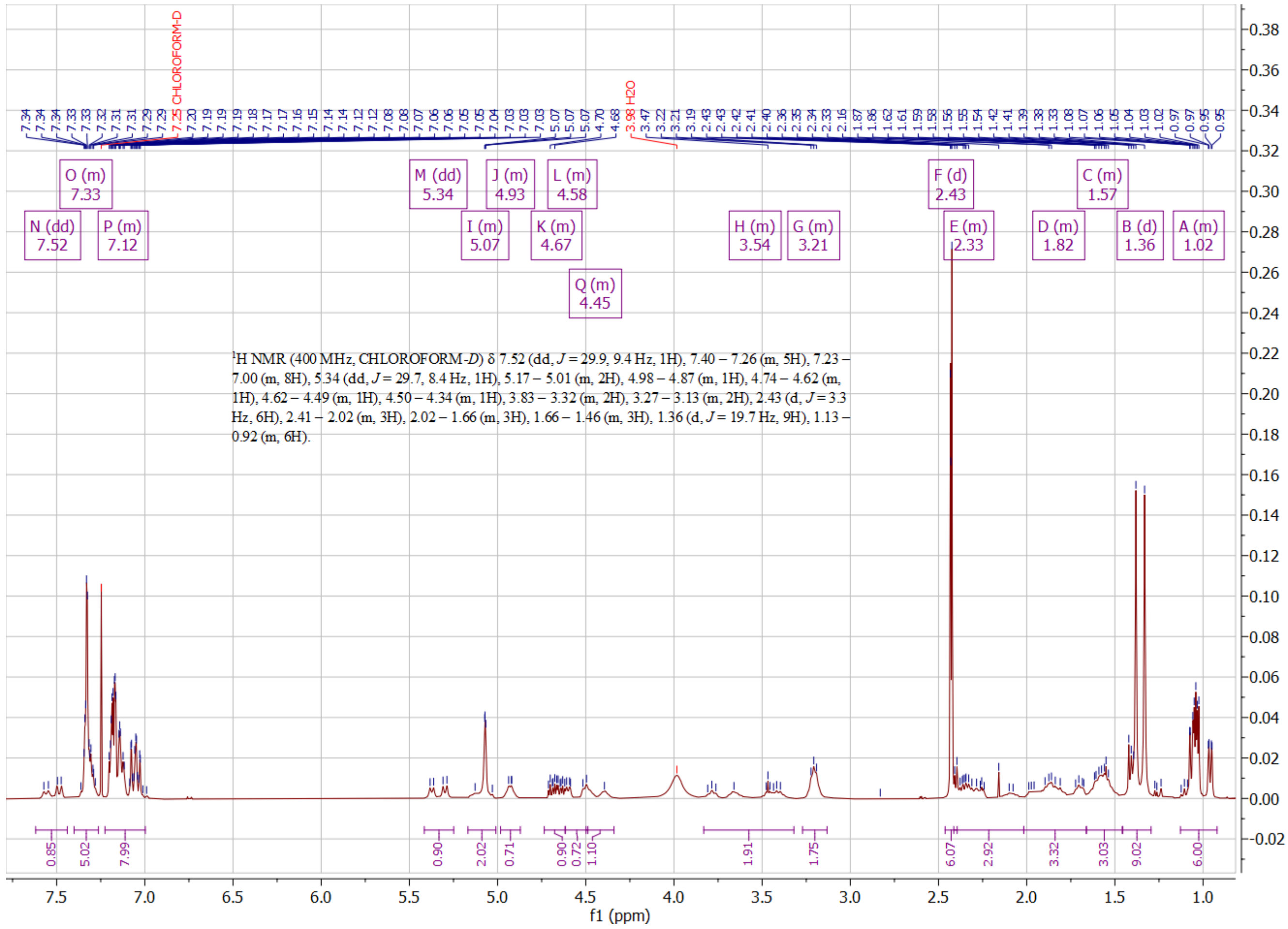
1H NMR (400 MHz, CHLOROFORM-D) δ 7.52 (dd, J = 29.9, 9.4 Hz, 1H), 7.40 – 7.26 (m, 5H), 7.23 – 7.00 (m, 8H), 5.34 (dd, J = 29.7, 8.4 Hz, 1H), 5.17 – 5.01 (m, 2H), 4.98 – 4.87 (m, 1H), 4.74 – 4.62 (m, 1H), 4.62 – 4.49 (m, 1H), 4.50 – 4.34 (m, 1H), 3.83 – 3.32 (m, 2H), 3.27 – 3.13 (m, 2H), 2.43 (d, J = 3.3 Hz, 6H), 2.41 – 2.02 (m, 3H), 2.02 – 1.66 (m, 3H), 1.66 – 1.46 (m, 3H), 1.36 (d, J = 19.7 Hz, 9H), 1.13 – 0.92 (m, 6H).
CHLOROFORM-D
H2O
f1 (ppm)

(109) Boc-L-Lys(Z)-L-Pro-Val^{P}(OPh-4-SCH_3)$_2$

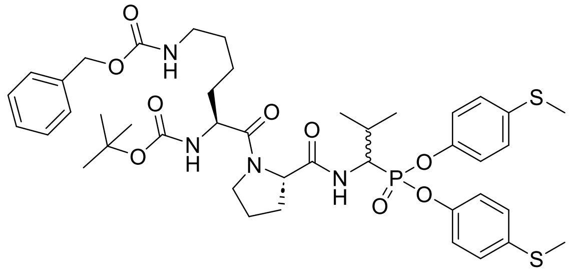

Name: benzyl *tert*-butyl ((5S)-6-((2S)-2-((1-(bis(4-(methylthio)phenoxy)phosphoryl)-2-methylpropyl)carbamoyl)pyrrolidin-1-yl)-6-oxohexane-1,5-diyl)dicarbamate

^{31}P NMR (162 MHz, Chloroform-d) δ 18.25 (s, 54%), 18.10 (s, 46%).

^{1}H NMR (400 MHz, Chloroform-d) δ 7.50 – 7.25 (m, 6H, 5xArH, NH), 7.20 – 6.94 (m, 8H, 8xArH), 5.45 – 5.20 (m, 1H, NH), 5.12 – 5.03 (m, 2H, CH_2), 4.95 – 4.64 (m, 2H, CHP, NH), 4.58 – 3.95 (m, 2H, 2xCH), 3.73 – 3.34 (m, 2H, CH_2), 3.24 – 2.89 (m, 2H, CH_2), 2.46 – 2.40 (m, 6H, 2xSCH_3), 2.40 – 2.19 (m, 2H, 0.5xCH_2, CH), 2.20 – 2.09 (m, 1H, 0.5xCH_2), 2.09 – 1.70 (m, 4H, 2xCH_2), 1.70 – 1.47 (m, 2H, CH_2), 1.39 (d, J = 7.0 Hz, 9H, Boc), 1.32 – 1.24 (m, 2H, CH_2), 1.10 – 0.87 (m, 6H, 2xCH_3).

HRMS: $C_{42}H_{57}N_4O_9PS_2$ [M+Na]$^+$ calc. 879.3202 found 879.3179

HPLC: purity >99%; retention time 18.930 and 19.167 min

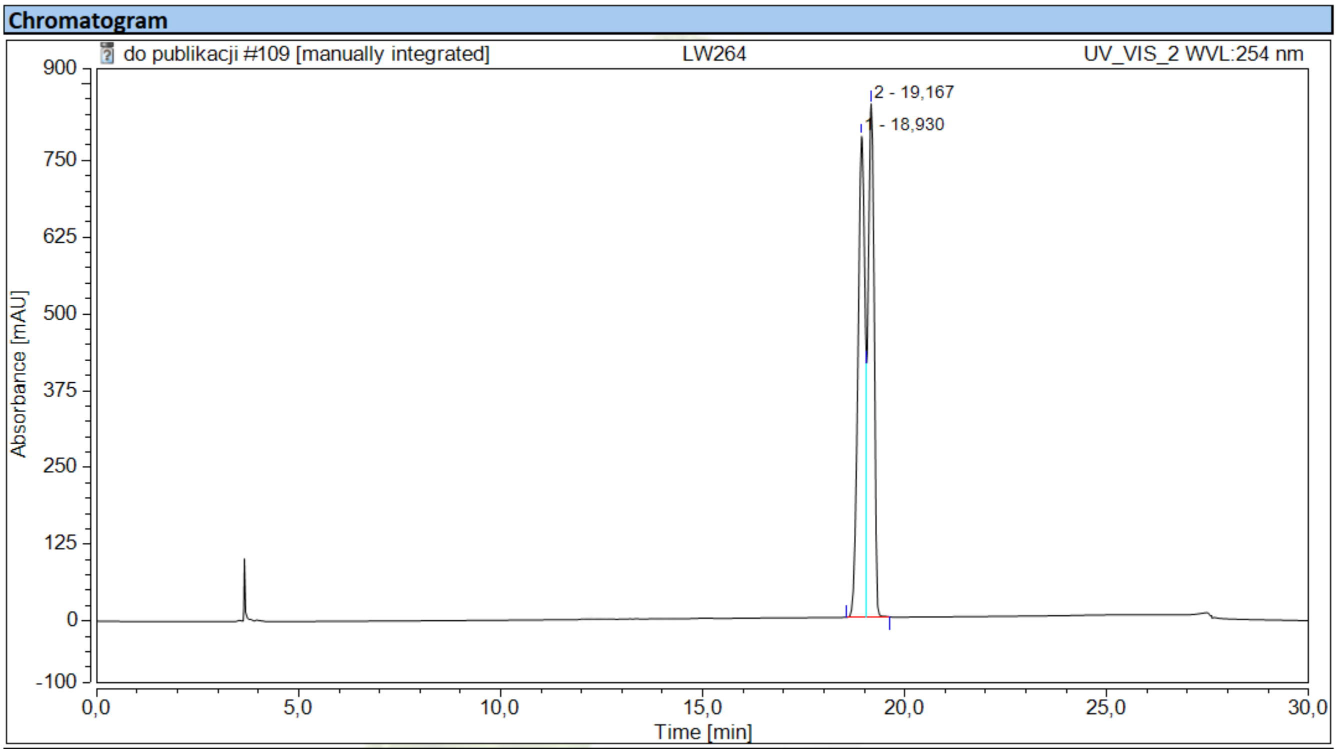

Integration Results

No.	Peak Name	Retention Time min	Area mAU*min	Height mAU	Relative Area %	Relative Height %	Amount n.a.
1		18,930	157,219	783,143	52,23	48,37	n.a.
2		19,167	143,785	835,823	47,77	51,63	n.a.
**Total:**			**301,004**	**1618,966**	**100,00**	**100,00**	

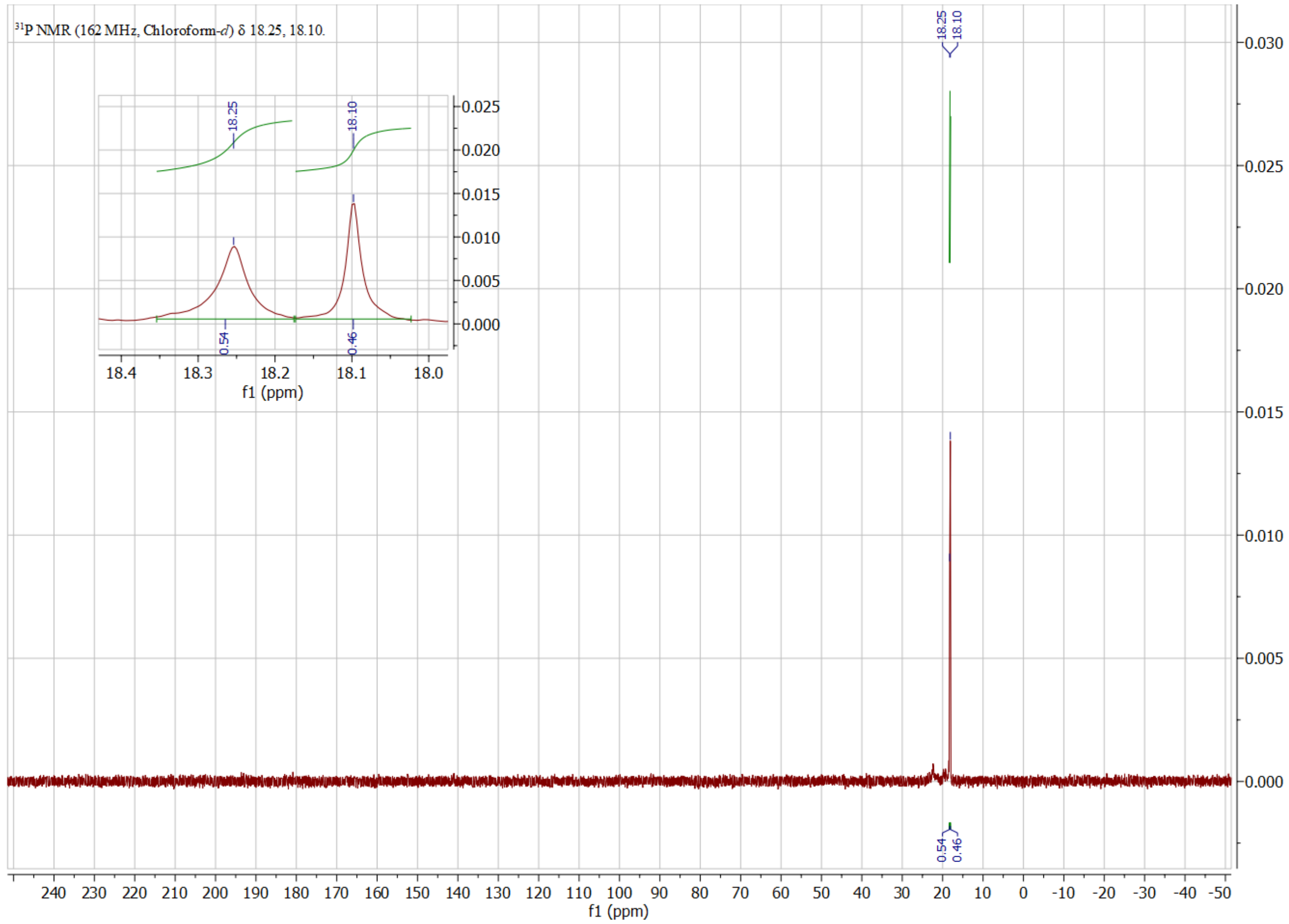
^{31}P NMR (162 MHz, Chloroform-*d*) δ 18.25, 18.10.
18.25
18.10
0.54
0.46
f1 (ppm)

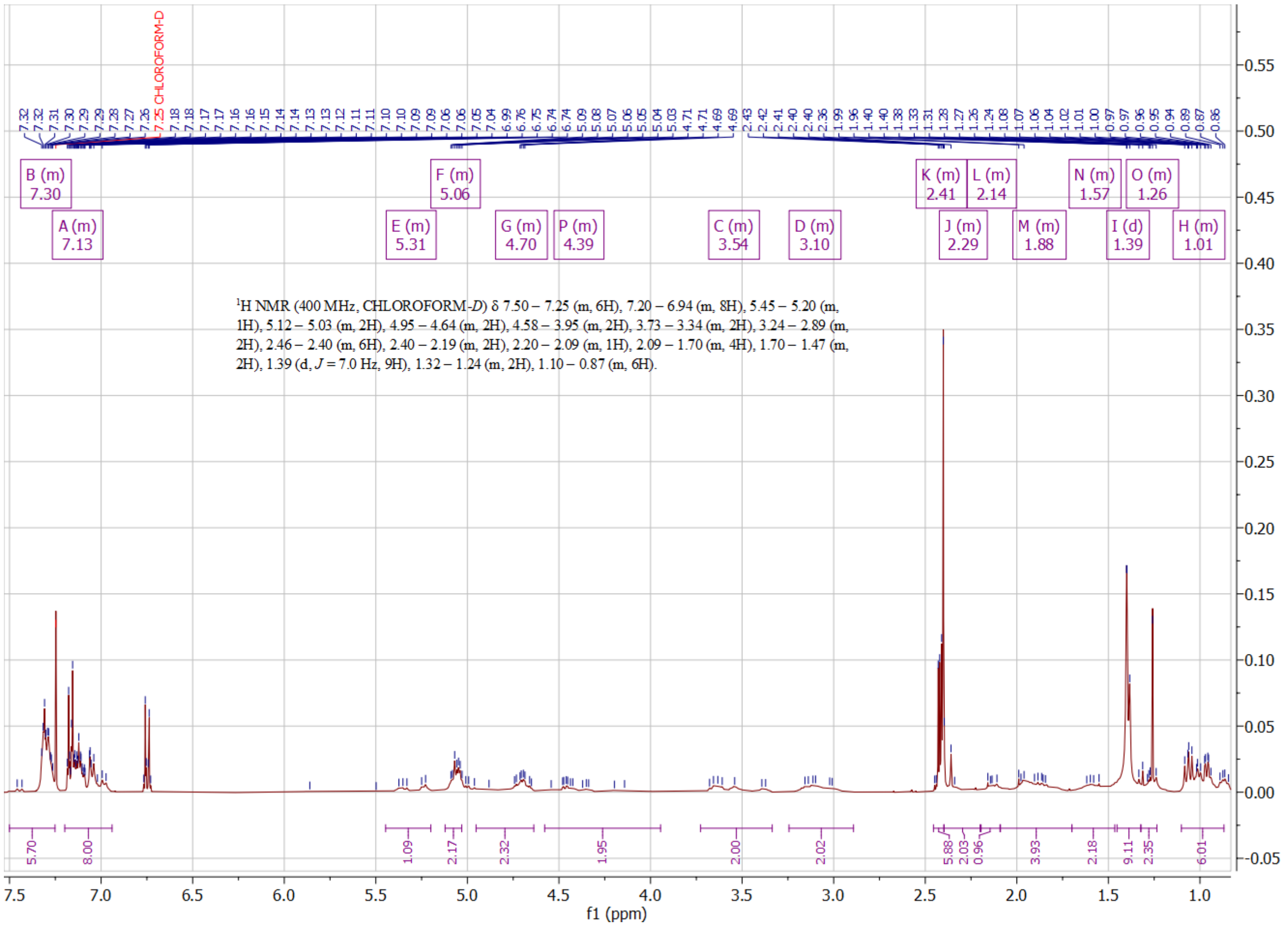
^{1}H NMR (400 MHz, CHLOROFORM-*D*) δ 7.50 – 7.25 (m, 6H), 7.20 – 6.94 (m, 8H), 5.45 – 5.20 (m, 1H), 5.12 – 5.03 (m, 2H), 4.95 – 4.64 (m, 2H), 4.58 – 3.95 (m, 2H), 3.73 – 3.34 (m, 2H), 3.24 – 2.89 (m, 2H), 2.46 – 2.40 (m, 6H), 2.40 – 2.19 (m, 2H), 2.20 – 2.09 (m, 1H), 2.09 – 1.70 (m, 4H), 1.70 – 1.47 (m, 2H), 1.39 (d, J = 7.0 Hz, 9H), 1.32 – 1.24 (m, 2H), 1.10 – 0.87 (m, 6H).
CHLOROFORM-D
f1 (ppm)

(110) Boc-D-Lys(Z)-L-Pro-Val^{P}(OPh-4-SCH$_3$)$_2$

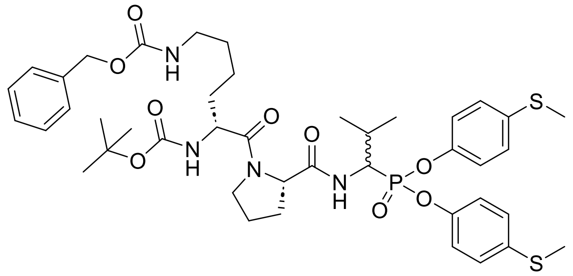

Name: benzyl *tert*-butyl ((5R)-6-((2S)-2-((1-(bis(4-(methylthio)phenoxy)phosphoryl)-2-methylpropyl)carbamoyl)pyrrolidin-1-yl)-6-oxohexane-1,5-diyl)dicarbamate

^{31}P NMR (162 MHz, Chloroform-d) δ 18.48 (s, 66%), 18.20 (s, 34%).

^{1}H NMR (400 MHz, Chloroform-d) δ 7.52 (dd, J = 44.3, 10.3 Hz, 1H, NH), 7.38 – 7.26 (m, 5H, 5xArH), 7.22 – 7.10 (m, 6H, 6xArH), 7.10 – 7.00 (m, 2H, 2xArH), 5.35 (dd, J = 24.7, 8.2 Hz, 1H, NH), 5.17 – 5.04 (m, 2H, CH$_2$), 4.93 – 4.85 (m, 1H, NH), 4.73 – 4.62 (m, 1H, CHP), 4.64 – 4.47 (m, 1H, CH), 4.49 – 4.32 (m, 1H, CH), 3.85 – 3.65 (m, 1H, 0.5xCH$_2$), 3.52 – 3.36 (m, 1H, 0.5xCH$_2$), 3.22 – 3.12 (m, 2H, CH$_2$), 2.43 (d, J = 3.1 Hz, 6H, 2xSCH$_3$), 2.40 – 2.22 (m, 2H, 0.5xCH$_2$, CH), 2.15 – 1.94 (m, 1H, 0.5xCH$_2$), 1.94 – 1.75 (m, 2H, CH$_2$), 1.75 – 1.39 (m, 6H, 3xCH$_2$), 1.35 (d, J = 18.2 Hz, 9H, Boc), 1.12 – 0.93 (m, 6H, 2xCH$_3$).

HRMS: $C_{42}H_{57}N_4O_9PS_2$ [M+Na]$^+$ calc. 879.3202 found 879.3180

HPLC: purity >99%; retention time 19.027 min

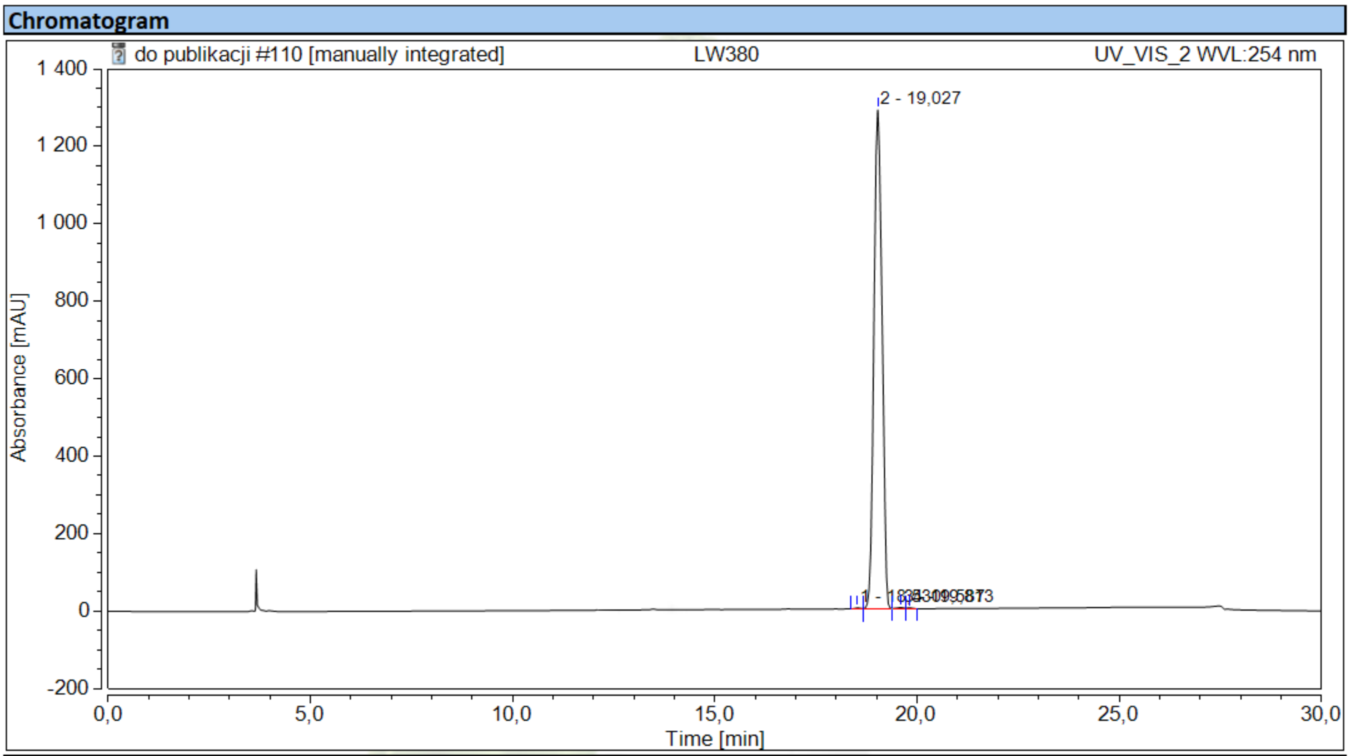

Integration Results

No.	Peak Name	Retention Time min	Area mAU*min	Height mAU	Relative Area %	Relative Height %	Amount n.a.
1		18,530	0,394	2,408	0,13	0,19	n.a.
2		19,027	300,981	1288,143	99,53	99,41	n.a.
3		19,587	0,636	2,927	0,21	0,23	n.a.
4		19,813	0,382	2,265	0,13	0,17	n.a.
**Total:**			**302,392**	**1295,743**	**100,00**	**100,00**	

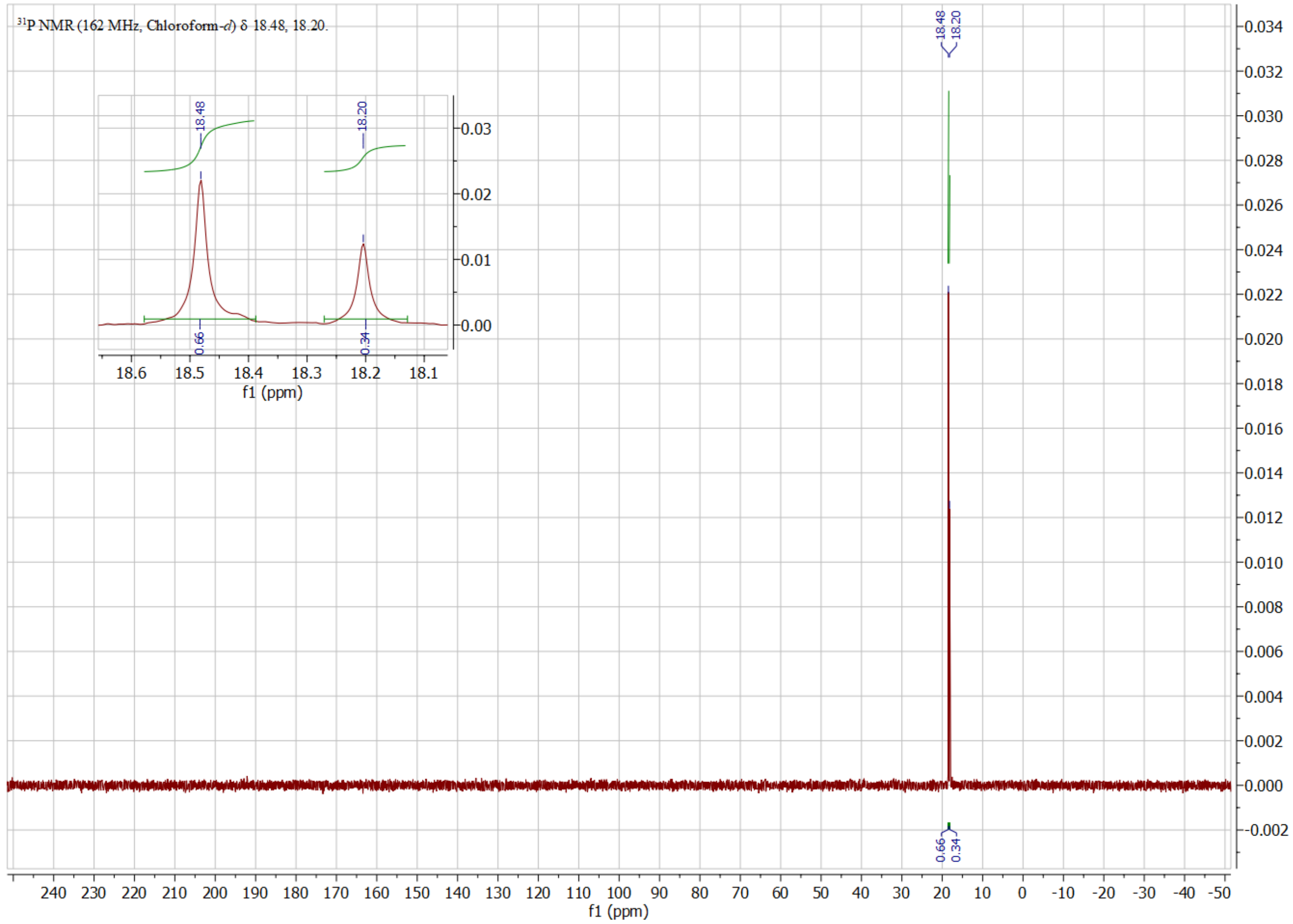
^{31}P NMR (162 MHz, Chloroform-*d*) δ 18.48, 18.20.
f1 (ppm)

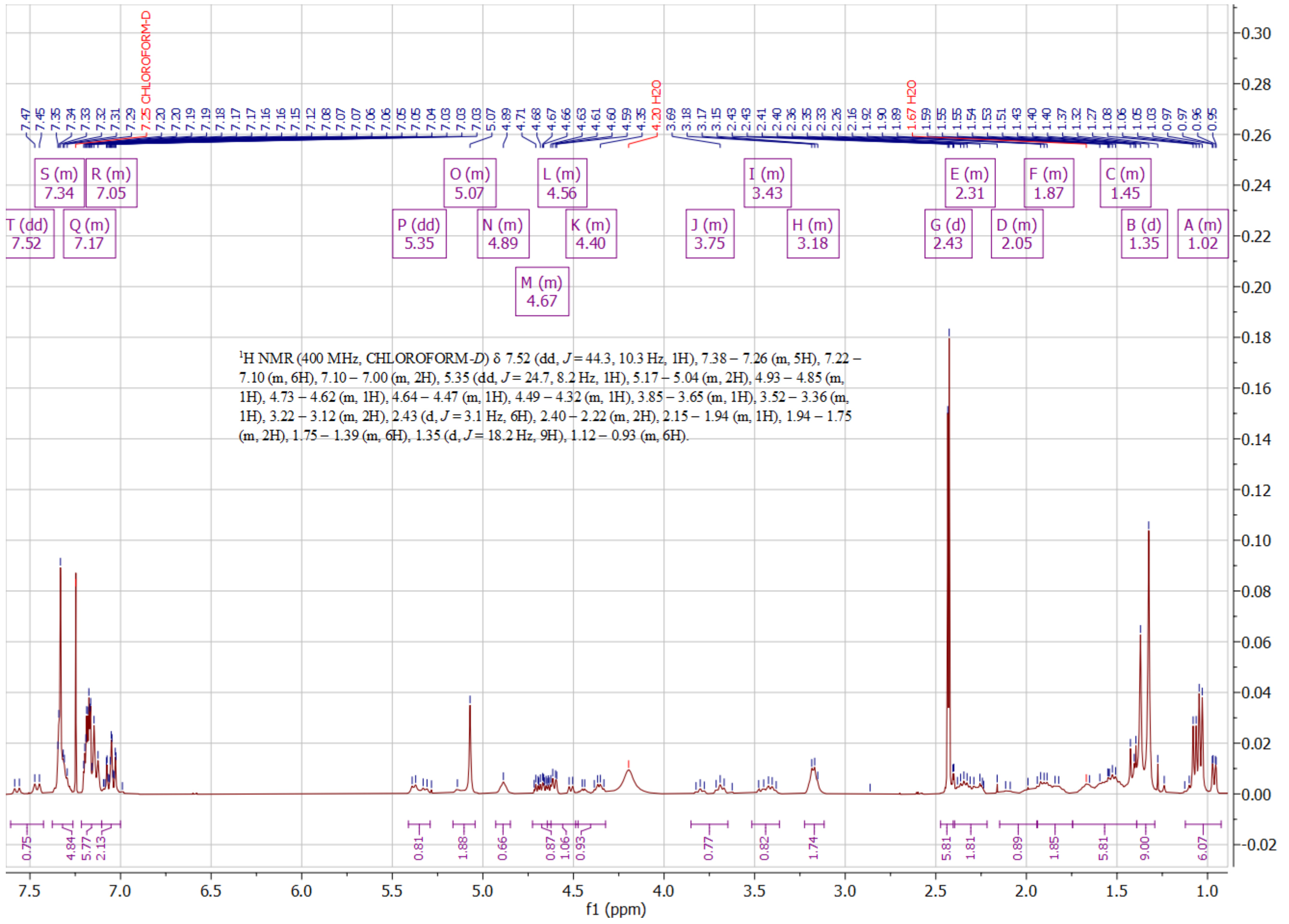
^{1}H NMR (400 MHz, CHLOROFORM-*D*) δ 7.52 (dd, *J* = 44.3, 10.3 Hz, 1H), 7.38 – 7.26 (m, 5H), 7.22 – 7.10 (m, 6H), 7.10 – 7.00 (m, 2H), 5.35 (dd, *J* = 24.7, 8.2 Hz, 1H), 5.17 – 5.04 (m, 2H), 4.93 – 4.85 (m, 1H), 4.73 – 4.62 (m, 1H), 4.64 – 4.47 (m, 1H), 4.49 – 4.32 (m, 1H), 3.85 – 3.65 (m, 1H), 3.52 – 3.36 (m, 1H), 3.22 – 3.12 (m, 2H), 2.43 (d, *J* = 3.1 Hz, 6H), 2.40 – 2.22 (m, 2H), 2.15 – 1.94 (m, 1H), 1.94 – 1.75 (m, 2H), 1.75 – 1.39 (m, 6H), 1.35 (d, *J* = 18.2 Hz, 9H), 1.12 – 0.93 (m, 6H).
f1 (ppm)

(111) Hm-L-Met(O$_2$)-L-Pro- Val^P(OPh-4-SCH$_3$)$_2$

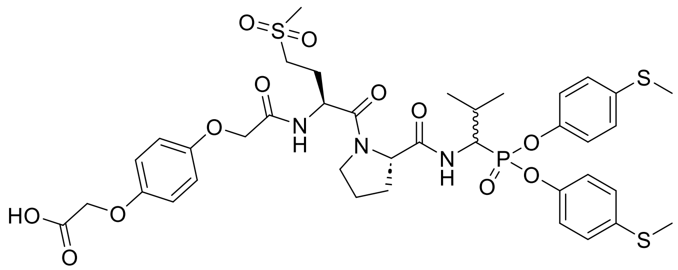

Name: 2-(4-(2-(((2S)-1-((2S)-2-((1-(bis(4-(methylthio)phenoxy)phosphoryl)-2-methylpropyl)carbamoyl)pyrrolidin-1-yl)-4-(methylsulfonyl)-1-oxobutan-2-yl)amino)-2-oxoethoxy)phenoxy)acetic acid

^{31}P NMR (243 MHz, Methanol-*d*$_4$) δ 18.45 (s, 47%), 18.20 (s, 53%).

^{1}H NMR (601 MHz, MeOD) δ 7.21 – 6.60 (m, 13H, 12xArH, NH), 4.87 – 4.81 (m, 1H, NH), 4.59 – 4.34 (m, 7H, 2xCH$_2$, CHP, 2xCH), 3.75 – 3.59 (m, 2H, CH$_2$), 3.10 – 2.98 (m, 2H, CH$_2$), 2.83 (d, J = 43.0 Hz, 3H, CH$_3$), 2.39 – 2.31 (m, 6H, 2xSCH$_3$), 2.32 – 2.15 (m, 2H, 0.5xCH$_2$, CH), 2.13 – 2.02 (m, 2H, CH$_2$), 2.00 – 1.65 (m, 3H, 1.5xCH$_2$), 1.07 (dd, J = 20.3, 6.9 Hz, 3H, CH$_3$), 1.01 (dd, J = 31.6, 6.6 Hz, 3H, CH$_3$).

HRMS: $C_{38}H_{48}N_3O_{12}PS_3$ [M+H]$^+$ calc. 866.2216 found 866.2238

HPLC: purity >99%; retention time 15.450 min

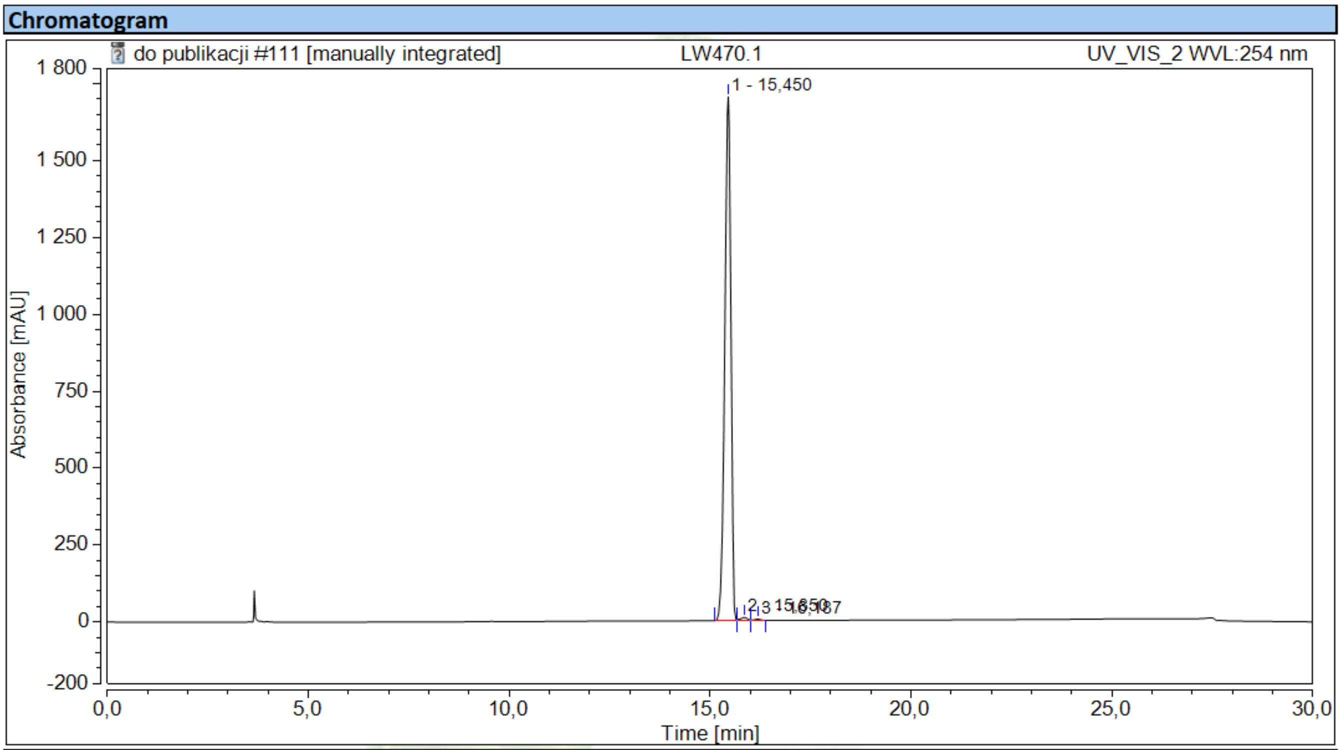

Integration Results

No.	Peak Name	Retention Time min	Area mAU*min	Height mAU	Relative Area %	Relative Height %	Amount n.a.
1		15,450	299,634	1703,150	99,08	99,17	n.a.
2		15,850	2,051	10,168	0,68	0,59	n.a.
3		16,187	0,738	4,025	0,24	0,23	n.a.
**Total:**			**302,423**	**1717,343**	**100,00**	**100,00**	

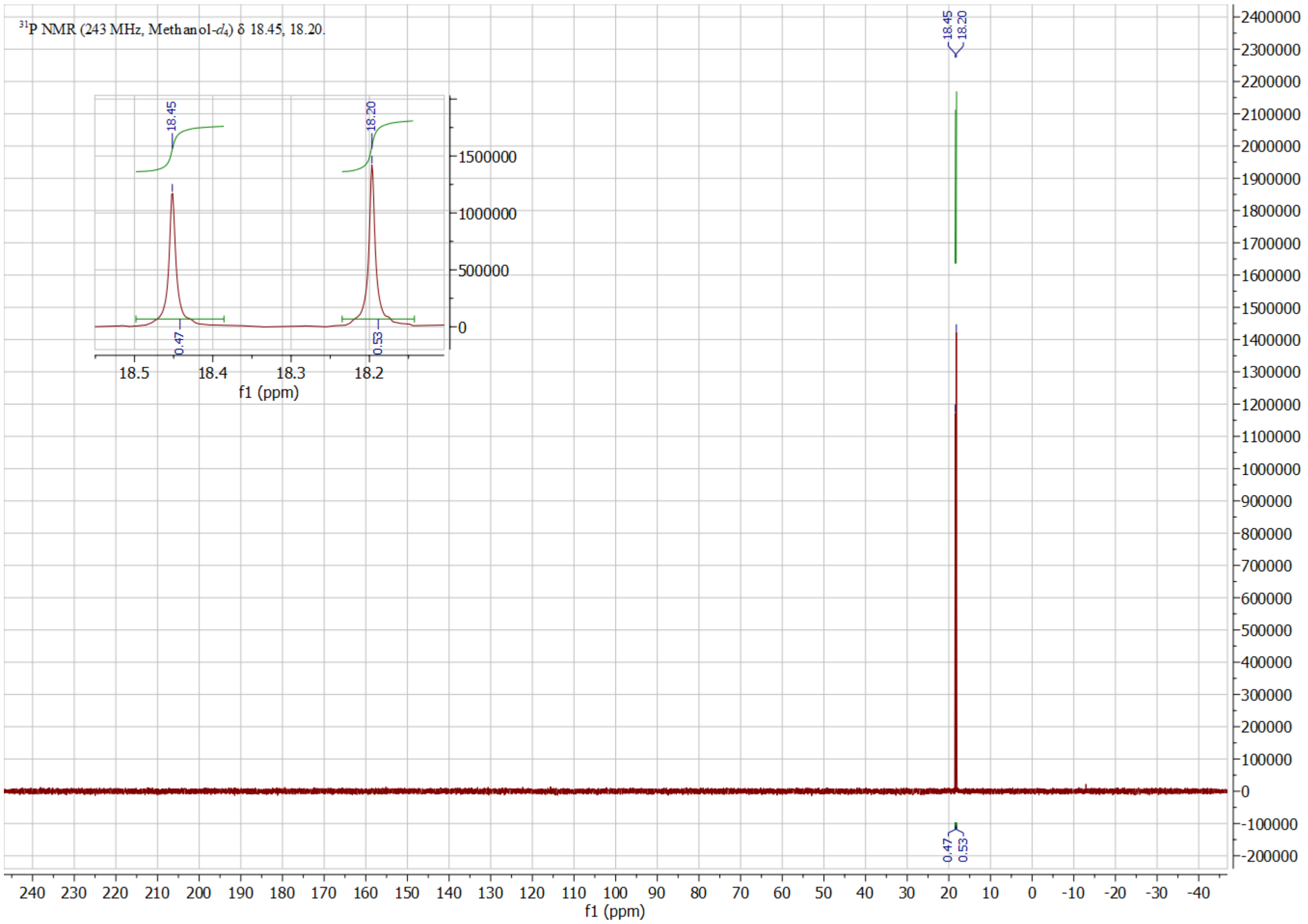
^{31}P NMR (243 MHz, Methanol-d_4) δ 18.45, 18.20.
f1 (ppm)

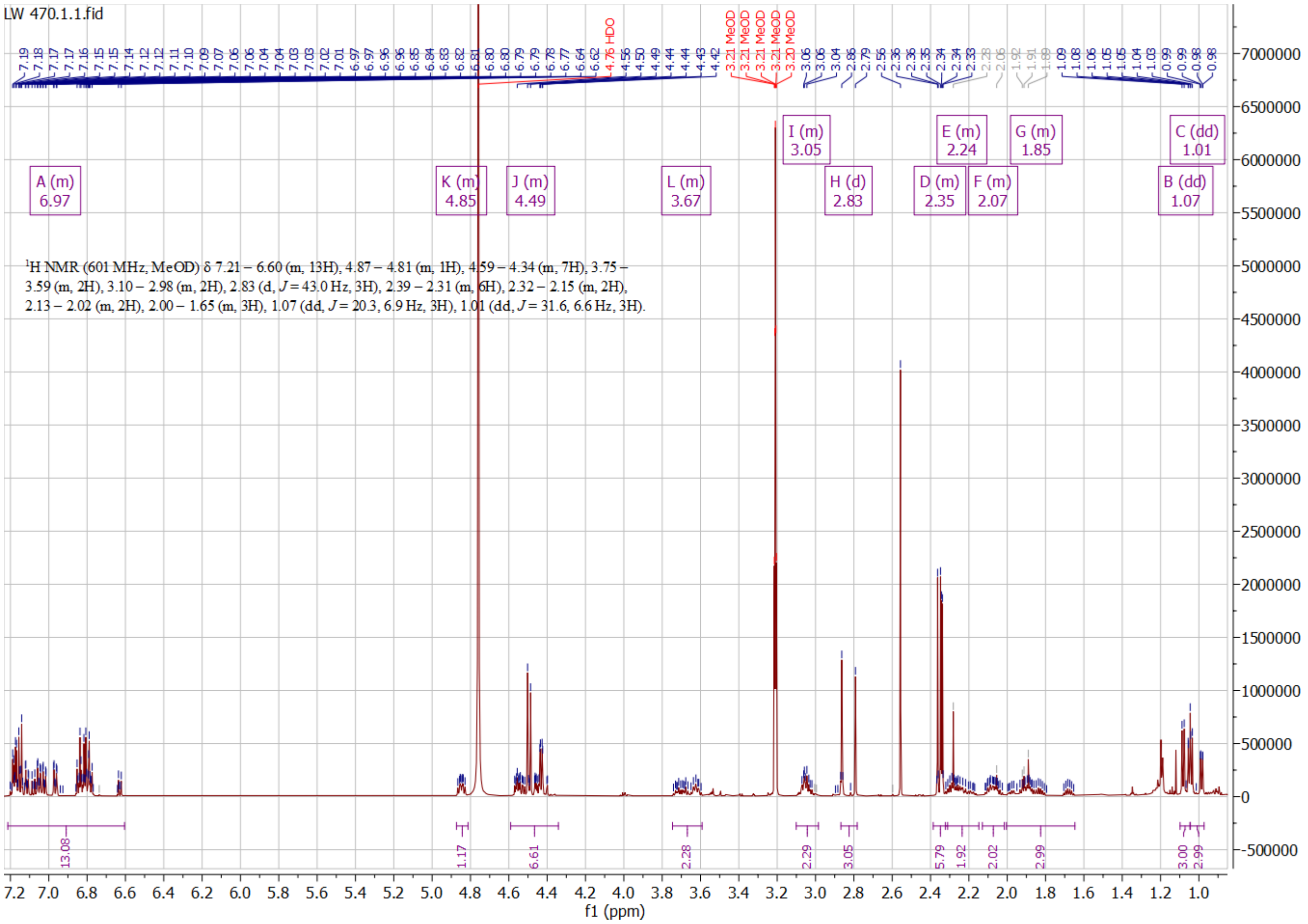
LW 470.1.1.fid
^{1}H NMR (601 MHz, MeOD) δ 7.21 – 6.60 (m, 13H), 4.87 – 4.81 (m, 1H), 4.59 – 4.34 (m, 7H), 3.75 – 3.59 (m, 2H), 3.10 – 2.98 (m, 2H), 2.83 (d, J = 43.0 Hz, 3H), 2.39 – 2.31 (m, 6H), 2.32 – 2.15 (m, 2H), 2.13 – 2.02 (m, 2H), 2.00 – 1.65 (m, 3H), 1.07 (dd, J = 20.3, 6.9 Hz, 3H), 1.01 (dd, J = 31.6, 6.6 Hz, 3H).
f1 (ppm)

(112) Hm-L-Met(O$_2$)-L-Pro-D-Val^P(OPh-4-SCH$_3$)$_2$

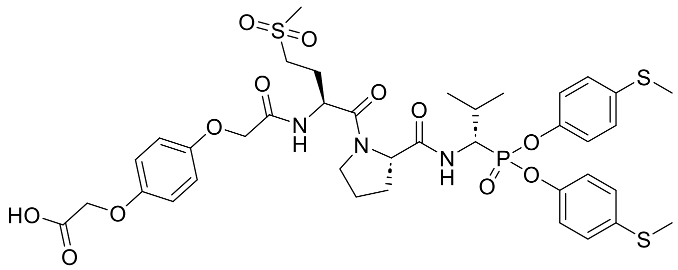

Name: 2-(4-(2-(((S)-1-((S)-2-(((S)-1-(bis(4-(methylthio)phenoxy)phosphoryl)-2-methylpropyl)carbamoyl)pyrrolidin-1-yl)-4-(methylsulfonyl)-1-oxobutan-2-yl)amino)-2-oxoethoxy)phenoxy)acetic acid

^{31}P NMR (243 MHz, MeOD) δ 18.45 (s).

^{1}H NMR (601 MHz, MeOD) δ 7.21 – 6.61 (m, 13H, 12xArH, NH), 4.85 (dd, J = 7.6, 5.5 Hz, 1H, NH), 4.61 – 4.36 (m, 7H, 2xCH$_2$, CHP, 2xCH), 3.75 – 3.69 (m, 1H, 0.5xCH$_2$), 3.66 – 3.60 (m, 1H, 0.5xCH$_2$), 3.09 – 2.99 (m, 2H, CH$_2$), 2.79 (s, 3H, CH$_3$), 2.34 (d, J = 3.0 Hz, 6H, 2xSCH$_3$), 2.32 – 2.18 (m, 2H, 0.5xCH$_2$, CH), 2.17 – 2.03 (m, 2H, CH$_2$), 2.03 – 1.80 (m, 3H, 1.5xCH$_2$), 1.04 (d, J = 6.8 Hz, 3H, CH$_3$), 0.99 (d, J = 6.8 Hz, 3H, CH$_3$).

HRMS: $C_{38}H_{48}N_3O_{12}PS_3$ $[M+H]^+$ calc. 866.2216 found 866.2233

HPLC: purity >99%; retention time 15.407 min

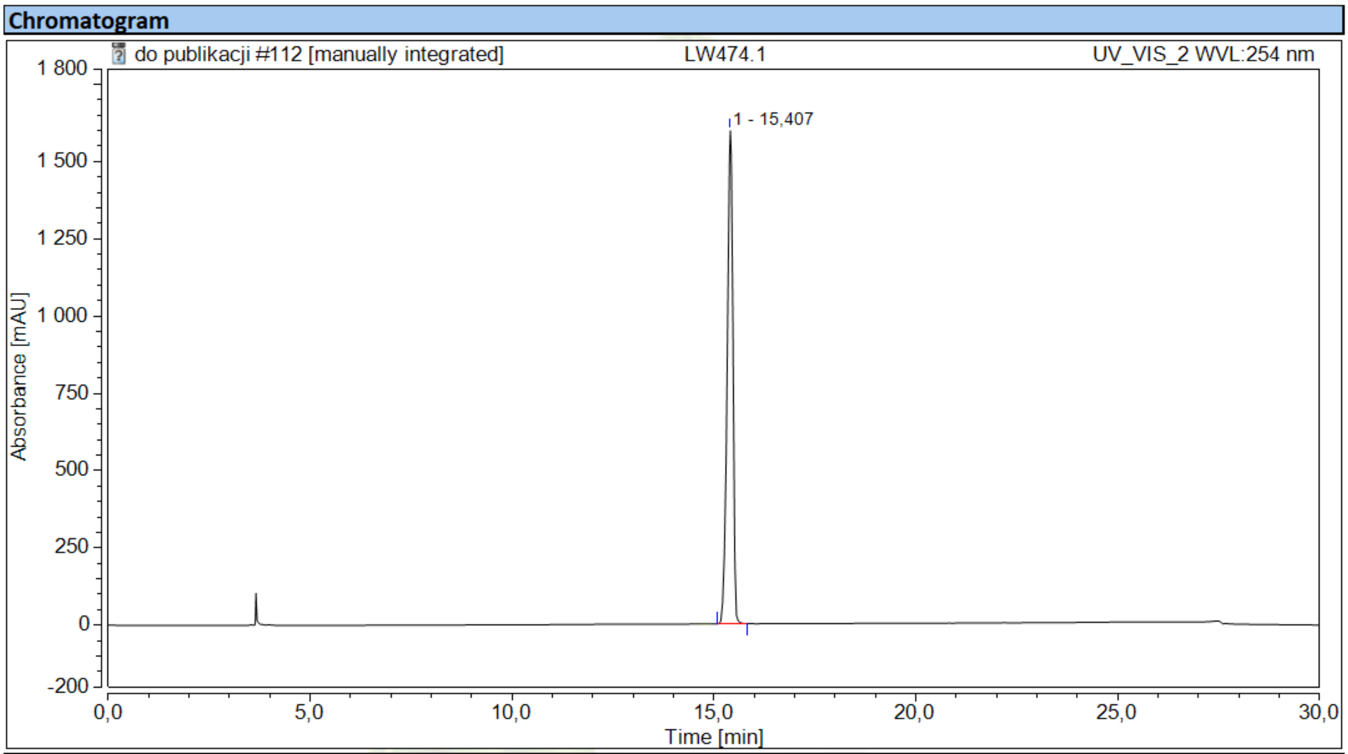

Integration Results

No.	Peak Name	Retention Time min	Area mAU*min	Height mAU	Relative Area %	Relative Height %	Amount n.a.
1		15,407	260,576	1595,347	100,00	100,00	n.a.
**Total:**			**260,576**	**1595,347**	**100,00**	**100,00**	

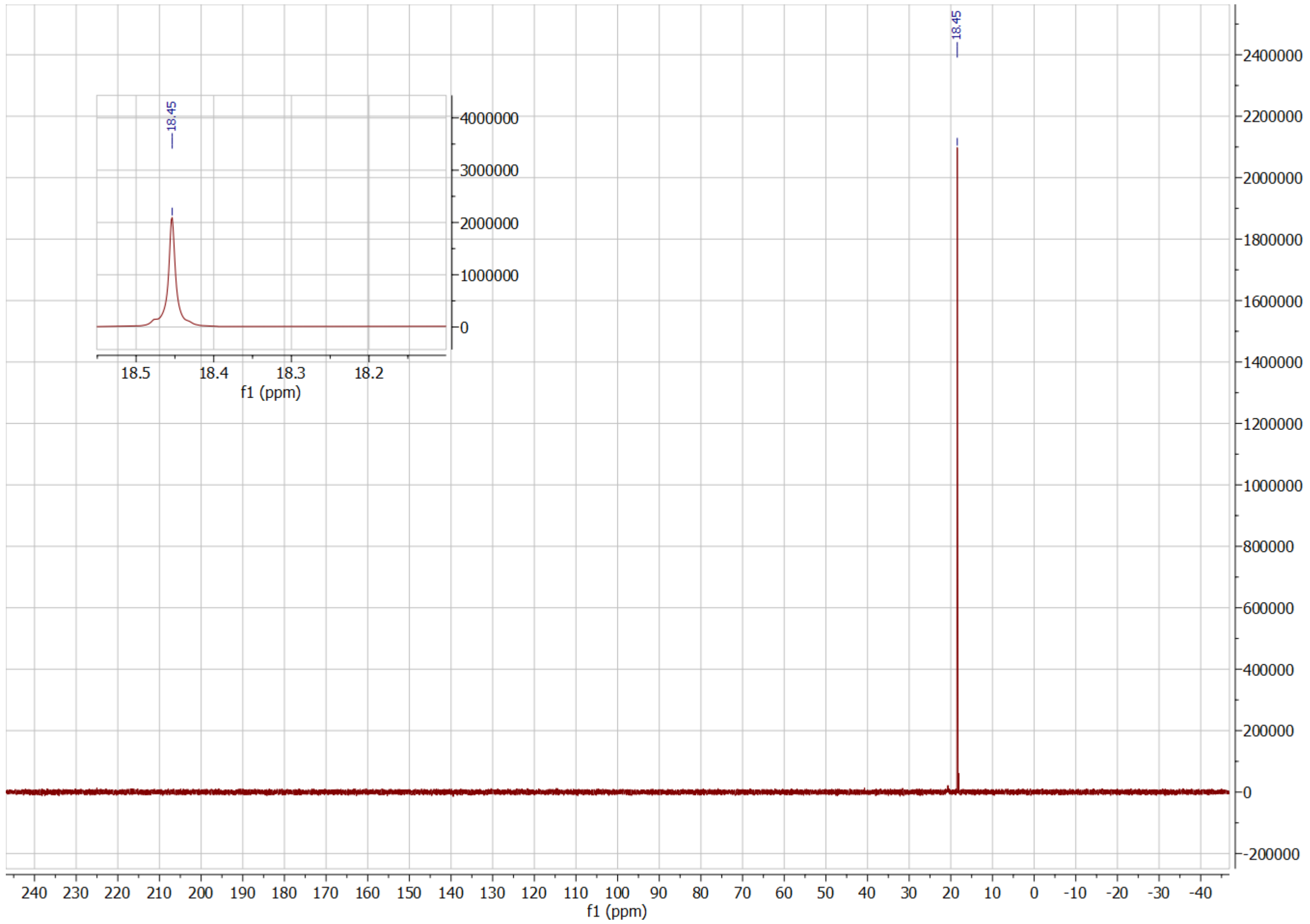
18.45
f1 (ppm)

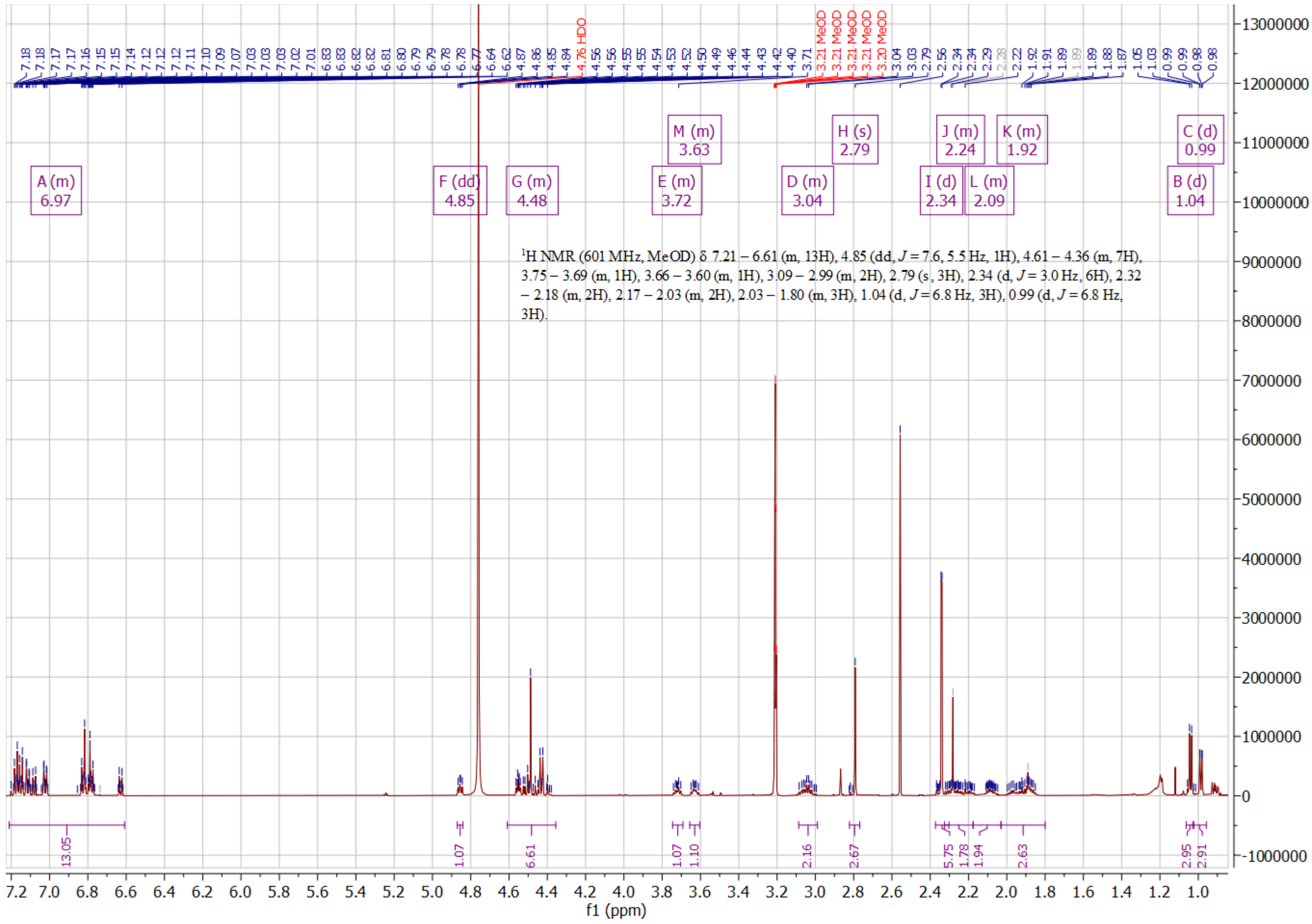
^{1}H NMR (601 MHz, MeOD) δ 7.21 – 6.61 (m, 13H), 4.85 (dd, J = 7.6, 5.5 Hz, 1H), 4.61 – 4.36 (m, 7H), 3.75 – 3.69 (m, 1H), 3.66 – 3.60 (m, 1H), 3.09 – 2.99 (m, 2H), 2.79 (s, 3H), 2.34 (d, J = 3.0 Hz, 6H), 2.32 – 2.18 (m, 2H), 2.17 – 2.03 (m, 2H), 2.03 – 1.80 (m, 3H), 1.04 (d, J = 6.8 Hz, 3H), 0.99 (d, J = 6.8 Hz, 3H).
f1 (ppm)

(113) Hm-L-Met(O2)-L-Pro-L-ValP(OPh-4-SCH3)2

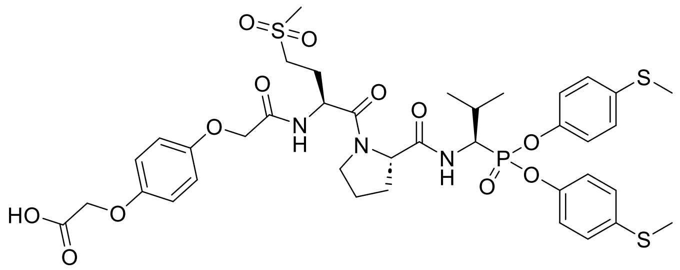

Name: 2-(4-(2-(((S)-1-((S)-2-(((R)-1-(bis(4-(methylthio)phenoxy)phosphoryl)-2-methylpropyl)carbamoyl)pyrrolidin-1-yl)-4-(methylsulfonyl)-1-oxobutan-2-yl)amino)-2-oxoethoxy)phenoxy)acetic acid

^{31}P NMR (243 MHz, MeOD) δ 18.20 (s).

^{1}H NMR (601 MHz, MeOD) δ 7.24 – 6.73 (m, 13H, 12xArH, NH), 4.85 – 4.82 (m, 1H, NH), 4.60 – 4.35 (m, 7H, 2xCH2, CHP, 2xCH), 3.71 – 3.59 (m, 2H, CH2), 3.06 (t, J = 8.0 Hz, 2H, CH2), 2.86 (s, 3H, CH3), 2.36 (d, J = 9.1 Hz, 6H, 2xSCH3), 2.31 – 2.22 (m, 2H, 0.5xCH2, CH), 2.12 – 2.02 (m, 2H, CH2), 1.94 – 1.80 (m, 2H, CH2), 1.72 – 1.64 (m, 1H, 0.5xCH2), 1.08 (d, J = 6.8 Hz, 3H, CH3), 1.05 (d, J = 6.8 Hz, 3H, CH3).

HRMS: $C_{38}H_{48}N_3O_{12}PS_3$ $[M+H]^+$ calc. 866.2216 found 866.22338

HPLC: purity >99%; retention time 15.480 min

Chromatogram

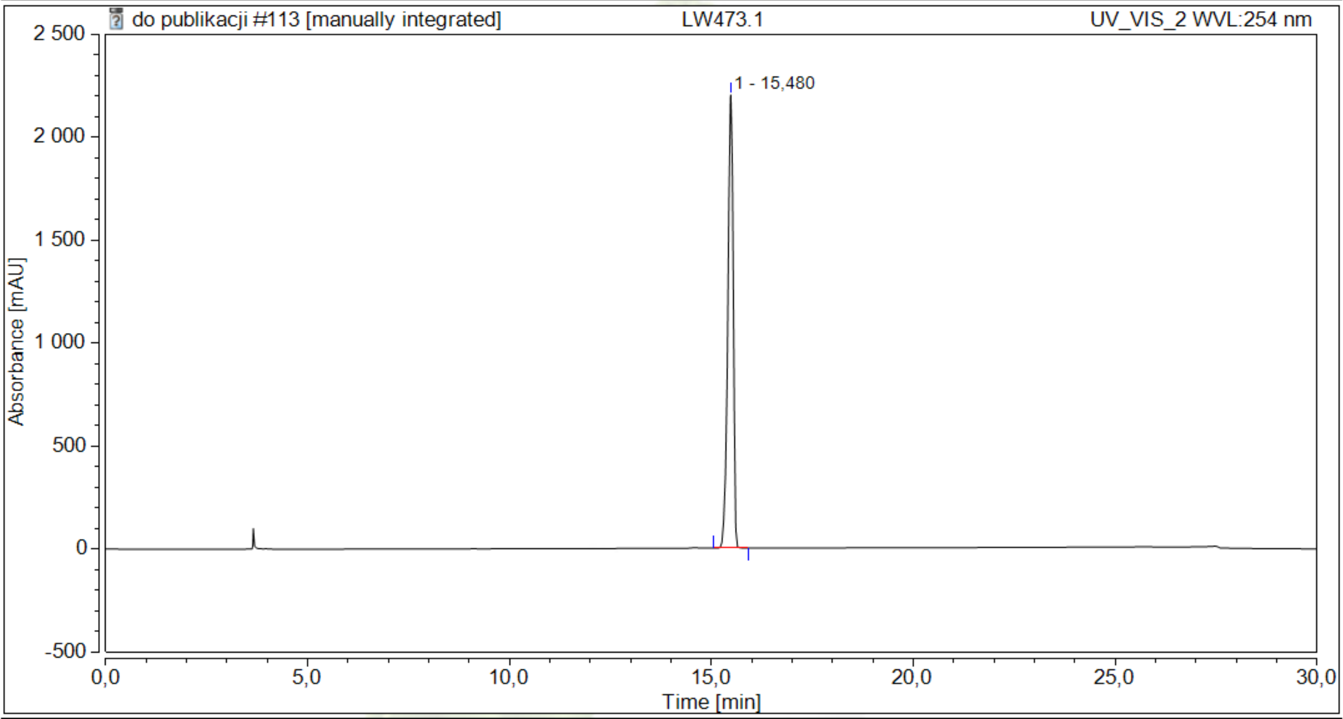

Integration Results

No.	Peak Name	Retention Time min	Area mAU*min	Height mAU	Relative Area %	Relative Height %	Amount n.a.
1		15,480	341,486	2200,731	100,00	100,00	n.a.
**Total:**			**341,486**	**2200,731**	**100,00**	**100,00**	

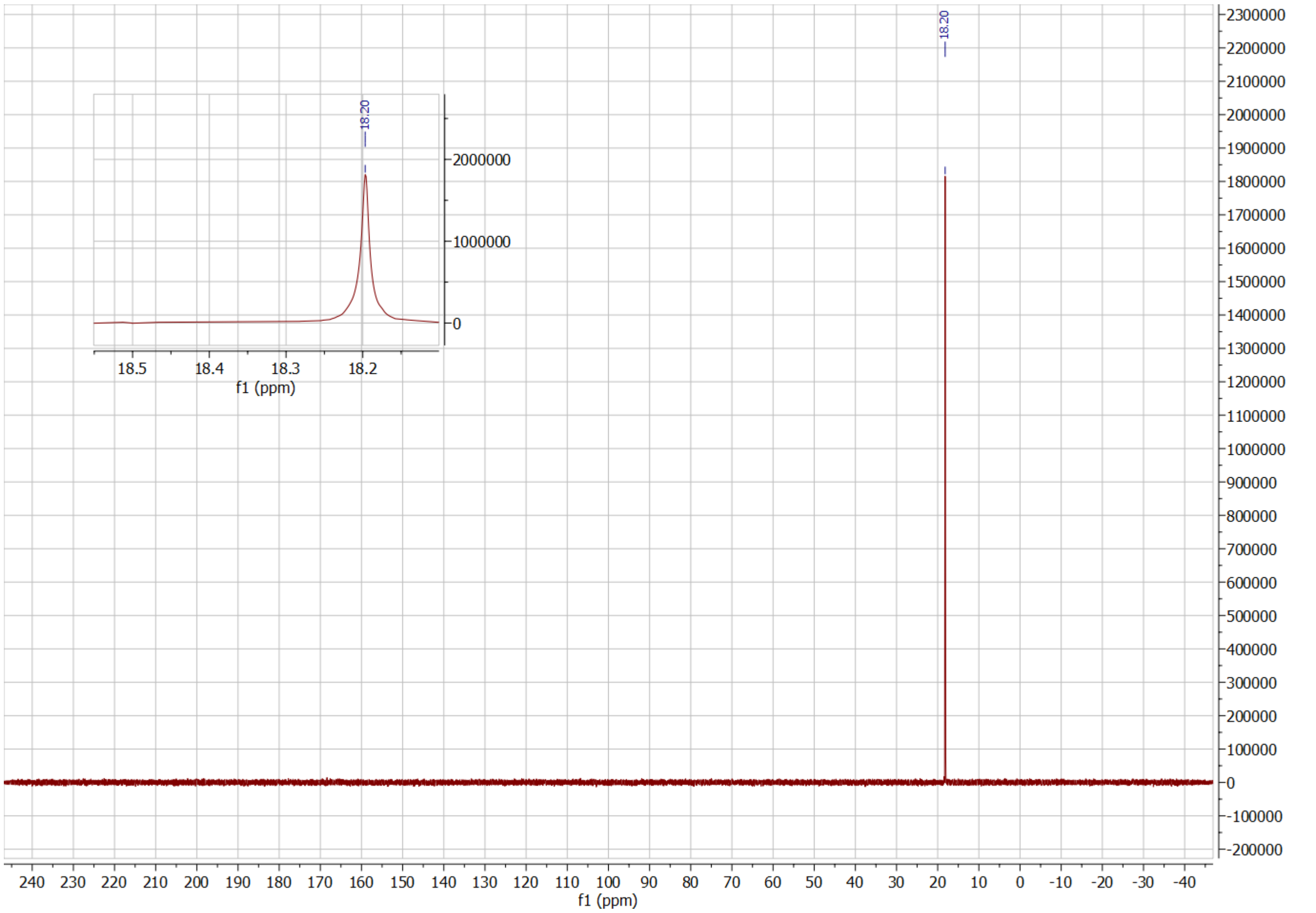
18.20
f1 (ppm)

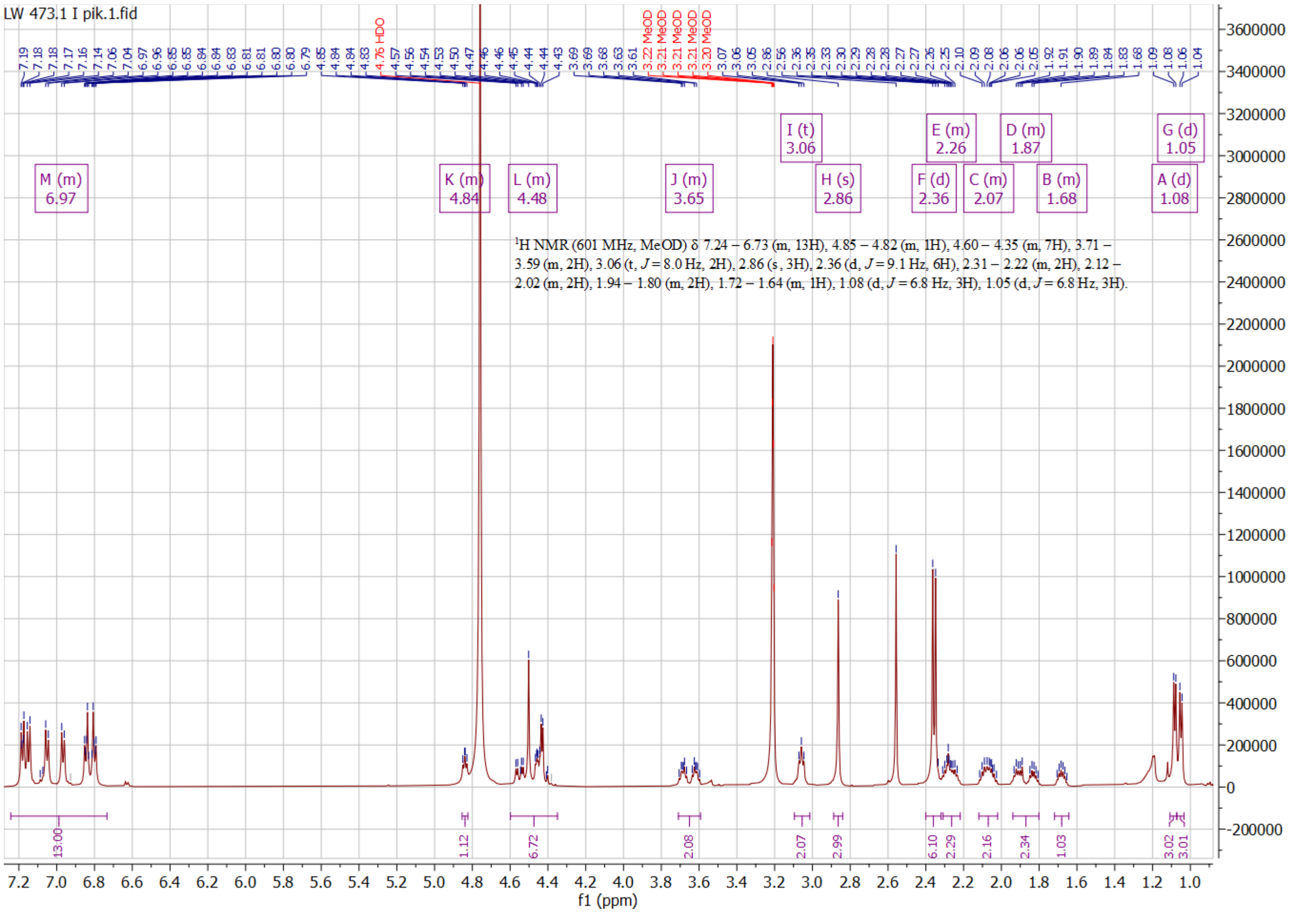
LW 473.1 I pik.1.fid
M (m) 6.97
K (m) 4.84
L (m) 4.48
J (m) 3.65
I (t) 3.06
H (s) 2.86
F (d) 2.36
E (m) 2.26
C (m) 2.07
D (m) 1.87
B (m) 1.68
A (d) 1.08
G (d) 1.05
^{1}H NMR (601 MHz, MeOD) δ 7.24 – 6.73 (m, 13H), 4.85 – 4.82 (m, 1H), 4.60 – 4.35 (m, 7H), 3.71 – 3.59 (m, 2H), 3.06 (t, J = 8.0 Hz, 2H), 2.86 (s, 3H), 2.36 (d, J = 9.1 Hz, 6H), 2.31 – 2.22 (m, 2H), 2.12 – 2.02 (m, 2H), 1.94 – 1.80 (m, 2H), 1.72 – 1.64 (m, 1H), 1.08 (d, J = 6.8 Hz, 3H), 1.05 (d, J = 6.8 Hz, 3H).
f1 (ppm)
